# Nickel-catalyzed asymmetric reductive arylation of α-chlorosulfones with aryl halides†

**DOI:** 10.1039/d1sc00283j

**Published:** 2021-02-19

**Authors:** Deli Sun, Guobin Ma, Xinluo Zhao, Chuanhu Lei, Hegui Gong

**Affiliations:** Center for Supramolecular Chemistry and Catalysis, College of Sciences, Shanghai University 99 Shang-Da Road Shanghai 200444 China hegui_gong@shu.edu.cn chlei@shu.edu.cn

## Abstract

We report an asymmetric Ni-catalyzed reductive cross-coupling of aryl/heteroaryl halides with racemic α-chlorosulfones to afford enantioenriched sulfones. The reaction tolerates a variety of functional groups under mild reaction conditions, which complements the current methods. The utility of this work was demonstrated by facile late-stage functionalization of commercial drugs.

## Abstract 2

In this work Ni-Catalyzed reductive cross-coupling between (hetero)aryl halides and racemic α-chlorosulfones to prepare enantioenriched α,α-disubstituted sulfones was demonstrated, allowing facile structural derivatization of drug precursors.

## Graphical abstract

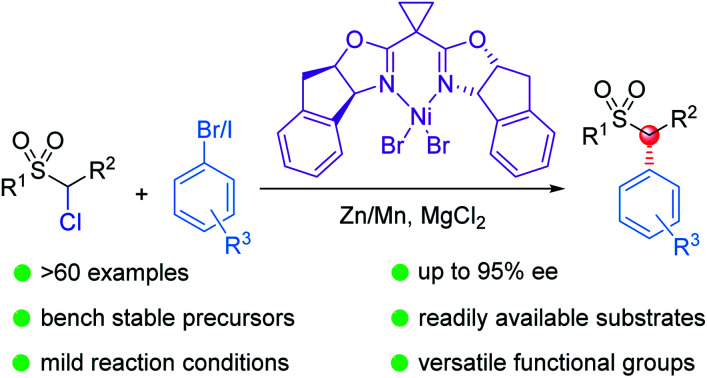

## Introduction

Sulfones are not only important functional units in synthetic chemistry^1^ but also unique compounds that have been widely studied in medicinal chemistry.^2^ Among them, a number of alkyl sulfones are FDA-approved drugs (*e.g.*, bicalutamide for prostate cancer and apremilast for psoriatic arthritis).^3^ Many efforts based upon transition-metal catalyzed coupling reactions have been devoted to the functionalization of the α-positions of alkylsulfones.^4–6^ However, asymmetric methods are largely confined to Tsuji–Trost allylation and Michael addition reactions by virtue of formation of relatively stable α-sulfonyl carbanions.^6^ Fu and co-workers have reported the first enantioselective Negishi coupling of α-bromosulfonamides and -sulfones with organozinc and organozirconium reagents to furnish secondary benzylic and allylic sulfonamides and sulfones in good yields and high levels of enantioselectivities.^7^ Asymmetric alkylation of α-bromosulfonamides with alkenes was later found to be effective under Ni–H–alkene insertion/alkylation conditions which afforded enantioenriched α-alkyl sulfones and sulfonamides.^8^ In both cases, the involvement of α-sulfonyl carbon radicals is thought to be key. Although this progress significantly advances the asymmetric functionalization of α-sulfone derivatives, there are a few issues remaining to be addressed. First, asymmetric introduction of heteroaromatics to the α-carbon of sulfones remains elusive.^8,9^ Second, the substrate scope may be limited with the current methods due to the use of organometallic nucleophiles and terminal alkenes.^7,8^ We envision that a cross-electrophile coupling strategy that utilizes readily accessible aryl electrophiles and α-halosulfones may otherwise exhibit different substrate scope and functional group tolerance compared to the reported methods.^10^

In this work, we present a Ni-catalyzed reductive cross-coupling strategy for asymmetric arylation of α-chlorosulfones with aryl halides.^11–13^ The reaction offers expeditious access to enantioenriched sulfones with no need of pre-generating organozinc reagents, and displays broad substrate scope including a set of heteroaromatics with excellent functional group tolerance. To the best of our knowledge, this work represents the first asymmetric functionalization of α-sulfonyl compounds using the cross-electrophile coupling strategy that complements the reported Negishi protocol.

## Results and discussion

### Optimization of the reaction conditions

Our investigations began with the coupling between **1** and **2** using chiral bis(oxazoline) ligands (Table 1).^14^ An extensive screening of reaction parameters revealed that the use of NiBr_2_(dme) (10 mol%) and **L1** (20 mol%), Zn as the reductant and MgCl_2_ as the additive in DMA delivered sulfone **3** in 73% yield and 91% ee (Table 1, entry 1). Under the latter conditions, replacing **L1** with other chiral dibox ligands **L2–L6** did not result in better outcomes in terms of both yields and ee's (entries 2–6). The use of Mn to replace Zn diminished the ee (entry 7). MgCl_2_ appeared to be crucial for this transformation, whereas MgBr_2_ is much less effective, and TBAB gave trace amounts of the coupling product (entries 8–9).^15^ Change of other parameters such as solvent and the ratio of the coupling partners was not satisfactory (Table S1†).^14^ The addition of the pre-coordinated **L1**·NiBr_2_ complex (10 mol%) as the precatalyst to replace NiBr_2_(dme) (10 mol%) and **L1** (20 mol%) led to slightly higher yield and the same ee (entry 10).

**Table 1:** Optimization for the formation of **3**^a^

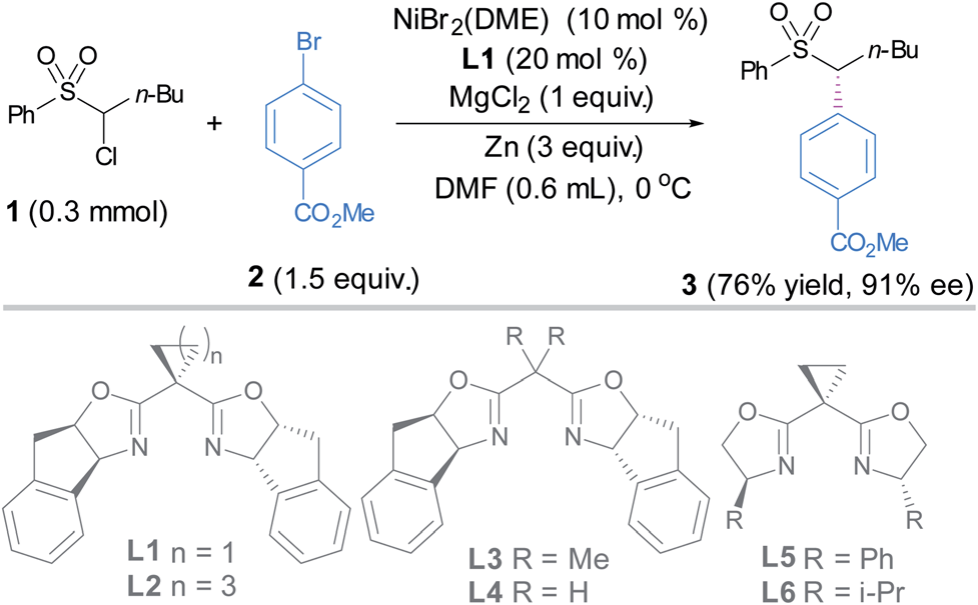		
Entry	Deviation from the standard conditions	Yield% (ee%)
1	No changes	73 (91)
2	**L2** (20%) instead of **L1**	71 (90)
3	**L3** (20%) instead of **L1**	28 (88)
4	**L4** (20%) instead of **L1**	34 (40)
5	**L5** (20%) instead of **L1**	65 (88)
6	**L6** (20%) instead of **L1**	31 (77)
7	Mn instead of Zn	74 (88)
8	TBAB instead of MgCl_2_	Trace (NA)
9	MgBr_2_ instead of MgCl_2_	63 (55)
10	**L1**·NiBr_2_ (10%)	76 (91)

a Reactions are conducted under N_2_ on a 0.15 mmol scale for 48 h; NMR yield is obtained using 2,5-dimethyl furan as the internal standard, ee is determined by chiral HPLC analysis, DMF = *N*,*N*-dimethyl formamide.

We tentatively speculate that **L1** adopting a rigid cyclopropyl linker features a larger bond angle of the C(oxazoline)–C(methylene)–C(oxazoline) bond than those in **L2–L4** (entries 1–4), which appears to have a crucial effect on the enantioselectivity. On the other hand, by comparing the results arising from **L1**, **L2** and **L5** (entries 1, 2 and 5), it is reasonable to conclude that the rigid tricyclic fused rings in **L1** and **L2** also fulfill an optimal steric and rigidity requirement for enantioselective control.

### Substrate scope

**Fig. 1:**
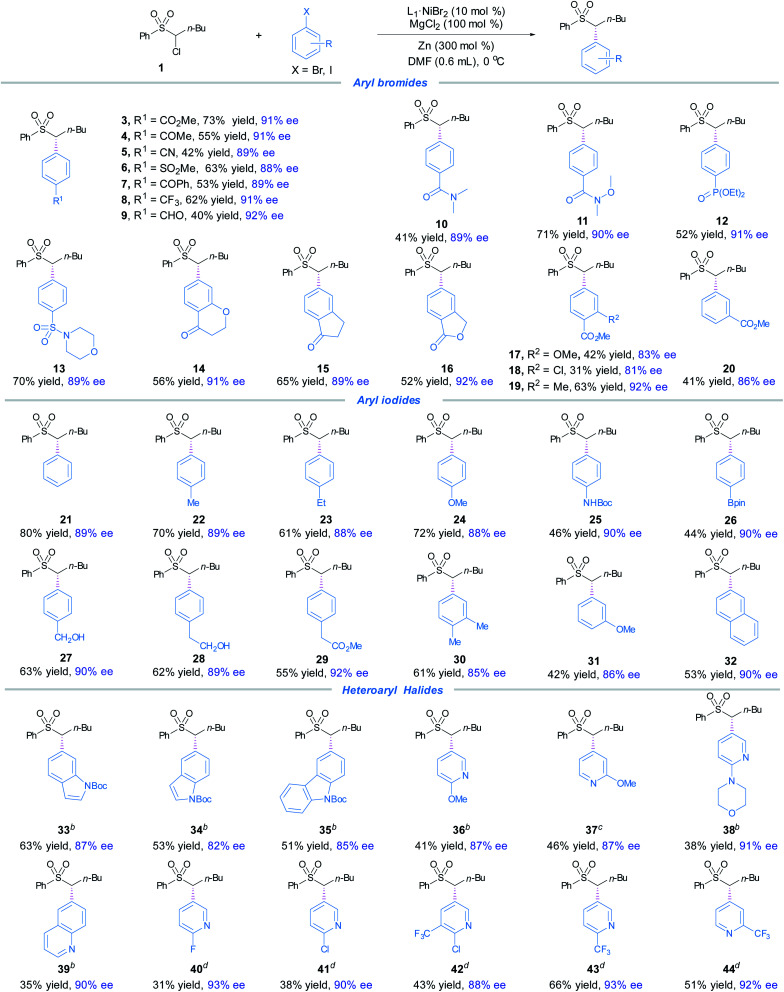
The scope of aryl halides. ^*a*^The standard reaction conditions; isolated yields are provided; ee is determined by chiral HPLC analysis. ^*b*^Mn instead of Zn, 1.5 equiv. heteroaryl iodide used. ^*c*^Mn instead of Zn, 1.5 equiv. heteroaryl bromide used. ^*d*^Mn instead of Zn, 2.0 equiv. heteroaryl bromide used, dioxane is used as the solvent, rt.

The optimized conditions (Table 1, entry 10) proved to be effective for the preparation of a wide range of chiral α,α-disubstituted sulfones **4–32** (Fig. 1). A variety of aryl bromides bearing electron-withdrawing groups underwent smooth cross-coupling with **1** to furnish the α,α-disubstituted sulfones **4–20** in moderate to good yields and high levels of enantioselectivities (Fig. 1). The functional groups included aryl esters (**3**, **16–20**), electron-poor acetones (**4**, **7**, **14–15**), aldehyde (**9**), amides (**10–11**), phosphonate (**12**), ethers (**14**, **17**), sulfone (**6**), sulphonamide (**13**), and aryl chloride (**18**). For electron-neutral and -rich arenes, aryliodides were found to effectively couple with **1**, furnishing **21–32** in good yields and high ees. The competent functional groups included boronates (**26**), ethers (**24**, **31**) and ester (**29**).

It was noted that amide (**25**) and alcohols (**27–28**) with acidic protons were compatible. Lastly, 2-iodonaphthalene gave the product **32** in 53% yield and 90% ee. Next, a range of heteroaryl halides were subjected to the coupling conditions (Fig. 1). By replacing Zn with Mn, the slightly modified reaction conditions effectively accommodated electron-rich indole, 9*H*-carbazole, quinolone and electron-deficient pyridine moieties to afford enantioenriched α,α-disubstituted products **33–34**. By selection of suitable aryl iodides and bromides as the coupling partners, indole, 9*H*-carbazole and pyridine bearing electron-rich substituents afforded **33–36** and **38**, whereas pyridine bearing electron-deficient groups delivered **40–44** in high ees. This asymmetric protocol also exhibited excellent chemoselectivity toward bromides/iodides, leaving (hetero)aryl chlorides untouched (**18**, **41–42**).

**Fig. 2:**
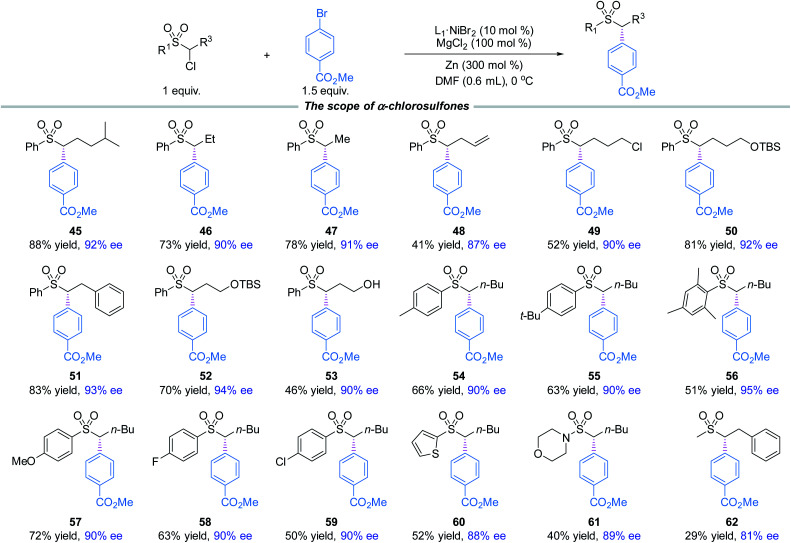
The standard reaction conditions; isolated yields are provided; ee is determined by chiral HPLC analysis.

Moreover, a broad scope of substituted α-chlorosulfones were investigated for the coupling with methyl 4-bromobenzoate (**2**) (Fig. 2). The coupling products (**45–62**) were produced in moderate to good yields and high enantioselectivities. Notable functional groups included vinyl and hydroxyl. The aryl groups attached to the sulfone moieties did not seem to have key effects on the enantioselectivities as evidenced in **54–60**. Although α-chloro-arylsulfone electrophiles were the primary focus of our research, α-chlorosulfonamide (**61**) and α-chloroalkylsulfone (**62**) were also investigated. The yields were modest and the enantioselectivities were reasonably good.

**Fig. 3:**
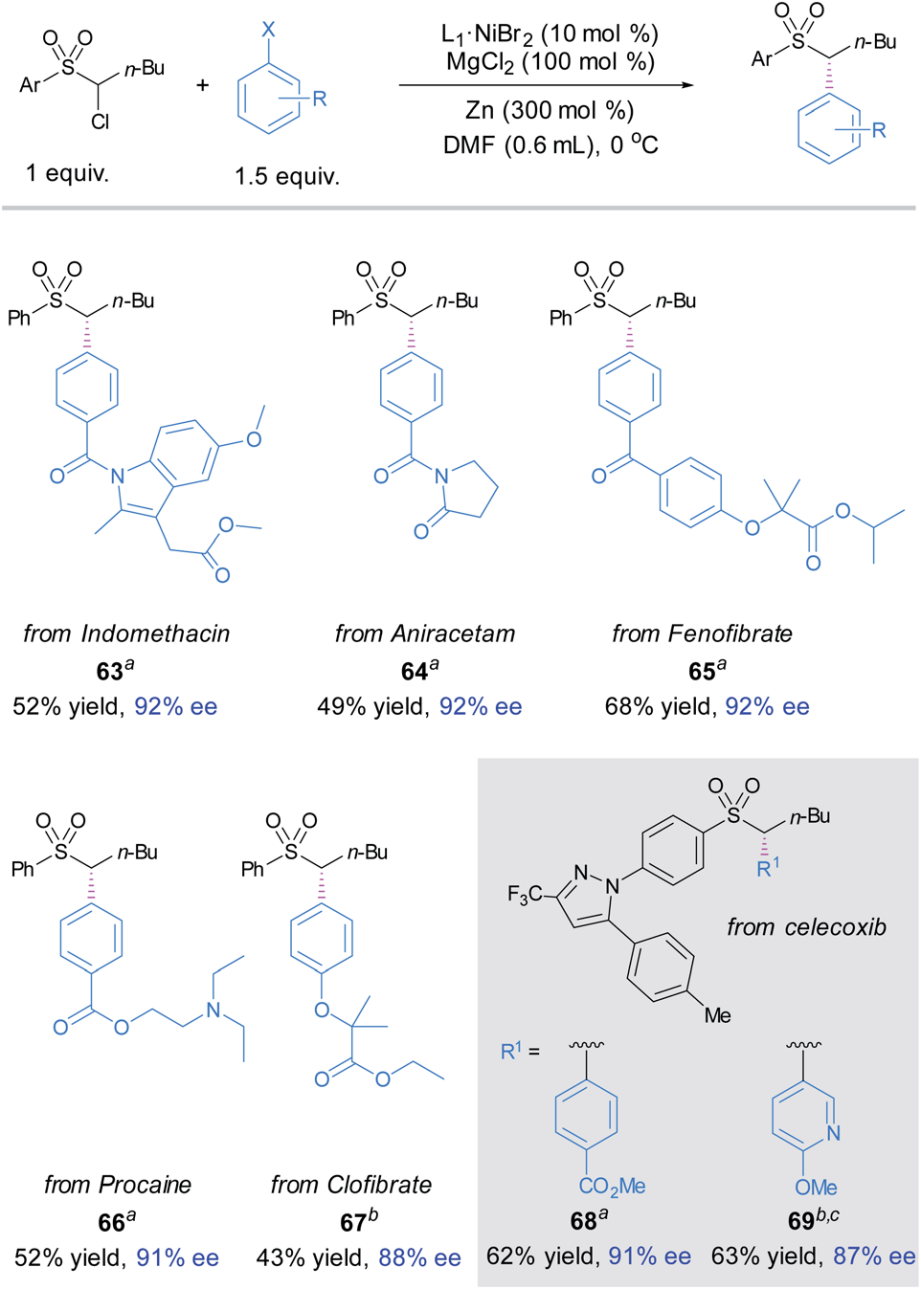
Late-stage functionalization. The standard reaction conditions; isolated yields are provided; ee is determined by chiral HPLC analysis. ^*a*^X = Br. ^*b*^X = I. ^*c*^Mn instead of Zn.

The applicability of this asymmetric α-sulfonyl arylation method was showcased by late-stage diversification of a number of commercial drugs. To our delight, the coupling of α-chlorosulfone **1** with bromo/iodo-substituted indomethacin, aniracetam, fenofibrate, procacine and clofibrate derivatives delivered the enantioenriched products **63–67** in high ees. Next, we examined the arylation of celecoxib-derived α-chlorosulfone; compounds **68** and **69** were obtained in modest yields and high enantioselectivities (Fig. 3).^16^

We have also examined the coupling of α-chlorosulfone **1** with 4-bromoanisole, and **1** with methyl 4-iodobenzoate. Both reactions gave the products in low yields (∼15%). The mass balance for the reaction with less reactive 4-bromoanisole includes hydro-dechlorination of **1** and a large amount of recovered 4-bromoanisole. In the case of methyl 4-iodobenzoate, we recovered substantial amounts of **1** and the aryl iodide. The first case (with 4-bromoanisole) can be easily attributed to the low reactivity of aryl bromide deactivated by the electron-donating group (MeO), leading to mismatched reactivity of the coupling partners with the Ni catalyst. The inhibition of the reaction when using more reactive methyl 4-iodobenzoate is surprising. We reason that a different competing path may operate, which prevents recycling of the Ni catalyst for the coupling process. Such a phenomenon is often seen in our previous studies, *e.g.*, recovery of aryl–I from the aryl–Ni(ii)–I complex.^17^ Thus, we speculate that the use of electron-poor aryl bromides and electron-neutral and -rich aryl iodides ensures matched reactivities of the coupling partners with the Ni catalyst.

### Mechanistic consideration

**Fig. 4:**
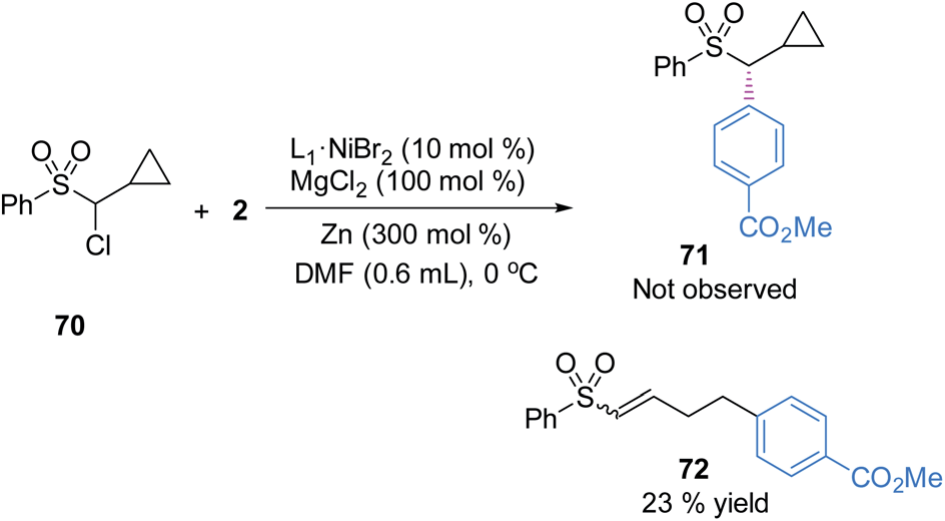
Radical clock.

To understand the possible mechanism of this transformation, several experiments were conducted. A cyclopropyl-containing substrate **70** was prepared and subjected to the standard reaction conditions. We did not detect the corresponding cyclopropane-retaining product **71**. Rather, only the ring-opening coupling product **72** was obtained in 23% yield (Fig. 4), which indicated the involvement of a radical ring-opening process.^12*c*,*d*,15^ The radical nature of the coupling event was further verified by the inhibition of the reaction of **1** and **2** with externally added TEMPO (1 equivalent), the yield of **3** dropped to 5% with 90% ee; no TEMPO-trapping adducts were detected.

In general, the coupling of an aryl electrophile with an alkyl halide follows a radical-chain mechanism, as initially proposed by Weix.^18^ One of the key experiments therein showed that a stable Ar–Ni(ii)–X complex can initiate radical formation by reacting with an alkyl iodide to generate the coupling product. The process involves halide abstraction to generate an alkyl radical and an Ar–Ni(iii)X_2_ intermediate. The radical then diffuses to bulk solution and finds Ar–Ni(ii)–X to form Ar–Ni(iii)X–alkyl, whose reductive elimination gives the product. Following this, we first conducted a catalytic reaction of **1** with methyl 4-bromobenzoate which generated racemic **3** in 65% yield along with the hydrodechlorination product **1-H** in 25% yield (eqn (1)). Next, we performed the stoichiometric reaction of Ar–Ni(ii)–Br (complex **73**) with **1** in DMF at 0 °C, and the racemic product **3** was obtained in 23% yield associated with recovered **1** (eqn (2)). Addition of MgCl_2_ and/or Zn increased the coupling yield of **3**. With Zn, reduction of **1** was observed. These results are in accordance with a radical-chain mechanism, in which the addition of an alkyl radical to Ar–Ni(ii)X is featured.1
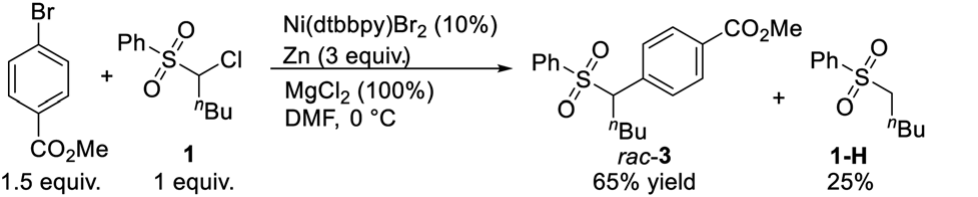
2
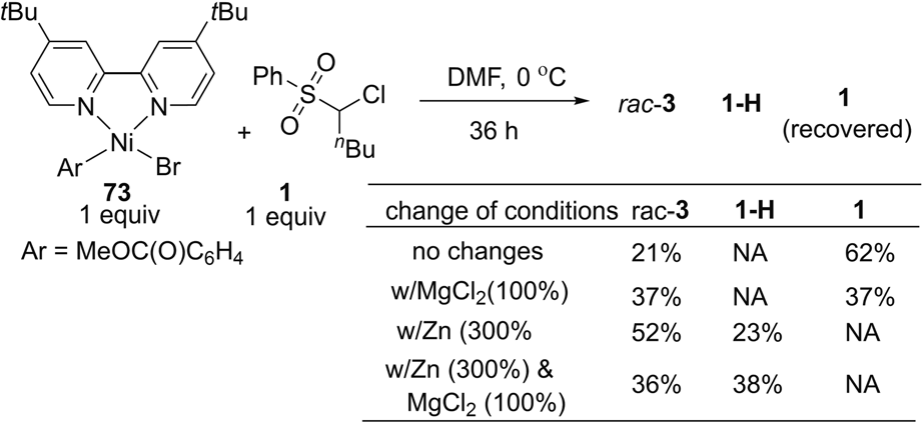


**Scheme 1:**
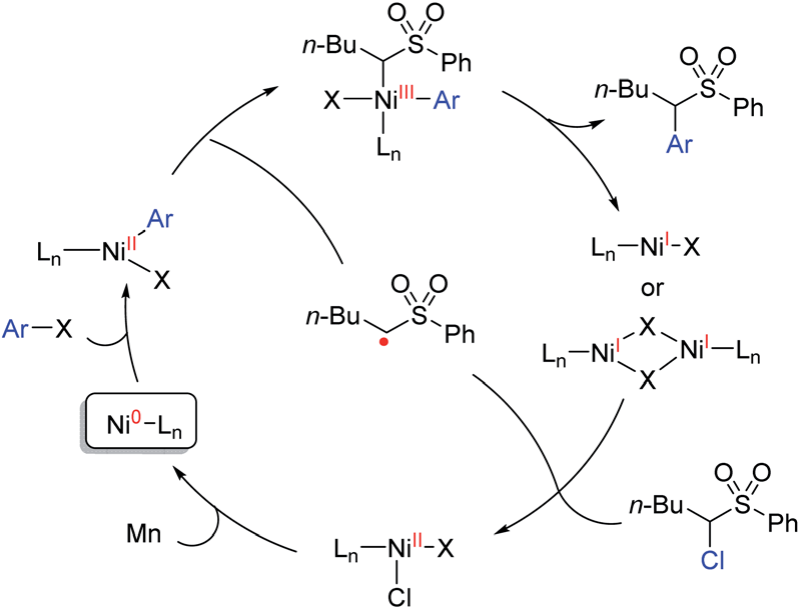
A working hypothesis of the reaction mechanism.

Thus, we propose a mechanism that the oxidative addition of an aryl halide to Ni(0) results in an Ar–Ni(ii)–X complex, which intercepts an α-sulfonyl radical to give the Ar–Ni(iii)X–alkyl complex, and upon reductive elimination, the product is omitted along with a Ni(i)X complex or a dimeric Ni(i) species.^19^ The latter species reacts with α-chlorosulfone to generate the alkyl radical and Ni(ii)ClX. The radical diffuses to the bulk solution to combine with the Ar–Ni(ii)X complex, whereas the Ni(ii)ClX is reduced to Ni(0) by Mn, allowing the catalytic cycle to continue (Scheme 1).

## Conclusions

In summary, we have developed a Ni-catalyzed asymmetric reductive cross-coupling between α-chlorosulfones and aryl halides. This transformation enables the synthesis of enantioenriched α,α-disubstituted sulfones from simple organic halides under mild conditions, including easy introduction of a wide set of heteroaromatic moieties into the α-sulfonyl positions. The present method is tolerant of a variety of functional groups, affording the coupling products generally in modest to good yields and high enantioselectivities. Finally, this work provides a convenient tool for late-stage functionalization of bioactive compounds by facile introduction of chiral α-sulfonyl subunits.

## Conflicts of interest

There are no conflicts to declare.

## Supplementary Material

SC-012-D1SC00283J-s001
