# Supplementary material for: Nickel-catalyzed asymmetric reductive arylation of α-chlorosulfones with aryl halides
Source: Chem Sci. 2021 Feb 19;12(14):5253–8. doi: 10.1039/d1sc00283j (PMC8179603; doi:10.1039/d1sc00283j)

Electronic Supplementary Material (ESI) for Chemical Science.
This journal is © The Royal Society of Chemistry 2021

Supporting Information

Nickel-Catalyzed Asymmetric Reductive Arylation of *α*-Chlorosulfones with Aryl Halides

Deli Sun, Guobin Ma, Xinluo Zhao, Chuanhu Lei* and Hegui Gong*

Center for Supramolecular Chemistry and Catalysis, College of Sciences, Shanghai University, 99 Shang-Da Road, Shanghai 200444, China

Hegui_gong@shu.edu.cn

Table of Contents

I. Experimental Section S2

Part 1. General Information S2

Part 2. Optimization experiments S2

Part 3. Preparation of *α*-chlorosulfones S4

Part 4. Cross-coupling reactions and product characterization S13

Part 5. Control experiments and Limitations S53

II. Reference S57

III. HPLC Traces S58

IV. NMR Data S125

I. Experimental Section

Part 1. General Information

Commercial reagents were purchased from Adamas, Aldrich, Bide, Energy Chemical, TCI, Leyan and J&K chemical. Column chromatography was performed using silica gel 200-300 mesh (purchased from Qingdao-Haiyang Co., China) as the solid support. All NMR spectra were recorded on Bruker Avance 600 MHz spectrometer at STP. Data for ^{1}H NMR spectra are reported as follows: chemical shift (δ ppm), multiplicity, coupling constant (Hz) and integration. ^{13}C NMR spectra were recorded at 151 MHz. Data for ^{13}C NMR spectra are reported in terms of chemical shift. NMR spectra are internally referenced to residual proton solvent signals (note: $CDCl_3$ referenced at 7.26 ppm for ^{1}H NMR and 77.0 ppm for ^{13}C NMR; Acetone-*d*6 at 2.05 for ^{1}H NMR, 29.84 ppm for ^{13}C NMR). Coupling constants were reported in Hz, and multiplicity was indicated as follows: s (singlet); d (doublet); t (triplet); q (quartet); quint (quintet); m (multiplet); dd (doublet of doublets); ddd (doublet of doublet of doublets); dt (doublet of triplets); td (triplet of doublets). High-resolution mass spectra (HRMS) were obtained using Bruker APEXIII 7.0 TESLA FTMS or Agilent 6545 Accurate-Mass Q-TOF LC/MS System. Optical rotations were taken on JASCO P1030. Enantiomeric excesses were determined by chiral HPLC using a Shimadzu instrument. Melting point was recorded on a micro melting point apparatus (X-4, YUHUA Co., Ltd, Gongyi, China).

Part 2. Optimization experiments

General procedure of optimization experiments: To a flame-dried tube with a stir bar vial was added the appropriate ligand (0.03 mmol, 20 mol %), reductant (0.45 mmol, 3.0 equiv), $NiBr_2$(dme) (0.015 mmol, 10 mol %), aryl bromide **2** (0.225 mmol, 1.5 equiv), and additive (0.15 mmol, 1.0 equiv). The vial was transferred into an N_2-filled glovebox and charged with the appropriate solvent (0.3 mL). The vial was sealed and removed from the glovebox. The mixture was stirred vigorously, ensuring that the reductant was uniformly suspended. After 5 min the mixture was cooled to 0 °C, then a solution of *α*-chlorosulfone **1** (0.15 mmol, 1.0 equiv) in DMF (0.3 mL) was added to the vial with a syringe over 1 min. The mixture was stirred vigorously at 0 °C for 48 h. The reaction mixture was loaded directly onto a silica gel to give the target molecule. The yield of target product was determined using 2,5-dimethylfuran as standard by ^{1}H NMR, and the enantioselectivity was determined by HPLC.

Table S1 Optimization of the reaction conditions [a,b]

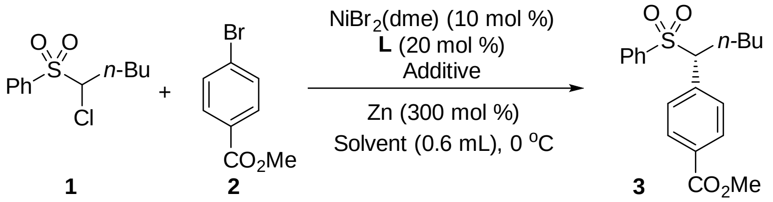

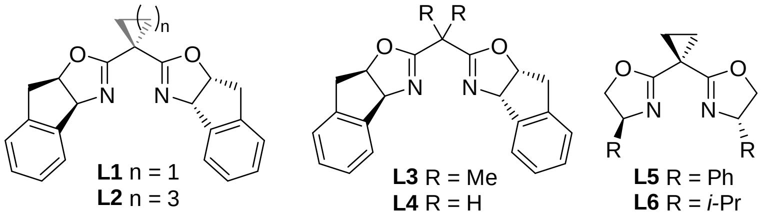

Entry	Ligand	Solvent	Ratio of 1/2	Additive (equiv.)	Yield [b] %	*e.e.*%
1	**L1**	DMA	1.5/1	$MgCl_2$ (2)	37	78
2	**L1**	THF	1.5/1	$MgCl_2$ (2)	37	88
3	**L1**	NMP	1.5/1	$MgCl_2$ (2)	20	86
4	**L1**	DMF	1.5/1	$MgCl_2$ (2)	42	84
5	**L1**	DMF	1/1	$MgCl_2$ (2)	50	87
6	**L1**	DMF	1/1.5	$MgCl_2$ (2)	67	89
7	**L1**	THF	1/1.5	$MgCl_2$ (2)	38	90
8	**L1**	DMF	1/2	$MgCl_2$ (2)	64	89
9	**L1**	DMF	1/1.5	$MgCl_2$ (1)	73	91
10	**L1**	DMF	1/1.5	$MgCl_2$ (0.5)	41	91
11	**L1**	DMF	1/1.5	$MgBr_2$ (1)	63	55
12 [c]	**L1**	DMF	1/1.5	TBAB (1)	trace	ND
13 [c]	**L1**	DMF	1/1.5	NaI (1)	trace	ND
14	**L2**	DMF	1/1.5	$MgCl_2$ (1)	71	90
15	**L3**	DMF	1/1.5	$MgCl_2$ (1)	28	88
16	**L4**	DMF	1/1.5	$MgCl_2$ (1)	34	40
17	**L5**	DMF	1/1.5	$MgCl_2$ (1)	65	88
18	**L6**	DMF	1/1.5	$MgCl_2$ (1)	31	77
19 [d]	**L1**	DMF	1/1.5	$MgCl_2$ (1)	74	88
20 [e]	**L1**	DMF	1/1.5	$MgCl_2$ (1)	76	91
21 [f]	**L1**	DMF	1/1.5	$MgCl_2$ (1)	73	91
22 [g]	(3*R*, 8*S*)-**L1**	DMF	1/1.5	$MgCl_2$ (1)	75	-92
23	**L1**	DMF	1/1.5	$MgCl_2$ (1)<br>TEMPO (1)	5	90

[a] Reactions conducted under N_2 on 0.15 mmol scale for 48 h. [b] NMR yield using 2,5-dimethyl furan as the internal standard. [c] ND = no detection. [d] Mn instead of Zn. [e] 10 mol % preformed **L1**·$NiBr_2$ complex used. [f] 10 mol % preformed **L1**·$NiCl_2$ complex used. [g] 10 mol % preformed (3*R*, 8*S*)-**L1**·$NiBr_2$ complex used.

Part 3. Preparation of α-Chlorosulfones

General procedure for preparation of α-chlorosulfones **(GP A)**.

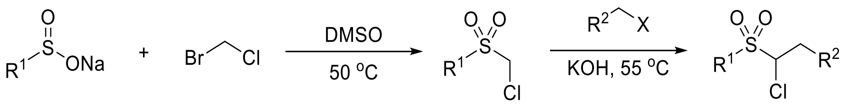

The α-chlorosulfones were prepared according to the literature reported.[1, 2] To a round-bottom flask with a stir bar was charged with the corrresponding substituted sulfinate (1.0 equiv) and DMSO (0.1 M) followed by drop-wise addition of CH_2ClBr (1.2 equiv). The reaction mixture was warmed to 50 °C and stirred for 16 hours, then quenched with sat. NH_4Cl (aq). The aqueous phase was extracted with EtOAc. The organic phase were collected, dried over $MgSO_4$, concentrated under reduced pressure. The crude material was purified by flash column chromatograph on silica gel to give chloromethylsuifone. To a round-bottom flask with a stirring bar was charged with the resulting chloromethylsuifone (1.0 equiv) and an alkyl bromide (1.5 equiv) followed by addition of KOH (1.5 equiv). The mixture was stirred at 55 °C for 48 hours. Then quenched with sat. NH_4Cl (aq). The aqueous phase was extracted with EtOAc, the organic phase were collected, dried over $MgSO_4$, concentrated under reduced pressure, the crude material was purified by flash column chromatograph on silica gel to give α-chlorosulfone.

General procedure for preparation of α-chlorosulfones **(GP B)**.

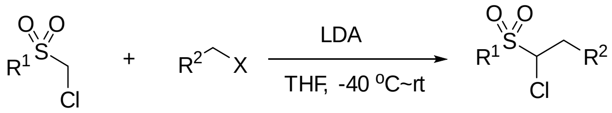

The α-chlorosulfones were prepared according to the literature reported.[3] To a round-bottom flask with a stir bar was charged with according chloromethylsuifone (5 mmol, 1.0 equiv) and THF (50 mL, 0.1 M), the mixture was cooled to -40 °C, followed by drop-wise addition of LDA (6 mmol, 1.2 equiv). The reaction mixture was stirred at this temperature for 30 minutes, then a solution of an alkyl bromide (6 mmol, 1.2 equiv) in THF (6 mL) was added over 10 min. The reaction mixture was stirred at this temperature for 2 hours, and then it was allowed to warm to room temperature and stirred for 2 hours. The resulting reaction mixture was stirred at r.t. overnight, and then it was quenched with sat. NH_4Cl (aq), the aqueous phase was extracted with EtOAc. The organic phase were collected, dried over $MgSO_4$, concentrated under reduced pressure. The crude material was purified by flash column

chromatograph on silica to afford target material.

((1-Chloropentyl)sulfonyl)benzene (SM1)

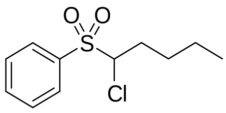

SM1 was prepared by ***GPA*** as a white solid (630 mg, 51% yield).

^{1}H NMR (600 MHz, $CDCl_3$) δ 8.01 – 7.93 (m, 2H), 7.74 – 7.69 (m, 1H), 7.60 (t, *J* = 7.8 Hz, 2H), 4.63 (dd, *J* = 10.9, 2.9 Hz, 1H), 2.44 – 2.40 (m, 1H), 1.81 – 1.85 (m, 1H), 1.69 – 1.60 (m, 1H), 1.48 – 1.29 (m, 3H), 0.92 (t, *J* = 7.3 Hz, 3H).

^{13}C NMR (151 MHz, $CDCl_3$) δ 135.4, 134.6, 130.2, 129.2, 75.1, 30.4, 28.1, 22.0, 13.9.

HRMS (ESI) calcd for: $C_{11}H_{19}ClNO_2S$ $[M+NH_4]^+$) m/z 264.0820, found 264.0817.

M.P. 39-41 °C.

1-((1-Chloropentyl)sulfonyl)-4-methylbenzene (SM2)

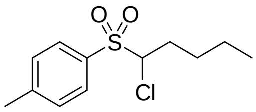

SM2 was prepared by ***GPA*** as a white solid (620 mg, 46% yield).

^{1}H NMR (600 MHz, $CDCl_3$) δ 7.89 – 7.79 (m, 2H), 7.42 – 7.35 (m, 2H), 4.61 (dd, *J* = 11.0, 2.8 Hz, 1H), 2.47 (s, 3H), 2.47 – 2.38 (m, 1H), 1.84 – 1.77 (m, 1H), 1.69 – 1.59 (m, 1H), 1.48 – 1.30 (m, 3H), 0.92 (t, *J* = 7.3 Hz, 3H).

^{13}C NMR (151 MHz, $CDCl_3$) δ 145.8, 132.3, 130.2, 129.9, 75.3, 30.5, 28.2, 22.0, 21.9, 13.9.

HRMS (ESI) calcd for: $C_{12}H_{21}ClNO_2S$ ($[M+NH_4]^+$) m/z 278.0976, found 278.0979.

M.P. 41.8 - 43.5 °C.

1-((1-Chloropentyl)sulfonyl)-4-methoxybenzene (SM3)

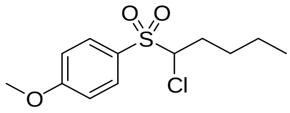

SM3 was prepared by ***GPA*** as a white solid (595 mg, 43 % yield).

^{1}H NMR (600 MHz, CDCl$_3$) δ 7.83 (dd, *J* = 5.0, 1.4 Hz, 1H), 7.80 (dd, *J* = 3.8, 1.4 Hz, 1H), 7.22 (dd, *J* = 5.0, 3.8 Hz, 1H), 4.70 (dd, *J* = 10.9, 2.9 Hz, 1H), 2.44 – 2.41 (m, 1H), 1.87 – 1.81 (m, 1H), 1.69 – 1.60 (m, 1H), 1.52 – 1.31 (m, 3H), 0.93 (t, *J* = 7.3 Hz, 3H).

^{13}C NMR (151 MHz, CDCl$_3$) δ 137.0, 135.9, 135.3, 128.1, 76.1, 30.9, 28.2, 22.0, 13.9.

HRMS (ESI) calcd for: $C_{12}H_{16}ClO_3S$ ($[M-H]^-$) m/z 275.0514, found 275.0512.

M.P. 52.1 - 53.8 °C.

2-((1-Chloropentyl)sulfonyl)thiophene (SM4)

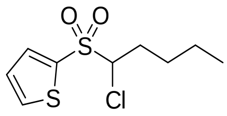

SM4 was prepared by ***GPA*** as a light-yellow oil (442 mg, 35 % yield).

^{1}H NMR (600 MHz, CDCl$_3$) δ 7.83 (dd, *J* = 5.0, 1.4 Hz, 1H), 7.80 (dd, *J* = 3.8, 1.4 Hz, 1H), 7.22 (dd, *J* = 5.0, 3.8 Hz, 1H), 4.70 (dd, *J* = 10.9, 2.9 Hz, 1H), 2.44 – 2.41 (m, 1H), 1.87 – 1.81 (m, 1H), 1.69 – 1.60 (m, 1H), 1.52 – 1.31 (m, 3H), 0.93 (t, *J* = 7.3 Hz, 3H).

^{13}C NMR (151 MHz, CDCl$_3$) δ 1 δ 137.0, 135.9, 135.3, 128.1, 76.1, 30.9, 28.2, 22.0, 13.9.

HRMS (ESI) calcd for: $C_9H_{17}ClNO_2S_2$ ($[M+NH_4]^+$) m/z 270.0384, found 270.0378.

1-((1-Chloropentyl)sulfonyl)-4-fluorobenzene (SM5)

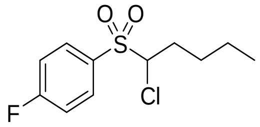

SM5 was prepared by ***GPA*** as a colorless oil (450 mg, 34 % yield).

^{1}H NMR (600 MHz, CDCl$_3$) δ 8.00 – 7.96 (m, 2H), 7.30 – 7.26 (m, 2H), 4.61 (dd, *J* = 10.9, 2.9 Hz, 1H), 2.47 – 2.39 (m, 1H), 1.88 – 1.77 (m, 1H), 1.70 – 1.60 (m, 1H), 1.48 – 1.32 (m, 3H), 0.93 (t, *J* = 7.3 Hz, 3H).

^{13}C NMR (151 MHz, CDCl$_3$) δ 166.5 (d, *J* = 257.7 Hz), 133.1 (d, *J* = 9.9 Hz), 131.3 (d, *J* = 3.2 Hz), 116.6 (d, *J* = 22.7 Hz), 75.2, 30.4, 28.1, 21.9, 13.8.

^{19}F NMR (565 MHz, CDCl$_3$) δ -101.96.

HRMS (ESI) calcd for: $C_{11}H_{14}ClFNaO_2S$ ($[M+Na]^+$) m/z 287.0279, found 287.0272.

2-((1-Chloropentyl)sulfonyl)-1,3,5-trimethylbenzene (SM6)

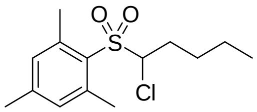

SM6 was prepared by ***GPA*** as a white solid (420 mg, 29 % yield).

^{1}H NMR (600 MHz, CDCl$_3$) δ 6.99 (s, 2H), 4.71 (dd, *J* = 10.6, 3.0 Hz, 1H), 2.68 (s, 6H), 2.42 – 2.37 (m, 1H), 2.32 (s, 3H), 2.10 – 2.03 (m, 1H), 1.70 – 1.67 (m, 1H), 1.52 – 1.32 (m, 3H), 0.94 (t, *J* = 7.3 Hz, 3H).

^{13}C NMR (151 MHz, CDCl$_3$) δ 144.2, 141.4, 132.5, 130.5, 75.0, 29.1, 28.2, 23.3, 22.1, 21.2, 13.9.

HRMS (ESI) calcd for: $C_{14}H_{21}ClNaO_2S$ ([M+Na]$^+$) m/z 311.0843, found 311.0841.

M.P. 119.8 – 121.5 °C.

((1-Chloroethyl)sulfonyl)benzene (SM7)

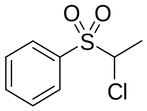

SM7 was prepared by ***GPA*** as a white solid (360 mg, 35 % yield).

^{1}H NMR (600 MHz, CDCl$_3$) δ 8.01 – 7.91 (m, 2H), 7.75 – 7.70 (m, 1H), 7.61 (t, *J* = 7.9 Hz, 2H), 4.80 (q, *J* = 6.8 Hz, 1H), 1.85 (d, *J* = 6.8 Hz, 3H).

^{13}C NMR (151 MHz, CDCl$_3$) δ 134.8, 134.73, 130.3, 129.3, 70.0, 18.3.

HRMS (ESI) calcd for: $C_8H_9ClNaO_2S$ ([M+Na]$^+$) m/z 226.9904, found 226.9905.

M.P. 50.8 - 52.3 °C.

(2-Chloro-2-(phenylsulfonyl)ethyl)benzene (SM8)

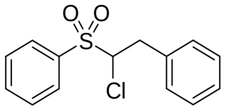

SM8 was prepared by ***GPA*** as a white solid (330 mg, 33 % yield).

^{1}H NMR (600 MHz, CDCl$_3$) δ 8.04 – 7.98 (m, 2H), 7.75 – 7.71 (m, 1H), 7.62 (t, *J* = 7.9 Hz, 2H), 7.33 (dd, *J* = 8.1, 6.5 Hz, 2H), 7.30 – 7.27 (m, 1H), 7.25 – 7.22 (m, 2H), 4.79 (dd, *J* = 11.4, 2.5 Hz, 1H), 3.88 (dd, *J* = 14.3, 2.5 Hz, 1H), 2.98 (dd, *J* = 14.3, 11.4 Hz, 1H).

^{13}C NMR (151 MHz, CDCl$_3$) δ 135.2, 134.8, 134.7, 130.2, 129.6, 129.3, 128.9, 127.8, 75.7, 37.0.

HRMS (ESI) calcd for: $C_{14}H_{13}ClNaO_2S$ ([M+Na]$^+$) m/z 303.0217, found 303.0211.

M.P. 45.3 – 46.9 °C.

tert-butyl(4-chloro-4-(phenylsulfonyl)butoxy)dimethylsilane (SM9)

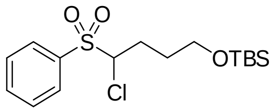

SM9 was prepared by ***GPB*** as a colorless oil (1.11 g, 61 % yield).

^{1}H NMR (600 MHz, $CDCl_3$) δ 8.00 – 7.91 (m, 2H), 7.74 – 7.68 (m, 1H), 7.62 – 7.56 (m, 2H), 4.79 (dd, J = 10.8, 2.9 Hz, 1H), 3.69 – 3.62 (m, 2H), 2.56 – 2.48 (m, 1H), 1.93 – 1.80 (m, 2H), 1.73-1.65 (m, 1H), 0.86 (s, 9H), 0.03 (s, 3H), 0.03 (s, 3H)..

^{13}C NMR (151 MHz, $CDCl_3$) δ 135.4, 134.6, 130.2, 129.2, 75.1, 62.1, 28.9, 28.2, 26.0, 18.4, -5.3.

HRMS (ESI) calcd for: $C_{16}H_{27}ClNaO_3SSi$ ($[M+Na]^+$) m/z 385.1031, found 385.1032.

((1,4-Dichlorobutyl)sulfonyl)benzene (SM10)

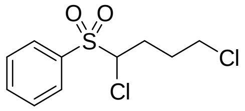

SM10 was prepared by ***GPA*** as a white solid (388 mg, 29 % yield).

^{1}H NMR (600 MHz, $CDCl_3$) δ 8.00 – 7.93 (m, 2H), 7.76 – 7.71 (m, 1H), 7.64 – 7.58 (m, 2H), 4.69 (dd, J = 10.1, 3.3 Hz, 1H), 3.59 (t, J = 6.2 Hz, 2H), 2.64 – 2.59 (m, 1H), 2.20 – 2.12 (m, 1H), 2.07 – 1.93 (m, 2H).

^{13}C NMR (151 MHz, $CDCl_3$) δ 135.1, 134.8, 130.2, 129.3, 74.1, 43.7, 28.9, 28.6.

HRMS (ESI) calcd for: $C_{10}H_{16}Cl_2NO_2S$ ($[M+NH_4]^+$) m/z 284.0273, found 284.0269.

M.P. 47.5 – 49.1 °C.

((1-Chloro-4-methylpentyl)sulfonyl)benzene (SM11)

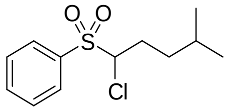

SM11 was prepared by ***GPA*** as a colorless oil (470 mg, 36 % yield).

^{1}H NMR (600 MHz, $CDCl_3$) δ 8.00 – 7.92 (m, 2H), 7.75 – 7.68 (m, 1H), 7.60 (t, J = 7.8 Hz, 2H), 4.60 (dd, J = 10.8, 2.8 Hz, 1H), 2.47 – 2.42 (m, 1H), 1.86 – 1.79 (m, 1H), 1.61 – 1.56 (m, 1H), 1.54 – 1.50 (m, 1H), 1.37 – 1.31 (m, 1H), 0.91 (dd, J = 12.4, 6.6 Hz, 6H).

^{13}C NMR (151 MHz, CDCl$_3$) δ 135.4, 134.6, 130.1, 129.2, 75.4, 35.0, 28.7, 27.7, 22.8, 22.2.

HRMS (ESI) calcd for: $C_{12}H_{21}ClNO_2S$ ($[M+NH_4]^+$) m/z 278.0976, found 278.0974.

((1-Chloropropyl)sulfonyl)benzene (SM12)

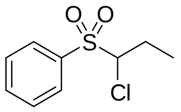

SM12 was prepared by ***GPA*** as a white solid (383 mg, 35 % yield).

^{1}H NMR (600 MHz, CDCl$_3$) δ 8.00 – 7.94 (m, 2H), 7.76 – 7.69 (m, 1H), 7.64 – 7.56 (m, 2H), 4.58 (dd, *J* = 10.6, 3.0 Hz, 1H), 2.51 – 2.45 (m, 1H), 1.90 – 1.82 (m, 1H), 1.16 (t, *J* = 7.3 Hz, 3H).

^{13}C NMR (151 MHz, CDCl$_3$) δ 135.4, 134.6, 130.2, 129.2, 76.6 24.7, 10.8.

HRMS (ESI) calcd for: $C_9H_{15}ClNO_2S$ ($[M+NH_4]^+$) m/z 236.0507, found 236.0503.

M.P. 58.7 – 60.3 °C.

((1-Chlorobutyl)sulfonyl)benzene (SM13)

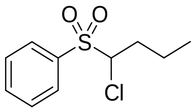

SM13 was prepared by ***GPA*** as a white solid (385 mg, 33 % yield).

^{1}H NMR (600 MHz, CDCl$_3$) δ 8.00 – 7.92 (m, 2H), 7.74 – 7.68 (m, 1H), 7.59 (t, *J* = 7.9 Hz, 2H), 4.65 (dd, *J* = 11.0, 2.9 Hz, 1H), 2.39 – 2.34 (m, 1H), 1.85 – 1.79 (m, 1H), 1.74 – 1.66 (m, 1H), 1.51 – 1.44 (m, 1H), 0.97 (t, *J* = 7.4 Hz, 3H).

^{13}C NMR (151 MHz, CDCl$_3$) δ 135.3, 134.6, 130.2, 129.2, 74.8, 32.6, 19.4, 13.3.

HRMS (ESI) calcd for: $C_{10}H_{17}ClNO_2S$ ($[M+NH_4]^+$) m/z 250.0663, found 250.0660.

M.P. 41.3 – 42.8 °C.

1-(*tert*-Butyl)-4-((1-chloropentyl)sulfonyl)benzene (SM14)

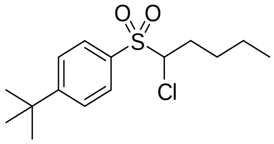

SM14 was prepared by ***GPA*** as a colorless oil (425 mg, 28 % yield).

1H NMR (600 MHz, CDCl3) δ 7.89 – 7.84 (m, 2H), 7.60 – 7.56 (m, 2H), 4.62 (dd, *J* = 10.9, 2.9 Hz, 1H), 2.44 – 2.38 (m, 1H), 1.86 – 1.79 (m, 1H), 1.67 – 1.60 (m, 1H), 1.48 – 1.38 (m, 2H), 1.36 (s, 10H), 0.92 (t, *J* = 7.3 Hz, 3H).

13C NMR (151 MHz, CDCl3) δ 158.7, 132.3, 130.0, 126.2, 75.2, 35.5, 31.2, 30.5, 28.2, 22.0, 13.9.

HRMS (ESI) calcd for: $C_{15}H_{23}ClNaO_2S$ ([M+Na]$^+$) m/z 325.1000 , found 325.0999.

(2-Chloro-2-(methylsulfonyl)ethyl)benzene (SM15)

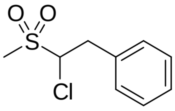

SM15 was prepared by ***GPA*** as a white solid (120 mg, 11 % yield).

1H NMR (600 MHz, CDCl3) δ 7.39 – 7.35 (m, 2H), 7.34 – 7.31 (m, 1H), 7.29 (dd, *J* = 6.9, 1.7 Hz, 2H), 4.75 (dd, *J* = 11.0, 2.7 Hz, 1H), 3.81 (dd, *J* = 14.4, 2.7 Hz, 1H), 3.12 – 3.04 (m, 4H).

13C NMR (151 MHz, CDCl3) δ 134.4, 129.7, 129.0, 127.9, 74.5, 37.6, 35.8.

HRMS (ESI) calcd for: $C_9H_{15}ClNO_2S$ ([M+NH$_4$]$^+$) m/z 236.0507, found 236.0501.

M.P. 71.5 – 73.2 ℃.

4-((1-Chloropentyl)sulfonyl)morpholine (SM16)

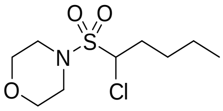

SM16 was prepared by ***GPB*** as a white solid (350 mg, 68% yield).

1H NMR (600 MHz, CDCl3) δ 4.68 (dd, *J* = 10.6, 3.1 Hz, 1H), 3.79 – 3.69 (m, 4H), 3.54 – 3.42 (m, 4H), 2.32 – 2.28 (m, 1H), 1.99 – 1.94 (m, 1H), 1.69 – 1.61 (m, 1H), 1.49 – 1.31 (m, 3H), 0.94 (t, *J* = 7.2 Hz, 3H).

13C NMR (151 MHz, CDCl3) δ 73.2, 67.1, 47.2, 32.3, 28.1, 22.0, 13.9.

HRMS (ESI) calcd for: $C_9H_{18}ClNNaO_3S$ ([M+Na]$^+$) m/z 278.0588, found 278.0582.

M.P. 54.1 – 55.9 ℃.

1-Chloro-4-((1-chloropentyl)sulfonyl)benzene (SM17)

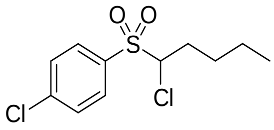

SM17 was prepared by ***GPA*** as a white solid (338 mg, 24% yield).

^{1}H NMR (600 MHz, $CDCl_3$) δ 7.94 – 7.85 (m, 2H), 7.61 – 7.53 (m, 2H), 4.62 (dd, *J* = 10.9, 2.9 Hz, 1H), 2.45 – 2.39 (m, 1H), 1.85 – 1.78 (m, 1H), 1.68 – 1.60 (m, 1H), 1.48 – 1.32 (m, 3H), 0.93 (t, *J* = 7.3 Hz, 3H).

^{13}C NMR (151 MHz, $CDCl_3$) δ 141.6, 133.8, 131.6, 129.6, 75.1, 30.4, 28.1, 22.0, 13.9.

HRMS (ESI) calcd for: $C_{11}H_{18}Cl_2NO_2S$ ($[M+NH_4]^+$) m/z 298.0430, found 298.0422.

M.P. 33.9 – 35.8 °C.

((1-Chlorobut-3-en-1-yl)sulfonyl)benzene (SM18)

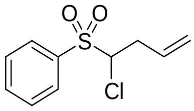

SM18 was prepared by ***GPA*** as a colorless oil (369 mg, 32% yield).

^{1}H NMR (600 MHz, $CDCl_3$) δ 8.02 – 7.94 (m, 2H), 7.74 – 7.71 (m, 1H), 7.64 – 7.58 (m, 2H), 5.84 – 5.77 (m, 1H), 5.29 – 5.21 (m, 2H), 4.66 (dd, *J* = 10.5, 3.1 Hz, 1H), 3.21 – 3.16 (m, 1H), 2.62 – 2.56 (m, 1H).

^{13}C NMR (151 MHz, $CDCl_3$) δ 135.2, 134.8, 130.9, 130.2, 129.2, 120.4, 73.9, 35.3.

HRMS (ESI) calcd for: $C_{10}H_{15}ClNO_2S$ ($[M+NH_4]^+$) m/z 248.0507, found 248.0499.

***tert*-Butyl(3-chloro-3-(phenylsulfonyl)propoxy)dimethylsilane (SM19)**

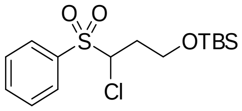

SM19 was prepared by ***GPA*** as a colorless oil (1.15 g, 66% yield).

^{1}H NMR (600 MHz, $CDCl_3$) δ 7.96 (dd, *J* = 8.2, 1.4 Hz, 2H), 7.75 – 7.68 (m, 1H), 7.60 (t, *J* = 7.8 Hz, 2H), 4.94 (dd, *J* = 11.1, 2.6 Hz, 1H), 3.87 – 3.84 (m, 1H), 3.76 (td, *J* = 10.4, 3.4 Hz, 1H), 2.68 – 2.62 (m, 1H), 1.88 – 1.83 (m, 1H), 0.87 (s, 9H), 0.04 (s, 3H), 0.04 (s, 3H)..

^{13}C NMR (151 MHz, $CDCl_3$) δ 135.4, 134.6, 130.1, 129.2, 72.0, 58.2, 33.9, 26.0, 18.3, -5.3, -5.4.

HRMS (ESI) calcd for: $C_{15}H_{25}ClNaO_3SSi$ ($[M+Na]^+$) m/z 371.0874, found 371.0874.

3-Chloro-3-(phenylsulfonyl)propan-1-ol (SM20)

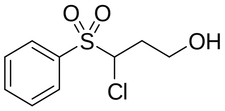

To a stirred solution of SM19 (698 mg, 2.0 mmol) in THF was added TBAF (1.0 M in THF) at rt, and then the mixture was stirred at rt overnight. Then the reaction was quenched with sat. NH_4Cl (aq), the aqueous phase was extracted with EtOAc. The organic phase were collected, dried over $MgSO_4$, concentrated under reduced pressure. The crude material was purified by flash column chromatograph on silica to give **SM20** as a colorless oil (380 mg, 81% yield).

^{1}H NMR (600 MHz, $CDCl_3$) δ 7.96 (d, *J* = 8.0 Hz, 2H), 7.74 – 7.68 (m, 1H), 7.63 – 7.57 (m, 2H), 5.00 (dt, *J* = 10.5, 2.1 Hz, 1H), 3.96 (dt, *J* = 10.1, 4.6 Hz, 1H), 3.81 (s, 1H), 2.71 – 2.65 (m, 1H), 2.03 – 1.98 (m, 2H).

^{13}C NMR (151 MHz, $CDCl_3$) δ 135.2, 134.8, 130.1, 129.3, 71.8, 58.0, 33.6.

HRMS (ESI) calcd for: $C_9H_{11}ClNaO_3S$ ($[M+Na]^+$) m/z 257.0010, found 257.0007.

1-(4-((1-Chloropentyl)sulfonyl)phenyl)-5-(p-tolyl)-3-(trifluoromethyl)-1*H*-pyrazole (SM21)

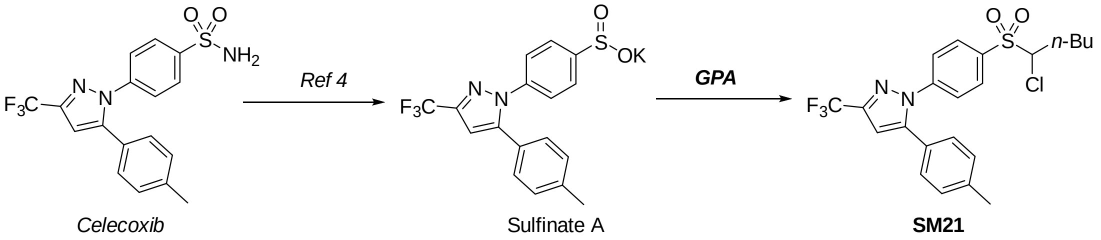

Sulfinate A was prepared from Celecoxib according to reported literature.[4] **SM21** was prepared by ***GPA*** as a white solid (470 mg, 20% yield).

^{1}H NMR (600 MHz, $CDCl_3$) δ 7.97 – 7.89 (m, 2H), 7.58 – 7.52 (m, 2H), 7.18 (d, *J* = 7.9 Hz, 1H)，7.13 – 7.08 (m, 2H), 4.65 – 4.58 (m, 1H), 2.39 (s, 4H), 1.87 – 1.76 (m, 1H), 1.68 – 1.58 (m, 1H), 1.48 – 1.31 (m, 3H), 0.93 (t, *J* = 7.2 Hz, 3H).

^{13}C NMR (151 MHz, $CDCl_3$) δ 145.6, 144.5 (q, *J* = 38.7 Hz), 144.2, 140.1, 134.5, 131.3, 130.0, 128.9, 125.7, 125.4, 121.1(q, *J* = 269.5 Hz), 106.7 (q, *J* =, 1.51), 75.1, 30.4, 28.1, 22.0, 21.5, 13.8.

^{19}F NMR (565 MHz, $CDCl_3$) δ -62.53.

HRMS (ESI) calcd for: $C_{22}H_{22}ClF_3N_2NaO_2S$ ($[M+Na]^+$) m/z 493.0935, found 493.0935.

M.P. 75.8 – 76.9 °C.

((Chloro(cyclopropyl)methyl)sulfonyl)benzene (69)

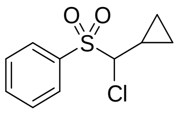 **69** was prepared according to the reported literature (207 mg, 9% yield).[5]

^{1}H NMR (600 MHz, CDCl$_3$) δ 7.97 (dd, *J* = 8.2, 1.4 Hz, 2H), 7.73 – 7.67 (m, 1H), 7.58 (t, *J* = 7.9 Hz, 2H), 4.24 (d, *J* = 8.7 Hz, 1H), 1.39 – 1.32 (m, 1H), 0.86 – 0.80 (m, 1H), 0.78 – 0.71 (m, 1H), 0.67 – 0.61 (m, 1H), 0.46-0.42 (m, 1H).

^{13}C NMR (151 MHz, CDCl$_3$) δ 135.7, 134.6, 130.2, 129.1, 79.3, 12.8, 7.0, 4.3.

HRMS (ESI) calcd for: $C_{10}H_{11}ClNaO_2S$ ([M+Na]$^+$) m/z 253.0061, found 253.0063.

Part 4. Cross-coupling reactions and product characterization

General procedure for reductive enantioselective reductive cross-coupling reactions: To a flame-dried tube with a stir bar vial was added the appropriate reductant (0.45 mmol, 3.0 equiv), aryl halide (0.225 mmol, 1.5 equiv), $MgCl_2$ (0.15 mmol, 1.0 equiv), and **L1**·$NiBr_2$ (0.015mmol, 10 mol %).[6] The vial was transferred into an N_2-filled glovebox and charged with the appropriate solvent (0.3 mL). The vial was sealed and removed from the glovebox. The mixture was stirred vigorously, ensuring that the reductant was uniformly suspended. After 5 min the mixture was cooled to 0 °C, then a solution of *α*-chlorosulfone (0.15 mmol, 1.0 equiv) in solvent (0.3 mL) was added to the vial by syringe over 1 min. The mixture was stirred vigorously at 0 °C for 48 h. The reaction mixture was loaded directly onto a silica gel to give the target molecular.

The absolute stereochemistry was assigned by comparing the optical rotation of compound **21** with the literature reported measurement.[3] A *R*-configuration was determined for **21** accordingly, and was applied to all the products reported in this work.

Methyl (*R*)-4-(1-(phenylsulfonyl)pentyl)benzoate (3)

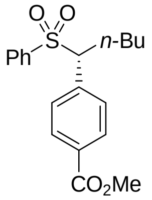

This compound was prepared according to the general procedure with Zn using ((1-chloropentyl)sulfonyl)benzene (37 mg, 0.15 mmol, 1.0 equiv) and methyl 4-bromobenzoate (48.4 mg, 0.225 mmol, 1.5 equiv). The crude mixture was purified by flash column chromatograph to afford **3** as an off-white solid (37.9 mg, 73% yield, 91% ee).

Chiral HPLC (CHIRALCEL OD-H, *i*PrOH-hexanes 10/90, 1.0 mL/min, 240 nm, t_R (minor) = 9.7 min,

t_R (major) = 12.5 min.

^{1}H NMR (600 MHz, $CDCl_3$) δ 7.92 – 7.86 (m, 2H), 7.56 – 7.49 (m, 3H), 7.41 – 7.34 (m, 2H), 7.19 – 7.14 (m, 2H), 4.08 (dd, J = 11.6, 3.6 Hz, 1H), 3.90 (s, 3H), 2.47 – 2.41 m, 1H), 2.18 – 2.12 (m, 1H), 1.36 – 1.22 (m, 2H), 1.20 – 1.08 (m, 2H), 0.81 (t, J = 7.3 Hz, 3H).

^{13}C NMR (151 MHz, $CDCl_3$) δ 166.68, 137.81, 137.31, 133.77, 130.52, 129.98, 129.73, 129.08, 128.87, 71.54, 52.37, 28.97, 27.17, 22.35, 13.81.

HRMS (ESI) calcd. for $C_{19}H_{21}O_4S$ ($[M-H]^-$) m/z 345.1166, found 345.1160.

M.P. 75.2 – 76.9 ^{o}C.

$[\alpha]_D$ (25.0 °C, c = 1.00 in $CHCl_3$) = +87°.

(*R*)-1-(4-(1-(Phenylsulfonyl)pentyl)phenyl)ethan-1-one (4)

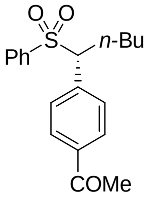

This compound was prepared according to the general procedure with Zn using ((1-chloropentyl)sulfonyl)benzene (37 mg, 0.15 mmol, 1.0 equiv) and 1-(4-bromophenyl)ethan-1-one (44.8 mg, 0.225 mmol, 1.5 equiv). The crude mixture was purified by flash column chromatograph to afford **4** as an off-white solid (27.3 mg, 55% yield, 91% ee).

Chiral HPLC (CHIRALCEL OD-H, *i*PrOH-hexanes 10/90, 1.0 mL/min, 240 nm, t_R (minor) = 12.9 min, t_R (major) = 16.8 min.

^{1}H NMR (600 MHz, $CDCl_3$) δ 7.85 – 7.79 (m, 2H), 7.57 – 7.49 (m, 3H), 7.41 – 7.35 (m, 2H), 7.23 – 7.17 (m, 2H), 4.09 (dd, J = 11.7, 3.6 Hz, 1H), 2.57 (s, 3H), 2.44 – 2.38 (m, 1H), 2.18 – 2.11 (m, 1H), 1.34 – 1.21 (m, 3H), 0.81 (t, J = 7.4 Hz, 3H).

^{13}C NMR (151 MHz, $CDCl_3$) δ 197.6, 137.9, 137.3, 137.3, 133.8, 130.2, 129.1, 128.9, 128.5, 71.5, 28.9, 27.2, 26.8, 22.3, 13.8.

HRMS (ESI) calcd for $C_{19}H_{23}O_3S$ ($[M+H]^+$) *m/z* 331.1362, found 331.1372.

M.P. 94.8 – 96.3 ^{o}C.

$[\alpha]_D$ (25.0 °C, c = 0.45 in $CHCl_3$) = +81.333°.

(*R*)-4-(1-(Phenylsulfonyl)pentyl)benzonitrile (5)

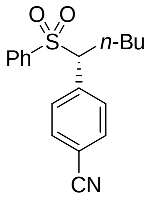

This compound was prepared according to the general procedure with Zn using ((1-chloropentyl)sulfonyl)benzene (37 mg, 0.15 mmol, 1.0 equiv) and 4-bromobenzonitrile (41 mg, 0.225 mmol, 1.5 equiv). The crude mixture was purified by flash column chromatograph to afford **5** as an off-white solid (19.8 mg, 42% yield, 89% ee)

Chiral HPLC (CHIRALCEL OD-H**,** *i*PrOH-hexanes 10/90, 1.0 mL/min, 240 nm, t_R (minor) = 12.8 min, t_R (major) = 16.6 min.

^{1}H NMR (600 MHz, CDCl$_3$) δ 7.62 – 7.56 (m, 1H), 7.56 – 7.49 (m, 4H), 7.45 – 7.39 (m, 2H), 7.25 – 7.20 (m, 2H), 4.07 (dd, *J* = 11.6, 3.7 Hz, 1H), 2.46 – 2.37 (m, 1H), 2.16 – 2.10 (m, 1H), 1.34 – 1.26 (m, 2H), 1.20 – 1.07 (m, 2H), 0.82 (t, *J* = 7.4 Hz, 3H).

^{13}C NMR (151 MHz, CDCl$_3$) δ 138.2, 137.1, 134.0, 132.3, 130.7, 129.1, 129.0, 118.4, 112.8, 71.4, 28.9, 27.2, 22.3, 13.8.

HRMS (ESI) calcd. for $C_{18}H_{19}NNaO_2S$ ([M+Na]$^+$) *m/z* 336.1028, found 336.1023.

M.P. 87.3 – 89 ^{o}C.

$[\alpha]_D$ (25.0 °C, c = 0.50 in $CHCl_3$) = +91.6°.

(*R*)-1-(Methylsulfonyl)-4-(1-(phenylsulfonyl)pentyl)benzene (6)

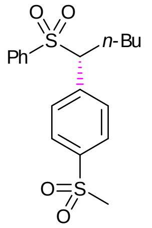

This compound was prepared according to the general procedure with Zn using ((1-chloropentyl)sulfonyl)benzene (0.15 mmol, 1.0 equiv) and 1-bromo-4-(methylsulfonyl)benzene (52.9 mg, 0.225 mmol, 1.5 equiv). The crude mixture was purified by flash column chromatograph to afford **6** as an off-white solid (34.6 mg, 63% yield, 88% ee).

Chiral HPLC (CHIRALCEL OD-H**,** *i*PrOH-hexanes 20/80, 1.0 mL/min, 210 nm, t_R (minor) = 19.8 min, t_R (major) = 26.4 min.

^{1}H NMR (600 MHz, CDCl$_3$) δ 7.86 – 7.80 (m, 2H), 7.62 – 7.55 (m, 3H), 7.45 – 7.40 (m, 2H), 7.38 – 7.33 (m, 2H), 4.12 (dd, *J* = 11.6, 3.7 Hz, 1H), 3.05 (s, 3H), 2.39 – 2.33 (m, 1H), 2.7 – 2.11 (m, 1H), 1.33 – 1.21 (m, 2H), 1.17 – 1.07 (m, 2H), 0.81 (t, *J* = 7.4 Hz, 3H).

^{13}C NMR (151 MHz, CDCl$_3$) δ 140.9, 139.0, 137.2, 134.1, 131.0, 129.1, 129.0, 127.6, 71.2, 44.6, 28.9, 27.5, 22.3, 13.8.

HRMS (ESI) calcd. for $C_{18}H_{21}O_4S_2$ ([M-H]$^-$) *m/z* 365.0887, found 365.0894.

M.P. 54.2 – 56 ºC.

[$\boldsymbol{\alpha}$]$_D$ (25.0 °C, c = 0.5 in $CHCl_3$) = +79.4°.

(*R*)-Phenyl(4-(1-(phenylsulfonyl)pentyl)phenyl)methanone (7)

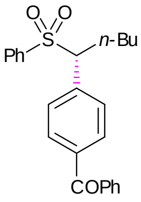

This compound was prepared according to the general procedure with Zn using ((1-chloropentyl)sulfonyl)benzene (37 mg, 0.15 mmol, 1.0 equiv) and (4-bromophenyl)(phenyl)methanone (58.8 mg, 0.225 mmol, 1.5 equiv). The crude mixture was purified by flash column chromatograph to afford **7** as an off-white solid (31.2 mg, 53% yield, 89% ee).

Chiral HPLC (CHIRALCEL OD-H**,** *i*PrOH-hexanes 10/90, 1.0 mL/min, 254 nm, t_R (minor) = 12.8 min, t_R (major) = 16.3 min.

^{1}H NMR (600 MHz, $CDCl_3$) δ δ 7.79 – 7.74 (m, 2H), 7.71 – 7.65 (m, 2H), 7.63 – 7.55 (m, 4H), 7.49 (dd, *J* = 8.4, 7.1 Hz, 2H), 7.42 (dd, *J* = 8.4, 7.4 Hz, 2H), 7.23 (d, *J* = 1.7 Hz, 2H), 4.12 (dd, *J* = 11.6, 3.6 Hz, 1H), 2.46 – 2.41 (m, 1H), 2.22 - 2.15 (m, 1H), 1.38 – 1.27 (m, 2H), 1.20 (q, *J* = 8.2 Hz, 2H), 0.84 (t, *J* = 7.3 Hz, 3H).

^{13}C NMR (151 MHz, $CDCl_3$) δ 196.2, 137.8, 137.4, 137.4, 137.2, 133.8, 132.8, 130.2, 130.1, 129.9, 129.1, 128.9, 128.5, 71.6, 29.0, 27.3, 22.4, 13.8.

HRMS (ESI) calcd for $C_{24}H_{25}O_3S$ ($[M+H]^+$) *m/z* 393.1519, found 393.1521.

[$\boldsymbol{\alpha}$]$_D$ (25.0 °C, c = 0.8 in $CHCl_3$) = +72.75°.

***(R)*-1-(1-(Phenylsulfonyl)pentyl)-4-(trifluoromethyl)benzene (8)**

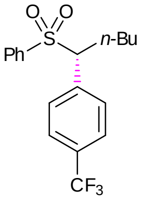

This compound was prepared according to the general procedure with Zn using ((1-chloropentyl)sulfonyl)benzene (37 mg, 0.15 mmol, 1.0 equiv) and 1-bromo-4-(trifluoromethyl)benzene (50.6 mg, 0.225 mmol, 1.5 equiv). The crude mixture was purified by flash column chromatograph to afford **8** as an off-white solid (33.2 mg, 62% yield, 91% ee).

Chiral HPLC (CHIRALCEL OD-H**,** *i*PrOH-hexanes 10/90, 1.0 mL/min, 220 nm, t_R (minor) = 6.0 min, t_R (major) = 7.3 min.

^{1}H NMR (600 MHz, $CDCl_3$) δ 7.59 – 7.52 (m, 3H), 7.50 (d, *J* = 8.1 Hz, 2H), 7.44 – 7.37 (m, 2H), 7.23 (d, *J* = 8.0 Hz, 2H), 4.08 (dd, *J* = 11.6, 3.6 Hz, 1H), 2.44 – 2.39 (m, 1H), 2.18 – 2.11 (m, 1H), 1.37 –

1.22 (m, 2H), 1.21 – 1.09 (m, 2H), 0.82 (t, *J* = 7.4 Hz, 3H).

^{13}C NMR (151 MHz, $CDCl_3$) δ δ 137.2, 136.8, 133.9, 131.0 (q, *J* = 32.7 Hz), 130.4, 129.1, 128.9, 125.5(q, *J* = 3.7 Hz), 123.95 (d, *J* = 272.2 Hz), 71.3, 28.9, 27.3, 22.3, 13.8.

^{19}F NMR (565 MHz, $CDCl_3$) δ -62.75.

HRMS (ESI) calcd. for $C_{18}H_{23}F_3NO_2S$ ($[M+NH_4]^+$) *m/z* 374.1396, found: 374.1398.

M.P. 113.1 – 114.9 °C.

$[\alpha]_D$ (25.0 °C, c = 0.72 in $CHCl_3$) = +77.778°.

***(R)*-4-(1-(Phenylsulfonyl)pentyl)benzaldehyde (9)**

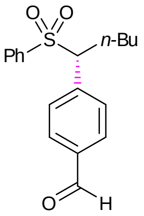

This compound was prepared according to the general procedure with Zn using ((1-chloropentyl)sulfonyl)benzene (37 mg, 0.15 mmol, 1.0 equiv) and 4-bromobenzaldehyde (41.6 mg, 0.225 mmol, 1.5 equiv). The crude mixture was purified by flash column chromatograph to afford **9** as an off-white solid (19 mg, 40% yield, 92% ee).

Chiral HPLC (CHIRALCEL OD-H**,** *i*PrOH-hexanes 20/80, 1.0 mL/min, 254 nm, t_R (minor) = 8.5 min, t_R (major) = 10.7 min.

^{1}H NMR (600 MHz, $CDCl_3$) δ 9.99 (s, 1H), 7.76 (d, *J* = 7.9 Hz, 2H), 7.60 – 7.51 (m, 3H), 7.40 (t, *J* = 7.7 Hz, 2H), 7.29 (d, *J* = 7.9 Hz, 2H), 4.12 (dd, *J* = 11.7, 3.6 Hz, 1H), 2.47 – 2.42 (m, 1H), 2.21 – 2.14 (m, 1H), 1.36 – 1.26 (m, 2H), 1.22 – 1.09 (m, 2H), 0.82 (t, *J* = 7.3 Hz, 3H).

^{13}C NMR (151 MHz, $CDCl_3$) δ 191.77, 139.45, 137.24, 136.47, 133.89, 130.64, 129.76, 129.05, 128.95, 71.61, 28.96, 27.25, 22.34, 13.80.

HRMS (ESI) calcd. for $C_{18}H_{20}NaO_3S$ ($[M+Na]^+$) *m/z* 339.1025, found 339.1025.

M.P. 74.3 – 76.1 °C.

$[\alpha]_D$ (25.0 °C, c = 0.50 in $CHCl_3$) = +73.8°.

(*R*)-*N*,*N*-Dimethyl-4-(1-(phenylsulfonyl)pentyl)benzamide (10)

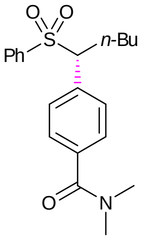

This compound was prepared according to the general procedure with Zn using ((1-chloropentyl)sulfonyl)benzene (37 mg, 0.15 mmol, 1.0 equiv) and 4-bromo-N,N-dimethylbenzamide (51.3 mg, 0.225 mmol, 1.5 equiv). The crude mixture was purified

by flash column chromatograph to afford **10** as a light-yellow oil (22.1 mg, 41% yield, 89% ee).

Chiral HPLC (CHIRALCEL OD-H**,** *i*PrOH-hexanes 20/80, 1.0 mL/min, 240 nm, t_R (minor) = 11.0 min, t_R (major) = 13.9 min.

^{1}H NMR (600 MHz, $CDCl_3$) δ 7.65 – 7.47 (m, 3H), 7.44 – 7.35 (m, 2H), 7.33 – 7.26 (m, 2H), 7.14 (d, *J* = 8.1 Hz, 2H), 4.04 (dd, *J* = 11.7, 3.6 Hz, 1H), 3.01 (d, *J* = 97.7 Hz, 6H), 2.44 – 2.32 (m, 1H), 2.20 – 2.08 (m, 1H), 1.34 – 1.21 (m, 2H), 1.18 – 1.12 (m, 2H), 0.81 (t, *J* = 7.3 Hz, 3H).

^{13}C NMR (151 MHz, $CDCl_3$) δ 171.0, 137.5, 136.8, 134.1, 133.7, 130.0, 129.1, 128.8, 127.3, 71.5, 28.9, 27.2, 22.3, 13.8.

HRMS (ESI) calcd. for $C_{20}H_{26}NO_3S$ ($[M+H]^+$) *m/z* 360.1628, found 360.1631.

$[\alpha]_D$ (25.0 °C, c = 0.9 in $CHCl_3$) = +59.222°.

(*R*)-*N*-Methoxy-*N*-methyl-4-(1-(phenylsulfonyl)pentyl)benzamide (11)

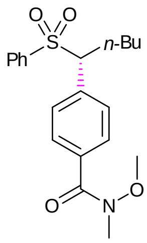

This compound was prepared according to the general procedure with Zn using ((1-chloropentyl)sulfonyl)benzene (37 mg, 0.15 mmol, 1.0 equiv) and 4-bromo-N-methoxy-N-methylbenzamide (54.9 mg, 0.225 mmol, 1.5 equiv). The crude mixture was purified by flash column chromatograph to afford **11** as an off-white solid (40 mg, 71% yield, 90% ee).

Chiral HPLC (CHIRALCEL OD-H**,** *i*PrOH-hexanes 20/80, 1.0 mL/min, 254 nm, t_R (minor) = 9.3 min, t_R (major) = 11.7 min.

^{1}H NMR (600 MHz, $CDCl_3$) δ 7.54-7.50 (m, 5H), 7.38 - 7.32 (m, 2H), 7.15 – 7.10 (m, 2H), 4.05 (dd, *J* = 11.6, 3.6 Hz, 1H), 3.51 (s, 3H), 3.33 (s, 3H), 2.44 – 2.38 (m, 1H), 2.19 – 2.09 (m, 1H), 1.37 – 1.21 (m, 2H), 1.20 – 1.09 (m, 2H), 0.80 (t, *J* = 7.3 Hz, 3H).

^{13}C NMR (151 MHz, $CDCl_3$) δ 166.2, 137.4, 135.2, 134.4, 133.6, 129.6, 129.1, 128.8, 128.4, 71.4, 61.1, 28.9, 27.1, 22.3, 13.8.

HRMS (ESI) calcd. for $C_{20}H_{26}NO_4S$ ($[M+H]^+$) *m/z* 376.1577, found: 376.1583.

M.P. 48.9 – 51.3 °C .

$[\alpha]_D$ (25.0 °C, c = 0.36 in $CHCl_3$) = +105.833°.

Diethyl (*R*)-(4-(1-(phenylsulfonyl)pentyl)phenyl)phosphonate (12)

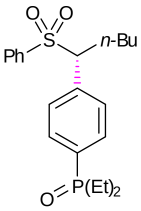

This compound was prepared according to the general procedure with Zn using ((1-chloropentyl)sulfonyl)benzene (37 mg, 0.15 mmol, 1.0 equiv) and diethyl (4-bromophenyl)phosphonate (66 mg, 0.225 mmol, 1.5 equiv). The crude mixture was purified by flash column chromatograph to afford **12** as a yellow oil (33.1 mg, 52% yield, 91% ee).

Chiral HPLC (CHIRALCEL OD-H**,** *i*PrOH-hexanes 20/80, 1.0 mL/min, 240 nm, t_R (minor) = 6.5 min, t_R (major) = 7.9 min.

^{1}H NMR (600 MHz, $CDCl_3$) δ 7.66 (dd, *J* = 13.0, 8.0 Hz, 2H), 7.57 – 7.49 (m, 3H), 7.36 (t, *J* = 7.8 Hz, 2H), 7.20 (dd, *J* = 8.1, 3.7 Hz, 2H), 4.18 – 4.00 (m, 5H), 2.43 – 2.37 (m, 1H), 2.19 – 2.10 (m, 1H), 1.31 (td, *J* = 7.1, 2.0 Hz, 6H), 1.29 – 1.21 (m, 2H), 1.18 – 1.09 (m, 2H), 0.81 (t, *J* = 7.3 Hz, 3H).

^{13}C NMR (151 MHz, $CDCl_3$) δ 137.3, 137.21 (d, *J* = 3.3 Hz), 133.78 , 131.90 (d, *J* = 10.4 Hz), 130.0 (d, *J* = 15.2 Hz), 129.1 (d, *J* = 188.75 Hz), 129.1, 128.8, 71.54 , 62.38 , 62.35 , 28.94 , 27.20 , 22.33 , 16.48 , 16.44 , 13.78 .

HRMS (ESI) calcd. for $C_{21}H_{30}O_5PS$ ($[M+H]^+$) *m/z* 425.1546, found: 425.1554.

$[\alpha]_D$ (25.0 °C, c = 1.40 in $CHCl_3$) = +54.429°.

(*R*)-4-((4-(1-(Phenylsulfonyl)pentyl)phenyl)sulfonyl)morpholine (13)

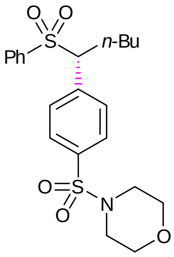

This compound was prepared according to the general procedure with Zn using ((1-chloropentyl)sulfonyl)benzene (37 mg, 0.15 mmol, 1.0 equiv) and 4-((4-bromophenyl)sulfonyl)morpholine (68.9 mg, 0.225 mmol, 1.5 equiv). The crude mixture was purified by flash column chromatograph to afford **13** as an off-white solid (46 mg, 70% yield, 89% ee).

Chiral HPLC (CHIRALCEL OD-H**,** *i*PrOH-hexanes 10/90, 1.0 mL/min, 254 nm, t_R (minor) = 19.6 min, t_R (major) = 31.5 min.

^{1}H NMR (600 MHz, $CDCl_3$) δ 7.59 (d, *J* = 7.9 Hz, 2H), 7.56 – 7.53 (m, 1H), 7.52 – 7.47 (m, 2H), 7.43 – 7.37 (m, 2H), 7.30 (d, *J* = 8.0 Hz, 2H), 4.11 (dd, *J* = 11.5, 3.7 Hz, 1H), 3.74 (t, *J* = 4.8 Hz, 4H), 2.95 – 2.89 (m, 4H), 2.47 – 2.42 (m, 1H), 2.19 – 2.13 (m, 1H), 1.34 – 1.24 (m, 2H), 1.21 – 1.09 (m, 2H), 0.83 (t, *J* = 7.3 Hz, 3H).

^{13}C NMR (151 MHz, $CDCl_3$) δ 138.4, 137.2, 135.2, 133.9, 130.7, 129.0, 128.9, 128.0, 71.3, 66.1, 46.1, 29.0, 27.2, 22.3, 13.8.

HRMS (ESI) calcd. for $C_{21}H_{28}NO_5S_2$ ($[M+H]^+$) *m/z* 438.1403, found: 438.1411.

M.P. 127.8 – 129.4 °C.

$[\alpha]_D$ (25.0 °C, c = 1.00 in $CHCl_3$) = +76.515°.

(*R*)-7-(1-(Phenylsulfonyl)pentyl)chroman-4-one (14)

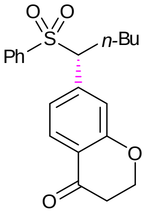

This compound was prepared according to the general procedure with Zn using ((1-chloropentyl)sulfonyl)benzene (37 mg, 0.15 mmol, 1.0 equiv) and 7-bromochroman-4-one (51.1 mg, 0.225 mmol, 1.5 equiv). The crude mixture was purified by flash column chromatograph to afford **14** as an off-white solid (30.1mg, 56% yield, 91% ee).

Chiral HPLC (CHIRALCEL OD-H**,** *i*PrOH-hexanes 20/80, 1.0 mL/min, 254 nm, t_R (minor) = 12.0 min, t_R (major) = 16.3 min.

^{1}H NMR (600 MHz, CDCl$_3$) δ 7.73 (d, *J* = 8.1 Hz, 1H), 7.64 – 7.53 (m, 3H), 7.42 (t, *J* = 7.9 Hz, 2H), 6.80 (d, *J* = 1.7 Hz, 1H), 6.72 (dd, *J* = 8.1, 1.7 Hz, 1H), 4.57 – 4.44 (m, 2H), 4.00 (dd, *J* = 11.7, 3.6 Hz, 1H), 2.79 (dd, *J* = 7.1, 5.8 Hz, 2H), 2.38 – 2.32 (m, 1H), 2.15 – 2.04 (m, 1H), 1.35 – 1.23 (m, 13.7, 11.2, 6.8 Hz, 2H), 1.14 (p, *J* = 7.7 Hz, 2H), 0.81 (t, *J* = 7.3 Hz, 3H).

^{13}C NMR (151 MHz, CDCl$_3$) δ 191.5, 161.7, 141.1, 137.3, 133.9, 129.1, 128.9, 127.3, 122.9, 121.4, 119.5, 71.5, 67.2, 37.8, 28.9, 27.4, 22.4, 13.8.

HRMS (ESI) calcd for $C_{20}H_{23}O_4S$ ($[M+H]^+$) *m/z* 359.1312, found 359.1319.

M.P. 96.4 – 97.8 °C.

$[\alpha]_D$ (25.0 °C, c = 0.5 in $CHCl_3$) = +107.6°.

(*R*)-5-(1-(Phenylsulfonyl)pentyl)-2,3-dihydro-1*H*-inden-1-one (15)

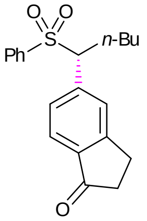

This compound was prepared according to the general procedure with Zn using ((1-chloropentyl)sulfonyl)benzene (37 mg, 0.15 mmol, 1.0 equiv) and 5-bromo-2,3-dihydro-1*H*-inden-1-one (47.5 mg, 0.225 mmol, 1.5 equiv). The crude mixture was purified by flash column chromatograph to afford **15** as an off-white solid (33.4 mg, 65% yield, 89% ee).

Chiral HPLC (CHIRALCEL OD-H**,** *i*PrOH-hexanes 10/90, 1.0 mL/min, 251 nm, t_R (minor) = 19.5 min, t_R (major) = 26 min.

^{1}H NMR (600 MHz, CDCl$_3$) δ 7.61 – 7.52 (m, 4H), 7.44 – 7.37 (m, 2H), 7.32 (s, 1H), 7.06 – 6.99 (m, 1H), 4.11 (dd, *J* = 11.6, 3.7 Hz, 1H), 3.13 – 3.02 (m, 2H), 2.72 – 2.65 (m, 2H), 2.43 – 2.37 (m, 1H), 2.19-2.12 (m, 1H), 1.36 – 1.23 (m, 2H), 1.18- 1.12 (m, 2H), 0.81 (t, *J* = 7.4 Hz, 3H).

^{13}C NMR (151 MHz, CDCl$_3$) δ 206.4, 155.3, 139.6, 137.5, 137.3, 133.8, 129.3, 129.1, 128.9, 128.3, 123.7, 71.8, 36.5, 29.0, 27.5, 25.8, 22.4, 13.8.

HRMS (ESI) calcd. for $C_{20}H_{23}O_3S$ ($[M+H]^+$) m/z 343.1362, found: 343.1366.

M.P. 105.9 – 107.2 °C.

$[\alpha]_D$ (25.0 °C, c = 0.5 in $CHCl_3$) = +90.2°.

(*R*)-5-(1-(Phenylsulfonyl)pentyl)isobenzofuran-1(3*H*)-one (16)

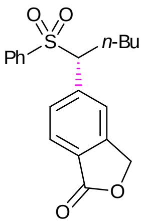

This compound was prepared according to the general procedure with Zn using ((1-chloropentyl)sulfonyl)benzene (37 mg, 0.15 mmol, 1.0 equiv) and 5-bromoisobenzofuran-1(3*H*)-one (47.9 mg, 0.225 mmol, 1.5 equiv). The crude mixture was purified by flash column chromatograph to afford **16** as an off-white solid (26.9 mg, 52% yield, 92% ee).

Chiral HPLC (CHIRALCEL OD-H**,** *i*PrOH-hexanes 20/80, 1.0 mL/min, 240 nm, t_R (minor) = 16.0 min, t_R (major) = 19.6 min.

^{1}H NMR (600 MHz, CDCl$_3$) δ 7.76 (d, *J* = 7.9 Hz, 1H), 7.62 – 7.51 (m, 3H), 7.46 – 7.34 (m, 2H), 7.18 (dd, *J* = 7.9, 1.4 Hz, 1H), 5.27 (q, *J* = 15.3 Hz, 2H), 4.16 (dd, *J* = 11.6, 3.6 Hz, 1H), 2.44 – 2.38 (m, 1H), 2.19 – 2.12 (m, 1H), 1.35 – 1.23 (m, 2H), 1.21 – 1.05 (m, 2H), 0.81 (t, *J* = 7.3 Hz, 3H).

^{13}C NMR (151 MHz, CDCl$_3$) δ 170.4, 146.9, 139.5, 137.2, 134.0, 131.1, 129.1, 129.0, 126.3, 125.8, 123.6, 71.6, 69.6, 29.0, 27.6, 22.3, 13.8.

HRMS (ESI) () calcd. for $C_{19}H_{20}KO_4S$ ($[M+K]^+$) *m/z* 383.0714, found 383.0718.

M.P. 101.5 – 102.9 °C.

$[\alpha]_D$ (25.0 °C, c = 0.5 in $CHCl_3$) = +96.8°.

Methyl (*R*)-2-methoxy-4-(1-(phenylsulfonyl)pentyl)benzoate (17)

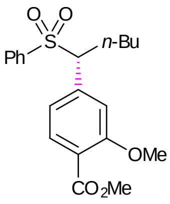

This compound was prepared according to the general procedure with Zn using ((1-chloropentyl)sulfonyl)benzene (37 mg, 0.15 mmol, 1.0 equiv) and methyl 4-bromo-2-methoxybenzoate (55.1 mg, 0.225 mmol, 1.5 equiv). The crude mixture was purified by flash column chromatograph to afford **17** as an off-white solid (23.7 mg, 42% yield, 83% ee).

Chiral HPLC (CHIRALCEL OD-H**,** *i*PrOH-hexanes 10/90, 1.0 mL/min, 240 nm, t_R (minor) = 14.0 min, t_R (major) = 16.6 min.

^{1}H NMR (600 MHz, $CDCl_3$) δ 7.64 (d, *J* = 8.3 Hz, 1H), 7.58 – 7.52 (m, 3H), 7.42 – 7.37 (m, 2H), 6.68 (dt, *J* = 4.3, 2.2 Hz, 2H), 4.02 (dd, *J* = 11.6, 3.6 Hz, 1H), 3.87 (s, 3H), 3.75 (s, 3H), 2.47 – 2.42 (m, 1H), 2.16 – 2.09 (m, 1H), 1.36 – 1.32 (m, 1H), 1.31 – 1.11 (m, 3H), 0.83 (t, *J* = 7.3 Hz, 3H).

^{13}C NMR (151 MHz, $CDCl_3$) δ 166.3, 159.1, 138.6, 137.4, 133.8, 131.8, 129.1, 128.9, 121.7, 120.3, 113.5, 71.7, 56.2, 52.3, 29.0, 27.1, 22.4, 13.8.

HRMS (ESI) calcd. for $C_{20}H_{25}O_5S$ ($[M+H]^+$) *m/z* 377.1417, found 377.1418.

M.P. 95.5 – 97.2 °C.

$[\alpha]_D$ (25.0 °C, c = 1.00 in $CHCl_3$) = + 61.7°.

Methyl (*R*)-2-chloro-4-(1-(phenylsulfonyl)pentyl)benzoate (18)

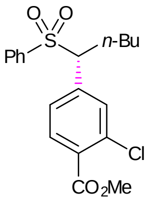

This compound was prepared according to the general procedure with Zn using ((1-chloropentyl)sulfonyl)benzene (37 mg, 0.15 mmol, 1.0 equiv) and methyl 4-bromo-2-chlorobenzoate (56.1 mg, 0.225 mmol, 1.5 equiv). The crude mixture was purified by flash column chromatograph to afford **18** as an off-white solid (17.7 mg, 31% yield, 81% ee).

Chiral HPLC (CHIRALCEL OD-H**,** *i*PrOH-hexanes 20/80, 1.0 mL/min, 210 nm, t_R (minor) = 10.1 min, t_R (major) = 14.8 min.

^{1}H NMR (600 MHz, $CDCl_3$) δ 7.72 (d, *J* = 8.0 Hz, 1H), 7.61 – 7.54 (m, 3H), 7.46 – 7.41 (m, 2H), 7.13 (d, *J* = 1.7 Hz, 1H), 7.10 (dd, *J* = 8.1, 1.8 Hz, 1H), 4.01 (dd, *J* = 11.6, 3.6 Hz, 1H), 3.92 (s, 3H), 2.43 – 2.38 (m, 1H), 2.12 – 2.04 (m, 1H), 1.36 – 1.23 (m, 2H), 1.19 – 1.09 (m, 2H), 0.83 (t, *J* = 7.3 Hz, 3H).

^{13}C NMR (151 MHz, $CDCl_3$) δ 165.7, 138.0, 137.0, 134.1, 134.0, 132.4, 131.6, 130.2, 129.1, 129.1, 128.0, 71.0, 52.7, 28.9, 27.2, 22.3, 13.8.

HRMS (ESI) calcd for $C_{19}H_{22}ClO_4S$ ($[M+H]^+$) *m/z* 381.0922, found 381.0919.

M.P. 83.7 – 85.3 °C.

[α]$_D$ (25.0 °C, c = 0.50 in $CHCl_3$) = +67°.

Methyl (*R*)-2-methyl-4-(1-(phenylsulfonyl)pentyl)benzoate (19)

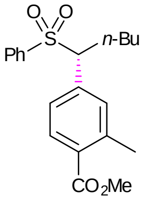

This compound was prepared according to the general procedure with Zn using ((1-chloropentyl)sulfonyl)benzene (37 mg, 0.15 mmol, 1.0 equiv) and methyl 4-bromo-2-methylbenzoate (51.5 mg, 0.225 mmol, 1.5 equiv). The crude mixture was purified by flash column chromatograph to afford **19** as an off-white solid (34.1 mg, 63% yield, 92% ee).

Chiral HPLC (CHIRALCEL OD-H, *i*PrOH-hexanes 10/90, 1.0 mL/min, 240 nm, t_R (minor) = 8.2 min, t_R (major) = 10.1 min.

^{1}H NMR (600 MHz, $CDCl_3$) δ 7.77 (d, *J* = 8.1 Hz, 1H), 7.57 – 7.51 (m, 3H), 7.42 – 7.37 (m, 2H), 6.98 (dd, *J* = 8.1, 1.8 Hz, 1H), 6.94 – 6.90 (m, 1H), 4.01 (dd, *J* = 11.7, 3.6 Hz, 1H), 3.87 (s, 3H), 2.48 (s, 3H), 2.48 – 2.36 (m, 1H), 2.19 – 2.08 (m, 1H), 1.34 – 1.23 (m, 2H), 1.18 – 1.10 (m, 2H), 0.81 (t, *J* = 7.3 Hz, 3H).

^{13}C NMR (151 MHz, $CDCl_3$) δ 167.7, 140.5, 137.3, 136.6, 133.7, 133.3, 130.8, 129.9, 129.2, 128.8, 127.1, 71.4, 52.1, 29.0, 27.1, 22.3, 21.7, 13.8.

HRMS (ESI) calcd. for $C_{20}H_{24}KO_4S$ ($[M+K]^+$) m/z 399.1027, found: 399. 1028.

M.P. 89.6 – 91.5 °C.

[α]$_D$ (25.0 °C, c = 0.68 in $CHCl_3$) = +76.029°.

Methyl (*R*)-3-(1-(phenylsulfonyl)pentyl)benzoate (20)

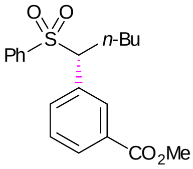

This compound was prepared according to the general procedure with Zn using ((1-chloropentyl)sulfonyl)benzene (37 mg, 0.15 mmol, 1.0 equiv) and methyl 3-bromobenzoate (48.4 mg, 0.225 mmol, 1.5 equiv). The crude mixture was purified by flash column chromatograph to afford **20** as an off-white solid (21.3 mg, 41% yield, 86% ee).

Chiral HPLC (CHIRALCEL OD-H, *i*PrOH-hexanes 10/90, 1.0 mL/min, 254 nm, t_R (minor) = 10.4 min, t_R (major) = 12.7 min.

^{1}H NMR (600 MHz, $CDCl_3$) δ 7.98 – 2.94 (m, 1H), 7.69 (d, *J* = 1.9 Hz, 1H), 7.56 – 7.49 (m, 3H), 7.41

– 7.31 (m, 4H), 4.08 (dd, *J* = 11.6, 3.6 Hz, 1H), 3.88 (s, 3H), 2.45 – 2.39 (m, 1H), 2.19 – 2.13 (m, 1H), 1.35 – 1.25 (m, 2H), 1.20 – 1.11 (m, 2H), 0.82 (t, *J* = 7.3 Hz, 3H).

^{13}C NMR (151 MHz, $CDCl_3$) δ) δ 167.4, 138.1, 134.0, 133.4, 132.3, 131.8, 130.7, 129.6, 129.0, 128.6, 128.3, 63.8, 52.2, 28.7, 27.6, 22.4, 13.8.

HRMS (ESI) calcd. for $C_{19}H_{26}NO_4S$ ($[M+NH_4]^+$) *m/z* 364.1577, found 364.1574.

M.P. 73.4 – 74.9 °C.

$[\alpha]_D$ (25.0 °C, c = 0.5 in $CHCl_3$) = +39°.

(*R*)-((1-Phenylpentyl)sulfonyl)benzene (21) [3]

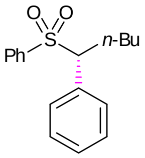

This compound was prepared according to the general procedure with Zn using ((1-chloropentyl)sulfonyl)benzene (37 mg, 0.15 mmol, 1.0 equiv) and iodobenzene (35,3 mg, 0.225 mmol, 1.5 equiv). The crude mixture was purified by flash column chromatograph to afford **21** as an off-white solid (45.9 mg, 80% yield, 89% ee).

Chiral HPLC (CHIRALCEL OD-H**,** *i*PrOH-hexanes 5/95, 1.0 mL/min, 230 nm, t_R (minor) = 8.9 min, t_R (major) = 11.6 min.

^{1}H NMR (600 MHz, $CDCl_3$) δ 7.54-7.50 (m, , 3H), 7.36 (t, *J* = 7.7 Hz, 2H), 7.28 (d, *J* = 7.4 Hz, 1H), 7.22 (t, *J* = 7.5 Hz, 2H), 7.08 (d, *J* = 7.4 Hz, 2H), 4.02 (dd, *J* = 11.7, 3.6 Hz, 1H), 2.45 – 2.39 (m, 1H), 2.20 – 2.09 (m, 1H), 1.38 – 1.23 (m, 2H), 1.22 -1.15 (m, 2H), 0.83 (t, *J* = 7.3 Hz, 3H).

^{13}C NMR (151 MHz, $CDCl_3$) δ 137.6, 133.5, 132.5, 130.0, 129.1, 128.8, 128.7, 128.5, 71.8, 29.0, 27.1, 22.4, 13.8.

M.P. 76.9 – 78.6 °C.

$[\alpha]_D$ (25.0 °C, c = 1.00 in $CHCl_3$) = +71.7°.

Lit: $[\alpha]$ (25.0 °C, c = 1.08 in $CHCl_3$) = -78° (*S* enantiomer, 84% ee).[3] Based on the literature precedent, we assign our product as the *R* enantiomer.

(*R*)-1-Methyl-4-(1-(phenylsulfonyl)pentyl)benzene (22)

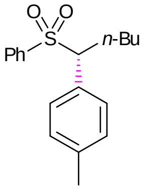

This compound was prepared according to the general procedure with Zn using ((1-chloropentyl)sulfonyl)benzene (37 mg, 0.15 mmol, 1.0 equiv) and 1-iodo-4-methylbenzene (38.5 mg, 0.225 mmol, 1.5 equiv). The crude mixture was purified by flash column chromatograph to afford **22** as an off-white solid (49.1 mg, 70% yield, 89% ee).

Chiral HPLC (CHIRALCEL OD-H**,** *i*PrOH-hexanes 5/95, 1.0 mL/min, 210 nm, t_R (minor) = 8.2 min, t_R (major) = 10.0 min.

^{1}H NMR (600 MHz, $CDCl_3$) δ 7.57 – 7.49 (m, 3H), 7.37 (dd, *J* = 8.5, 7.1 Hz, 2H), 7.03 (d, *J* = 7.8 Hz, 2H), 7.00 – 6.94 (m, 2H), 3.98 (dd, *J* = 11.7, 3.6 Hz, 1H), 2.43 – 2.34 (m, 1H), 2.31 (s, 3H), 2.16 – 2.05 (m, 1H), 1.36 – 1.22 (m, 2H), 1.21 – 1.10 (m, 2H), 0.81 (t, *J* = 7.3 Hz, 3H).

^{13}C NMR (151 MHz, $CDCl_3$) δ 138.7, 137.67, 133.4, 129.8, 129.3, 129.3, 129.2, 128.7, 71.4, 29.0 27.1, 22.4, 21.3, 13.8.

HRMS (ESI) calcd for $C_{18}H_{22}NaO_2S$ ([M+Na]$^+$) m/z 325.1233, found: 325.1240.

M.P. 80.5 – 81.8 °C.

$[\alpha]_D$ (25.0 °C, c = 0.52 in $CHCl_3$) = +78.654°.

(*R*)-1-Ethyl-4-(1-(phenylsulfonyl)pentyl)benzene (23)

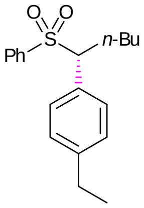

This compound was prepared according to the general procedure with Zn using ((1-chloropentyl)sulfonyl)benzene (37 mg, 0.15 mmol, 1.0 equiv) and 1-ethyl-4-iodo-benzene (52.2 mg, 0.225 mmol, 1.5 equiv). The crude mixture was purified by flash column chromatograph to afford **23** as an off-white solid (29 mg, 61% yield, 88% ee).

Chiral HPLC (CHIRALCEL OD-H**,** *i*PrOH-hexanes 5/95, 1.0 mL/min, 240 nm, t_R (minor) = 7.3 min, t_R (major) = 8.5 min.

^{1}H NMR (600 MHz, $CDCl_3$) δ 7.56 – 7.47 (m, 3H), 7.39 – 7.33 (m, 2H), 7.05 (d, *J* = 8.0 Hz, 2H), 7.01 – 6.95 (m, 2H), 3.99 (dd, *J* = 11.6, 3.6 Hz, 1H), 2.60 (q, *J* = 7.6 Hz, 2H), 2.40 – 2.35 (m, 1H), 2.15 – 2.09 (m, 1H), 1.35 – 1.26 (m, 2H), 1.23 – 1.13 (m, 5H), 0.82 (t, *J* = 7.3 Hz, 3H).

^{13}C NMR (151 MHz, $CDCl_3$) δ 145.1, 137.7, 133.4, 129.9, 129.5, 129.2, 128.6, 128.1, 71.5, 29.0, 28.7, 27.1, 22.4, 15.6, 13.9.

HRMS (ESI) calcd for $C_{19}H_{24}NaO_2S$ ([M+Na]$^+$) *m/z* 339.1389, found: 339.1394.

M.P. 65.9 – 67.6 °C.

$[\alpha]_D$ (25.0 °C, c = 0.5 in $CHCl_3$) = +71.4°.

(*R*)-1-Methoxy-4-(1-(phenylsulfonyl)pentyl)benzene (24)

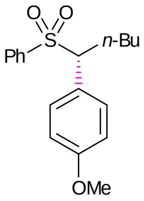

This compound was prepared according to the general procedure with Zn using ((1-chloropentyl)sulfonyl)benzene (37 mg, 0.15 mmol, 1.0 equiv) and 1-iodo-4-methoxybenzene (42.1 mg, 0.225 mmol, 1.5 equiv). The crude mixture was purified by flash column chromatograph to afford **24** as an off-white solid (52.7 mg, 72% yield, 88% ee).

Chiral HPLC (CHIRALCEL OD-H**,** *i*PrOH-hexanes 10/90, 1.0 mL/min, 240 nm, t_R (minor) = 8.5 min, t_R (major) = 10.4 min.

^{1}H NMR (600 MHz, $CDCl_3$) δ 7.57 – 7.48 (m, 3H), 7.41 – 7.34 (m, 2H), 7.03 – 6.96 (m, 2H), 6.79 – 6.73 (m, 2H), 3.97 (dd, *J* = 11.7, 3.6 Hz, 1H), 3.78 (s, 3H), 2.41 – 2.35 (m, 1H), 2.14 – 2.03 (m, 1H), 1.37 – 1.23 (m, 2H), 1.19 – 1.13 (m, 2H), 0.82 (t, *J* = 7.3 Hz, 3H).

^{13}C NMR (151 MHz, $CDCl_3$) δ 160.0, 137.7, 133.4, 131.1, 129.2, 128.7, 124.2, 114.0, 71.1, 55.4, 29.0, 27.1, 22.4, 13.9.

HRMS (ESI) calcd for $C_{18}H_{22}NaO_3S$ ($[M+Na]^+$) *m/z* 341.1182, found 341.1189.

M.P. 81.5 – 83.9 °C.

$[\alpha]_D$ (25.0 °C, c = 1.1 in $CHCl_3$) = +81.091°.

***tert*-Butyl (*R*)-(4-(1-(phenylsulfonyl)pentyl)phenyl)carbamate (25)**

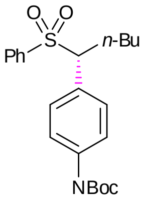

This compound was prepared according to the general procedure with Zn using ((1-chloropentyl)sulfonyl)benzene (37 mg, 0.15 mmol, 1.0 equiv) and *tert*-butyl (4-iodophenyl)carbamate (71.8 mg, 0.225 mmol, 1.5 equiv). The crude mixture was purified by flash column chromatograph to afford **25** as an off-white solid (27.9 mg, 46% yield, 90% ee).

Chiral HPLC (CHIRALCEL OD-H**,** *i*PrOH-hexanes 5/95, 1.0 mL/min, 254 nm, t_R (minor) = 21.8 min, t_R (major) = 23.4 min.

^{1}H NMR (600 MHz, $CDCl_3$) δ 7.56 – 7.49 (m, 3H), 7.38 (dd, *J* = 8.5, 7.1 Hz, 2H), 7.23 (d, *J* = 8.2 Hz, 2H), 7.01 – 6.96 (m, 2H), 6.53 (s, 1H), 3.97 (dd, *J* = 11.7, 3.5 Hz, 1H), 2.41 – 2.35 (m, 1H), 2.14 – 2.01 (m, 1H), 1.50 (s, 9H), 1.35 – 1.21 (m, 3H), 1.19 – 1.10 (m, 2H), 0.81 (t, *J* = 7.3 Hz, 3H).

^{13}C NMR (151 MHz, CDCl$_3$) δ 152.6, 138.9, 137.6, 133.5, 130.6, 129.2, 128.7, 126.6, 118.2, 80.9, 71.2, 29.0, 28.4, 27.1, 22.4, 13.9.

HRMS (ESI) calcd for $C_{22}H_{29}NNaO_4S$ ($[M+Na]^+$) *m/z* 426.1710, found 426.1712.

M.P. 154.4 – 155.8 °C.

$[\alpha]_D$ (25.0 °C, c = 0.5 in $CHCl_3$) = +74.8°.

(*R*)-4,4,5,5-Tetramethyl-2-(4-(1-(phenylsulfonyl)pentyl)phenyl)-1,3,2-dioxaborolane (26)

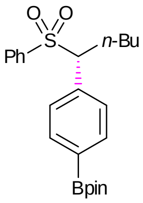

This compound was prepared according to the general procedure with Zn using ((1-chloropentyl)sulfonyl)benzene (37 mg, 0.15 mmol, 1.0 equiv) and 2-(4-iodophenyl)-4,4,5,5-tetramethyl-1,3,2-dioxaborolane (74.2 mg, .225 mmol, 1.5 equiv). The crude mixture was purified by flash column chromatograph to afford **26** as an off-white solid (27.4 mg, 44% yield, 90% ee).

Chiral HPLC (CHIRALCEL OD-H**,** *i*PrOH-hexanes 10/90, 1.0 mL/min, 230 nm, t_R (minor) = 5.6 min, t_R (major) = 6.2 min.

^{1}H NMR (600 MHz, CDCl$_3$) δ 7.65 (d, *J* = 7.7 Hz, 2H), 7.55 – 7.48 (m, 3H), 7.37 (dd, *J* = 8.6, 7.1 Hz, 2H), 7.09 (d, *J* = 7.7 Hz, 2H), 4.02 (dd, *J* = 11.7, 3.6 Hz, 1H), 2.45 – 2.39 (m, 1H), 2.17 – 2.11 (m, 1H), 1.34 (s, 14H), 1.18 – 1.11 (m, 2H), 0.81 (t, *J* = 7.3 Hz, 3H).

^{13}C NMR (151 MHz, CDCl$_3$) δ 137.5, 135.6, 134.9, 133.6, 129.3, 129.2, 128.8, 84.1, 71.9, 29.0, 27.2, 25.0, 22.4, 13.8.

HRMS (ESI) calcd for $C_{23}H_{31}BKO_4S$ ($[M+K]^+$) *m/z* 453.1668, found 453.1676.

$[\alpha]_D$ (25.0 °C, c = 0.4 in $CHCl_3$) = +60.5°.

(*R*)-(4-(1-(Phenylsulfonyl)pentyl)phenyl)methanol (27)

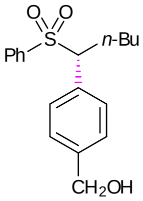

This compound was prepared according to the general procedure with Zn using ((1-chloropentyl)sulfonyl)benzene (37 mg, 0.15 mmol, 1.0 equiv) and (4-iodophenyl)-methanol (52.7 mg, 0.225 mmol, 1.5 equiv). The crude mixture was purified by flash column chromatograph to afford **27** as an off-white solid (30.1 mg, 63% yield, 90% ee).

Chiral HPLC (CHIRALCEL OD-H**,** *i*PrOH-hexanes 20/80, 1.0 mL/min, 240 nm, t_R (minor) = 7.5 min, t_R (major) = 11.5 min.

^{1}H NMR (600 MHz, CDCl$_3$) δ 7.59 – 7.48 (m, 3H), 7.38 (t, *J* = 7.8 Hz, 2H), 7.23 (d, *J* = 7.9 Hz, 2H), 7.09 (d, *J* = 7.9 Hz, 2H), 4.66 (s, 2H), 4.02 (dd, *J* = 11.7, 3.5 Hz, 1H), 2.4 – 2.34 (m, 1H), 2.15 – 2.08 (m, 1H), 1.85 (s, 1H), 1.35 – 1.21 (m, 2H), 1.18 – 1.12 (m, 2H), 0.81 (t, *J* = 7.3 Hz, 3H).

^{13}C NMR (151 MHz, CDCl$_3$) δ 141.6, 137.5, 133.6, 131.7, 130.2, 129.1, 128.8, 127.0, 71.4, 64.9, 29.0, 27.2, 22.4, 13.8.

HRMS (ESI) calcd for $C_{18}H_{22}KO_3S$ ($[M+K]^+$) *m/z* 357.0921, found 357.0925.

M.P. 88 – 89.5 °C.

$[\alpha]_D$ (25.0 °C, c = 0.5 in CHCl$_3$) = +79.8°.

(*R*)-2-(4-(1-(Phenylsulfonyl)pentyl)phenyl)ethan-1-ol (28)

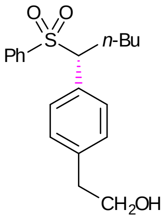

This compound was prepared according to the general procedure with Zn using ((1-chloropentyl)sulfonyl)benzene (37 mg, 0.15 mmol, 1.0 equiv) and 2-(4-iodophenyl)ethan-1-ol (55.8 mg, 0.225 mmol, 1.5 equiv). The crude mixture was purified by flash column chromatograph to afford **28** as an off-white solid (30.9mg, 62% yield, 89% ee).

Chiral HPLC (CHIRALCEL OD-H, *i*PrOH-hexanes 20/80, 1.0 mL/min, 240 nm, t_R (minor) = 7.4 min, t_R (major) = 9.5 min.

^{1}H NMR (600 MHz, CDCl$_3$) δ 7.56 – 7.49 (m, 3H), 7.40 – 7.35 (m, 2H), 7.09 (d, *J* = 8.0 Hz, 2H), 7.04 (d, *J* = 8.1 Hz, 2H), 4.00 (dd, *J* = 11.6, 3.6 Hz, 1H), 3.82 (t, *J* = 6.6 Hz, 2H), 2.83 (t, *J* = 6.6 Hz, 2H), 2.39 – 2.32 (m, 1H), 2.15-2.10 (m, 1H), 1.35 – 1.26 (m, 3H), 1.19 – 1.14 (m, 2H), 0.81 (t, *J* = 7.3 Hz, 3H).

^{13}C NMR (151 MHz, CDCl$_3$) δ 139.4, 137.6, 133.5, 130.5, 130.2, 129.2, 129.1, 128.7, 71.4, 63.6, 39.0, 29.0, 27.2, 22.4, 13.8.

HRMS (ESI) calcd. for $C_{19}H_{24}NaO_3S$ ($[M+Na]^+$) *m/z* 355.1338, found 355.1342.

M.P. 72.7 – 74.5 °C.

$[\alpha]_D$ (25.0 °C, c = 1.00 in CHCl$_3$) = +70.4°.

Methyl (*R*)-2-(4-(1-(phenylsulfonyl)pentyl)phenyl)acetate (29)

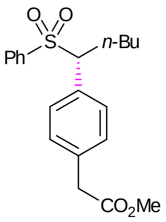

This compound was prepared according to the general procedure with Zn using ((1-chloropentyl)sulfonyl)benzene (37 mg, 0.15 mmol, 1.0 equiv) and methyl 2-(4-iodophenyl)acetate (66.1 mg, 0.225 mmol, 1.5 equiv). The crude mixture was purified by flash column chromatograph to afford **29** as an off-white solid (29.7 mg, 55% yield, 92% ee).

Chiral HPLC (CHIRALCEL OD-H**,** *i*PrOH-hexanes 20/80, 1.0 mL/min, 240 nm, t_R (minor) = 7.3 min, t_R (major) = 9.2 min.

^{1}H NMR (600 MHz, CDCl$_3$) δ 7.56 – 7.47 (m, 3H), 7.40 – 7.34 (m, 2H), 7.15 (d, *J* = 8.0 Hz, 2H), 7.08 – 7.02 (m, 2H), 4.00 (dd, *J* = 11.6, 3.6 Hz, 1H), 3.70 (s, 3H), 3.59 (s, 2H), 2.41 – 2.35 (m, 1H), 2.15 – 2.08 (m, 1H), 1.35 – 1.23 (m, 2H), 1.20 – 1.13 (m, 2H), 0.82 (t, *J* = 7.3 Hz, 3H).

^{13}C NMR (151 MHz, CDCl$_3$) δ 171.8, 137.5, 134.7, 133.5, 131.3, 130.2, 129.5, 129.2, 128.7, 71.4, 52.2, 40.9, 29.0, 27.2, 22.4, 13.8.

HRMS (ESI) calcd. for $C_{20}H_{24}NaO_4S$ ([M+Na]$^+$) *m/z* 383.1288, found 383.1289.

M.P. 76.9 – 78.6 °C.

[α]$_D$ (25.0 °C, c = 0.9 in CHCl$_3$) = +59.222°.

(*R*)-1,2-Dimethyl-4-(1-(phenylsulfonyl)pentyl)benzene (30)

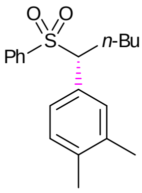

This compound was prepared according to the general procedure with Zn using ((1-chloropentyl)sulfonyl)benzene (37 mg, 0.15 mmol, 1.0 equiv) and 4-iodo-1,2-dimethylbenzene (55.2 mg, 0.225 mmol, 1.5 equiv). The crude mixture was purified by flash column chromatograph to afford **30** as an off-white solid (29 mg, 61% yield, 85% ee).

Chiral HPLC (CHIRALCEL OD-H**,** *i*PrOH-hexanes 5/95, 1.0 mL/min, 254 nm, t_R (minor) = 7.6 min, t_R (major) = 8.8 min.

^{1}H NMR (600 MHz, CDCl$_3$) δ 7.57 – 7.50 (m, 3H), 7.42 – 7.35 (m, 2H), 6.98 (d, *J* = 7.7 Hz, 1H), 6.83 (d, *J* = 1.9 Hz, 1H), 6.79 (dd, *J* = 7.7, 2.0 Hz, 1H), 3.95 (dd, *J* = 11.7, 3.6 Hz, 1H), 2.39 – 2.29 (m, 1H), 2.21 (s, 3H), 2.15 (s, 3H), 2.13 – 2.05 (m, 1H), 1.34 – 1.26 (m, 2H), 1.21 – 1.11 (m, 2H), 0.81 (t, *J* = 7.3 Hz, 3H).

^{13}C NMR (151 MHz, CDCl$_3$) δ 137.7, 137.3, 136.8, 133.4, 131.2, 129.7, 129.50, 129.3, 128.6, 127.3, 71.5, 29.0, 27.2, 22.4, 19.8, 19.6, 13.8.

HRMS (ESI) calcd. for $C_{19}H_{25}O_2S$ ($[M+NH_4]^+$) *m/z* 334.1835, found 334.1841..

M.P. 111.1 – 112.9 °C.

$[\alpha]_D$ (25.0 °C, c = 0.6 in $CHCl_3$) = +65.167°.

(*R*)-1-Methoxy-3-(1-(phenylsulfonyl)pentyl)benzene (31)

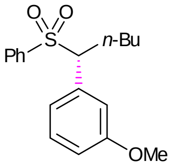

This compound was prepared according to the general procedure with Zn using ((1-chloropentyl)sulfonyl)benzene (37 mg, 0.15 mmol, 1.0 equiv) and 1-iodo-3-methoxybenzene (52.7 mg, 0.225 mmol, 1.5 equiv). The crude mixture was purified by flash column chromatograph to afford **31** as an off-white solid (20.1 mg, 42% yield, 86% ee).

Chiral HPLC (CHIRALCEL OD-H**,** *i*PrOH-hexanes 10/90, 1.0 mL/min, 210 nm, t_R (minor) = 7.4 min, t_R (major) = 9.5 min.

^{1}H NMR (600 MHz, CDCl$_3$) δ 7.53 (dd, *J* = 7.7, 6.2 Hz, 3H), 7.38 (dd, *J* = 8.8, 6.9 Hz, 2H), 7.12 (t, *J* = 7.9 Hz, 1H), 6.81 (dd, *J* = 8.2, 2.6 Hz, 1H), 6.66 (d, *J* = 7.7 Hz, 1H), 6.62 (t, *J* = 2.1 Hz, 1H), 3.98 (dd, *J* = 11.6, 3.6 Hz, 1H), 3.70 (s, 3H), 2.43 – 2.37 (m, 1H), 2.19 – 2.06 (m, 1H), 1.36 – 1.26 (m, 2H), 1.22 – 1.16 (m, 2H), 0.83 (t, *J* = 7.3 Hz, 3H).

^{13}C NMR (151 MHz, CDCl$_3$) δ 159.6, 137.6, 134.0, 133.5, 129.5, 129.2, 128.7, 122.4, 115.3, 114.5, 71.8, 55.4, 29.0, 27.2, 22.4, 13.9.

HRMS (ESI) calcd. for $C_{18}H_{22}NaO_3S$ ($[M+Na]^+$) *m/z* 341.1182, found 341.1188.

M.P. 97.2 – 98.8 °C.

$[\alpha]_D$ (25.0 °C, c = 0.5 in $CHCl_3$) = +91.8°.

(*R*)-2-(1-(Phenylsulfonyl)pentyl)naphthalene (32)

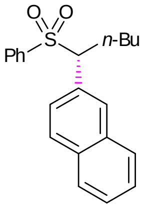

This compound was prepared according to the general procedure with Zn using ((1-chloropentyl)sulfonyl)benzene (37 mg, 0.15 mmol, 1.0 equiv) and 2-iodonaphthalene (57.1 mg, 0.225 mmol, 1.5 equiv). The crude mixture was purified by flash column chromatograph to afford **32** as an off-white solid (26.9 mg, 53% yield, 90% ee).

Chiral HPLC (CHIRALCEL OD-H**,** *i*PrOH-hexanes 10/90, 1.0 mL/min, 230 nm, t_R (minor) = 8.0 min, t_R (major) = 9.8 min.

^{1}H NMR (600 MHz, CDCl$_3$) δ 7.83 – 7.78 (m, 1H), 7.73 (d, *J* = 8.5 Hz, 1H), 7.71 – 7.66 (m, 1H), 7.56 – 7.42 (m, 6H), 7.31 (t, *J* = 7.8 Hz, 2H), 7.26 – 7.24 (s, 1H), 4.19 (dd, *J* = 11.7, 3.6 Hz, 1H), 2.51 –

2.46 (m, 1H), 2.32 – 2.21 (m, 1H), 1.38 – 1.25 (m, 2H), 1.22 - 1.15 (m, 2H), 0.81 (t, *J* = 7.3 Hz, 3H).

^{13}C NMR (151 MHz, $CDCl_3$) δ 137.5, 133.5, 133.3, 133.1, 130.0, 129.9, 129.2, 128.7, 128.3, 128.1, 127.8, 126.8, 126.7, 126.4, 71.9, 29.1, 27.2, 22.4, 13.9.

HRMS (ESI) calcd. for $C_{21}H_{26}NO_2S$ ($[M+NH_4]^+$) *m/z* 356.1679, found 356.1684.

M.P. 110.5 – 102.3 °C.

$[\alpha]_D$ (25.0 °C, c = 0.5 in $CHCl_3$) = +79.6°.

tert-Butyl (R)-6-(1-(phenylsulfonyl)pentyl)-1H-indole-1-carboxylate (33)

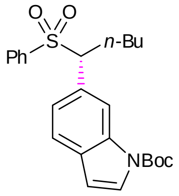

This compound was prepared according to the general procedure with Mn using ((1-chloropentyl)sulfonyl)benzene (37 mg, 0.15 mmol, 1.0 equiv) and *tert*-butyl 6-iodo-1*H*-indole-1-carboxylate (77.2 mg, 0.225 mmol, 1.5 equiv). The crude mixture was purified by flash column chromatograph to afford **33** as an off-white solid (40.4 mg, 63% yield, 87% ee).

Chiral HPLC (CHIRALCEL OD-H**,** *i*PrOH-hexanes 10/90, 0.5 mL/min, 254 nm, t_R (minor) = 13.9 min, t_R (major) = 15.2 min.

^{1}H NMR (600 MHz, $CDCl_3$) δ 7.87 (s, 1H), 7.59 (s, 1H), 7.56 – 7.47 (m, 3H), 7.42 (d, *J* = 8.0 Hz, 1H), 7.34 (t, *J* = 7.8 Hz, 2H), 7.00 (d, *J* = 8.2 Hz, 1H), 6.53 (d, *J* = 3.7 Hz, 1H), 4.15 (dd, *J* = 11.7, 3.6 Hz, 1H), 2.46- 2.41 (m, 1H), 2.25 – 2.13 (m, 1H), 1.64 (s, 9H), 1.36 – 1.28 (m, 2H), 1.26 – 1.7 (m, 2H), 0.81 (t, *J* = 7.3 Hz, 3H).

^{13}C NMR (151 MHz, $CDCl_3$) δ 149.6, 137.7, 135.1, 133.3, 131.0, 129.2, 128.6, 128.2, 126.9, 124.3, 120.9, 117.1, 107.1, 84.0, 72.3, 29.1, 28.3, 27.6, 22.4, 13.9.

HRMS (ESI) calcd. for $C_{24}H_{29}NNaO_4S$ ($[M+Na]^+$) *m/z* 450.1710, found 450.1709.

M.P. 110.8 – 112.2 °C.

$[\alpha]_D$ (22.0 °C, c = 0.5 in $CHCl_3$) = +54.6°.

tert-Butyl (R)-5-(1-(phenylsulfonyl)pentyl)-1H-indole-1-carboxylate (34)

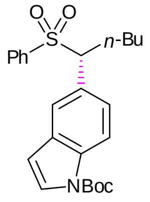

This compound was prepared according to the general procedure with Mn using ((1-chloropentyl)sulfonyl)benzene (37 mg, 0.15 mmol, 1.0 equiv) and *tert*-butyl 5-iodo-1*H*-indole-1-carboxylate (77.2 mg, 0.225 mmol, 1.5 equiv). The crude mixture

was purified by flash column chromatograph to afford **34** as an off-white solid (34 mg, 53% yield, 82% ee).

Chiral HPLC (CHIRALCEL OD-H**,** *i*PrOH-hexanes 10/90, 1.0 mL/min, 240 nm, t_R (minor) = 6.7 min, t_R (major) = 7.9 min.

^{1}H NMR (600 MHz, CDCl$_3$) δ 7.99 (d, *J* = 8.6 Hz, 1H), 7.65 – 7.45 (m, 4H), 7.41 – 7.29 (m, 3H), 7.02 (d, *J* = 8.6 Hz, 1H), 6.47 (d, *J* = 3.7 Hz, 1H), 4.14 – 4.08 (m, 1H), 2.50 – 2.40 (m, 1H), 2.24 – 2.13 (m, 1H), 1.66 (s, 9H), 1.36 – 1.23 (m, 2H), 1.19- 1.14 (m, 2H), 0.80 (t, *J* = 7.3 Hz, 3H).

^{13}C NMR (151 MHz, CDCl$_3$) δ 149.7, 137.7, 135.3, 133.4, 130.7, 129.2, 128.7, 126.7, 126.6, 126.0, 122.60, 115.2, 107.3, 84.1, 71.7, 29.0, 28.3, 27.5, 22.4, 13.9.

HRMS (ESI) calcd. for $C_{24}H_{29}NNaO_4S$ ($[M+Na]^+$) *m/z* 450.1710, found 450.1710.

M.P. 69.1 – 70.8 °C.

$[\alpha]_D$ (22.0 °C, c = 0.3 in $CHCl_3$) = +130.333°.

***tert*-Butyl (*R*)-3-(1-(phenylsulfonyl)pentyl)-9*H*-carbazole-9-carboxylate (35)**

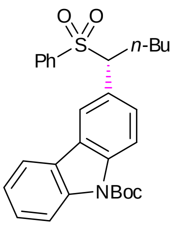

This compound was prepared according to the general procedure with Mn using ((1-chloropentyl)sulfonyl)benzene (37 mg, 0.15 mmol, 1.0 equiv) and *tert*-butyl 3-iodo-9*H*-carbazole-9-carboxylate (88.5 mg, 0.225 mmol, 1.5 equiv). The crude mixture was purified by flash column chromatograph to afford **35** as an off-white solid (36.5 mg, 51% yield, 85% ee).

Chiral HPLC (CHIRALCEL OD-H**,** *i*PrOH-hexanes 10/90, 1.0 mL/min, 254 nm, t_R (minor) = 7.1 min, t_R (major) = 8.0 min.

^{1}H NMR (600 MHz, CDCl$_3$) δ 8.28 (d, *J* = 8.4 Hz, 1H), 8.16 (d, *J* = 8.6 Hz, 1H), 7.88 (d, *J* = 7.7 Hz, 1H), 7.71 (s, 1H), 7.59 – 7.52 (m, 2H), 7.49 (ddd, *J* = 13.8, 10.2, 7.3 Hz, 2H), 7.38 – 7.31 (m, 3H), 7.17 (d, *J* = 8.6 Hz, 1H), 4.19 (dd, *J* = 11.9, 3.5 Hz, 1H), 2.53 – 2.48 (m, 1H), 2.30 – 2.20 (m, 1H), 1.75 (s, 9H), 1.38 – 1.26 (m, 2H), 1.23-1.17 (m, 2H), 0.81 (td, *J* = 7.3, 1.4 Hz, 3H).

^{13}C NMR (151 MHz, CDCl$_3$) δ 151.0, 138.9, 138.7, 137.6, 133.5, 129.2, 128.8, 128.7, 127.6, 127.1, 126.0, 125.3, 123.3, 121.0, 119.9, 116.4, 116.3, 84.4, 71.7, 29.1, 28.5, 27.5, 22.4, 13.9.

HRMS (ESI) calcd. for $C_{28}H_{31}NNaO_4S$ ($[M+Na]^+$) *m/z* 500.1866, found 500.1868.

M.P. 104.4 – 106.1 °C.

$[\alpha]_D$ (22.0 °C, c = 0.5 in $CHCl_3$) = +1.2.6°.

(*R*)-2-Methoxy-5-(1-(phenylsulfonyl)pentyl)pyridine (36)

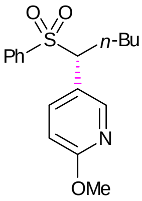

This compound was prepared according to the general procedure with Mn using ((1-chloropentyl)sulfonyl)benzene (37 mg, 0.15 mmol, 1.0 equiv) and 5-iodo-2-methoxypyridine (52.9 mg, 0.225 mmol, 1.5 equiv). The crude mixture was purified by flash column chromatograph to afford **36** as an off-white solid (19.2 mg, 41% yield, 87% ee; with Zn instead of Mn 21% yield, 87% ee).

Chiral HPLC (CHIRALCEL OD-H***,*** *i*PrOH-hexanes 10/90, 1.0 mL/min, 254 nm, t_R (minor) = 8.3 min, t_R (major) = 10.2 min.

<u>^{1}H NMR (600 MHz, CDCl$_3$)</u> δ 7.65 (d, *J* = 2.5 Hz, 1H), 7.56 (dd, *J* = 8.1, 2.3 Hz, 3H), 7.49 (dd, *J* = 8.7, 2.5 Hz, 1H), 7.42 (t, *J* = 7.7 Hz, 2H), 6.69 (d, *J* = 8.6 Hz, 1H), 3.96 (dd, *J* = 11.8, 3.6 Hz, 1H), 3.88 (s, 3H), 2.41 – 2.36 (m, 1H), 2.12 – 2.00 (m, 1H), 1.34 – 1.22 (m, 2H), 1.18 – 1.13 (m, 2H), 0.82 (t, *J* = 7.3 Hz, 3H).

<u>^{13}C NMR (151 MHz, CDCl$_3$)</u> δ 164.5, 148.6, 139.1, 137.3, 133.8, 129.1, 129.0, 121.1, 111.1, 68.5, 53.7, 28.9, 26.8, 22.3, 13.8.

<u>HRMS</u> (ESI) calcd. for $C_{17}H_{21}NNaO_3S$ ($[M+Na]^+$) *m/z* 342.1134, found 342.139.

<u>M.P.</u> 76.5 – 78.1 °C.

$[\alpha]_D$ (22.0 °C, c = 0.5 in $CHCl_3$) = +59.8°.

(*R*)-2-Methoxy-4-(1-(phenylsulfonyl)pentyl)pyridine (37)

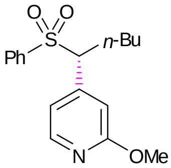

This compound was prepared according to the general procedure with Mn using ((1-chloropentyl)sulfonyl)benzene (37 mg, 0.15 mmol, 1.0 equiv) and 4-bromo-2-methoxypyridine (42.3 mg, 0.225 mmol, 1.5 equiv). The crude mixture was purified by flash column chromatograph to afford **37** as an off-white solid (22.1 mg, 46% yield, 87% ee).

Chiral HPLC (CHIRALCEL OD-H***,*** *i*PrOH-hexanes 10/90, 1.0 mL/min, 230 nm, t_R (minor) = 7.3 min, t_R (major) = 10.9 min.

<u>^{1}H NMR (600 MHz, CDCl$_3$)</u> δ 8.05 – 8.01 (m, 1H), 7.63 – 7.55 (m, 3H), 7.47 – 7.39 (m, 2H), 6.66 (dd, *J* = 5.3, 1.5 Hz, 1H), 6.47 – 6.43 (m, 1H), 3.94 (dd, *J* = 11.6, 3.5 Hz, 1H), 3.88 (s, 3H), 2.39 – 2.33 (m, 1H), 2.11 – 2.04 (m, 1H), 1.37 – 1.20 (m, 2H), 1.17 – 1.12 (m, 2H), 0.82 (t, *J* = 7.3 Hz, 3H).

<u>^{13}C NMR (151 MHz, CDCl$_3$)</u> δ 164.5, 147.2, 144.5, 137.2, 134.0, 129.1, 129.0, 117.6, 112.2, 70.8,

53.7, 28.9, 27.1, 22.4, 13.8.

HRMS (ESI) calcd. for $C_{17}H_{21}NNaO_3S$ ($[M+Na]^+$) *m/z* 342.1134, found 342.1139.

M.P. 64.3 – 65.9 °C.

$[\alpha]_D$ (22.0 °C, c = 0.5 in $CHCl_3$) = +74.8°.

(*R*)-4-(5-(1-(Phenylsulfonyl)pentyl)pyridin-2-yl)morpholine (38)

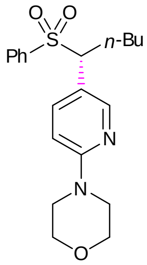

This compound was prepared according to the general procedure with Mn using ((1-chloropentyl)sulfonyl)benzene (37 mg, 0.15 mmol, 1.0 equiv) and 4-(5-iodopyridin-2-yl)morpholine (66.3 mg, 0.225 mmol, 1.5 equiv). The crude mixture was purified by flash column chromatograph to afford **38** as an off-white solid (21.4 mg, 38% yield, 91% ee).

Chiral HPLC (CHIRALCEL OD-H**,** *i*PrOH-hexanes 20/80, 1.0 mL/min, 254 nm, t_R (minor) = 11.4 min, t_R (major) = 13.7 min.

^{1}H NMR (600 MHz, $CDCl_3$) δ 7.68 (d, *J* = 2.5 Hz, 1H), 7.63 – 7.54 (m, 3H), 7.43 (ddd, *J* = 8.5, 6.3, 1.9 Hz, 3H), 6.57 (d, *J* = 8.8 Hz, 1H), 3.92 (dd, *J* = 11.8, 3.6 Hz, 1H), 3.84 – 3.75 (m, 4H), 3.48 (dd, *J* = 5.9, 3.9 Hz, 4H), 2.36 – 2.30 (m, 1H), 2.07 – 2.00 (m, 1H), 1.35 – 1.23 (m, 2H), 1.17 – 1.12 (m, 2H), 0.81 (t, *J* = 7.3 Hz, 3H).

^{13}C NMR (151 MHz, $CDCl_3$) δ 159.5, 149.6, 138.2, 137.5, 133.7, 129.1, 128.9, 117.2, 106.7, 68.6, 66.8, 45.5, 28.9, 26.8, 22.3, 13.8.

HRMS (ESI) calcd. for $C_{20}H_{26}N_2NaO_3S$ ($[M+Na]^+$) *m/z* 397.1556, found 397.1556.

M.P. 145.1 – 146.3 °C.

$[\alpha]_D$ (22.0 °C, c = 0.5 in $CHCl_3$) = +106.8°.

(*R*)-6-(1-(Phenylsulfonyl)pentyl)quinoline (39)

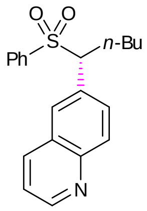

This compound was prepared according to the general procedure with Mn using ((1-chloropentyl)sulfonyl)benzene (37 mg, 0.15 mmol, 1.0 equiv) and 6-iodoquinoline (46.8 mg, 0.225 mmol, 1.5 equiv). The crude mixture was purified by flash column chromatograph to afford **39** as an off-white solid (17.9 mg, 35% yield, 90% ee).

Chiral HPLC (CHIRALCEL OD-H**,** *i*PrOH-hexanes 10/90, 1.0 mL/min, 264 nm, t_R (minor) = 16.4 min, t_R (major) = 22.0 min.

^{1}H NMR (600 MHz, CDCl$_3$) δ 8.97 – 8.89 (m, 1H), 8.05 (d, *J* = 8.3 Hz, 1H), 7.98 (d, *J* = 8.7 Hz, 1H), 7.61 – 7.48 (m, 4H), 7.45 – 7.38 (m, 2H), 7.33 (t, *J* = 8.1 Hz, 2H), 4.22 (dd, *J* = 11.7, 3.5 Hz, 1H), 2.55 – 2.44 (m, 1H), 2.29 – 2.22 (m, 1H), 1.37 – 1.25 (m, 2H), 1.23 – 1.12 (m, *J* = 7.1 Hz, 2H), 0.81 (t, *J* = 7.3 Hz, 3H).

^{13}C NMR (151 MHz, CDCl$_3$) δ 151.3, 148.2, 137.3, 136.3, 133.7, 131.1, 130.5, 129.8, 129.7, 129.1, 128.9, 128.0, 121.70, 71.6, 29.1, 27.4, 22.4, 13.8.

HRMS (ESI) calcd. for $C_{20}H_{21}NNaO_2S$ ($[M+Na]^+$) *m/z* 362.1185, found 362.1190.

M.P. 39.9 – 41.5 °C.

$[\alpha]_D$ (22.0 °C, c = 0.5 in $CHCl_3$) = +94°.

(*R*)-2-Fluoro-5-(1-(phenylsulfonyl)pentyl)pyridine (40)

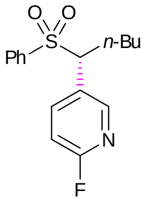

This compound was prepared according to the general procedure with Mn using ((1-chloropentyl)sulfonyl)benzene (37 mg, 0.15 mmol, 1.0 equiv) and 5-bromo-2-fluoropyridine (39.6 mg, 0.225 mmol, 1.5 equiv). The crude mixture was purified by flash column chromatograph to afford **40** as an off-white solid (14.4 mg, 31% yield, 93% ee).

Chiral HPLC (CHIRALCEL OD-H**,** *i*PrOH-hexanes 10/90, 1.0 mL/min, 254 nm, t_R (minor) = 11.3 min, t_R (major) = 13.4 min.

^{1}H NMR (600 MHz, CDCl$_3$) δ 7.78-7.75 (m, 1H), 7.71 (s, 1H)7.62 – 7.53 (m, 3H), 7.44 (t, *J* = 7.7 Hz, 2H), 6.91 (dd, *J* = 8.6, 2.7 Hz, 1H), 4.05 (dd, *J* = 11.9, 3.6 Hz, 1H), 2.47 – 2.37 (m, 1H), 2.10 – 2.04 (m, 1H), 1.35 – 1.24 (m, 2H), 1.19 – 1.11 (m, 2H), 0.83 (t, *J* = 7.4 Hz, 3H).

^{13}C NMR (151 MHz, CDCl$_3$) δ 163.8 (d, *J* = 241.9 Hz), 149.2 (d, *J* = 15.1 Hz), 141.9 (d, *J* = 8.2 Hz), 136.9, 134.2, 129.2, 129.0, 126.6 (d, *J* = 4.7 Hz), 109.9 (d, *J* = 37.5 Hz), 68.1, 28.9, 27.0, 22.3, 13.8.

^{19}F NMR (567 MHz, CDCl$_3$) δ -67.24.

HRMS (ESI) calcd. for $C_{16}H_{18}FNNaO_2S$ ($[M+Na]^+$) *m/z* 330.0935, found 330.0934.

M.P. 75.6 – 76.8 °C.

$[\alpha]_D$ (22.0 °C, c = 0.5 in $CHCl_3$) = +50.8°.

(*R*)-2-Chloro-5-(1-(phenylsulfonyl)pentyl)pyridine (41)

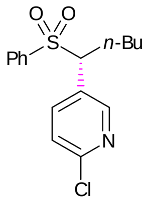

This compound was prepared according to the general procedure with Mn using ((1-chloropentyl)sulfonyl)benzene (37 mg, 0.15 mmol, 1.0 equiv) and 5-bromo-2-chloropyridine (43.3 mg, 0.225 mmol, 1.5 equiv). The crude mixture was purified by flash column chromatograph to afford **41** as an off-white solid (18.6 mg, 38% yield, 90% ee).

Chiral HPLC (CHIRALCEL OD-H**,** *i*PrOH-hexanes 10/90, 1.0 mL/min, 254 nm, t_R (minor) = 12.1 min, t_R (major) = 13.5 min.

^{1}H NMR (600 MHz, $CDCl_3$) δ 7.88 (t, *J* = 2.0 Hz, 1H), 7.64 – 7.54 (m, 4H), 7.45 (td, *J* = 8.0, 1.7 Hz, 2H), 7.30 (dd, *J* = 8.3, 1.6 Hz, 1H), 4.02 (ddd, *J* = 11.8, 3.9, 1.6 Hz, 1H), 2.45 – 2.37 (m, 1H), 2.13 – 2.03 (m, 1H), 1.34 – 1.22 (m, 2H), 1.19 – 1.09 (m, 2H), 0.82 (td, *J* = 7.4, 1.7 Hz, 3H).

^{13}C NMR (151 MHz, $CDCl_3$) δ 152.1, 151.0, 139.4, 136.9 134.2, 129.2, 129.0 , 127.8, 124.5, 68.3, 28.9, 26.9, 22.3, 13.8.

HRMS (ESI) calcd. for $C_{16}H_{18}ClNNaO_2S$ ($[M+Na]^+$) *m/z* 346.0639, found 346.0638.

M.P. 108.9 – 110.5 °C.

$[\alpha]_D$ (22.0 °C, c = 0.5 in $CHCl_3$) = +35.8°.

(*R*)-2-Chloro-5-(1-(phenylsulfonyl)pentyl)-3-(trifluoromethyl)pyridine (42)

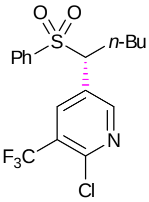

This compound was prepared according to the general procedure with Mn using ((1-chloropentyl)sulfonyl)benzene (37 mg, 0.15 mmol, 1.0 equiv) and 5-bromo-2-chloro-3-(trifluoromethyl)pyridine (58.6 mg, 0.225 mmol, 1.5 equiv). The crude mixture was purified by flash column chromatograph to afford **42** as an off-white solid (25.4 mg, 43% yield, 88% ee).

Chiral HPLC (CHIRALCEL OD-H**,** *i*PrOH-hexanes 10/90, 1.0 mL/min, 230 nm, t_R (minor) = 7.8 min, t_R (major) = 11.9 min.

^{1}H NMR (600 MHz, $CDCl_3$) δ 8.20 (t, *J* = 1.8 Hz, 1H), 7.76 (s, 1H), 7.65 (td, *J* = 7.4, 1.5 Hz, 1H), 7.61 – 7.54 (m, 2H), 7.53 – 7.47 (m, 2H), 4.12 – 4.07 (m, 1H), 2.45 – 2.36 (m, 1H), 2.13 – 2.05 (m, 1H), 1.35 – 1.24 (m, 2H), 1.20 – 1.10 (m, 2H), 0.83 (td, *J* = 7.4, 1.5 Hz, 3H).

^{13}C NMR (151 MHz, $CDCl_3$) δ 153.2, 149.5, 137.40, 136.4, 134.6, 129.4, 129.1, 128.2, 125.4 (q, *J* = 33.5 Hz), 121.9 (q, *J* = 273.1 Hz), 68.1, 28.9, 27.0, 22.2, 13.7.

^{19}F NMR (567 MHz, $CDCl_3$) δ -63.74.

HRMS (ESI) calcd. for $C_{17}H_{17}ClF_3NNaO_2S$ ([M+Na]$^+$) *m/z* 414.0513, found 414.0515.

M.P. 49.5 – 51.2 °C.

$[\alpha]_D$ (22.0 °C, c = 0.5 in $CHCl_3$) = +52.2°.

(*R*)-5-(1-(Phenylsulfonyl)pentyl)-2-(trifluoromethyl)pyridine (43)

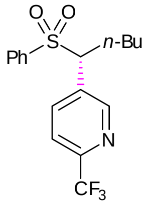

This compound was prepared according to the general procedure with Mn using ((1-chloropentyl)sulfonyl)benzene (37 mg, 0.15 mmol, 1.0 equiv) and 5-bromo-2-(trifluoromethyl)pyridine (50.9 mg, 0.225 mmol, 1.5 equiv). The crude mixture was purified by flash column chromatograph to afford **43** as an off-white solid (35.5 mg, 66% yield, 93% ee).

Chiral HPLC (CHIRALCEL OD-H**,** *i*PrOH-hexanes 10/90, 1.0 mL/min, 264 nm, t_R (minor) = 8.7 min, t_R (major) = 10.6 min.

^{1}H NMR (600 MHz, CDCl$_3$) δ 8.25 (d, *J* = 2.1 Hz, 1H), 7.85 (dd, *J* = 8.1, 2.2 Hz, 1H), 7.66 (d, *J* = 8.1 Hz, 1H), 7.62 (tt, *J* = 7.3, 1.3 Hz, 1H), 7.60 – 7.53 (m, 2H), 7.50 – 7.42 (m, 2H), 4.13 (dd, *J* = 11.6, 3.7 Hz, 1H), 2.47 – 2.42 (m, 1H), 2.17 – 2.13 (m, 1H), 1.36 – 1.23 (m, 2H), 1.22 – 1.09 (m, 2H), 0.83 (t, *J* = 7.4 Hz, 3H).

^{13}C NMR (151 MHz, CDCl$_3$) δ 151.34 , 148.53 (q, *J* = 35.3 Hz), 138.32 , 136.80 , 134.41 , 132.24 , 129.31 , 128.98 , 121.38 (d, *J* = 274.3 Hz), 120.42 (q, *J* = 2.3 Hz) , 68.70 , 28.90 , 27.11 , 22.30 , 13.74 .

^{19}F NMR (567 MHz, CDCl$_3$) δ -68.01.

HRMS (ESI) calcd. for $C_{17}H_{18}F_3NNaO_2S$ ([M+Na]$^+$) *m/z* 380.0903, found 380.0908.

M.P. 65.8 – 67.3 °C.

$[\alpha]_D$ (22.0 °C, c = 0.5 in $CHCl_3$) = +65°.

(*R*)-4-(1-(Phenylsulfonyl)pentyl)-2-(trifluoromethyl)pyridine (44)

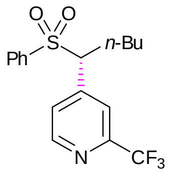

This compound was prepared according to the general procedure with Mn using ((1-chloropentyl)sulfonyl)benzene (37 mg, 0.15 mmol, 1.0 equiv) and 4-bromo-2-(trifluoromethyl)pyridine (50.9 mg, 0.225 mmol, 1.5 equiv). The crude mixture was purified by flash column chromatograph to afford **44** as an off-white solid (27.4 mg, 51% yield, 92% ee).

Chiral HPLC (CHIRALCEL OD-H, *i*PrOH-hexanes 10/90, 1.0 mL/min, 220 nm, t_R (minor) = 7.9 min, t_R (major) = 11.5 min.

^{1}H NMR (600 MHz, Acetone-*d*$_6$) δ 8.71 (d, *J* = 5.0 Hz, 1H), 7.73 (t, *J* = 7.4 Hz, 1H), 7.68 (d, *J* = 7.8 Hz, 2H), 7.65 – 7.60 (m, 2H), 7.57 (t, *J* = 7.7 Hz, 2H), 4.70 (dd, *J* = 11.2, 3.9 Hz, 1H), 2.36 – 2.29 (m, 1H), 2.27 – 2.21 (m, 1H), 1.34 – 1.26 (m, 2H), 1.26 – 1.12 (m, 2H), 0.80 (t, *J* = 7.3 Hz, 3H).

^{13}C NMR (151 MHz, Acetone-*d*$_6$) δ δ 151.2, 148.5 (q, *J* = 34.7 Hz), 145.6, 138.2, 135.0, 130.0, 129.8, 128.7, 122.8 (m), 122.51 (q, *J* = 273.5 Hz), 69.9, 29.4, 27.7, 22.7, 13.9.

^{19}F NMR (567 MHz, Acetone-*d*$_6$) δ -68.57.

HRMS (ESI) calcd. for $C_{17}H_{18}F_3NNaO_2S$ ($[M+Na]^+$) *m/z* 380.0903, found.380.0905.

M.P. 85.6 – 87.1 °C.

$[\alpha]_D$ (22.0 °C, c = 0.5 in $CHCl_3$) = +45.4°.

Methyl (*R*)-4-(4-methyl-1-(phenylsulfonyl)pentyl)benzoate (45)

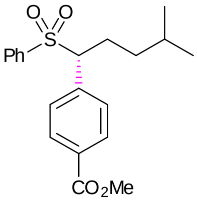

This compound was prepared according to the general procedure with Zn using ((1-chloro-4-methylpentyl)sulfonyl)benzene (39.1 mg, 0.15 mmol, 1.0 equiv) and methyl 4-bromobenzoate (48.5 mg, 0.225 mmol, 1.5 equiv). The crude mixture was purified by flash column chromatograph to afford **45** as an off-white solid (47 mg, 88% yield, 92% ee).

Chiral HPLC (CHIRALCEL OD-H, *i*PrOH-hexanes 10/90, 1.0 mL/min, 240 nm, t_R (minor) = 8.1 min, t_R (major) = 9.4 min.

^{1}H NMR (600 MHz, $CDCl_3$) δ 7.93 – 7.85 (m, 2H), 7.57 – 7.46 (m, 3H), 7.42 – 7.33 (m, 2H), 7.20 – 7.12 (m, 2H), 4.04 (dd, *J* = 11.7, 3.6 Hz, 1H), 3.90 (s, 3H), 2.49 – 2.43 (m, 1H), 2.18 – 2.11 (m, 1H), 1.56 – 1.47 (m, 1H), 1.13 – 1.07 (m, 1H), 1.00 – 0.91 (m, 1H), 0.82 (dd, *J* = 6.6, 4.2 Hz, 6H).

^{13}C NMR (151 MHz, $CDCl_3$) δ 166.7, 137.8, 137.3, 133.8, 130.5, 130.0, 129.7, 129.1, 128.9, 71.8, 52.4, 35.9, 27.9, 25.4, 22.7, 22.1.

HRMS (ESI) calcd for $C_{20}H_{24}NaO_4S$ ($[M+Na]^+$) *m/z* 383.1288, found 383.1289.

M.P. 102.7 – 104.4 °C.

$[\alpha]_D$ (25.0 °C, c = 1.00 in $CHCl_3$) = +84°.

Methyl (*R*)-4-(1-(phenylsulfonyl)propyl)benzoate (46)

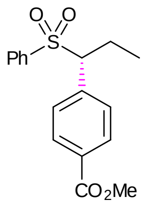

This compound was prepared according to the general procedure with Zn using ((1-chloropropyl)sulfonyl)benzene (32.8 mg, 0.15 mmol, 1.0 equiv) and methyl 4-bromobenzoate (48.4 mg, 0.225 mmol, 1.5 equiv). The crude mixture was purified by flash column chromatograph to afford **46** as an off-white solid (37.9 mg, 73% yield, 90% ee).

Chiral HPLC (CHIRALCEL OD-H**,** *i*PrOH-hexanes 10/90, 1.0 mL/min, 210 nm, t_R (minor) = 13.5 min, t_R (major) = 16.6 min.

^{1}H NMR (600 MHz, $CDCl_3$) δ 7.93 – 7.86 (m, 2H), 7.53 (ddt, *J* = 14.7, 7.5, 1.3 Hz, 3H), 7.41 – 7.33 (m, 2H), 7.21 – 7.14 (m, 2H), 4.01 (dd, *J* = 11.5, 3.7 Hz, 1H), 3.90 (s, 3H), 2.53 – 2.46 (m, 1H), 2.20 – 2.12 (m, 1H), 0.85 (t, *J* = 7.4 Hz, 3H).

^{13}C NMR (151 MHz, $CDCl_3$) δ 166.7, 137.4, 137.3, 133.8, 130.6, 130.0, 129.7, 129.1, 128.9, 73.0, 52.4, 21.2, 11.6.

HRMS (ESI) calcd. for $C_{17}H_{18}NaO_4S$ ($[M+Na]^+$) *m/z* 341.0818, found 341.0825.

M.P. 93.9 – 95.7 °C.

$[\alpha]_D$ (25.0 °C, c = 1.00 in $CHCl_3$) = +97°.

Methyl (*R*)-4-(1-(phenylsulfonyl)ethyl)benzoate (47)

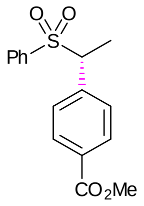

This compound was prepared according to the general procedure with Zn using ((1-chloroethyl)sulfonyl)benzene (30.7 mg, 0.15 mmol, 1.0 equiv) and methyl 4-bromobenzoate (48.4 mg, 0.225 mmol, 1.5 equiv). The crude mixture was purified by flash column chromatograph to afford **47** as an off-white solid (35.6 mg, 78% yield, 91% ee).

Chiral HPLC (CHIRALPAK AD-H**,** *i*PrOH-hexanes 5/95, 0.5 mL/min, 240 nm, t_R (minor) = 43.5 min, t_R (major) = 46.0 min.

^{1}H NMR (600 MHz, $CDCl_3$) δ 7.94 – 7.87 (m, 2H), 7.63 – 7.50 (m, 3H), 7.46 – 7.39 (m, 2H), 7.24 – 7.14 (m, 2H), 4.29 (q, *J* = 7.1 Hz, 1H), 3.90 (s, 3H), 1.78 (d, *J* = 7.1 Hz, 3H).

^{13}C NMR (151 MHz, $CDCl_3$) δ 166.6, 139.0, 136.7, 133.9, 130.6, 129.7, 129.5, 129.3, 128.9, 66.0, 52.4, 14.1.

HRMS (ESI) calcd. for $C_{16}H_{20}NO_4S$ ($[M+NH_4]^+$) *m/z* 322.1108, found 322.1108.

M.P. 107.1 – 108.8 °C.

$[\alpha]_D$ (25.0 °C, c = 0.5 in $CHCl_3$) = +90.8°.

Methyl (*R*)-4-(1-(phenylsulfonyl)but-3-en-1-yl)benzoate (48)

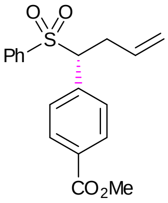

This compound was prepared according to the general procedure with Zn using ((1-chlorobut-3-en-1-yl)sulfonyl)benzene (34.6 mg, 0.15 mmol, 1.0 equiv) and methyl 4-bromobenzoate (48.4 mg, 0.225 mmol, 1.5 equiv). The crude mixture was purified by flash column chromatograph to afford **48** as an off-white solid (20.4 mg, 41% yield, 87% ee).

Chiral HPLC (CHIRALCEL OD-H, *i*PrOH-hexanes 20/80, 1.0 mL/min, 240 nm, t_R (minor) = 7.7 min, t_R (major) = 9.8 min.

^{1}H NMR (600 MHz, $CDCl_3$) δ 7.92 – 7.83 (m, 2H), 7.60 – 7.48 (m, 3H), 7.43 – 7.35 (m, 2H), 7.22 – 7.14 (m, 2H), 5.53 - 5.46 (m, 1H), 4.17 (dd, *J* = 11.5, 3.9 Hz, 1H), 3.90 (s, 3H), 3.26 – 3.17 (m, 1H), 2.94 – 2.89 (m, 1H).

^{13}C NMR (151 MHz, $CDCl_3$) δ 166.7, 137.1, 137.0, 134.0, 132.6, 130.6, 130.1, 129.7, 129.2, 129.0, 119.0, 70.9, 52.4, 32.0.

HRMS (ESI) calcd. for $C_{18}H_{19}O_4S$ ($[M+H]^+$) *m/z* 331.0999, found 331.1011.

M.P. 65.8 – 67.2 °C.

$[\alpha]_D$ (25.0 °C, c = 0.5 in $CHCl_3$) = +86.2°.

Methyl (*R*)-4-(4-chloro-1-(phenylsulfonyl)butyl)benzoate (49)

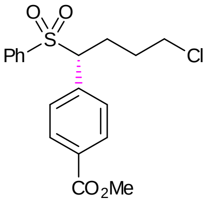

This compound was prepared according to the general procedure with Zn using ((1,4-dichlorobutyl)sulfonyl)benzene (40.1 mg, 0.15 mmol, 1.0 equiv) and methyl 4-bromobenzoate (48.4 mg, 0.225 mmol, 1.5 equiv). The crude mixture was purified by flash column chromatograph to afford **49** as an off-white solid (28.7 mg, 52% yield, 90% ee).

Chiral HPLC (CHIRALCEL OD-H, *i*PrOH-hexanes 10/90, 1.0 mL/min, 240 nm, t_R (minor) = 21.6 min, t_R (major) = 23.3 min.

^{1}H NMR (600 MHz, $CDCl_3$) δ 7.93 – 7.89 (m, 2H), 7.59 – 7.55 (m, 1H), 7.54 – 7.50 (m, 2H), 7.42 – 7.37 (m, 2H), 7.21 – 7.17 (m, 2H), 4.13 (dd, *J* = 11.2, 4.1 Hz, 1H), 3.91 (s, 3H), 3.56 – 3.44 (m, 2H), 2.64 – 2.59 (m, 1H), 2.37 – 2.30 (m, 1H), 1.79 – 1.71 (m, 1H), 1.70 – 1.62 (m, 1H).

^{13}C NMR (151 MHz, $CDCl_3$) δ 166.6, 137.2, 137.0, 134.0, 130.8, 129.9, 129.9, 129.1, 129.0, 70.8, 52.4, 44.0, 29.9, 25.3.

HRMS (ESI) calcd. for $C_{18}H_{20}ClO_4S$ ($[M+H]^+$) *m/z* 367.0765, found 367.0762.

M.P. 89.8 – 91.1 °C.

$[\alpha]_D$ (25.0 °C, c = 0.5 in $CHCl_3$) = +102°.

Methyl (*R*)-4-(4-((*tert*-butyldimethylsilyl)oxy)-1-(phenylsulfonyl)butyl)benzoate (50)

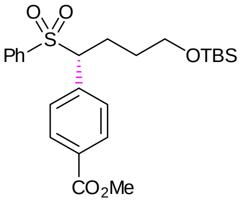

This compound was prepared according to the general procedure with Zn using *tert*-butyl(4-chloro-4-(phenylsulfonyl)butoxy)dimethylsilane (54.5 mg, 0.15 mmol, 1.0 equiv) and methyl 4-bromobenzoate (48.4 mg, 0.225 mmol, 1.5 equiv). The crude mixture was purified by flash column chromatograph to afford **50** as an off-white solid (56.3 mg, 81% yield, 92% ee).

Chiral HPLC (CHIRALCEL OD-H**,** *i*PrOH-hexanes 10/90, 1.0 mL/min, 240 nm, t_R (minor) = 7.5 min, t_R (major) = 8.5 min.

^{1}H NMR (600 MHz, $CDCl_3$) δ 7.94 – 7.85 (m, 2H), 7.57 – 7.51 (m, 3H), 7.41 – 7.36 (m, 2H), 7.21 – 7.16 (m, 2H), 4.17 (dd, *J* = 11.7, 3.8 Hz, 1H), 3.90 (s, 3H), 3.55 (td, *J* = 6.1, 1.8 Hz, 2H), 2.51 – 2.45 (m, 1H), 2.26 – 2.19 (m, 1H), 1.40 – 1.35 (m, 2H), 0.84 (s, 9H), -0.02 (d, *J* = 5.1 Hz, 6H).

^{13}C NMR (151 MHz, $CDCl_3$) δ 166.7, 137.6, 137.3 133.8, 130.6, 130.1, 129.7, 129.1, 128.9, 71.2, 6, 62.3, 52.4, 29.7, 26.0, 24.5, 18.4, -5.3.

HRMS (ESI) calcd. for $C_{24}H_{34}NaO_5SSi$ ($[M+Na]^+$) *m/z* 485.1788, found 485.1788,.

M.P. 110.3 – 112.3 °C.

$[\alpha]_D$ (25.0 °C, c = 0.90 in $CHCl_3$) = +53.111°.

Methyl (*R*)-4-(2-phenyl-1-(phenylsulfonyl)ethyl)benzoate (51)

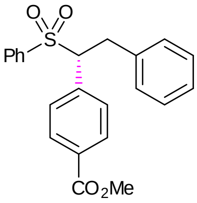

This compound was prepared according to the general procedure with Zn using (2-chloro-2-(phenylsulfonyl)ethyl)benzene (42.1 mg, 0.15 mmol, 1.0 equiv) and methyl 4-bromobenzoate (48.4 mg, 0.225 mmol, 1.5 equiv). The crude mixture was purified by flash column chromatograph to afford **51** as an off-white solid (47.4 mg, 83% yield, 93% ee).

Chiral HPLC (CHIRALCEL OD-H**,** *i*PrOH-hexanes 10/90, 1.0 mL/min, 240 nm, t_R (minor) = 19.0 min, t_R (major) = 37.1 min.

^{1}H NMR (600 MHz, $CDCl_3$) δ 7.87 – 7.78 (m, 2H), 7.61 – 7.51 (m, 3H), 7.38 (t, *J* = 7.8 Hz, 2H), 7.17

(d, J = 7.9 Hz, 2H), 7.14 – 7.05 (m, 3H), 6.98 – 6.89 (m, 2H), 4.34 (dd, J = 11.8, 3.3 Hz, 1H), 3.93 – 3.79 (m, 4H), 3.40 (dd, J = 14.0, 11.8 Hz, 1H).

^{13}C NMR (151 MHz, CDCl$_3$) δ 166.6, 137.2, 137.1, 136.4, 133.9, 130.5, 130.2, 129.6, 129.1, 129.0, 129.0, 128.6, 127.0, 72.9, 52.3, 34.0.

HRMS (ESI) calcd. for $C_{22}H_{20}NaO_4S$ ($[M+Na]^+$) *m/z* 403.0975, found 403.0974.

M.P. 125.5 – 127.2 °C.

$[\alpha]_D$ (25.0 °C, c = 0.55 in $CHCl_3$) = -4.364°.

Methyl (*R*)-4-(3-((*tert*-butyldimethylsilyl)oxy)-1-(phenylsulfonyl)propyl)benzoate (52)

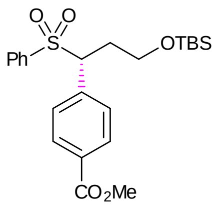

This compound was prepared according to the general procedure with Zn using *tert*-butyl(3-chloro-3-(phenylsulfonyl)propoxy)dimethylsilane (52.3 mg, 0.15 mmol, 1.0 equiv) and methyl 4-bromobenzoate (48.4 mg, 0.225 mmol, 1.5 equiv). The crude mixture was purified by flash column chromatograph to afford **52** as an off-white solid (47.3 mg, 70% yield, 94% ee).

Chiral HPLC (CHIRALCEL OD-H, *i*PrOH-hexanes 10/90, 1.0 mL/min, 240 nm, t_R (minor) = 7.5 min, t_R (major) = 13.6 min.

^{1}H NMR (600 MHz, CDCl$_3$) δ 7.96 – 7.85 (m, 2H), 7.60 – 7.47 (m, 3H), 7.40 (t, J = 7.5 Hz, 2H), 7.20 (d, J = 8.0 Hz, 2H), 4.42 (dd, J = 11.3, 3.6 Hz, 1H), 3.91 (d, J = 1.1 Hz, 3H), 3.69 (dt, J = 9.8, 4.5 Hz, 1H), 3.27 (td, J = 10.2, 4.0 Hz, 1H), 2.67 – 2.62 (m, 1H), 2.28 – 2.20 (m, 1H), 0.80 (d, J = 1.1 Hz, 9H), -0.12 (d, J = 31.1 Hz, 6H).

^{13}C NMR (151 MHz, CDCl$_3$) δ 166.7, 137.4, 137.3, 133.8, 130.5, 130.3, 129.7, 129.1, 129.0, 68.0, 59.1, 52.4, 30.8, 25.9, 18.2, -5.4, -5.5.

HRMS (ESI) calcd. for $C_{23}H_{32}NaO_5SSi$ ($[M+Na]^+$) *m/z* 471.1632, found 471.1635.

M.P. 130.2 – 131.9 °C.

$[\alpha]_D$ (25.0 °C, c = 0.55 in $CHCl_3$) = +44.909°.

Methyl (*R*)-4-(3-hydroxy-1-(phenylsulfonyl)propyl)benzoate (53)

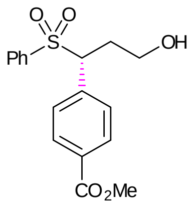

This compound was prepared according to the general procedure with Zn using 3-chloro-3-(phenylsulfonyl)propan-1-ol (35.2 mg, 0.15 mmol, 1.0 equiv) and methyl 4-bromobenzoate (48.4 mg, 0.225 mmol, 1.5 equiv). The crude mixture was purified by flash column chromatograph to afford **53** as an off-white solid (23.2 mg, 46% yield, 90% ee).

Chiral HPLC (CHIRALCEL OD-H**,** *i*PrOH-hexanes 20/80, 1.0 mL/min, 240 nm, t_R (minor) = 12.2 min, t_R (major) = 17.1 min.

^{1}H NMR (600 MHz, $CDCl_3$) δ 7.89 (d, *J* = 8.0 Hz, 2H), 7.59 – 7.49 (m, 3H), 7.43 – 7.34 (m, 2H), 7.21 (d, *J* = 8.0 Hz, 2H), 4.45 (dd, *J* = 10.5, 4.4 Hz, 1H), 3.90 (s, 3H), 3.82 (d, *J* = 10.7 Hz, 1H), 3.43 – 3.38 (m, 1H), 2.76 – 2.70 (m, 1H), 2.36 – 2.31 (m, 1H).

^{13}C NMR (151 MHz, $CDCl_3$) δ 166.6, 137.5, 137.2, 133.9, 130.6, 130.1, 129.8, 129.1, 129.0, 67.9, 59.2, 52.4, 30.9.

HRMS (ESI) calcd. for $C_{17}H_{18}NaO_5S$ ($[M+Na]^+$) *m/z* 357.0767, found 357.0766.

M.P. 140.1 – 141.3 °C.

$[\alpha]_D$ (25.0 °C, c = 0.7 in $CHCl_3$) = +96.286°.

Methyl (*R*)-4-(1-tosylpentyl)benzoate (54)

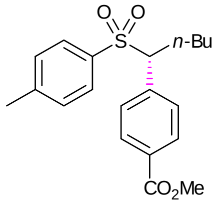

This compound was prepared according to the general procedure with Zn using 1-((1-chloropentyl)sulfonyl)-4-methylbenzene (39.1 mg, 0.15 mmol, 1.0 equiv) and methyl 4-bromobenzoate (48.4 mg, 0.225 mmol, 1.5 equiv). The crude mixture was purified by flash column chromatograph to afford **54** as an off-white solid (35.8 mg, 66% yield, 90% ee).

Chiral HPLC (CHIRALCEL OD-H**,** *i*PrOH-hexanes 10/90, 1.0 mL/min, 240 nm, t_R (minor) = 9.5 min, t_R (major) = 11.9 min.

^{1}H NMR (600 MHz, $CDCl_3$) δ 7.93 – 7.86 (m, 2H), 7.41 – 7.36 (m, 2H), 7.21 – 7.13 (m, 4H), 4.05 (dd, *J* = 11.7, 3.6 Hz, 1H), 3.91 (s, 3H), 2.46 – 2.35 (m, 4H), 2.16 – 2.10 (m, 1H), 1.35 – 1.22 (m, 2H), 1.19 – 1.07 (m, 2H), 0.81 (t, *J* = 7.3 Hz, 3H).

^{13}C NMR (151 MHz, $CDCl_3$) δ 166.7, 144.8, 138.0, 134.4, 130.5, 130.0, 129.70, 129.50, 129.1, 71.5, 52.4, 29.0, 27.3, 22.4, 21.7, 13.8.

HRMS (ESI) calcd. for $C_{20}H_{24}NaO_4S$ ($[M+Na]^+$) *m/z* 383.1288, found 383.1290.

M.P. 87.2 – 88.9 °C.

$[\alpha]_D$ (25.0 °C, c = 0.85 in $CHCl_3$) = +113.059°.

Methyl (*R*)-4-(1-((4-(*tert*-butyl)phenyl)sulfonyl)pentyl)benzoate (55)

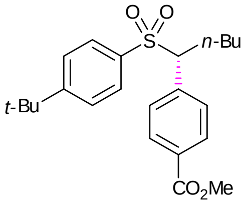

This compound was prepared according to the general procedure with Zn using 1-(*tert*-butyl)-4-((1-chloropentyl)sulfonyl)benzene (45.4 mg, 0.15 mmol, 1.0 equiv) and methyl 4-bromobenzoate (48.4 mg, 0.225 mmol, 1.5 equiv). The crude mixture was purified by flash column chromatograph to afford **55** as an off-white solid (38.1 mg, 63% yield, 90% ee).

Chiral HPLC (CHIRALCEL OD-H**,** *i*PrOH-hexanes 5/95, 1.0 mL/min, 240 nm, t_R (minor) = 13.8 min, t_R (major) = 14.5 min.

^{1}H NMR (600 MHz, $CDCl_3$) δ 7.94 – 7.84 (m, 2H), 7.46 – 7.40 (m, 2H), 7.41 – 7.35 (m, 2H), 7.22 – 7.15 (m, 2H), 4.06 (dd, *J* = 11.6, 3.7 Hz, 1H), 3.91 (s, 3H), 2.44 – 2.38 (m, 1H), 2.17 – 2.11 (m, 1H), 1.31 – 1.23 (m, 11H), 1.18 – 1.09 (m, 2H), 0.81 (t, *J* = 7.3 Hz, 3H).

^{13}C NMR (151 MHz, $CDCl_3$) δ 166.8, 157.8, 138.0, 134.3, 130.5, 130.0, 129.7, 129.0, 125.9, 71.6, 52.4, 35.4, 31.2, 29.0, 27.3, 22.4, 13.9.

HRMS (ESI) calcd for $C_{23}H_{30}NaO_4S$ ($[M+Na]^+$) *m/z* 425.1757, found: 425.1759.

M.P. 99.6 – 101.2 °C.

$[\alpha]_D$ (25.0 °C, c = 0.4 in $CHCl_3$) = +144°.

Methyl (*R*)-4-(1-(mesitylsulfonyl)pentyl)benzoate (56)

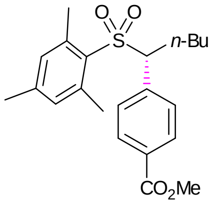

This compound was prepared according to the general procedure with Zn using 2-((1-chloropentyl)sulfonyl)-1,3,5-trimethylbenzene (43.3 mg, 0.15 mmol, 1.0 equiv) and methyl 4-bromobenzoate (48.4 mg, 0.225 mmol, 1.5 equiv). The crude mixture was purified by flash column chromatograph to afford **56** as an off-white solid (29.8 mg, 51% yield, 95% ee).

Chiral HPLC (CHIRALCEL OD-H**,** *i*PrOH-hexanes 10/90, 1.0 mL/min, 240 nm, t_R (minor) = 6.5 min, t_R (major) = 7.4 min.

^{1}H NMR (600 MHz, $CDCl_3$) δ 7.93 – 7.84 (m, 2H), 7.18 (d, *J* = 8.0 Hz, 2H), 6.82 (s, 2H), 4.15 (dd, *J* = 11.4, 3.8 Hz, 1H), 3.90 (s, 3H), 2.64 – 2.15 (m, 11H), 1.36 – 1.24 (m, 2H), 1.21 – 1.14 (m, 2H), 0.82

(t, J = 7.3 Hz, 3H).

^{13}C NMR (151 MHz, CDCl$_3$) δ 166.8, 143.4, 140.6, 137.7, 132.1, 131.7, 130.4, 130.3, 129.6, 70.5, 52.3, 28.9, 26.1, 22.9, 22.4, 21.1, 13.8.

HRMS (ESI) calcd. for $C_{22}H_{28}KO_4S$ ($[M+K]^+$) m/z 427.1340, found 427.1332.

M.P. 101.2 – 102.7 °C.

$[\alpha]_D$ (25.0 °C, c = 1.10 in CHCl$_3$) = + 148.6°.

Methyl (*R*)-4-(1-((4-methoxyphenyl)sulfonyl)pentyl)benzoate (57)

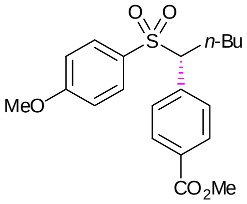

This compound was prepared according to the general procedure with Zn using 1-((1-chloropentyl)sulfonyl)-4-methoxybenzene (41.5 mg, 0.15 mmol, 1.0 equiv) and methyl 4-bromobenzoate (48.4 mg, 0.225 mmol, 1.5 equiv). The crude mixture was purified by flash column chromatograph to afford **57** as a light-yellow oil (40.6 mg, 72% yield, 90% ee).

Chiral HPLC (CHIRALCEL OD-H**,** *i*PrOH-hexanes 10/90, 1.0 mL/min, 254 nm, t_R (minor) = 14.6 min, t_R (major) = 16.7 min.

^{1}H NMR (600 MHz, CDCl$_3$) δ 7.93 – 7.87 (m, 2H), 7.44 – 7.38 (m, 2H), 7.21 – 7.15 (m, 2H), 6.85 – 6.79 (m, 2H), 4.04 (dd, J = 11.7, 3.6 Hz, 1H), 3.90 (s, 3H), 3.82 (s, 3H), 2.43 – 2.40 (m, 1H), 2.14 – 2.10 (m, 1H), 1.34 – 1.22 (m, 2H), 1.19 – 1.06 (m, 2H), 0.81 (t, J = 7.3 Hz, 3H).

^{13}C NMR (151 MHz, CDCl$_3$) δ 166.7, 163.8, 138.1, 131.2, 130.4, 130.0, 129.7, 128.8, 114.0, 71.7, 55.7, 52.3, 29.0, 27.3, 22.4, 13.8.

HRMS (ESI) calcd for $C_{20}H_{24}NaO_5S$ ($[M+Na]^+$) m/z 399.1237, found 399.1239.

$[\alpha]_D$ (25.0 °C, c = 0.8 in CHCl$_3$) = + 127.125°.

Methyl (*R*)-4-(1-((4-fluorophenyl)sulfonyl)pentyl)benzoate (58)

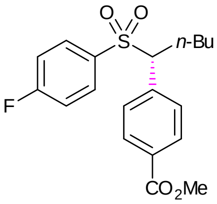

This compound was prepared according to the general procedure with Zn using 1-((1-chloropentyl)sulfonyl)-4-fluorobenzene (39.7 mg, 0.15 mmol, 1.0 equiv) and methyl 4-bromobenzoate (48.4 mg, 0.225 mmol, 1.5 equiv). The crude mixture was purified by flash column chromatograph to afford **58** as an off-white solid (34.6 mg, 63% yield, 90% ee).

Chiral HPLC (CHIRALCEL OD-H**,** *i*PrOH-hexanes 10/90, 1.0 mL/min, 240 nm, t_R (minor) = 8.8 min,

t_R (major) = 15.6 min.

^{1}H NMR (600 MHz, CDCl$_3$) δ 7.95 – 7.88 (m, 2H), 7.54 – 7.47 (m, 2H), 7.21 – 7.15 (m, 2H), 7.09 – 7.01 (m, 2H), 4.06 (dd, *J* = 11.6, 3.7 Hz, 1H), 3.92 (s, 3H), 2.46 – 2.44 (m, 1H), 2.16 – 2.14 (m, 1H), 1.35 – 1.26 (m, 2H), 1.19 – 1.11 (m, 2H), 0.82 (t, *J* = 7.3 Hz, 3H).

^{13}C NMR (151 MHz, CDCl$_3$) δ 166.6, 165.9 (d, *J* = 256.7 Hz), 137.7, 133.4 (d, *J* = 3.0 Hz), 131.9 (d, *J* = 9.5 Hz), 130.7, 130, 129.9, 116.2 (d, *J* = 22.6 Hz), 71.7, 52.4, 29.0, 27.2, 22.4, 13.8.

HRMS (ESI) calcd. for $C_{19}H_{21}FNaO_4S$ ([M+Na]$^+$) *m/z* 387.1037, found 387.1036.

^{19}F NMR (565 MHz, CDCl$_3$) δ -103.20.

M.P. 54.1 – 55.8 °C.

$[\alpha]_D$ (25.0 °C, c = 0.5 in $CHCl_3$) = +68 °.

Methyl (*R*)-4-(1-((4-chlorophenyl)sulfonyl)pentyl)benzoate (59)

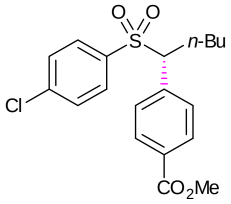

This compound was prepared according to the general procedure with Zn using 1-chloro-4-((1-chloropentyl)sulfonyl)benzene (42.2 mg, 0.15 mmol, 1.0 equiv) and methyl 4-bromobenzoate (48.4 mg, 0.225 mmol, 1.5 equiv). The crude mixture was purified by flash column chromatograph to afford **59** as a yellow solid (28.6 mg, 50% yield, 90% ee).

Chiral HPLC (CHIRALCEL OD-H, *i*PrOH-hexanes 10/90, 1.0 mL/min, 254 nm, t_R (minor) = 8.3 min, t_R (major) = 12.4 min.

^{1}H NMR (600 MHz, CDCl$_3$) δ 7.95 – 7.89 (m, 2H), 7.45 – 7.40 (m, 2H), 7.37 – 7.32 (m, 2H), 7.21 – 7.16 (m, 2H), 4.06 (dd, *J* = 11.6, 3.6 Hz, 1H), 3.92 (s, 3H), 2.47 – 2.41 (m, 1H), 2.18 – 2.11 (m, 1H), 1.36 – 1.22 (m, 2H), 1.22 – 1.08 (m, 2H), 0.82 (t, *J* = 7.3 Hz, 3H).

^{13}C NMR (151 MHz, CDCl$_3$) δ 166.6, 140.6, 137.5, 135.8, 130.6, 130.8, 130.0, 129.9, 129.2, 71.6, 52.4, 29.0, 27.2, 22.4, 13.8.

HRMS (ESI) calcd. for $C_{19}H_{22}ClO_4S$ ([M+H]$^+$) *m/z* 381.0922, found 381.0925.

$[\alpha]_D$ (25.0 °C, c = 1.3 in $CHCl_3$) = +55.545 °.

Methyl (*R*)-4-(1-(thiophen-2-ylsulfonyl)pentyl)benzoate (60)

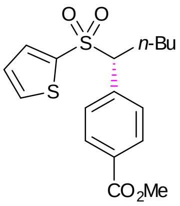

This compound was prepared according to the general procedure with Zn using 2-((1-chloropentyl)sulfonyl)thiophene (37.9 mg, 0.15 mmol, 1.0 equiv) and methyl 4-bromobenzoate (48.4 mg, 0.225 mmol, 1.5 equiv). The crude mixture was purified by flash column chromatograph to afford **60** as an off-white solid (27.6 mg, 52% yield, 88% ee).

Chiral HPLC (CHIRALCEL OD-H**,** *i*PrOH-hexanes 10/90, 1.0 mL/min, 240 nm, t_R (minor) = 12.1 min, t_R (major) = 16.2 min.

^{1}H NMR (600 MHz, $CDCl_3$) δ 7.97 – 7.91 (m, 2H), 7.60 (dd, *J* = 4.9, 1.3 Hz, 1H), 7.26 – 7.24 (m, 3H), 6.99 (dd, *J* = 4.9, 3.8 Hz, 1H), 4.17 (dd, *J* = 11.5, 3.7 Hz, 1H), 3.92 (s, 3H), 2.52 – 2.45 (m, 1H), 2.23 – 2.16 (m, 1H), 1.38 – 1.27 (m, 2H), 1.23 – 1.12 (m, 2H), 0.83 (t, *J* = 7.3 Hz, 3H).

^{13}C NMR (151 MHz, $CDCl_3$) δ 166.7, 138.1, 138.0, 135.2, 134.6, 130.7, 129.9, 129.9, 127.7, 72.8, 52.4, 29.1, 27.5, 22.4, 13.8.

HRMS (ESI) calcd. for $C_{17}H_{21}O_4S_2$ ($[M+H]^+$) *m/z* 353.0876, found 353.0873.

M.P. 81.2 – 83.1 °C.

$[\alpha]_D$ (25.0 °C, c = 0.5 in $CHCl_3$) = +98.6 °.

Methyl (*R*)-4-(1-(morpholinosulfonyl)pentyl)benzoate (61)

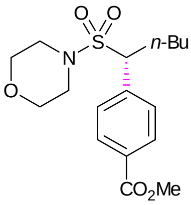

This compound was prepared according to the general procedure with Zn using 4-((1-chloropentyl)sulfonyl)morpholine (38.4 mg, 0.15 mmol, 1.0 equiv) and methyl 4-bromobenzoate (48.4 mg, 0.225 mmol, 1.5 equiv). The crude mixture was purified by flash column chromatograph to afford **61** as an off-white solid (21.4 mg, 40% yield, 89% ee).

Chiral HPLC (CHIRALCEL OD-H**,** *i*PrOH-hexanes 10/90, 1.0 mL/min, 240 nm, t_R (major) = 10.4 min, t_R (minor) = 12.8 min.

^{1}H NMR (600 MHz, $CDCl_3$) δ 8.12 – 8.01 (m, 2H), 7.56 – 7.44 (m, 2H), 4.08 (dd, *J* = 11.4, 3.8 Hz, 1H), 3.93 (s, 3H), 3.56 – 3.52 (m, 2H), 3.48 – 3.44 (m, 2H), 3.07 – 3.03 (m, 2H), 2.78 (s, 2H), 2.37 – 2.32 (m, 1H), 2.19 – 2.09 (m, 1H), 1.38 – 1.22 (m, 2H), 1.21 – 1.04 (m, 2H), 0.83 (t, *J* = 7.3 Hz, 3H).

^{13}C NMR (151 MHz, $CDCl_3$) δ 166.6, 138.7, 130.9, 130.2, 129.8, 68.3, 66.9, 52.5, 46.4, 29.8, 28.8, 22.3, 13.9.

HRMS (ESI) calcd. for $C_{17}H_{25}NNaO_5S$ ($[M+Na]^+$) *m/z* 378.1346, found 378.1348.

M.P. 81.8 – 83.5 °C.

$[\alpha]_D$ (25.0 °C, c = 0.5 in $CHCl_3$) = +34 °.

Methyl (*R*)-4-(1-(methylsulfonyl)-2-phenylethyl)benzoate (62)

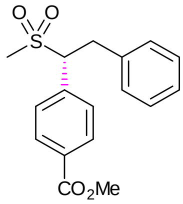

This compound was prepared according to the general procedure with Zn using (2-chloro-2-(methylsulfonyl)ethyl)benzene (32.8 mg, 0.15 mmol, 1.0 equiv) and methyl 4-bromobenzoate (48.4 mg, 0.225 mmol, 1.5 equiv). The crude mixture was purified by flash column chromatograph to afford **62** as an off-white solid (13.8 mg, 29% yield, 81% ee).

Chiral HPLC (CHIRALCEL OD-H**,** *i*PrOH-hexanes 20/80, 1.0 mL/min, 240 nm, t_R (minor) = 18.1min, t_R (major) = 24.5 min.

^{1}H NMR (600 MHz, $CDCl_3$) δ 8.01 (d, *J* = 8.2 Hz, 2H), 7.45 (d, *J* = 8.1 Hz, 2H), 7.22 – 7.09 (m, 3H), 7.05 – 6.91 (m, 2H), 4.30 (dd, *J* = 11.4, 3.6 Hz, 1H), 3.91 (s, 3H), 3.80 (dd, *J* = 13.9, 3.6 Hz, 1H), 3.31 (dd, *J* = 13.9, 11.3 Hz, 1H), 2.64 (s, 3H).

^{13}C NMR (151 MHz, $CDCl_3$) δ 166.5, 137.8, 136.2, 131.1, 130.3, 129.8, 129.1, 128.8, 127.1, 71.7, 52.5, 39.3, 33.8.

HRMS (ESI) calcd. for $C_{17}H_{18}NaO_4S$ ($[M+Na]^+$) *m/z* 341.0818, found 341.0812.

M.P. 86.9 – 88.5 °C.

$[\alpha]_D$ (25.0 °C, c = 0.32 in $CHCl_3$) = -98.125°.

Methyl (*R*)-2-(5-methoxy-2-methyl-1-(4-(1-(phenylsulfonyl)pentyl)benzoyl)-1*H*-indol-3-yl)acetate (63)

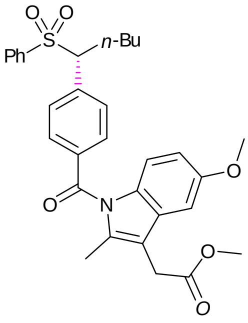

This compound was prepared according to the general procedure with Zn using ((1-chloropentyl)sulfonyl)benzene (37 mg, 0.15 mmol, 1.0 equiv) and methyl 2-(1-(4-bromobenzoyl)-5-methoxy-2-methyl-1H-indol-3-yl)–acetate (93.7 mg, 0.225 mmol, 1.5 equiv). The crude mixture was purified by flash column chromatograph to afford **63** as an off-white solid (42.8 mg, 52% yield, 92% ee).

Chiral HPLC (CHIRALCEL OD-H**,** *i*PrOH-hexanes 20/80, 1.0 mL/min, 210 nm, t_R (minor) = 25.7 min, t_R (major) = 35.5 min.

^{1}H NMR (600 MHz, CDCl$_3$) δ 7.66 – 7.52 (m, 5H), 7.47 – 7.40 (m, 2H), 7.23 (d, *J* = 7.8 Hz, 2H), 6.96 (d, *J* = 2.5 Hz, 1H), 6.83 (d, *J* = 9.0 Hz, 1H), 6.66 (dd, *J* = 9.0, 2.5 Hz, 1H), 4.13 (dd, *J* = 11.6, 3.6 Hz, 1H), 3.85 (s, 3H), 3.71 (s, 3H), 3.67 (s, 2H), 2.52 – 2.47 (m, 1H), 2.32 (s, 3H), 2.21 – 2.15 (m, 1H), 1.39 – 1.27 (m, 2H), 1.22 – 1.15 (m, 2H), 0.85 (t, *J* = 7.3 Hz, 3H).

^{13}C NMR (151 MHz, CDCl$_3$) δ 171.4, 168.9, 156.2, 137.9, 137.4, 136.0, 135.9, 133.9, 130.9, 130.8, 130.3, 129.8, 129.2, 128.9, 115.1, 112.7, 111.7, 101.3, 71.5, 55.9, 52.3, 30.3, 29.0, 27.2, 22.4, 13.9, 13.6.

HRMS (ESI) calcd. for $C_{31}H_{34}NO_6S$ ($[M+H]^+$) *m/z* 548.2101, found 548.2108.

M.P. 84.8 – 86.4 °C.

$[\alpha]_D$ (25.0 °C, c = 0.58 in CHCl$_3$) = +129.828 °.

(*R*)-1-(4-(1-(Phenylsulfonyl)pentyl)benzoyl)pyrrolidin-2-one (64)

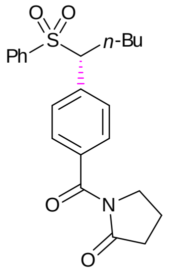

This compound was prepared according to the general procedure with Zn using ((1-chloropentyl)sulfonyl)benzene (37 mg, 0.15 mmol, 1.0 equiv) and 1-(4-bromobenzoyl)pyrrolidin-2-one (60.3 mg, 0.225 mmol, 1.5 equiv). The crude mixture was purified by flash column chromatograph to afford **64** as an off-white solid (29.5 mg, 49% yield, 92% ee).

Chiral HPLC (CHIRALCEL OD-H**,** *i*PrOH-hexanes 20/80, 1.0 mL/min, 254 nm, t_R (minor) = 16.9 min, t_R (major) = 21.0 min.

^{1}H NMR (600 MHz, CDCl$_3$) δ 7.52 - 7.51 (m, 1H), 7.50 – 7.46 (m, 2H), 7.46 – 7.41 (m, 2H), 7.39-7.36 (m, 2H), 7.11 (d, *J* = 8.1 Hz, 2H), 4.05 (dd, *J* = 11.5, 3.7 Hz, 1H), 3.94 (t, *J* = 7.1 Hz, 2H), 2.60 (t, *J* = 8.0 Hz, 2H), 2.53 – 2.44 (m, 1H), 2.21 – 2.09 (m, 3H), 1.37 – 1.17 (m, 7H), 0.84 (t, *J* = 7.3 Hz, 3H).

^{13}C NMR (151 MHz, CDCl$_3$) δ 174.5, 170.19, 137.2, 136.5, 134.9, 133.6, 129.3, 129.23, 129.0, 128.9, 71.7, 46.5, 33.4, 29.0, 26.9, 22.4, 17.8, 13.8.

HRMS (ESI) calcd. for $C_{22}H_{26}NO_4S$ ($[M+H]^+$) *m/z* 400.1577, found 400.1580.

M.P. 140.1 – 141.6 °C.

$[\alpha]_D$ (25.0 °C, c = 0.5 in CHCl$_3$) = + 84.6°.

Isopropyl (*R*)-2-methyl-2-(4-(4-(1-(phenylsulfonyl)pentyl)benzoyl)phenoxy)propanoate (65)

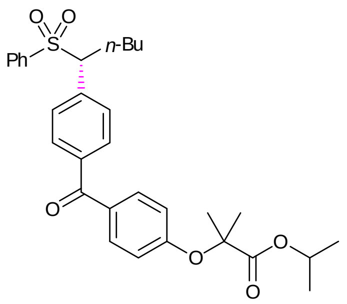

This compound was prepared according to the general procedure with Zn using ((1-chloropentyl)sulfonyl)benzene (37 mg, 0.15 mmol, 1.0 equiv) and isopropyl 2-(4-(4-bromobenzoyl)phenoxy)-2-methylpropanoate (91.2 mg, 0.225 mmol, 1.5 equiv). The crude mixture was purified by flash column chromatograph to afford **65** as an off-white solid (55 mg, 68% yield, 92% ee).

Chiral HPLC (CHIRALCEL OD-H**,** *i*PrOH-hexanes 20/80, 1.0 mL/min, 254 nm, t_R (minor) = 7.3 min, t_R (major) = 9.0 min.

^{1}H NMR (600 MHz, CDCl$_3$) δ 7.74 – 7.68 (m, 2H), 7.60 (d, *J* = 8.1 Hz, 2H), 7.58 – 7.53 (m, 3H), 7.43 – 7.36 (m, 2H), 7.21 (d, *J* = 8.0 Hz, 2H), 6.90 – 6.81 (m, 2H), 5.08 (hept, *J* = 6.3 Hz, 1H), 4.11 (dd, *J* = 11.6, 3.6 Hz, 1H), 2.444 – 2.38 (m, 1H), 2.24 – 2.11 (m, 1H), 1.65 (s, 6H), 1.36 – 1.23 (m, 4H), 1.23 – 1.14 (m, 8H), 0.82 (t, *J* = 7.3 Hz, 3H).

^{13}C NMR (151 MHz, CDCl$_3$) δ 194.9, 173.2, 159.9, 137.4, 136.6, 133.8, 132.1, 130.3, 129.9, 129.9, 129.1, 128.9, 117.3, 79.6, 71.5, 69.5, 29.0, 27.3, 25.5, 25.5, 22.4, 21.7, 13.8.

HRMS (ESI) calcd. for $C_{31}H_{37}O_6S$ ($[M+H]^+$) *m/z* 537.2305, found 537.2304.

M.P. 109.9 – 111.5 °C.

$[\alpha]_D$ (25.0 °C, c = 0.9 in CHCl$_3$) = +87 °.

2-(Diethylamino)ethyl (*R*)-4-(1-(phenylsulfonyl)pentyl)benzoate (66)

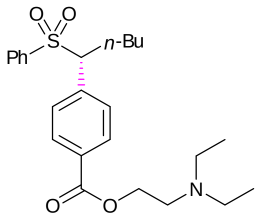

This compound was prepared according to the general procedure with Zn using ((1-chloropentyl)sulfonyl)benzene (37 mg, 0.15 mmol, 1.0 equiv) and 2-(diethylamino)ethyl 4-bromobenzoate (67.5 mg, 0.225 mmol, 1.5 equiv). The crude mixture was purified by flash column chromatograph to afford **66** as a yellow oil (33.8 mg, 52% yield, 91% ee).

Chiral HPLC (CHIRALCEL OD-H**,** *i*PrOH-hexanes 30/70, 1.0 mL/min, 240 nm, t_R (minor) = 4.7 min, t_R (major) = 5.4 min.

^{1}H NMR (600 MHz, CDCl$_3$) δ 7.89 (d, *J* = 8.3 Hz, 2H), 7.57 – 7.47 (m, 3H), 7.42 – 7.32 (m, 2H), 7.17 (d, *J* = 8.3 Hz, 2H), 4.37 (t, *J* = 6.2 Hz, 2H), 4.07 (dd, *J* = 11.6, 3.6 Hz, 1H), 2.83 (t, *J* = 6.2 Hz, 2H), 2.62 (q, *J* = 7.1 Hz, 4H), 2.47 – 2.41 (m, 1H), 2.18 – 2.12 (m, 1H), 1.38 – 1.22 (m, 2H), 1.06 (t, *J* = 7.1 Hz, 6H), 0.82 (t, *J* = 7.3 Hz, 3H).

^{13}C NMR (151 MHz, CDCl$_3$) δ 166.2, 137.8, 137.3, 133.8, 130.7, 130, 129.8, 129.1, 128.9, 71.6, 63.8, 51.2, 48.0, 29.0, 27.2, 22.4, 13.8, 12.2.

HRMS (ESI) calcd. for ([M+H]$^+$) $C_{24}H_{34}NO_4S$ *m/z* 432.2203, found 432.2210.

$[\alpha]_D$ (25.0 °C, c = 0.9 in $CHCl_3$) = + 69°.

Ethyl (*R*)-2-methyl-2-(4-(1-(phenylsulfonyl)pentyl)phenoxy)propanoate (67)

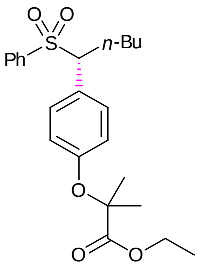

This compound was prepared according to the general procedure with Zn using ((1-chloropentyl)sulfonyl)benzene (37 mg, 0.15 mmol, 1.0 equiv) and ethyl 2-(4-iodophenoxy)-2-methylpropanoate (75.2 mg, 0.225 mmol, 1.5 equiv). The crude mixture was purified by flash column chromatograph to afford **67** as a yellow oil (27 mg, 43% yield, 88% ee).

Chiral HPLC (CHIRALCEL OD-H**,** *i*PrOH-hexanes 20/80, 1.0 mL/min, 254 nm, t_R (minor) = 5.3 min, t_R (major) = 6.3 min.

^{1}H NMR (600 MHz, CDCl$_3$) δ 7.54 – 7.45 (m, 3H), 7.37 – 7.32 (m, 2H), 7.00 – 6.86 (m, 2H), 6.74 – 6.60 (m, 2H), 4.21 (q, *J* = 7.1 Hz, 2H), 3.95 (dd, *J* = 11.6, 3.6 Hz, 1H), 2.42 – 2.37 (m, 1H), 2.17 – 1.97 (m, 1H), 1.56 (d, *J* = 4.8 Hz, 6H), 1.33 – 1.26 (m, 2H), 1.23 (t, *J* = 7.1 Hz, 3H), 1.16 (p, *J* = 7.6 Hz, 2H), 0.82 (t, *J* = 7.3 Hz, 3H).

^{13}C NMR (151 MHz, CDCl$_3$) δ 174.1, 155.9, 137.6, 133.4, 130.8, 129.1, 128.6, 125.9, 119.0, 79.4, 71.1, 61.6, 29.0, 26.9, 25.5, 25.4, 22.4, 14.2, 13.9.

HRMS (ESI) calcd. for $C_{23}H_{30}KO_5S$ ([M+K]$^+$) m/z 457.1446, found 457.1440.

$[\alpha]_D$ (25.0 °C, c = 0.9 in $CHCl_3$) = + 48.278°.

Methyl (*R*)-4-(1-((4-(5-(p-tolyl)-3-(trifluoromethyl)-1*H*-pyrazol-1-yl)phenyl)sulfonyl)pentyl)benzo -ate (68)

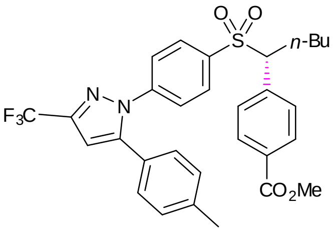

This compound was prepared according to the general procedure with Zn using 1-(4-((1-chloropentyl)sulfonyl)phenyl)-5-(p-tolyl)-3-(tri-fluoromethyl)-1*H*-pyrazole (70.6 mg, 0.15 mmol, 1.0 equiv) and methyl 4-bromobenzoate (48.4 mg, 0.225 mmol, 1.5 equiv). The crude mixture was purified by flash column chromatograph to afford **68** as an off-white solid (53.1 mg, 62% yield, 92% ee).

Chiral HPLC (CHIRALPAK IB-H**,** *i*PrOH-hexanes 10/90, 1.0 mL/min, 240 nm, t_R (minor) = 8.0 min, t_R (major) = 8.6 min.

^{1}H NMR (600 MHz, CDCl$_3$) δ 7.91 (d, *J* = 8.1 Hz, 2H), 7.48 (d, *J* = 8.6 Hz, 2H), 7.34 (d, *J* = 8.7 Hz, 2H), 7.17 (dd, *J* = 21.3, 7.9 Hz, 4H), 7.03 (d, *J* = 8.1 Hz, 2H), 6.71 (s, 1H), 4.09 (dd, *J* = 11.6, 3.6 Hz, 1H), 3.90 (s, 3H), 2.47 – 2.40 (m, 1H), 2.37 (s, 3H), 2.16 – 2.10 (m, 1H), 1.35 – 1.22 (m, 2H), 1.21 – 1.08 (m, 1H), 0.82 (t, *J* = 7.3 Hz, 3H).

^{13}C NMR (151 MHz, CDCl$_3$) δ 166.5, 145.4, 144.3 (q, *J* = 38.6 Hz), 143.4, 140.0, 137.4, 136.6, 130.8, 130.3, 130.1, 130.0, 129.9, 129.8, 128.8, 125.7, 125.1, 121.1 (q, *J* = 269.3 Hz), 106.6 (d, *J* = 2.2 Hz), 71.6, 52.4, 28.9, 27.2, 22.3, 21.4, 13.8.

^{19}F NMR (565 MHz, CDCl$_3$) δ -62.47.

HRMS (ESI) calcd. for $C_{30}H_{29}F_3N_2NaO_4S$ ($[M+Na]^+$) m/z 593.1692, found 593.1699.

M.P. 135.2 – 136.9 °C.

$[\alpha]_D$ (22.0 °C, c = 0.5 in $CHCl_3$) = + 108.4°.

(*R*)-2-Methoxy-5-(1-((4-(5-(p-tolyl)-3-(trifluoromethyl)-1*H*-pyrazol-1-yl)phenyl)sulfonyl)pentyl)pyridine (69)

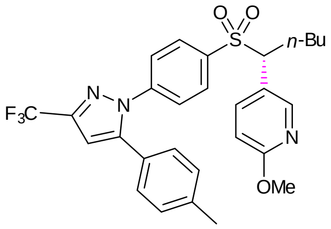

This compound was prepared according to the general procedure with Mn using 1-(4-((1-chloropentyl)sulfonyl)phenyl)-5-(p-tolyl)-3-(trifluoromethyl)-1*H*-pyrazole (70.6 mg, 0.15 mmol, 1.0 equiv) and methyl 5-iodo-2-methoxypyridine (52.9 mg, 0.225 mmol, 1.5 equiv). The crude mixture was purified by flash column chromatograph to afford **69** as an off-white solid (51.3 mg, 63% yield, 87% ee).

Chiral HPLC (CHIRALPAK IB-H**,** *i*PrOH-hexanes 10/90, 1.0 mL/min, 254 nm, t_R (minor) = 7.6 min, t_R (major) = 8.7 min.

^{1}H NMR (600 MHz, CDCl$_3$) δ 7.68 (d, *J* = 2.4 Hz, 1H), 7.55 (d, *J* = 8.4 Hz, 2H), 7.49 (dd, *J* = 8.7, 2.5 Hz, 1H), 7.38 (d, *J* = 8.4 Hz, 2H), 7.19 (d, *J* = 7.8 Hz, 2H), 7.05 (d, *J* = 7.8 Hz, 2H), 6.73 (s, 1H), 6.69 (d, *J* = 8.6 Hz, 1H), 3.98 (dd, *J* = 11.7, 3.6 Hz, 1H), 3.89 (s, 3H), 2.41 – 2.36 (m, 4H), 2.09 – 2.02 (m, 1H), 1.34 – 1.24 (m, 2H), 1.19 – 1.14 (m, 2H), 0.84 (t, *J* = 7.3 Hz, 3H).

^{13}C NMR (151 MHz, CDCl3) δ 164.6, 148.7, 145.5, 144.35 (q, J = 38.6 Hz), 143.4, 140.0, 138.9, 136.7, 130.0, 129.9, 128.8, 125.7, 125.4, 121.1 (q, J = 269.2 Hz), 120.7, 111.3, 106.6, 68.6, 53.7, 28.9, 26.7, 22.3, 21.5, 13.8.

^{19}F NMR (565 MHz, CDCl3) δ -62.48.

M.P. 52.2 – 54.0 °C.

[*α*]*D* (22.0 °C, c = 0.5 in $CHCl_3$) = + 48.8°.

Part 5. Control Experiments and Limitations

Part 5.1 Reaction in presence of radical scavengers

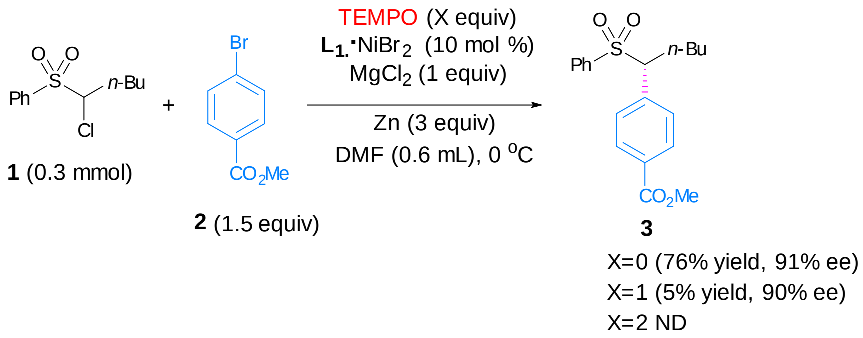

To a flame-dried tube with a stir bar vial was added the appropriate reductant Zn (29.5 mg, 0.45 mmol, 3.0 equiv), methyl 4-bromobenzoate (48.4 mg, 0.225 mmol, 1.5 equiv), $MgCl_2$ (0.15 mmol, 1.0 equiv), **L1**·$NiBr_2$ (0.015mmol, 10 mol %) and TEMPO (if needed, x equiv). The vial was transferred into an N_2-filled glovebox and charged with DMF (0.3 mL). The vial was sealed and removed from the glovebox. The mixture was stirred vigorously, ensuring that the reductant was uniformly suspended. After 5 min the mixture was cooled to 0 °C, then a solution of ((1-chloropentyl)sulfonyl)benzene (37mg, 0.15 mmol, 1.0 equiv) in DMF (0.3 mL) was added to the vial by syringe over 1 min. The mixture was stirred vigorously at 0 °C for 48 h. The reaction mixture was loaded directly onto a silica gel to give the target molecular and detected by ^{1}H NMR.

Part 5.2 Radical clock reaction

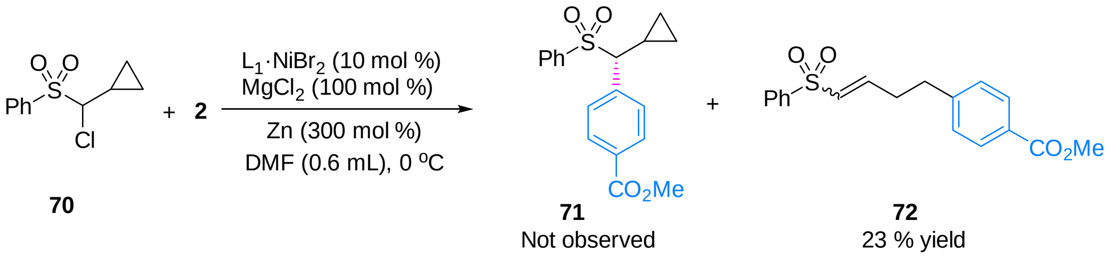

Methyl 4-(4-(phenylsulfonyl)but-3-en-1-yl)benzoate (72)

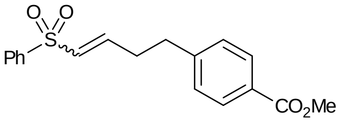

The reaction was proceeded according to the general procedure with Zn using ((chloro(cyclopropyl)methyl)sulfonyl)benzene (34.6 mg, 0.15 mmol, 1.0 equiv) and ethyl 2-(4-iodophenoxy)-2-methyl-propanoate (48.4 mg, 0.225 mmol, 1.5 equiv). The crude mixture was purified by flash column chromatograph to afford **72** as an off-white solid (11.5 mg, 23% yield).

^{1}H NMR (600 MHz, $CDCl_3$) δ 7.95 – 7.87 (m, 2H), 7.83 – 7.75 (m, 2H), 7.64 – 7.58 (m, 1H), 7.51 (t, *J* = 7.8 Hz, 2H), 7.20 – 7.15 (m, 2H), 6.97 (dt, *J* = 15.1, 6.9 Hz, 1H), 6.27 (dt, *J* = 15.1, 1.5 Hz, 1H), 3.91 (s, 3H), 2.84 (t, *J* = 7.6 Hz, 2H), 2.62 – 2.52 (m, 2H).

^{13}C NMR (151 MHz, $CDCl_3$) δ 167.0, 145.4, 145.3, 140.6, 133.5, 131.7, 130.1, 129.4, 128.6, 128.5, 127.7, 52.2, 34.0, 32.8.

HRMS (ESI) calcd. for $C_{18}H_{18}NaO_4S$ ($[M+Na]^+$) *m/z* 353.0818, found 353.0819.

M.P. 90.6 – 92.1 °C.

Part 5.3 Stoichiometric reaction of Ar-Ni(II) (73) with 1

Part 5.3.1 Synthesis of Ar-Ni(L)Br (73)

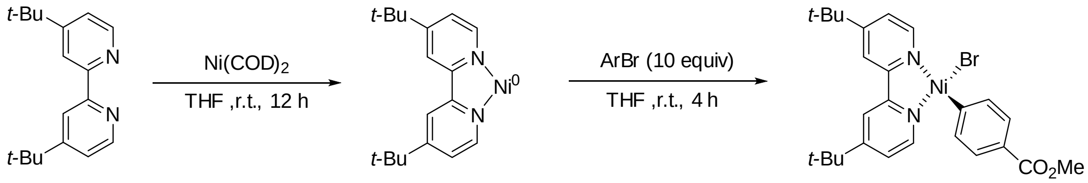

This compound was prepared according to the literature procedure.[7] In a nitrogen filled glove box, a flame-dried round-bottomed flask was charged with $Ni(COD)_2$ (138 mg, 0.5 mmol, 1.0 equiv), dry THF (5 mL) and 4,4'-di-tert-butyl-2,2'-bipyridine (134 mg, 0.5 mmol, 1.0 equiv). After stirring at room temperature overnight, methyl 4-bromobenzoate (1.1 g, 5 mmol, 10 equiv) was added and stirred for additional 4 h. Dry pentane (30 mL) was added slowly to the deep red colored mixture and filtered. The resulting precipitate was washed with pentane (5 x 8 mL) and dried under vacum to afford Ni(II) complex **73** as a brown solid (160 mg, 59% yield). The product was used without further purification. The complex was stored in a nitrogen filled glove box at -35 ºC.

^{1}H NMR (600 MHz, Acetone-d_6) δ 9.23 (s, 1H), 8.44 (d, *J* = 15.1 Hz, 2H), 7.80 – 7.64 (m, 3H), 7.51 (d, *J* = 7.8 Hz, 2H), 7.41 (brs, 1H), 7.10 – 7.03 (m, 1H), 3.82 (s, 3H), 1.40 (m, 18H).

Part 5.3.2 Stoichiometric reaction of Ar-Ni(II) (73) with 1

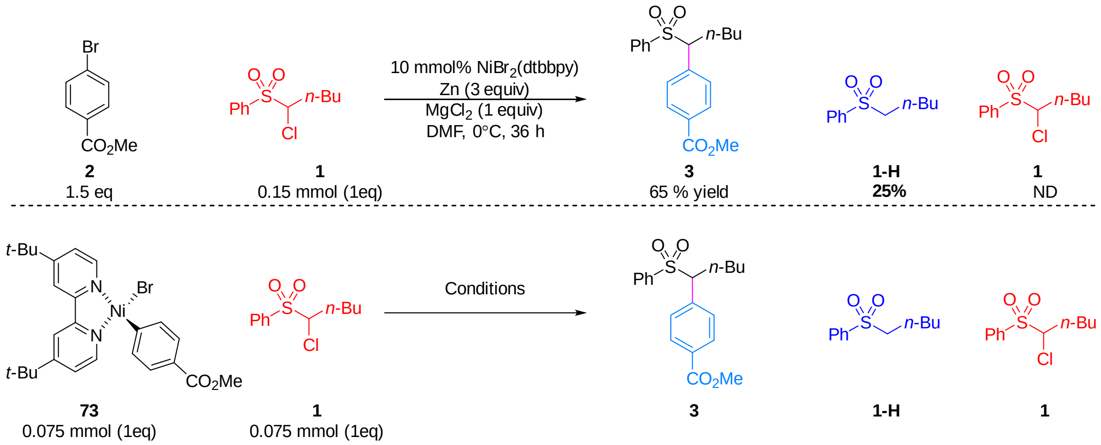

Entry	Zn	$MgCl_2$	Product **3**	Byproduct **1**-H	Residue **1**
1	3 equiv	1 equiv	36%	38%	ND
2	0	1 equiv	36%	ND	37%
3	3 equiv	0	52%	23%	ND
4	0	0	21%	ND	62%

The reaction was conducted in an argon-filled glove box. To a reaction tube equipped with a magnetic stir bar was added Ar-Ni(L)Br (**73**, 40.7 mg, 0.075 mmol), *α*-chlorosulfone **1** (18.5 mg, 0.075 mmol), Zn (if needed, 3 equiv) and $MgCl_2$ (if needed, 1 equiv), followed by DMF (0.3 mL). The reaction tube was sealed and removed from the glove box. The reaction mixture was stirred at 0 °C for 36 h. Then the reaction mixture was diluted with ethyl acetate (5 mL) and water (5 mL). The mixture was extracted with EtOAc (3 x 15 mL), the combined organic phases were washed with water and brine, then dried over $MgSO_4$ and concentrated. The reaction mixtures were analyzed by ^{1}H NMR with an internal standard.

Part 5.4 Limitations

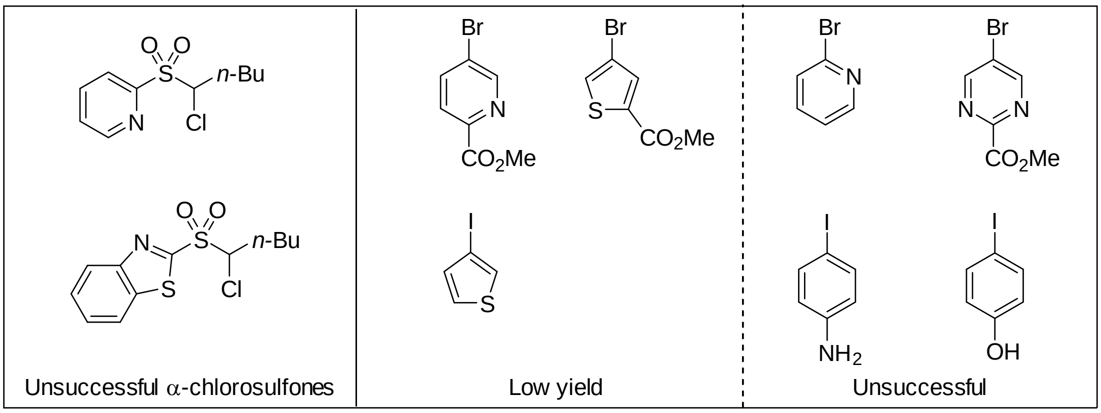

For the substances shown above, no noteworthy result was obtained under the standard reaction conditions.

II. Reference

1. M. Makosza, J. Golinski, and J. Baran, *J. Org. Chem.* **1984**, *49*, 1488.

2. G. Bram, A. Loupy, M. C. Roux-Schmitt, J. Sansoulet, T. Strzalko, and J. Seyden-Penne, *Synthesis*, **1987**, 56.

3. J. Choi, P. Martín-Gago and G. C. Fu, *J. Am. Chem. Soc*. **2014**, *136*, 12161.

4. P. S. Fier, and K. M. Maloney, *J. Am. Chem. Soc*. **2019**, *141*, 1441.

5. J.-M. Mattalia, M. Chanon, and C. J. M. Stirling, *J. Org. Chem.***1996**, *61*, 1153.

6. N. Suzuki, J. L. Hofstra, K. E. Poremba, and S. E. Reisman, *Org. Lett*. **2017**, *19*, 2150.

7. L. Guo, F. Song, S. Zhu, H. Li and L Chu, Nature *Nat. Commun.* **2018**, *9*, 4543.

III. HPLC Traces

3: racemic

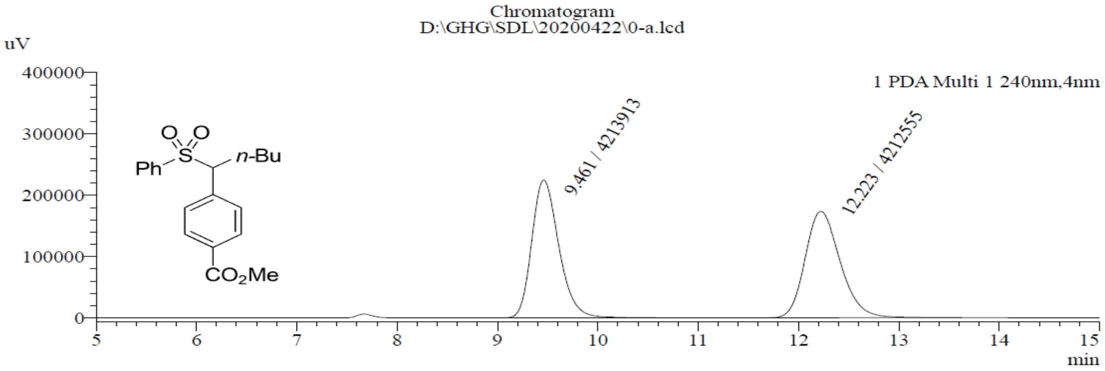

Peak Table

PDA Ch1 240nm

Peak#	Ret. Time [min]	Width [min]	Area [uV*s]	Height [uV]	Area %
1	9.461	0.2851	4213912.77	224904.05	50.0081
2	12.223	0.3714	4212554.96	173423.32	49.9919

3: enantioenriched, 91% ee

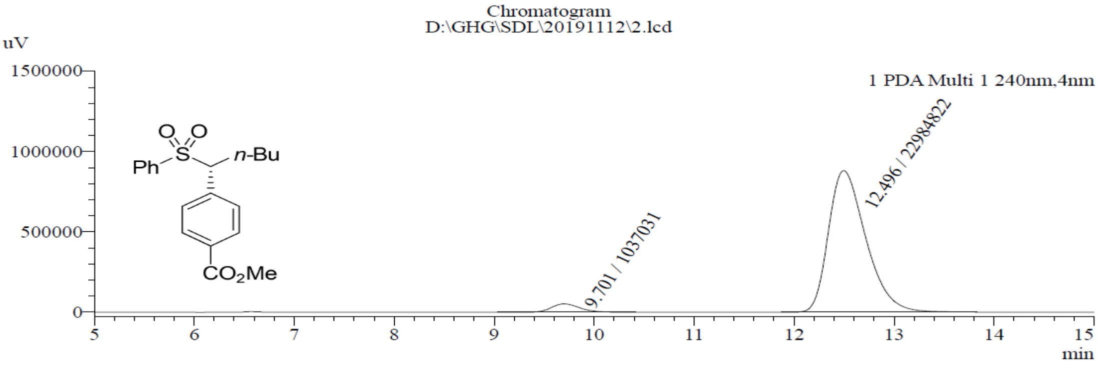

Peak Table

PDA Ch1 240nm

Peak#	Ret. Time [min]	Width [min]	Area [uV*s]	Height [uV]	Area %
1	9.701	0.3008	1037031.16	52278.25	4.3170
2	12.496	0.3981	22984822.47	881338.81	95.6830

3 *S*: enantioenriched, 92% ee

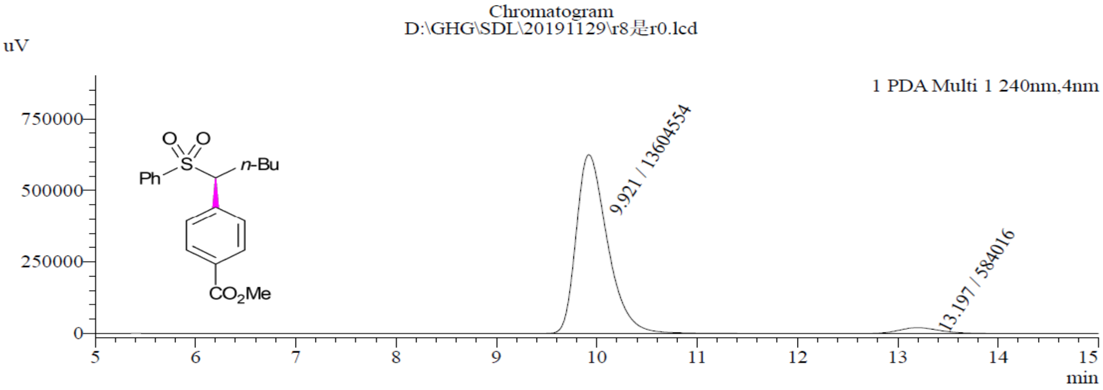

Peak Table

PDA Ch1 240nm

Peak#	Ret. Time [min]	Width [min]	Area [uV*s]	Height [uV]	Area %
1	9.921	0.3272	13604553.71	626504.51	95.8839
2	13.197	0.4341	584015.50	20435.30	4.1161

4: racemic

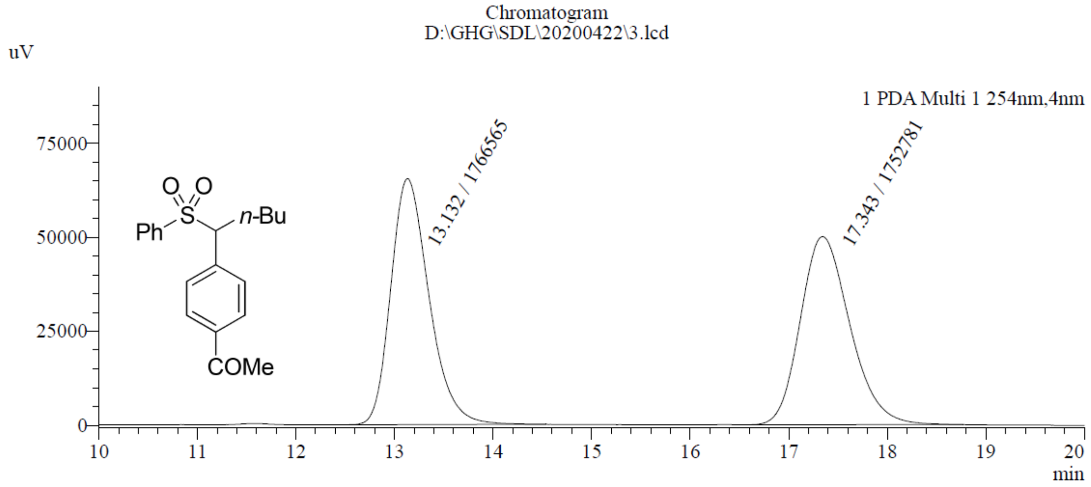

Peak Table

PDA Ch1 254nm

Peak#	Ret. Time [min]	Width [min]	Area [uV*s]	Height [uV]	Area %
1	13.132	0.4096	1766564.74	65458.87	50.1958
2	17.343	0.5369	1752781.36	50060.67	49.8042

4: enantioenriched, 91% ee

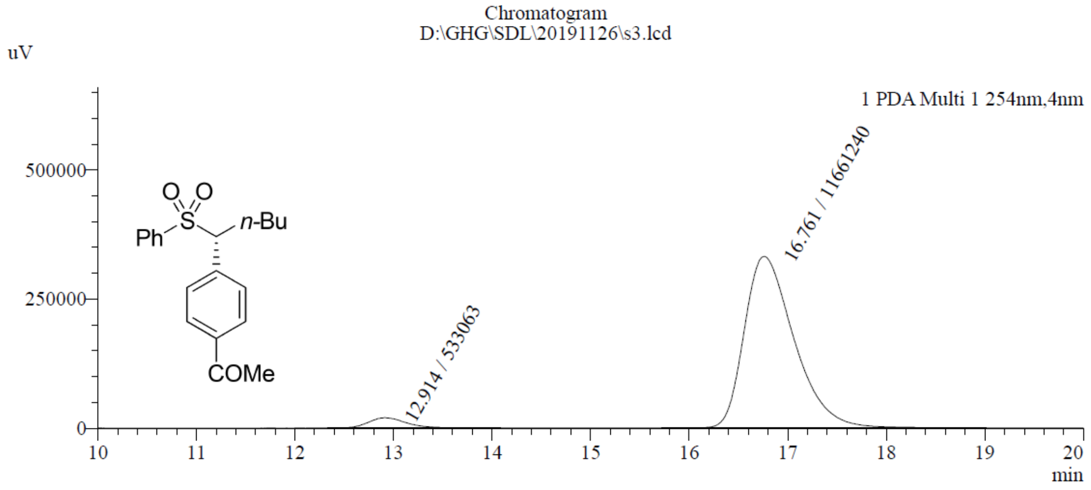

Peak Table

PDA Ch1 254nm

Peak#	Ret. Time [min]	Width [min]	Area [uV*s]	Height [uV]	Area %
1	12.914	0.4051	533063.07	19867.41	4.3714
2	16.761	0.5341	11661239.78	332091.75	95.6286

5: racemic

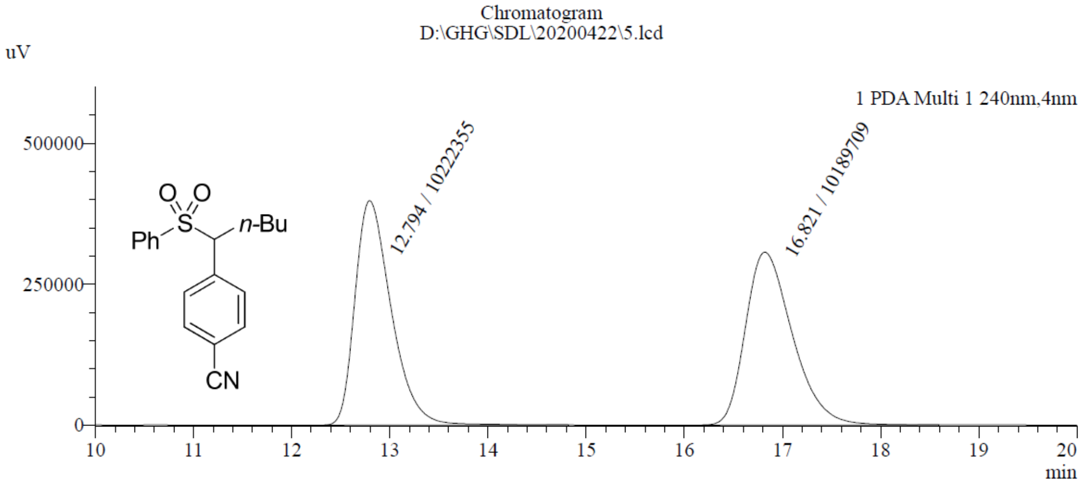

Peak Table

PDA Ch1 240nm

Peak#	Ret. Time [min]	Width [min]	Area [uV*s]	Height [uV]	Area %
1	12.794	0.3895	10222354.51	397908.39	50.0800
2	16.821	0.5088	10189709.11	306472.60	49.9200

5: enantioenriched, 89% ee

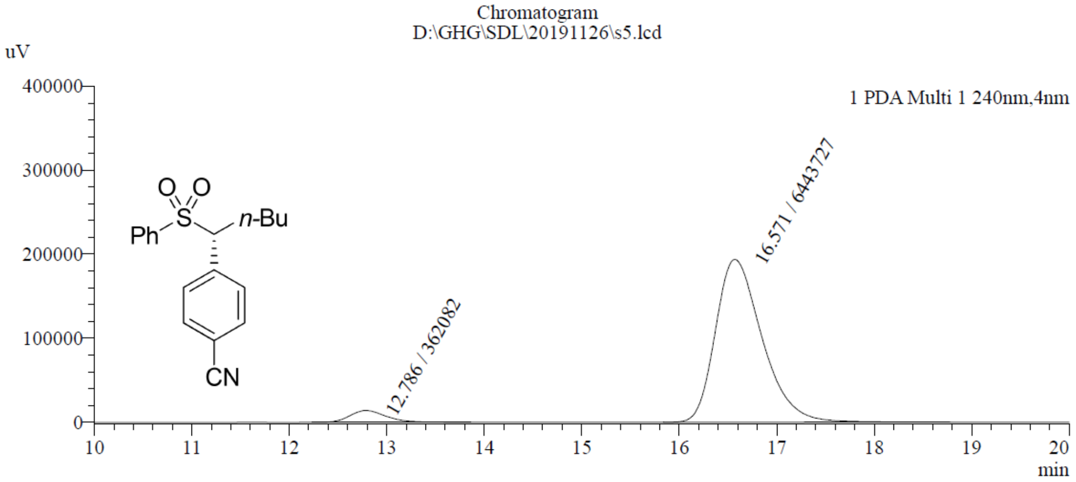

Peak Table

PDA Ch1 240nm

Peak#	Ret. Time [min]	Width [min]	Area [uV*s]	Height [uV]	Area %
1	12.786	0.3917	362081.73	14020.26	5.3202
2	16.571	0.5057	6443726.75	193691.55	94.6798

6: racemic

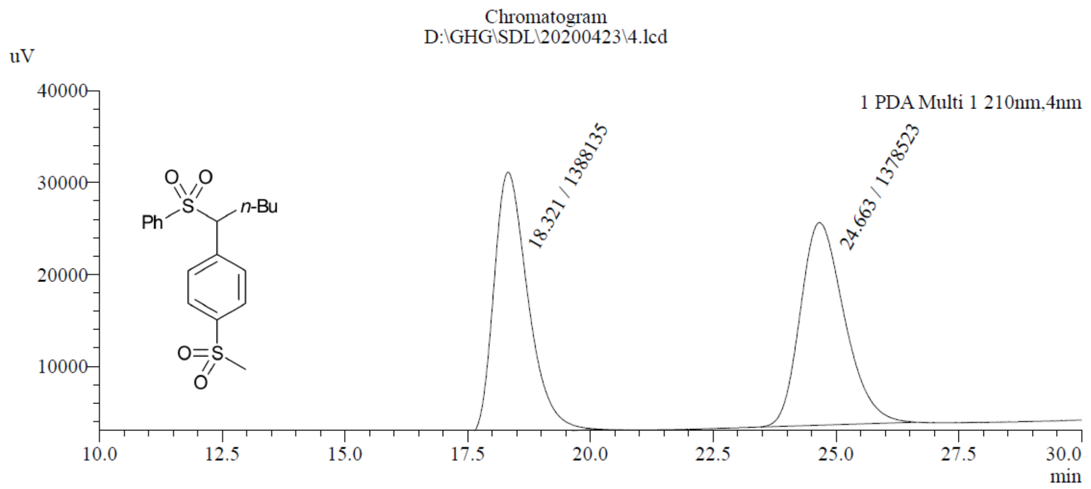

Peak Table

PDA Ch1 210nm

Peak#	Ret. Time [min]	Width [min]	Area [uV*s]	Height [uV]	Area %
1	18.321	0.7361	1388135.21	28565.29	50.1737
2	24.663	0.9627	1378523.48	22032.70	49.8263

6: enantioenriched, 88% ee

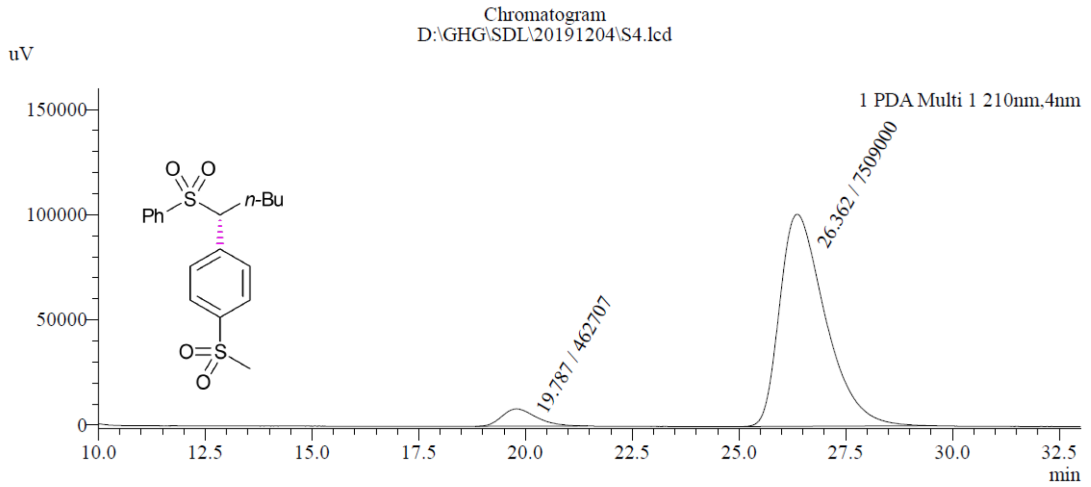

Peak Table

PDA Ch1 210nm

Peak#	Ret. Time [min]	Width [min]	Area [uV*s]	Height [uV]	Area %
1	19.787	0.8642	462707.41	8206.38	5.8044
2	26.362	1.1304	7508999.99	100925.66	94.1956

7: racemic

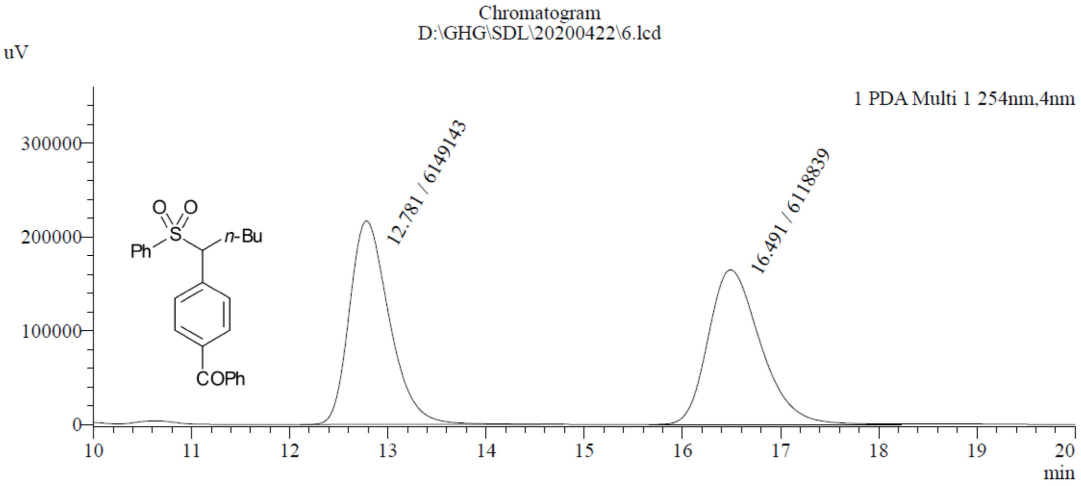

Peak Table

PDA Ch1 254nm

Peak#	Ret. Time [min]	Width [min]	Area [uV*s]	Height [uV]	Area %
1	12.781	0.4296	6149143.43	217120.37	50.1235
2	16.491	0.5652	6118839.24	164987.28	49.8765

7: enantioenriched, 89% ee

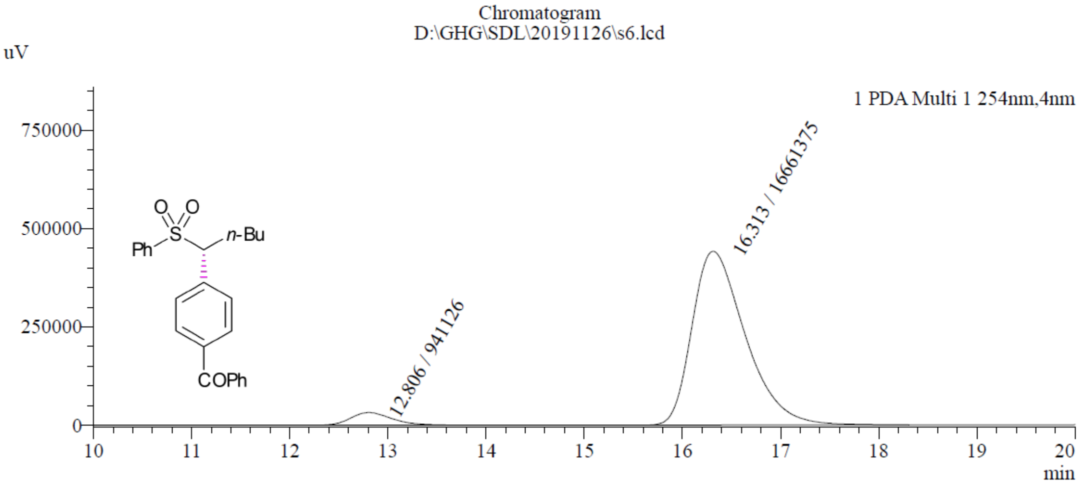

Peak Table

PDA Ch1 254nm

Peak#	Ret. Time [min]	Width [min]	Area [uV*s]	Height [uV]	Area %
1	12.806	0.4416	941126.10	32279.65	5.3465
2	16.313	0.5737	16661374.60	441888.06	94.6535

8: racemic

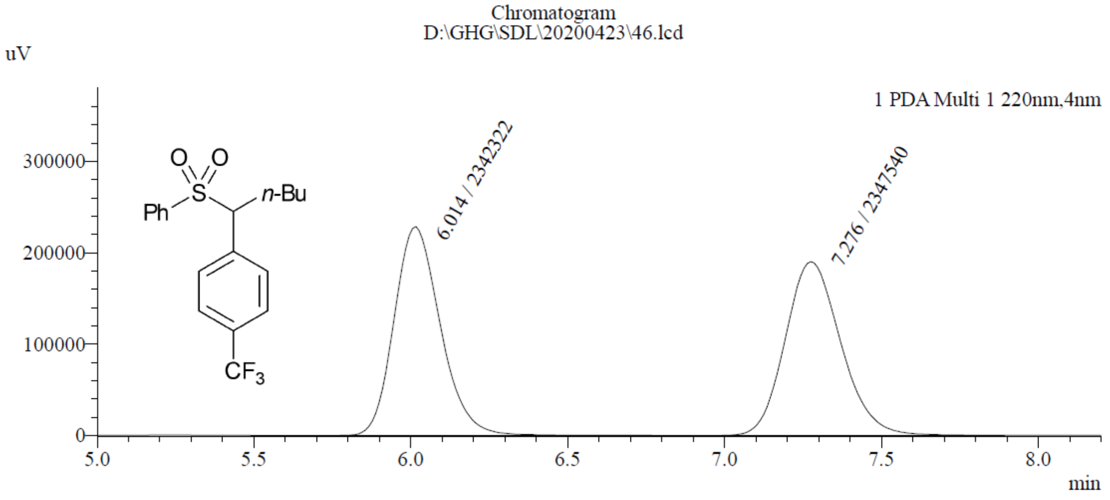

Peak Table

PDA Ch1 220nm

Peak#	Ret. Time [min]	Width [min]	Area [uV*s]	Height [uV]	Area %
1	6.014	0.1564	2342322.35	228418.54	49.9444
2	7.276	0.1892	2347539.89	190229.17	50.0556

8: enantioenriched, 91% ee

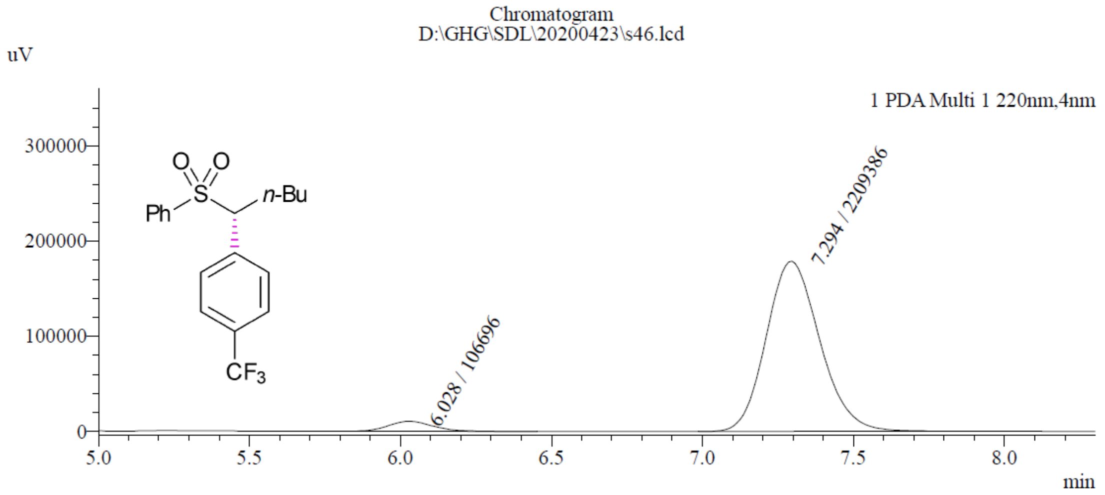

Peak Table

PDA Ch1 220nm

Peak#	Ret. Time [min]	Width [min]	Area [uV*s]	Height [uV]	Area %
1	6.028	0.1558	106696.19	10505.17	4.6068
2	7.294	0.1897	2209386.22	178942.08	95.3932

9: racemic

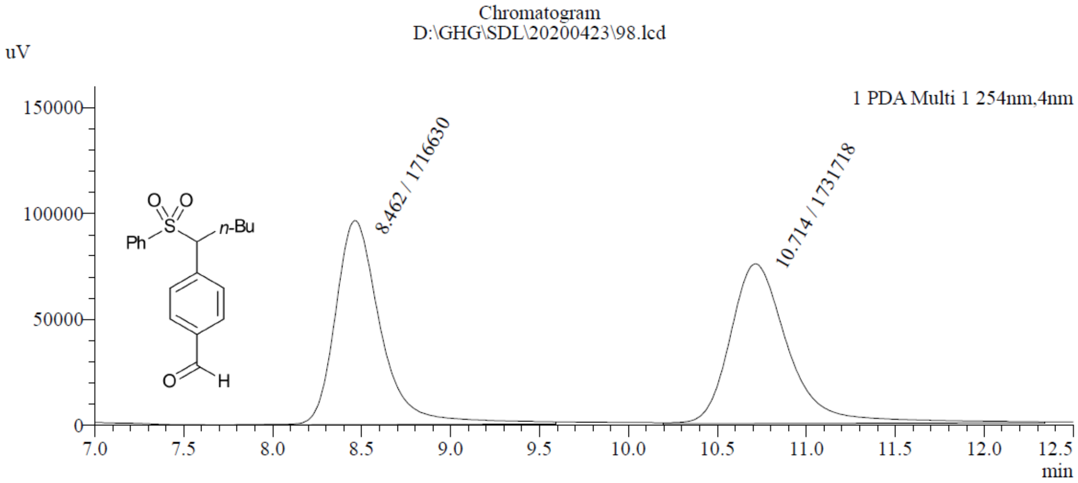

Peak Table

PDA Ch1 254nm

Peak#	Ret. Time [min]	Width [min]	Area [uV*s]	Height [uV]	Area %
1	8.462	0.2546	1716629.87	96384.32	49.7812
2	10.714	0.3281	1731717.78	75427.95	50.2188

9: enantioenriched, 92% ee

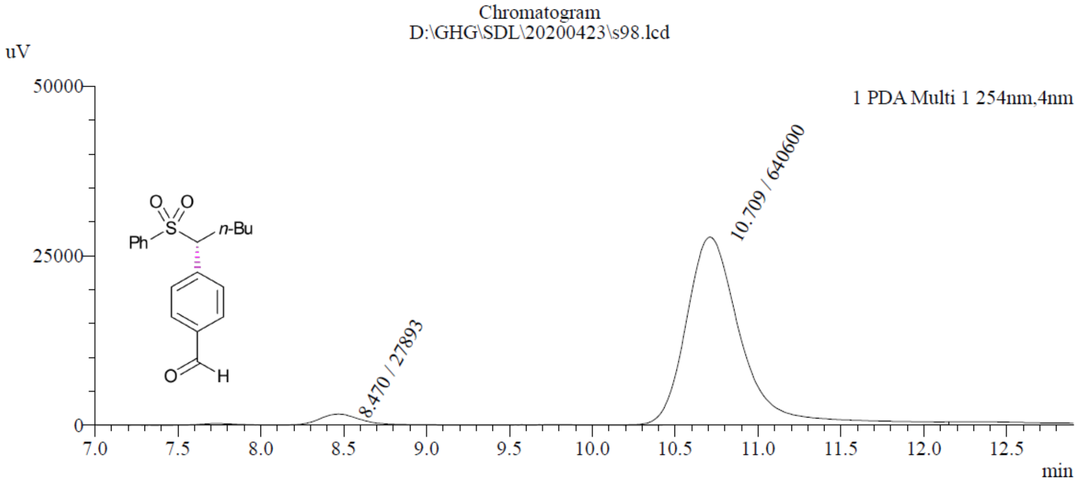

Peak Table

PDA Ch1 254nm

Peak#	Ret. Time [min]	Width [min]	Area [uV*s]	Height [uV]	Area %
1	8.470	0.2557	27892.68	1623.71	4.1725
2	10.709	0.3284	640599.51	27706.72	95.8275

10: racemic

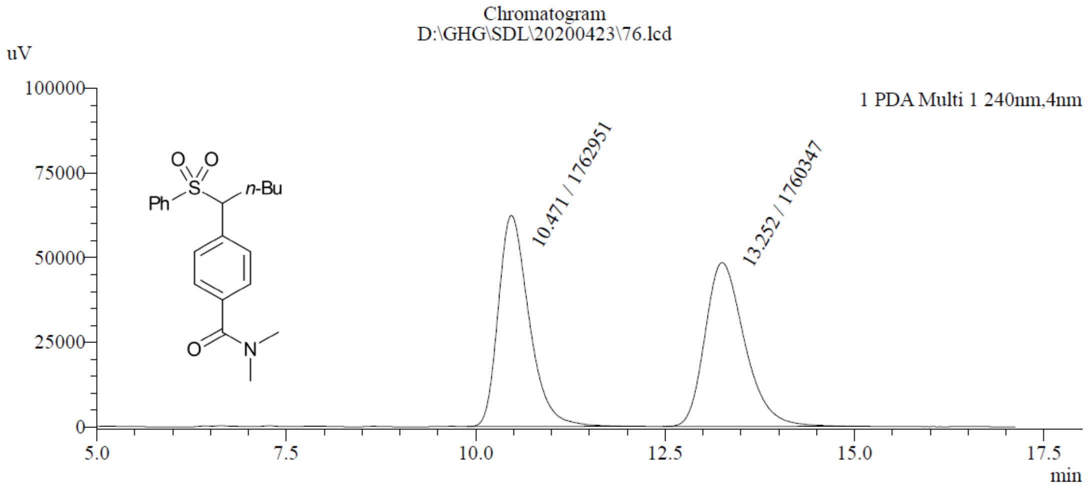

Peak Table

PDA Ch1 240nm

Peak#	Ret. Time [min]	Width [min]	Area [uV*s]	Height [uV]	Area %
1	10.471	0.4256	1762950.55	62303.61	50.0370
2	13.252	0.5521	1760346.72	48340.66	49.9630

10: enantioenriched, 89% ee

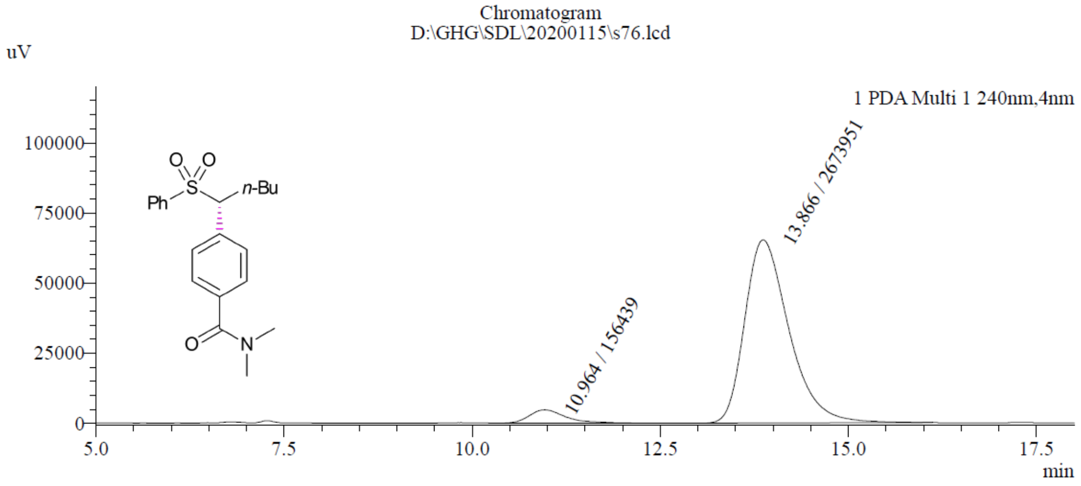

Peak Table

PDA Ch1 240nm

Peak#	Ret. Time [min]	Width [min]	Area [uV*s]	Height [uV]	Area %
1	10.964	0.4910	156439.24	4721.02	5.5271
2	13.866	0.6162	2673950.71	65266.25	94.4729

11: racemic

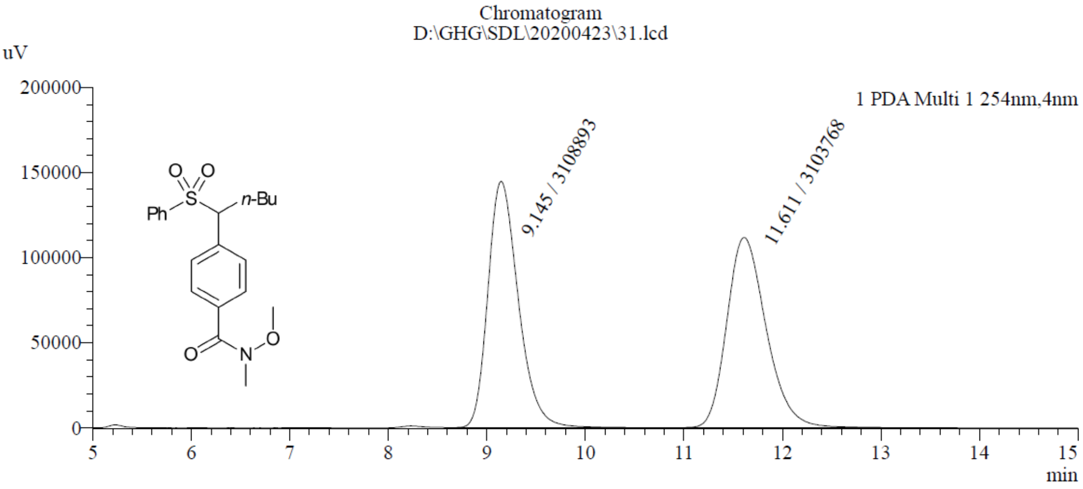

Peak Table

PDA Ch1 254nm

Peak#	Ret. Time [min]	Width [min]	Area [uV*s]	Height [uV]	Area %
1	9.145	0.3261	3108892.73	144732.59	50.0412
2	11.611	0.4244	3103767.78	111630.50	49.9588

11: enantioenriched, 90% ee

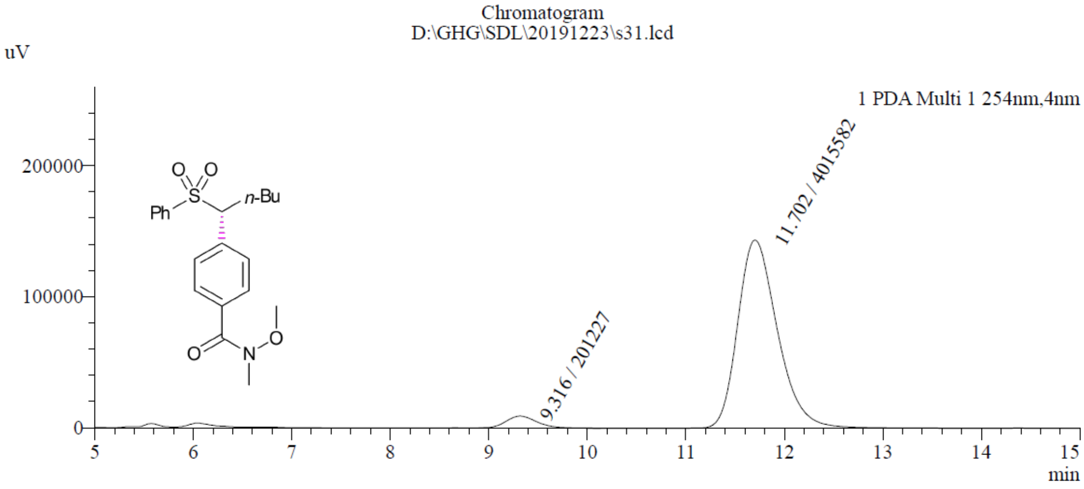

Peak Table

PDA Ch1 254nm

Peak#	Ret. Time [min]	Width [min]	Area [uV*s]	Height [uV]	Area %
1	9.316	0.3318	201226.97	9202.08	4.7720
2	11.702	0.4251	4015581.62	143635.80	95.2280

12: racemic

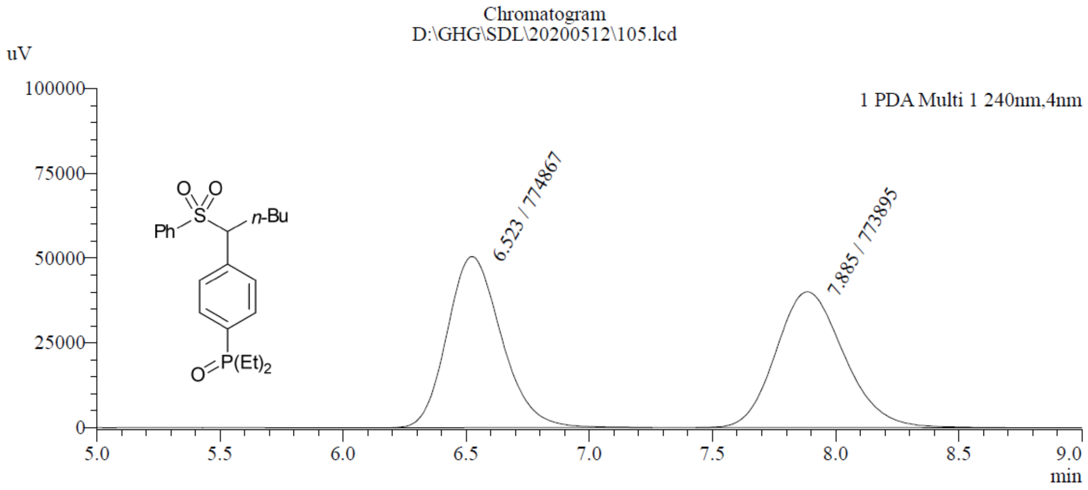

Peak Table

PDA Ch1 240nm

Peak#	Ret. Time [min]	Width [min]	Area [uV*s]	Height [uV]	Area %
1	6.523	0.2354	774866.82	50416.09	50.0314
2	7.885	0.2972	773894.71	40051.69	49.9686

12: enantioenriched, 91% ee

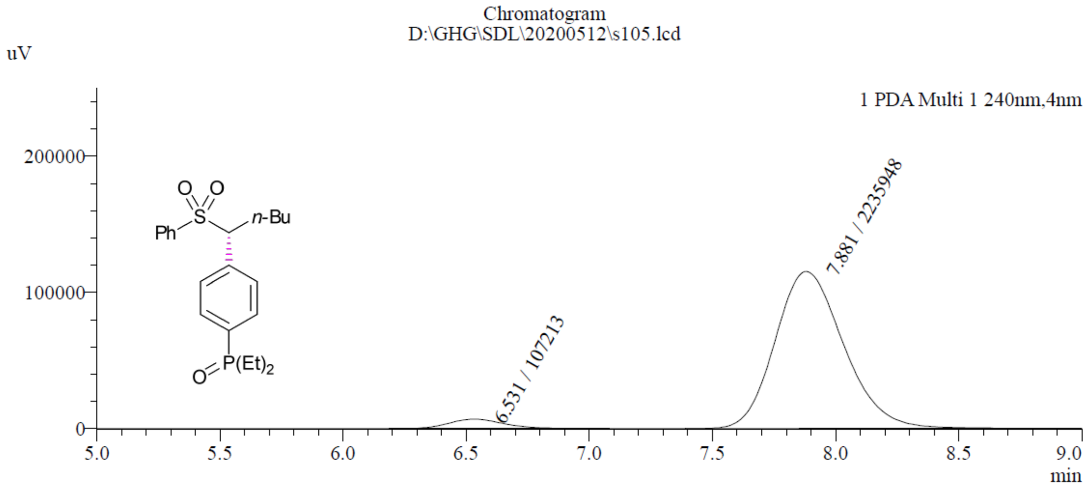

Peak Table

PDA Ch1 240nm

Peak#	Ret. Time [min]	Width [min]	Area [uV*s]	Height [uV]	Area %
1	6.531	0.2377	107212.59	6905.84	4.5756
2	7.881	0.2982	2235948.12	115291.33	95.4244

13: racemic

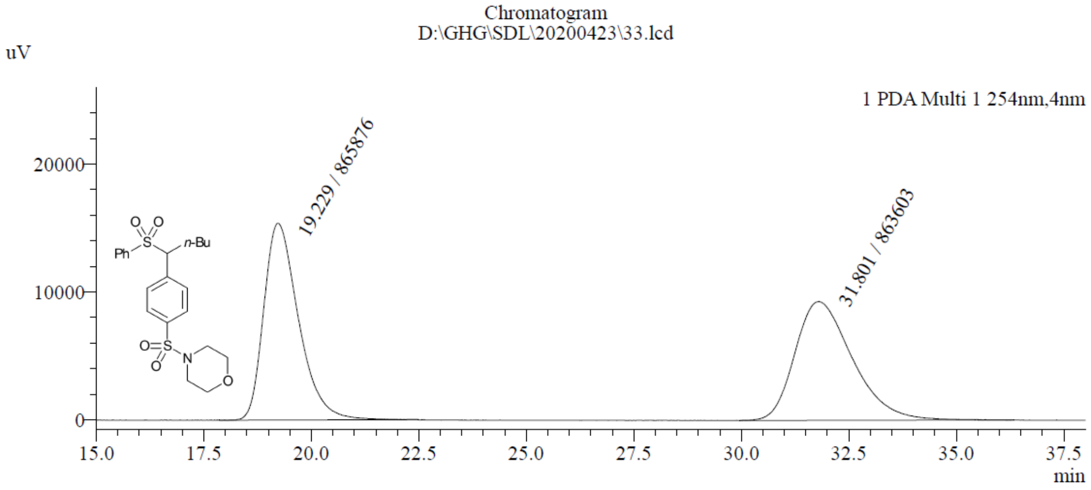

Peak Table

PDA Ch1 254nm

Peak#	Ret. Time [min]	Width [min]	Area [uV*s]	Height [uV]	Area %
1	19.229	0.8491	865875.59	15395.21	50.0657
2	31.801	1.4178	863602.75	9288.66	49.9343

13: enantioenriched, 89% ee

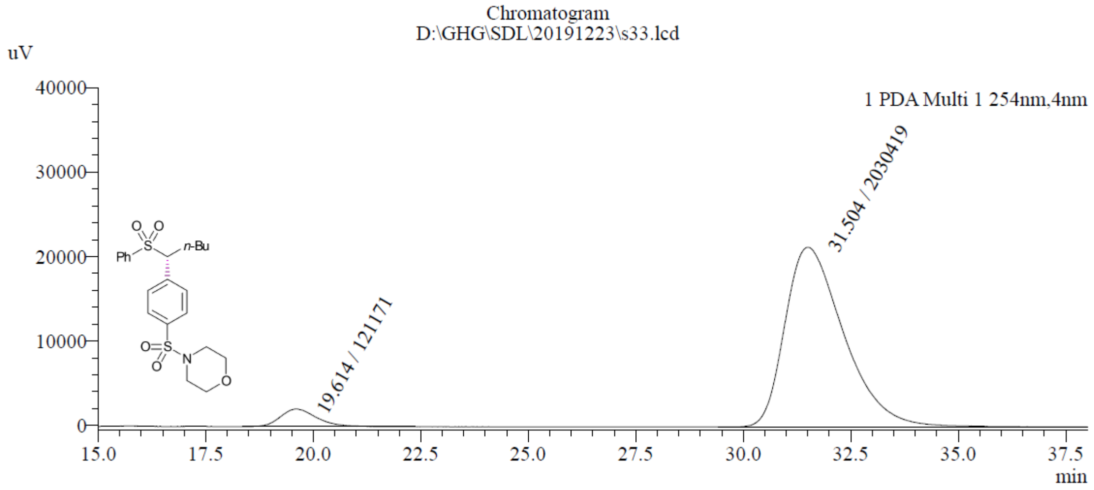

Peak Table

PDA Ch1 254nm

Peak#	Ret. Time [min]	Width [min]	Area [uV*s]	Height [uV]	Area %
1	19.614	0.8882	121171.12	2072.81	5.6317
2	31.504	1.4514	2030419.01	21276.57	94.3683

14: racemic

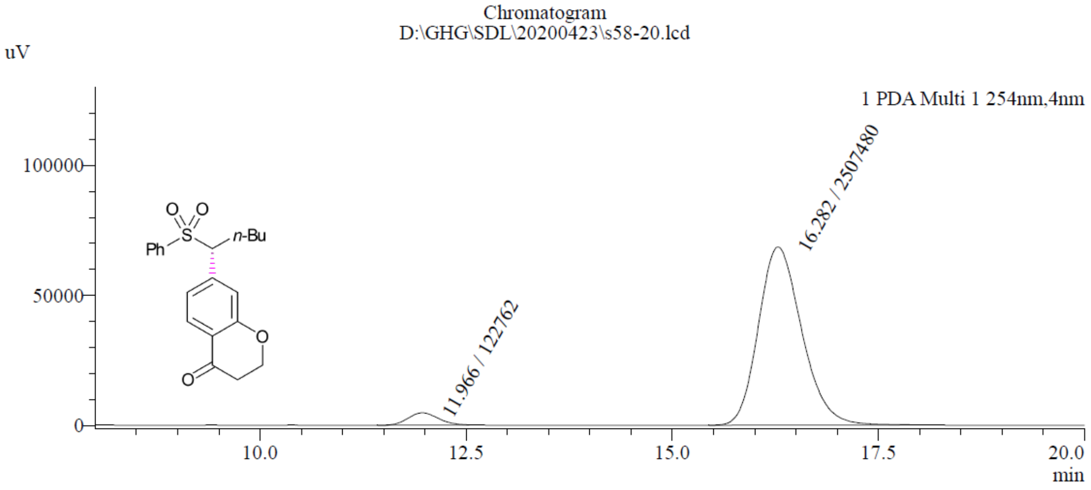

Peak Table

PDA Ch1 254nm

Peak#	Ret. Time [min]	Width [min]	Area [uV*s]	Height [uV]	Area %
1	11.966	0.4018	122761.68	4742.24	4.6673
2	16.282	0.5608	2507480.50	68489.89	95.3327

14: enantioenriched, 91% ee

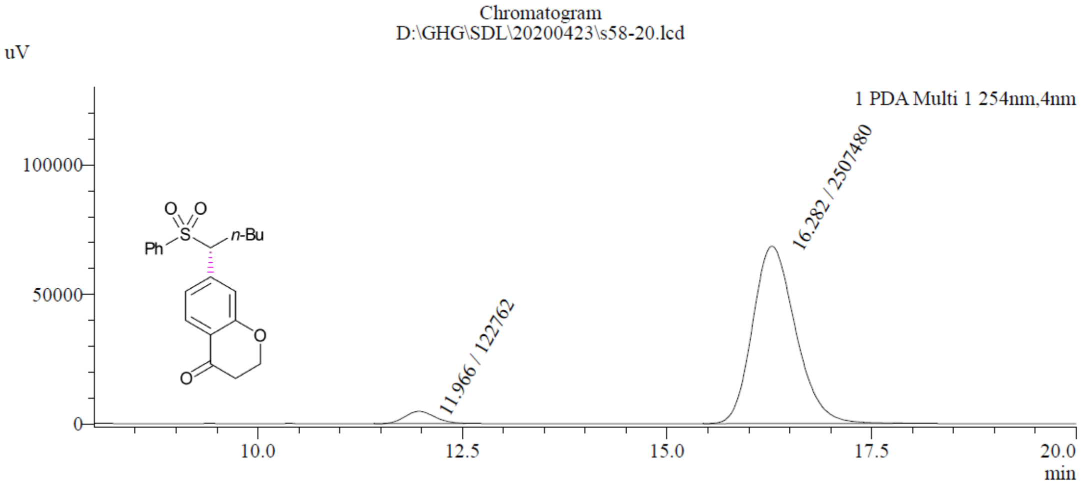

Peak Table

PDA Ch1 254nm

Peak#	Ret. Time [min]	Width [min]	Area [uV*s]	Height [uV]	Area %
1	11.966	0.4018	122761.68	4742.24	4.6673
2	16.282	0.5608	2507480.50	68489.89	95.3327

15: racemic

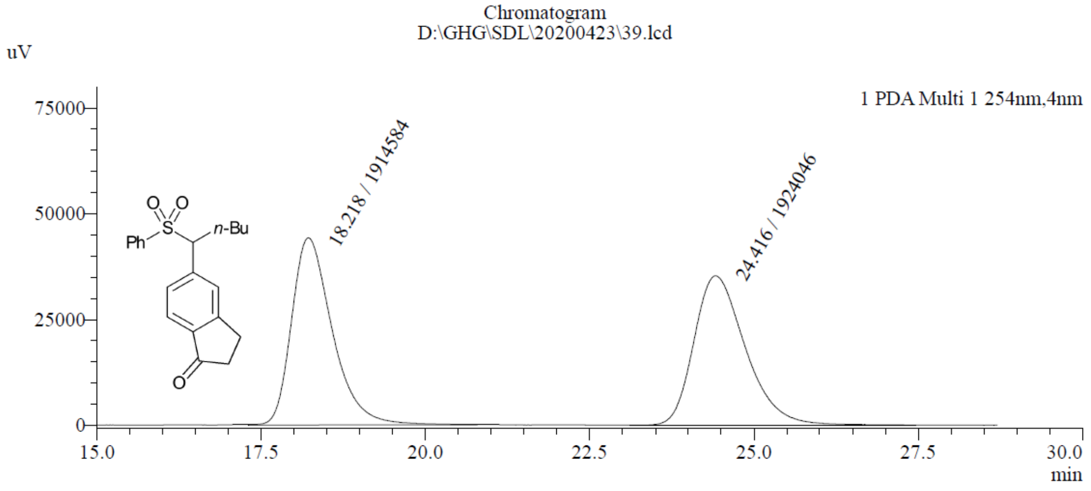

Peak Table

PDA Ch1 254nm

Peak#	Ret. Time [min]	Width [min]	Area [uV*s]	Height [uV]	Area %
1	18.218	0.6451	1914584.07	44248.12	49.8768
2	24.416	0.8288	1924045.93	35294.15	50.1232

15: enantioenriched, 89% ee

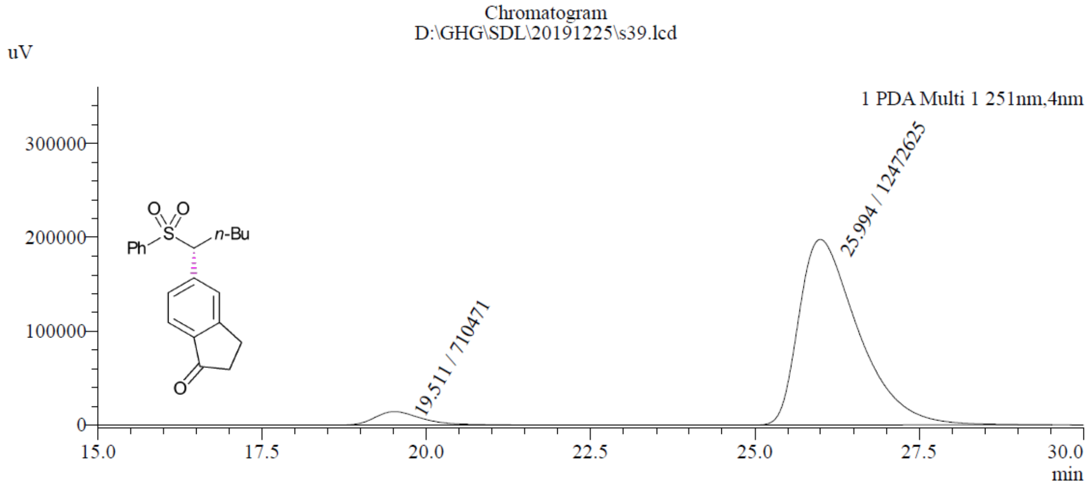

Peak Table

PDA Ch1 251nm

Peak#	Ret. Time [min]	Width [min]	Area [uV*s]	Height [uV]	Area %
1	19.511	0.7415	710471.24	14300.07	5.3893
2	25.994	0.9510	12472624.77	197825.89	94.6107

16: racemic

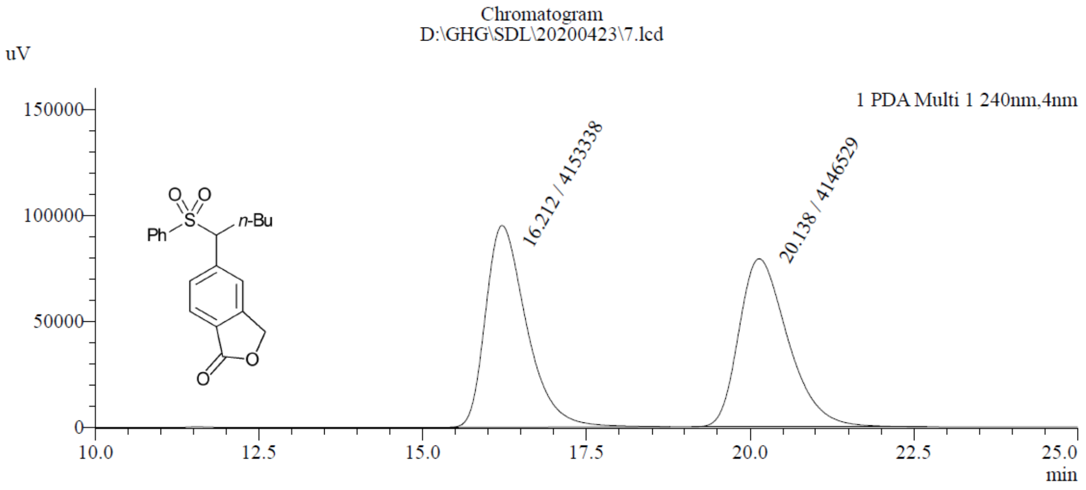

Peak Table

PDA Ch1 240nm

Peak#	Ret. Time [min]	Width [min]	Area [uV*s]	Height [uV]	Area %
1	16.212	0.6545	4153338.44	95174.11	50.0410
2	20.138	0.7969	4146529.35	79313.62	49.9590

16: enantioenriched, 92% ee

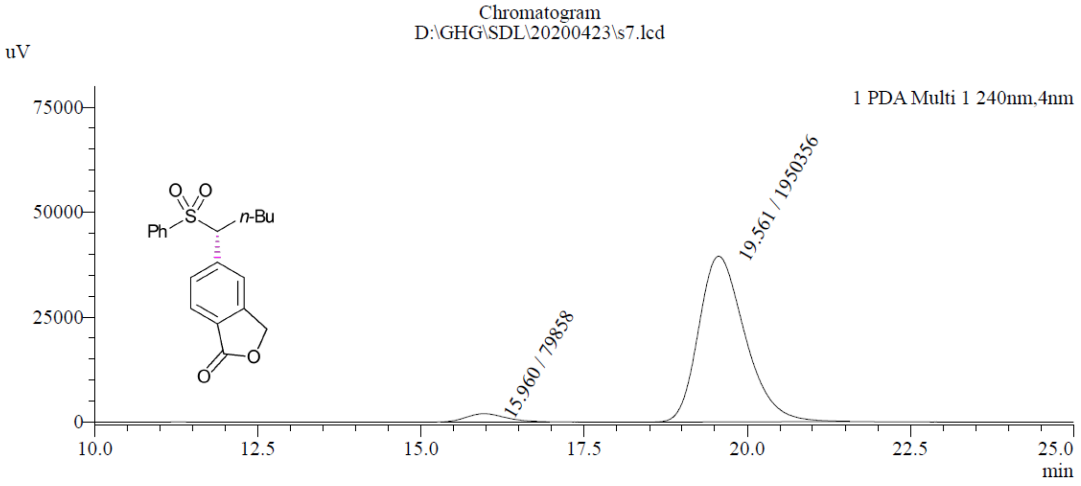

Peak Table

PDA Ch1 240nm

Peak#	Ret. Time [min]	Width [min]	Area [uV*s]	Height [uV]	Area %
1	15.960	0.6442	79858.30	1935.24	3.9335
2	19.561	0.7555	1950355.79	39442.56	96.0665

17: racemic

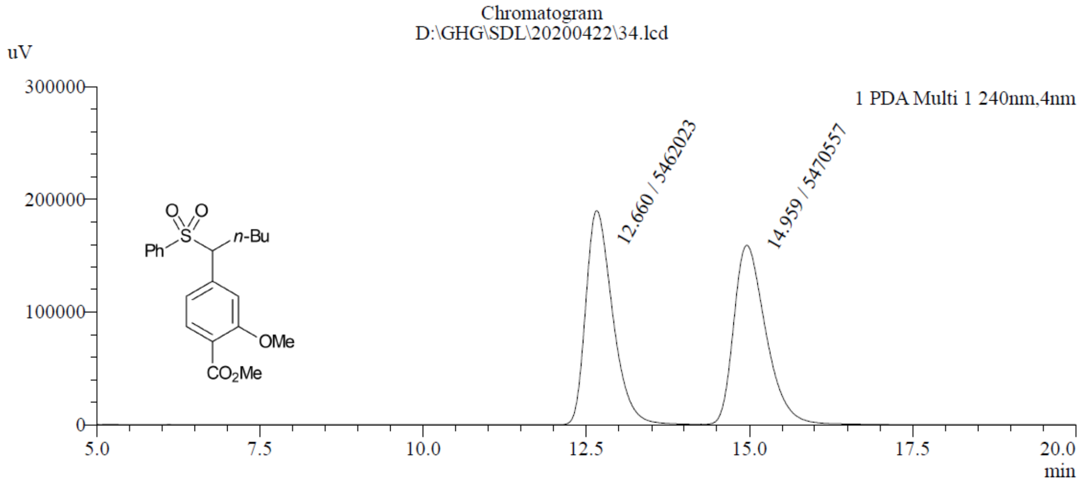

Peak Table

PDA Ch1 240nm

Peak#	Ret. Time [min]	Width [min]	Area [uV*s]	Height [uV]	Area %
1	12.660	0.4353	5462022.98	190092.99	49.9610
2	14.959	0.5207	5470556.97	159214.57	50.0390

17: enantioenriched, 83% ee

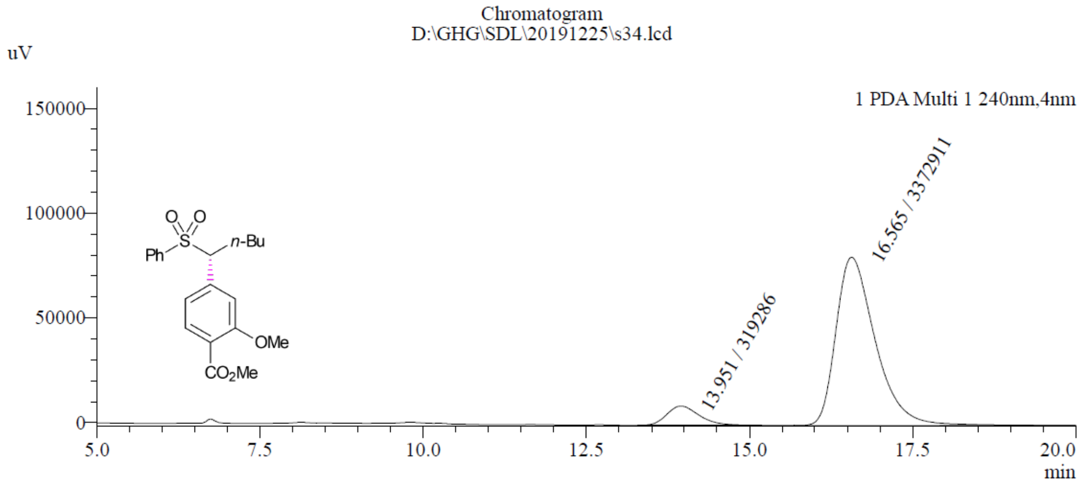

Peak Table

PDA Ch1 240nm

Peak#	Ret. Time [min]	Width [min]	Area [uV*s]	Height [uV]	Area %
1	13.951	0.5274	319285.86	9212.33	8.6476
2	16.565	0.6275	3372911.04	80342.14	91.3524

18: racemic

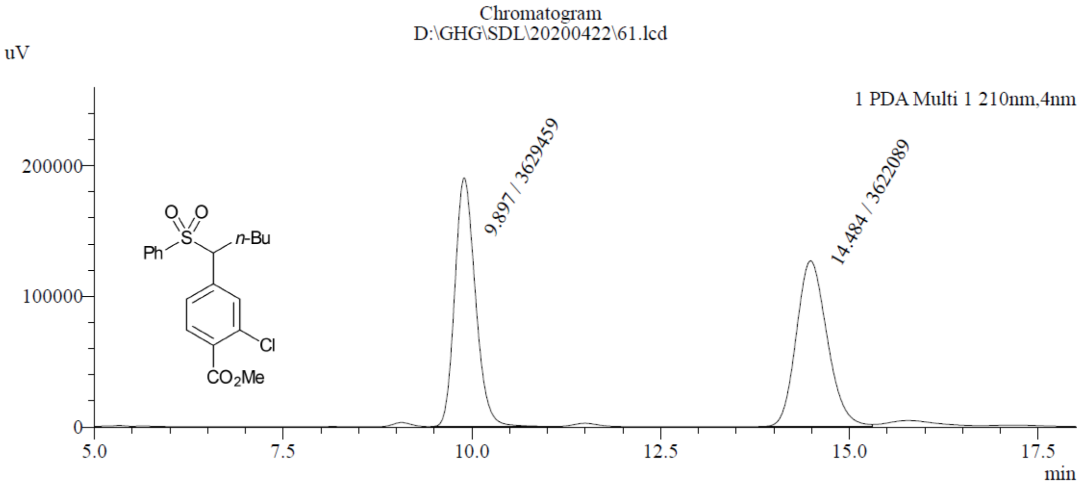

Peak Table

PDA Ch1 210nm

Peak#	Ret. Time [min]	Width [min]	Area [uV*s]	Height [uV]	Area %
1	9.897	0.2902	3629459.14	190416.93	50.0508
2	14.484	0.4384	3622088.98	126890.36	49.9492

18: enantioenriched, 81% ee

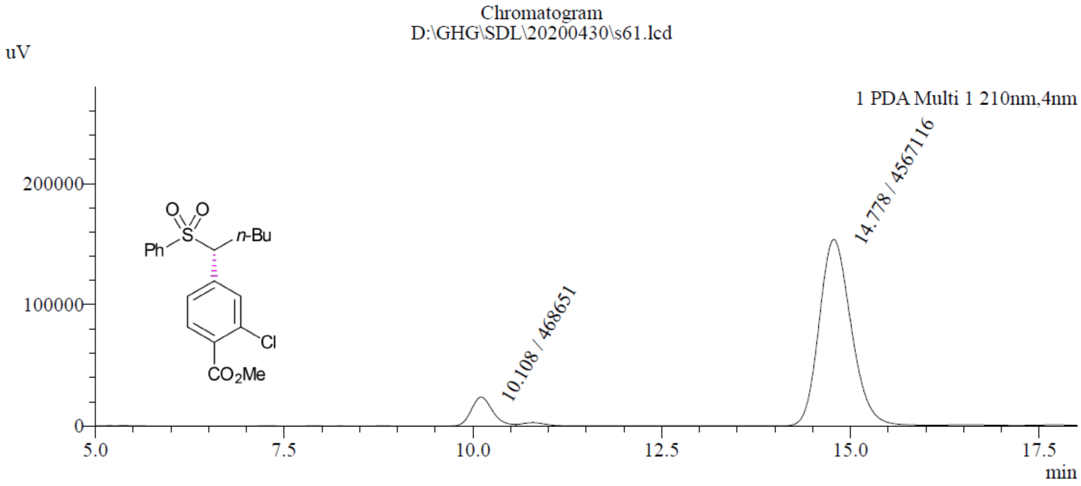

Peak Table

PDA Ch1 210nm

Peak#	Ret. Time [min]	Width [min]	Area [uV*s]	Height [uV]	Area %
1	10.108	0.2991	468651.05	23865.13	9.3064
2	14.778	0.4534	4567116.47	154022.44	90.6936

19: racemic

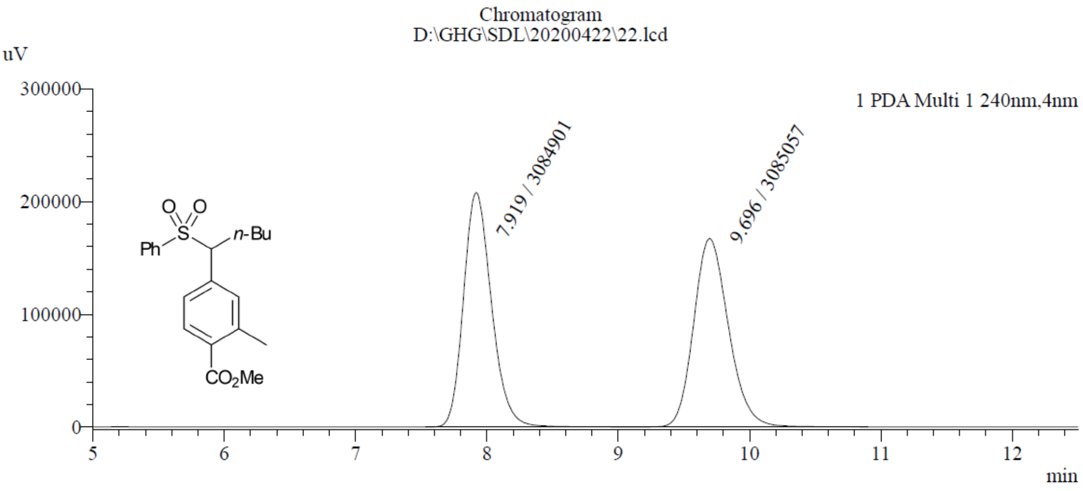

Peak Table

PDA Ch1 240nm

Peak#	Ret. Time [min]	Width [min]	Area [uV*s]	Height [uV]	Area %
1	7.919	0.2261	3084900.80	207930.37	49.9987
2	9.696	0.2822	3085057.05	167206.74	50.0013

19: enantioenriched, 92% ee

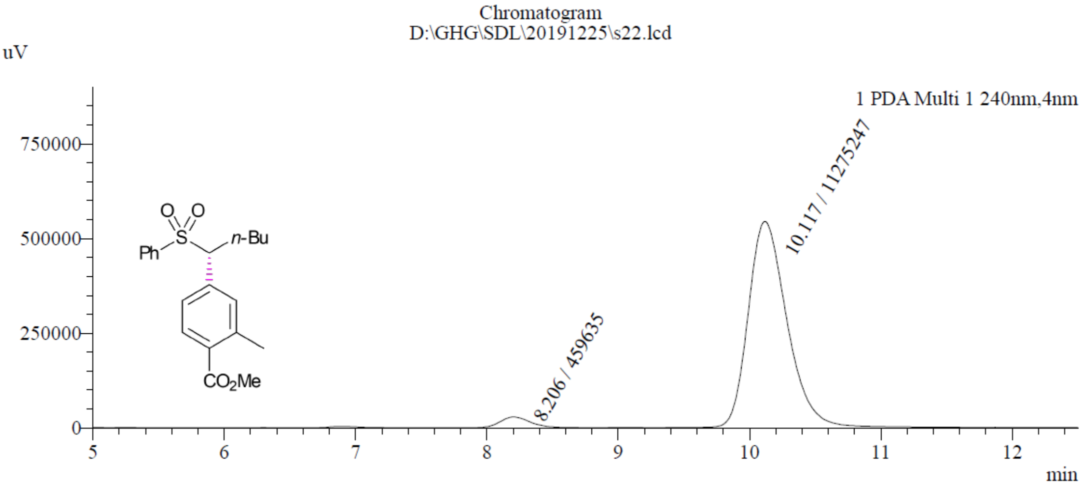

Peak Table

PDA Ch1 240nm

Peak#	Ret. Time [min]	Width [min]	Area [uV*s]	Height [uV]	Area %
1	8.206	0.2454	459635.02	28466.14	3.9168
2	10.117	0.3111	11275247.06	545015.28	96.0832

20: racemic

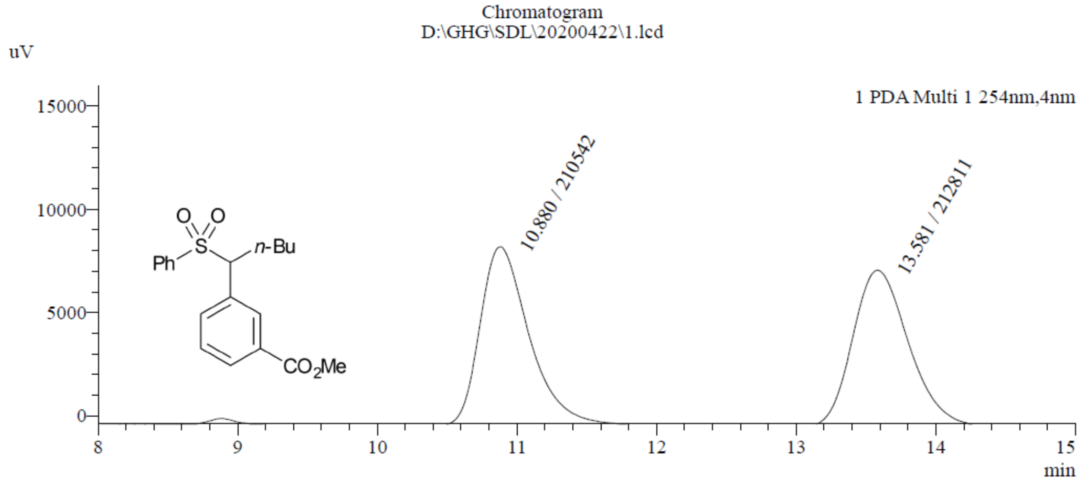

Peak Table

PDA Ch1 254nm

Peak#	Ret. Time [min]	Width [min]	Area [uV*s]	Height [uV]	Area %
1	10.880	0.3658	210541.64	8640.28	49.7319
2	13.581	0.4322	212811.42	7596.24	50.2681

20: enantioenriched, 86% ee

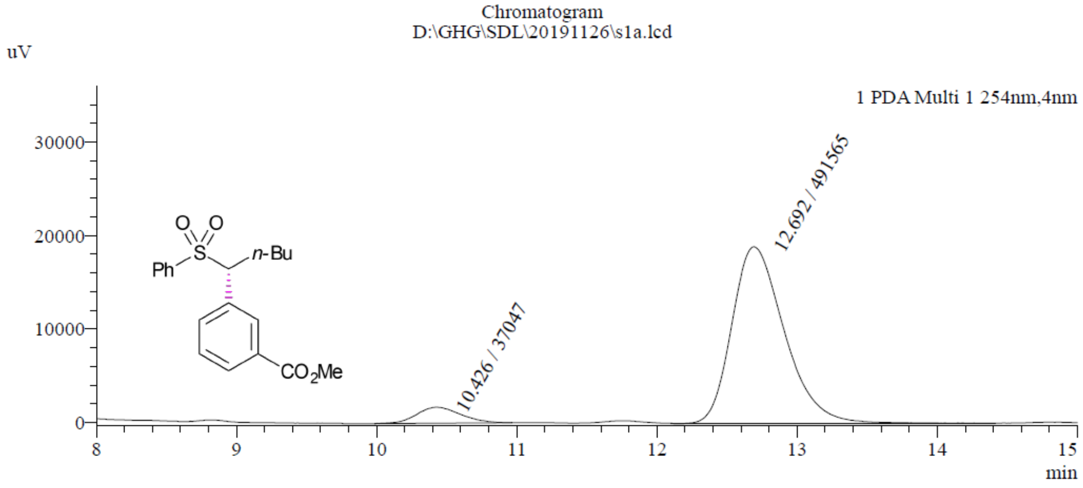

Peak Table

PDA Ch1 254nm

Peak#	Ret. Time [min]	Width [min]	Area [uV*s]	Height [uV]	Area %
1	10.426	0.3388	37046.67	1703.26	7.0083
2	12.692	0.3968	491565.43	18882.42	92.9917

21: racemic

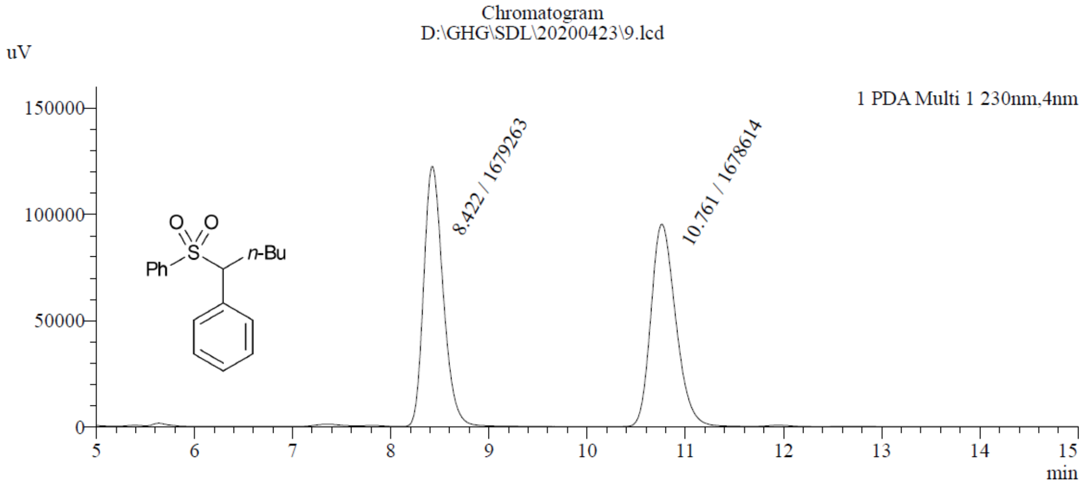

Peak Table

PDA Ch1 230nm

Peak#	Ret. Time [min]	Width [min]	Area [uV*s]	Height [uV]	Area %
1	8.422	0.2092	1679262.51	122342.79	50.0097
2	10.761	0.2698	1678613.76	95304.71	49.9903

21: enantioenriched, 89% ee

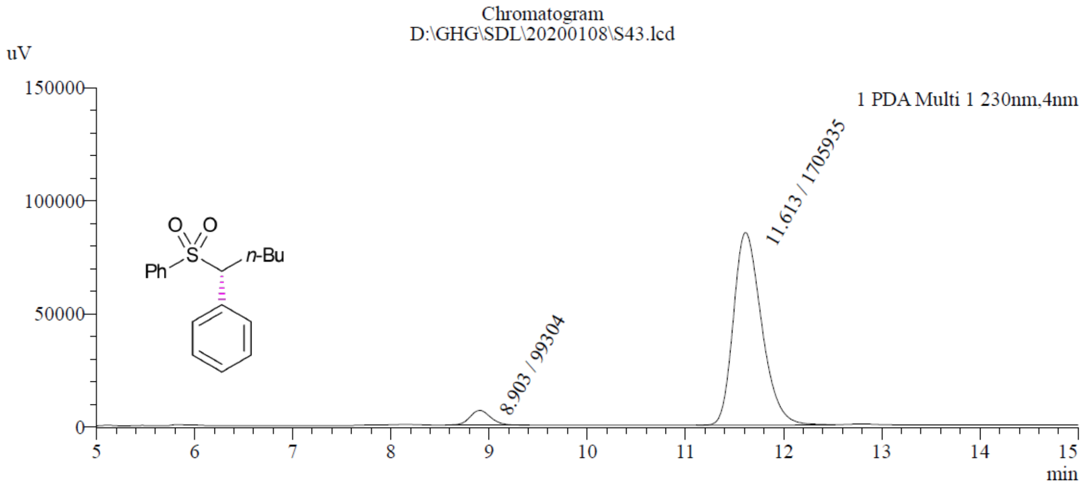

Peak Table

PDA Ch1 230nm

Peak#	Ret. Time [min]	Width [min]	Area [uV*s]	Height [uV]	Area %
1	8.903	0.2268	99304.42	6576.21	5.5009
2	11.613	0.3057	1705935.38	85199.81	94.4991

22: racemic

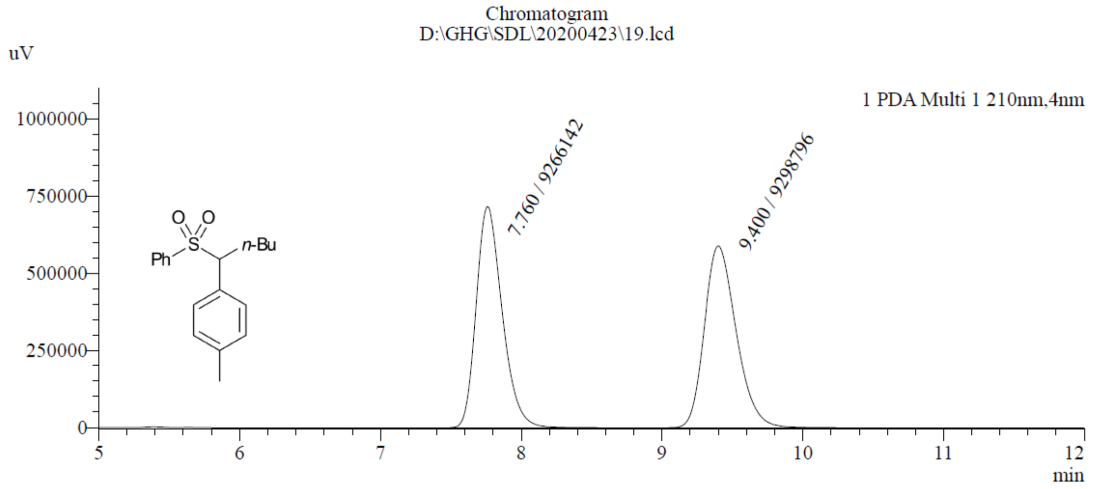

Peak Table

PDA Ch1 210nm

Peak#	Ret. Time [min]	Width [min]	Area [uV*s]	Height [uV]	Area %
1	7.760	0.1973	9266141.86	716510.86	49.9121
2	9.400	0.2413	9298796.21	589506.40	50.0879

22: enantioenriched, 89% ee

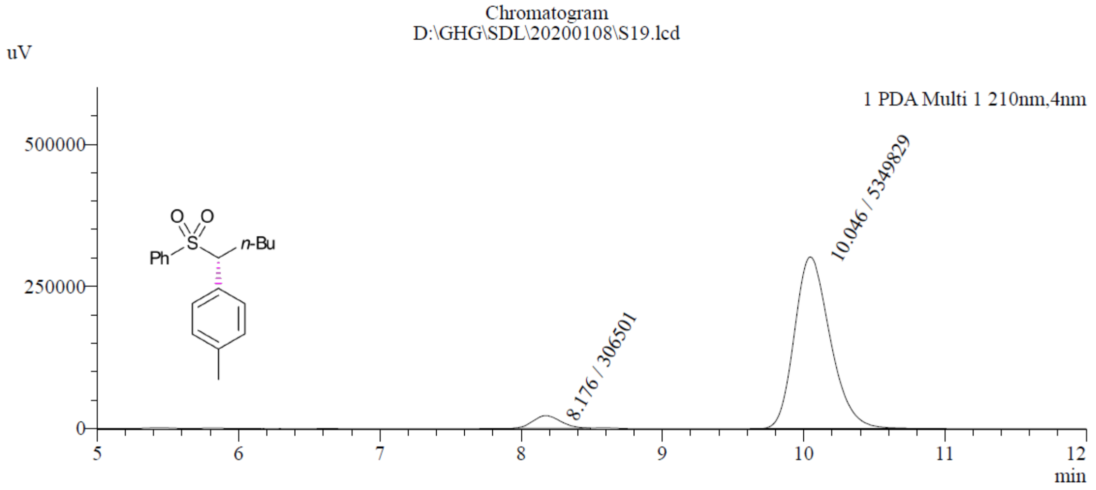

Peak Table

PDA Ch1 210nm

Peak#	Ret. Time [min]	Width [min]	Area [uV*s]	Height [uV]	Area %
1	8.176	0.2158	306500.80	22312.94	5.4187
2	10.046	0.2703	5349829.25	302492.60	94.5813

23: racemic

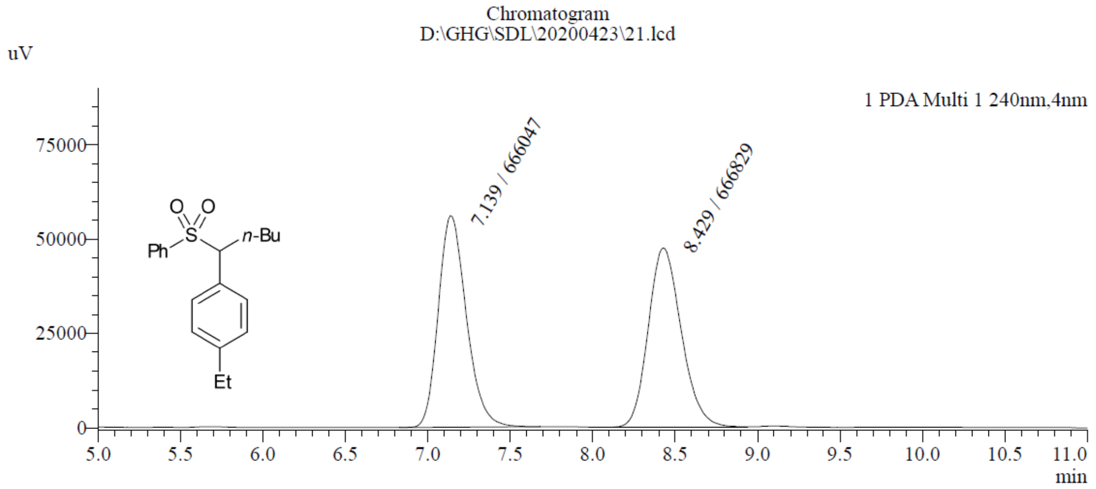

Peak Table

PDA Ch1 240nm

Peak#	Ret. Time [min]	Width [min]	Area [uV*s]	Height [uV]	Area %
1	7.139	0.1814	666046.54	56080.25	49.9707
2	8.429	0.2151	666828.78	47562.66	50.0293

23: enantioenriched, 88% ee

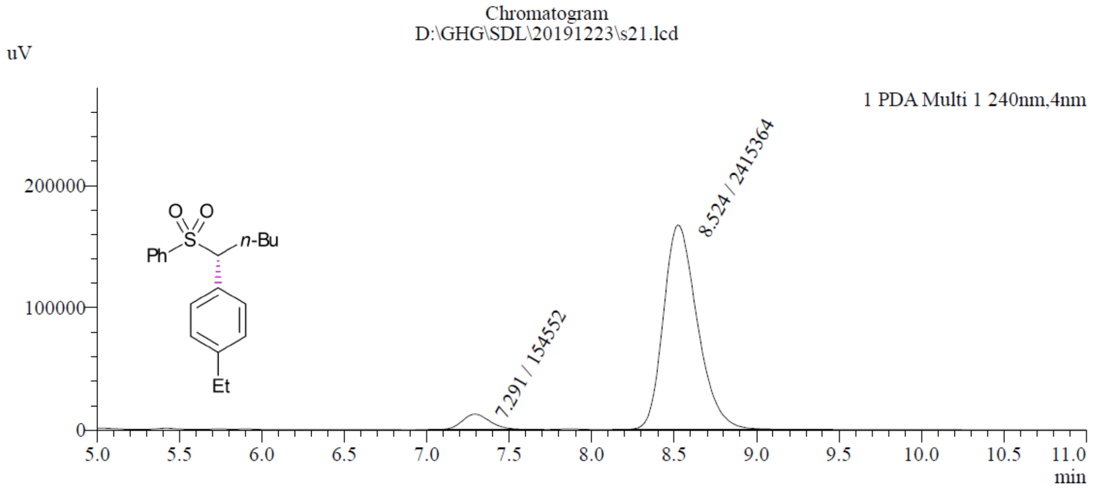

Peak Table

PDA Ch1 240nm

Peak#	Ret. Time [min]	Width [min]	Area [uV*s]	Height [uV]	Area %
1	7.291	0.1832	154551.77	12718.56	6.0139
2	8.524	0.2192	2415364.06	167646.39	93.9861

24: racemic

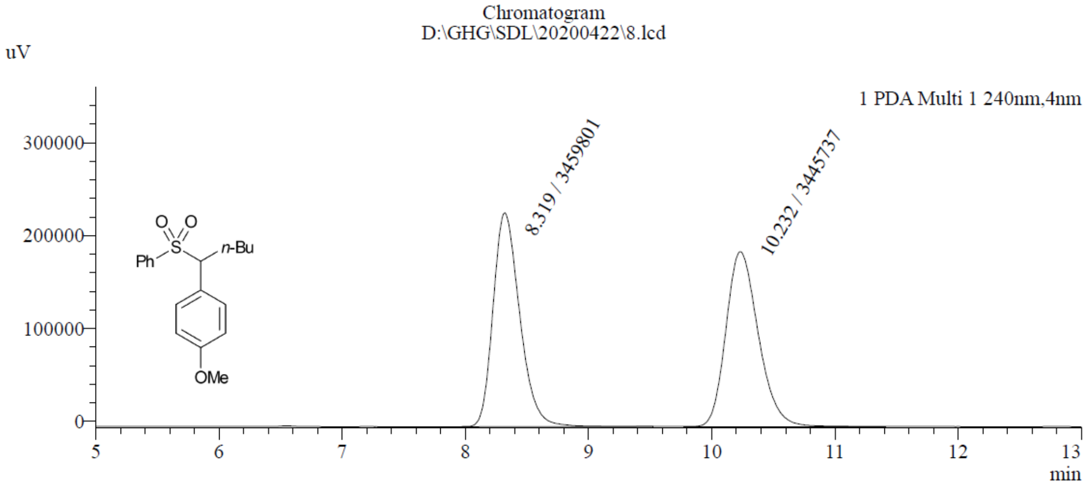

Peak Table

PDA Ch1 240nm

Peak#	Ret. Time [min]	Width [min]	Area [uV*s]	Height [uV]	Area %
1	8.319	0.2288	3459801.14	230114.75	50.1018
2	10.232	0.2801	3445737.02	188399.00	49.8982

24: enantioenriched, 88% ee

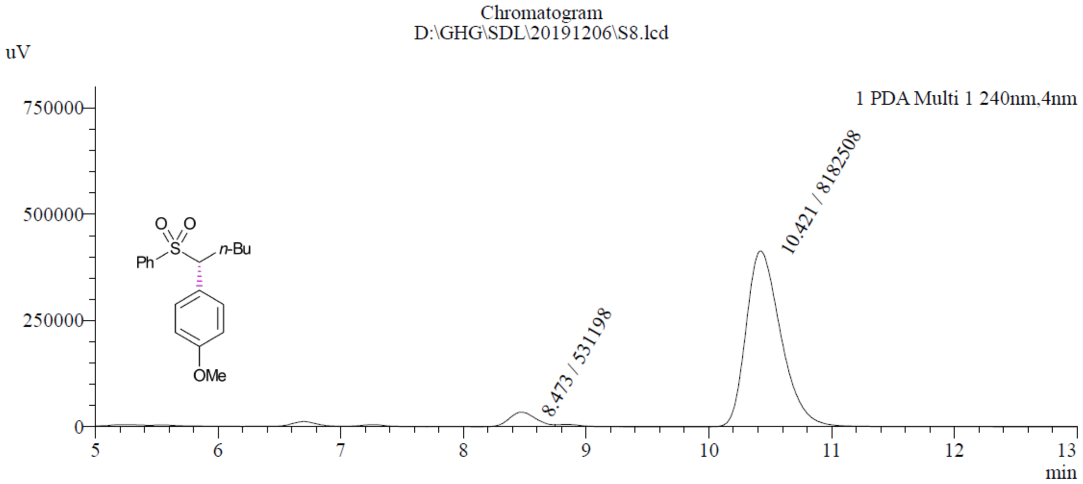

Peak Table

PDA Ch1 240nm

Peak#	Ret. Time [min]	Width [min]	Area [uV*s]	Height [uV]	Area %
1	8.473	0.2382	531198.34	34501.39	6.0961
2	10.421	0.3009	8182507.63	414076.30	93.9039

25: racemic

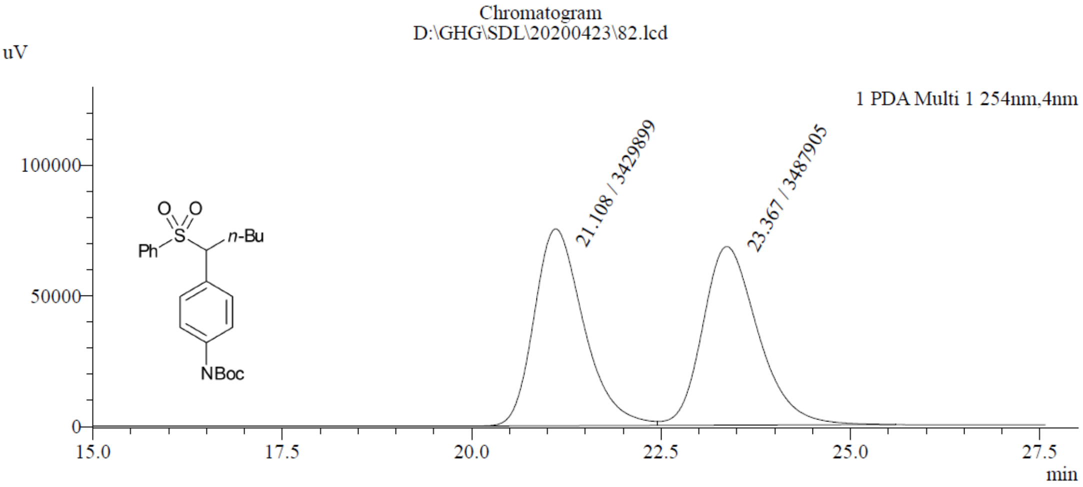

Peak Table

PDA Ch1 254nm

Peak#	Ret. Time [min]	Width [min]	Area [uV*s]	Height [uV]	Area %
1	21.108	0.6876	3429898.82	75397.72	49.5807
2	23.367	0.7704	3487904.83	68397.71	50.4193

25: enantioenriched, 90% ee

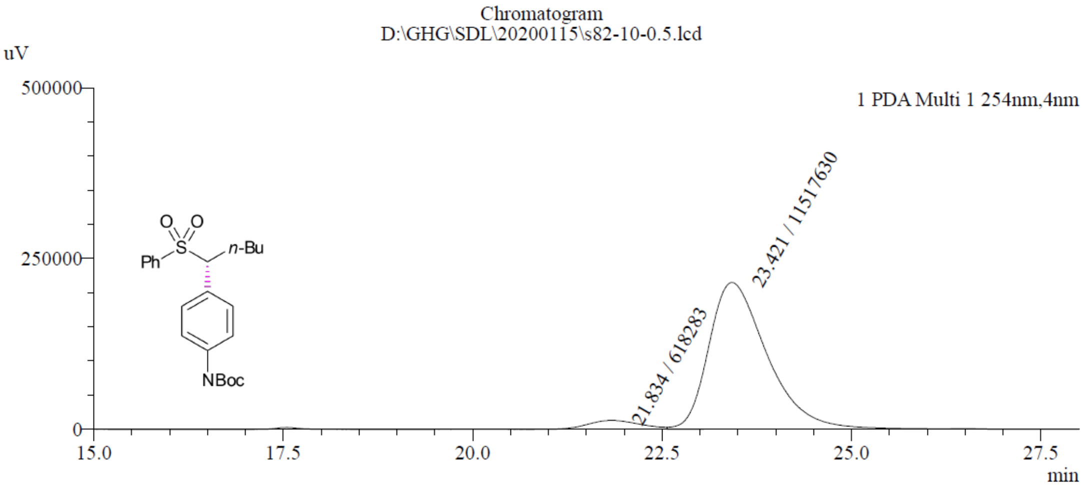

Peak Table

PDA Ch1 254nm

Peak#	Ret. Time [min]	Width [min]	Area [uV*s]	Height [uV]	Area %
1	21.834	0.7589	618282.55	12974.63	5.0947
2	23.421	0.8042	11517630.02	215133.32	94.9053

26: racemic

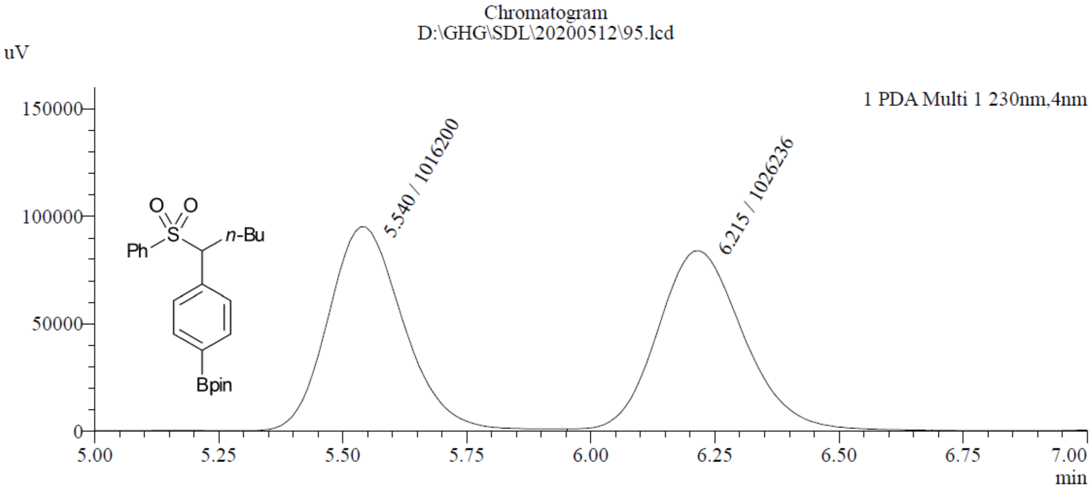

Peak Table

PDA Ch1 230nm

Peak#	Ret. Time [min]	Width [min]	Area [uV*s]	Height [uV]	Area %
1	5.540	0.1619	1016199.91	95264.63	49.7543
2	6.215	0.1852	1026235.70	84052.86	50.2457

26: enantioenriched, 90% ee

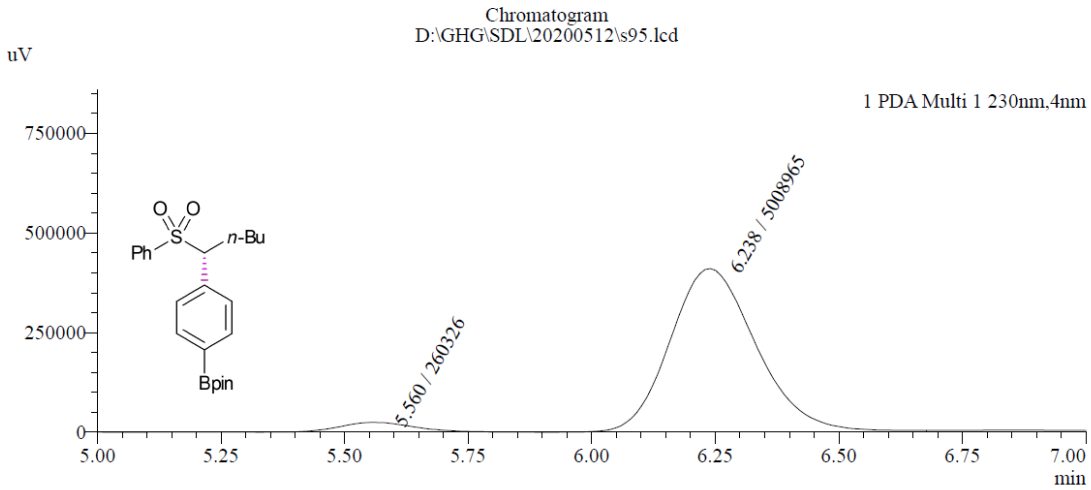

Peak Table

PDA Ch1 230nm

Peak#	Ret. Time [min]	Width [min]	Area [uV*s]	Height [uV]	Area %
1	5.560	0.1623	260326.12	24573.02	4.9404
2	6.238	0.1861	5008965.09	410794.60	95.0596

27: racemic

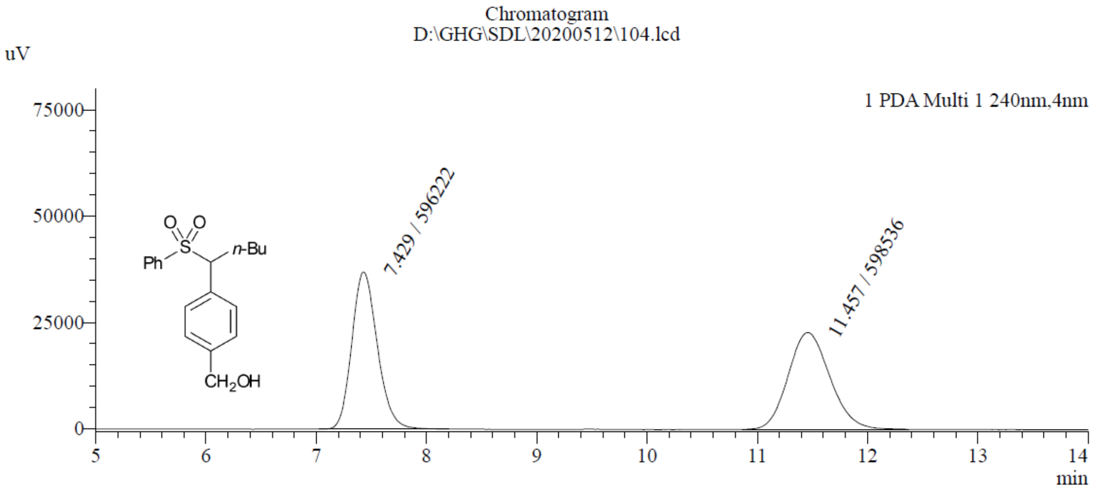

Peak Table

PDA Ch1 240nm

Peak#	Ret. Time [min]	Width [min]	Area [uV*s]	Height [uV]	Area %
1	7.429	0.2470	596221.67	36966.05	49.9032
2	11.457	0.4065	598535.79	22788.76	50.0968

27: enantioenriched, 90% ee

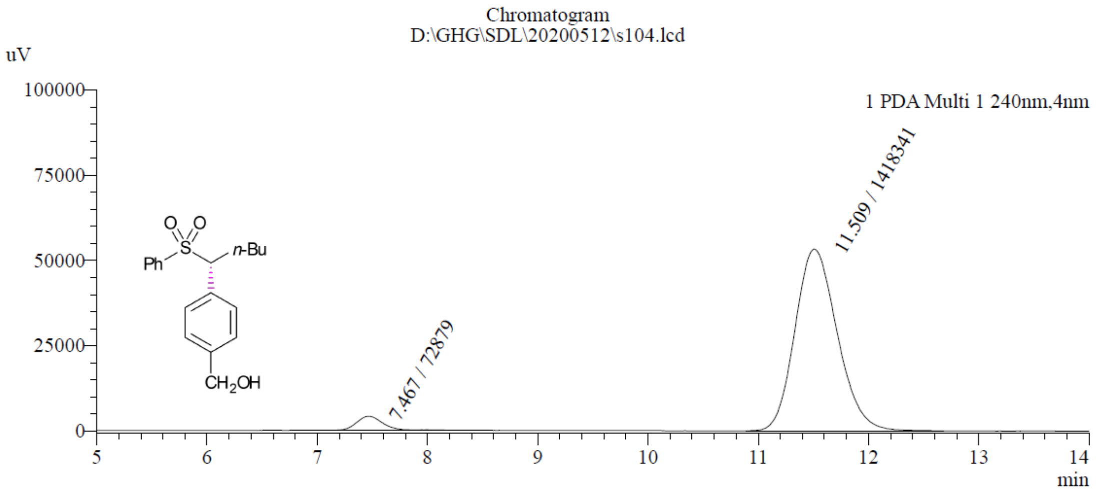

Peak Table

PDA Ch1 240nm

Peak#	Ret. Time [min]	Width [min]	Area [uV*s]	Height [uV]	Area %
1	7.467	0.2518	72879.18	4161.31	4.8872
2	11.509	0.4116	1418341.00	53246.24	95.1128

28: racemic

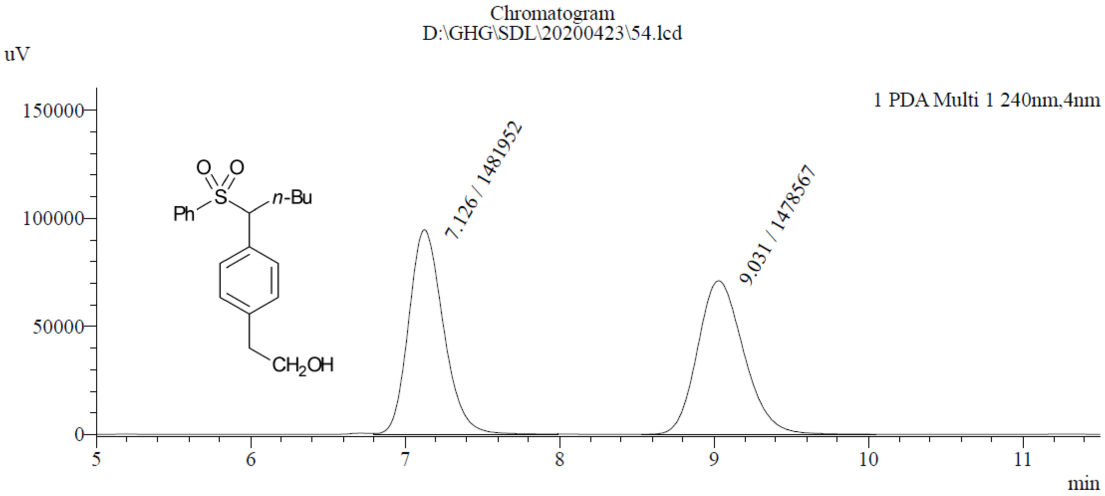

Peak Table

PDA Ch1 240nm

Peak#	Ret. Time [min]	Width [min]	Area [uV*s]	Height [uV]	Area %
1	7.126	0.2390	1481951.62	94663.07	50.0572
2	9.031	0.3202	1478567.32	71018.26	49.9428

28: enantioenriched, 89% ee

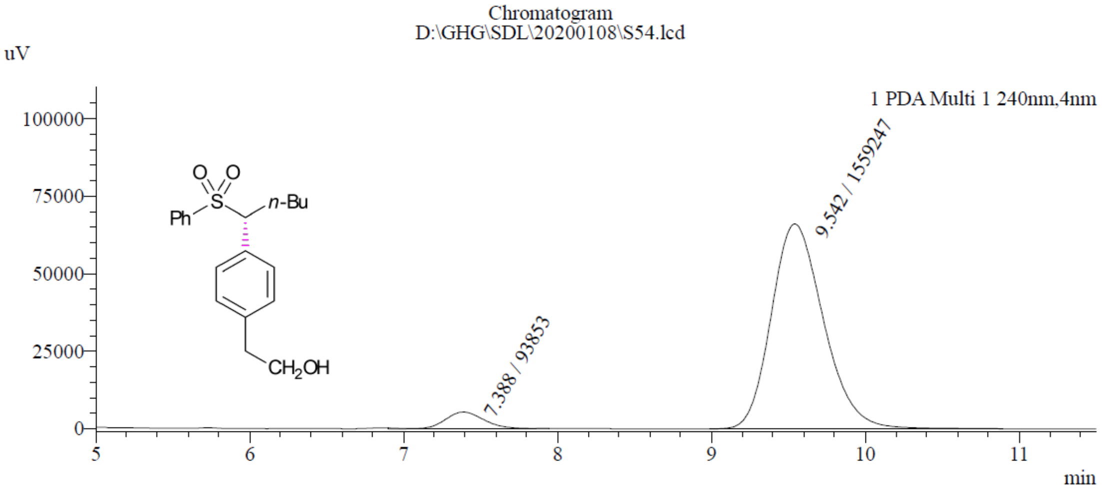

Peak Table

PDA Ch1 240nm

Peak#	Ret. Time [min]	Width [min]	Area [uV*s]	Height [uV]	Area %
1	7.388	0.2660	93852.62	5379.75	5.6774
2	9.542	0.3619	1559246.59	66026.67	94.3226

29: racemic

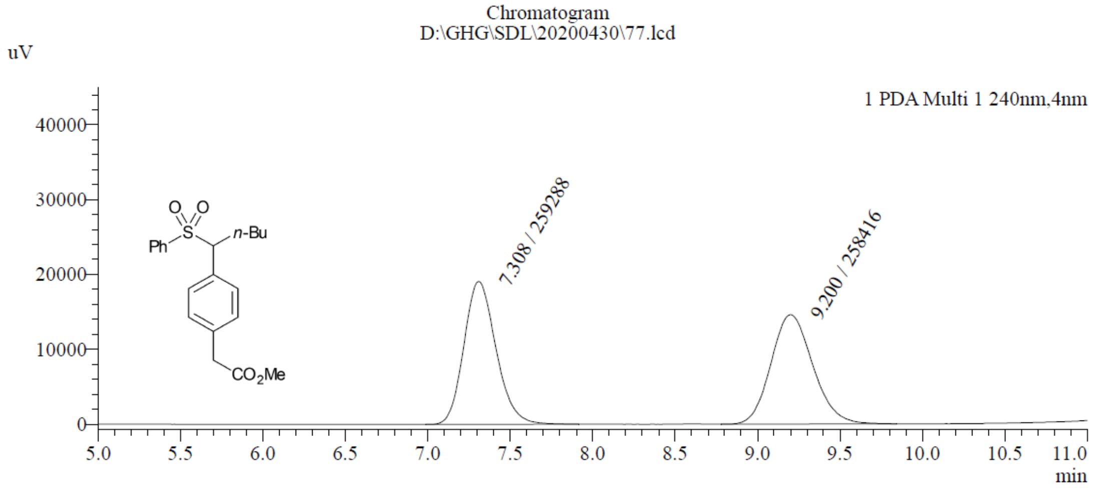

Peak Table

PDA Ch1 240nm

Peak#	Ret. Time [min]	Width [min]	Area [uV*s]	Height [uV]	Area %
1	7.308	0.2077	259287.95	19116.49	50.0842
2	9.200	0.2722	258415.92	14616.92	49.9158

29: enantioenriched, 92% ee

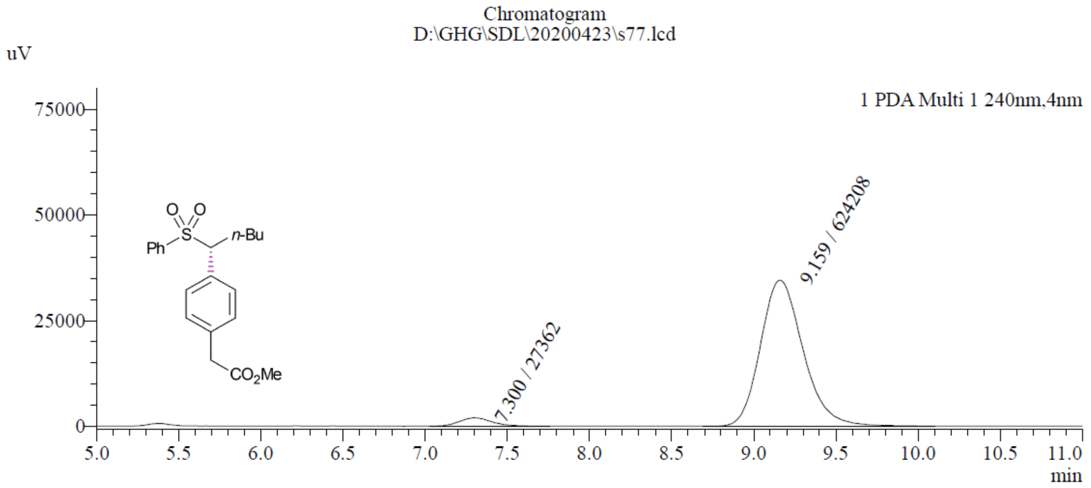

Peak Table

PDA Ch1 240nm

Peak#	Ret. Time [min]	Width [min]	Area [uV*s]	Height [uV]	Area %
1	7.300	0.2105	27361.77	1982.11	4.1994
2	9.159	0.2742	624208.11	34537.19	95.8006

30: racemic

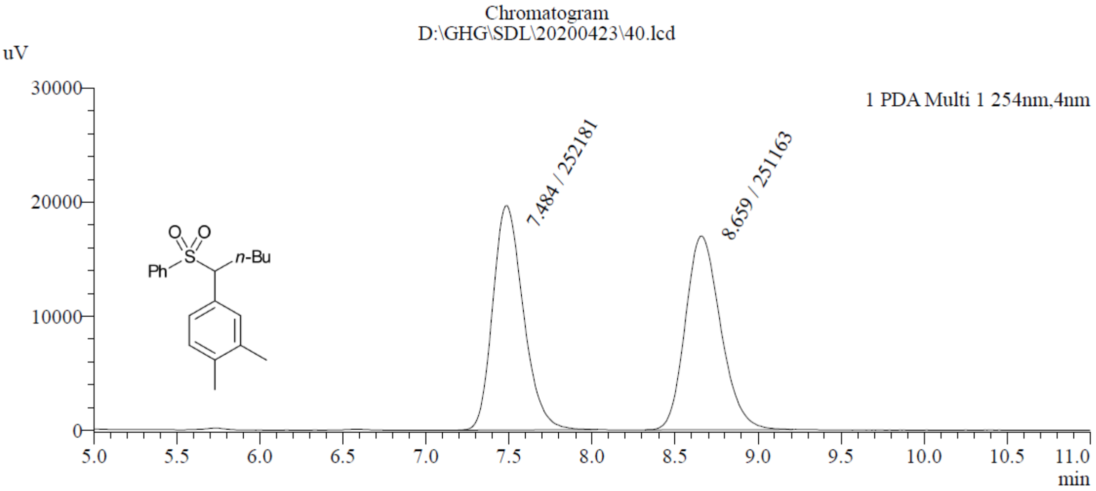

Peak Table

PDA Ch1 254nm

Peak#	Ret. Time [min]	Width [min]	Area [uV*s]	Height [uV]	Area %
1	7.484	0.1962	252180.94	19668.72	50.1012
2	8.659	0.2276	251162.54	16975.70	49.8988

30: enantioenriched, 85% ee

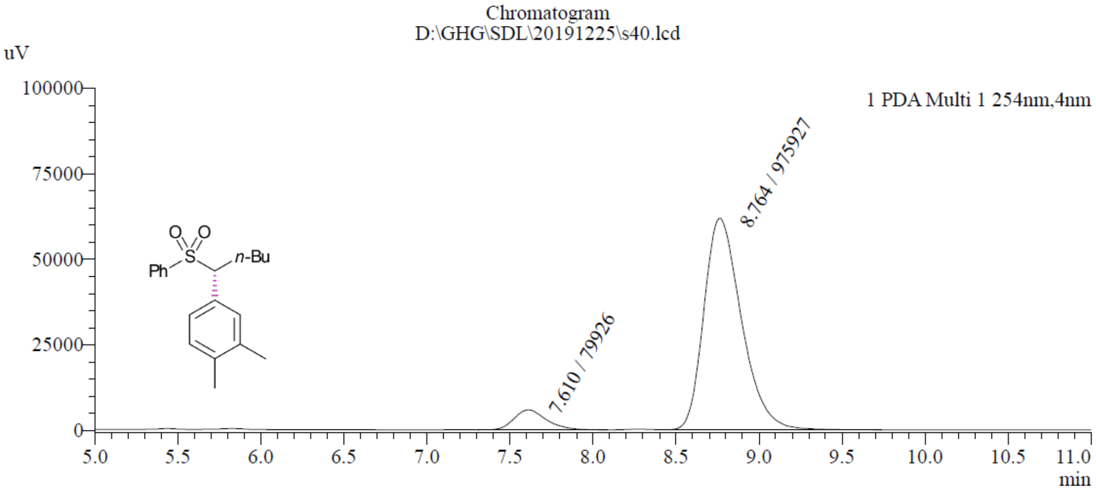

Peak Table

PDA Ch1 254nm

Peak#	Ret. Time [min]	Width [min]	Area [uV*s]	Height [uV]	Area %
1	7.610	0.2050	79925.74	5846.36	7.5698
2	8.764	0.2407	975927.35	61785.87	92.4302

31: racemic

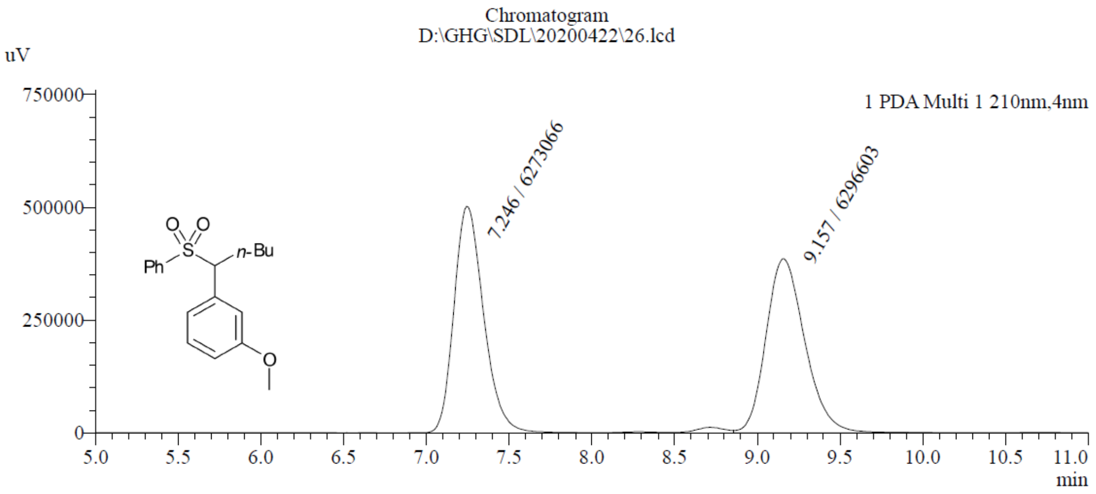

Peak Table

PDA Ch1 210nm

Peak#	Ret. Time [min]	Width [min]	Area [uV*s]	Height [uV]	Area %
1	7.246	0.1900	6273065.74	502465.21	49.9064
2	9.157	0.2494	6296602.82	385287.93	50.0936

31: enantioenriched, 86% ee

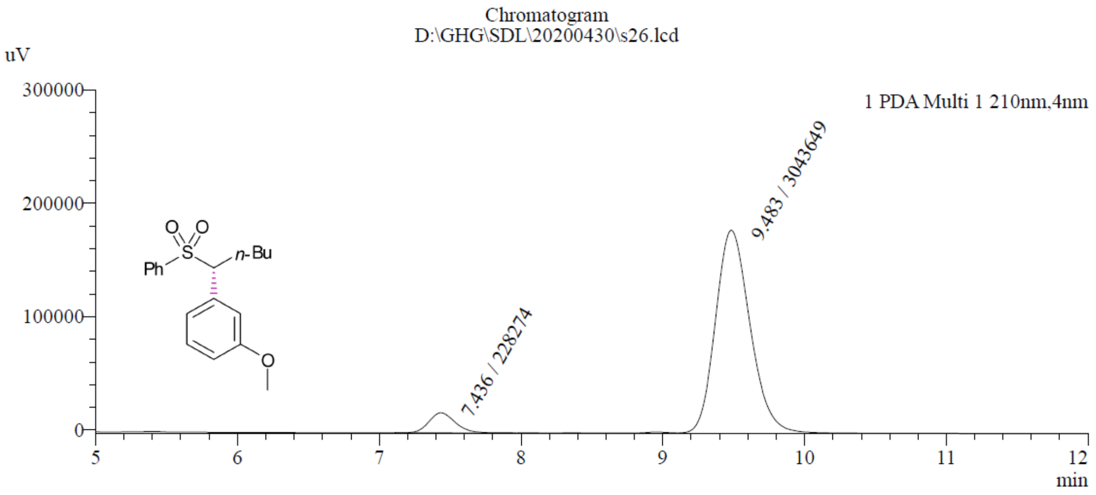

Peak Table

PDA Ch1 210nm

Peak#	Ret. Time [min]	Width [min]	Area [uV*s]	Height [uV]	Area %
1	7.436	0.1972	228274.47	17720.73	6.9768
2	9.483	0.2596	3043649.34	179135.73	93.0232

32: racemic

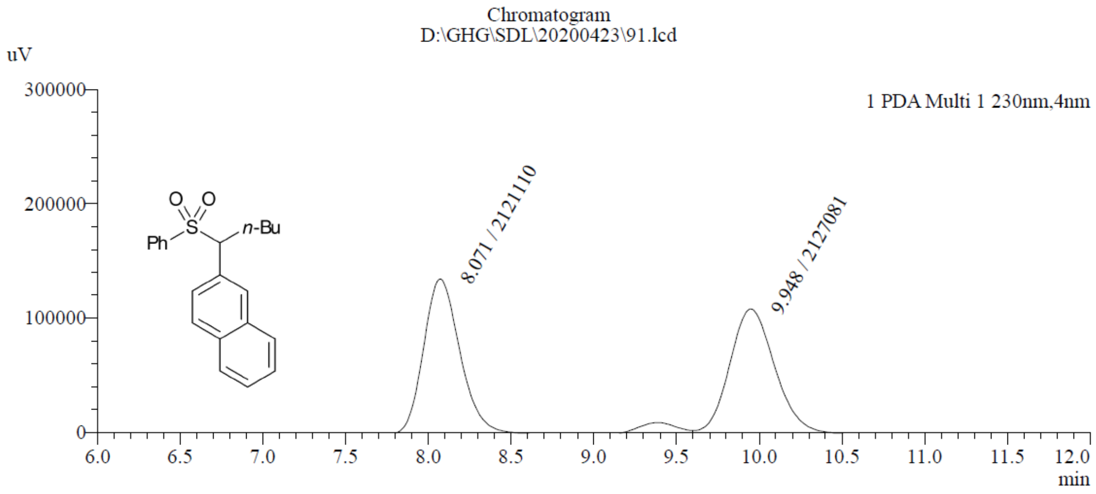

Peak Table

PDA Ch1 230nm

Peak#	Ret. Time [min]	Width [min]	Area [uV*s]	Height [uV]	Area %
1	8.071	0.2356	2121110.41	136120.55	49.9297
2	9.948	0.2937	2127080.57	109932.16	50.0703

32: enantioenriched, 90% ee

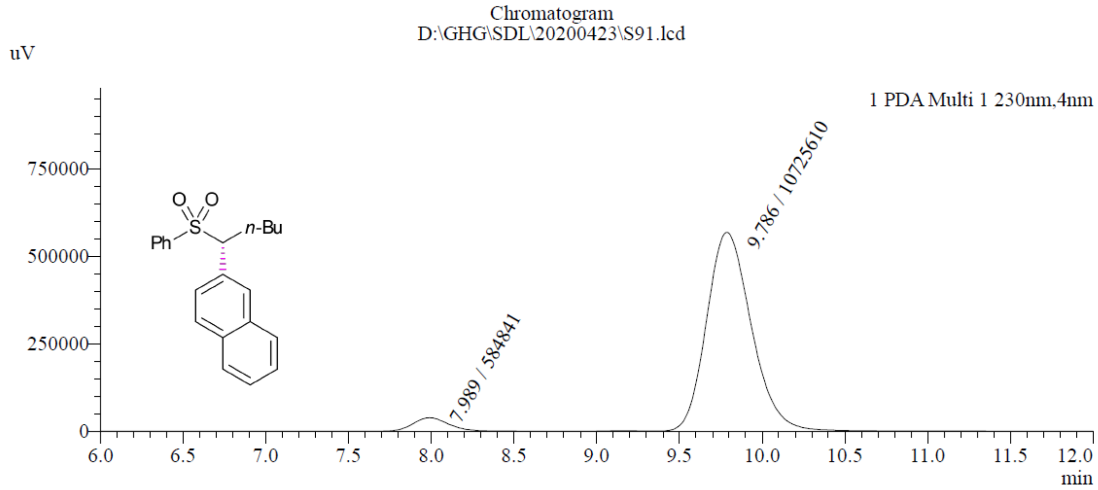

Peak Table

PDA Ch1 230nm

Peak#	Ret. Time [min]	Width [min]	Area [uV*s]	Height [uV]	Area %
1	7.989	0.2309	584841.13	38557.35	5.1708
2	9.786	0.2886	10725610.05	567833.47	94.8292

33: racemic

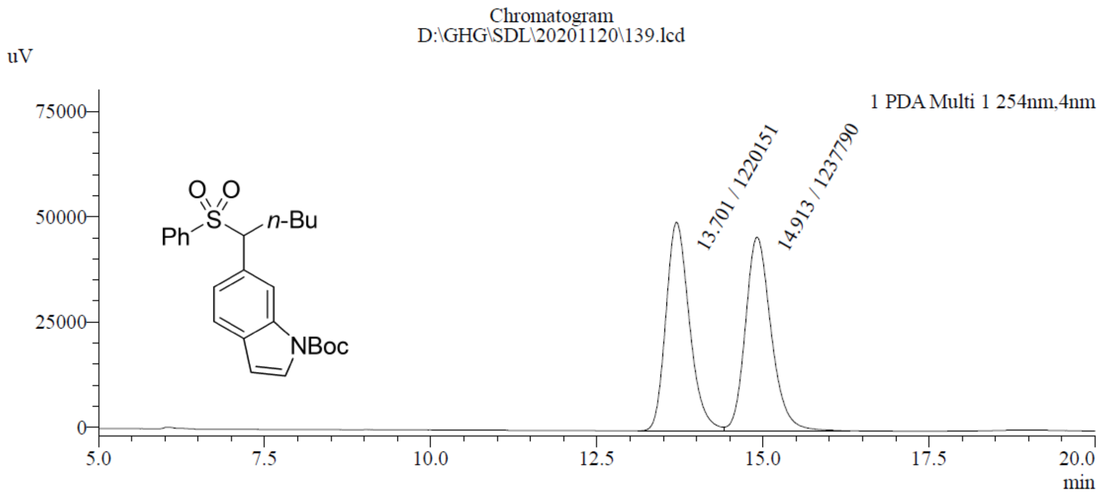

Peak Table

PDA Ch1 254nm

Peak#	Ret. Time [min]	Width [min]	Area [uV*s]	Height [uV]	Area %
1	13.701	0.3742	1220151.33	49515.09	49.6412
2	14.913	0.4052	1237789.90	45980.70	50.3588

33: enantioenriched, 87% ee

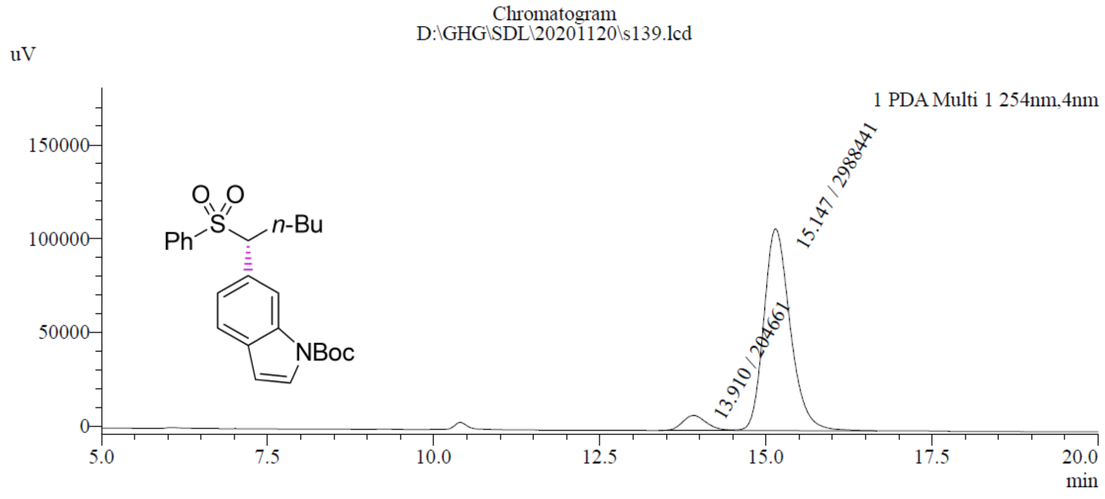

Peak Table

PDA Ch1 254nm

Peak#	Ret. Time [min]	Width [min]	Area [uV*s]	Height [uV]	Area %
1	13.910	0.3868	204660.57	8079.37	6.4095
2	15.147	0.4185	2988441.47	107671.33	93.5905

34: racemic

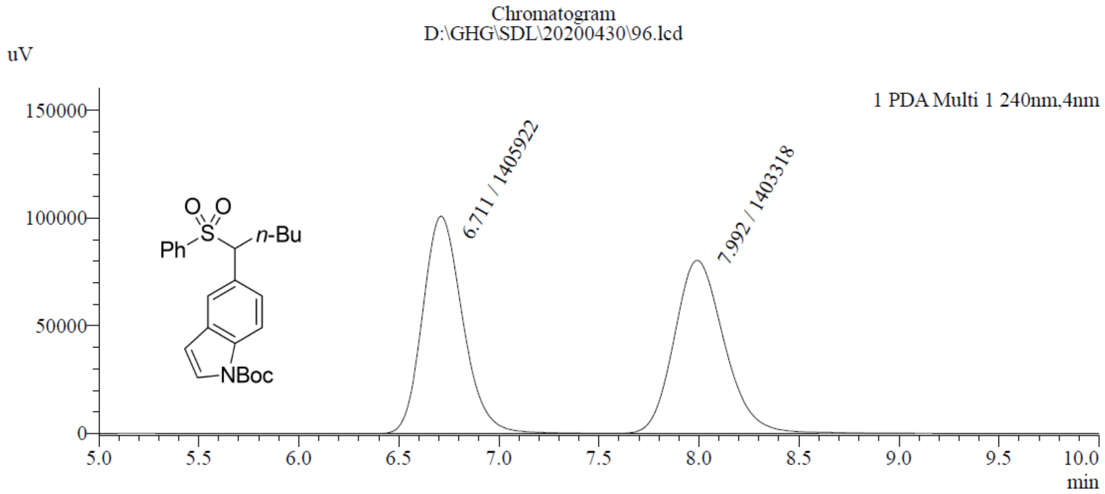

Peak Table

PDA Ch1 240nm

Peak#	Ret. Time [min]	Width [min]	Area [uV*s]	Height [uV]	Area %
1	6.711	0.2119	1405921.58	100969.53	50.0463
2	7.992	0.2656	1403318.03	80349.14	49.9537

34: enantioenriched, 82% ee

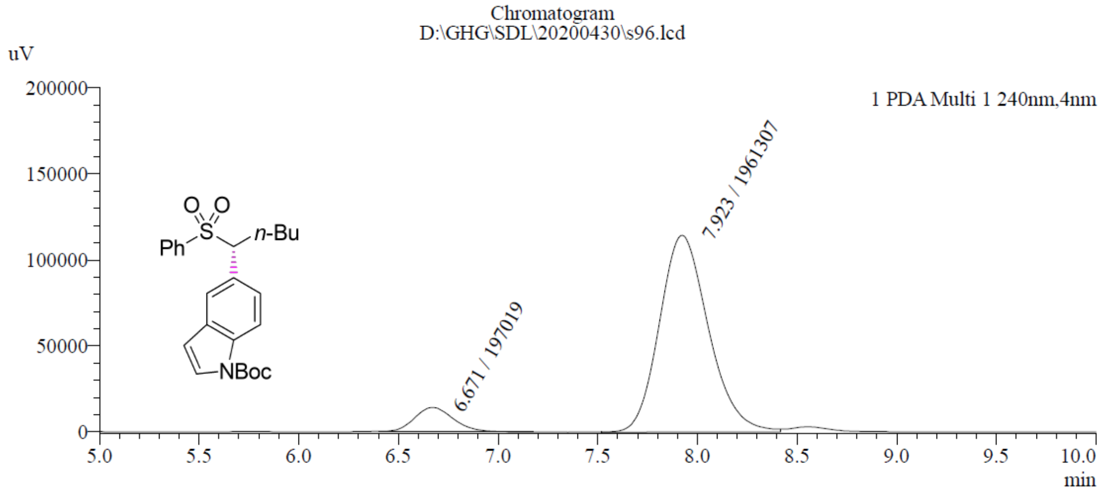

Peak Table

PDA Ch1 240nm

Peak#	Ret. Time [min]	Width [min]	Area [uV*s]	Height [uV]	Area %
1	6.671	0.2105	197018.58	14270.15	9.1283
2	7.923	0.2617	1961307.26	114474.64	90.8717

35: racemic

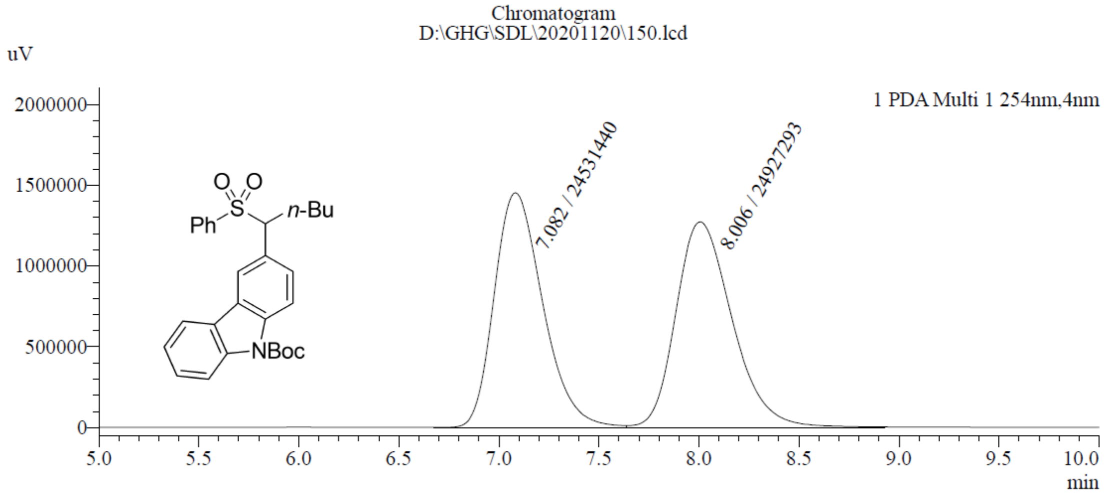

Peak Table

PDA Ch1 254nm

Peak#	Ret. Time [min]	Width [min]	Area [uV*s]	Height [uV]	Area %
1	7.082	0.2595	24531439.95	1453761.60	49.5998
2	8.006	0.2986	24927293.23	1273447.76	50.4002

35: enantioenriched, 85% ee

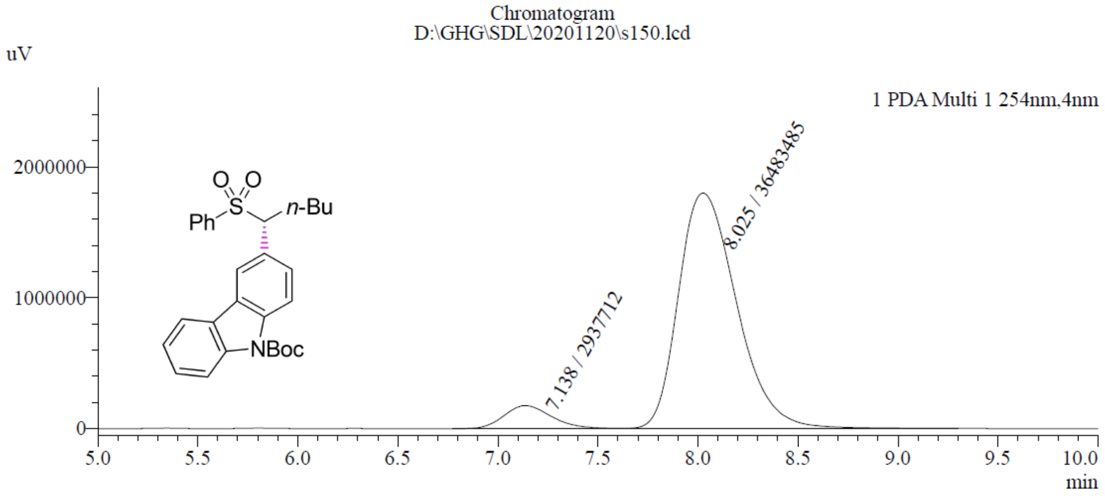

Peak Table

PDA Ch1 254nm

Peak#	Ret. Time [min]	Width [min]	Area [uV*s]	Height [uV]	Area %
1	7.138	0.2545	2937712.47	176556.48	7.4521
2	8.025	0.3114	36483485.22	1800515.21	92.5479

36: racemic

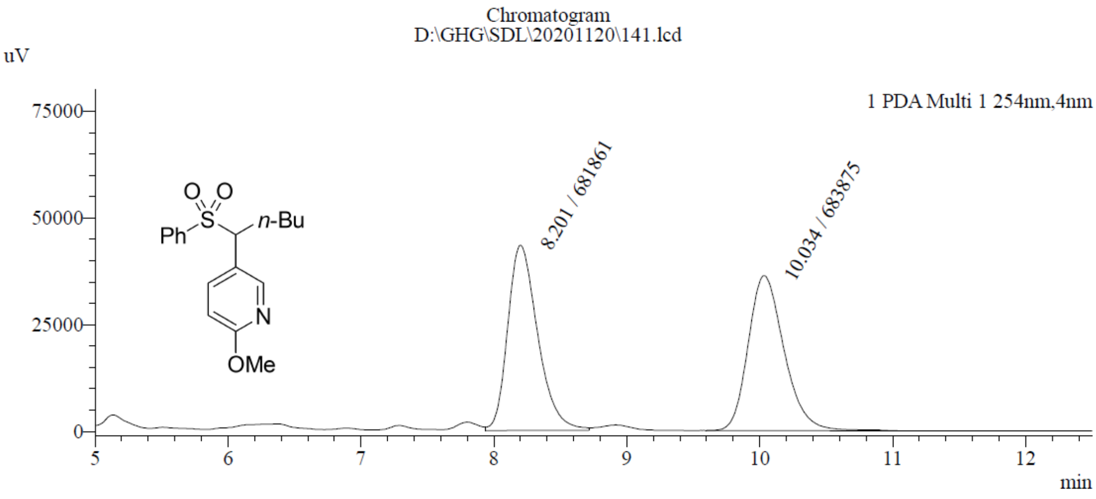

Peak Table

PDA Ch1 254nm

Peak#	Ret. Time [min]	Width [min]	Area [uV*s]	Height [uV]	Area %
1	8.201	0.2376	681860.63	43461.35	49.9263
2	10.034	0.2856	683875.06	36334.67	50.0737

36: enantioenriched, 87% ee

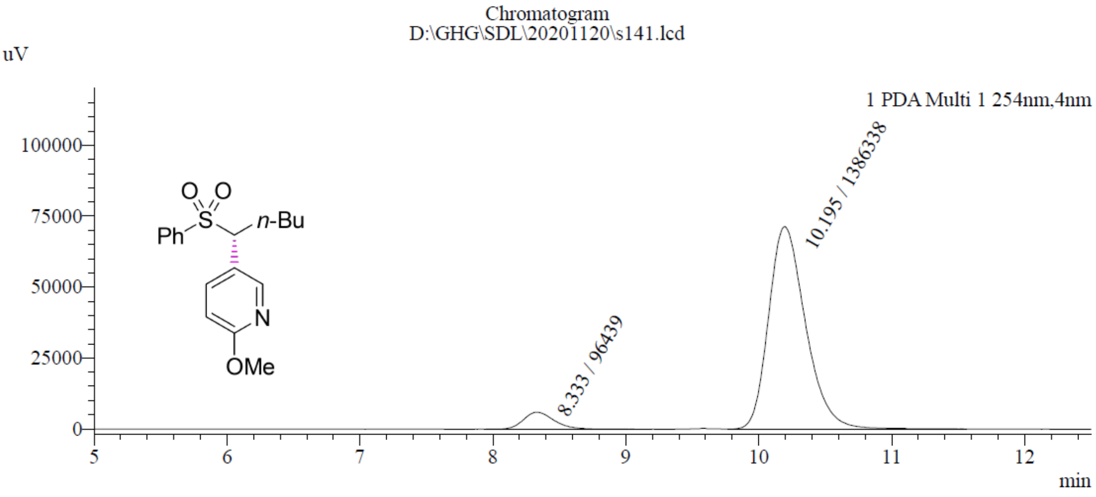

Peak Table

PDA Ch1 254nm

Peak#	Ret. Time [min]	Width [min]	Area [uV*s]	Height [uV]	Area %
1	8.333	0.2424	96438.50	6032.78	6.5039
2	10.195	0.2939	1386337.66	71408.47	93.4961

37: racemic

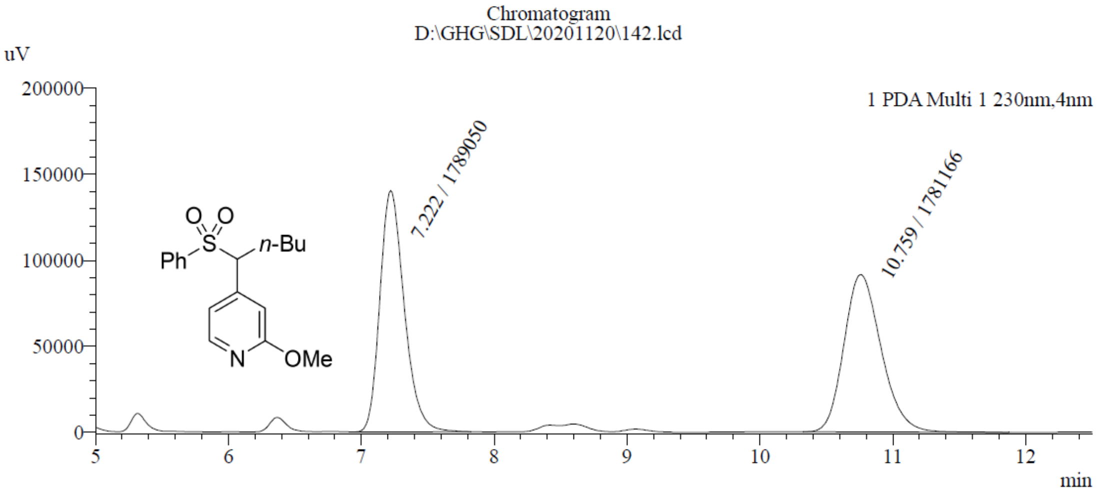

Peak Table

PDA Ch1 230nm

Peak#	Ret. Time [min]	Width [min]	Area [uV*s]	Height [uV]	Area %
1	7.222	0.1937	1789049.69	140272.63	50.1104
2	10.759	0.2986	1781165.98	91504.39	49.8896

37: enantioenriched, 87% ee

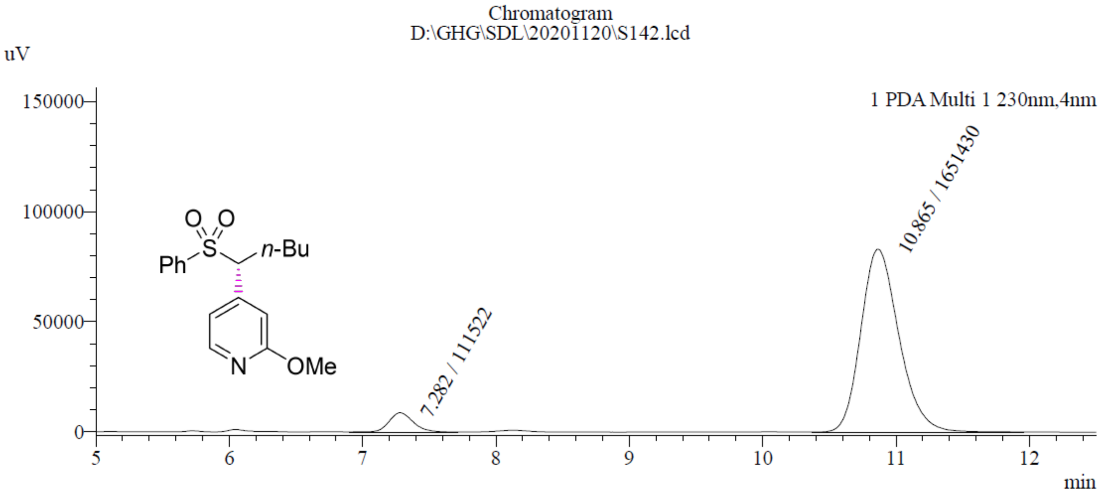

Peak Table

PDA Ch1 230nm

Peak#	Ret. Time [min]	Width [min]	Area [uV*s]	Height [uV]	Area %
1	7.282	0.1918	111521.70	8748.11	6.3258
2	10.865	0.3032	1651430.42	83097.78	93.6742

38: racemic

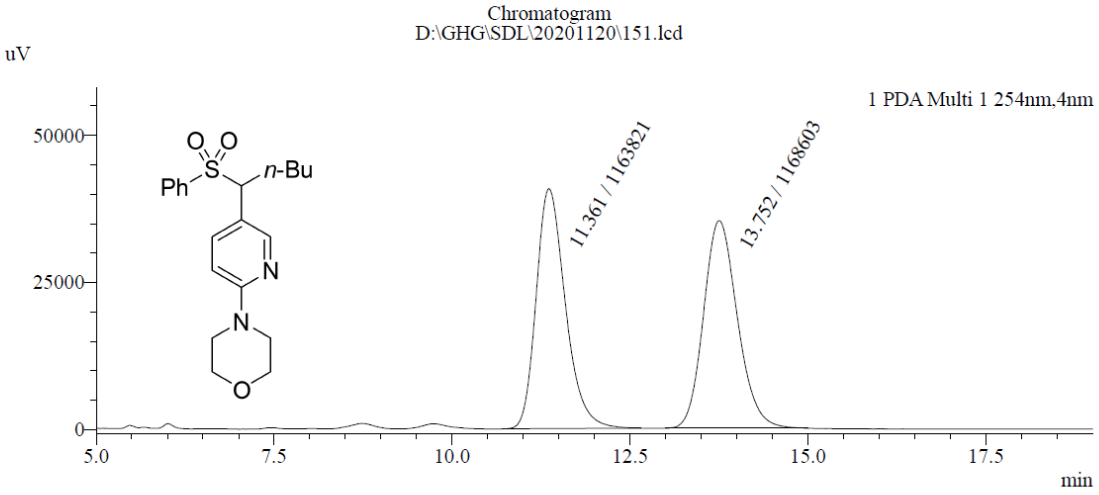

Peak Table

PDA Ch1 254nm

Peak#	Ret. Time [min]	Width [min]	Area [uV*s]	Height [uV]	Area %
1	11.361	0.4321	1163820.60	40759.23	49.8975
2	13.752	0.5075	1168603.47	35281.06	50.1025

38: enantioenriched, 91% ee

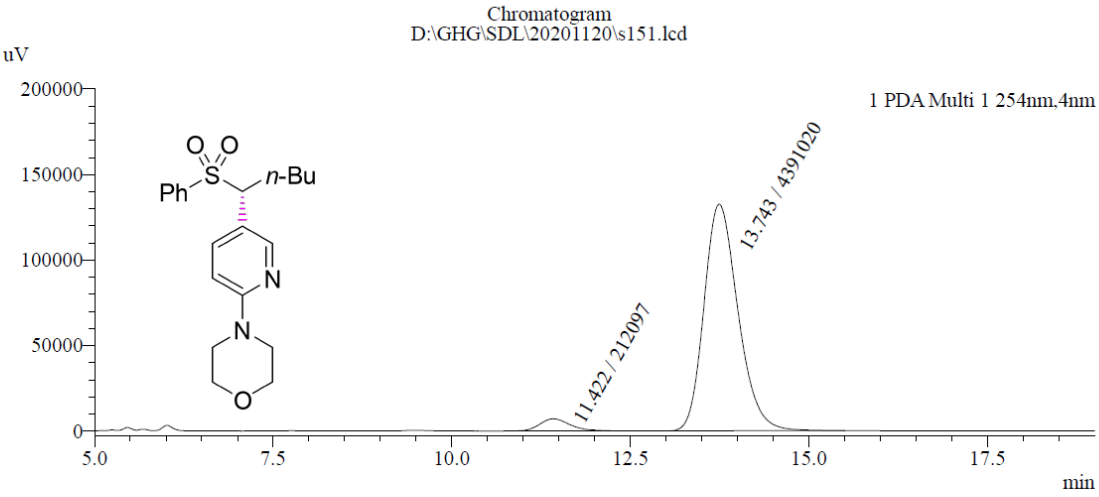

Peak Table

PDA Ch1 254nm

Peak#	Ret. Time [min]	Width [min]	Area [uV*s]	Height [uV]	Area %
1	11.422	0.4445	212097.28	7145.65	4.6077
2	13.743	0.5063	4391020.38	132570.58	95.3923

39: racemic

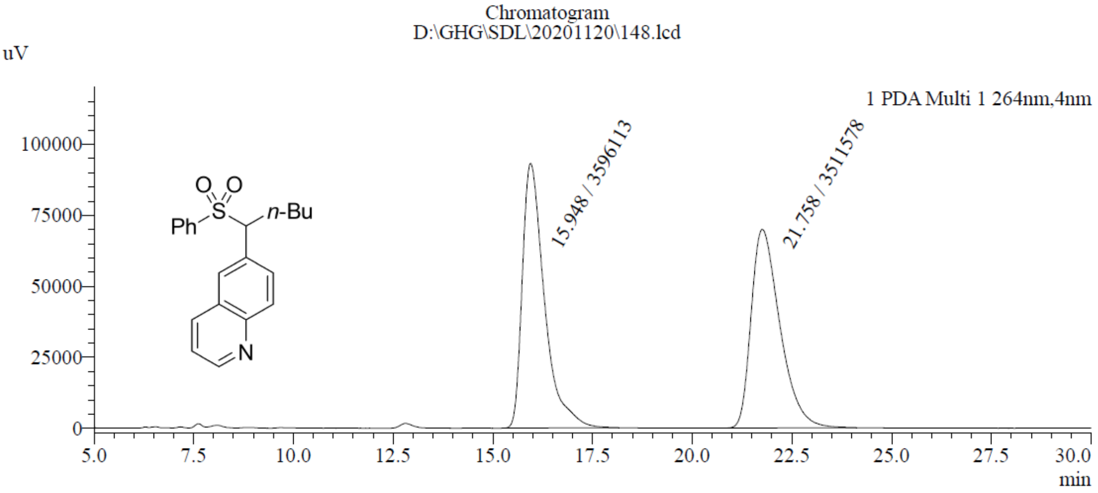

Peak Table

PDA Ch1 264nm

Peak#	Ret. Time [min]	Width [min]	Area [uV*s]	Height [uV]	Area %
1	15.948	0.5644	3596112.74	93211.29	50.5947
2	21.758	0.7638	3511578.18	69940.52	49.4053

39: enantioenriched, 90% ee

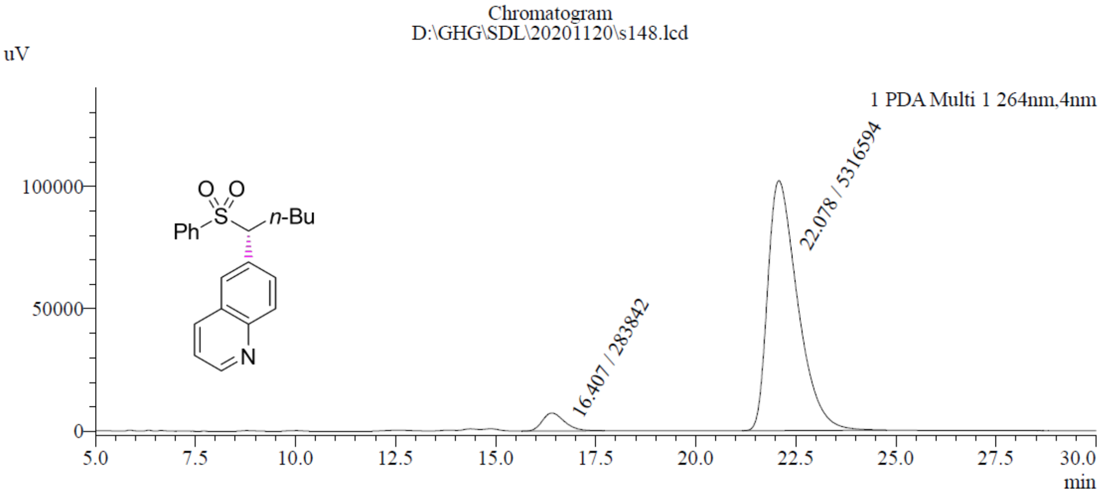

Peak Table

PDA Ch1 264nm

Peak#	Ret. Time [min]	Width [min]	Area [uV*s]	Height [uV]	Area %
1	16.407	0.5895	283842.06	7379.69	5.0682
2	22.078	0.7909	5316594.39	102008.76	94.9318

40: racemic

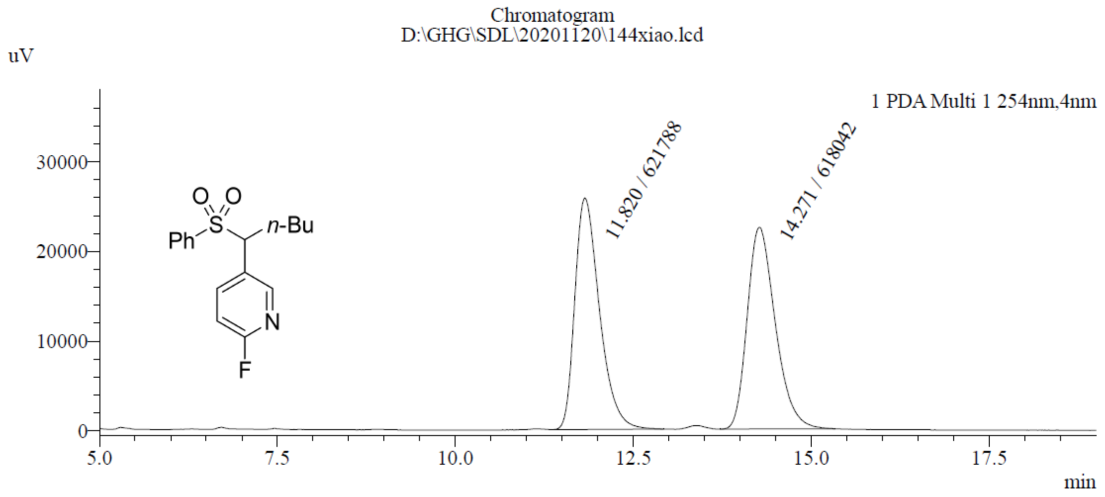

Peak Table

PDA Ch1 254nm

Peak#	Ret. Time [min]	Width [min]	Area [uV*s]	Height [uV]	Area %
1	11.820	0.3655	621787.53	25804.83	50.1510
2	14.271	0.4186	618042.28	22512.56	49.8490

40: enantioenriched, 93% ee

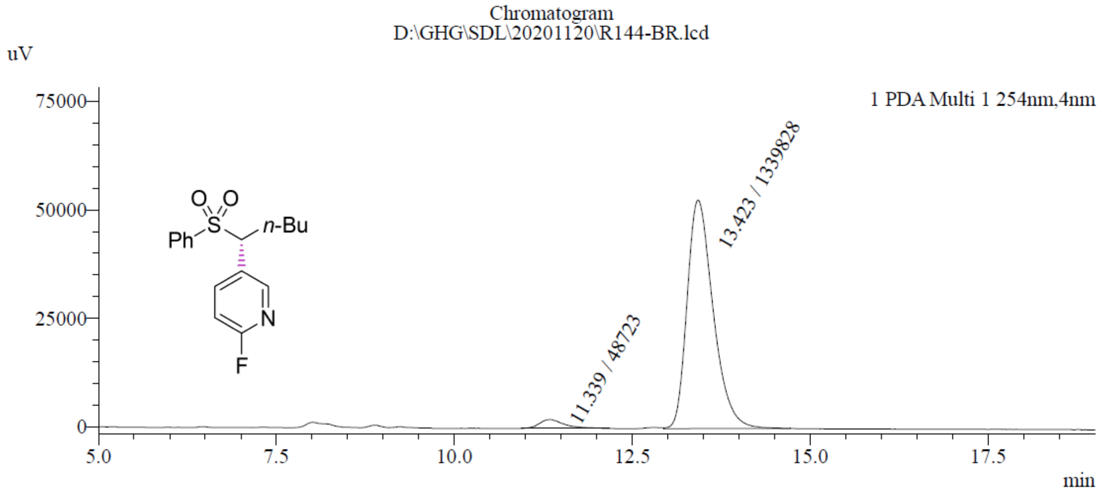

Peak Table

PDA Ch1 254nm

Peak#	Ret. Time [min]	Width [min]	Area [uV*s]	Height [uV]	Area %
1	11.339	0.3496	48723.19	2019.12	3.5089
2	13.423	0.3869	1339828.44	52560.57	96.4911

41: racemic

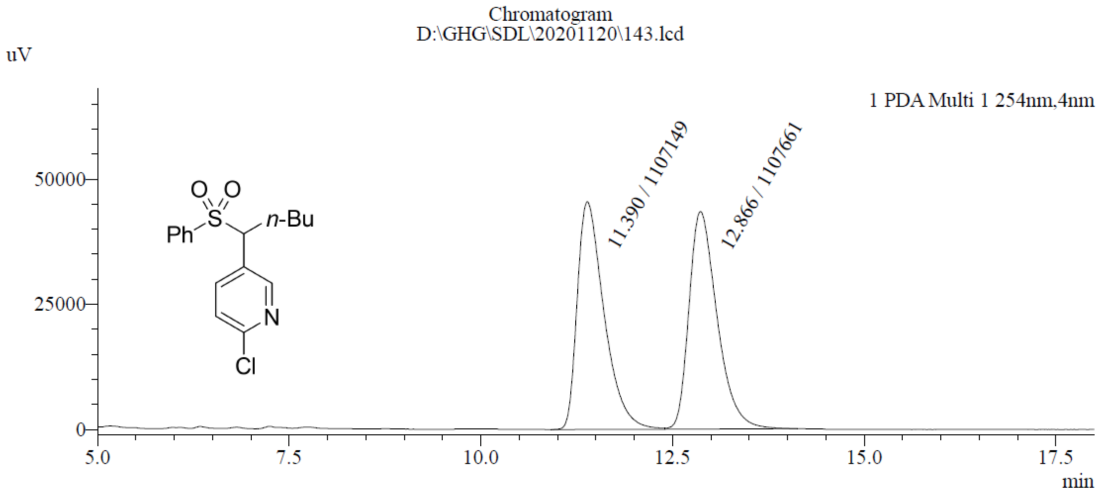

Peak Table

PDA Ch1 254nm

Peak#	Ret. Time [min]	Width [min]	Area [uV*s]	Height [uV]	Area %
1	11.390	0.3676	1107149.16	45450.31	49.9884
2	12.866	0.3851	1107661.15	43447.23	50.0116

41: enantioenriched, 90% ee

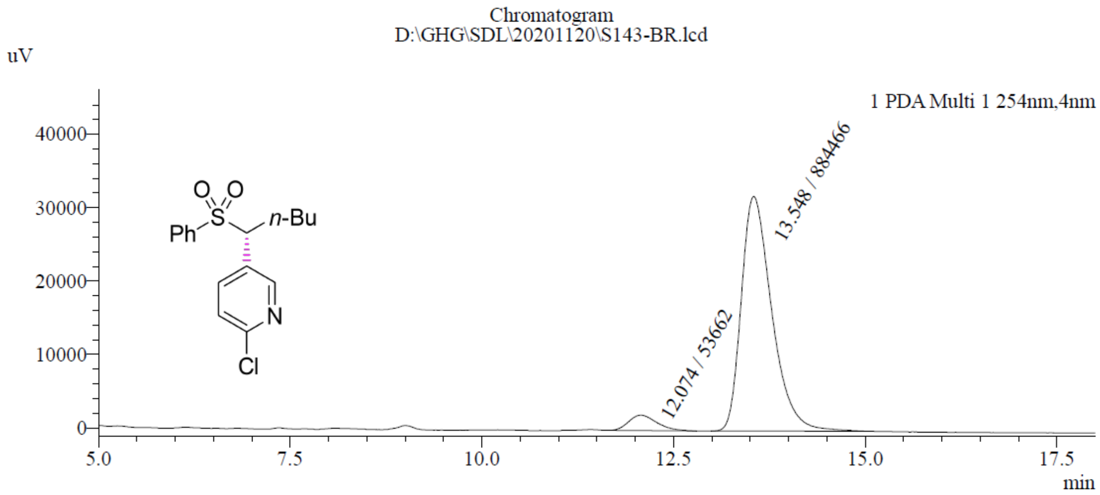

Peak Table

PDA Ch1 254nm

Peak#	Ret. Time [min]	Width [min]	Area [uV*s]	Height [uV]	Area %
1	12.074	0.3949	53662.08	2102.59	5.7201
2	13.548	0.4170	884466.18	31954.88	94.2799

42: racemic

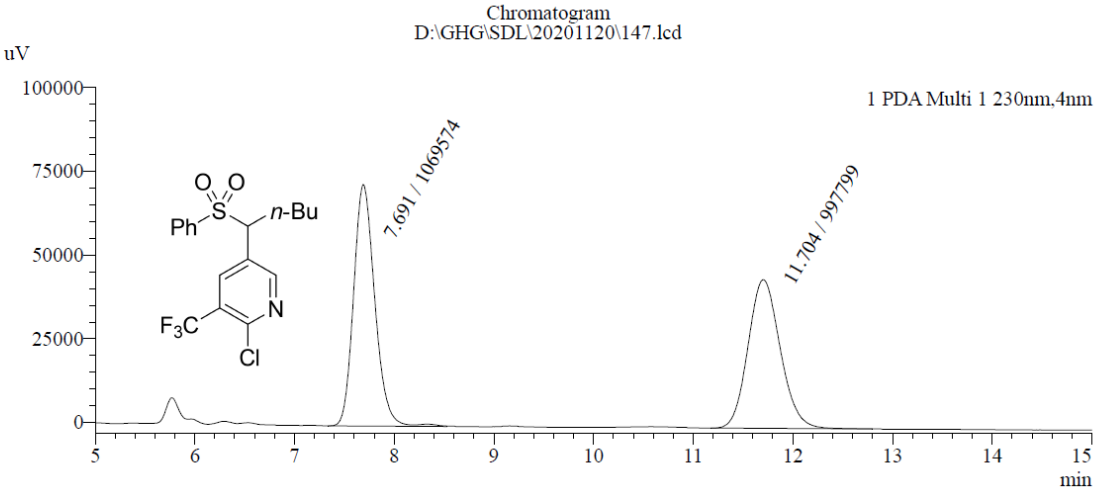

Peak Table

PDA Ch1 230nm

Peak#	Ret. Time [min]	Width [min]	Area [uV*s]	Height [uV]	Area %
1	7.691	0.2248	1069574.12	72181.19	51.7359
2	11.704	0.3455	997798.91	44447.66	48.2641

42: enantioenriched, 88% ee

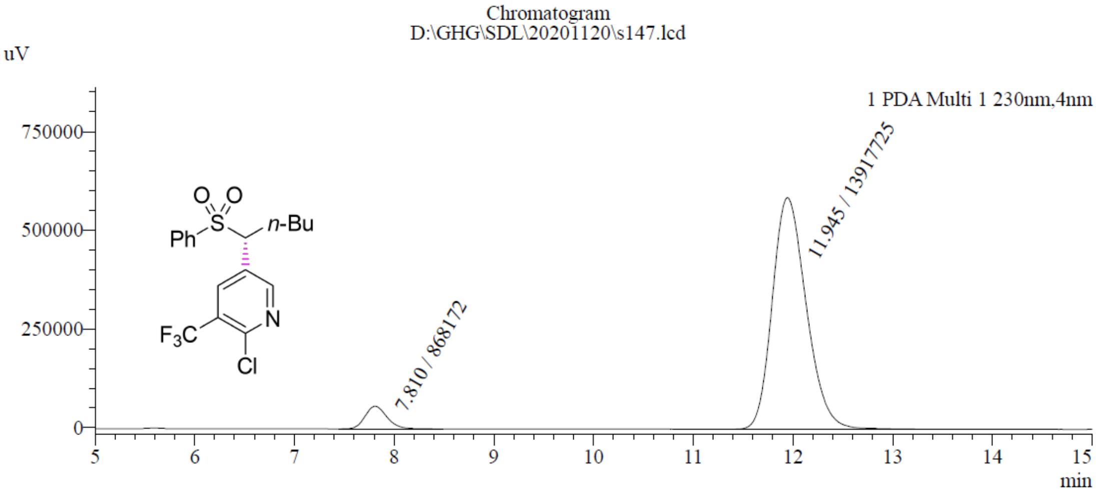

Peak Table

PDA Ch1 230nm

Peak#	Ret. Time [min]	Width [min]	Area [uV*s]	Height [uV]	Area %
1	7.810	0.2321	868171.99	57287.04	5.8716
2	11.945	0.3645	13917725.36	586639.42	94.1284

43: racemic

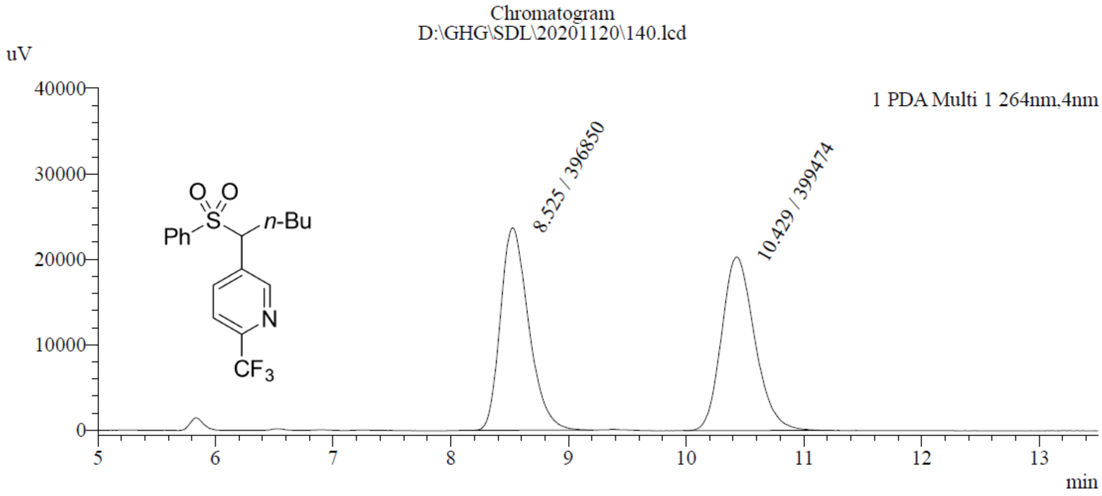

Peak Table

PDA Ch1 264nm

Peak#	Ret. Time [min]	Width [min]	Area [uV*s]	Height [uV]	Area %
1	8.525	0.2558	396850.27	23707.75	49.8353
2	10.429	0.3007	399474.12	20313.91	50.1647

43: enantioenriched, 93% ee

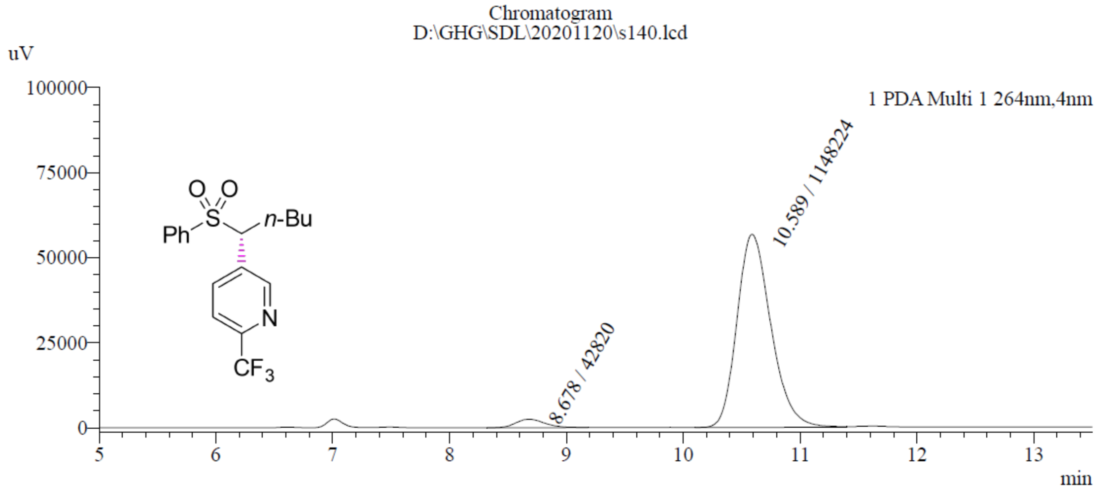

Peak Table

PDA Ch1 264nm

Peak#	Ret. Time [min]	Width [min]	Area [uV*s]	Height [uV]	Area %
1	8.678	0.2638	42819.99	2497.03	3.5952
2	10.589	0.3089	1148223.76	56765.33	96.4048

44: racemic

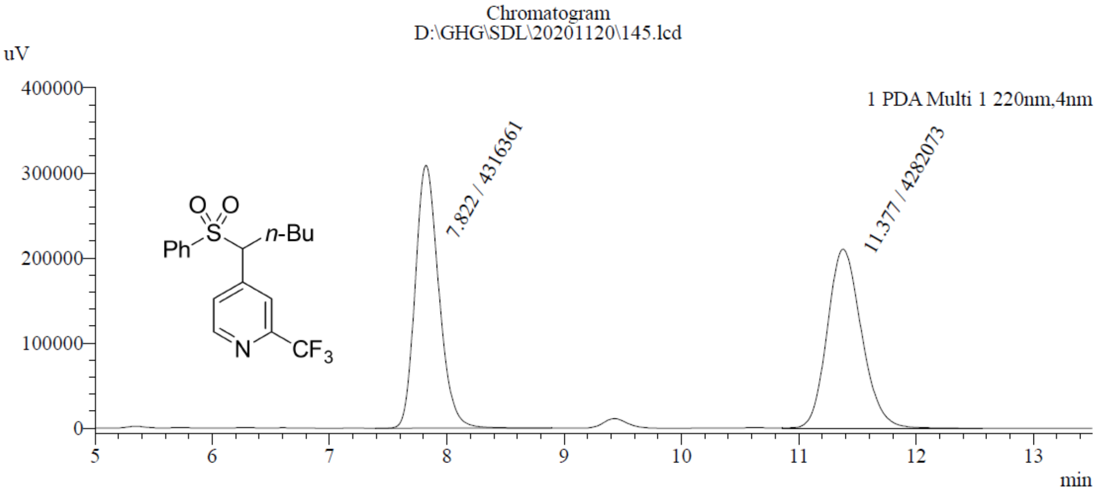

Peak Table

PDA Ch1 220nm

Peak#	Ret. Time [min]	Width [min]	Area [uV*s]	Height [uV]	Area %
1	7.822	0.2129	4316361.30	308962.21	50.1994
2	11.377	0.3120	4282073.40	210555.44	49.8006

44: enantioenriched, 92% ee

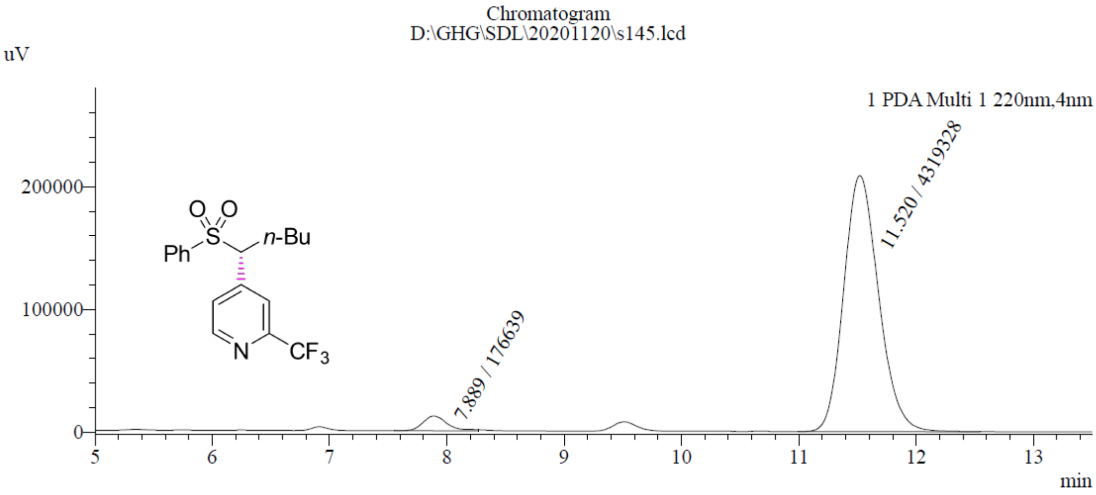

Peak Table

PDA Ch1 220nm

Peak#	Ret. Time [min]	Width [min]	Area [uV*s]	Height [uV]	Area %
1	7.889	0.2149	176638.55	12102.61	3.9288
2	11.520	0.3181	4319327.97	208682.77	96.0712

45: racemic

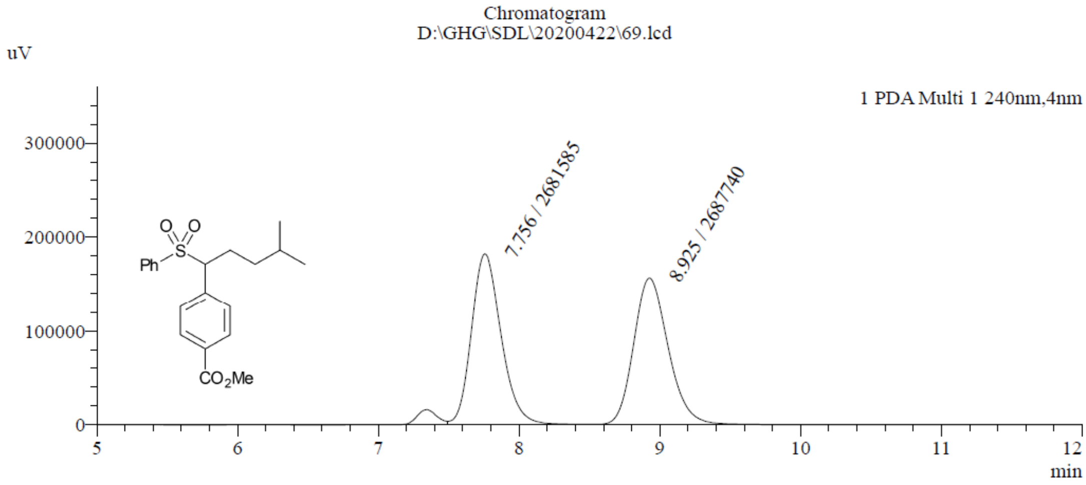

Peak Table

PDA Ch1 240nm

Peak#	Ret. Time [min]	Width [min]	Area [uV*s]	Height [uV]	Area %
1	7.756	0.2239	2681585.13	182088.89	49.9427
2	8.925	0.2612	2687740.43	156366.42	50.0573

45: enantioenriched, 92% ee

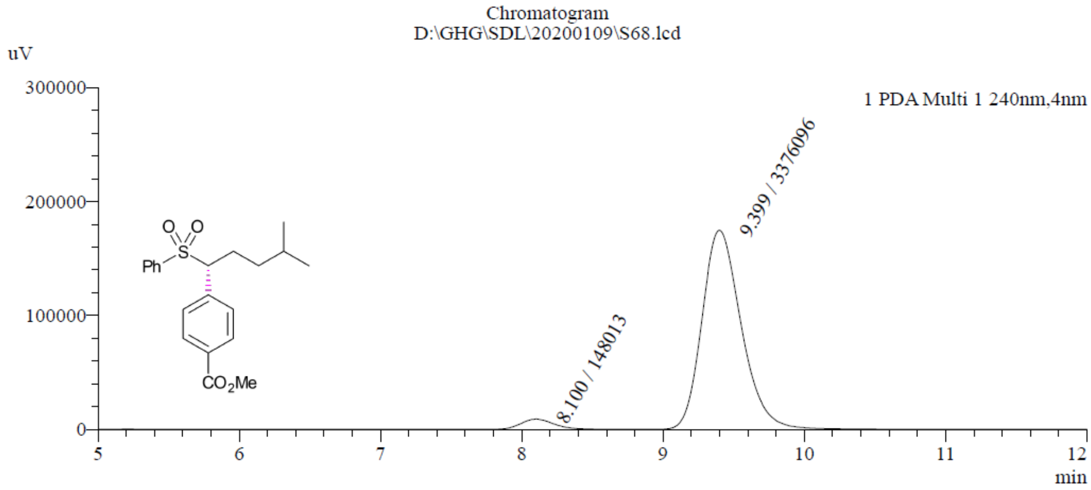

Peak Table

PDA Ch1 240nm

Peak#	Ret. Time [min]	Width [min]	Area [uV*s]	Height [uV]	Area %
1	8.100	0.2516	148013.13	8980.86	4.2000
2	9.399	0.2937	3376095.54	174869.49	95.8000

46: racemic

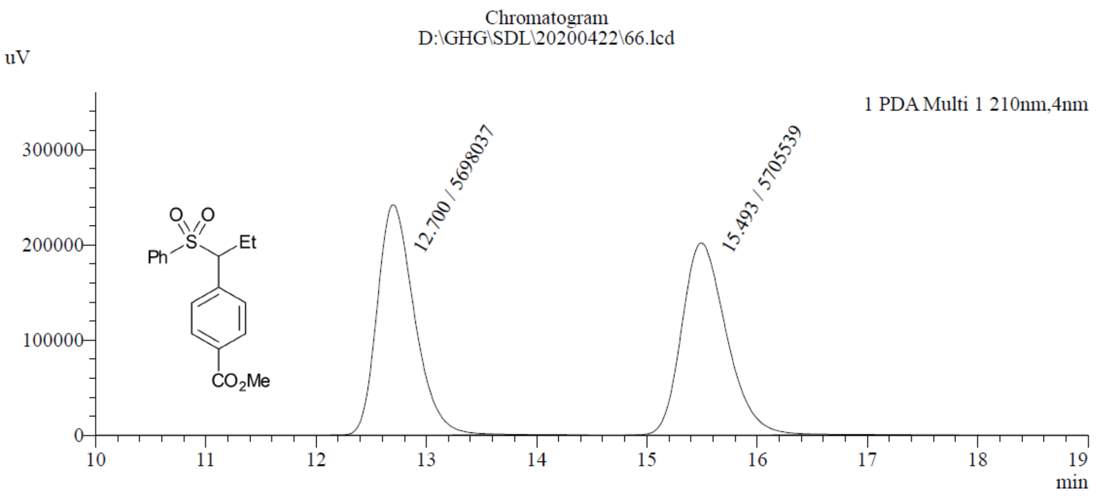

Peak Table

PDA Ch1 210nm

Peak#	Ret. Time [min]	Width [min]	Area [uV*s]	Height [uV]	Area %
1	12.700	0.3581	5698036.93	241953.41	49.9671
2	15.493	0.4317	5705539.00	201622.27	50.0329

46: enantioenriched, 90% ee

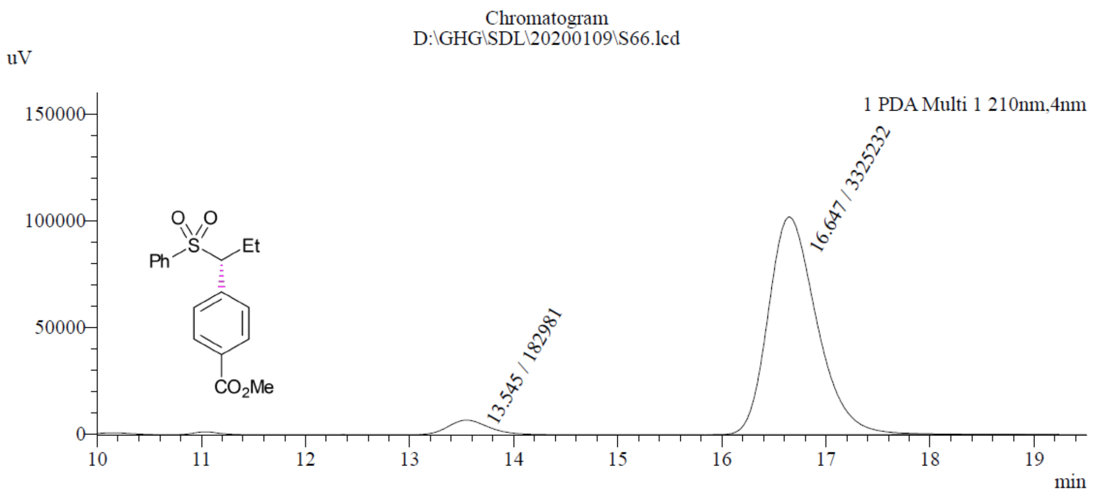

Peak Table

PDA Ch1 210nm

Peak#	Ret. Time [min]	Width [min]	Area [uV*s]	Height [uV]	Area %
1	13.545	0.4124	182981.34	6804.53	5.2158
2	16.647	0.4940	3325231.91	102074.83	94.7842

47: racemic

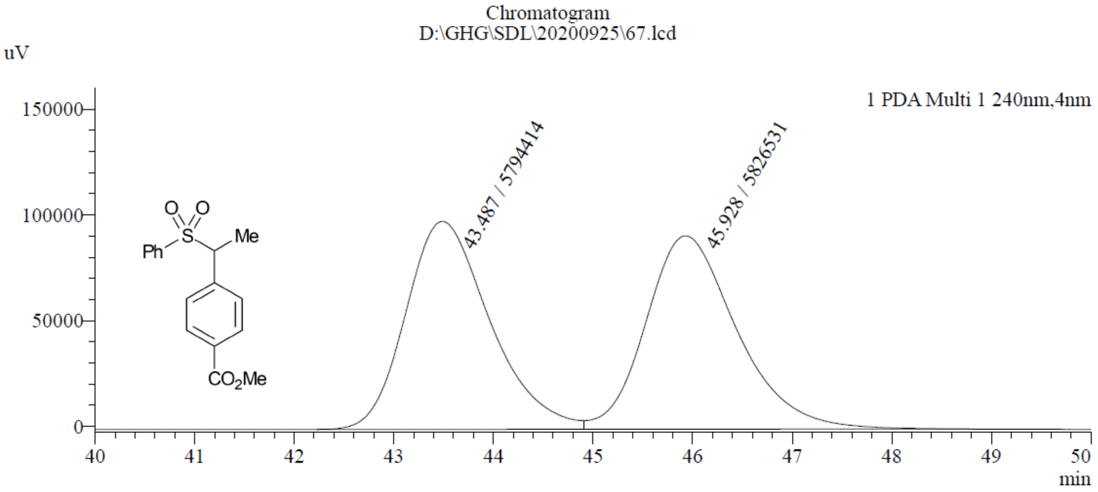

Peak Table

PDA Ch1 240nm

Peak#	Ret. Time [min]	Width [min]	Area [uV*s]	Height [uV]	Area %
1	43.487	0.8965	5794414.42	98339.98	49.8618
2	45.928	0.9572	5826530.86	91417.85	50.1382

47: enantioenriched, 91% ee

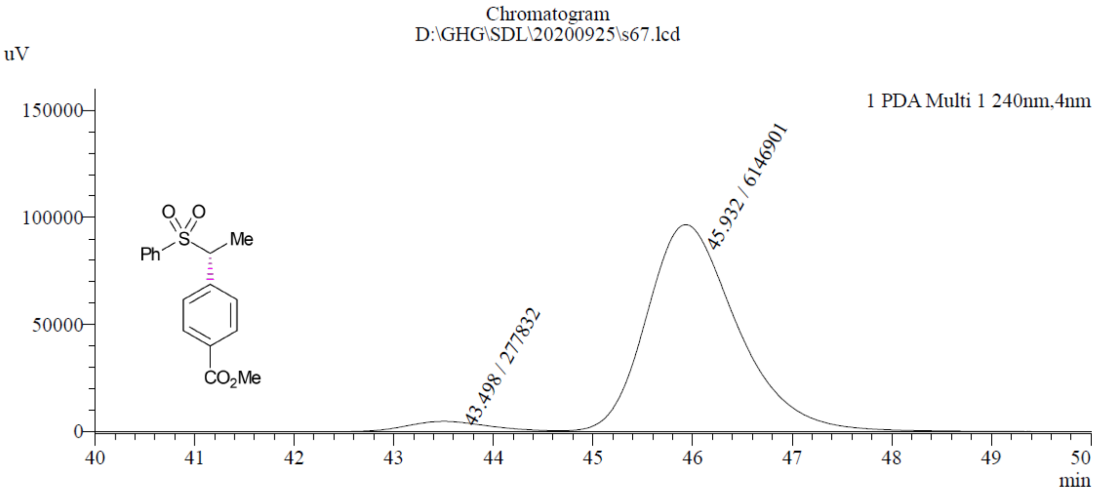

Peak Table

PDA Ch1 240nm

Peak#	Ret. Time [min]	Width [min]	Area [uV*s]	Height [uV]	Area %
1	43.498	0.8921	277832.03	4796.24	4.3244
2	45.932	0.9557	6146901.17	96675.47	95.6756

48: racemic

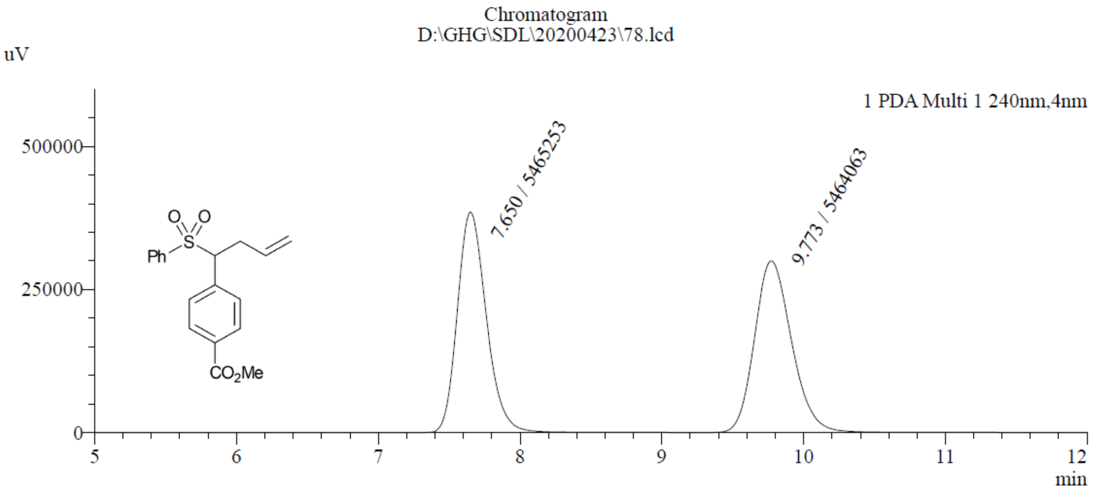

Peak Table

PDA Ch1 240nm

Peak#	Ret. Time [min]	Width [min]	Area [uV*s]	Height [uV]	Area %
1	7.650	0.2160	5465253.34	385550.02	50.0054
2	9.773	0.2788	5464062.54	300081.37	49.9946

48: enantioenriched, 87% ee

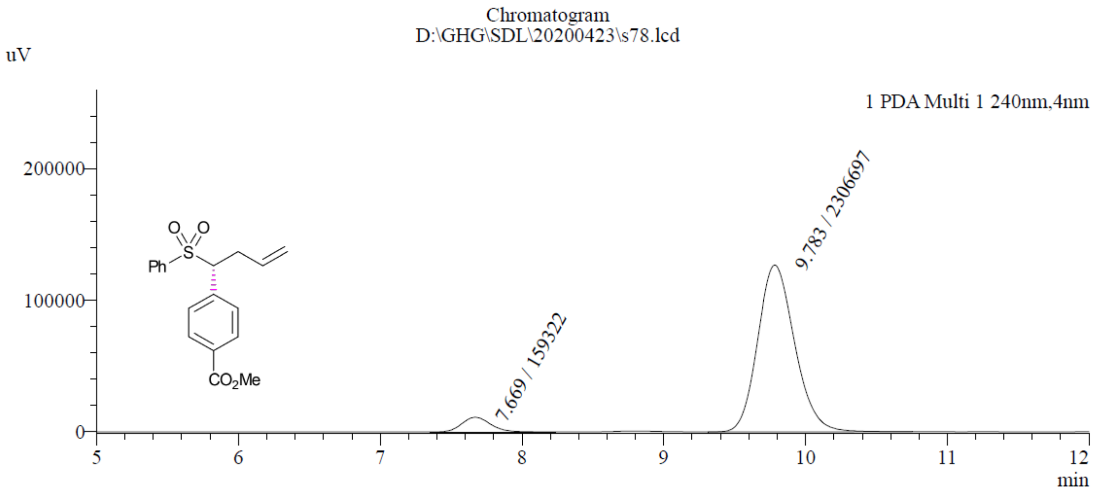

Peak Table

PDA Ch1 240nm

Peak#	Ret. Time [min]	Width [min]	Area [uV*s]	Height [uV]	Area %
1	7.669	0.2167	159321.92	11255.74	6.4607
2	9.783	0.2782	2306696.60	126998.45	93.5393

49: racemic

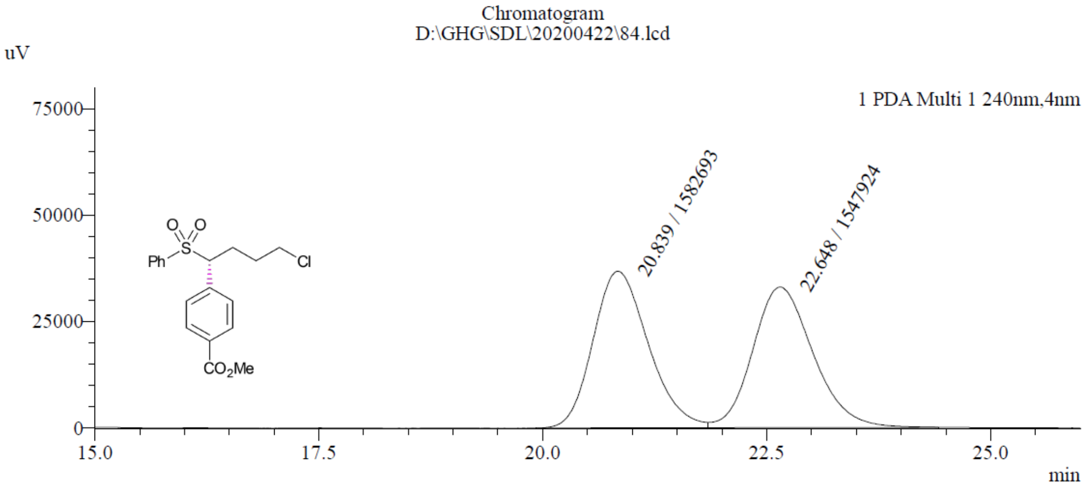

Peak Table

PDA Ch1 240nm

Peak#	Ret. Time [min]	Width [min]	Area [uV*s]	Height [uV]	Area %
1	20.839	0.6624	1582693.29	36816.78	50.5553
2	22.648	0.7152	1547924.14	33034.01	49.4447

49: enantioenriched, 90% ee

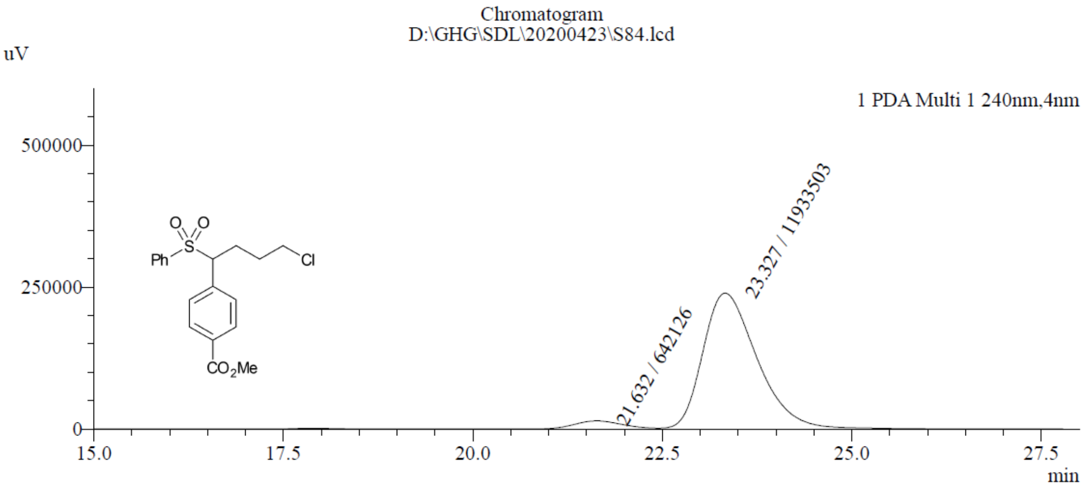

Peak Table

PDA Ch1 240nm

Peak#	Ret. Time [min]	Width [min]	Area [uV*s]	Height [uV]	Area %
1	21.632	0.6973	642126.03	14391.48	5.1061
2	23.327	0.7585	11933503.43	239244.48	94.8939

50: racemic

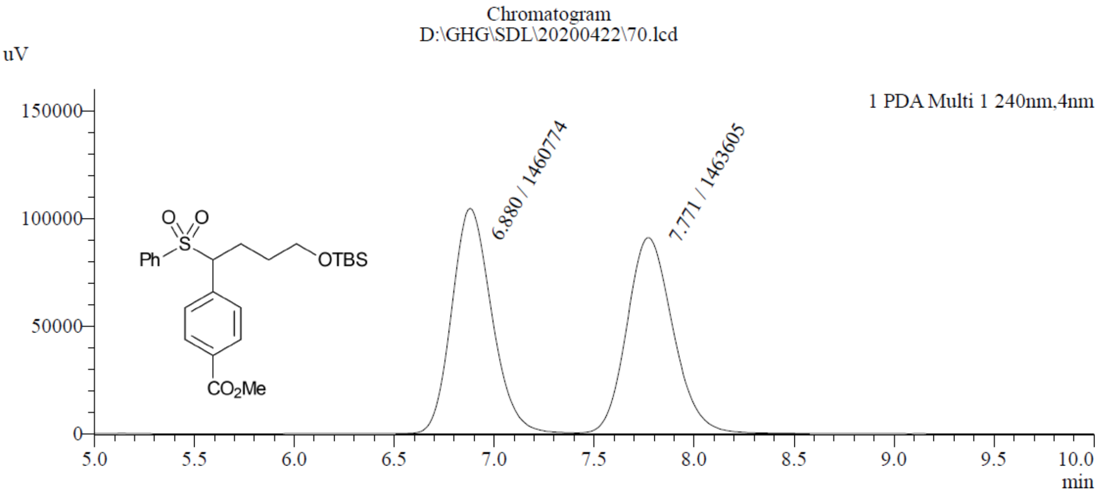

Peak Table

PDA Ch1 240nm

Peak#	Ret. Time [min]	Width [min]	Area [uV*s]	Height [uV]	Area %
1	6.880	0.2139	1460774.10	104627.33	49.9516
2	7.771	0.2463	1463604.87	90980.13	50.0484

50: enantioenriched, 92% ee

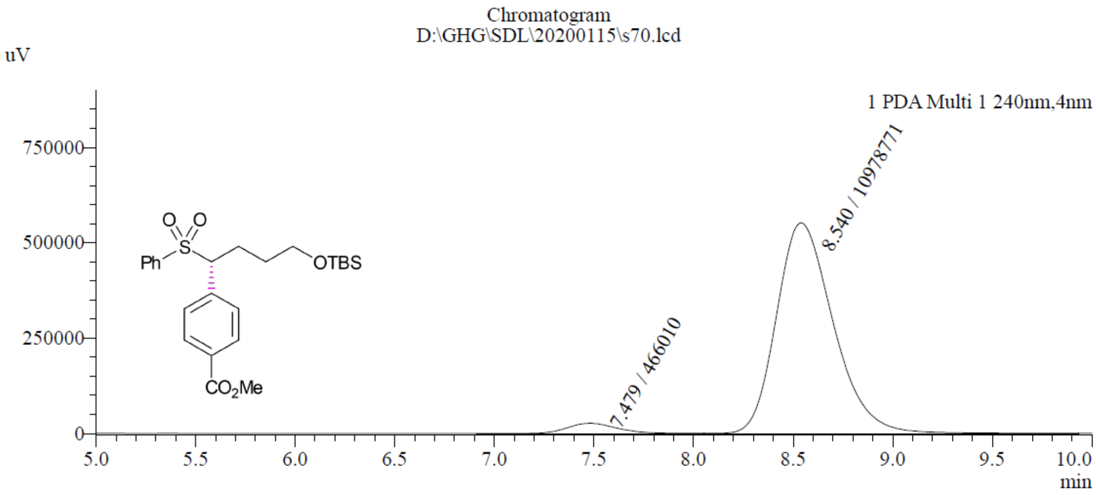

Peak Table

PDA Ch1 240nm

Peak#	Ret. Time [min]	Width [min]	Area [uV*s]	Height [uV]	Area %
1	7.479	0.2644	466010.45	26764.75	4.0718
2	8.540	0.3027	10978770.96	552092.66	95.9282

51: racemic

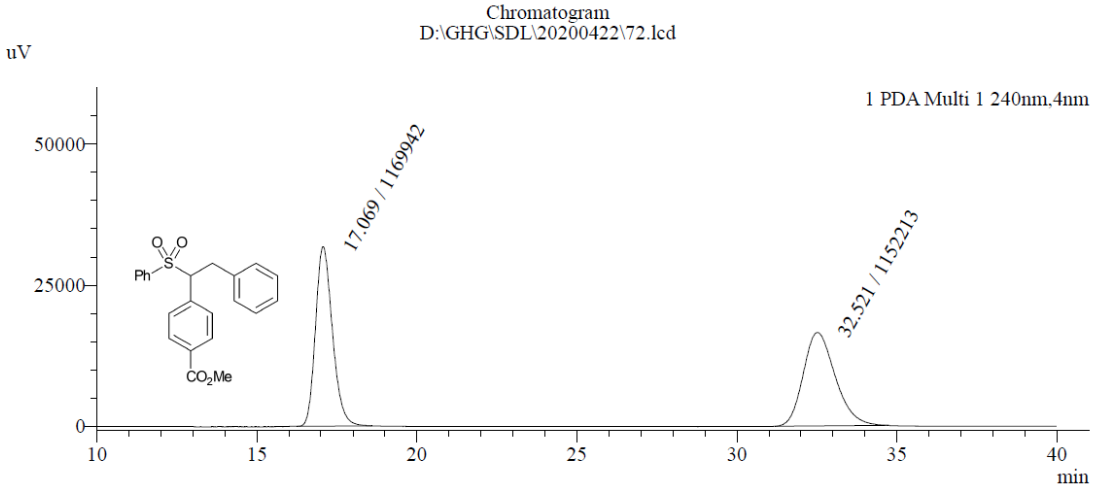

Peak Table

PDA Ch1 240nm

Peak#	Ret. Time [min]	Width [min]	Area [uV*s]	Height [uV]	Area %
1	17.069	0.5625	1169942.22	31784.07	50.3817
2	32.521	1.0667	1152213.42	16557.54	49.6183

51: enantioenriched, 93% ee

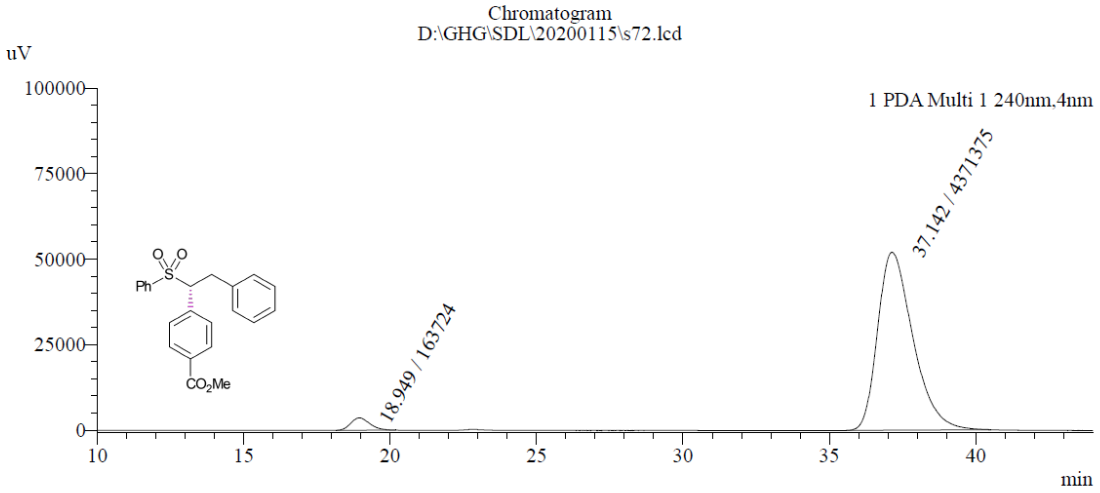

Peak Table

PDA Ch1 240nm

Peak#	Ret. Time [min]	Width [min]	Area [uV*s]	Height [uV]	Area %
1	18.949	0.6952	163723.81	3635.39	3.6101
2	37.142	1.2769	4371375.02	52048.54	96.3899

52: racemic

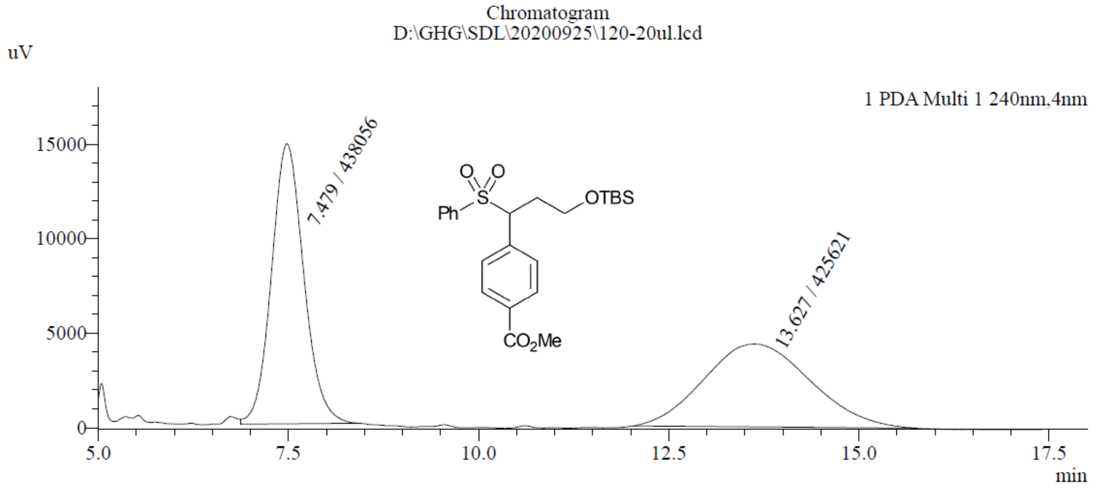

Peak Table

PDA Ch1 240nm

Peak#	Ret. Time [min]	Width [min]	Area [uV*s]	Height [uV]	Area %
1	7.479	0.4490	438056.39	14811.28	50.7199
2	13.627	1.5411	425621.33	4380.76	49.2801

52: enantioenriched, 94% ee

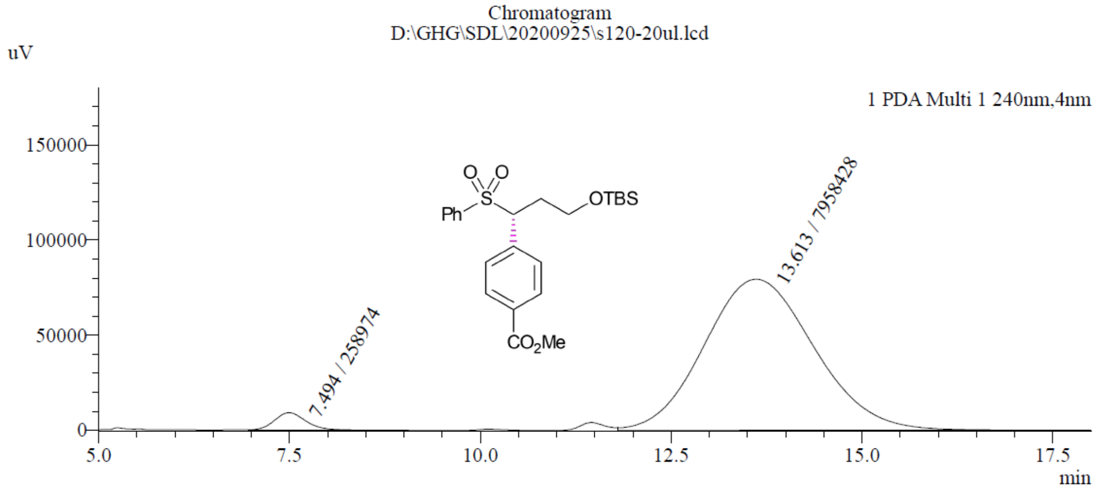

Peak Table

PDA Ch1 240nm

Peak#	Ret. Time [min]	Width [min]	Area [uV*s]	Height [uV]	Area %
1	7.494	0.4344	258974.25	9130.56	3.1515
2	13.613	1.5636	7958427.79	79345.43	96.8485

53: racemic

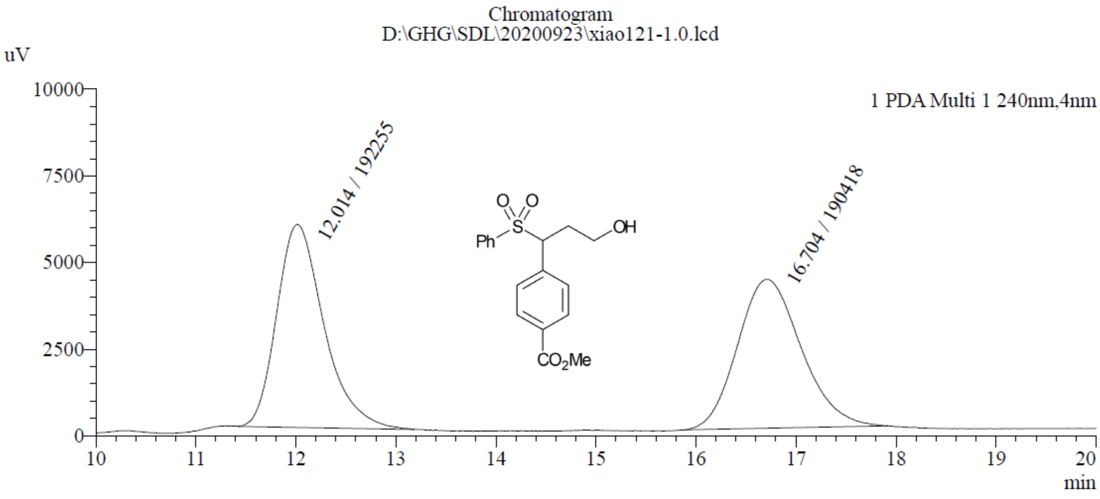

Peak Table

PDA Ch1 240nm

Peak#	Ret. Time [min]	Width [min]	Area [uV*s]	Height [uV]	Area %
1	12.014	0.4930	192255.33	5860.37	50.2400
2	16.704	0.6848	190418.42	4297.26	49.7600

53: enantioenriched, 90% ee

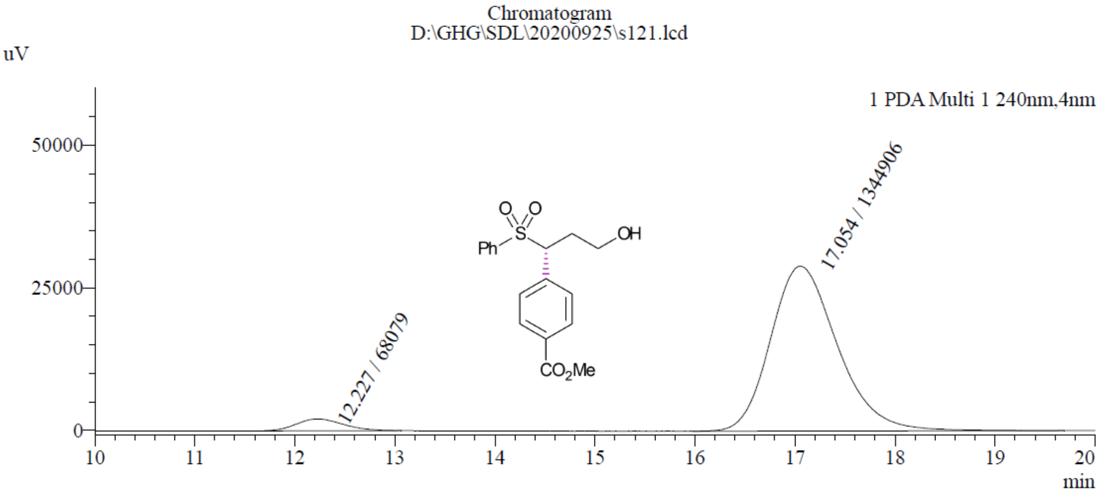

Peak Table

PDA Ch1 240nm

Peak#	Ret. Time [min]	Width [min]	Area [uV*s]	Height [uV]	Area %
1	12.227	0.5056	68078.85	2088.65	4.8181
2	17.054	0.7063	1344906.49	28925.98	95.1819

54: racemic

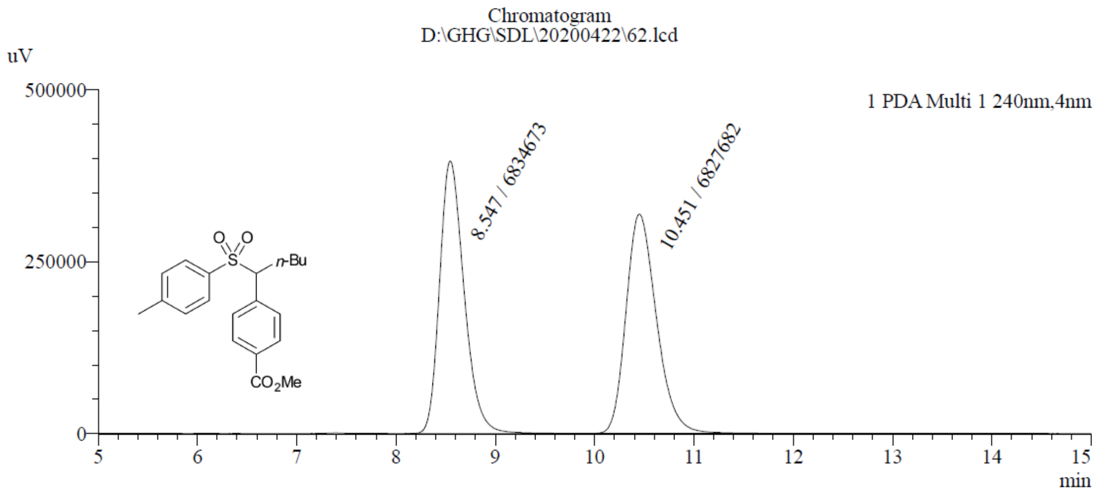

Peak Table

PDA Ch1 240nm

Peak#	Ret. Time [min]	Width [min]	Area [uV*s]	Height [uV]	Area %
1	8.547	0.2614	6834672.93	396957.52	50.0256
2	10.451	0.3257	6827681.72	319481.89	49.9744

54: enantioenriched, 90% ee

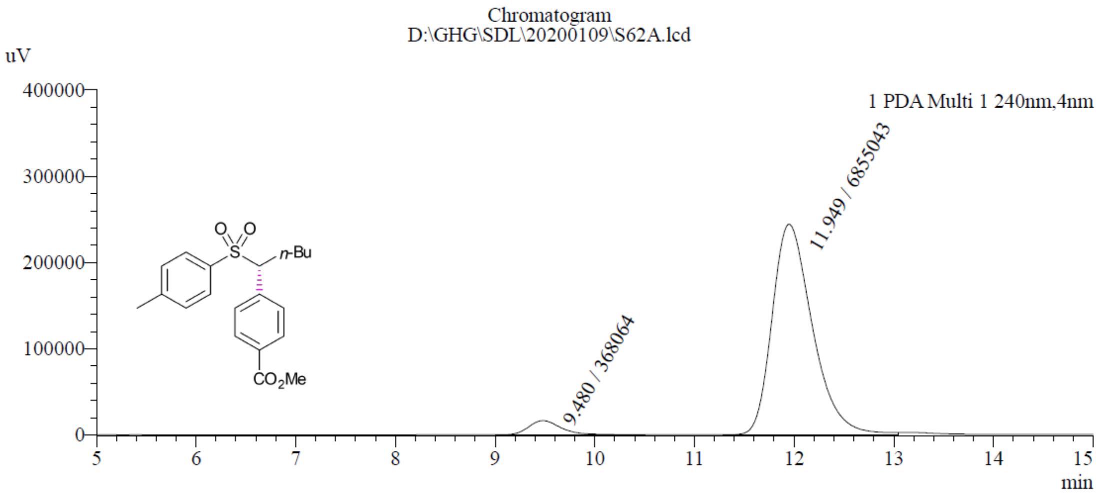

Peak Table

PDA Ch1 240nm

Peak#	Ret. Time [min]	Width [min]	Area [uV*s]	Height [uV]	Area %
1	9.480	0.3367	368063.58	16355.52	5.0956
2	11.949	0.4247	6855043.31	244095.20	94.9044

55: racemic

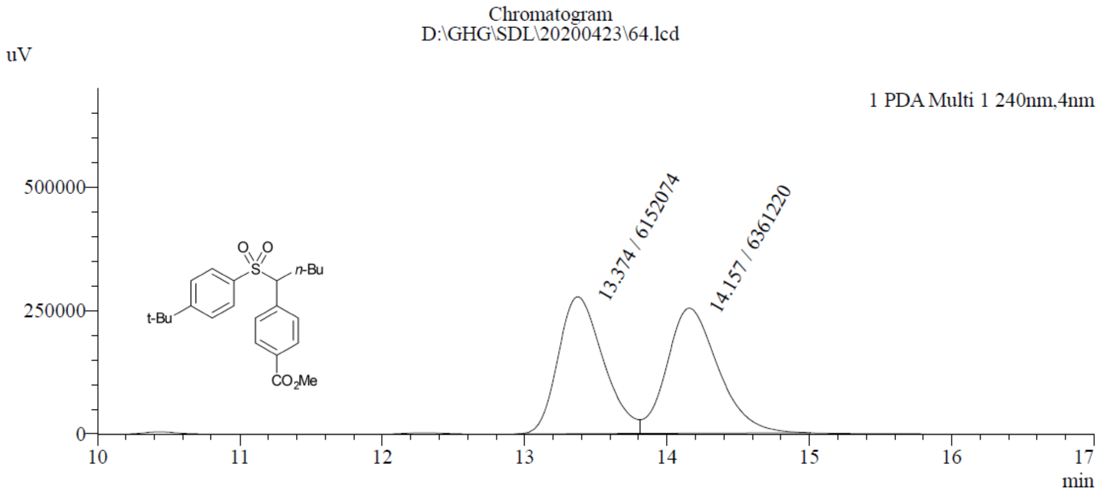

Peak Table

PDA Ch1 240nm

Peak#	Ret. Time [min]	Width [min]	Area [uV*s]	Height [uV]	Area %
1	13.374	0.3421	6152073.62	278259.31	49.1643
2	14.157	0.3797	6361219.66	254204.09	50.8357

55: enantioenriched, 90% ee

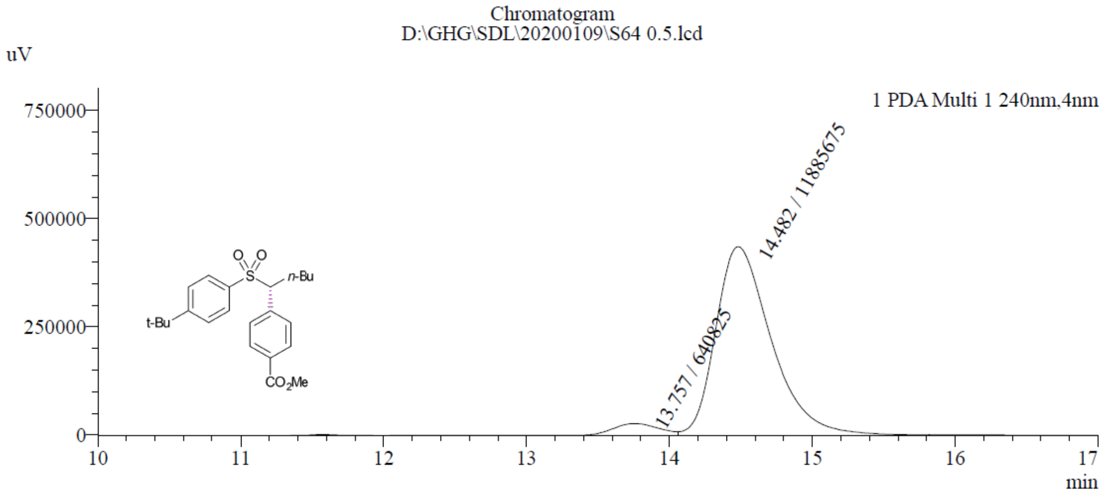

Peak Table

PDA Ch1 240nm

Peak#	Ret. Time [min]	Width [min]	Area [uV*s]	Height [uV]	Area %
1	13.757	0.3709	640824.77	28100.94	5.1158
2	14.482	0.4060	11885675.30	436760.00	94.8842

56: racemic

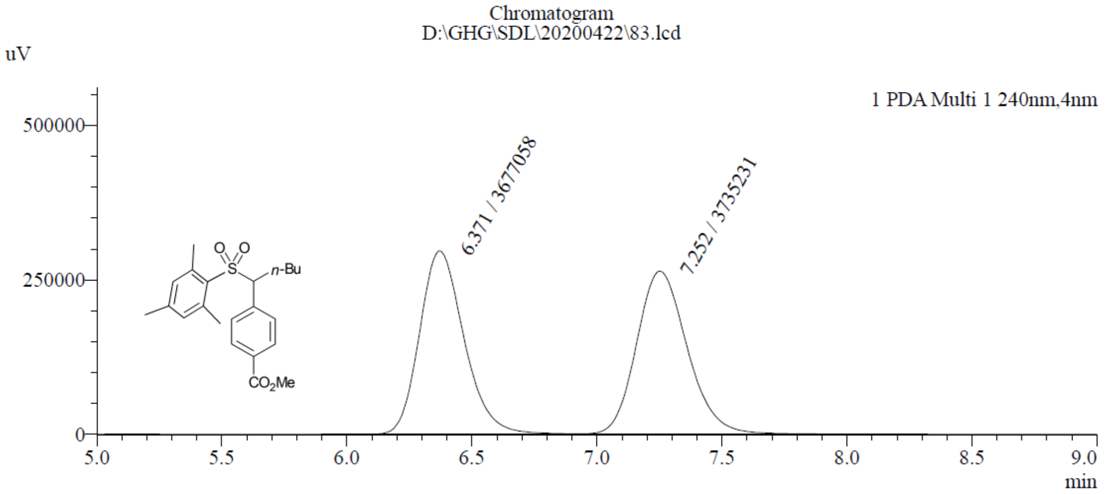

Peak Table

PDA Ch1 240nm

Peak#	Ret. Time [min]	Width [min]	Area [uV*s]	Height [uV]	Area %
1	6.371	0.1884	3677058.10	296757.65	49.6076
2	7.252	0.2158	3735230.79	264232.87	50.3924

56: enantioenriched, 95% ee

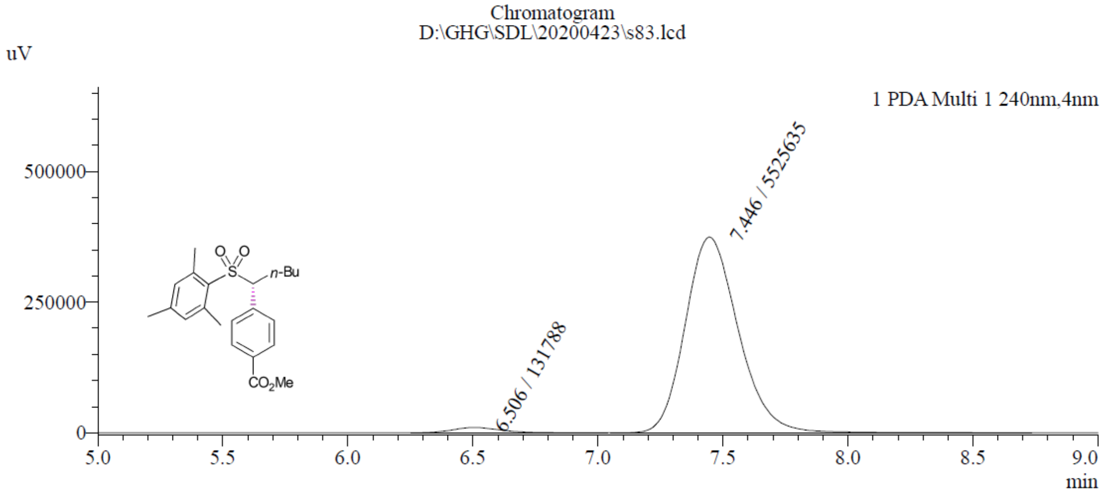

Peak Table

PDA Ch1 240nm

Peak#	Ret. Time [min]	Width [min]	Area [uV*s]	Height [uV]	Area %
1	6.506	0.1989	131788.34	10067.90	2.3295
2	7.446	0.2250	5525635.22	374672.53	97.6705

57: racemic

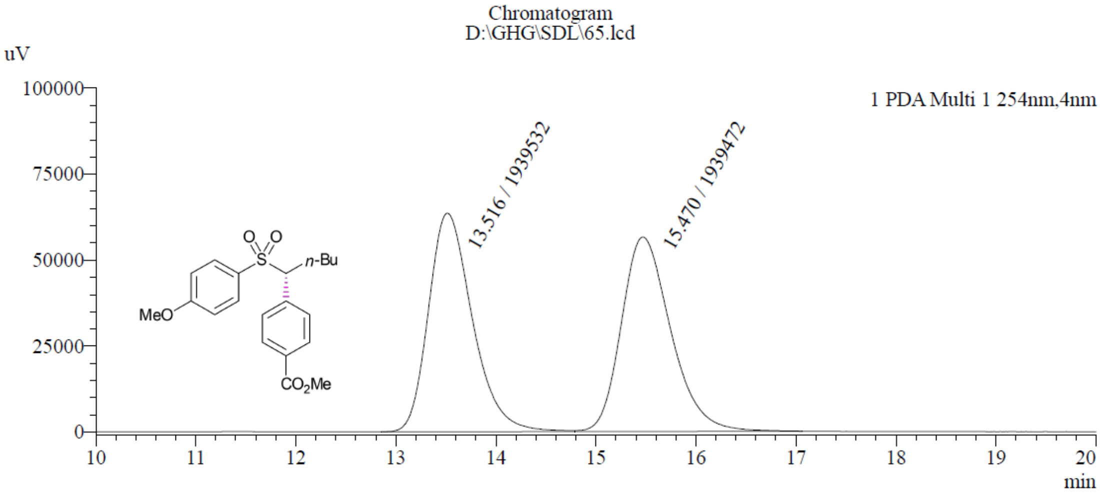

Peak Table

PDA Ch1 254nm

Peak#	Ret. Time [min]	Width [min]	Area [uV*s]	Height [uV]	Area %
1	13.516	0.4619	1939531.86	63619.45	50.0008
2	15.470	0.5216	1939472.34	56551.24	49.9992

57: enantioenriched, 90% ee

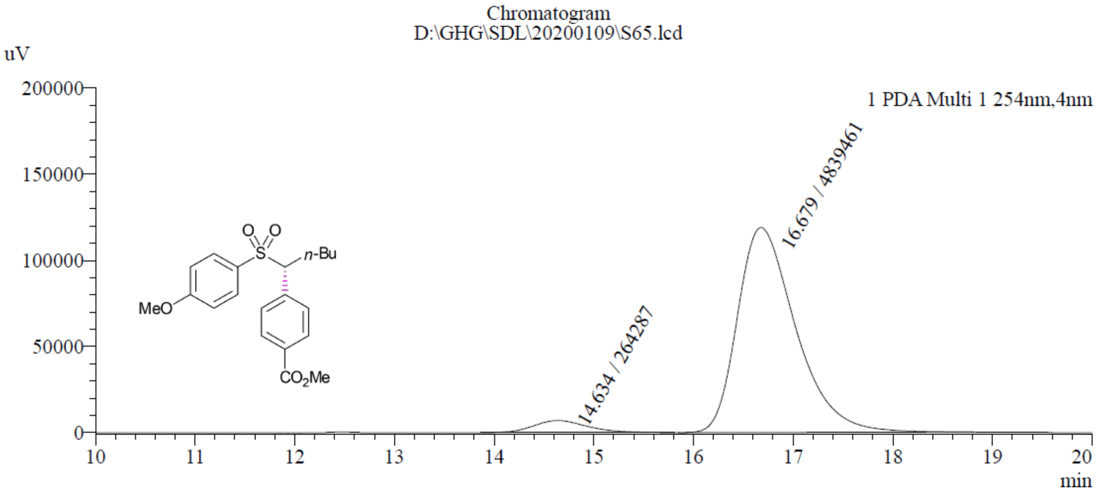

Peak Table

PDA Ch1 254nm

Peak#	Ret. Time [min]	Width [min]	Area [uV*s]	Height [uV]	Area %
1	14.634	0.5713	264286.82	7012.01	5.1783
2	16.679	0.6141	4839460.79	118997.58	94.8217

58: racemic

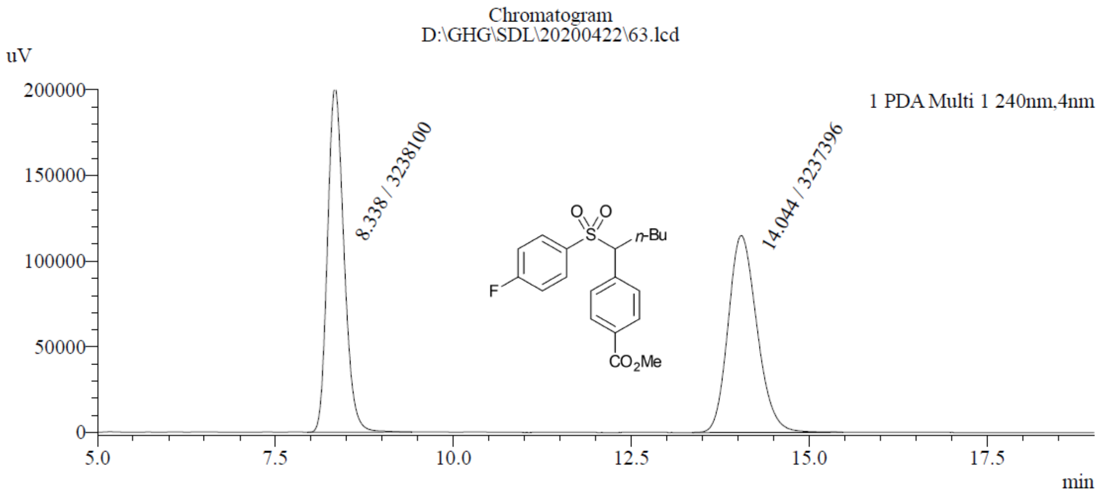

Peak Table

PDA Ch1 240nm

Peak#	Ret. Time [min]	Width [min]	Area [uV*s]	Height [uV]	Area %
1	8.338	0.2448	3238100.39	201596.87	50.0054
2	14.044	0.4327	3237395.73	114935.04	49.9946

58: enantioenriched, 90% ee

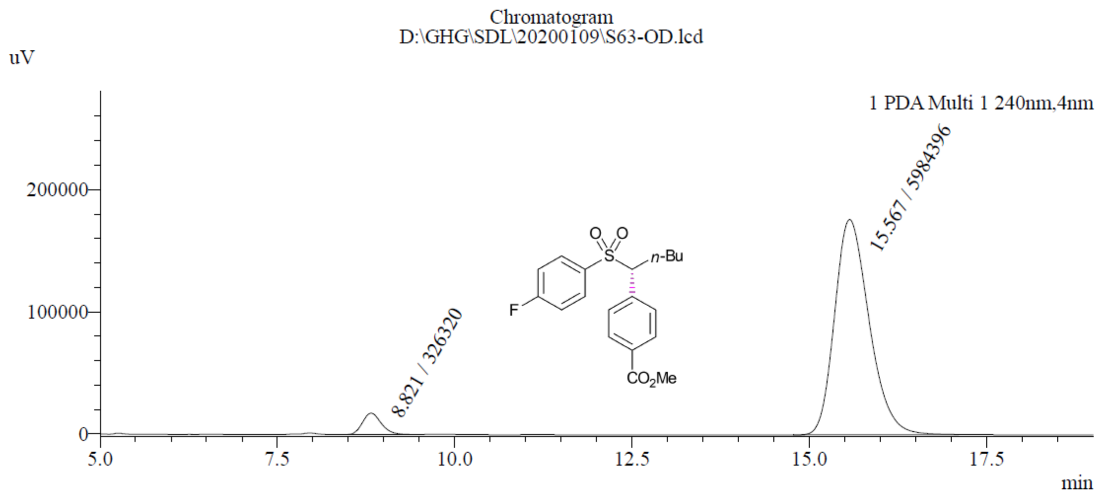

Peak Table

PDA Ch1 240nm

Peak#	Ret. Time [min]	Width [min]	Area [uV*s]	Height [uV]	Area %
1	8.821	0.2831	326320.42	17593.36	5.1709
2	15.567	0.5196	5984396.42	176123.24	94.8291

59: racemic

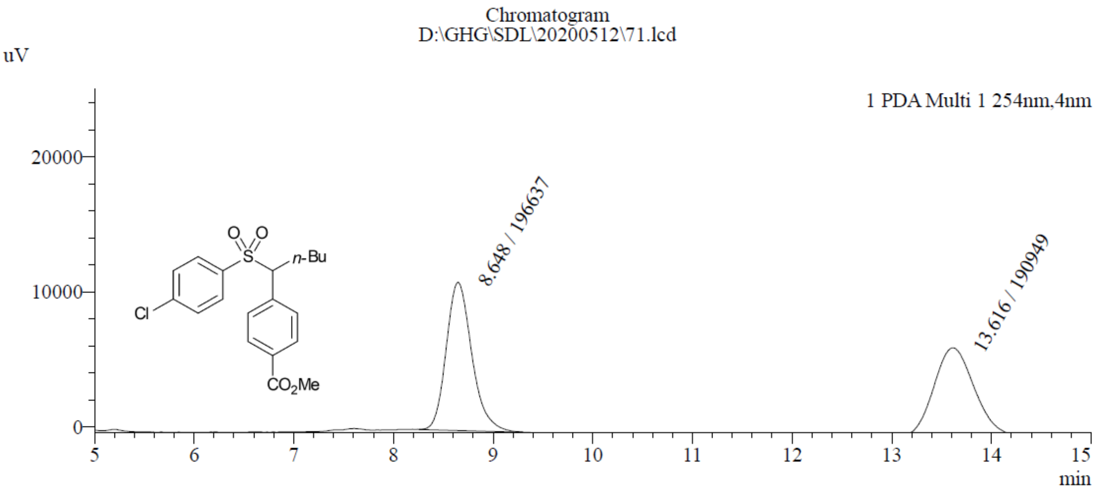

Peak Table

PDA Ch1 254nm

Peak#	Ret. Time [min]	Width [min]	Area [uV*s]	Height [uV]	Area %
1	8.648	0.2703	196636.51	10981.41	50.7337
2	13.616	0.4446	190948.79	6626.12	49.2663

59: enantioenriched, 90% ee

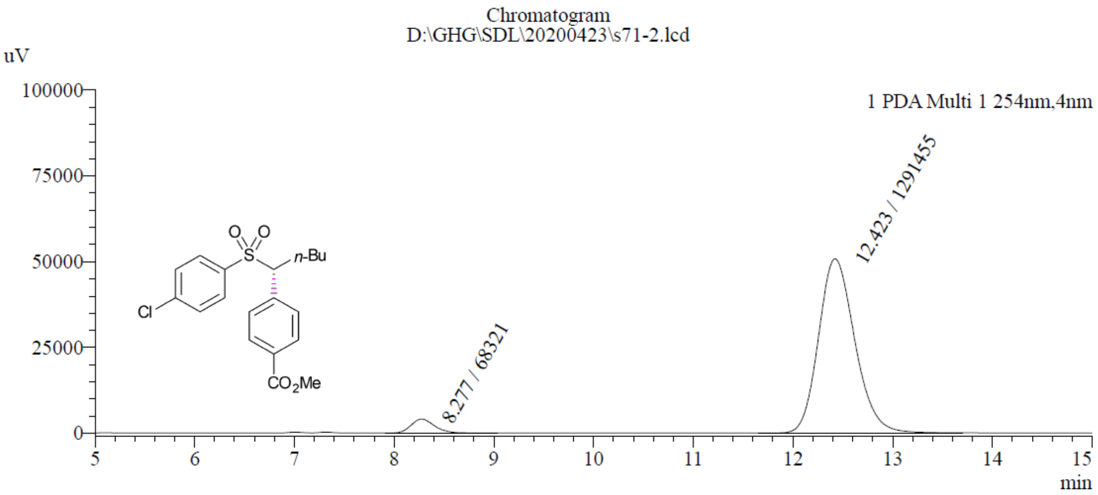

Peak Table

PDA Ch1 254nm

Peak#	Ret. Time [min]	Width [min]	Area [uV*s]	Height [uV]	Area %
1	8.277	0.2505	68320.74	4123.51	5.0244
2	12.423	0.3880	1291455.48	50831.61	94.9756

60: racemic

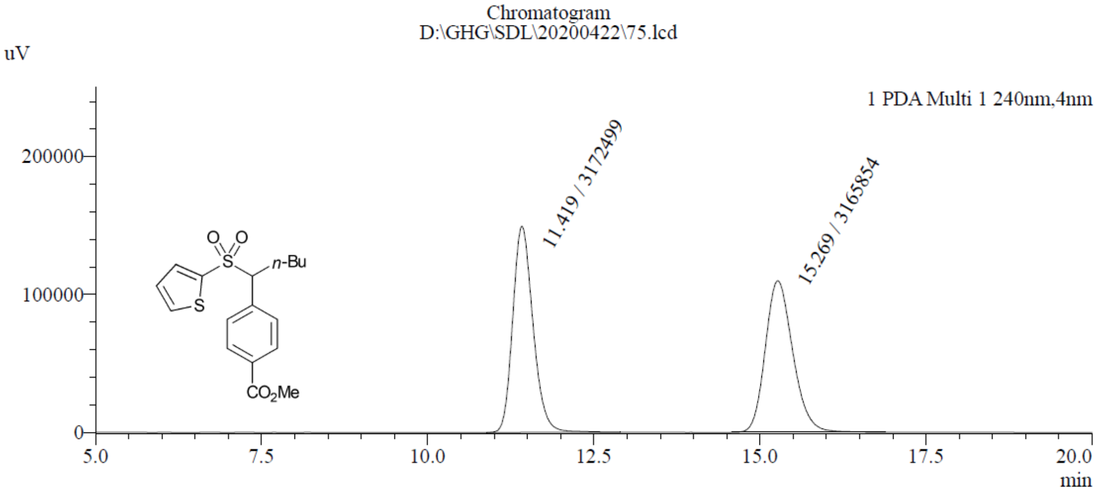

Peak Table

PDA Ch1 240nm

Peak#	Ret. Time [min]	Width [min]	Area [uV*s]	Height [uV]	Area %
1	11.419	0.3233	3172498.90	149352.61	50.0524
2	15.269	0.4412	3165854.02	109792.26	49.9476

60: enantioenriched, 88% ee

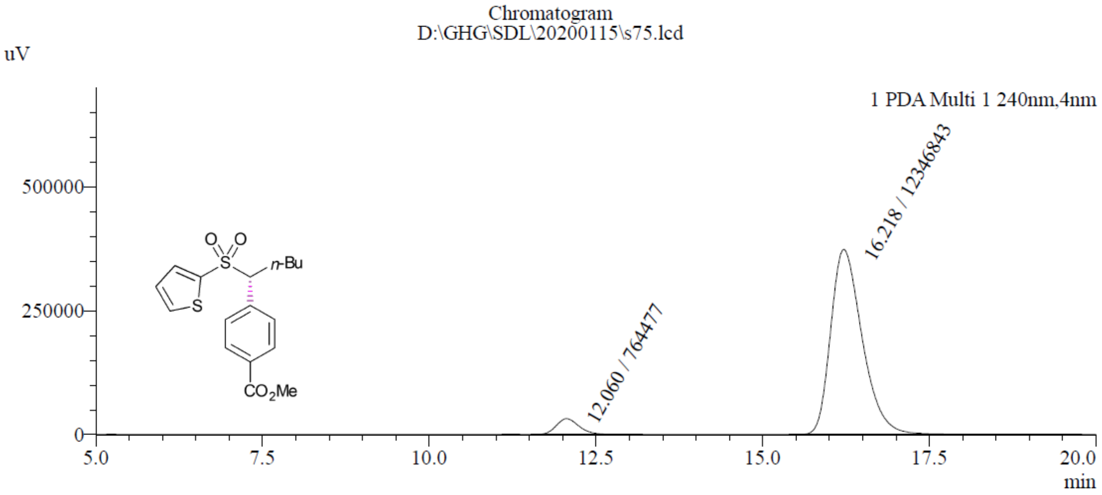

Peak Table

PDA Ch1 240nm

Peak#	Ret. Time [min]	Width [min]	Area [uV*s]	Height [uV]	Area %
1	12.060	0.3642	764476.82	31902.21	5.8307
2	16.218	0.5010	12346842.61	374157.70	94.1693

61: racemic

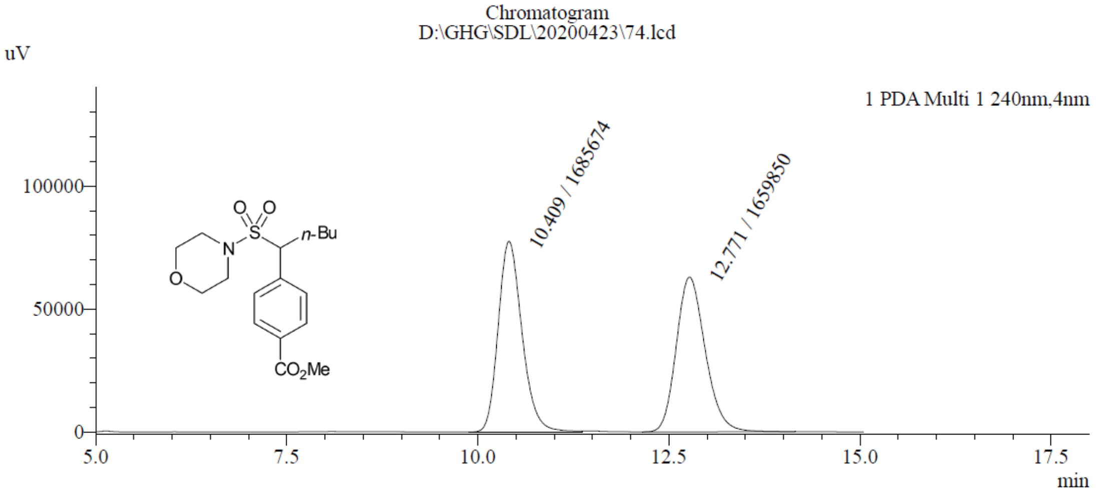

Peak Table

PDA Ch1 240nm

Peak#	Ret. Time [min]	Width [min]	Area [uV*s]	Height [uV]	Area %
1	10.409	0.3298	1685673.66	77583.83	50.3859
2	12.771	0.4033	1659849.63	62933.14	49.6141

61: enantioenriched, 89% ee

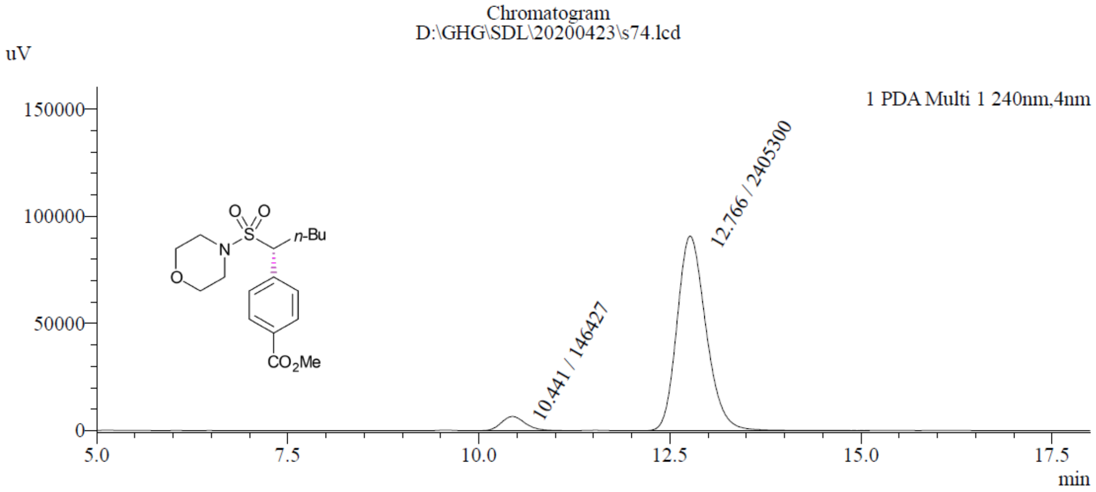

Peak Table

PDA Ch1 240nm

Peak#	Ret. Time [min]	Width [min]	Area [uV*s]	Height [uV]	Area %
1	10.441	0.3348	146427.42	6655.32	5.7384
2	12.766	0.4030	2405299.89	90859.26	94.2616

62: racemic

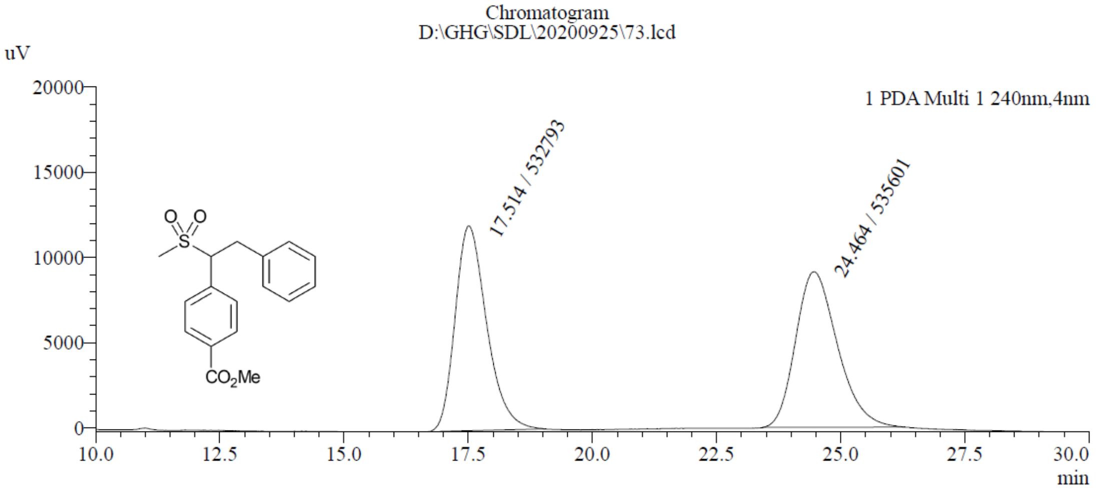

Peak Table

PDA Ch1 240nm

Peak#	Ret. Time [min]	Width [min]	Area [uV*s]	Height [uV]	Area %
1	17.514	0.6721	532793.45	12033.77	49.8686
2	24.464	0.9004	535601.09	9128.58	50.1314

62: enantioenriched, 81% ee

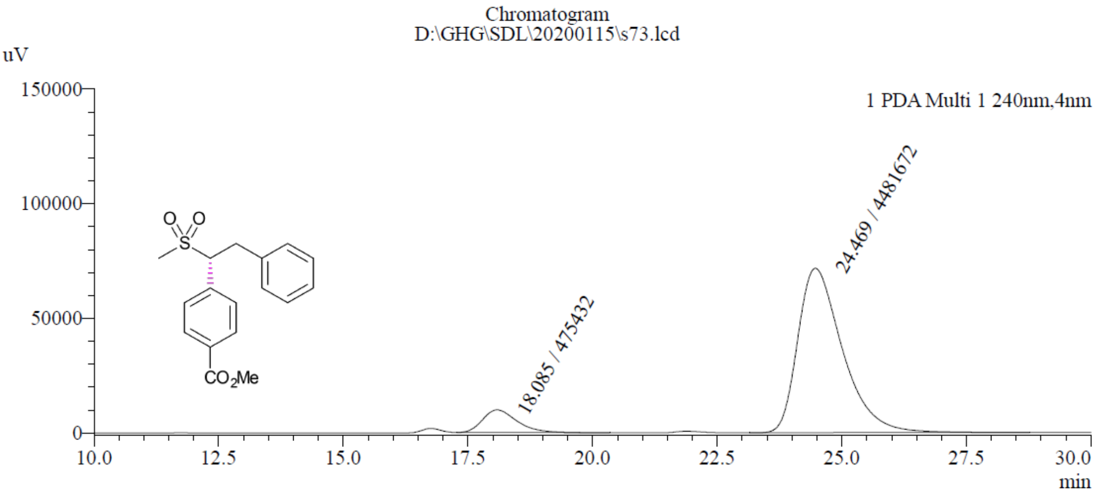

Peak Table

PDA Ch1 240nm

Peak#	Ret. Time [min]	Width [min]	Area [uV*s]	Height [uV]	Area %
1	18.085	0.7271	475432.05	9883.46	9.5909
2	24.469	0.9370	4481671.53	71715.28	90.4091

63: racemic

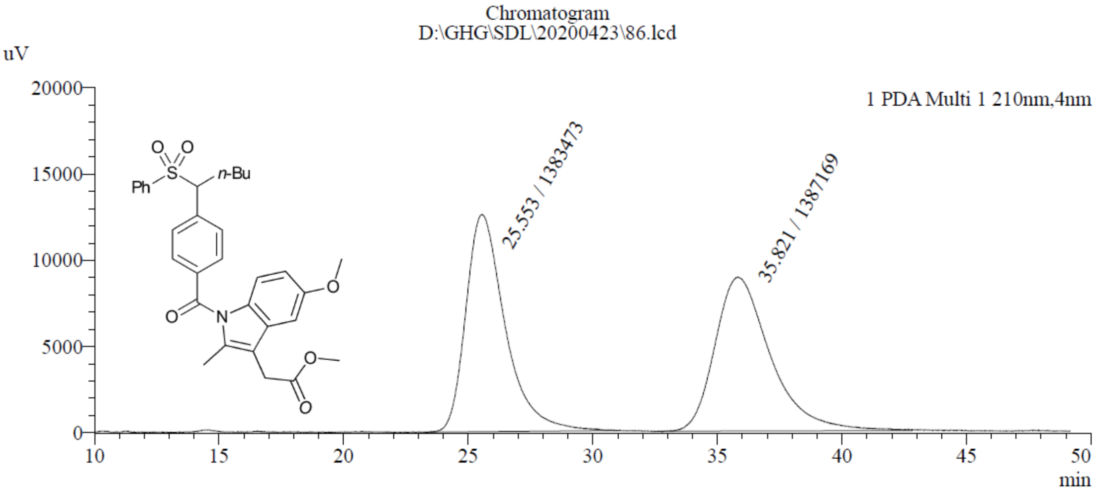

Peak Table

PDA Ch1 210nm

Peak#	Ret. Time [min]	Width [min]	Area [uV*s]	Height [uV]	Area %
1	25.553	1.6127	1383472.89	12587.36	49.9333
2	35.821	2.2835	1387168.78	8931.38	50.0667

63: enantioenriched, 92% ee

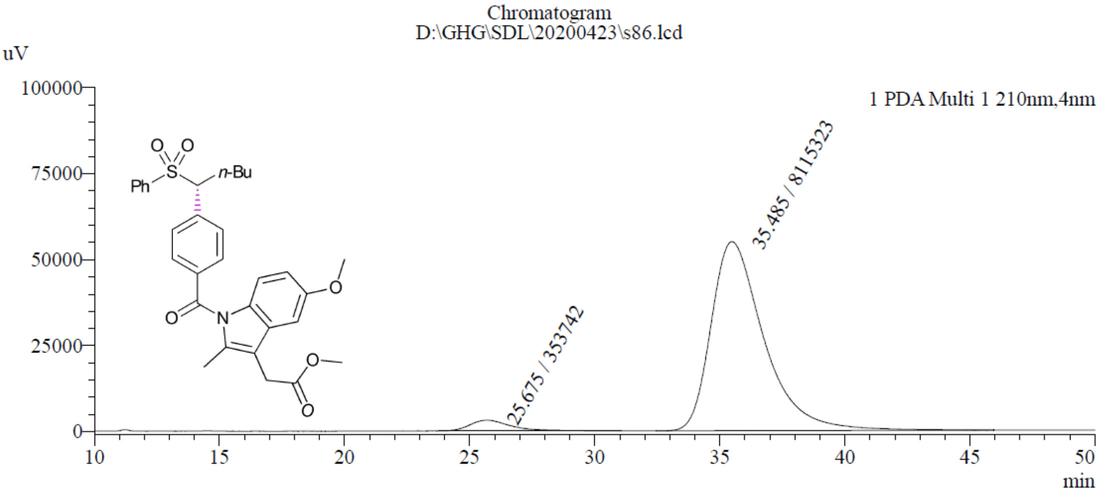

Peak Table

PDA Ch1 210nm

Peak#	Ret. Time [min]	Width [min]	Area [uV*s]	Height [uV]	Area %
1	25.675	1.6540	353742.48	3136.57	4.1769
2	35.485	2.1587	8115322.87	55023.23	95.8231

64: racemic

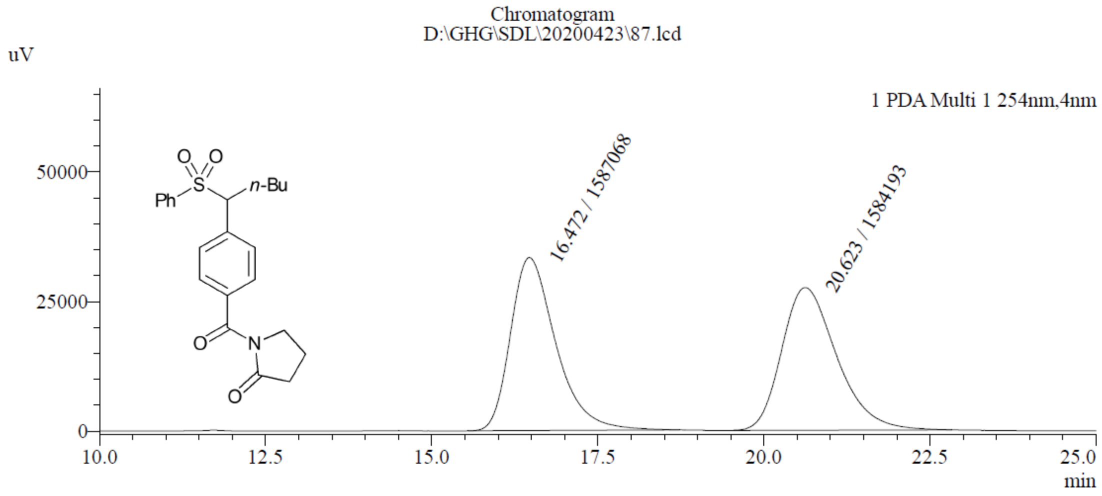

Peak Table

PDA Ch1 254nm

Peak#	Ret. Time [min]	Width [min]	Area [uV*s]	Height [uV]	Area %
1	16.472	0.7098	1587067.80	33390.97	50.0453
2	20.623	0.8765	1584192.67	27523.50	49.9547

64: enantioenriched, 92% ee

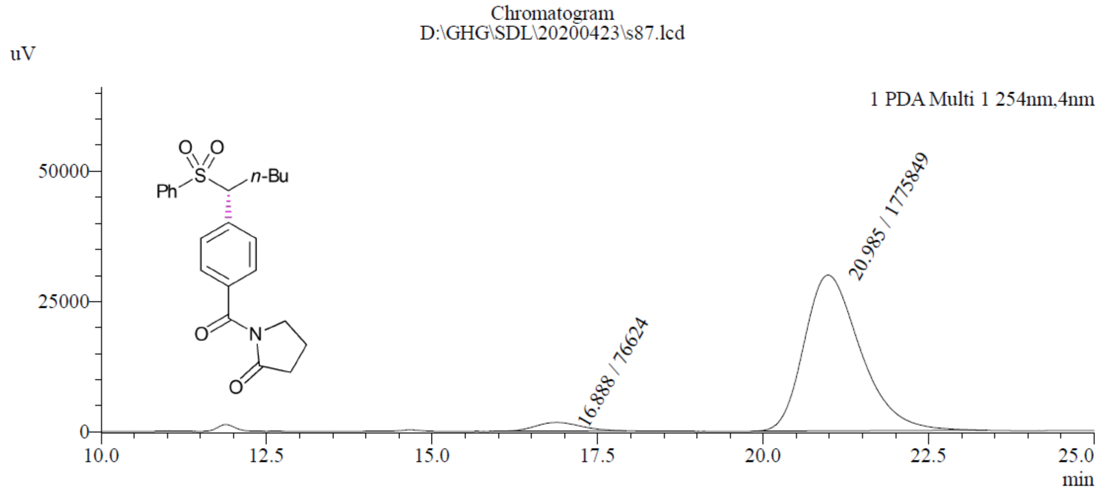

Peak Table

PDA Ch1 254nm

Peak#	Ret. Time [min]	Width [min]	Area [uV*s]	Height [uV]	Area %
1	16.888	0.7385	76624.50	1641.45	4.1363
2	20.985	0.9011	1775848.73	29914.68	95.8637

65: racemic

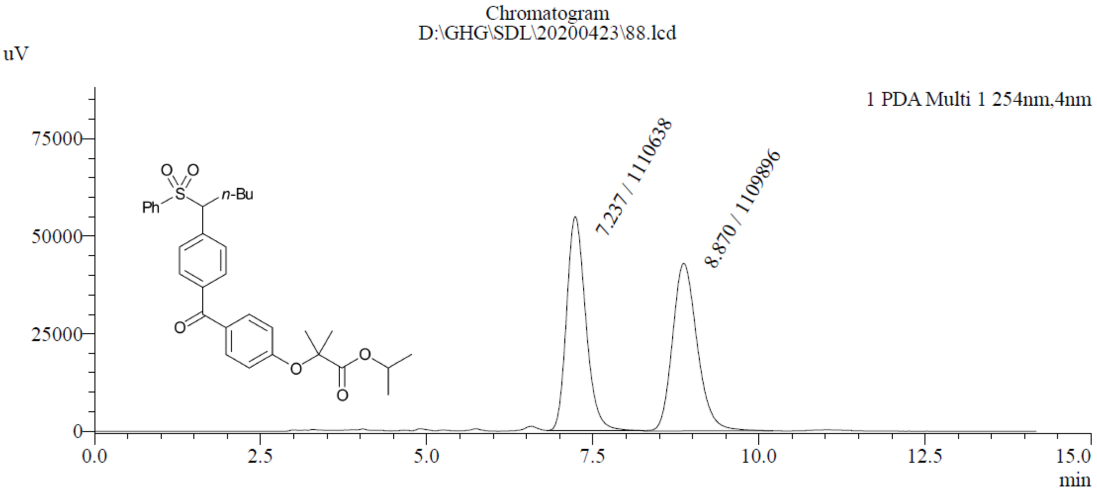

Peak Table

PDA Ch1 254nm

Peak#	Ret. Time [min]	Width [min]	Area [uV*s]	Height [uV]	Area %
1	7.237	0.3054	1110638.31	54949.75	50.0167
2	8.870	0.3919	1109896.20	42953.79	49.9833

65: enantioenriched, 92% ee

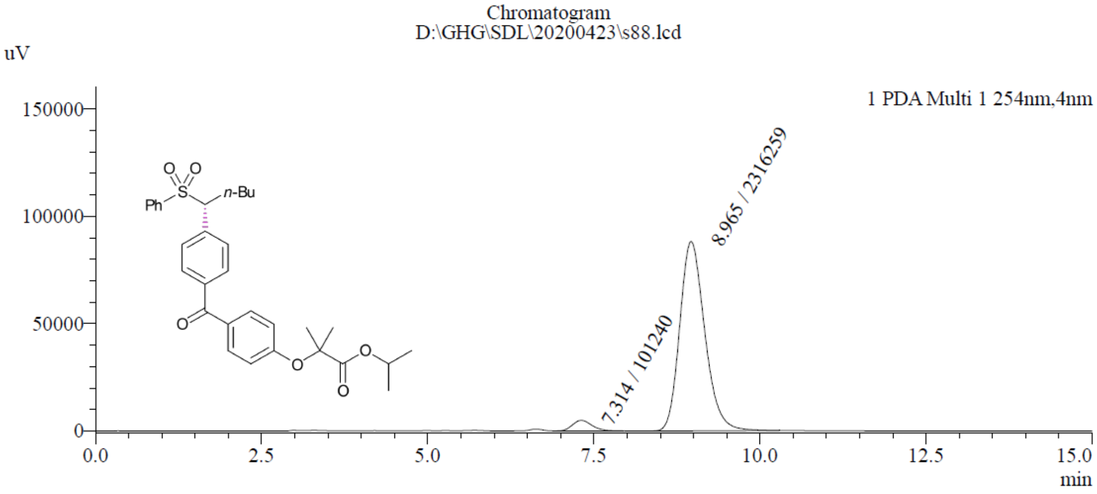

Peak Table

PDA Ch1 254nm

Peak#	Ret. Time [min]	Width [min]	Area [uV*s]	Height [uV]	Area %
1	7.314	0.3160	101240.20	4927.83	4.1878
2	8.965	0.4002	2316258.58	88189.84	95.8122

66: racemic

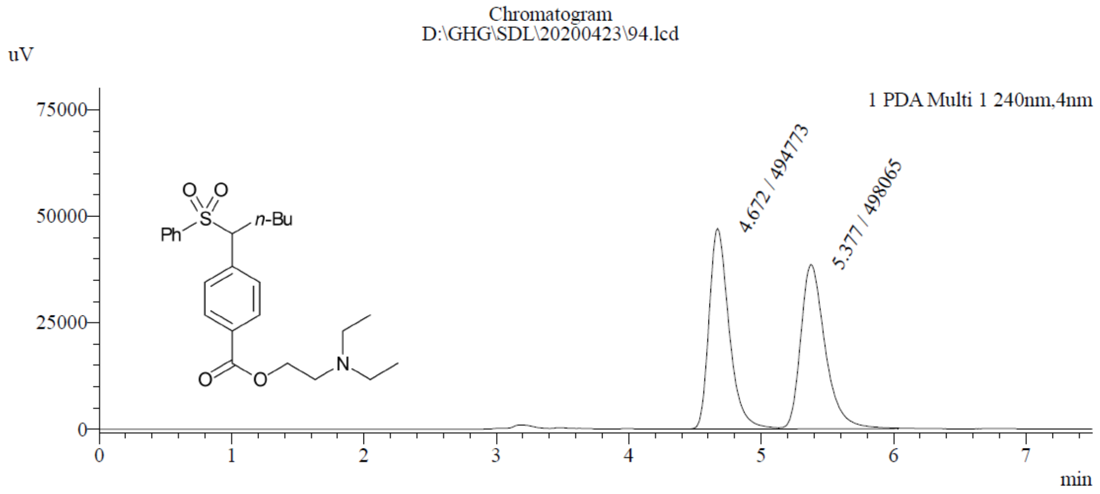

Peak Table

PDA Ch1 240nm

Peak#	Ret. Time [min]	Width [min]	Area [uV*s]	Height [uV]	Area %
1	4.672	0.1571	494772.93	47061.93	49.8342
2	5.377	0.1908	498064.72	38592.77	50.1658

66: enantioenriched, 91% ee

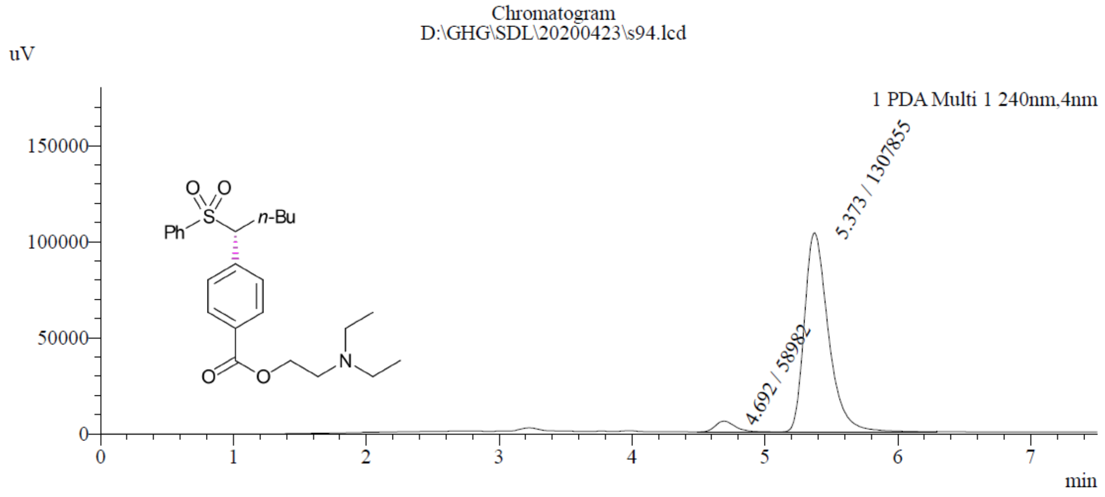

Peak Table

PDA Ch1 240nm

Peak#	Ret. Time [min]	Width [min]	Area [uV*s]	Height [uV]	Area %
1	4.692	0.1583	58982.28	5620.27	4.3152
2	5.373	0.1871	1307855.44	103821.66	95.6848

67: racemic

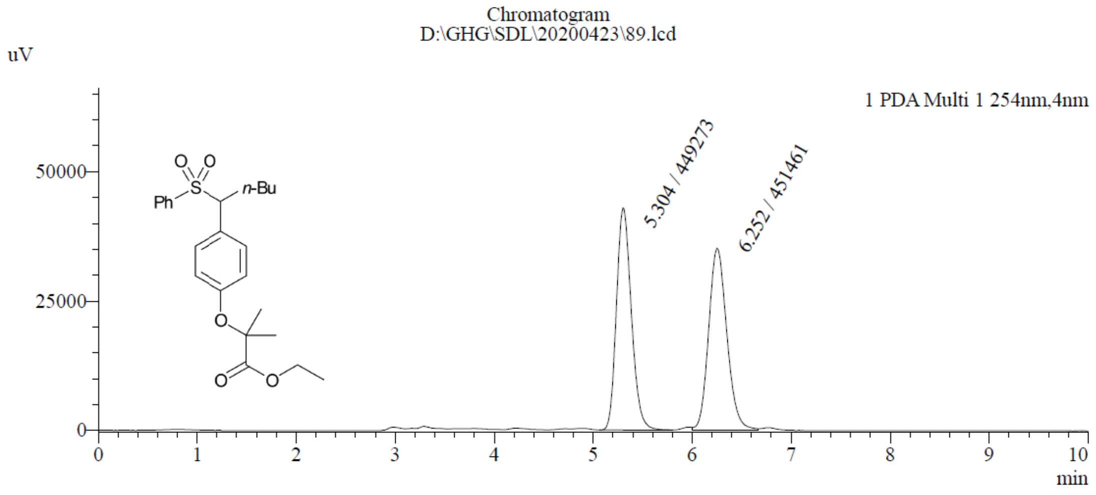

Peak Table

PDA Ch1 254nm

Peak#	Ret. Time [min]	Width [min]	Area [uV*s]	Height [uV]	Area %
1	5.304	0.1601	449273.02	42946.02	49.8785
2	6.252	0.1964	451461.11	35158.96	50.1215

67: enantioenriched, 88% ee

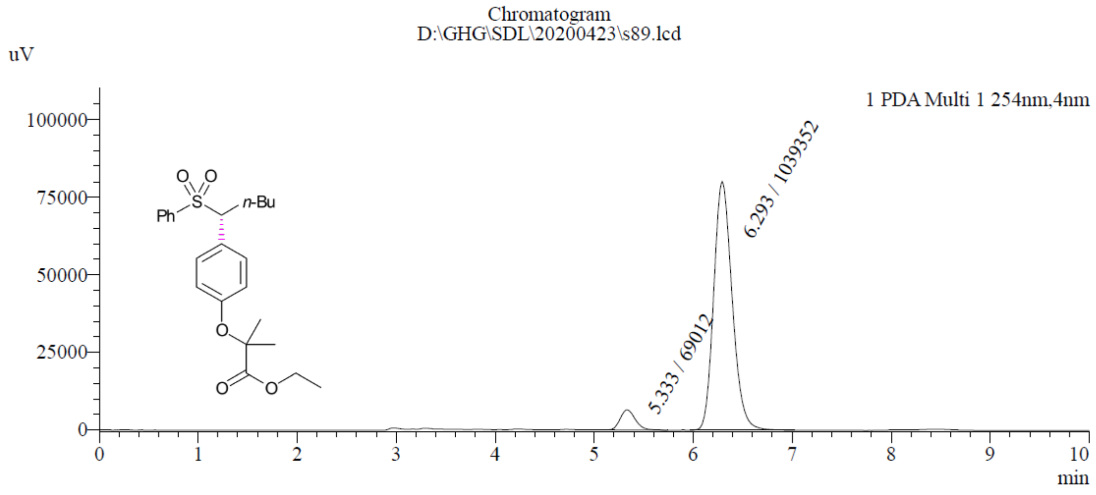

Peak Table

PDA Ch1 254nm

Peak#	Ret. Time [min]	Width [min]	Area [uV*s]	Height [uV]	Area %
1	5.333	0.1617	69011.70	6535.59	6.2265
2	6.293	0.1990	1039351.54	80081.11	93.7735

68: racemic

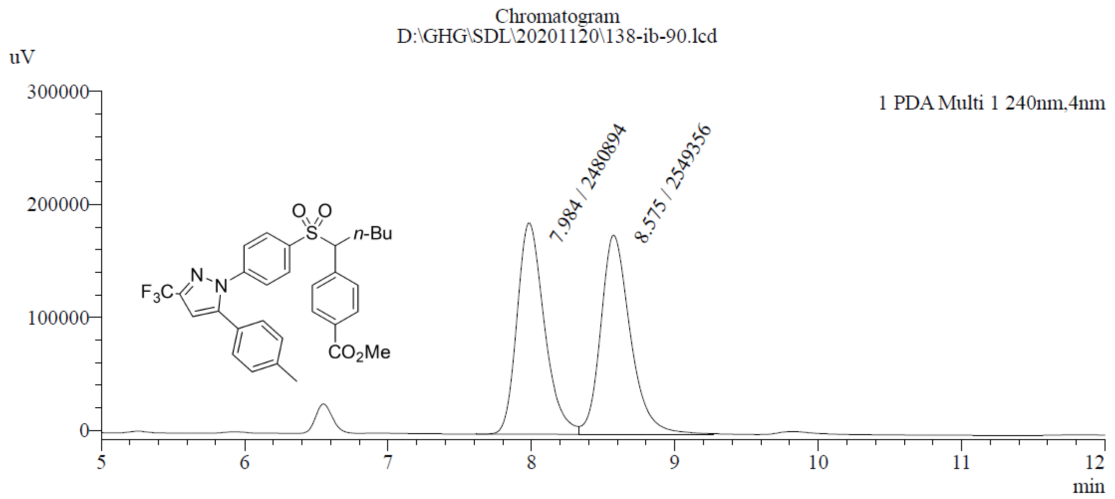

Peak Table

PDA Ch1 240nm

Peak#	Ret. Time [min]	Width [min]	Area [uV*s]	Height [uV]	Area %
1	7.984	0.2002	2480893.70	186877.17	49.3195
2	8.575	0.2139	2549356.03	176465.23	50.6805

68: enantioenriched, 91% ee

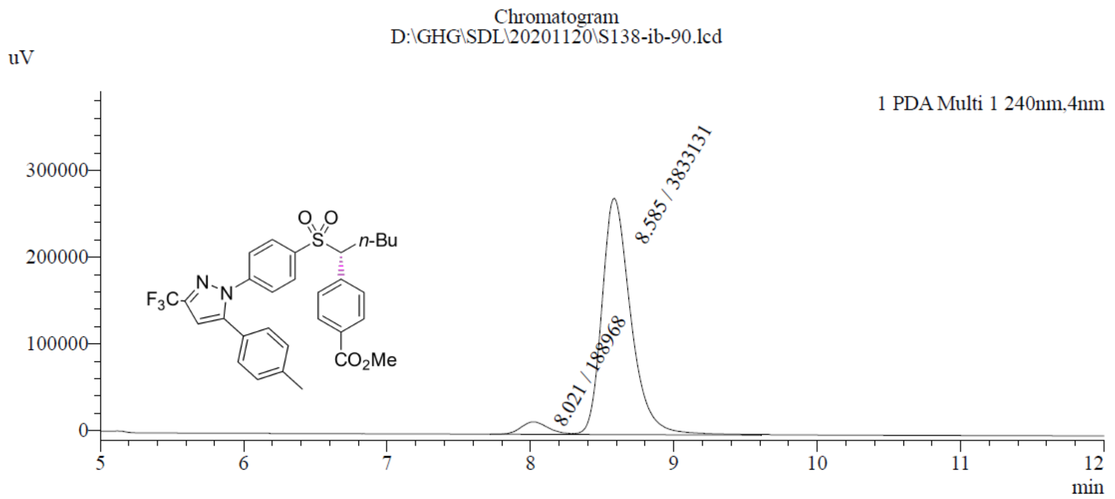

Peak Table

PDA Ch1 240nm

Peak#	Ret. Time [min]	Width [min]	Area [uV*s]	Height [uV]	Area %
1	8.021	0.2014	188968.13	14335.84	4.6982
2	8.585	0.2084	3833130.82	272472.59	95.3018

69: racemic

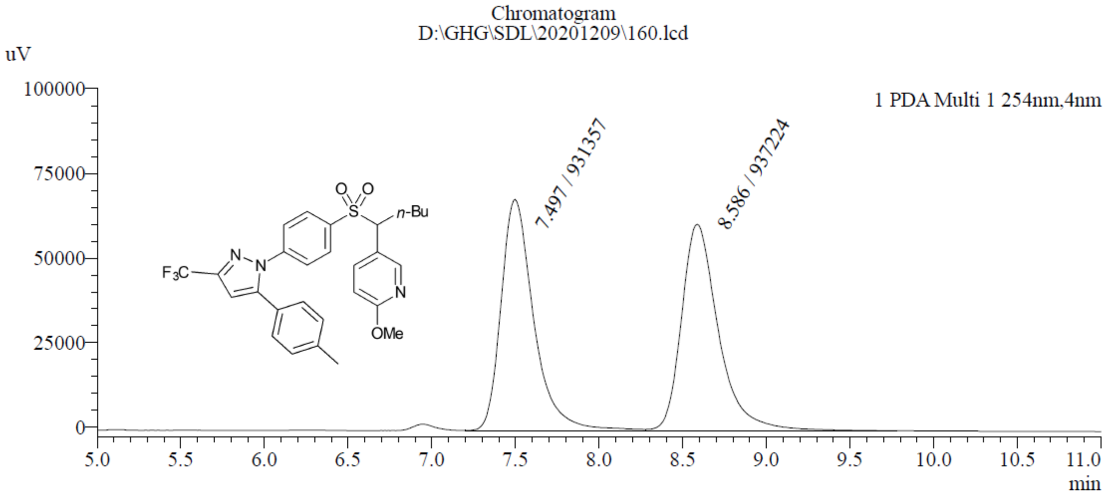

Peak Table

PDA Ch1 254nm

Peak#	Ret. Time [min]	Width [min]	Area [uV*s]	Height [uV]	Area %
1	7.497	0.1981	931357.12	68363.69	49.8430
2	8.586	0.2234	937224.29	61018.30	50.1570

69: enantioenriched, 87% ee

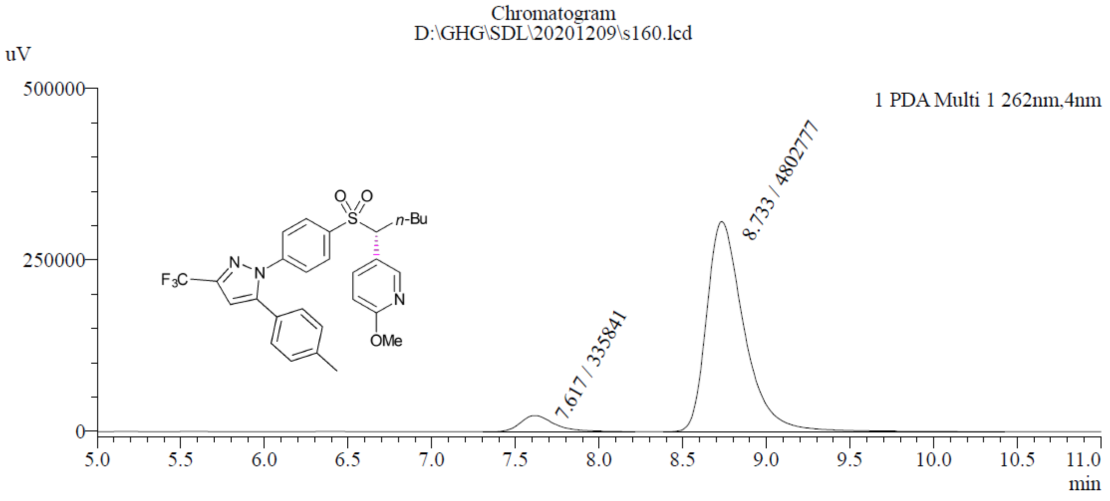

Peak Table

PDA Ch1 262nm

Peak#	Ret. Time [min]	Width [min]	Area [uV*s]	Height [uV]	Area %
1	7.617	0.2063	335841.01	23656.21	6.5356
2	8.733	0.2292	4802777.28	306962.04	93.4644

IV. NMR Data

SM1, ^{1}H-NMR (600 MHZ, $CDCl_3$)

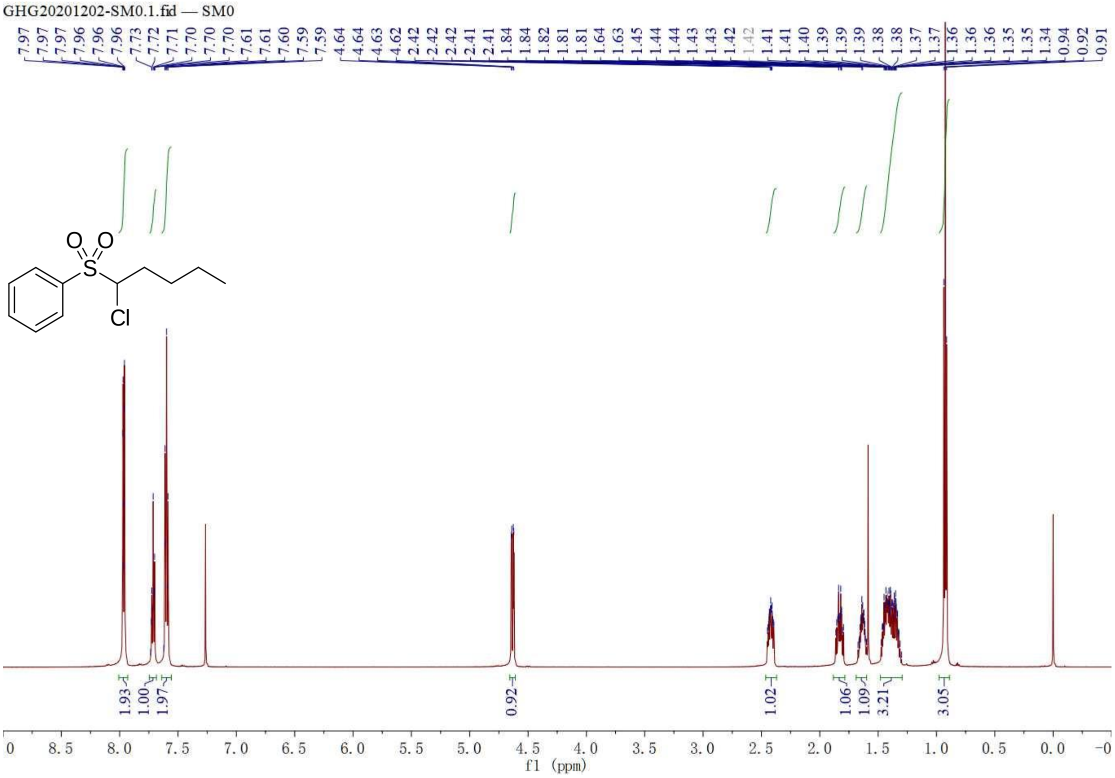

SM1, ^{13}C NMR (151 MHz, $CDCl_3$)

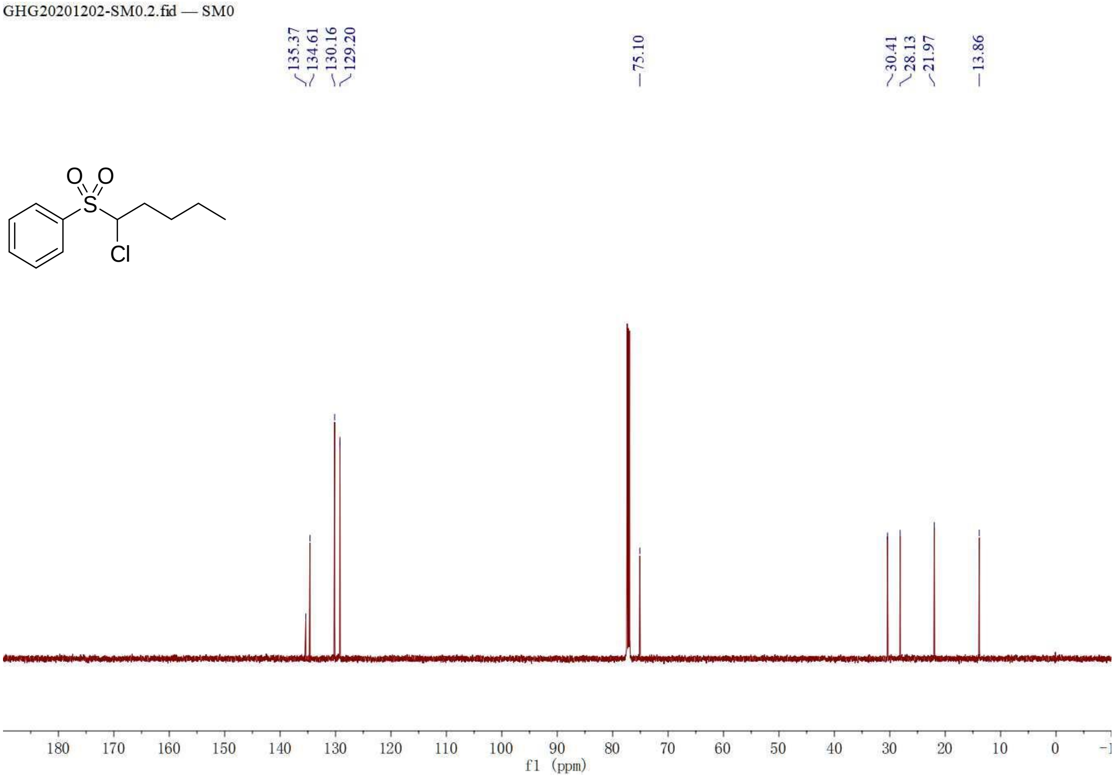

SM2, ^{1}H-NMR (600 MHZ, $CDCl_3$)

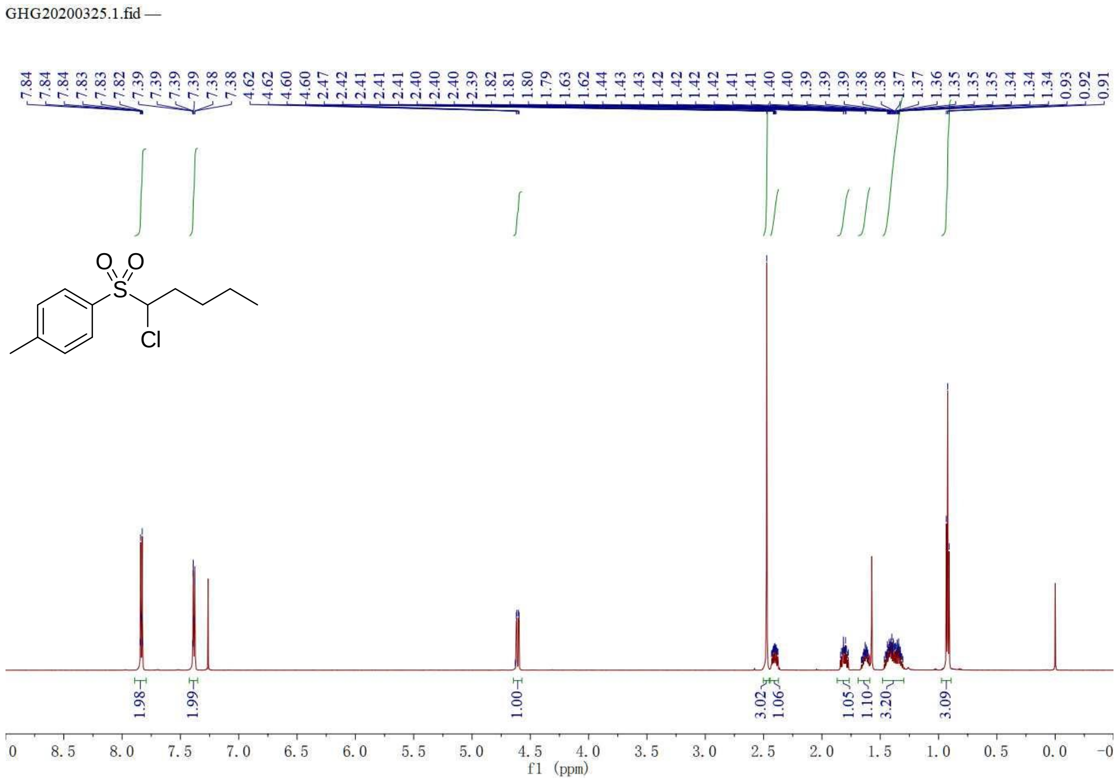

SM2, ^{13}C NMR (151 MHz, $CDCl_3$)

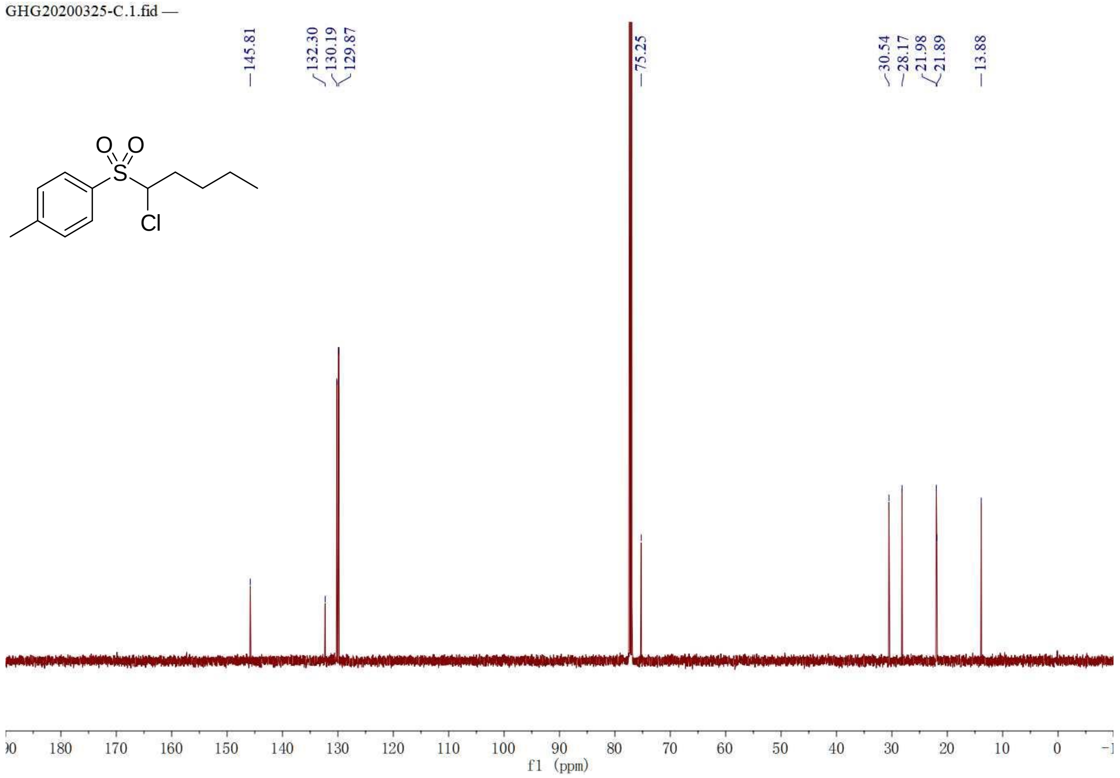

SM3, ^{1}H-NMR (600 MHZ, $CDCl_3$)

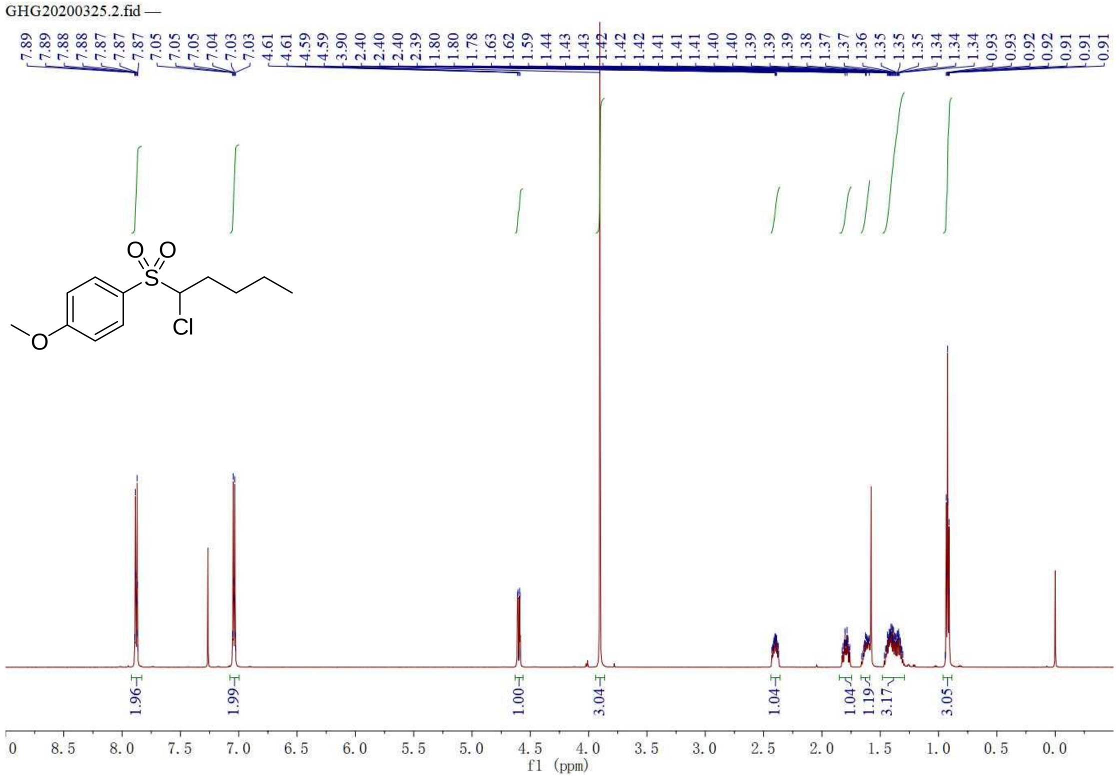

SM3, ^{13}C NMR (151 MHz, $CDCl_3$)

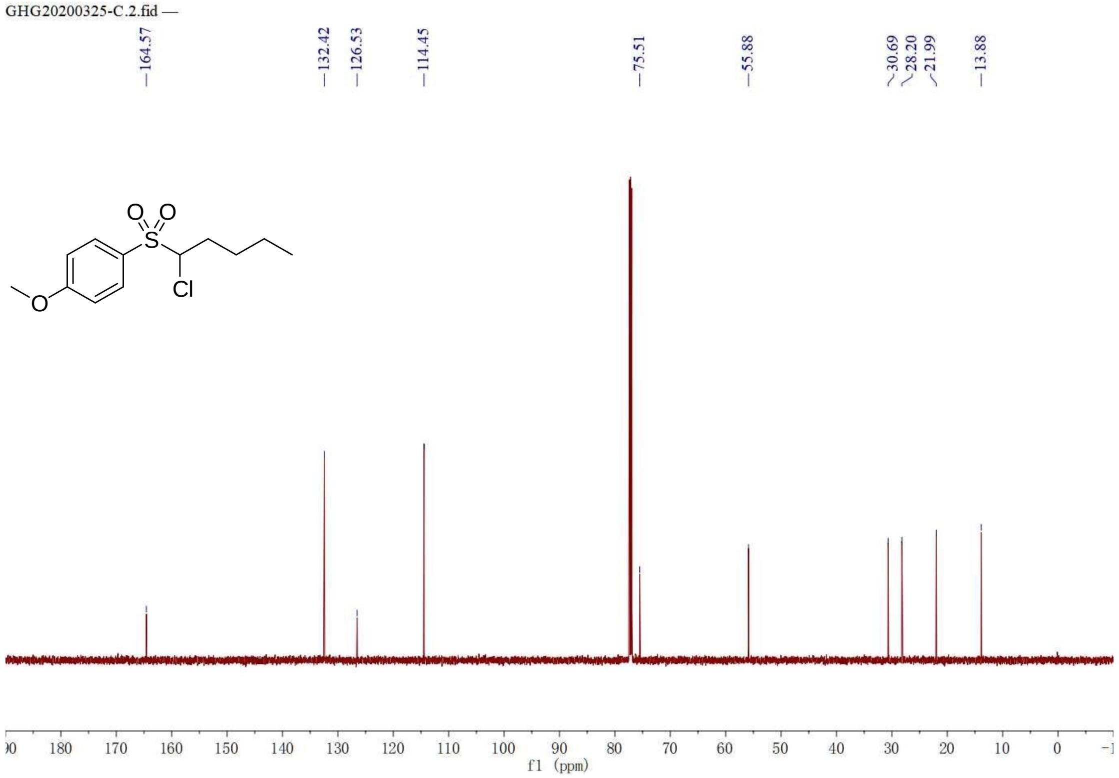

SM4, ^{1}H-NMR (600 MHZ, $CDCl_3$)

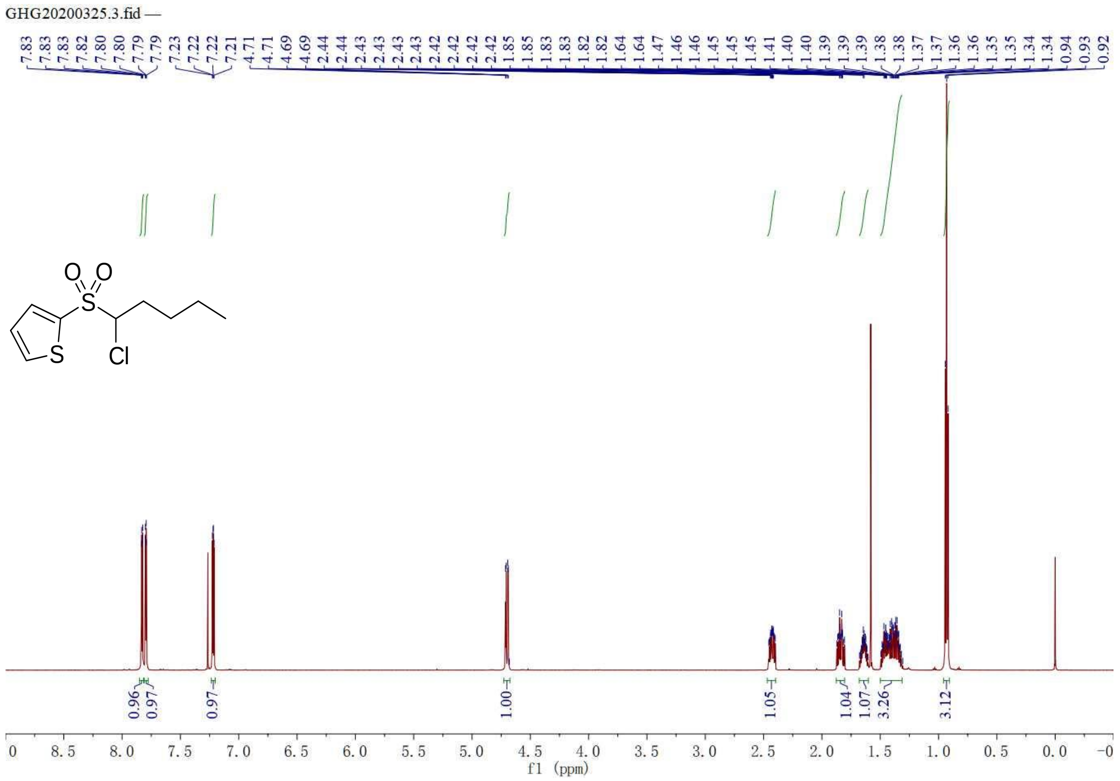

SM4, ^{13}C NMR (151 MHz, $CDCl_3$)

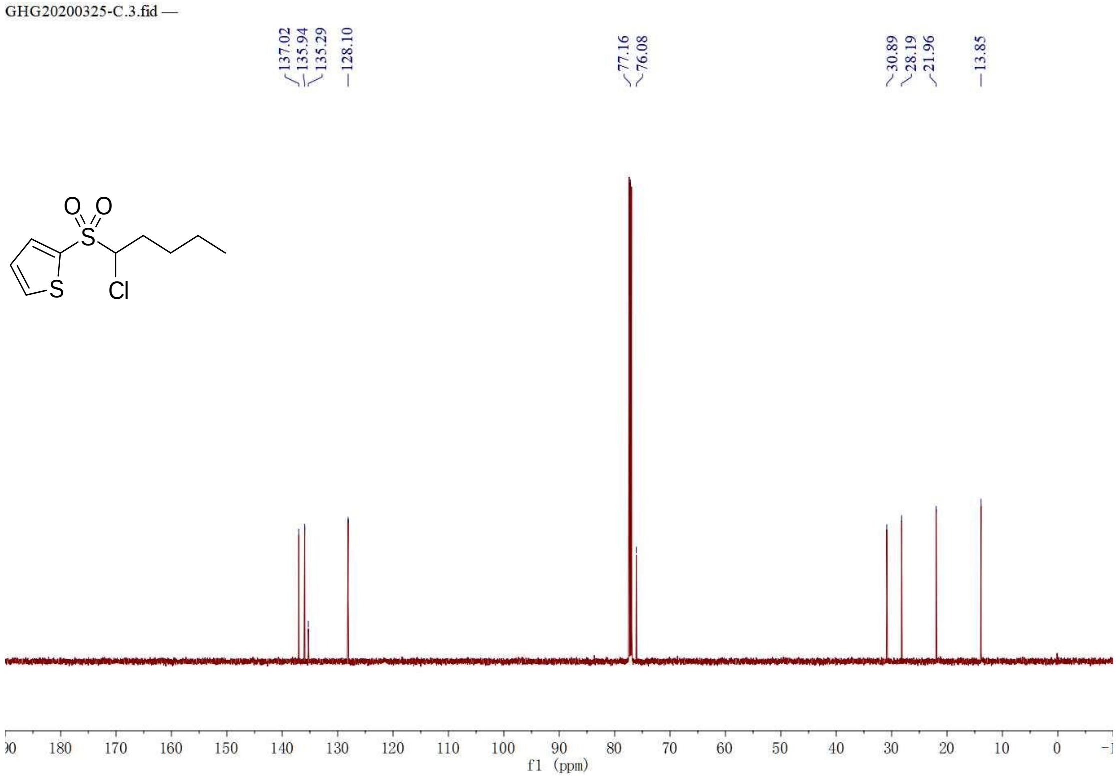

SM5, ^{1}H-NMR (600 MHZ, $CDCl_3$)

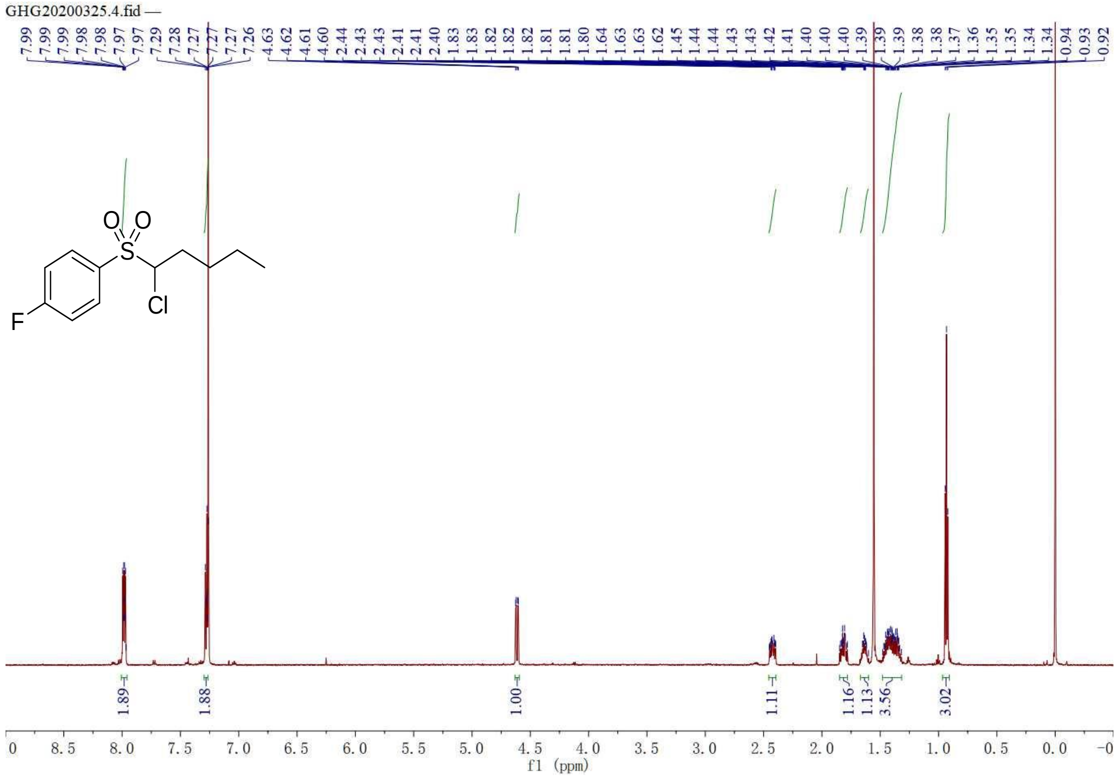

SM5, ^{13}C NMR (151 MHz, $CDCl_3$)

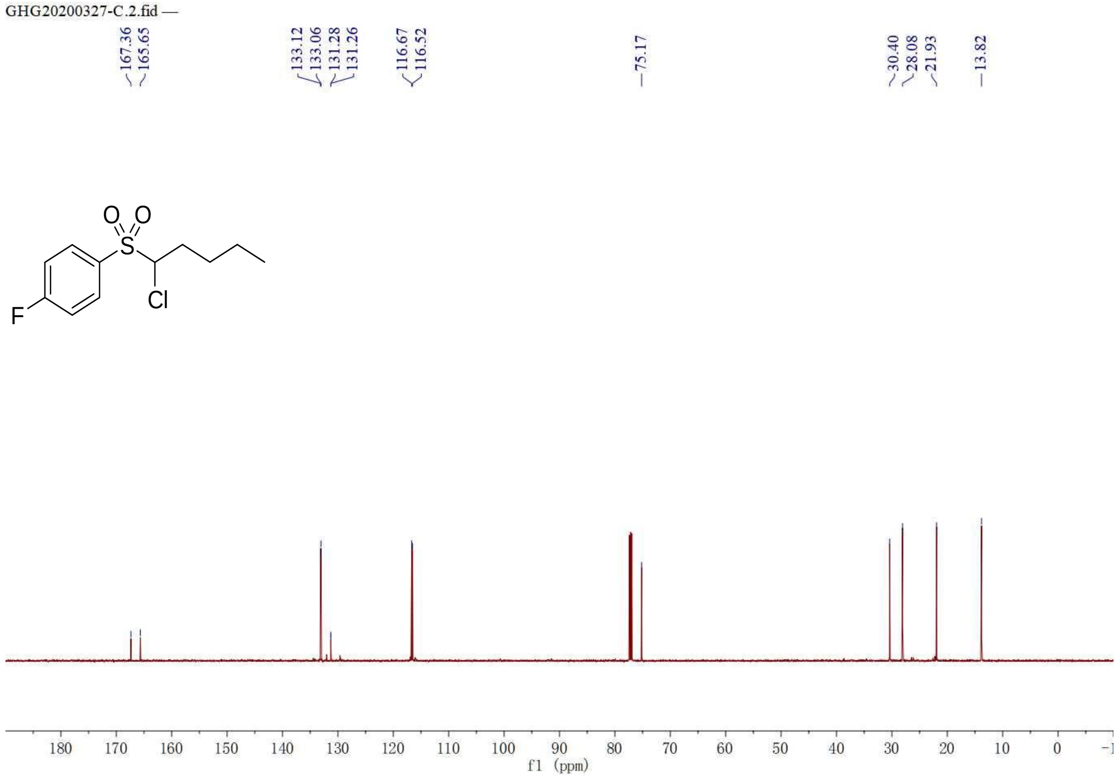

SM5, ^{19}F NMR (565 MHz, $CDCl_3$)

GHG20200416-F.1.fid — SM5

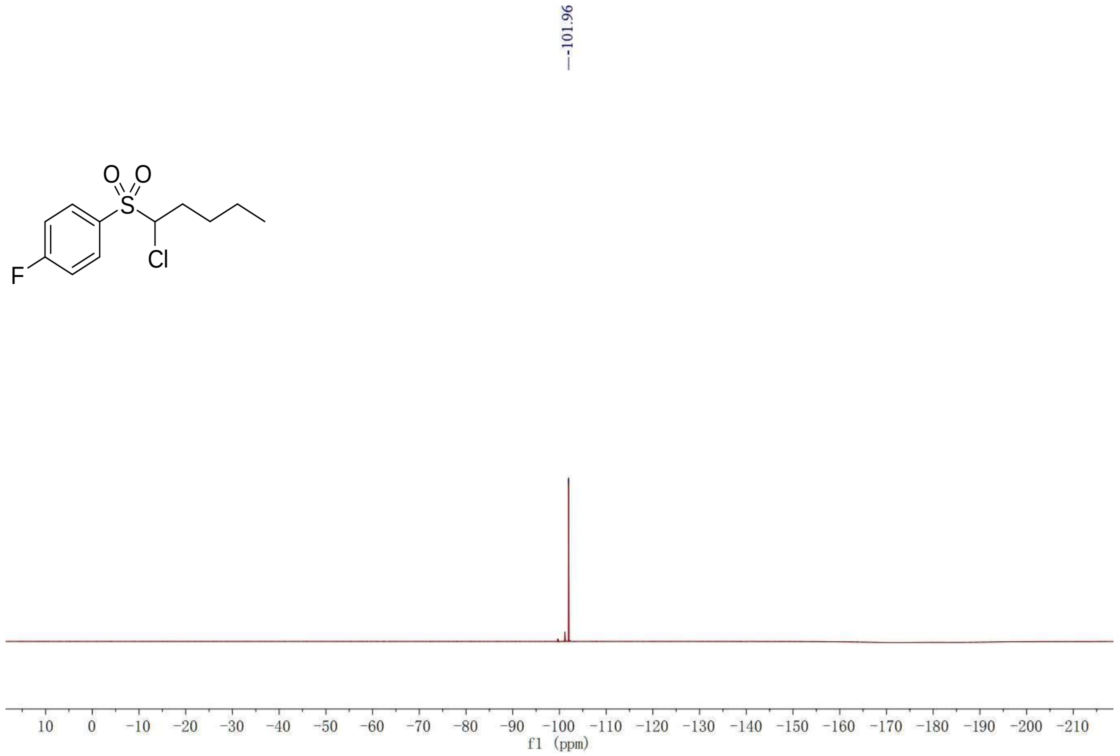

SM6, ^{1}H-NMR (600 MHZ, $CDCl_3$)

GHG20200325.5.fid —

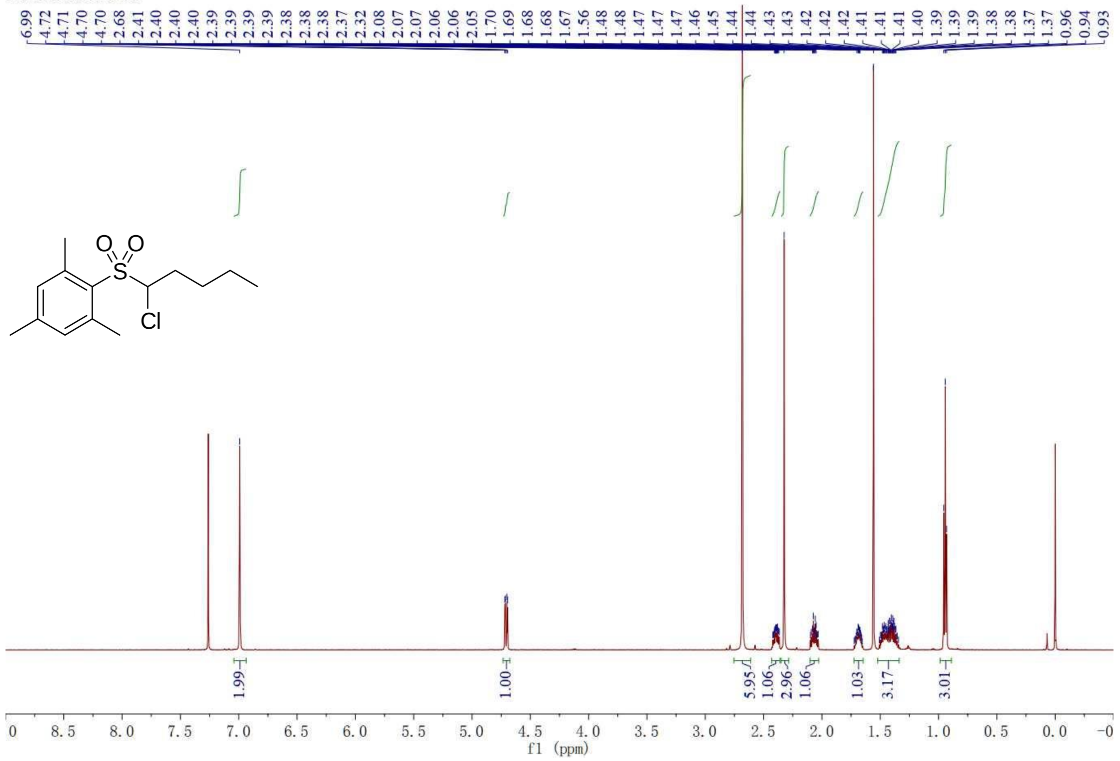

SM6, ^{13}C NMR (151 MHz, $CDCl_3$)

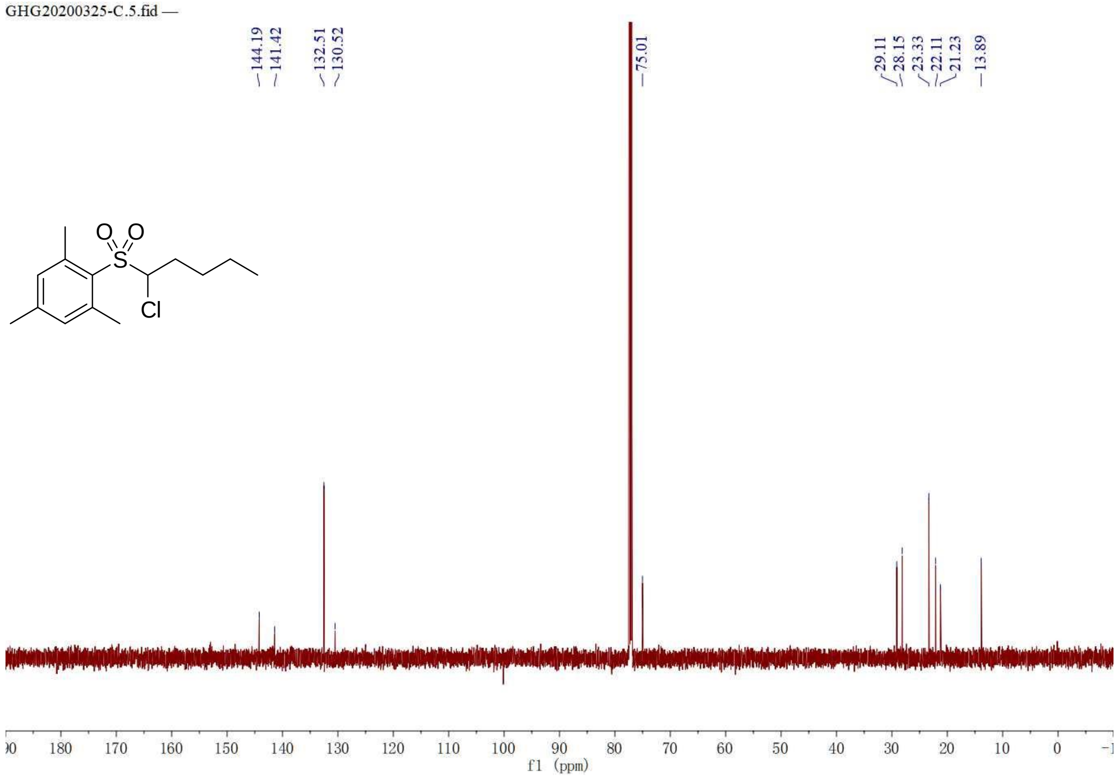

SM7, ^1H-NMR (600 MHZ, $CDCl_3$)

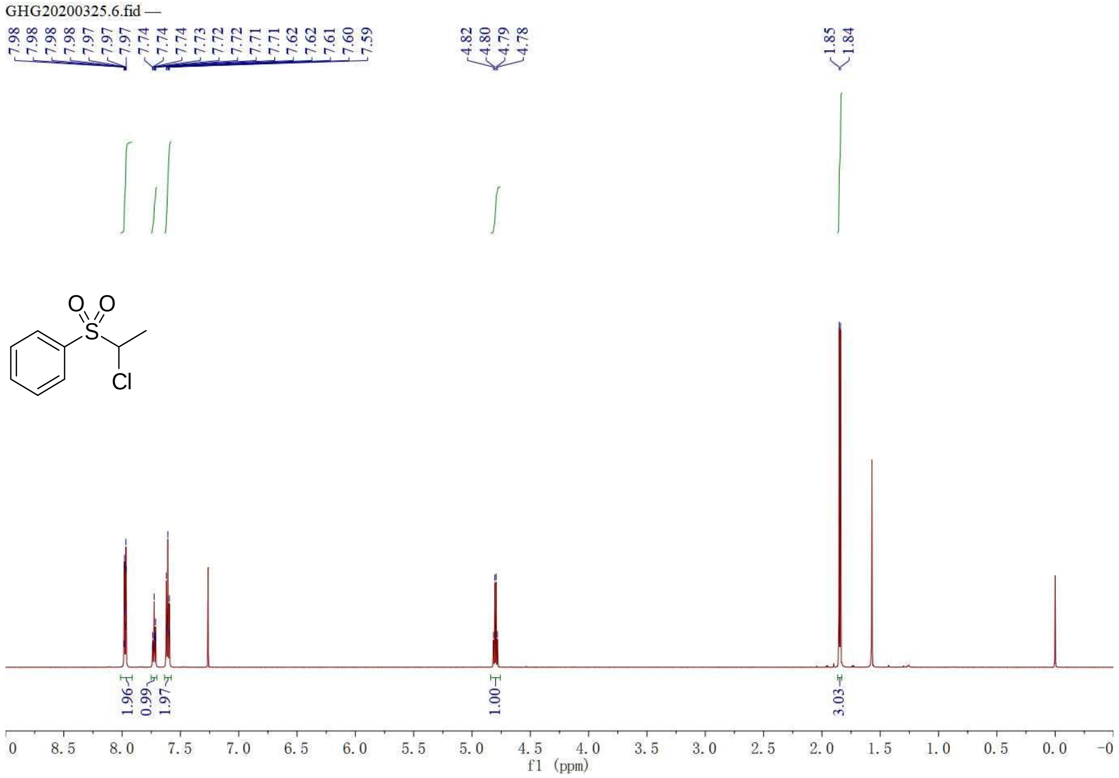

SM7, ^{13}C NMR (151 MHz, $CDCl_3$)

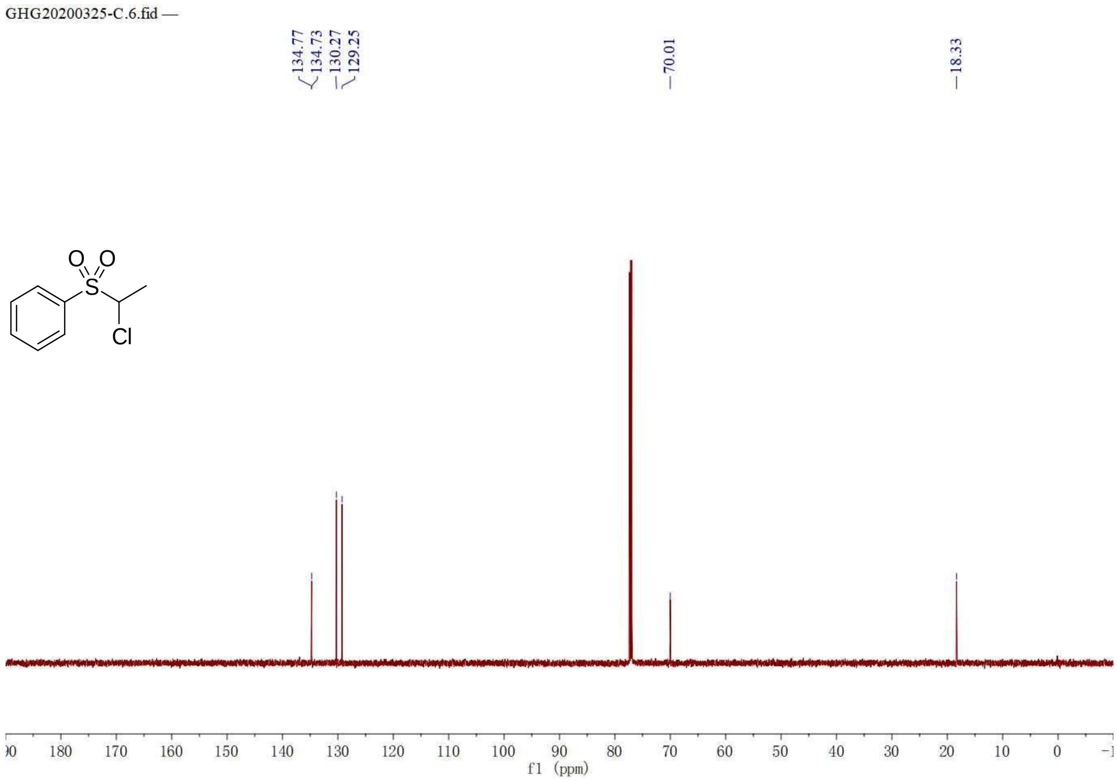

SM8, ^{1}H-NMR (600 MHZ, $CDCl_3$)

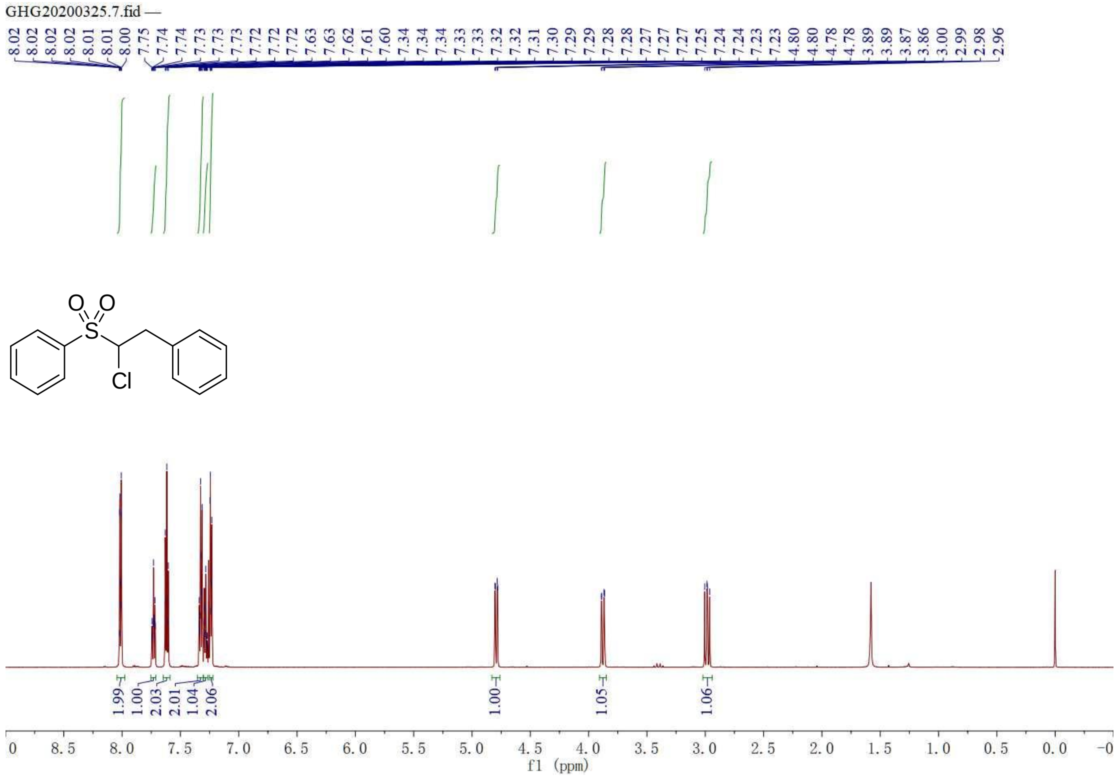

SM8, ^{13}C NMR (151 MHz, $CDCl_3$)

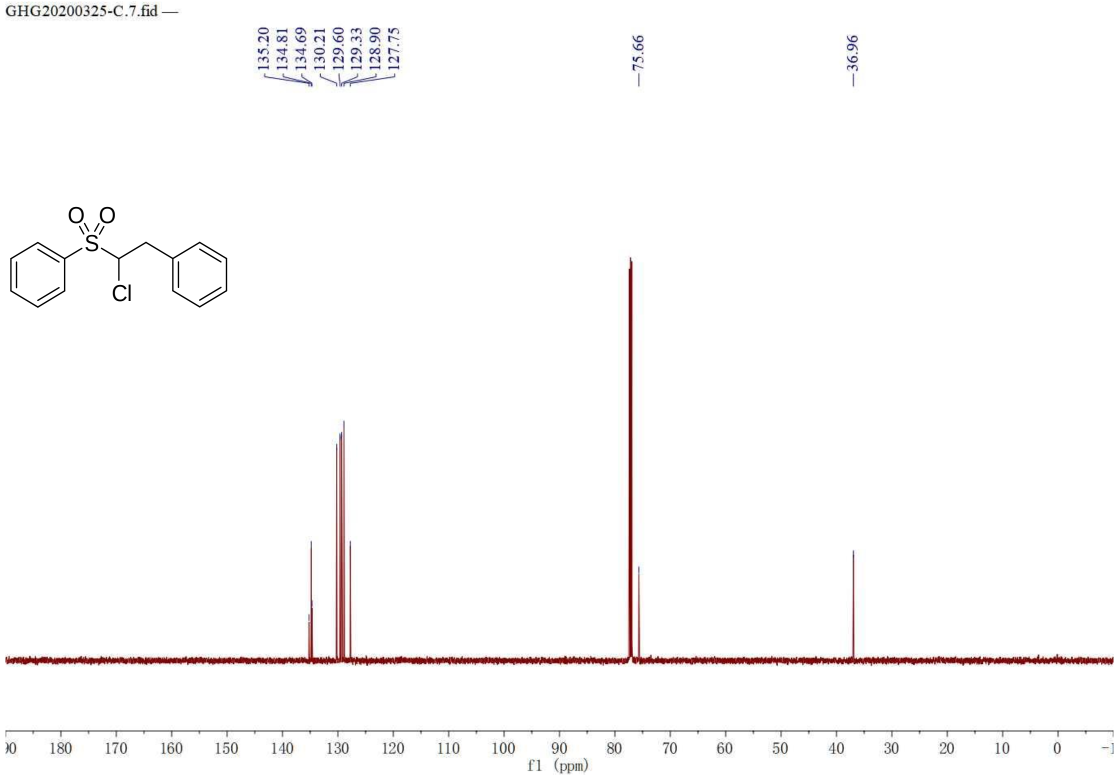

SM9, ^{1}H-NMR (600 MHZ, $CDCl_3$)

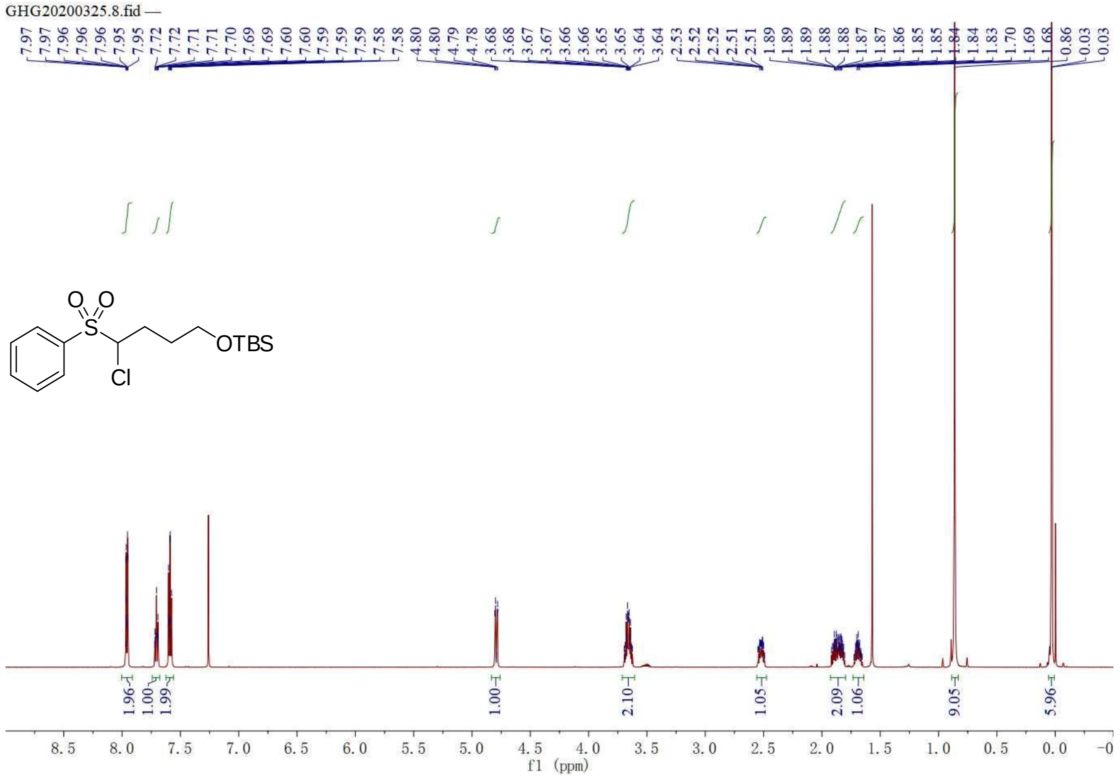

SM9, ^{13}C NMR (151 MHz, $CDCl_3$)

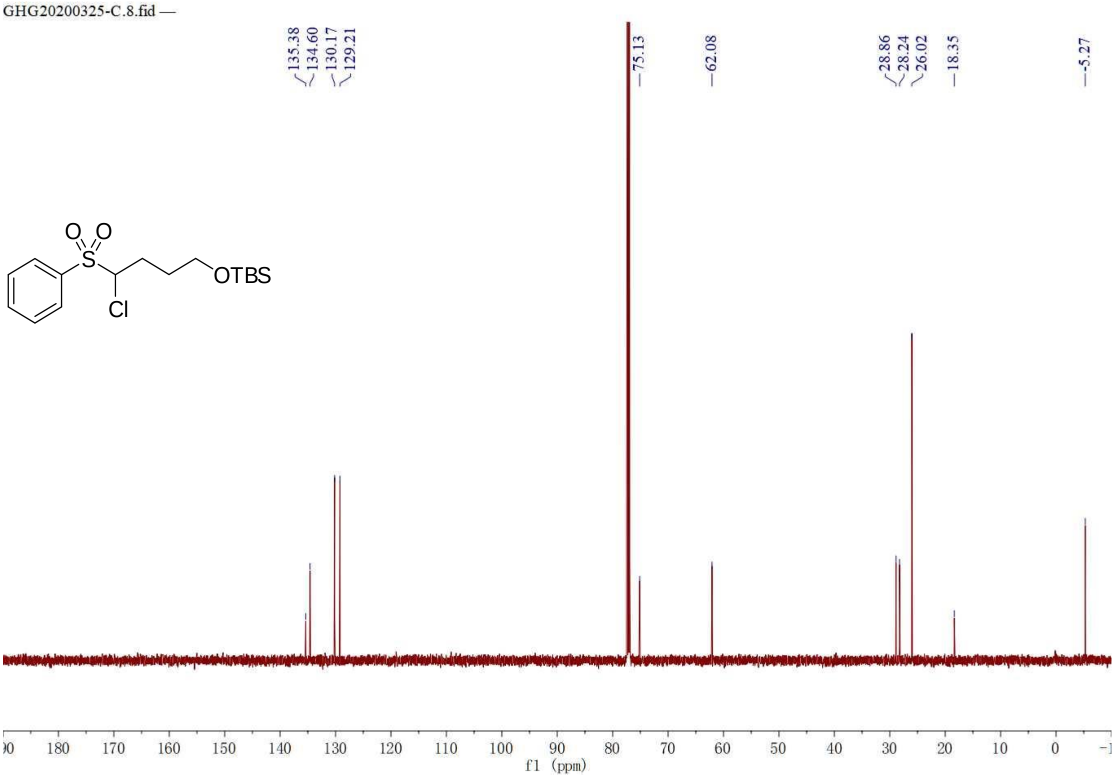

SM10, ^{1}H-NMR (600 MHZ, $CDCl_3$)

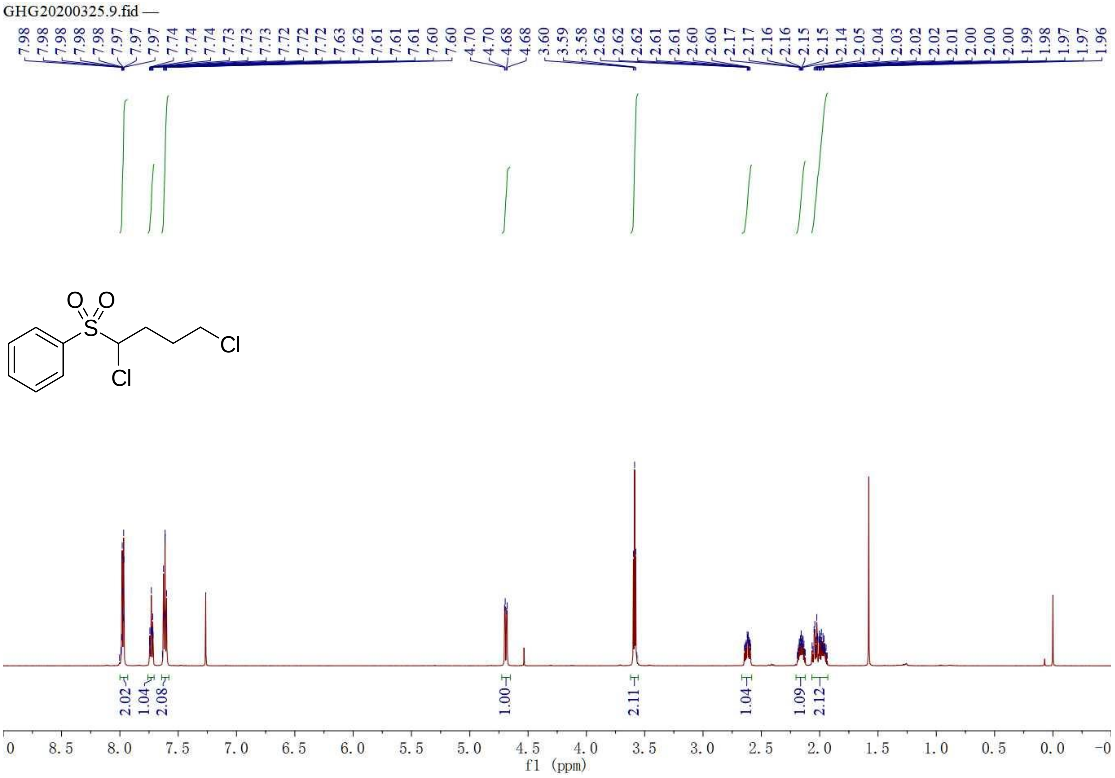

SM10, ^{13}C NMR (151 MHz, $CDCl_3$)

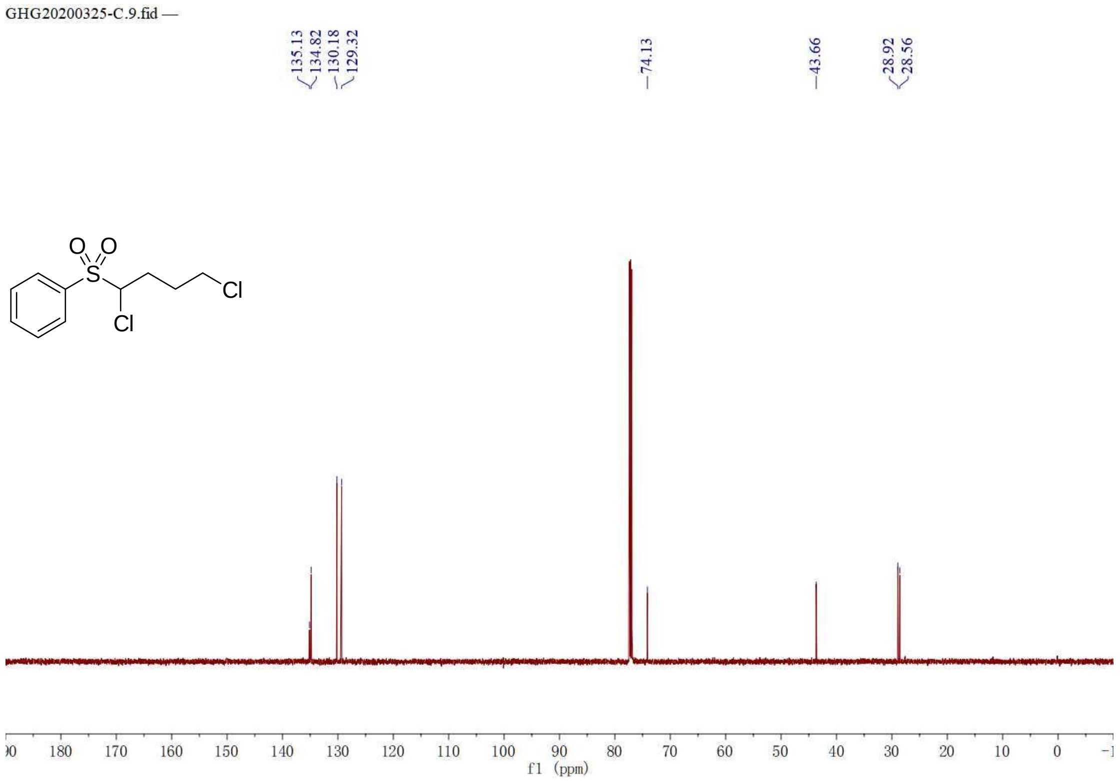

SM11, ^{1}H-NMR (600 MHZ, $CDCl_3$)

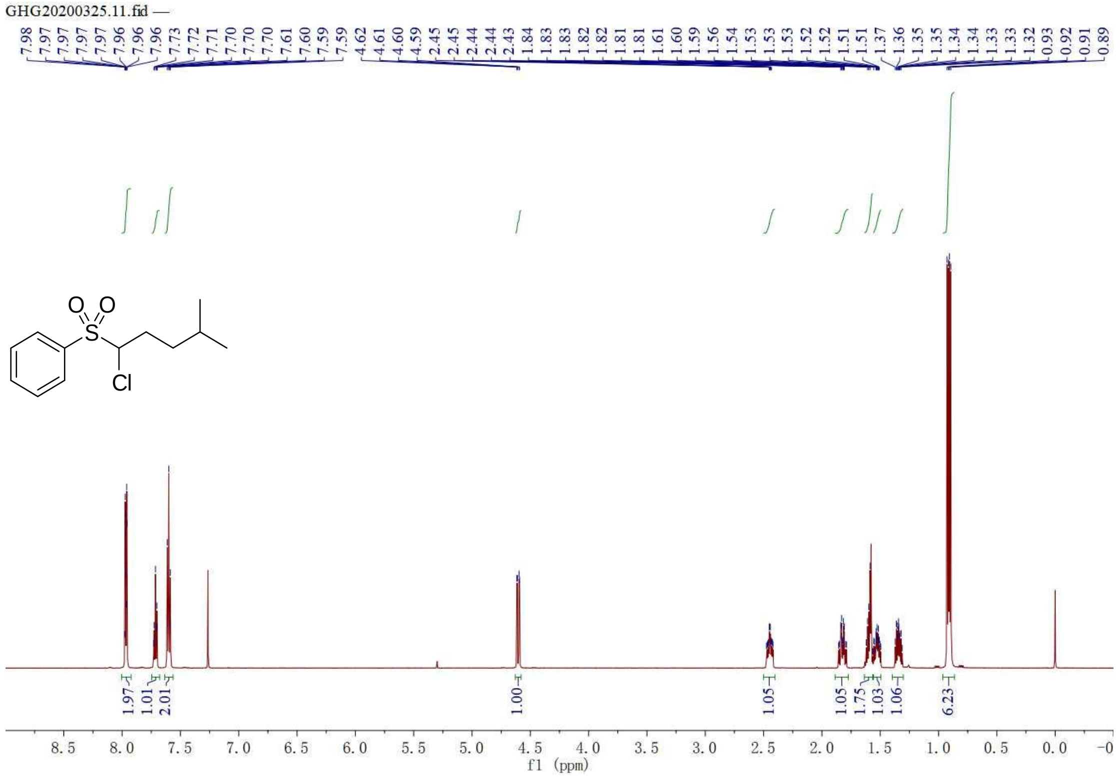

SM11, ^{13}C NMR (151 MHz, $CDCl_3$)

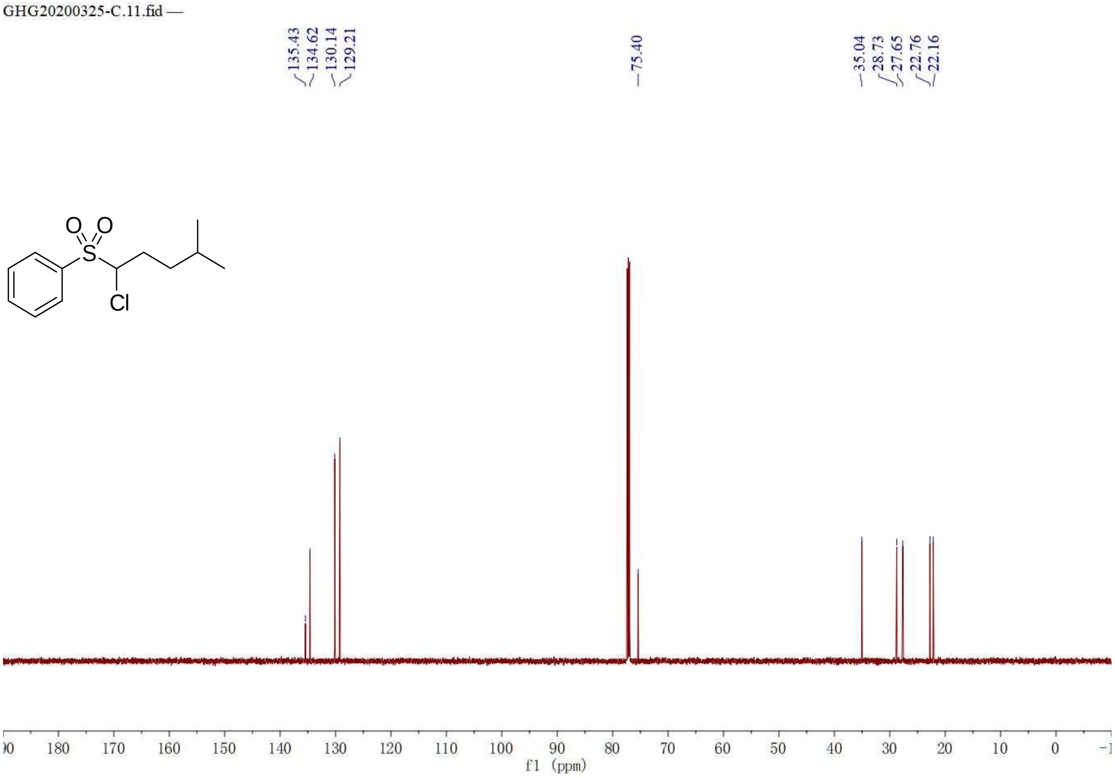

SM12, ^{1}H-NMR (600 MHZ, $CDCl_3$)

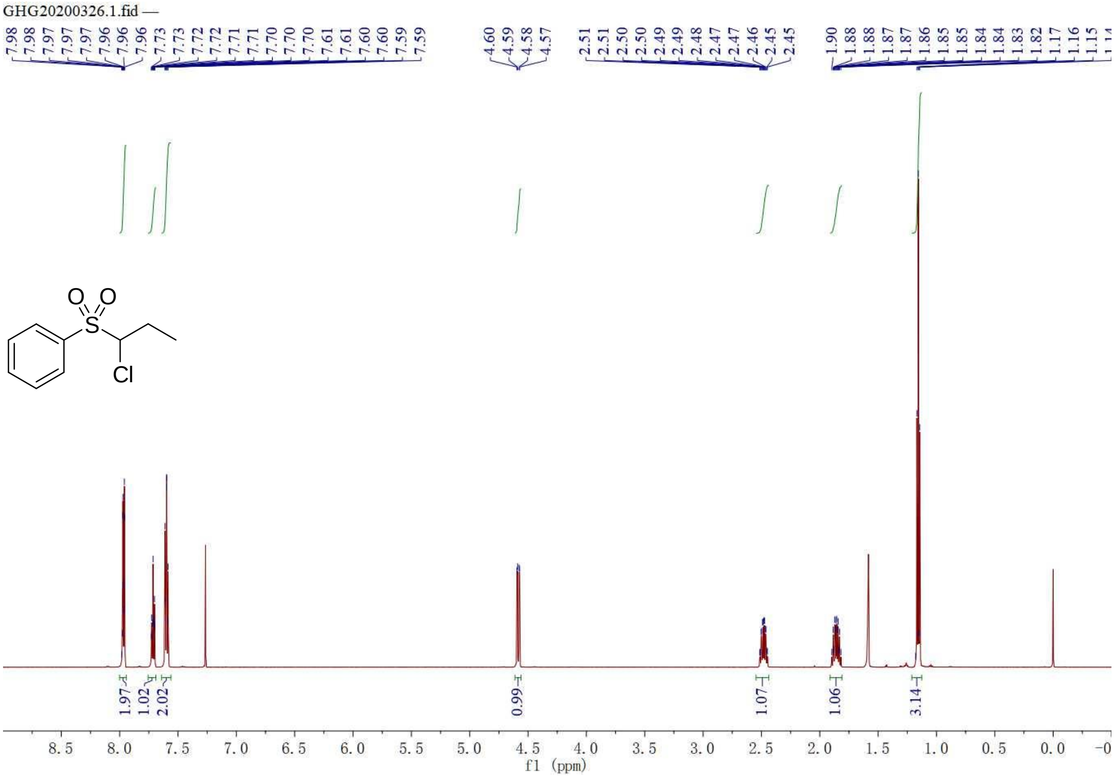

SM12, ^{13}C NMR (151 MHz, $CDCl_3$)

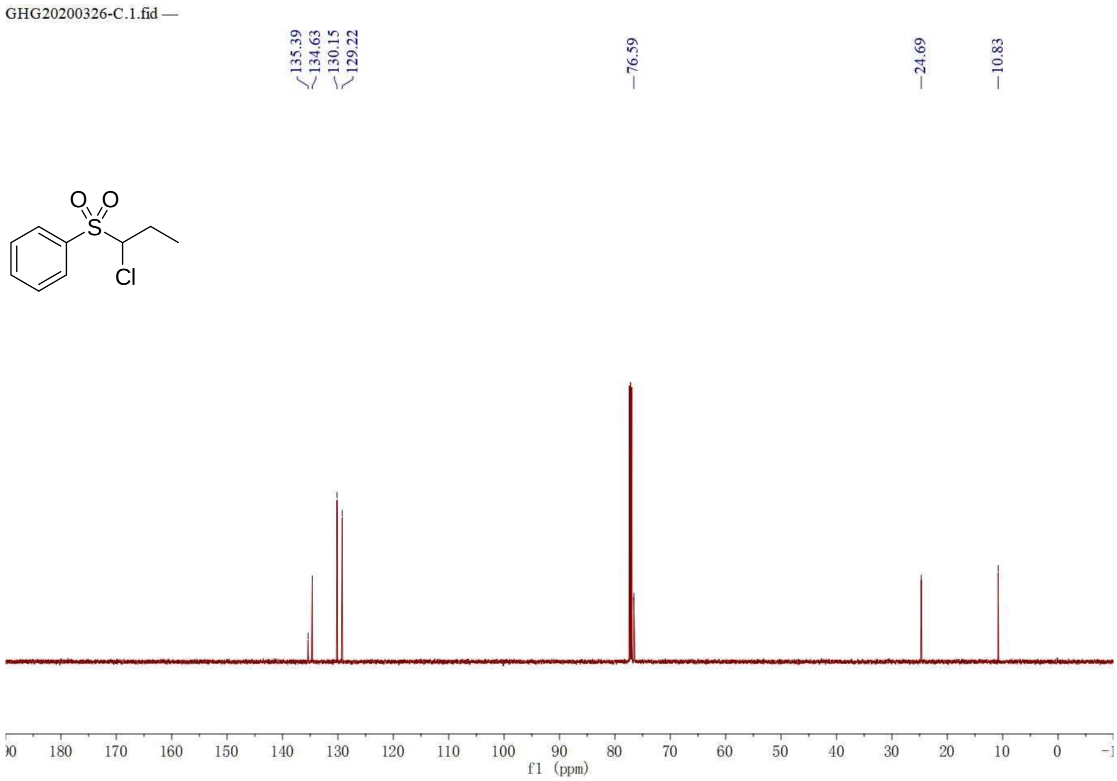

SM13, ^{1}H-NMR (600 MHZ, $CDCl_3$)

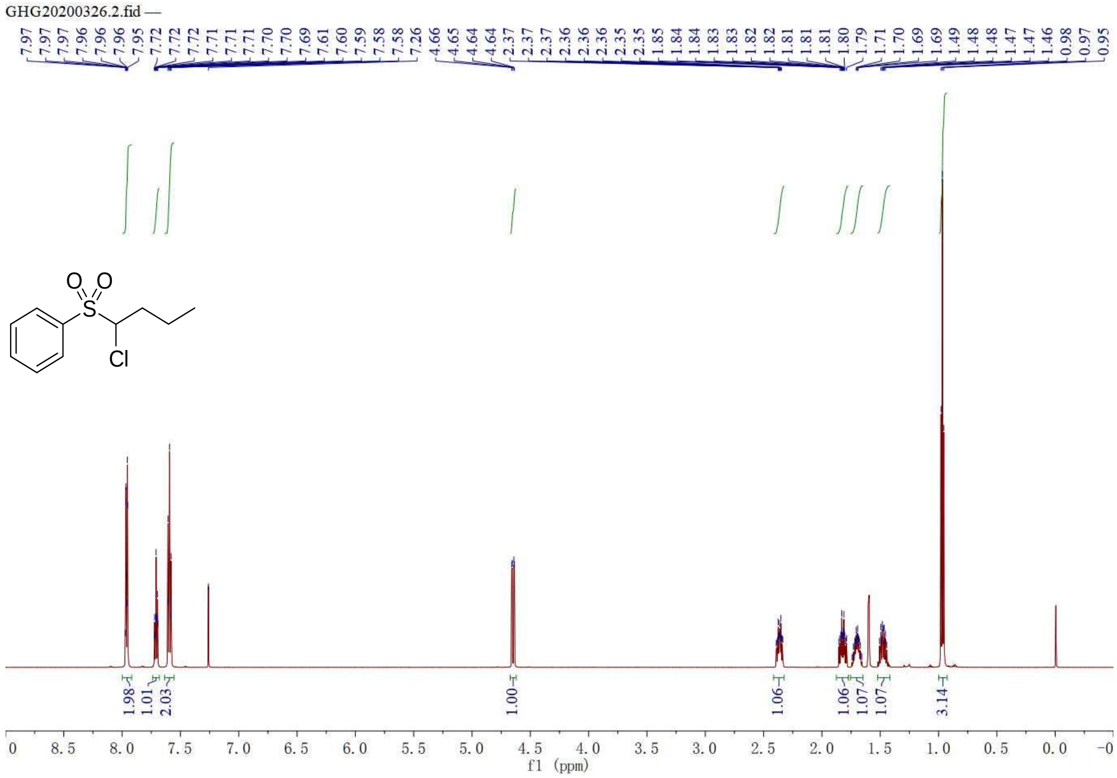

SM13, ^{13}C NMR (151 MHz, CDCl$_3$)

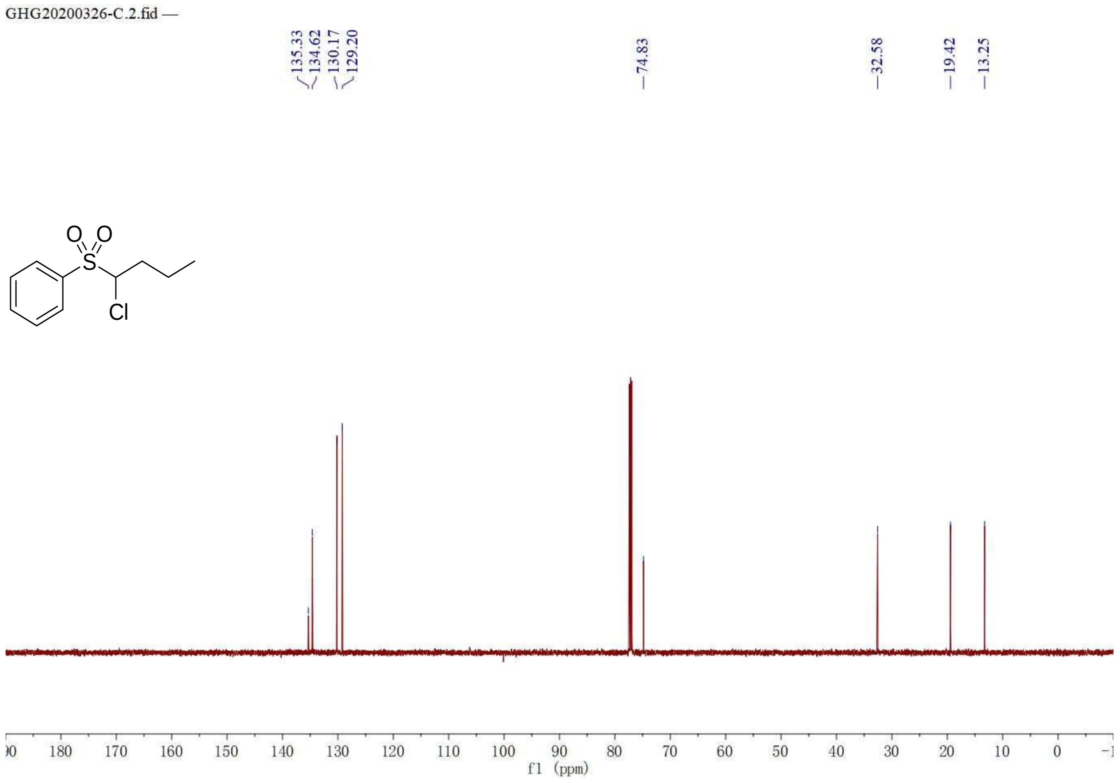

SM14, ^{1}H-NMR (600 MHZ, CDCl$_3$)

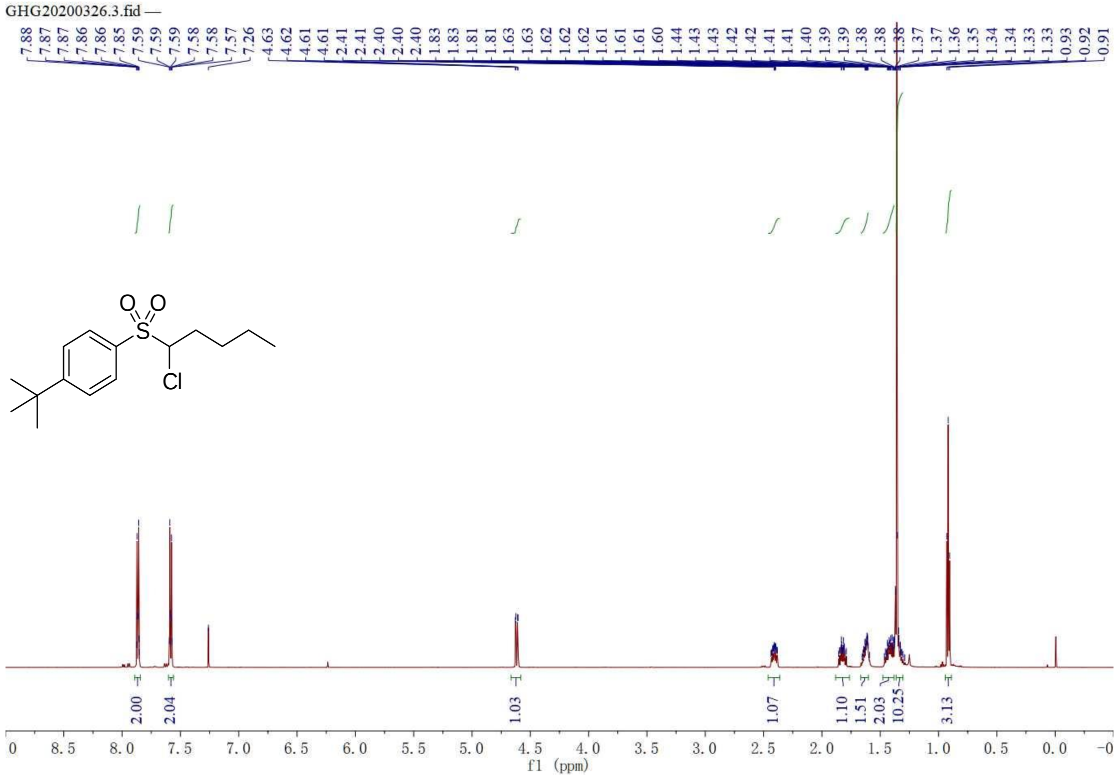

SM14, ^{13}C NMR (151 MHz, $CDCl_3$)

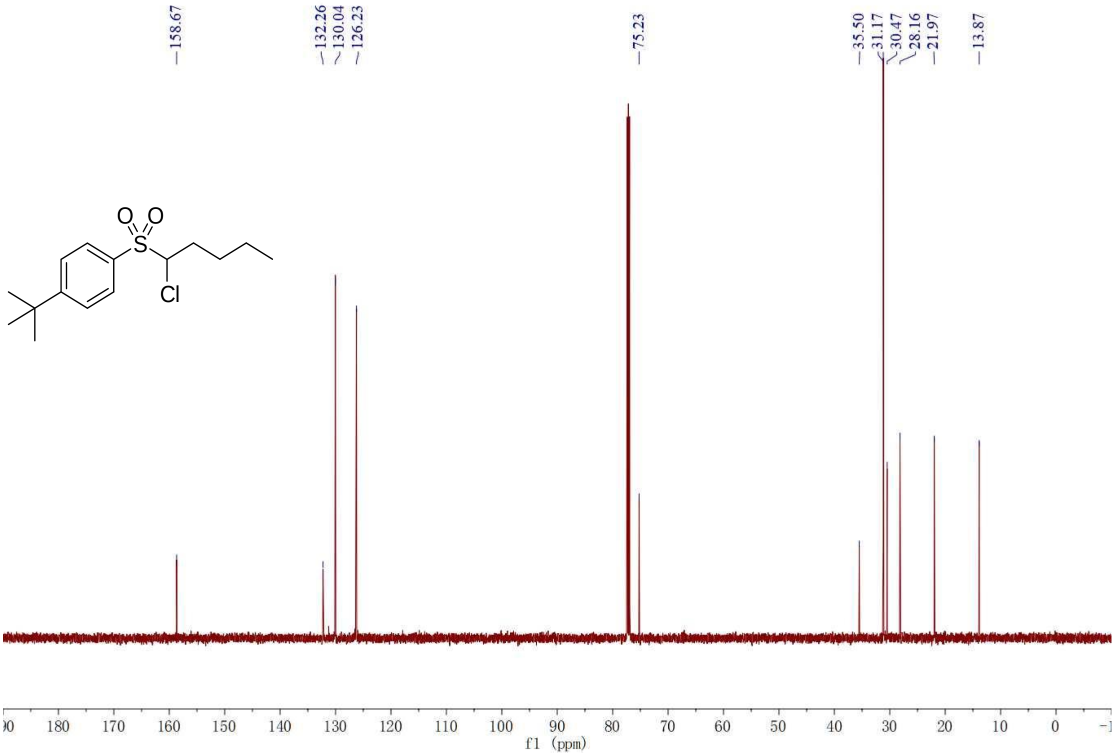

SM15, -NMR (600 MHZ, $CDCl_3$)

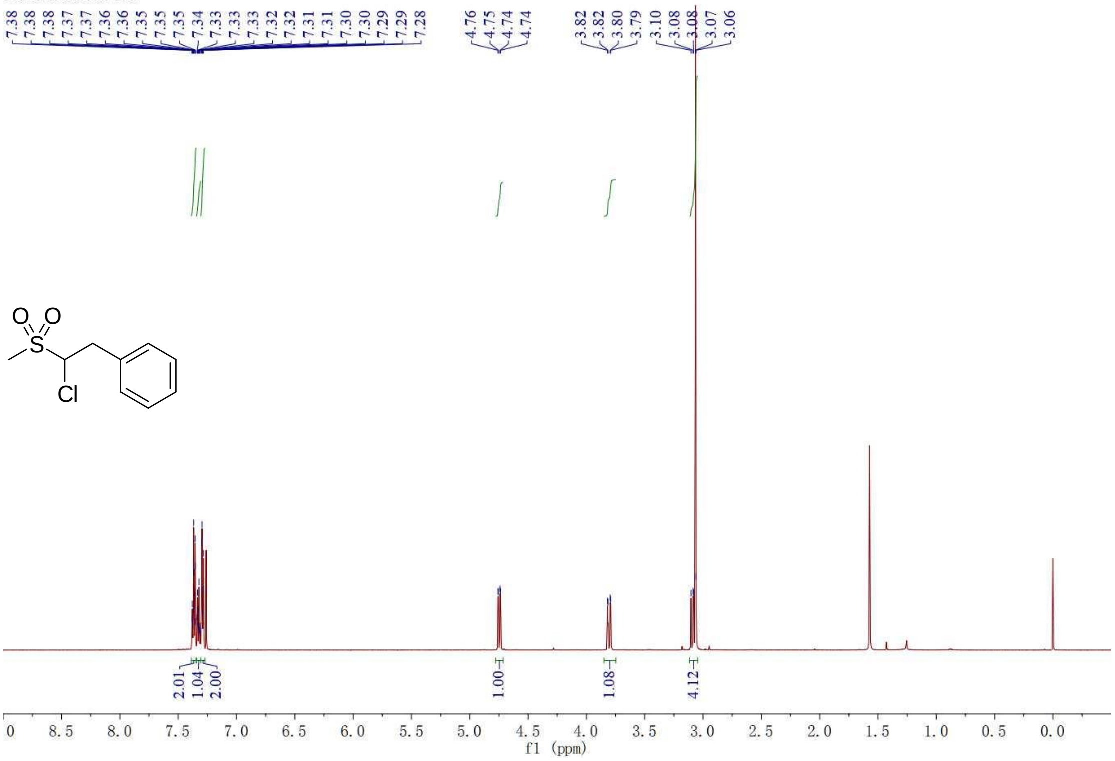

SM15, ^{13}C NMR (151 MHz, $CDCl_3$)

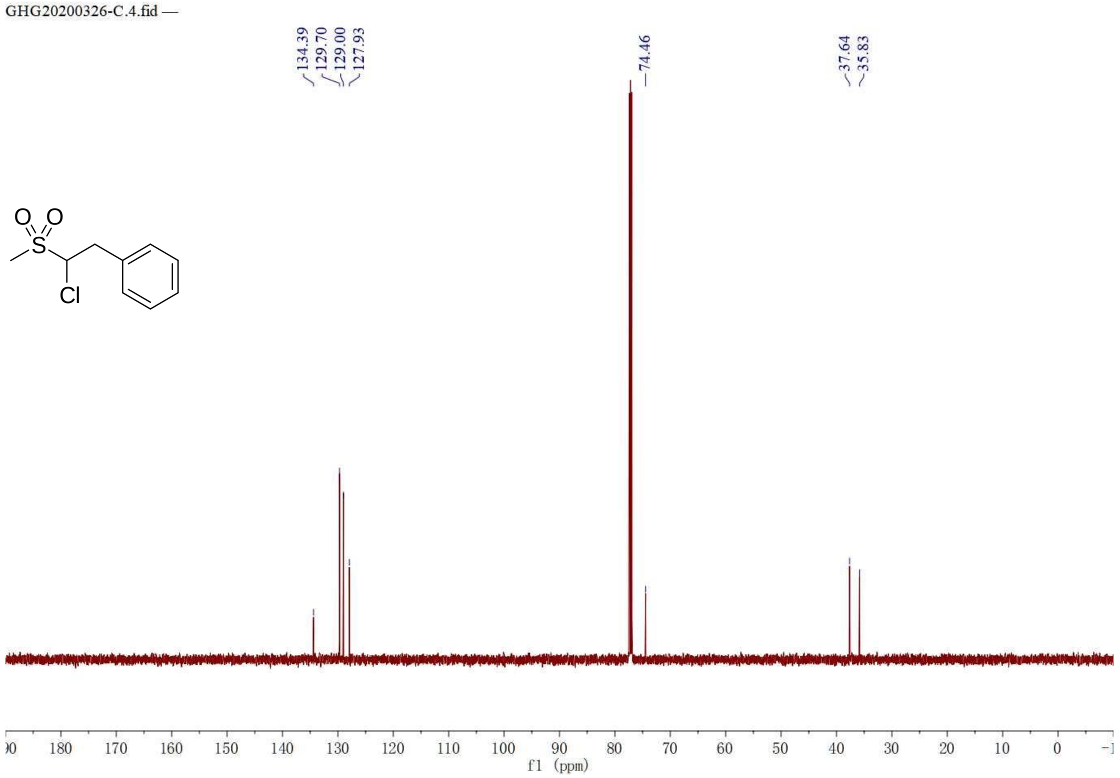

SM16, ^{1}H-NMR (600 MHZ, $CDCl_3$)

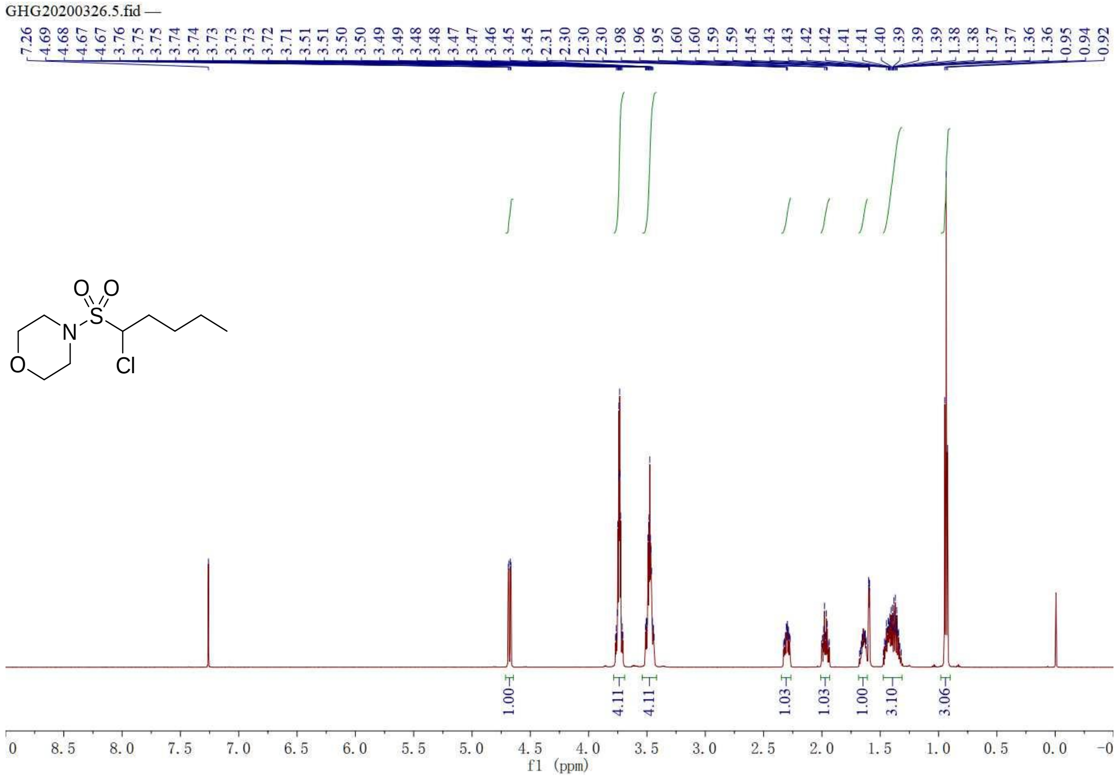

SM16, ^{13}C NMR (151 MHz, $CDCl_3$)

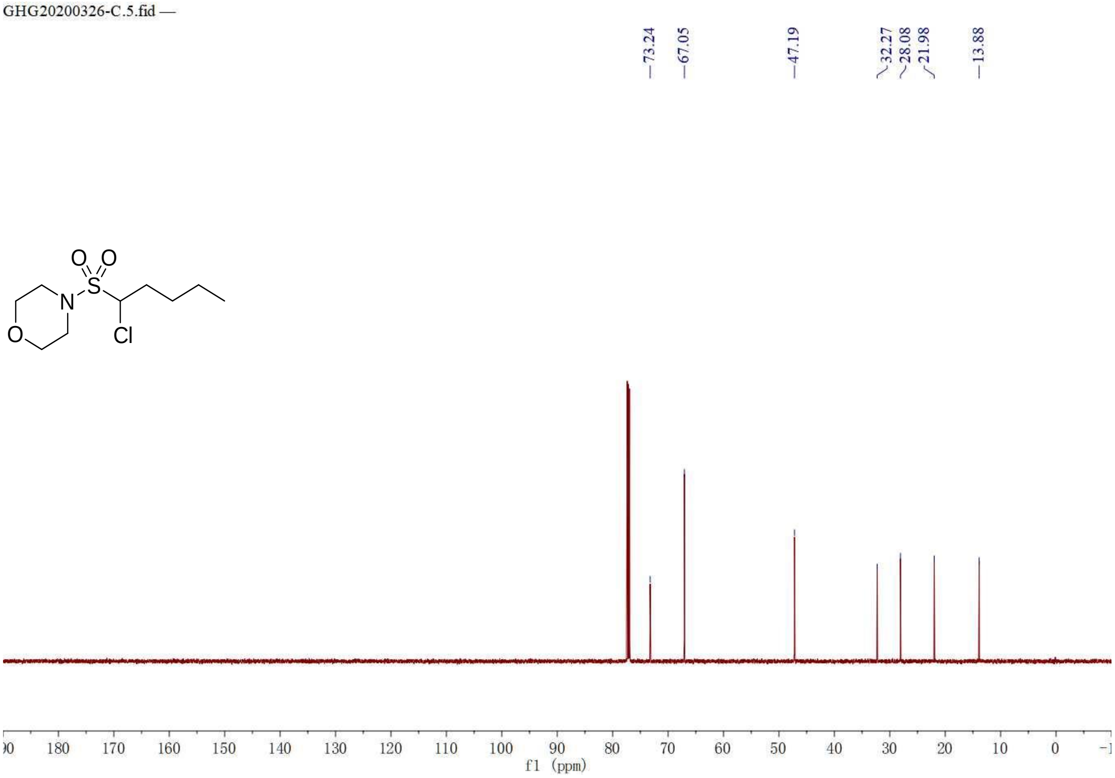

SM17, ^{1}H-NMR (600 MHZ, $CDCl_3$)

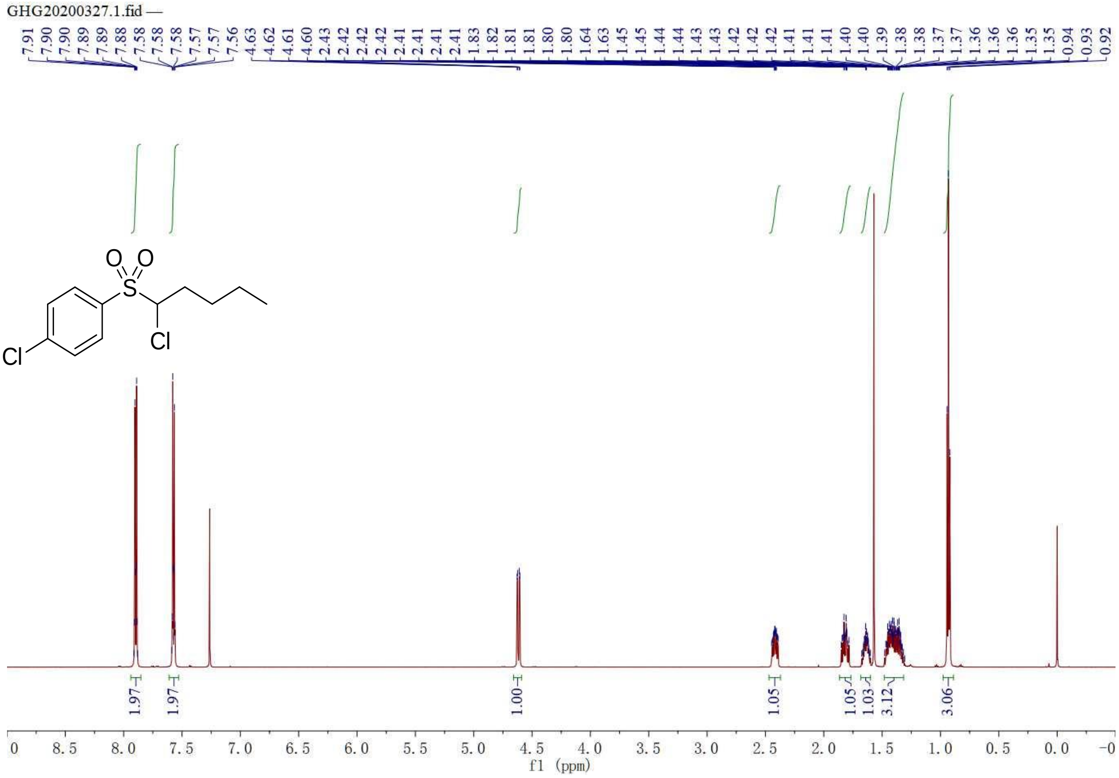

SM17, ^{13}C NMR (151 MHz, CDCl$_3$)

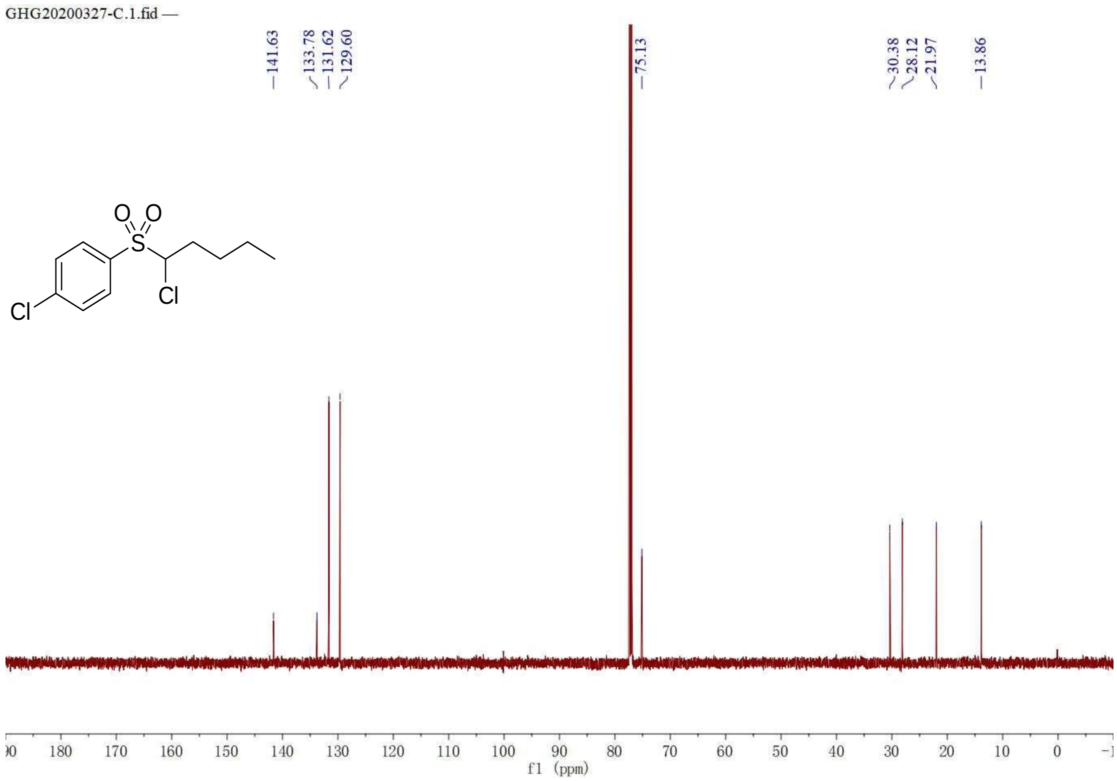

SM18, ^{1}H-NMR (600 MHZ, CDCl$_3$)

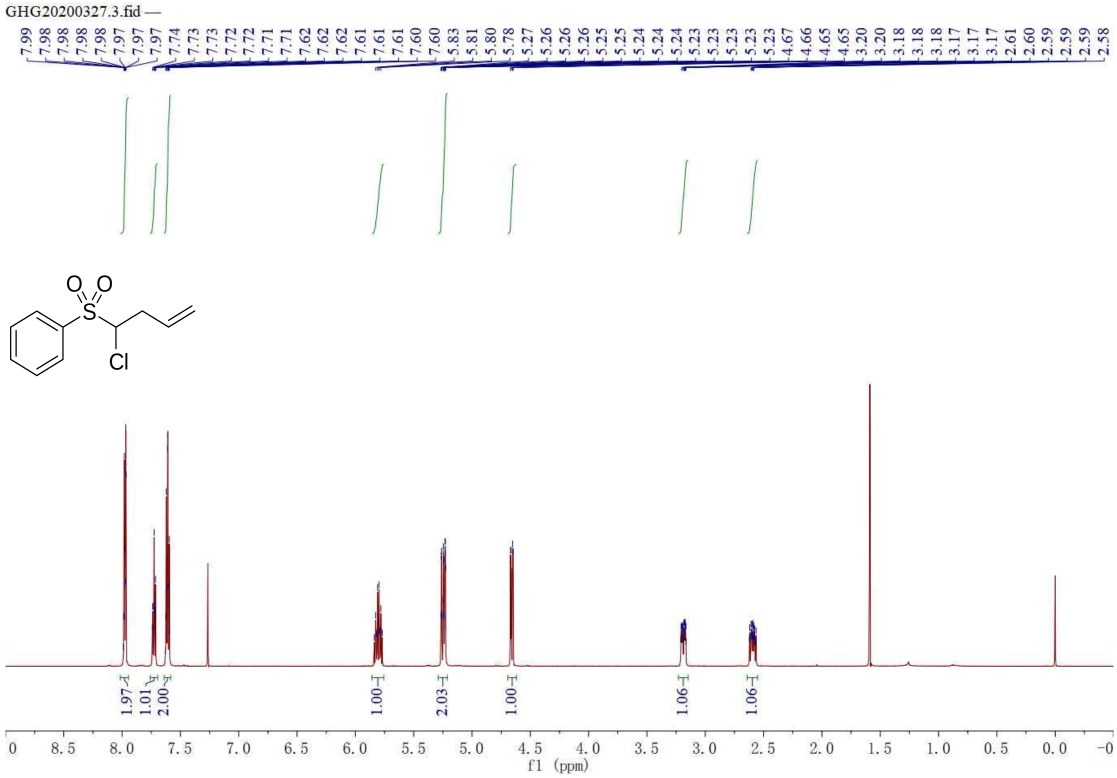

SM18, ^{13}C NMR (151 MHz, CDCl$_3$)

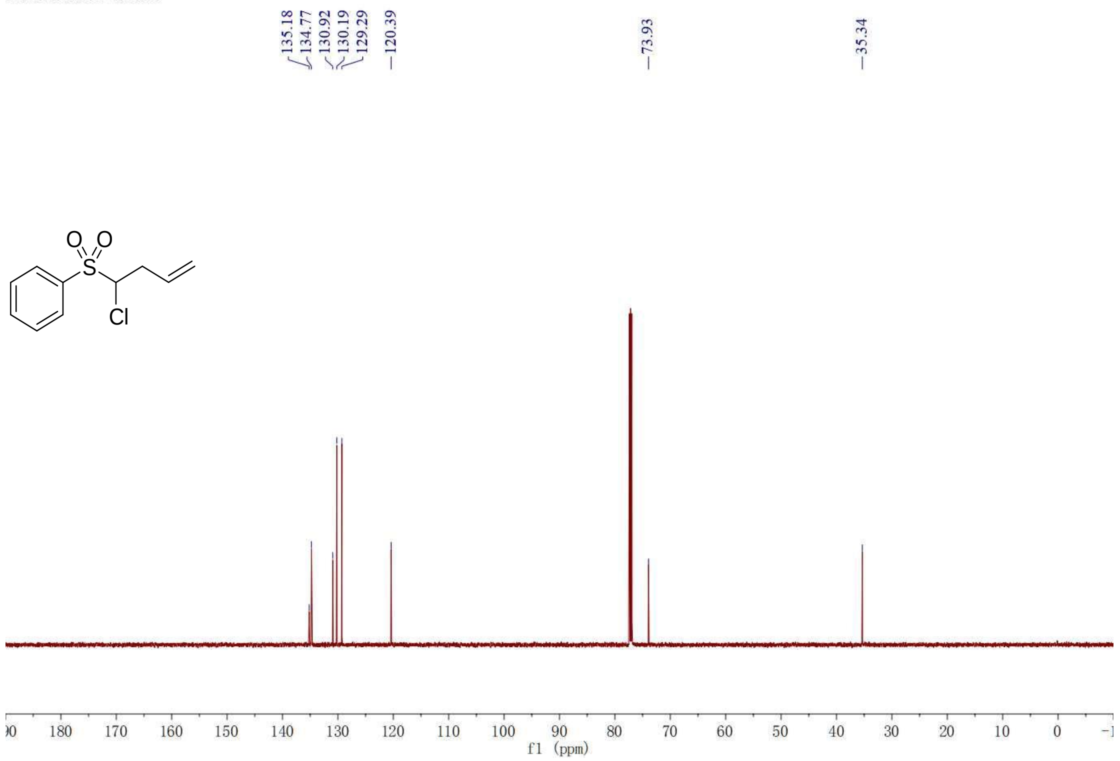

SM19, ^{1}H-NMR (600 MHZ, CDCl$_3$)

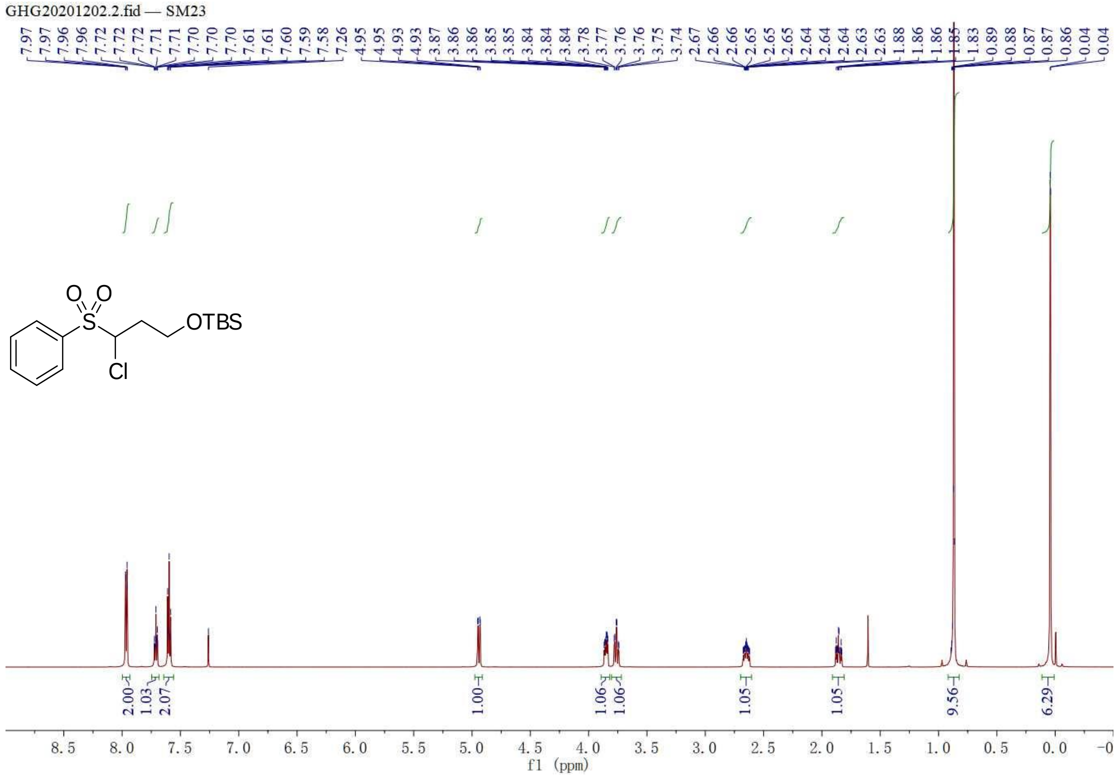

SM19, ^{13}C NMR (151 MHz, $CDCl_3$)

GHG20201202.6.fid — SM23

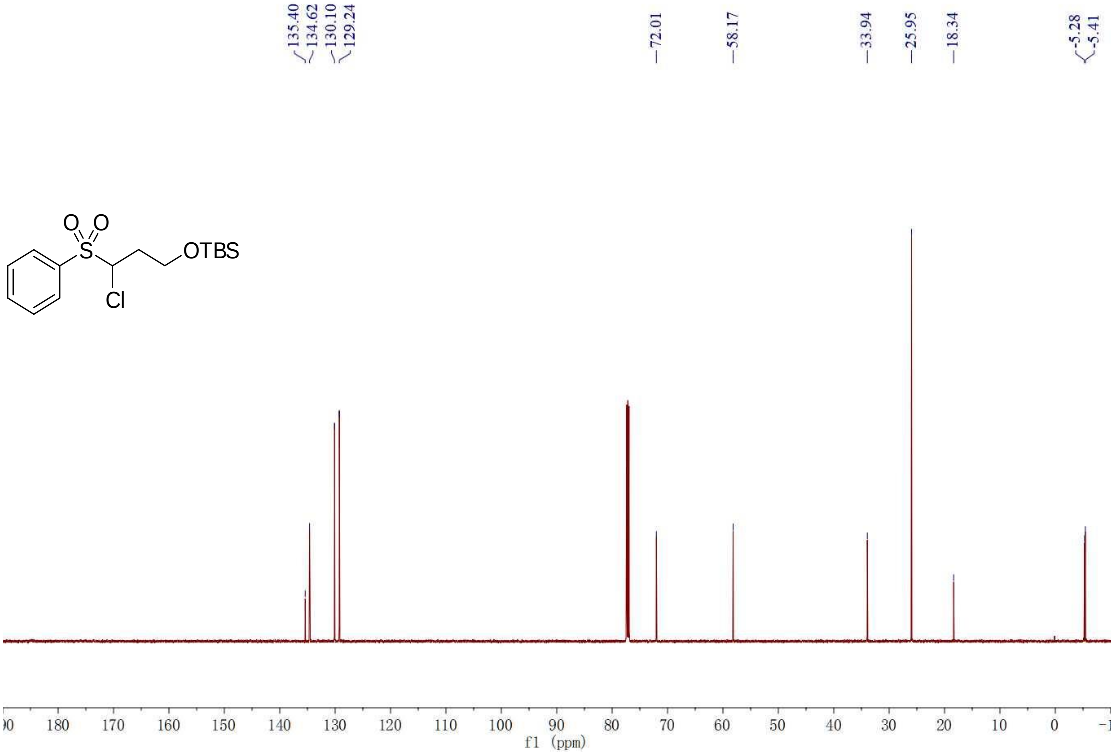

SM20, ^{1}H-NMR (600 MHZ, $CDCl_3$)

GHG20200909.7.fid — SM-OH

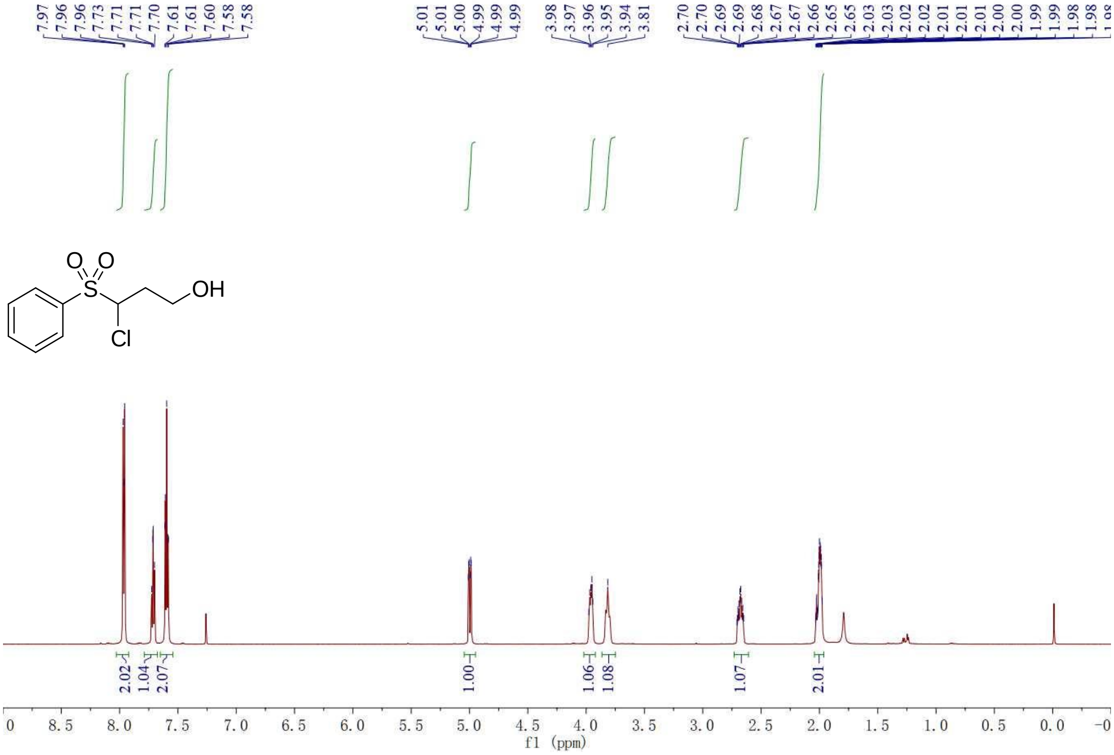

SM20, ^{13}C NMR (151 MHz, CDCl$_3$)

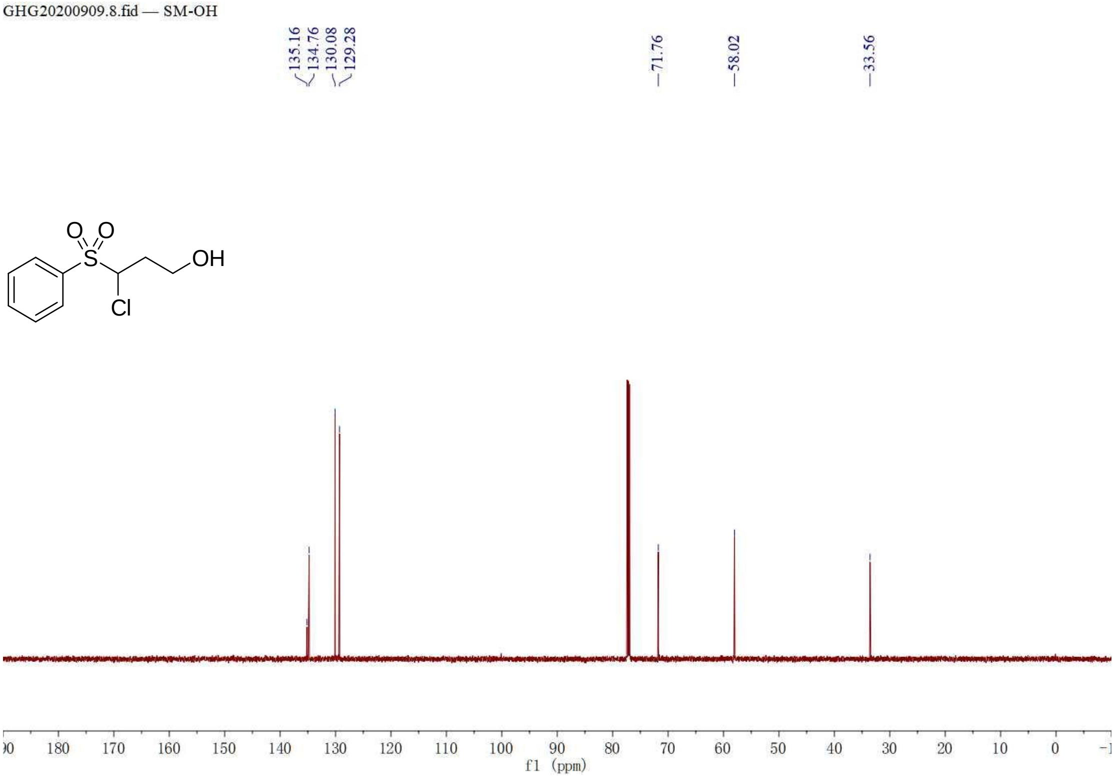

SM21, ^{1}H-NMR (600 MHZ, CDCl$_3$)

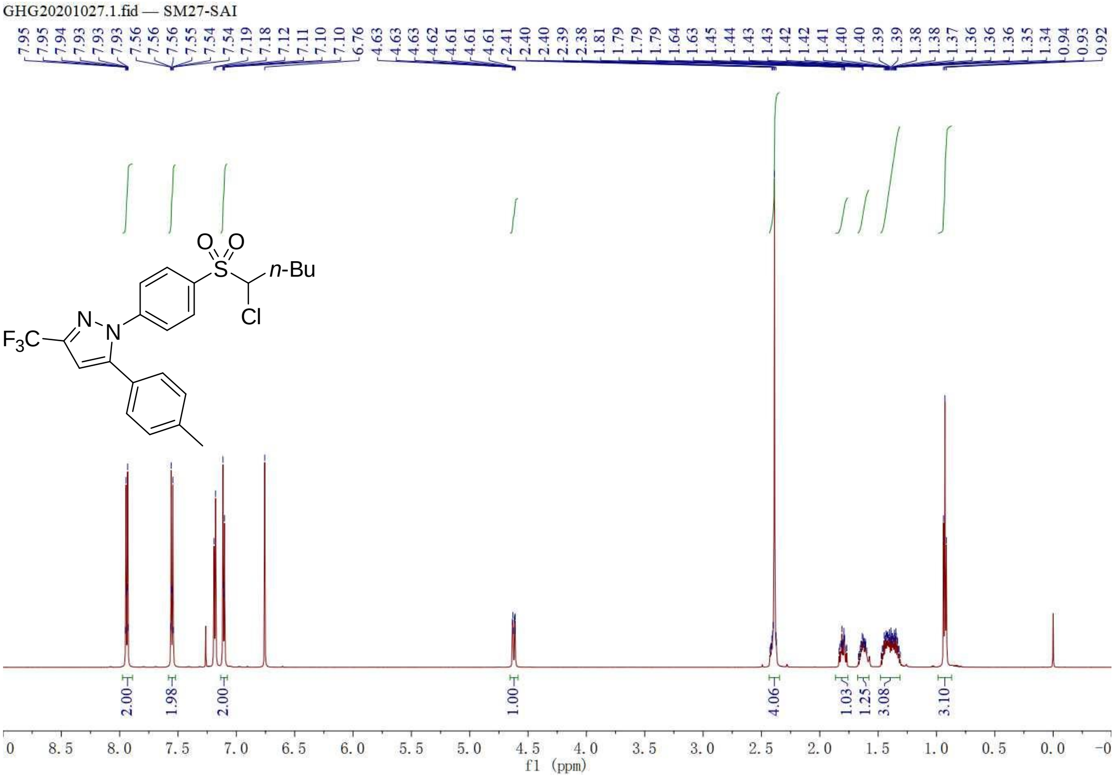

SM21, ^{13}C NMR (151 MHz, $CDCl_3$)

GHG20201027.11.fid — SM27-SAI

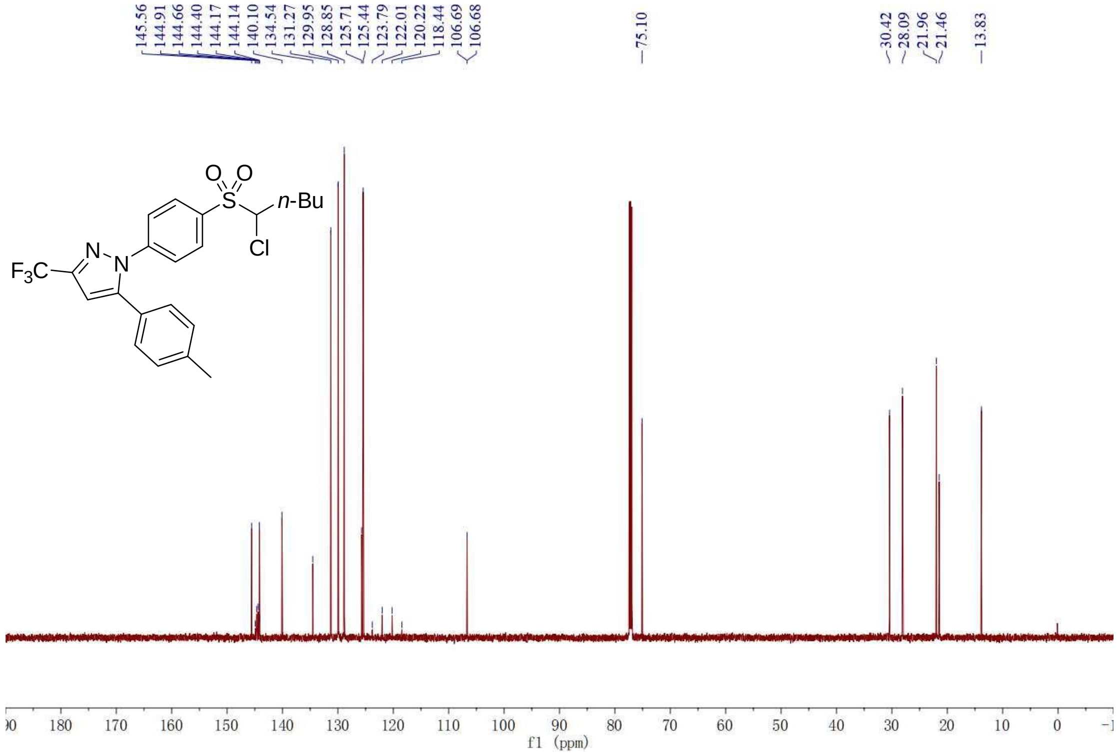

SM21, ^{19}F NMR (565 MHz, $CDCl_3$)

GHG20201027.10.fid — SM27-SAI

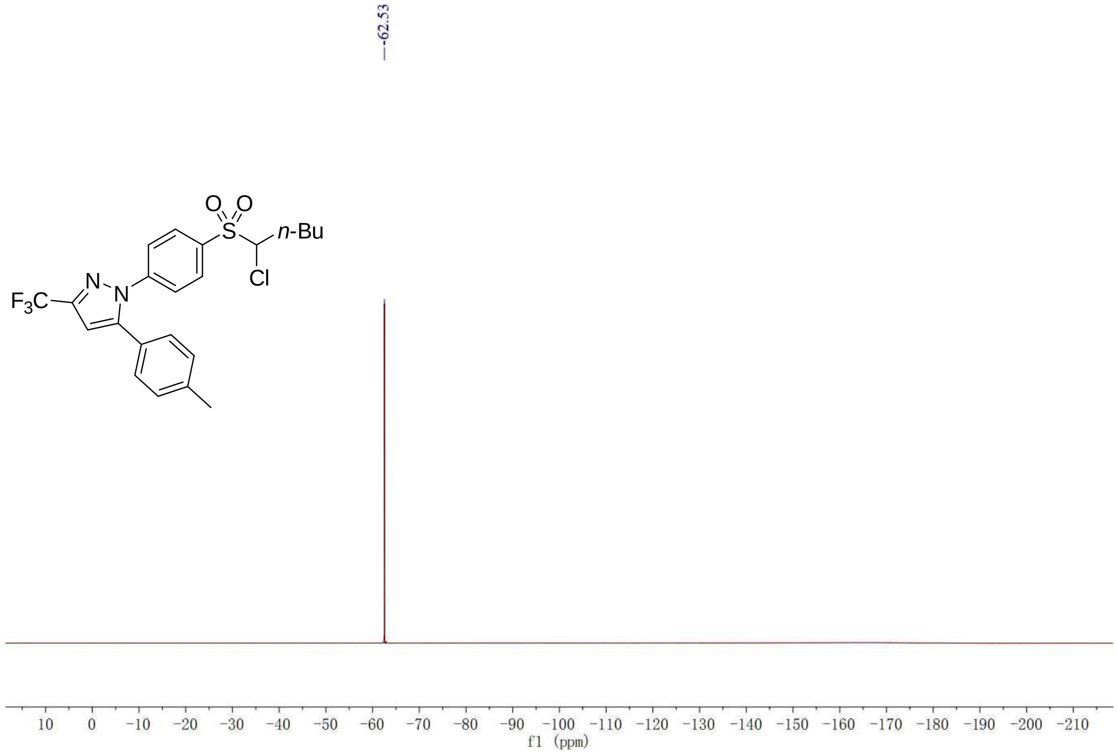

70, ^{1}H-NMR (600 MHZ, $CDCl_3$)

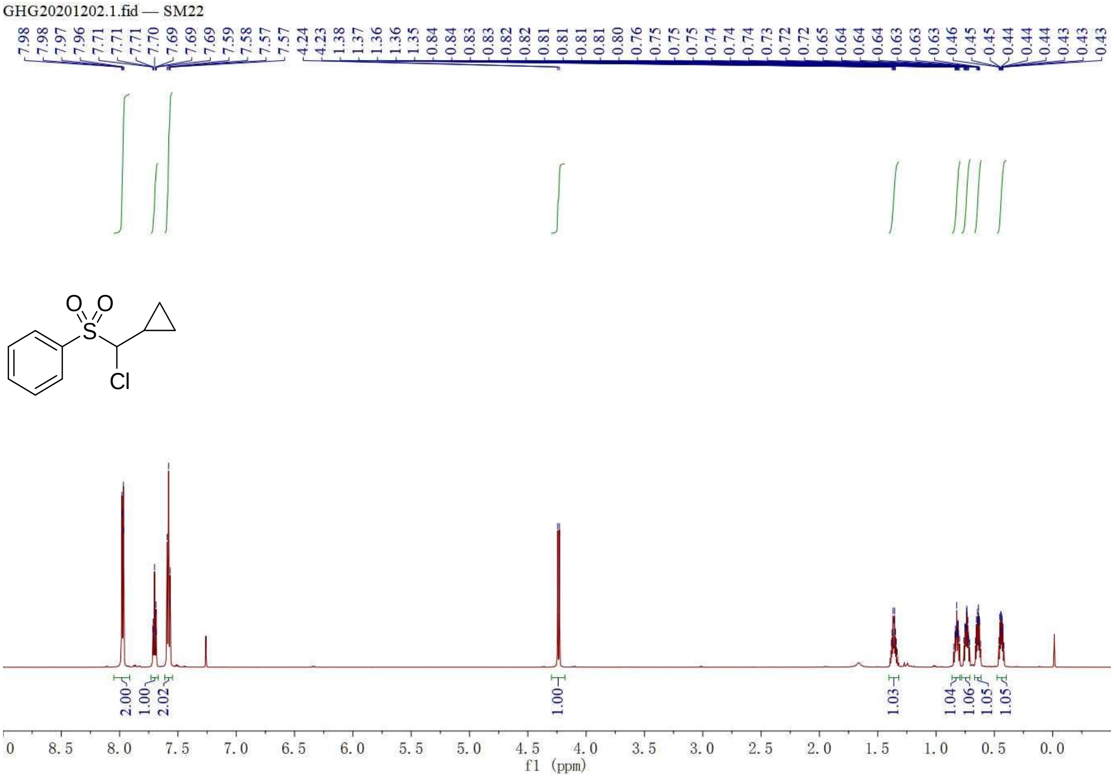

70, ^{13}C NMR (151 MHz, $CDCl_3$)

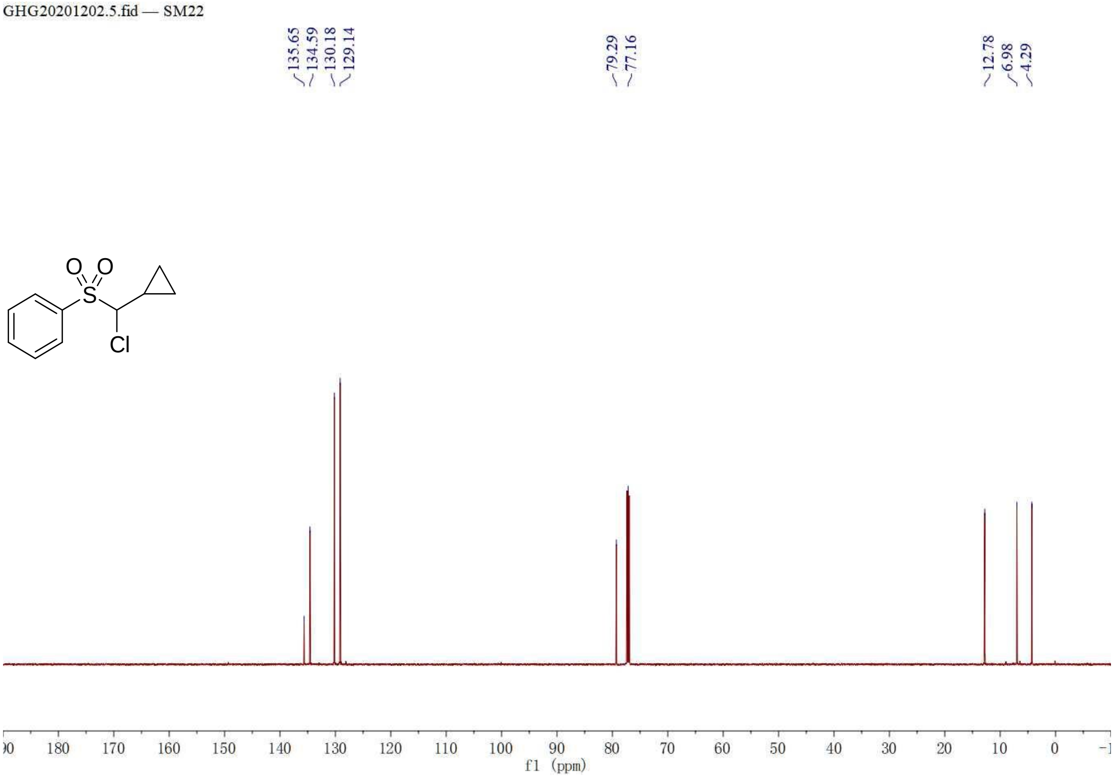

3, ^{1}H-NMR (600 MHZ, $CDCl_3$)

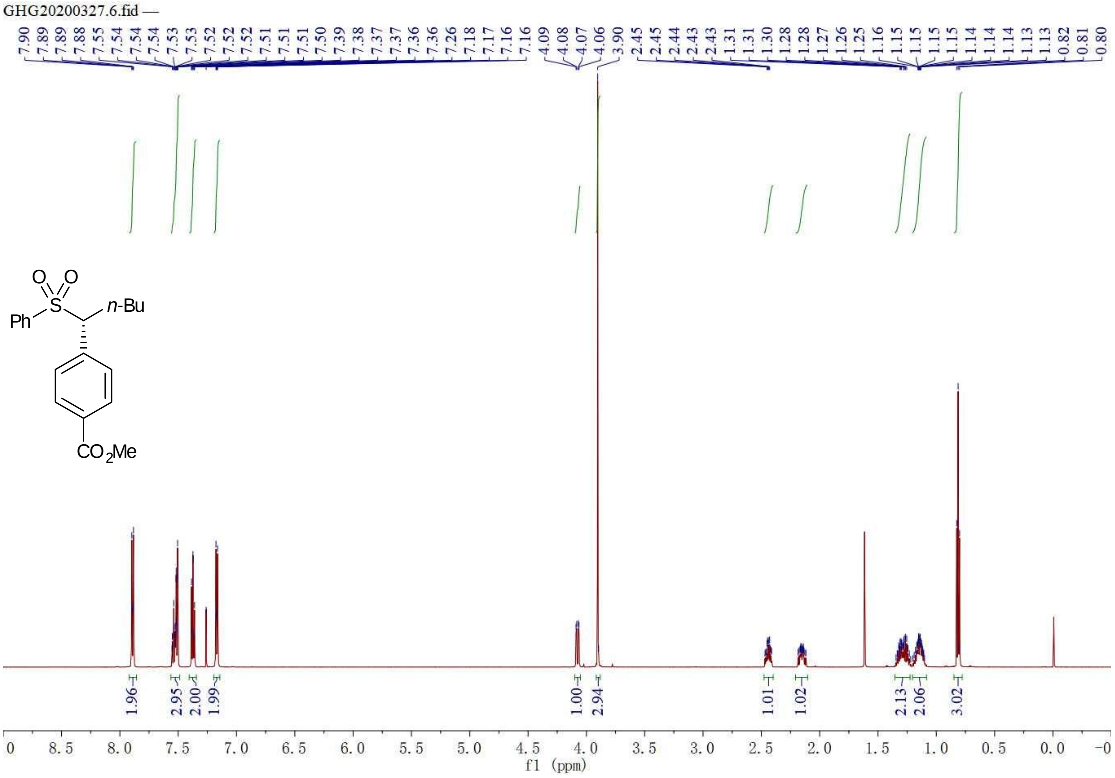

3, ^{13}C NMR (151 MHz, $CDCl_3$)

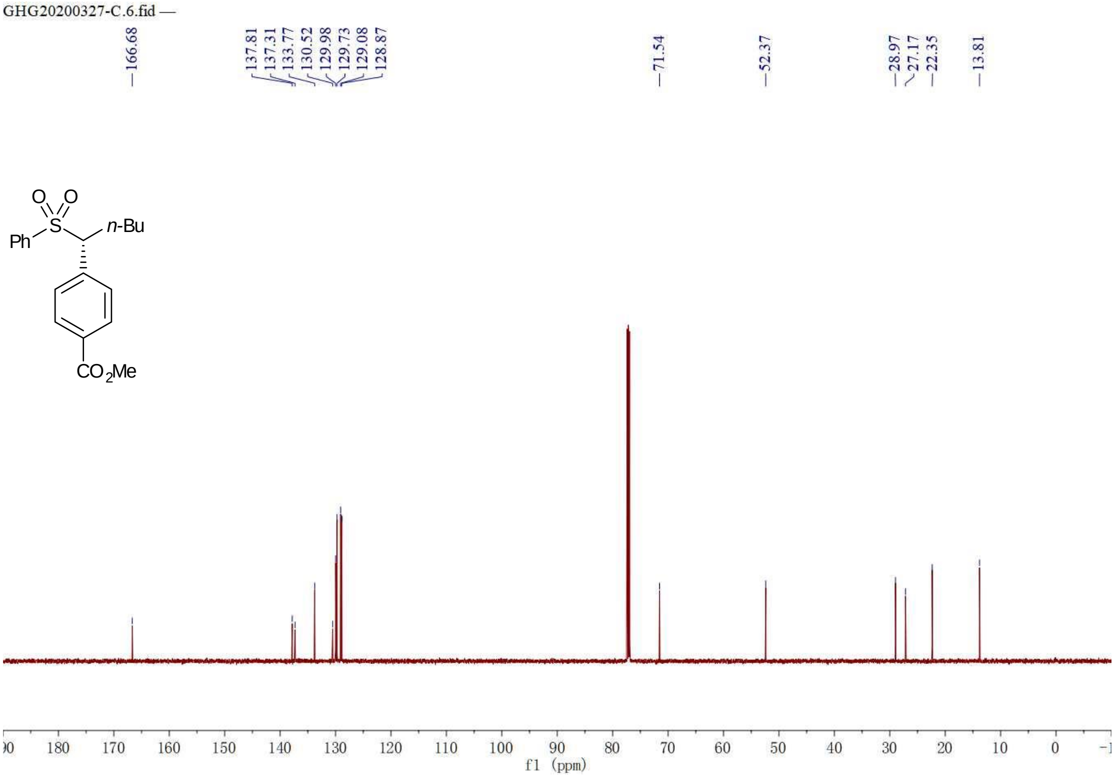

4, ^{1}H-NMR (600 MHZ, $CDCl_3$)

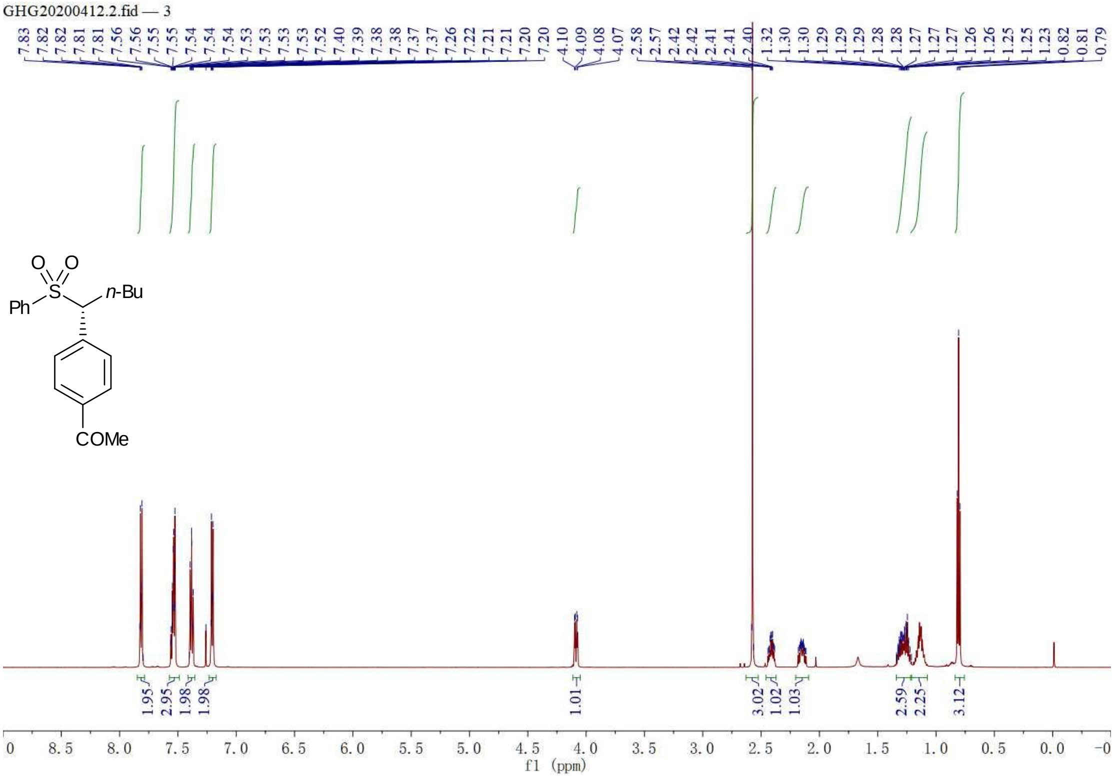

4, ^{13}C NMR (151 MHz, $CDCl_3$)

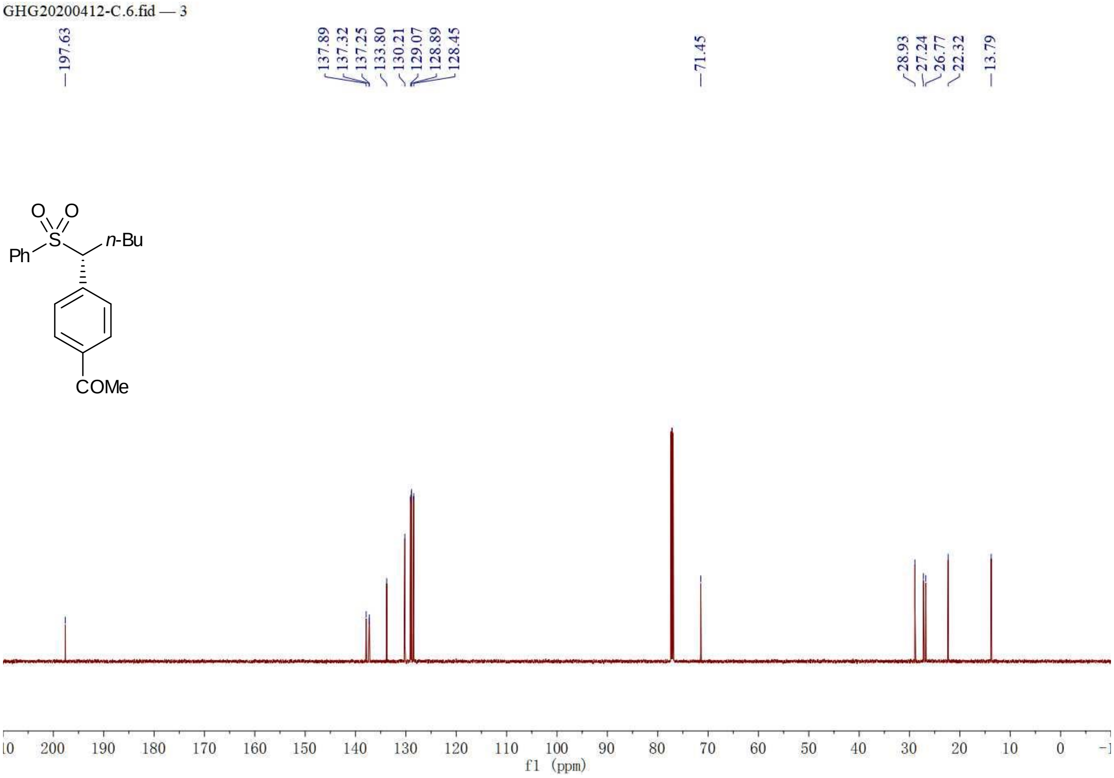

5, ^{1}H-NMR (600 MHZ, CDCl$_3$)

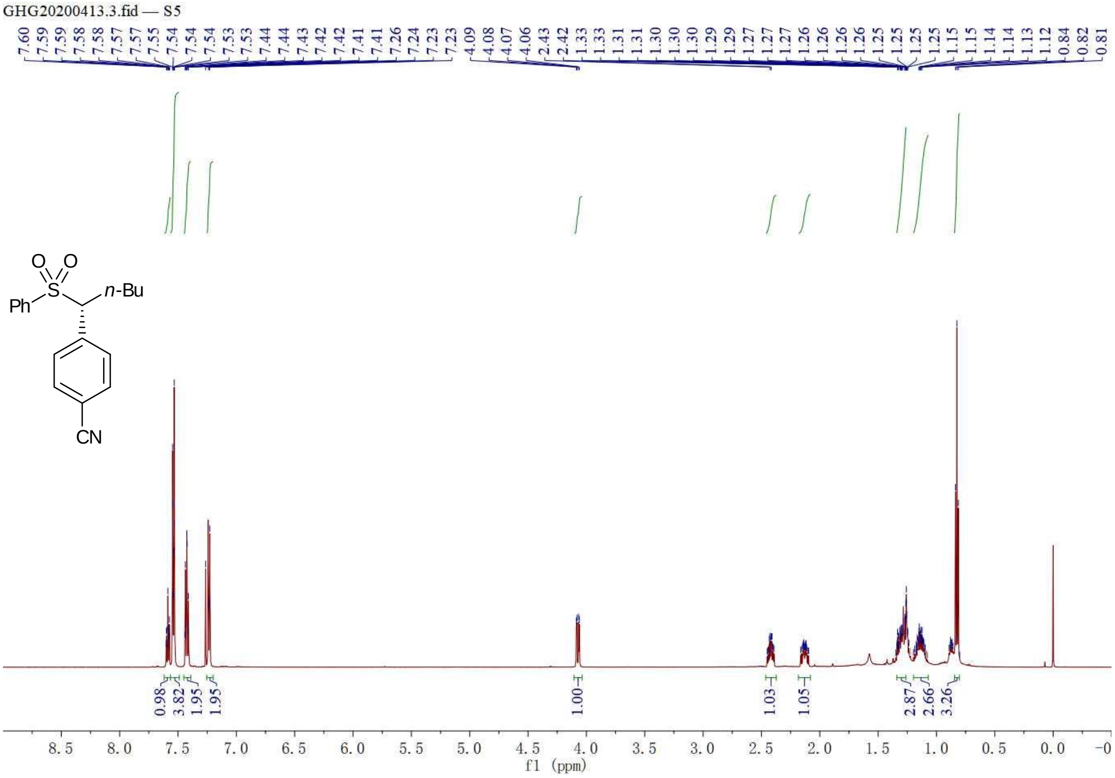

5, ^{13}C NMR (151 MHz, CDCl$_3$)

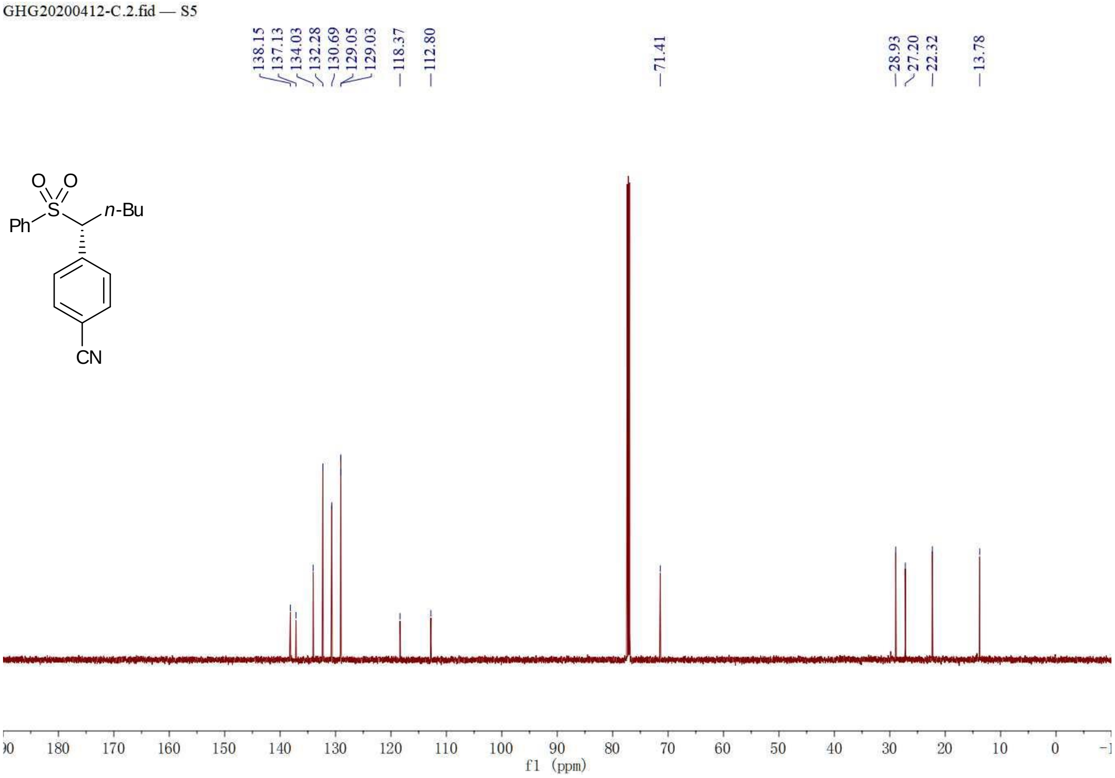

6, ^{1}H-NMR (600 MHZ, $CDCl_3$)

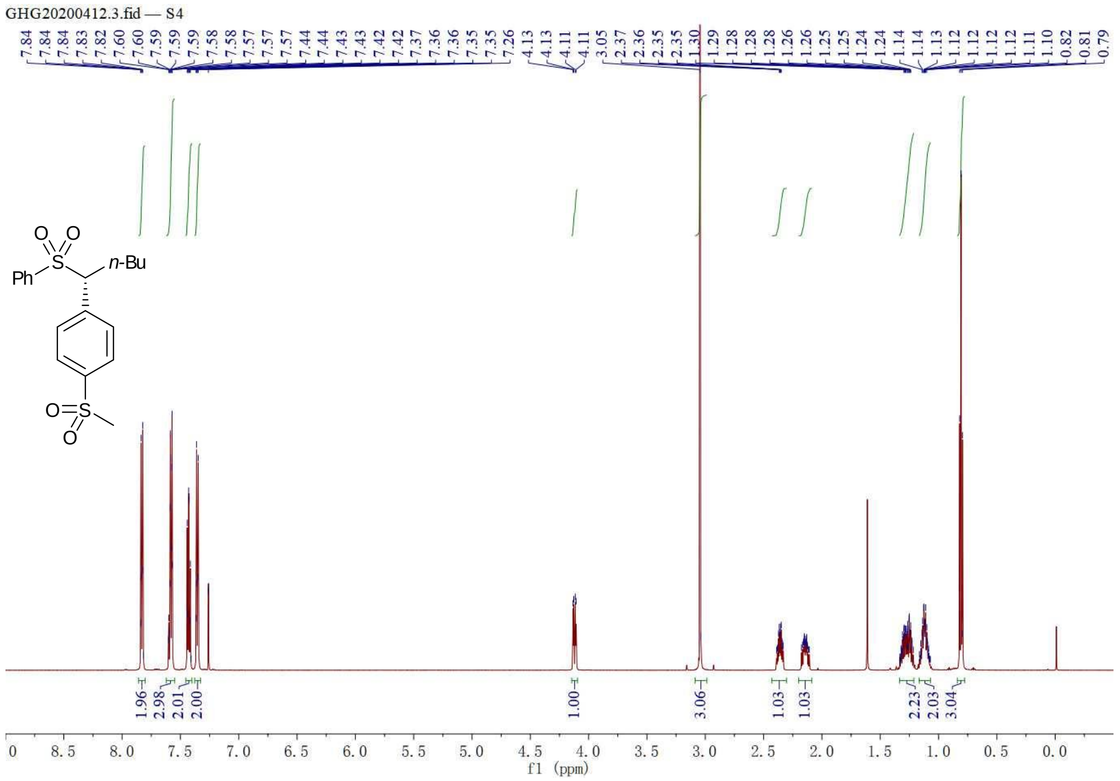

6, ^{13}C NMR (151 MHz, $CDCl_3$)

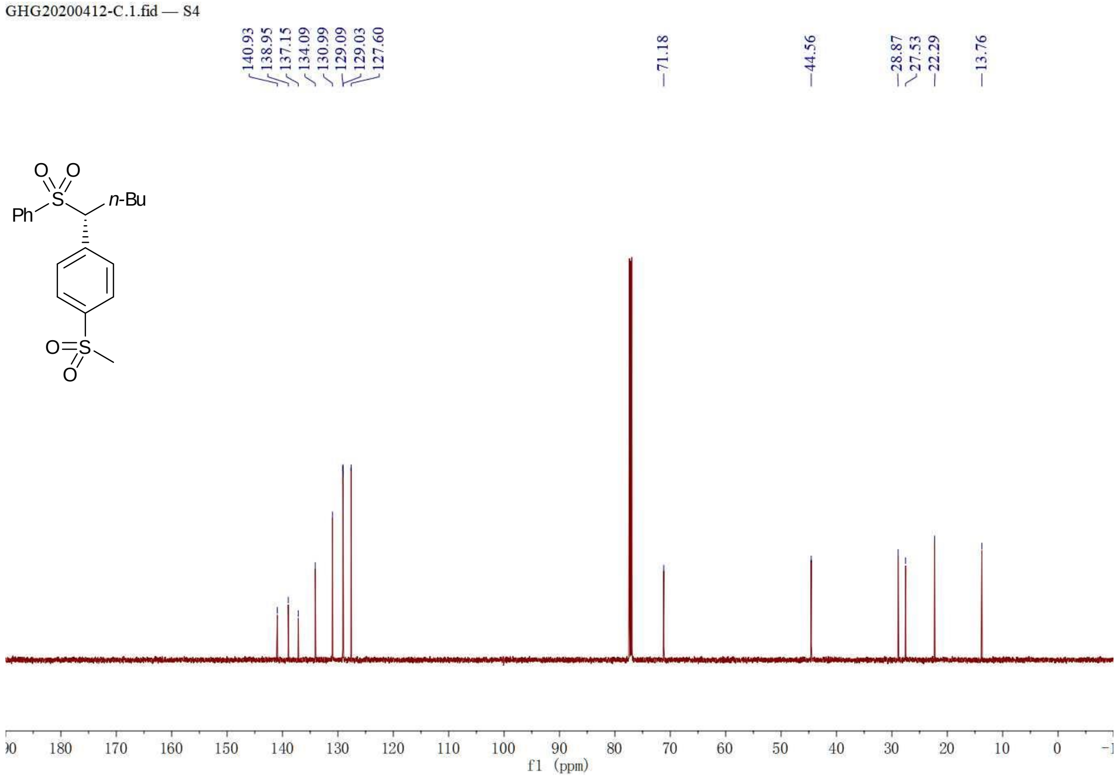

7, ^{1}H-NMR (600 MHZ, CDCl$_3$)

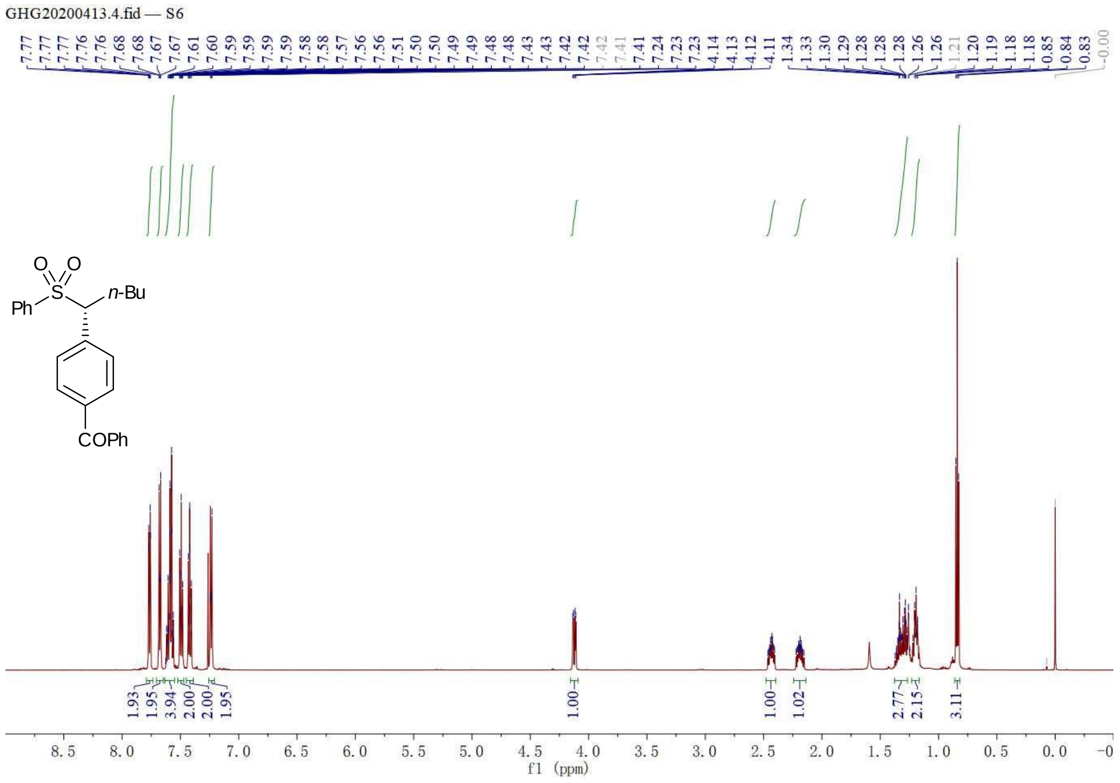

7, ^{13}C NMR (151 MHz, CDCl$_3$)

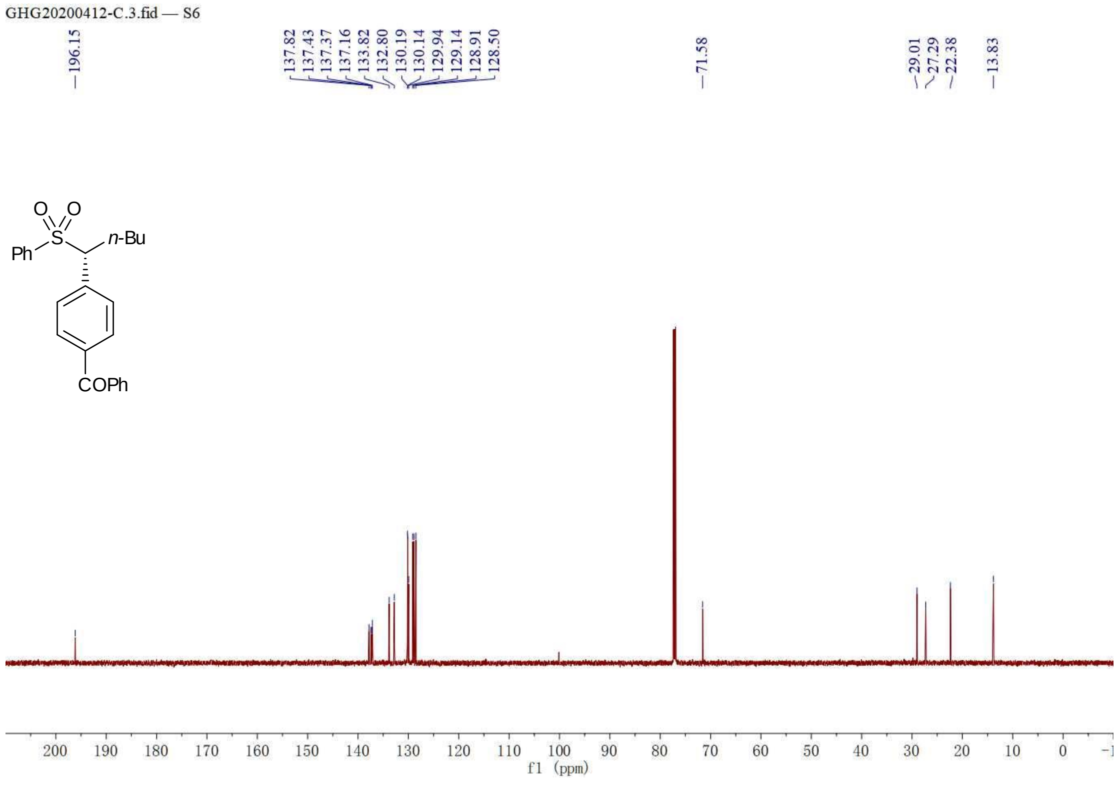

8, ^{1}H-NMR (600 MHZ, $CDCl_3$)

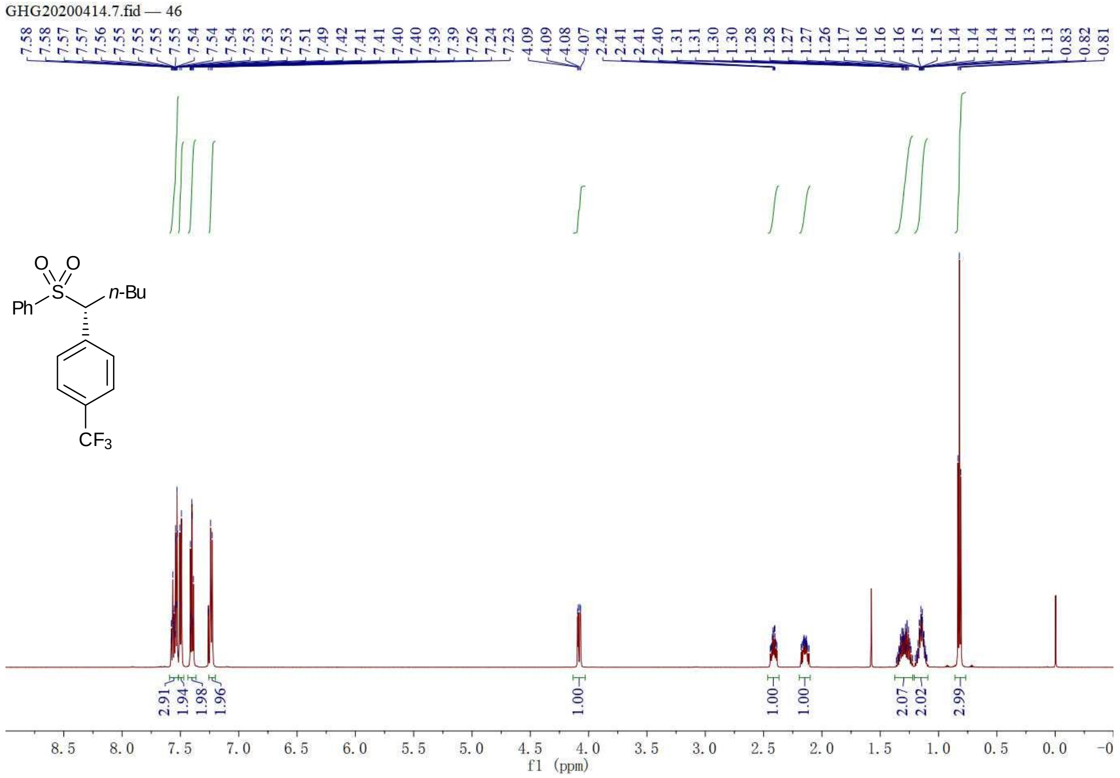

8, ^{13}C NMR (151 MHz, $CDCl_3$)

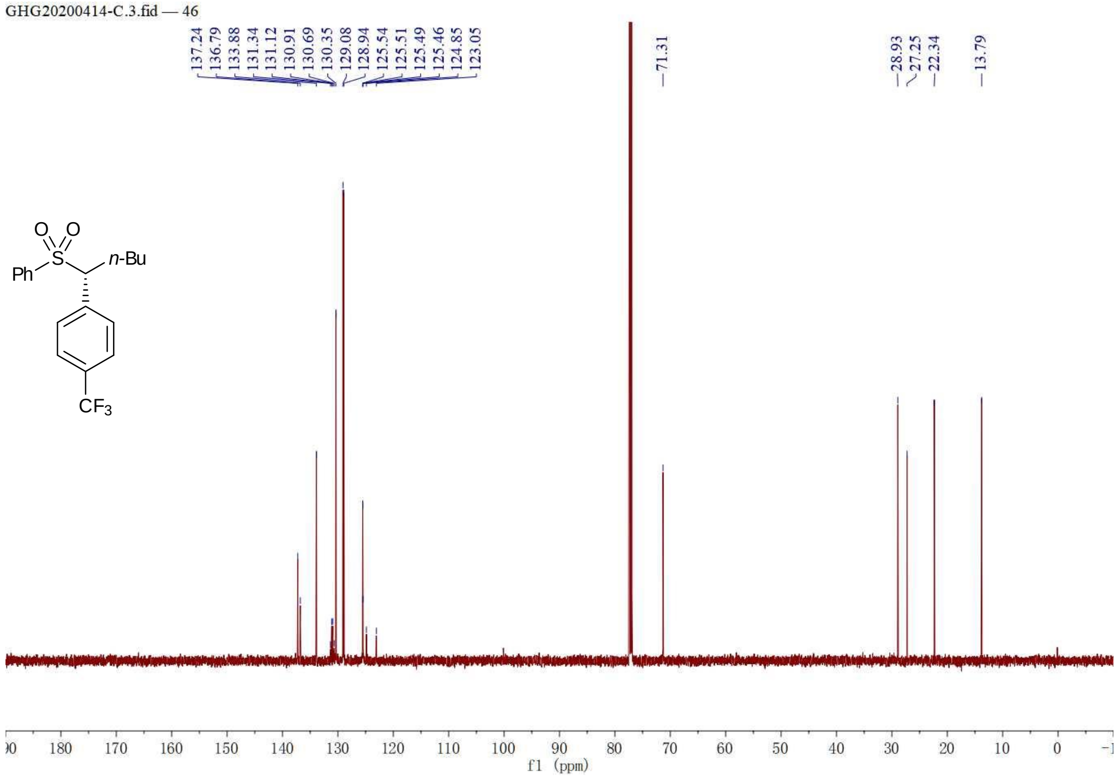

8, ^{19}F NMR (567 MHz, CDCl$_3$)

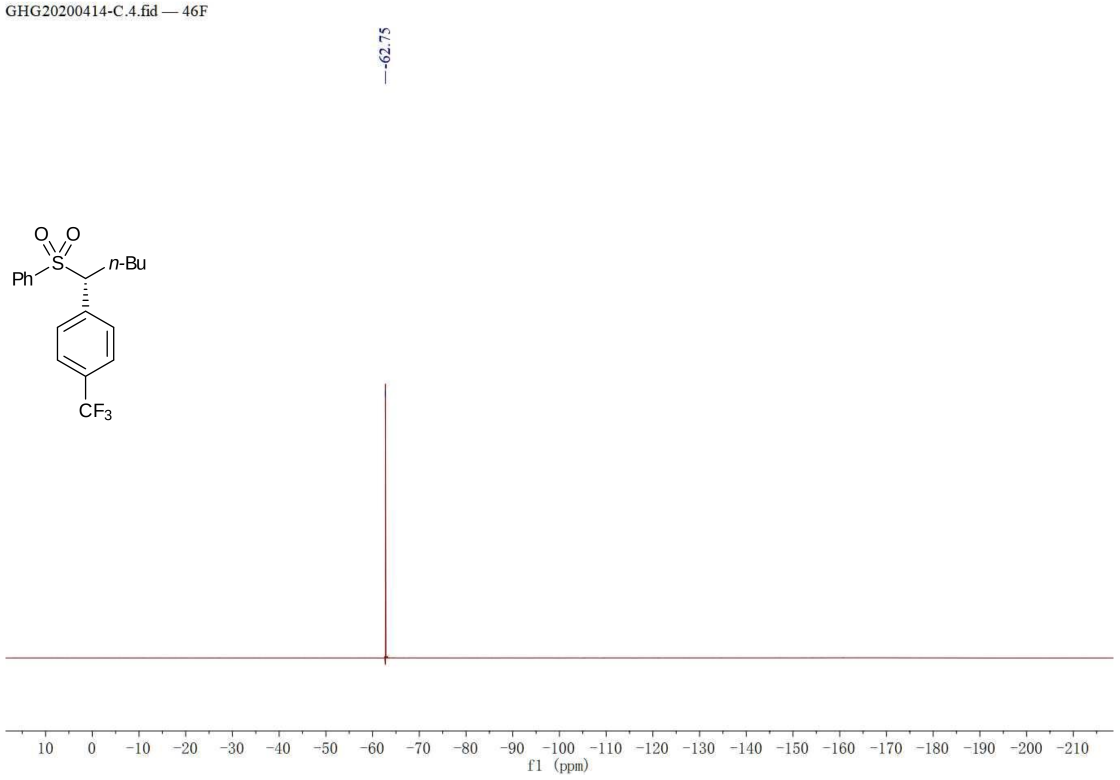

9, ^{1}H-NMR (600 MHZ, CDCl$_3$)

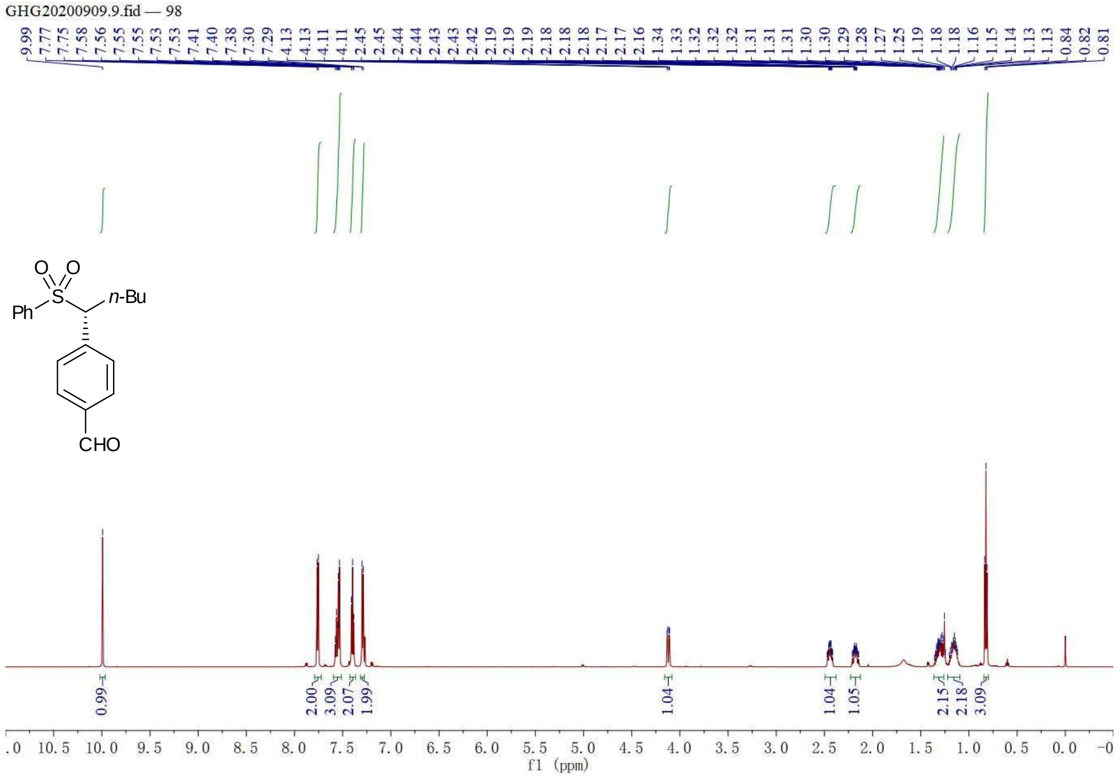

9, ^{13}C NMR (151 MHz, $CDCl_3$)

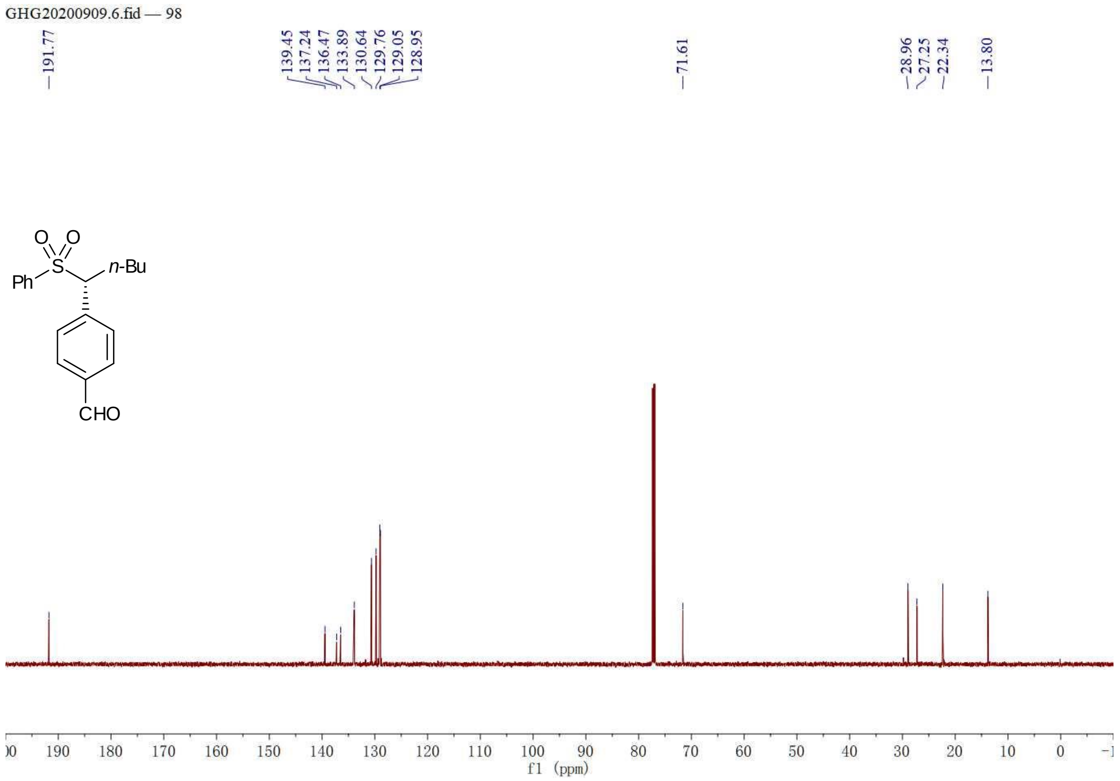

10, ^{1}H-NMR (600 MHZ, $CDCl_3$)

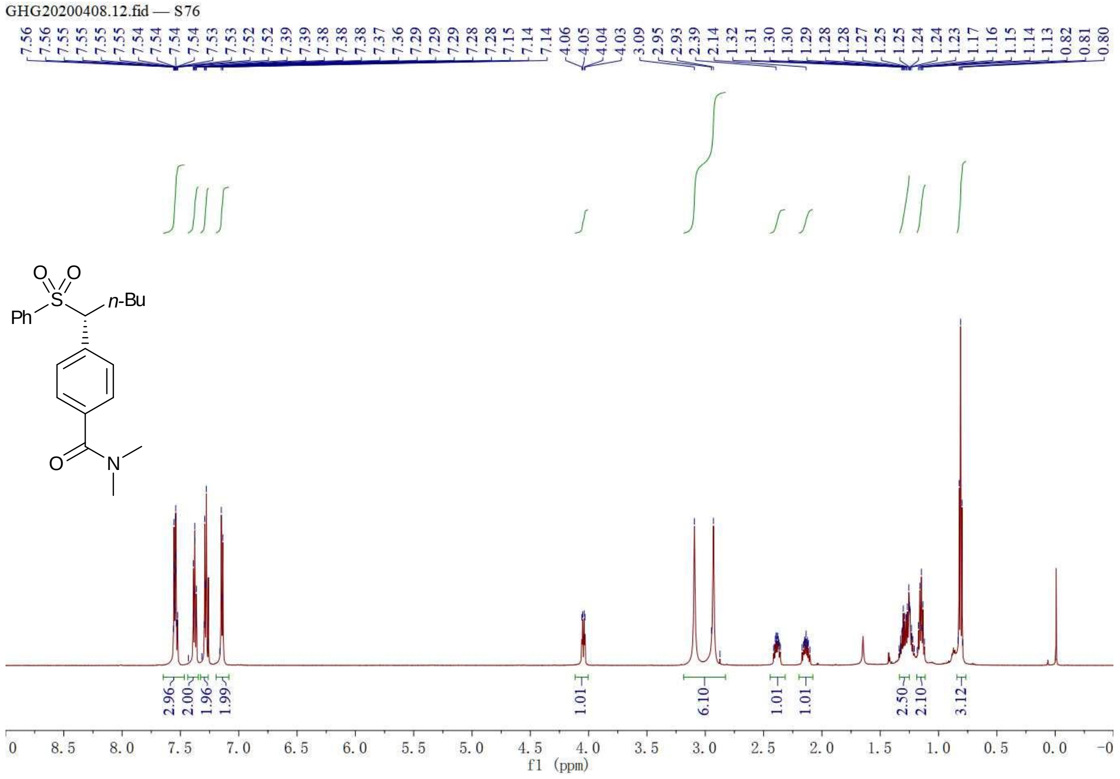

10, ^{13}C NMR (151 MHz, CDCl$_3$)

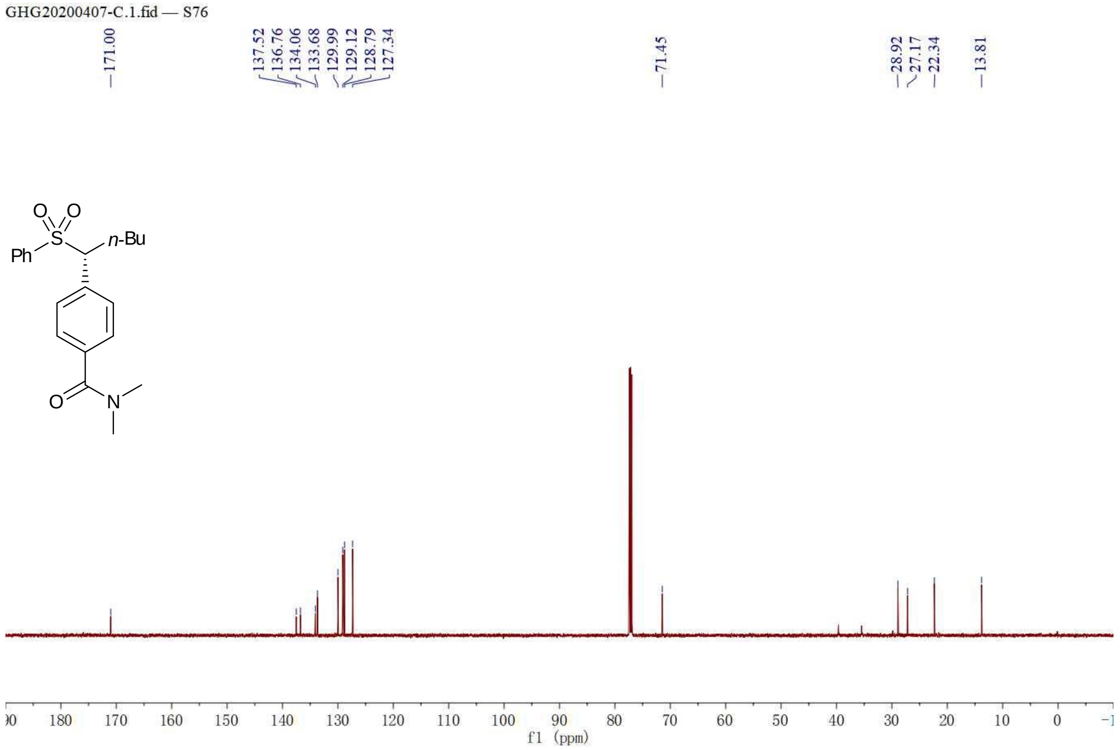

11, ^{1}H-NMR (600 MHZ, CDCl$_3$)

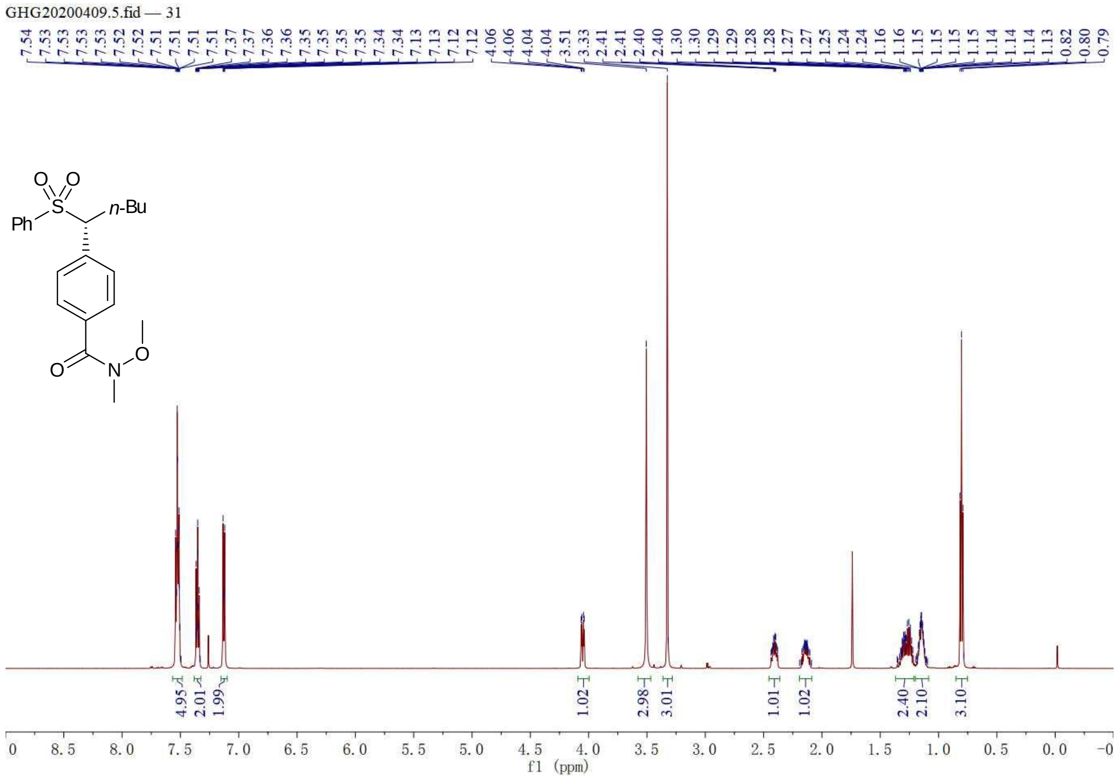

11, ^{13}C NMR (151 MHz, $CDCl_3$)

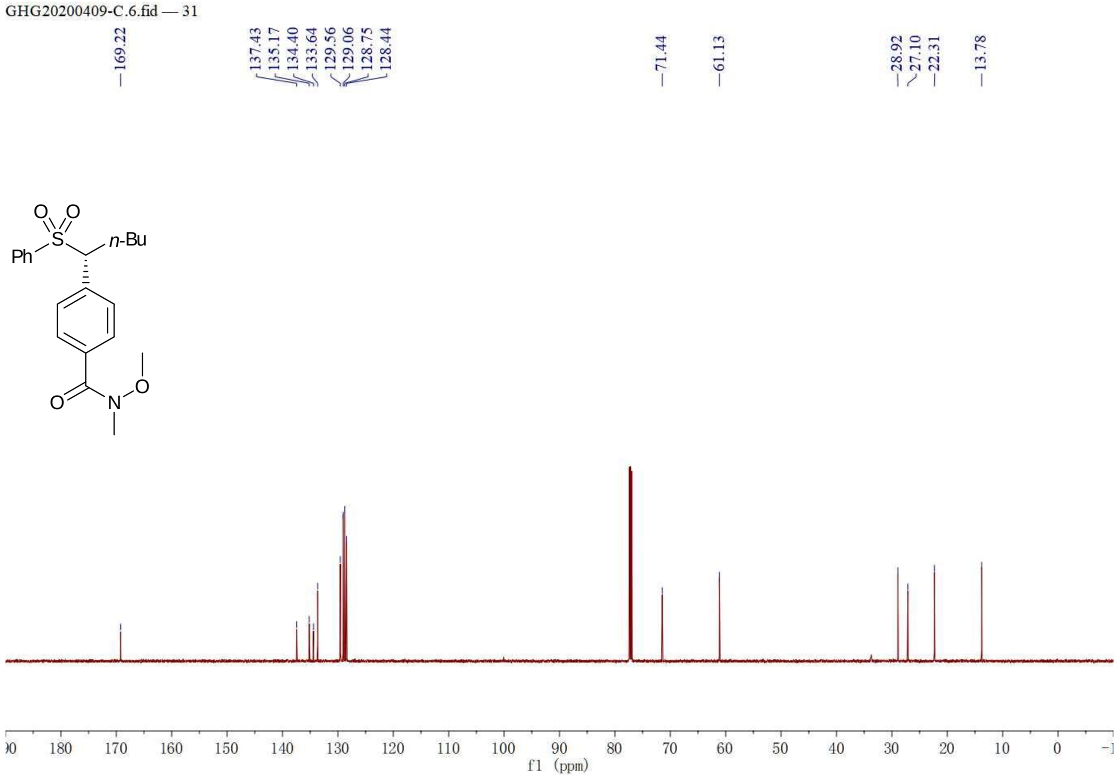

12, ^{1}H-NMR (600 MHZ, $CDCl_3$)

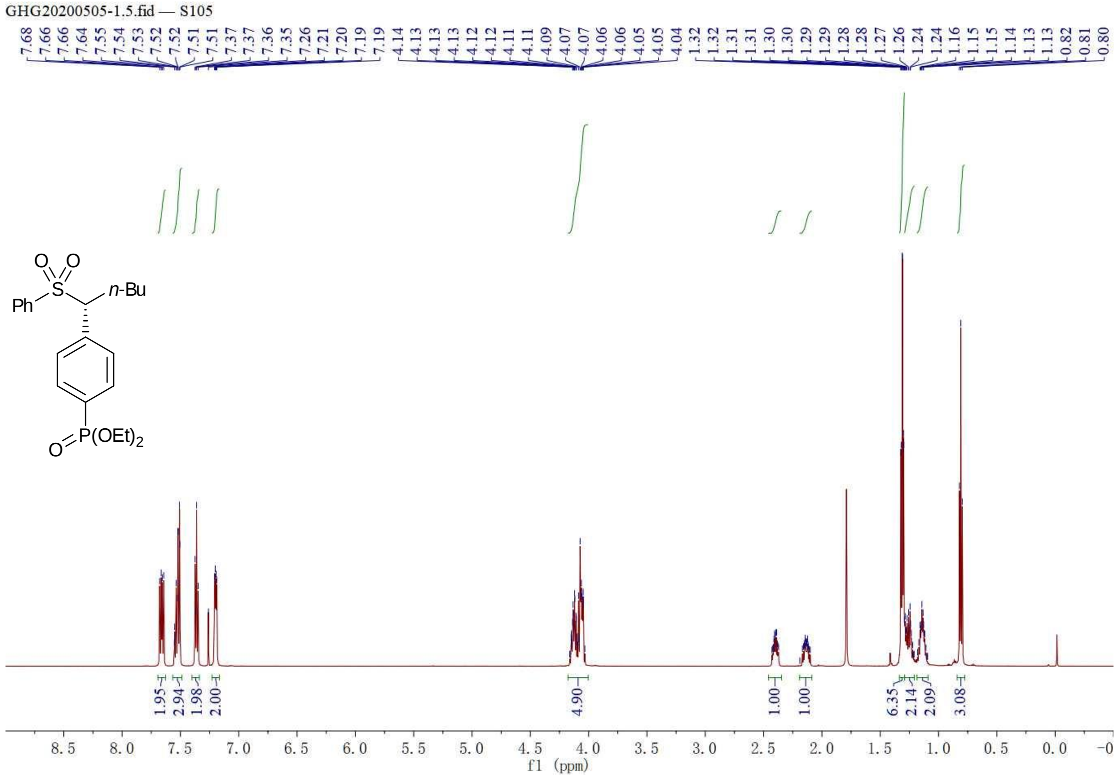

12, ^{13}C NMR (151 MHz, $CDCl_3$)

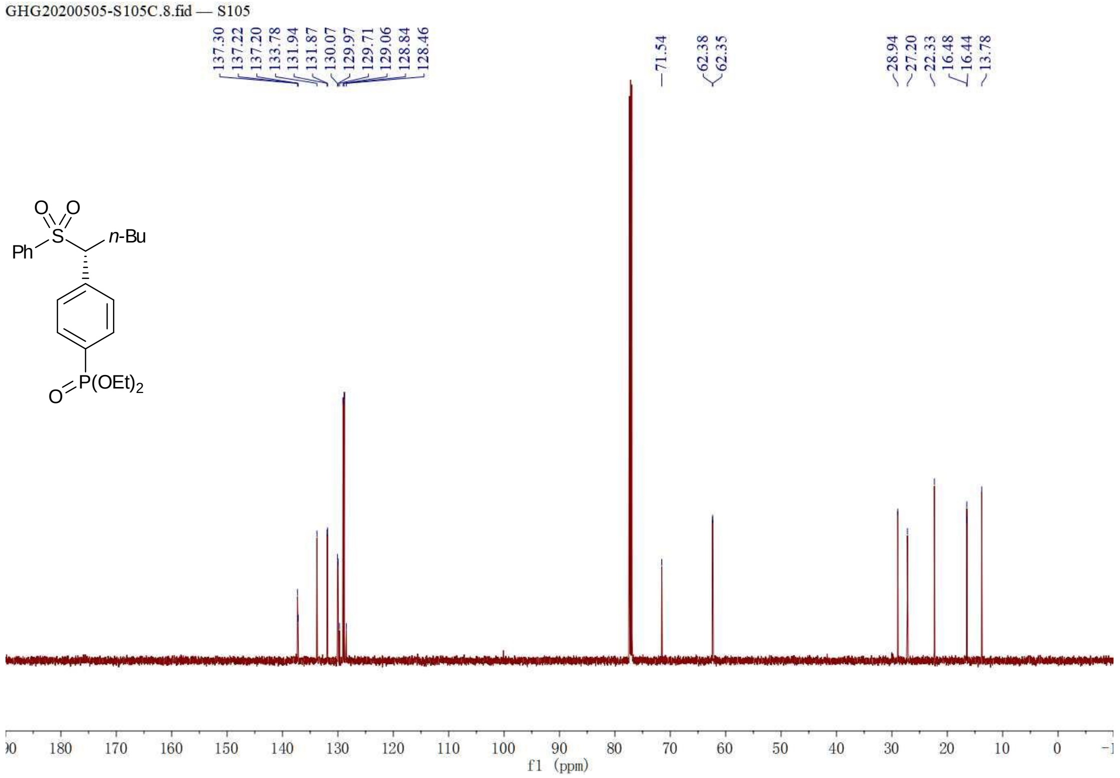

13, ^{1}H-NMR (600 MHZ, $CDCl_3$)

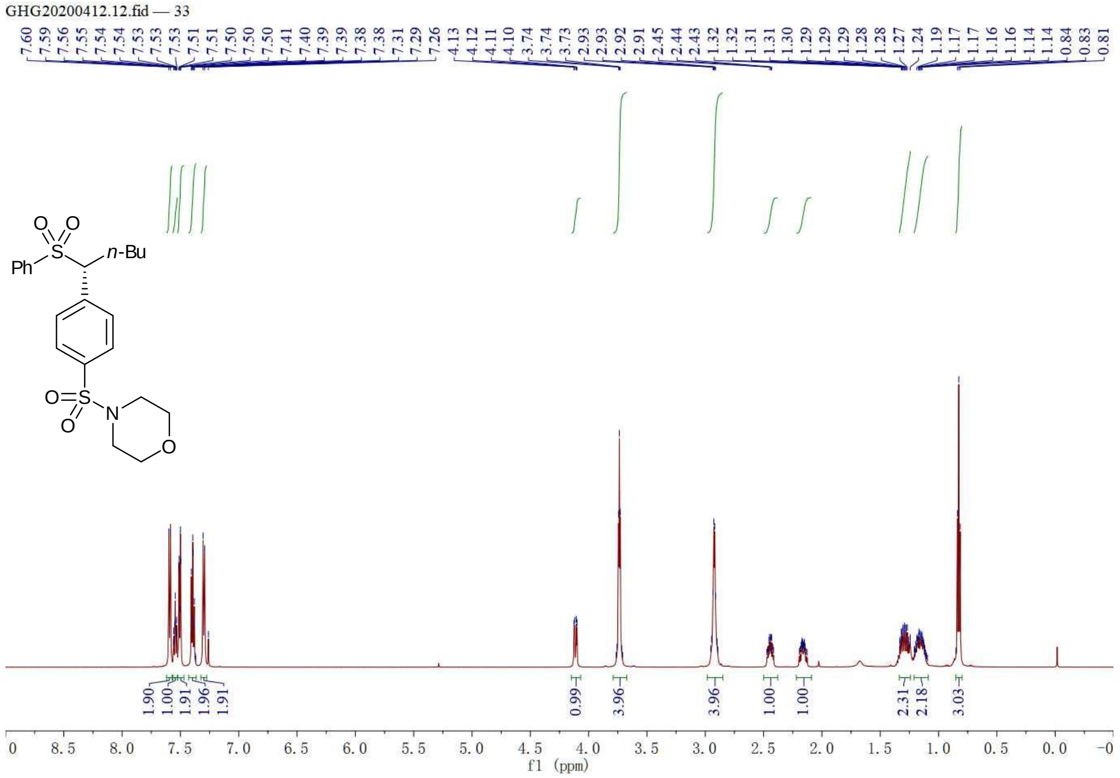

13, ^{13}C NMR (151 MHz, $CDCl_3$)

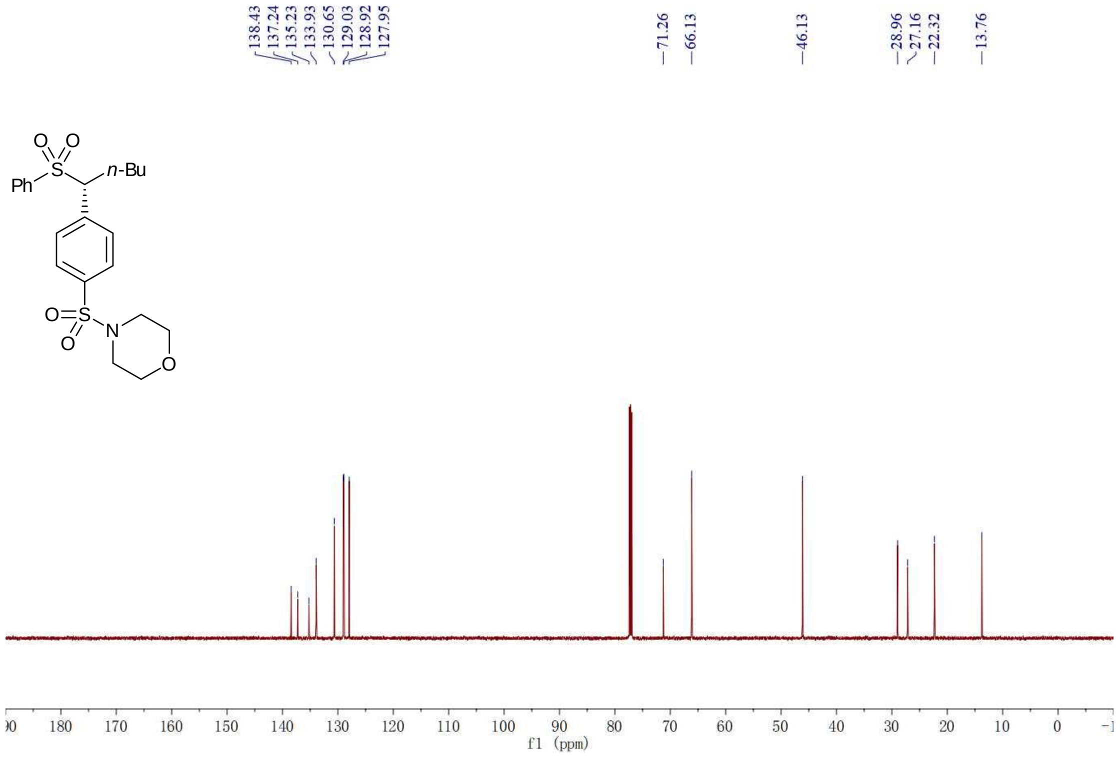

14, ^{1}H-NMR (600 MHZ, $CDCl_3$)

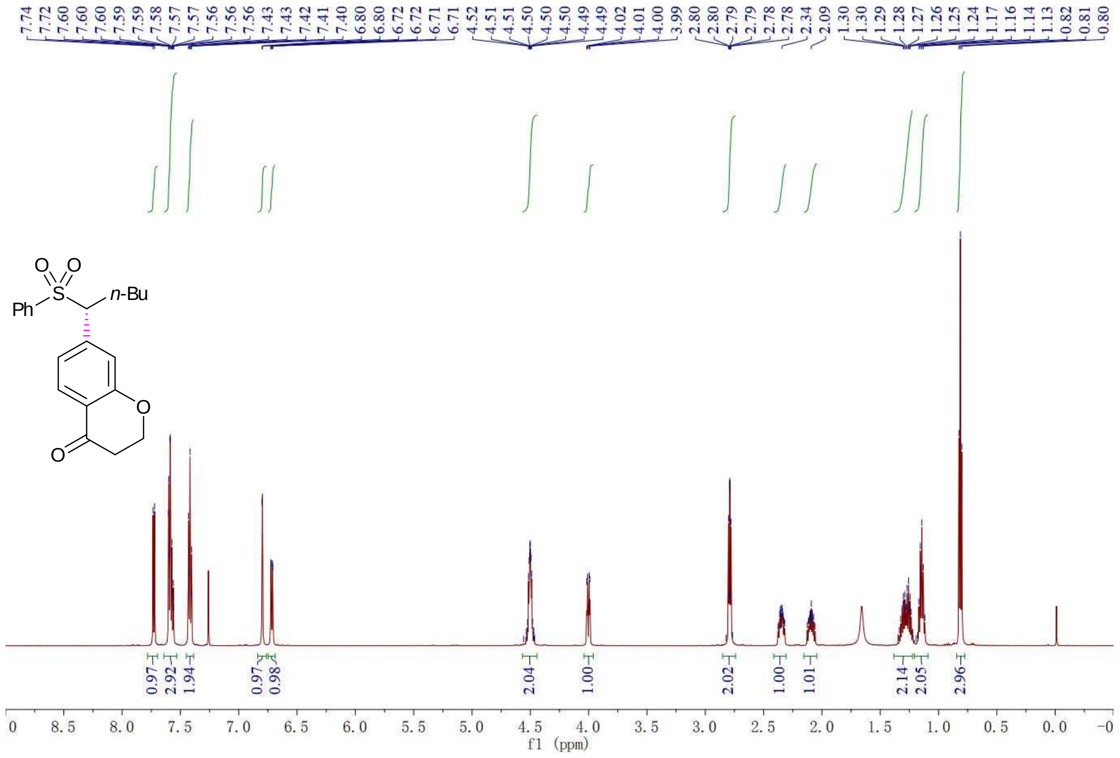

14, ^{13}C NMR (151 MHz, CDCl$_3$)

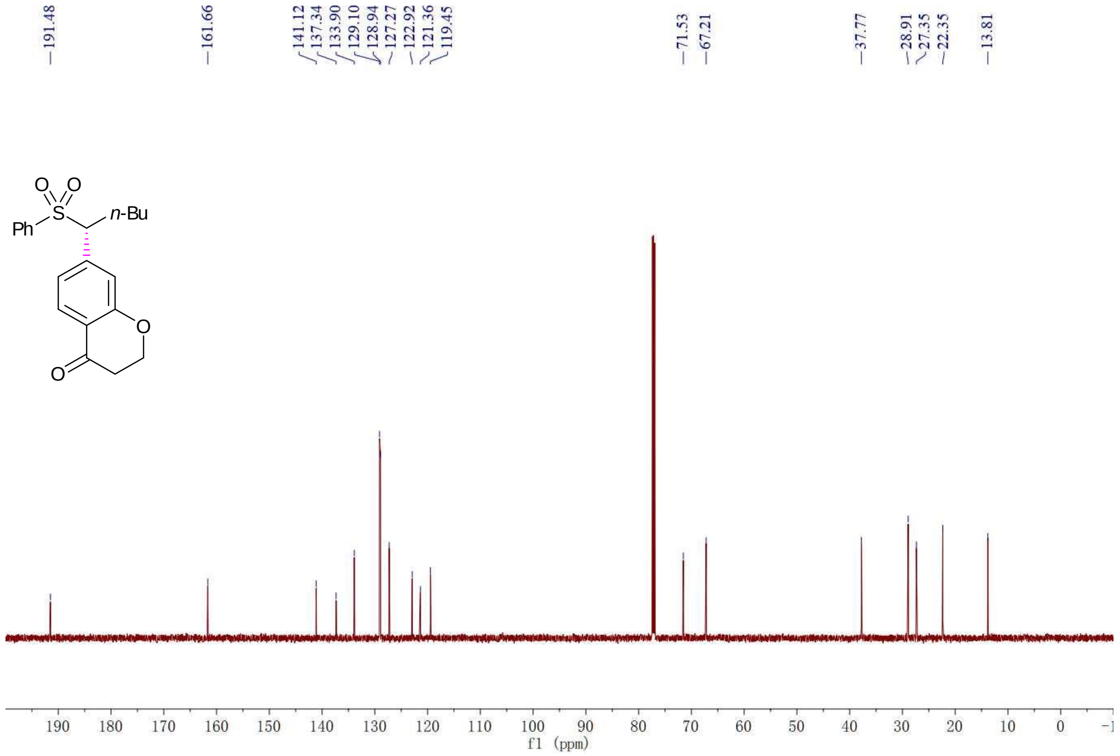

15, ^{1}H-NMR (600 MHZ, CDCl$_3$)

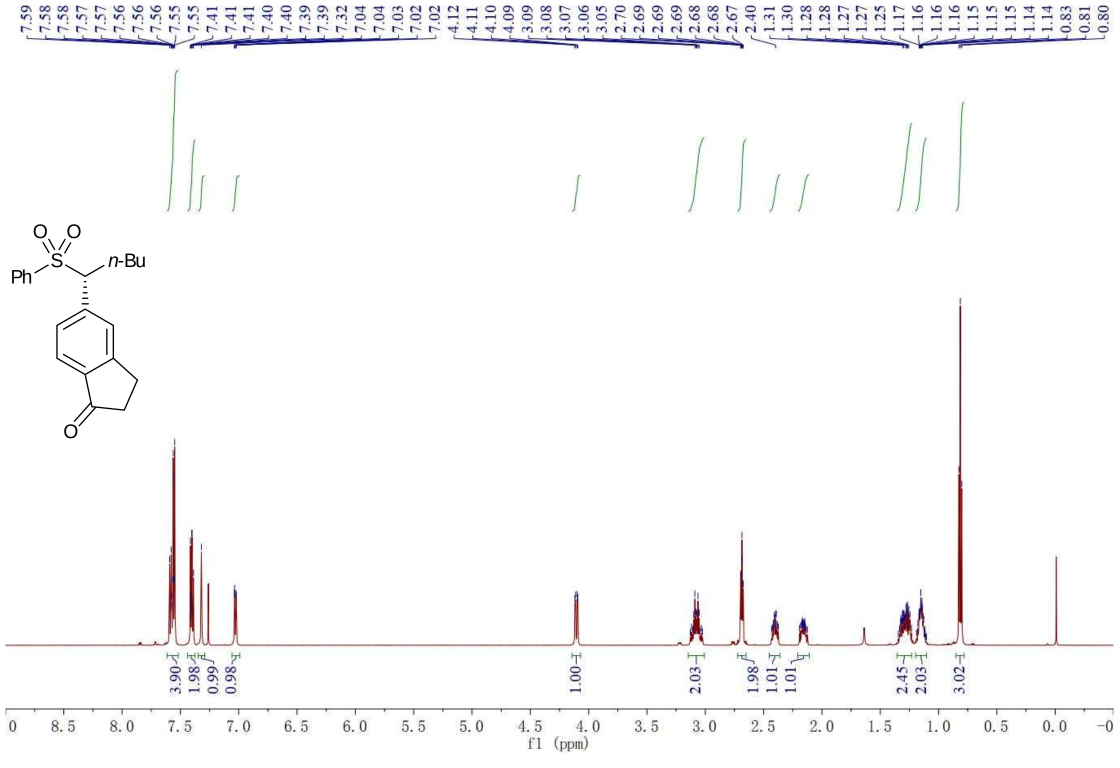

15, ^{13}C NMR (151 MHz, $CDCl_3$)

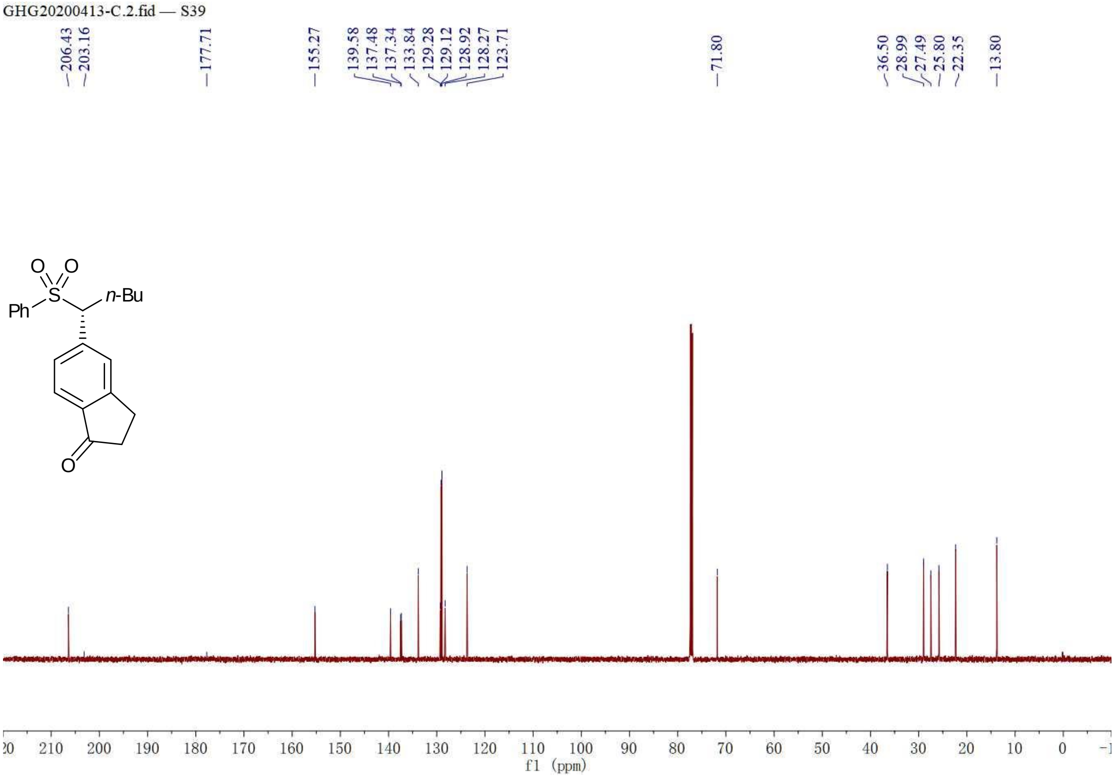

16, ^{1}H-NMR (600 MHZ, $CDCl_3$)

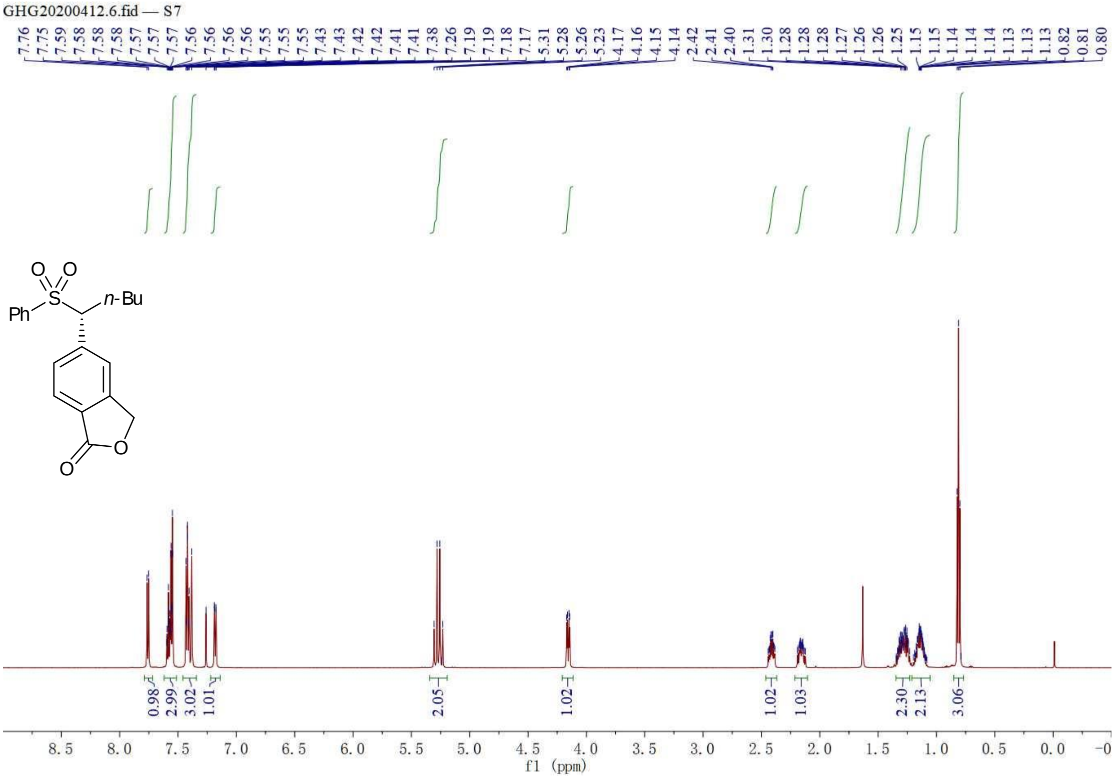

16, ^{13}C NMR (151 MHz, CDCl$_3$)

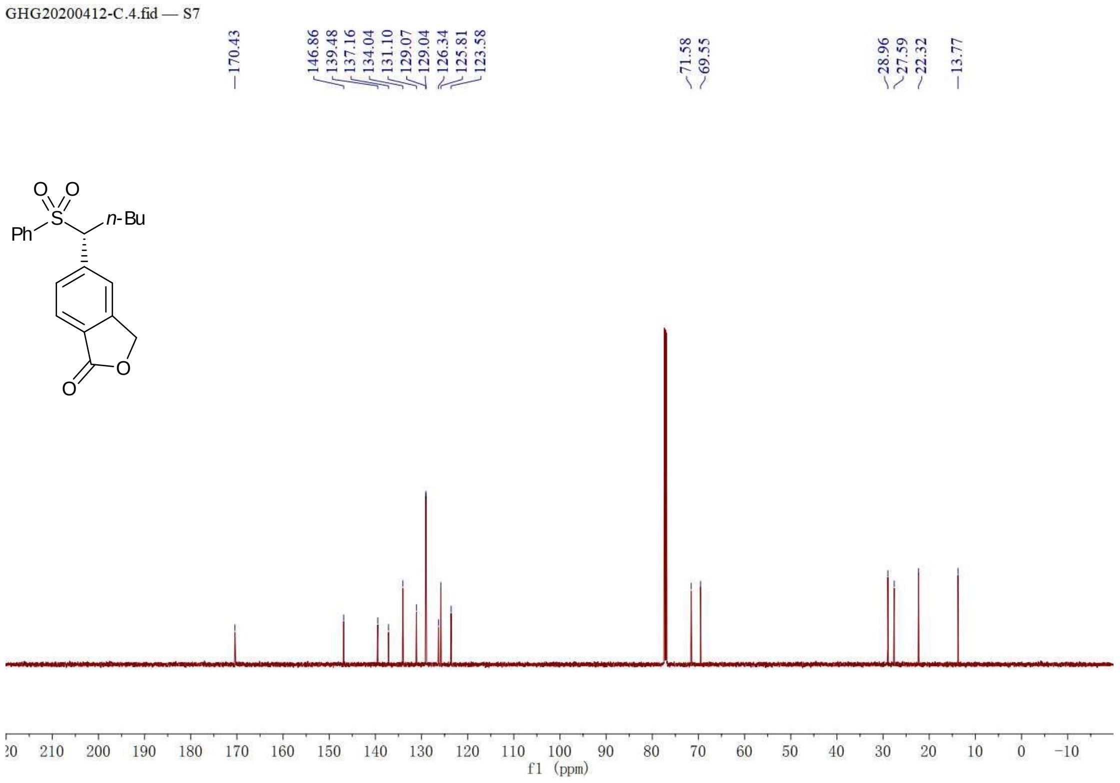

17, ^{1}H-NMR (600 MHZ, CDCl$_3$)

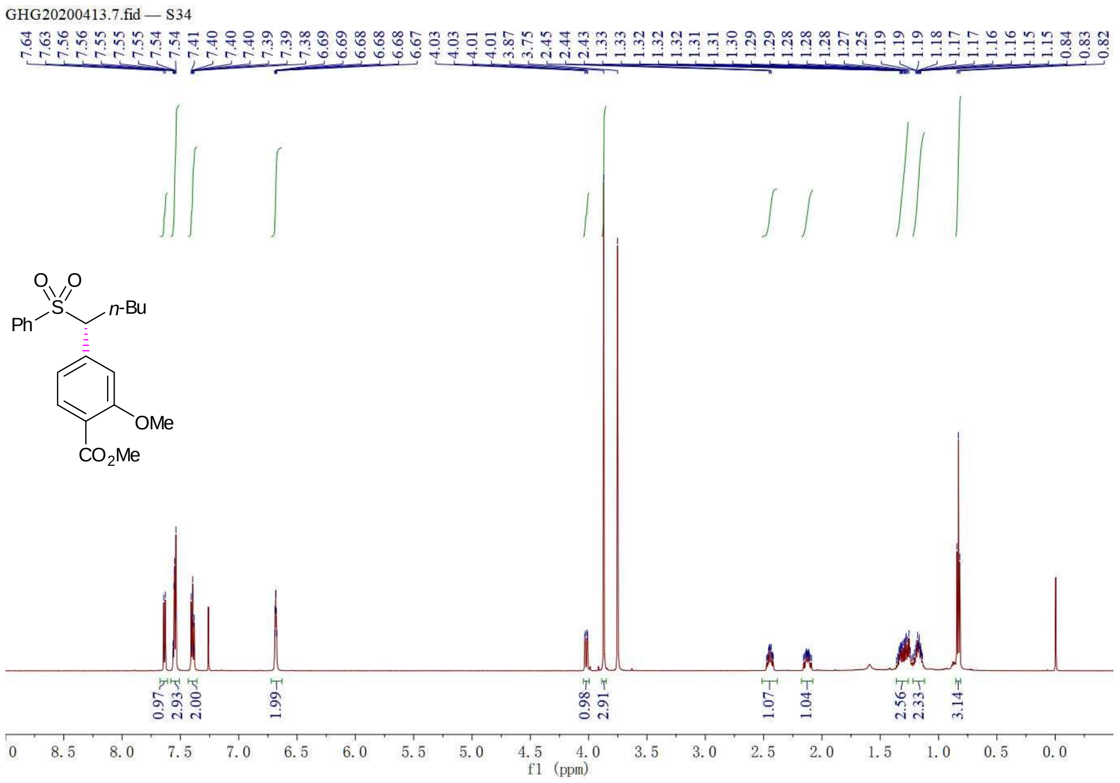

17, ^{13}C NMR (151 MHz, $CDCl_3$)

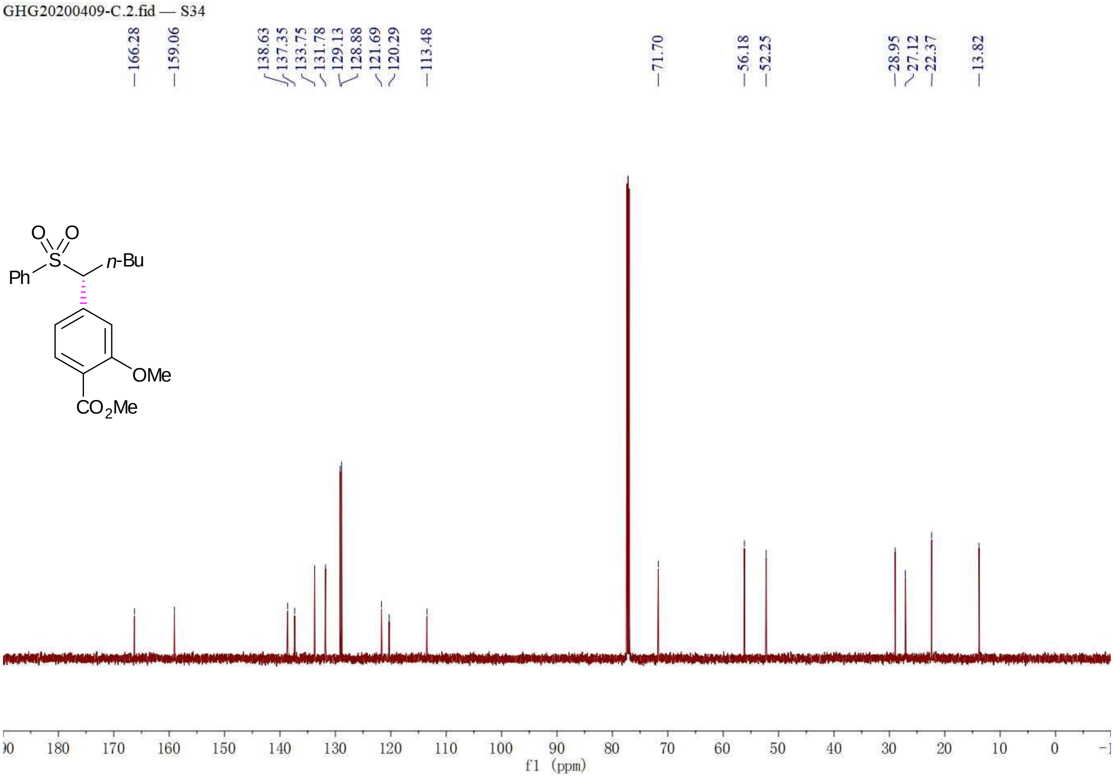

18, ^1H-NMR (600 MHZ, $CDCl_3$)

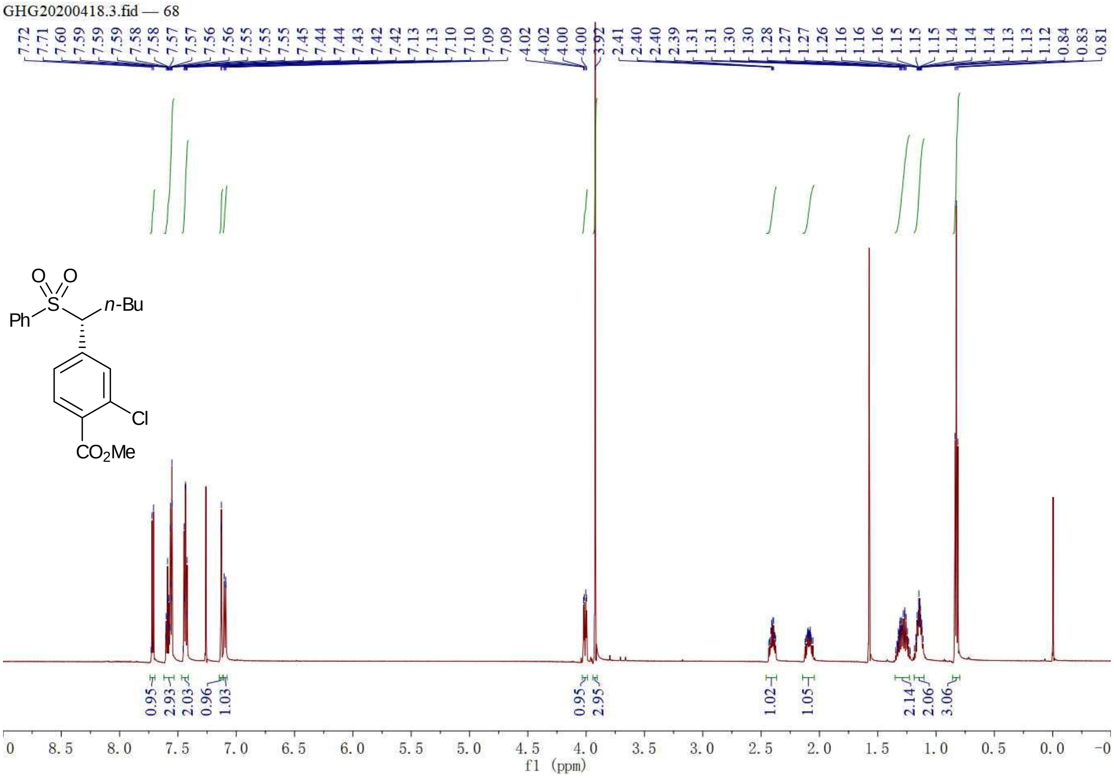

18, ^{13}C NMR (151 MHz, CDCl$_3$)

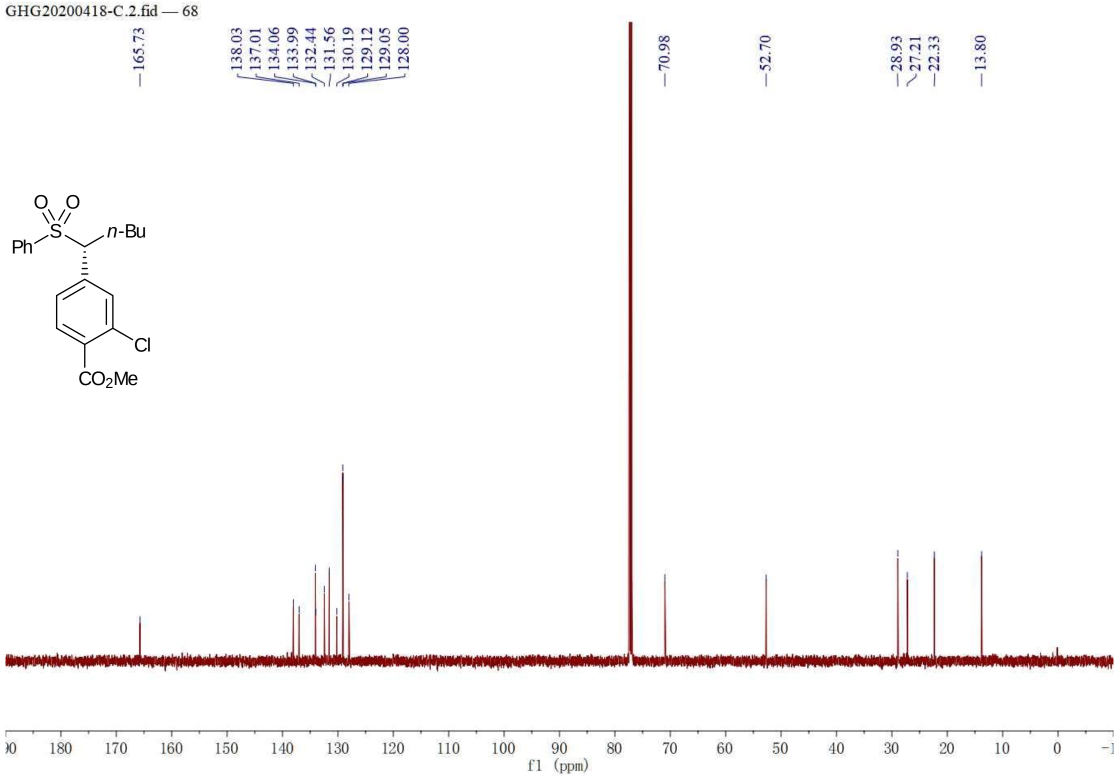

19, ^{1}H-NMR (600 MHZ, CDCl$_3$)

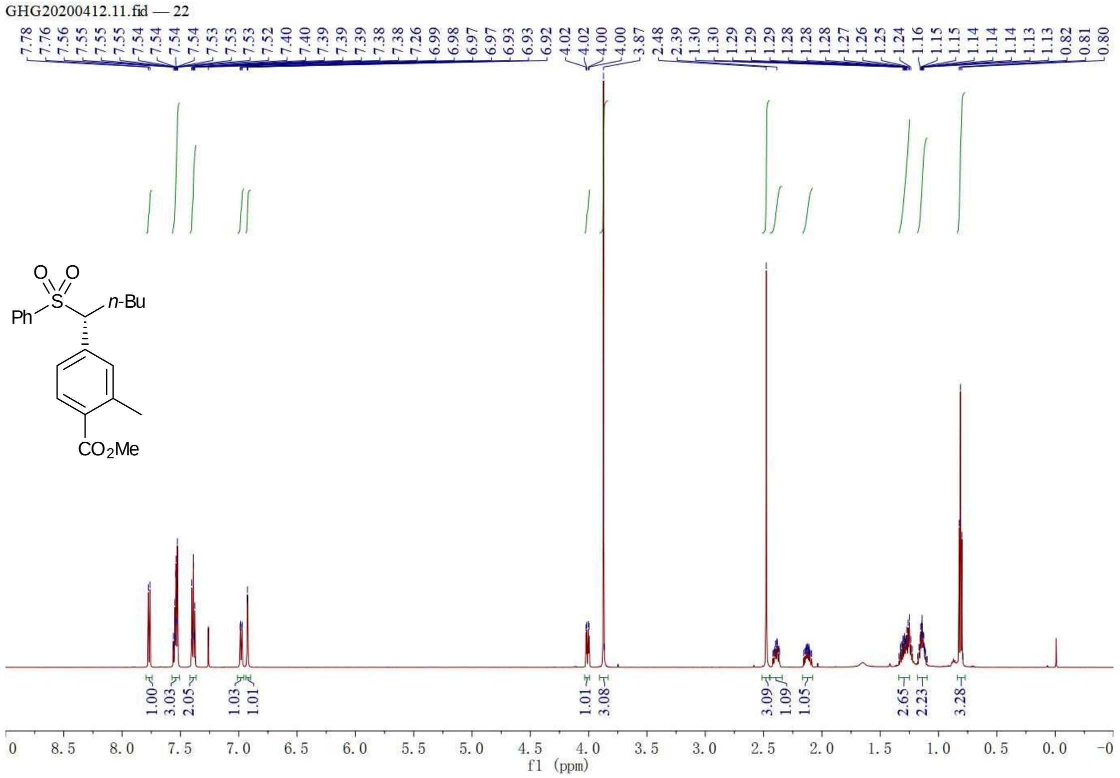

19, ^{13}C NMR (151 MHz, $CDCl_3$)

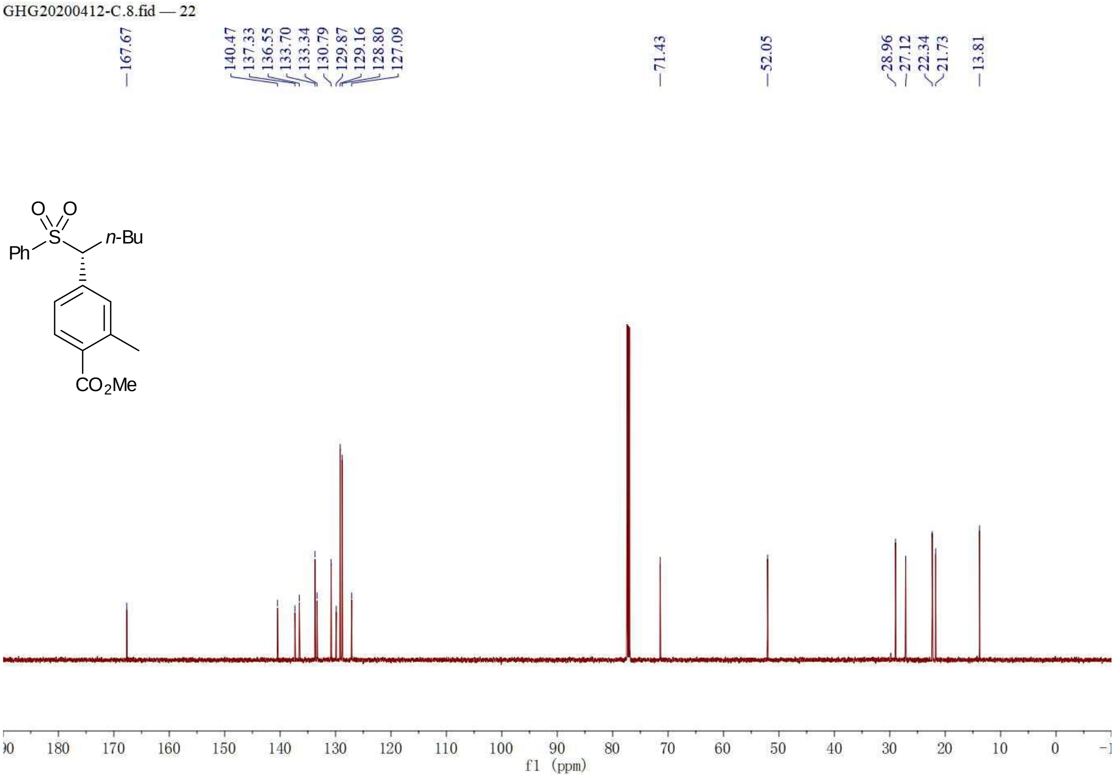

20, ^{1}H-NMR (600 MHZ, $CDCl_3$)

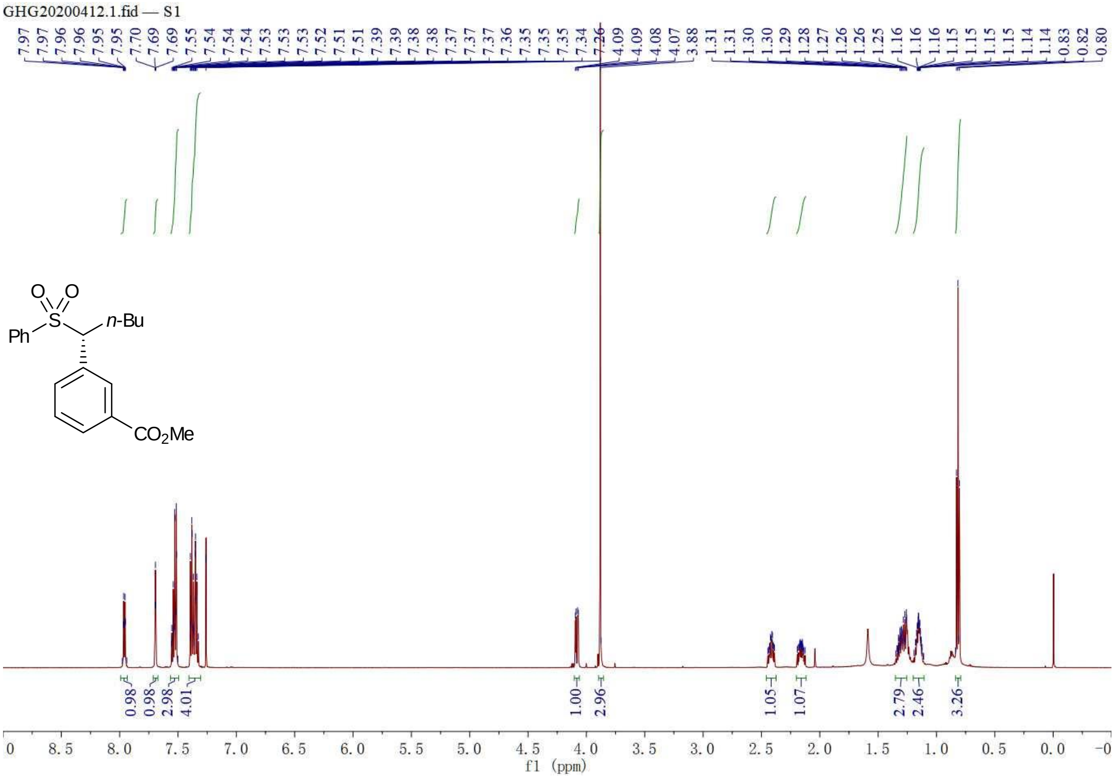

20, ^{13}C NMR (151 MHz, $CDCl_3$)

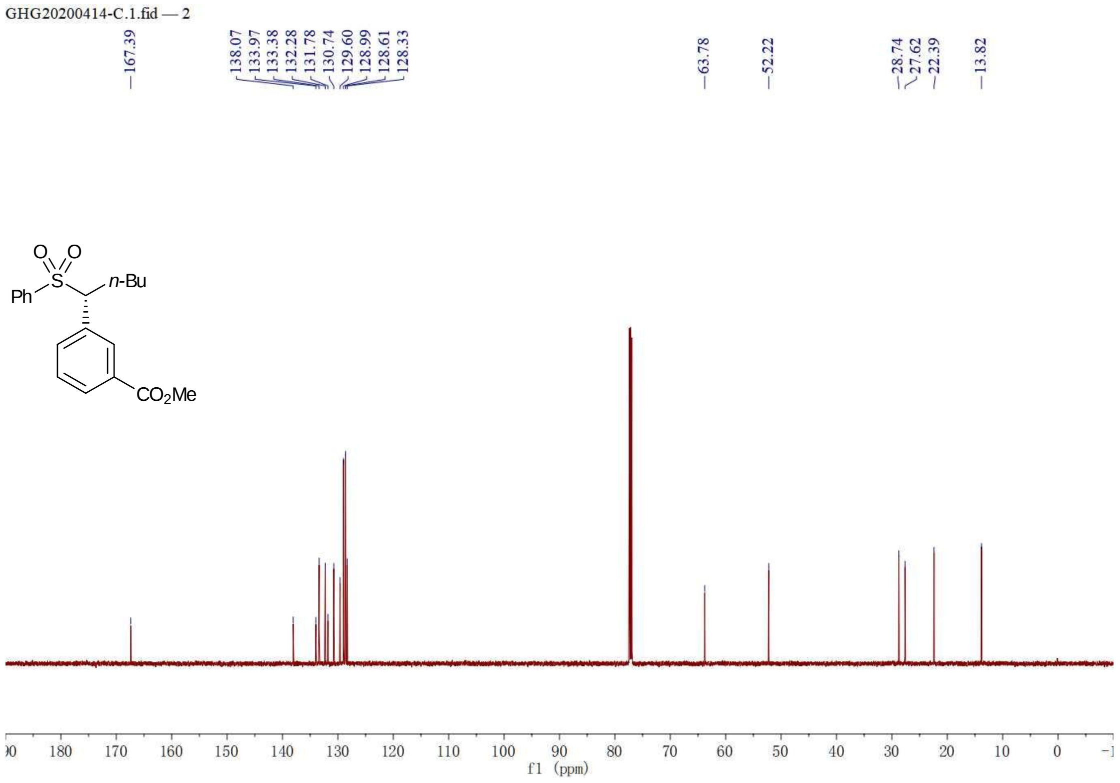

21, ^{1}H-NMR (600 MHZ, $CDCl_3$)

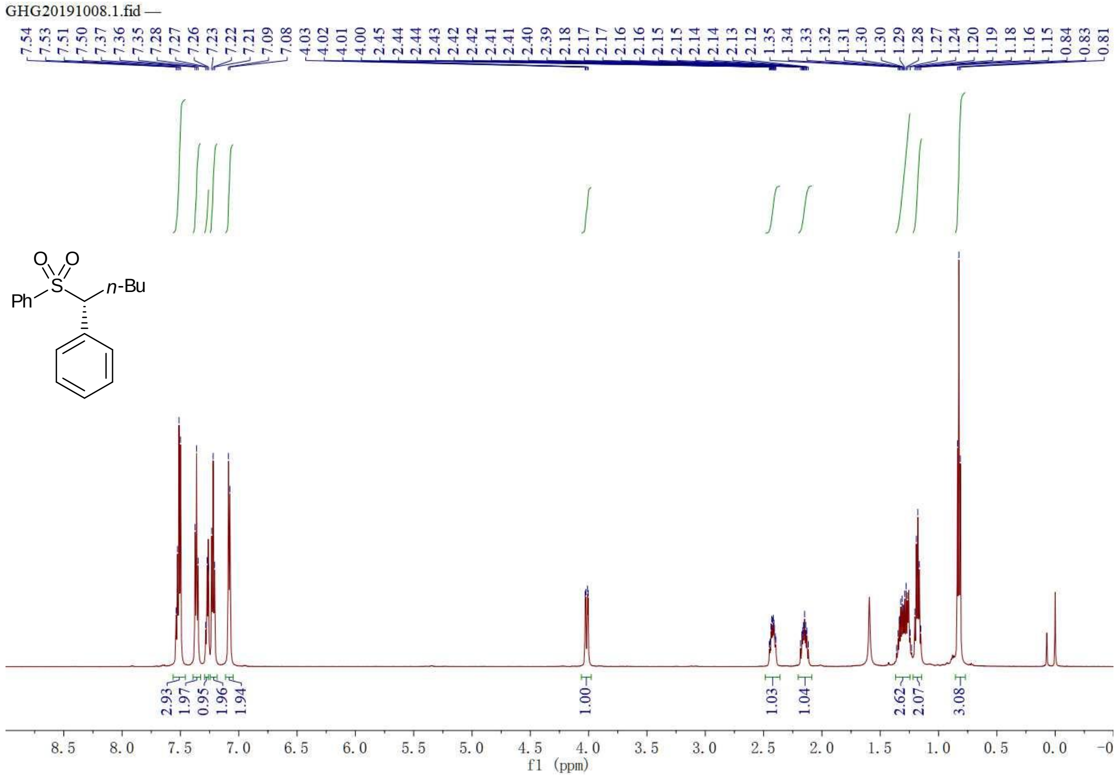

21, ^{13}C NMR (151 MHz, CDCl$_3$)

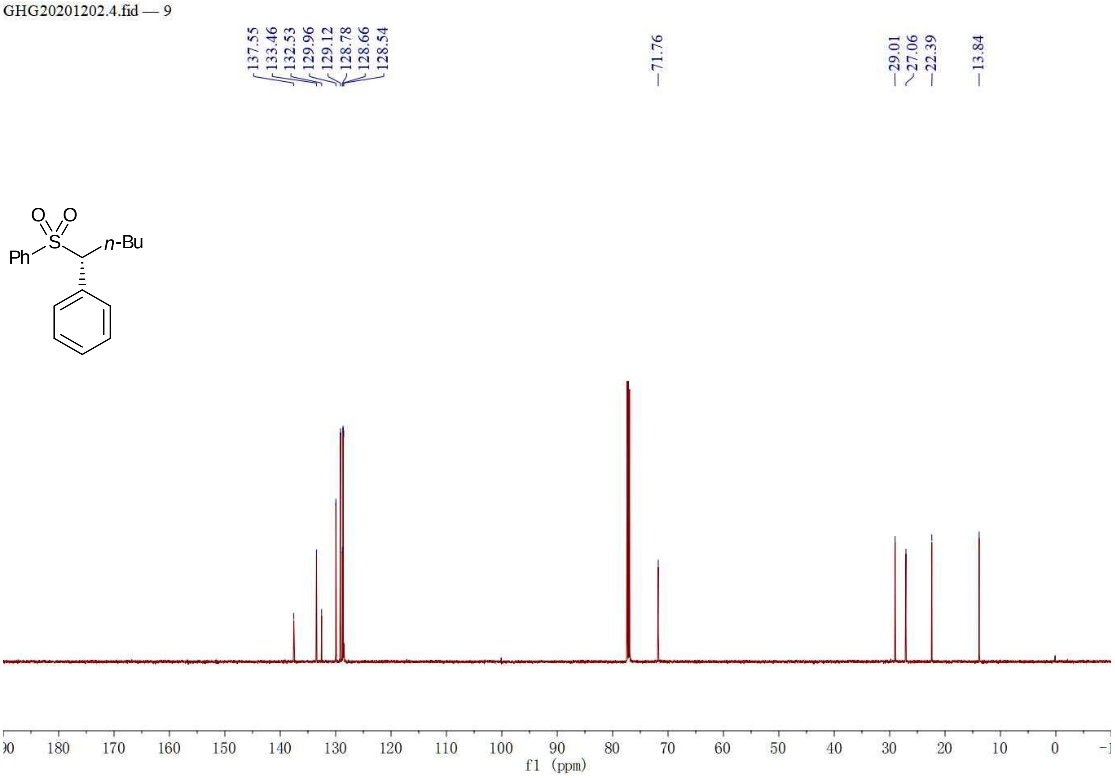

22, ^{1}H-NMR (600 MHZ, CDCl$_3$)

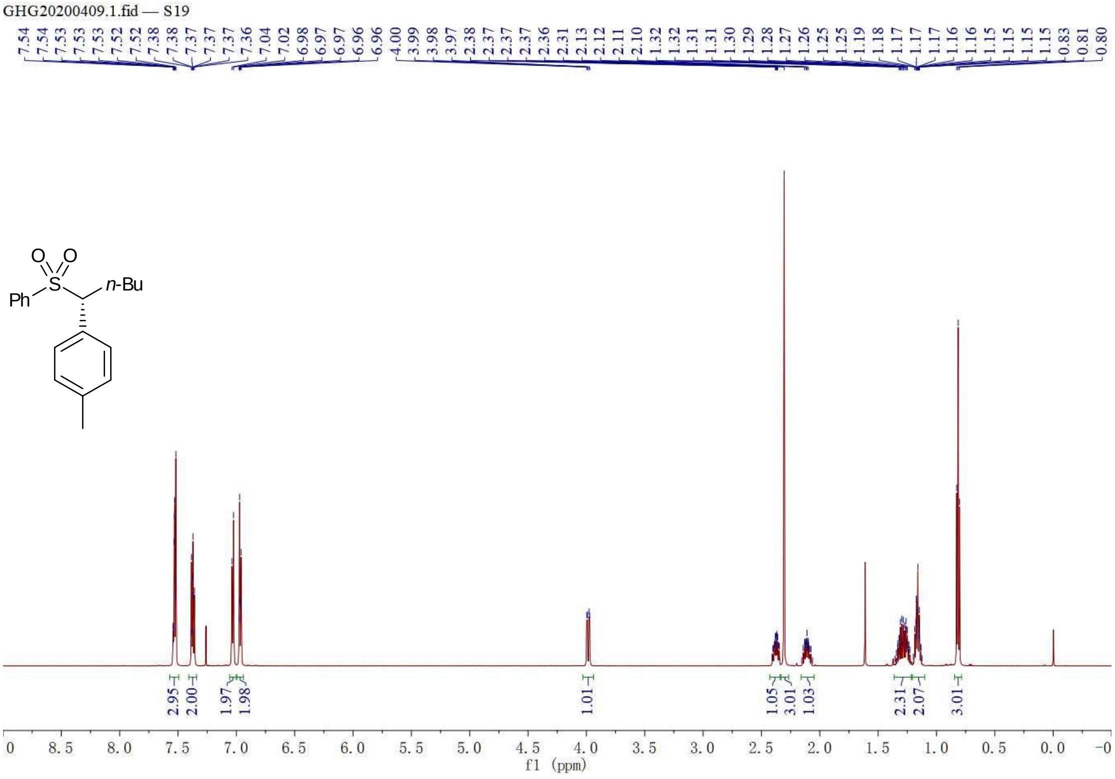

22, ^{13}C NMR (151 MHz, CDCl$_3$)

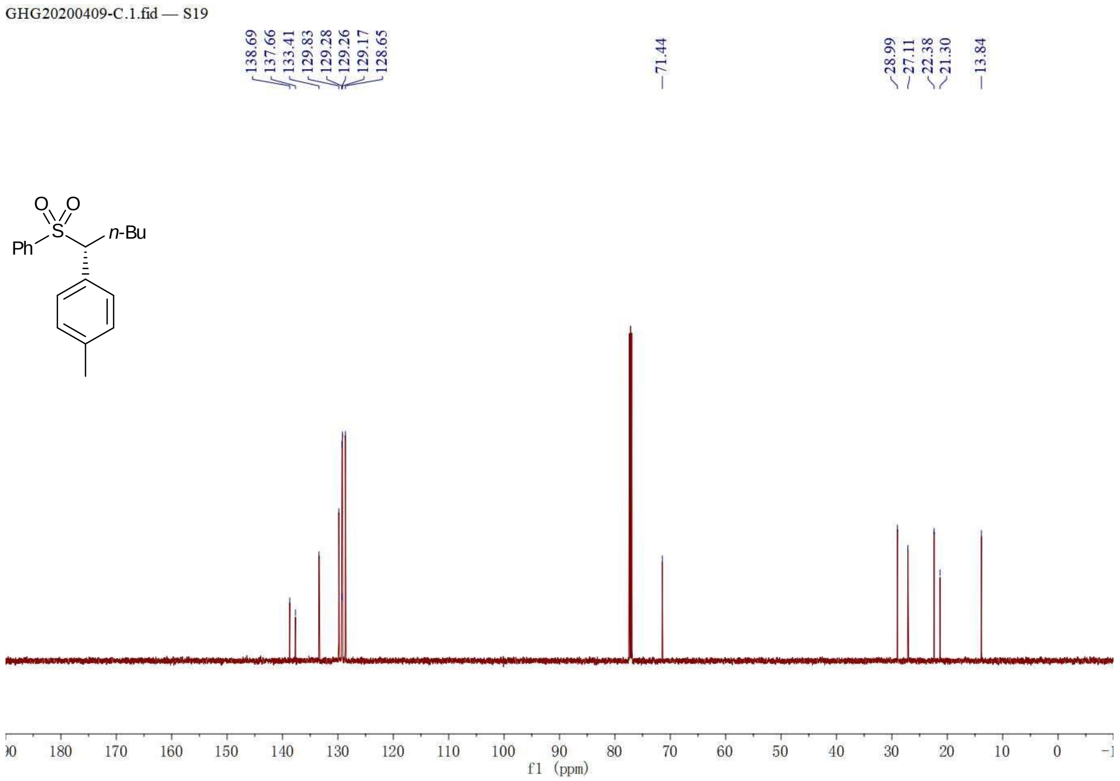

23, ^{1}H-NMR (600 MHZ, CDCl$_3$)

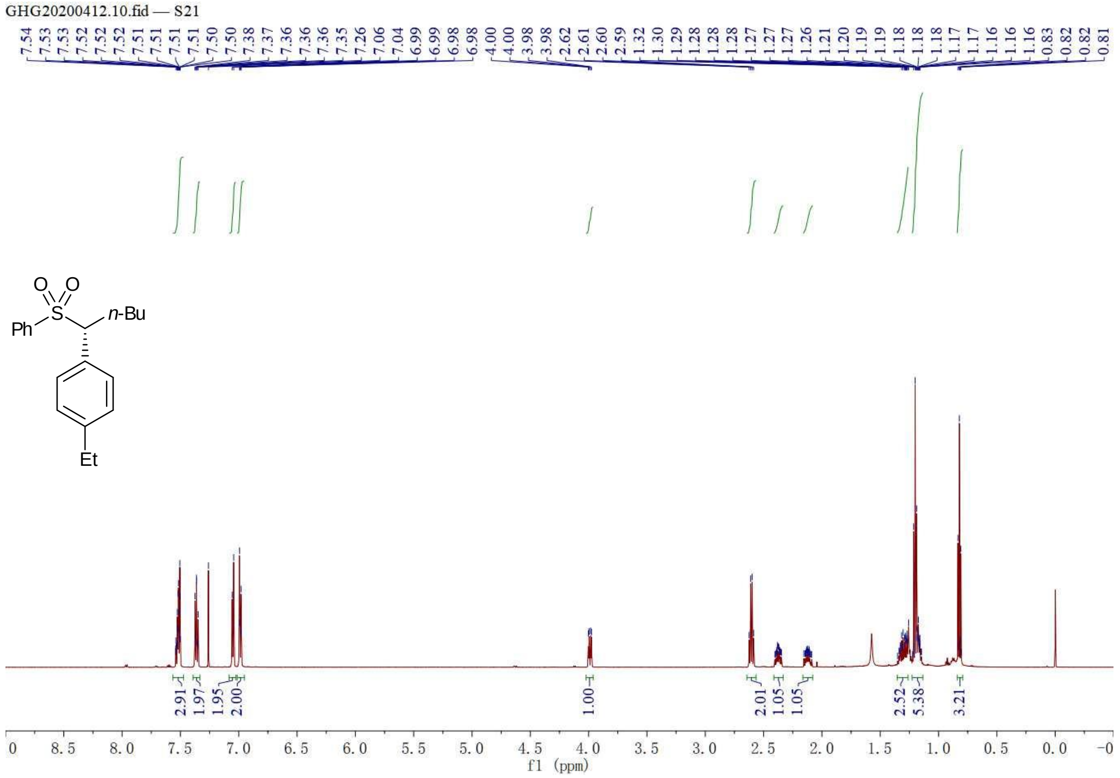

23, ^{13}C NMR (151 MHz, $CDCl_3$)

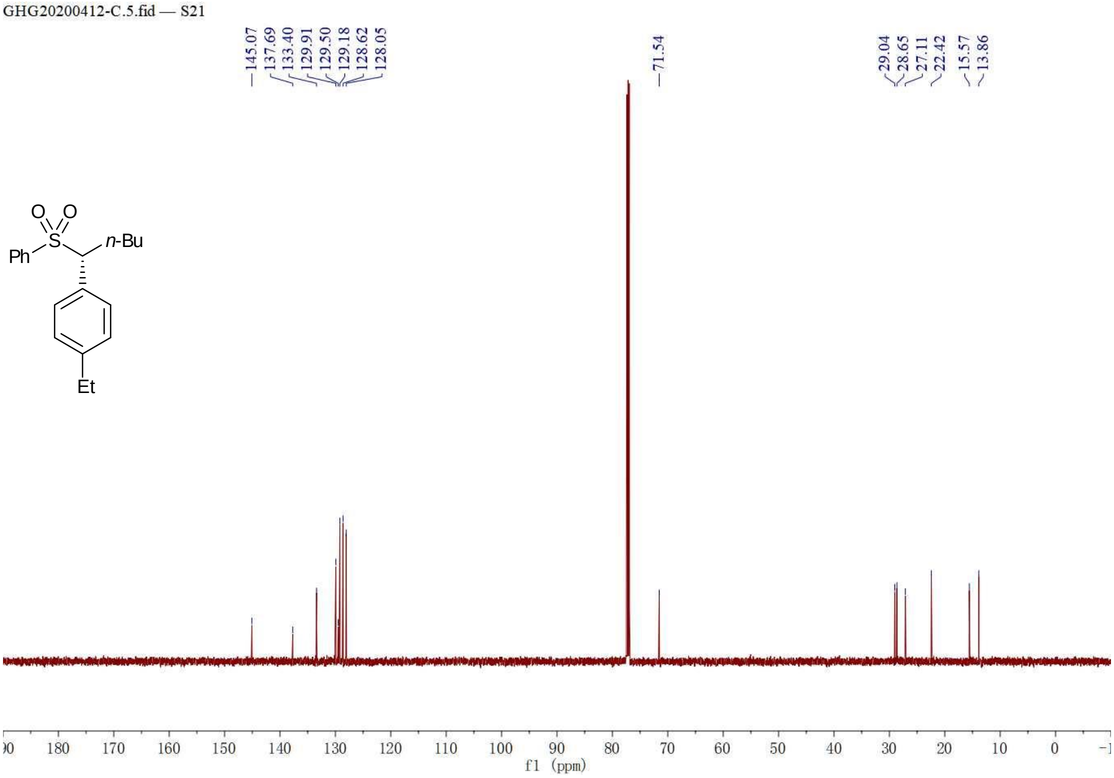

24, ^{1}H-NMR (600 MHZ, $CDCl_3$)

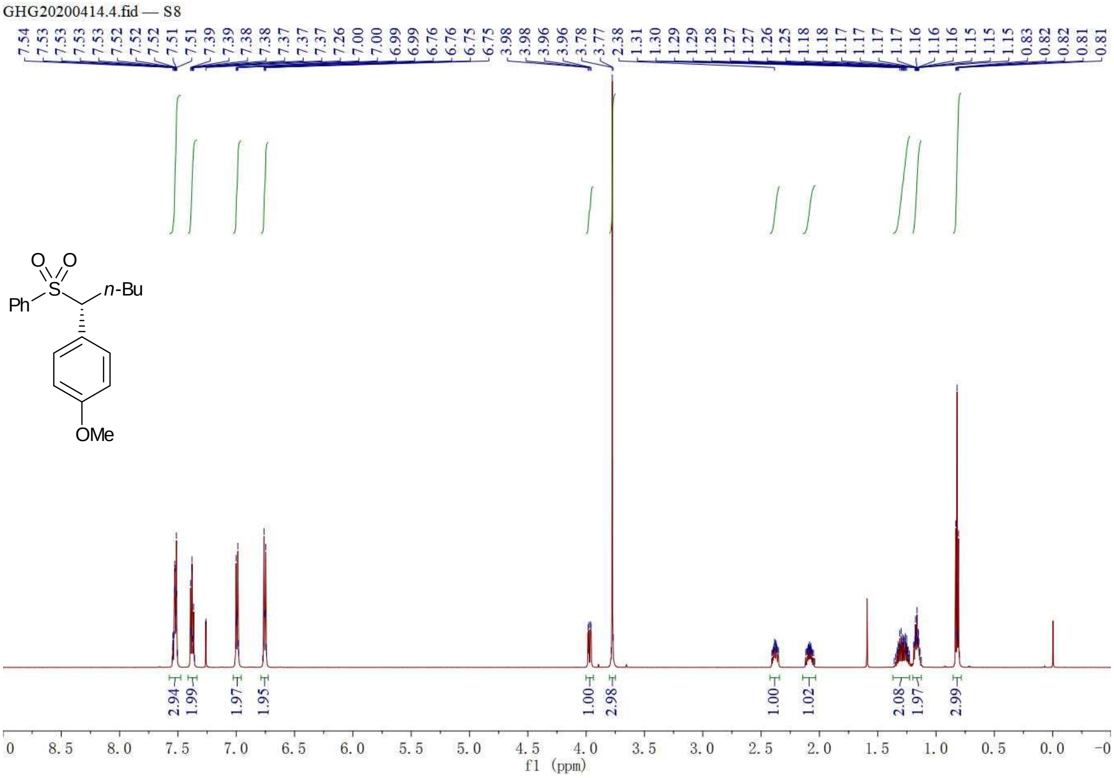

24, ^{13}C NMR (151 MHz, CDCl$_3$)

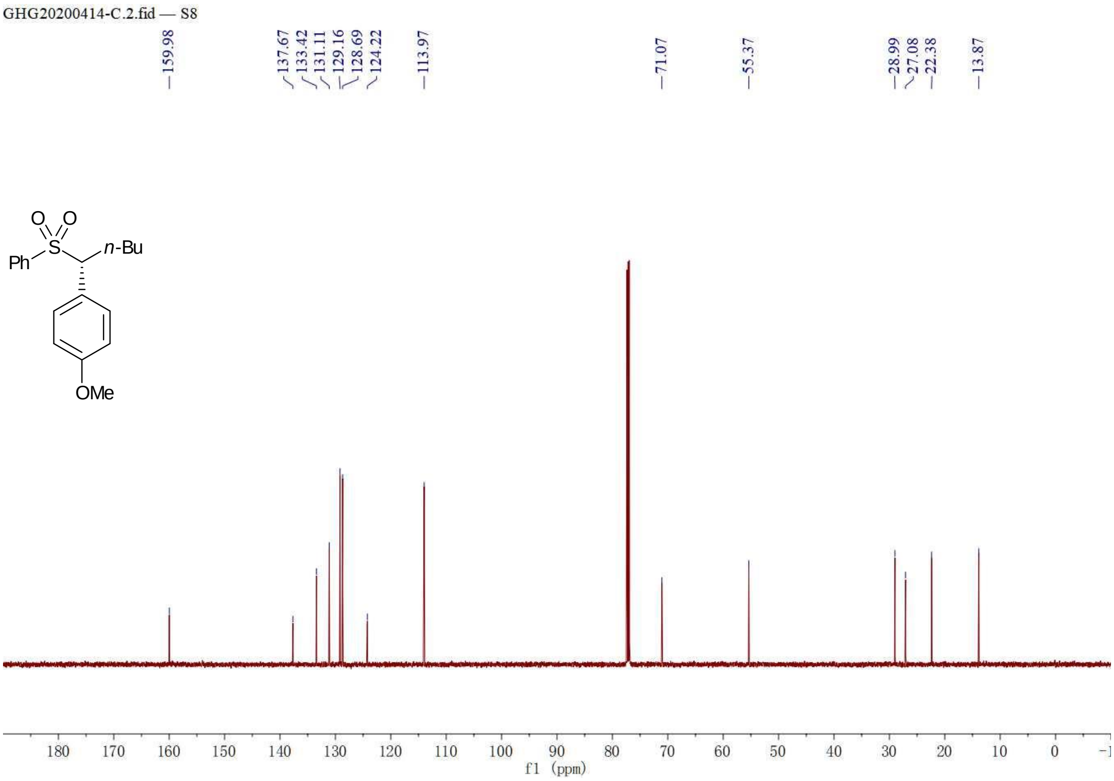

25, ^{1}H-NMR (600 MHZ, CDCl$_3$)

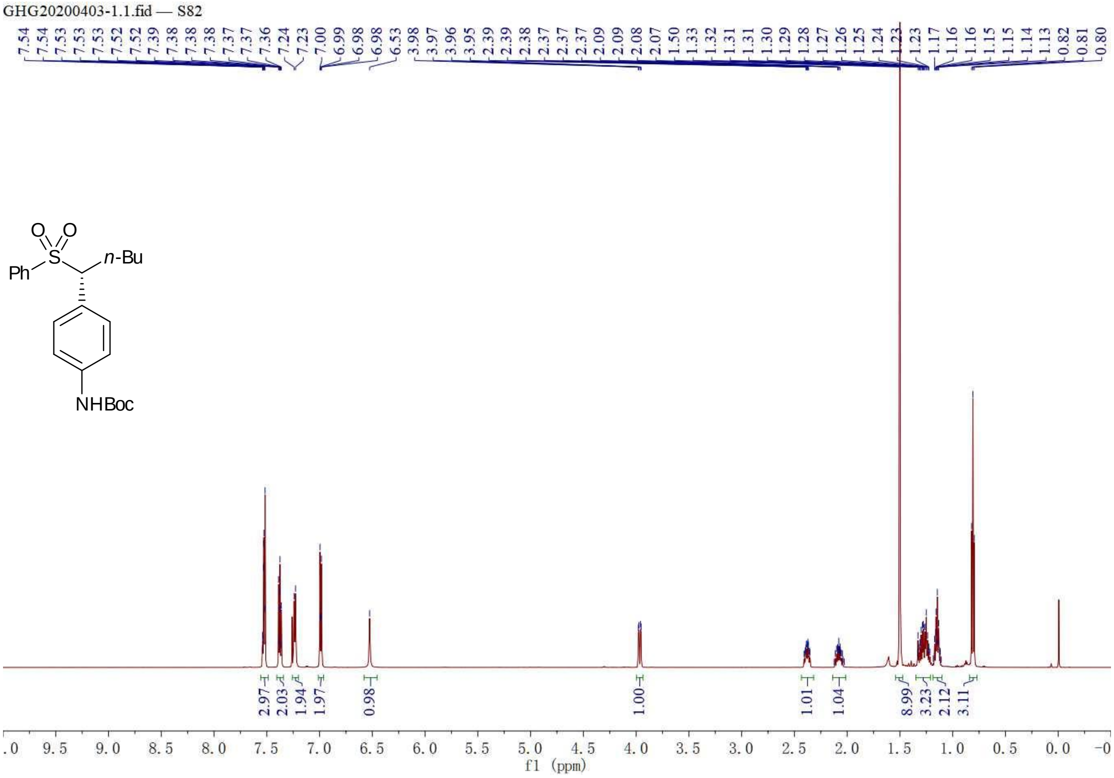

25, ^{13}C NMR (151 MHz, $CDCl_3$)

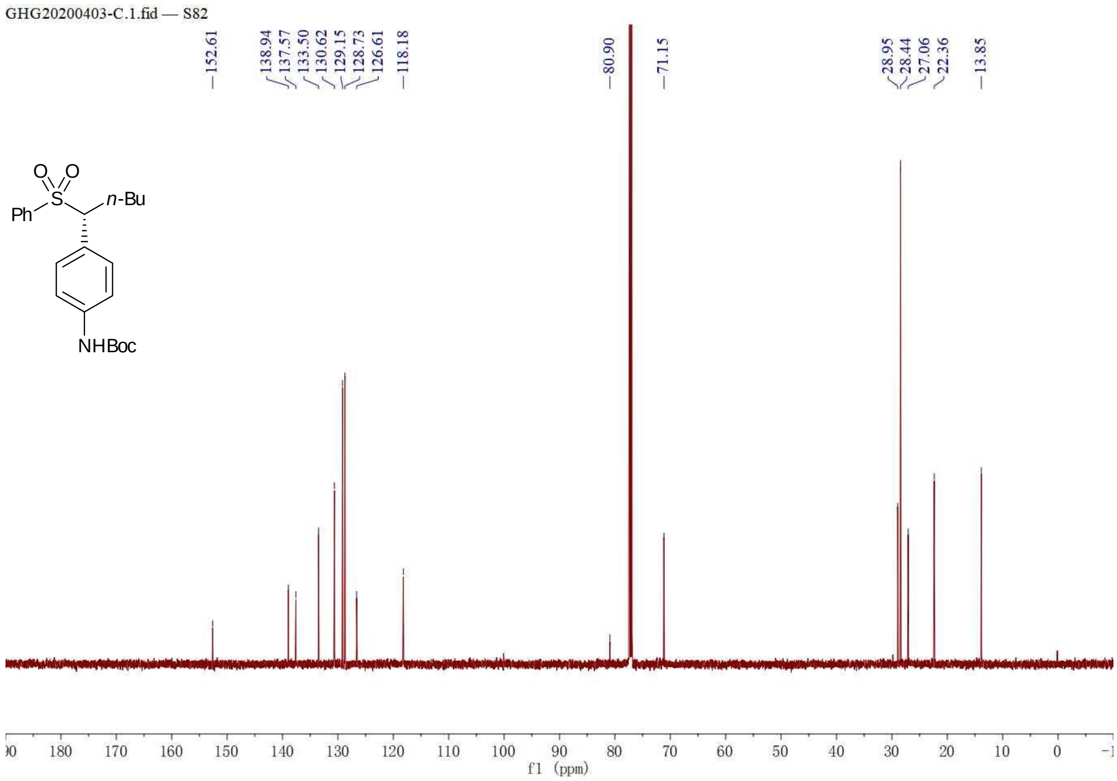

26, ^{1}H-NMR (600 MHZ, $CDCl_3$)

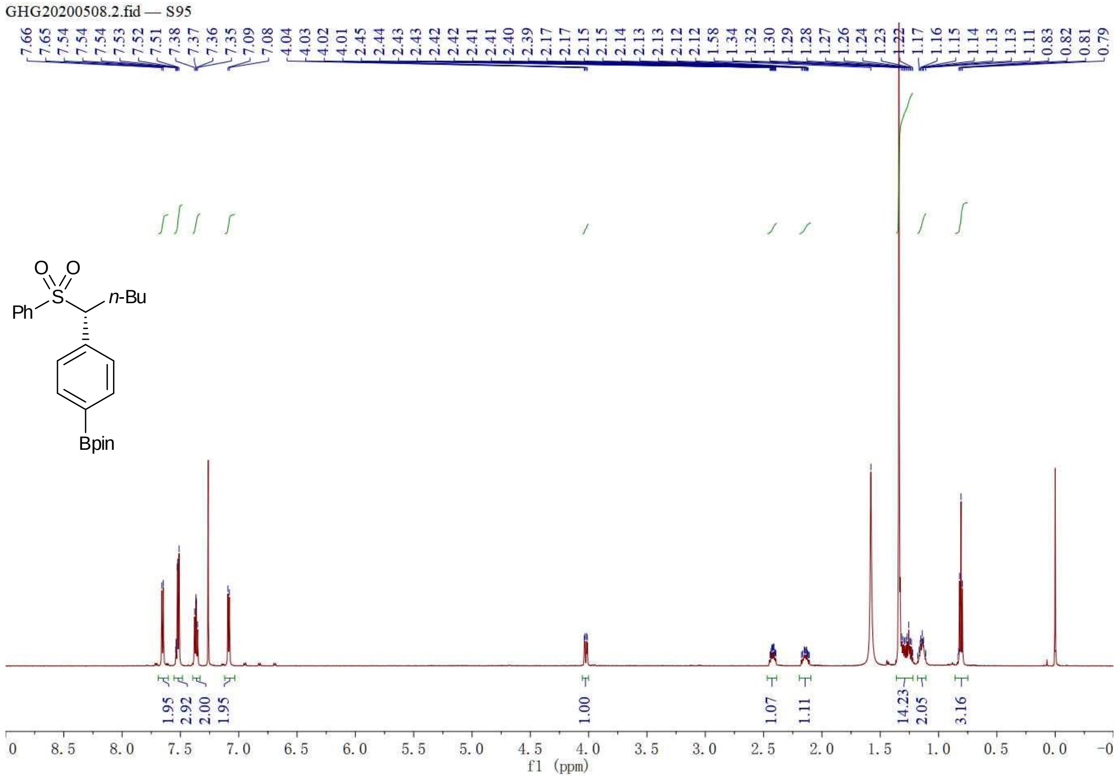

26, ^{13}C NMR (151 MHz, $CDCl_3$)

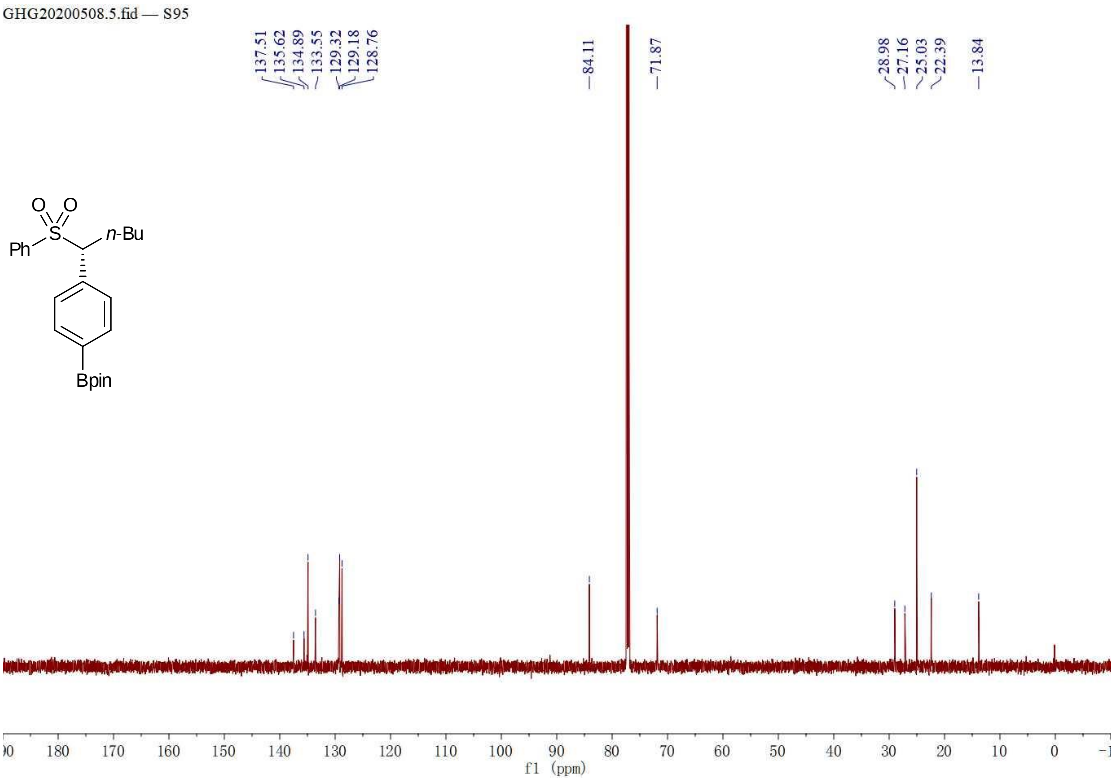

27, ^{1}H-NMR (600 MHZ, $CDCl_3$)

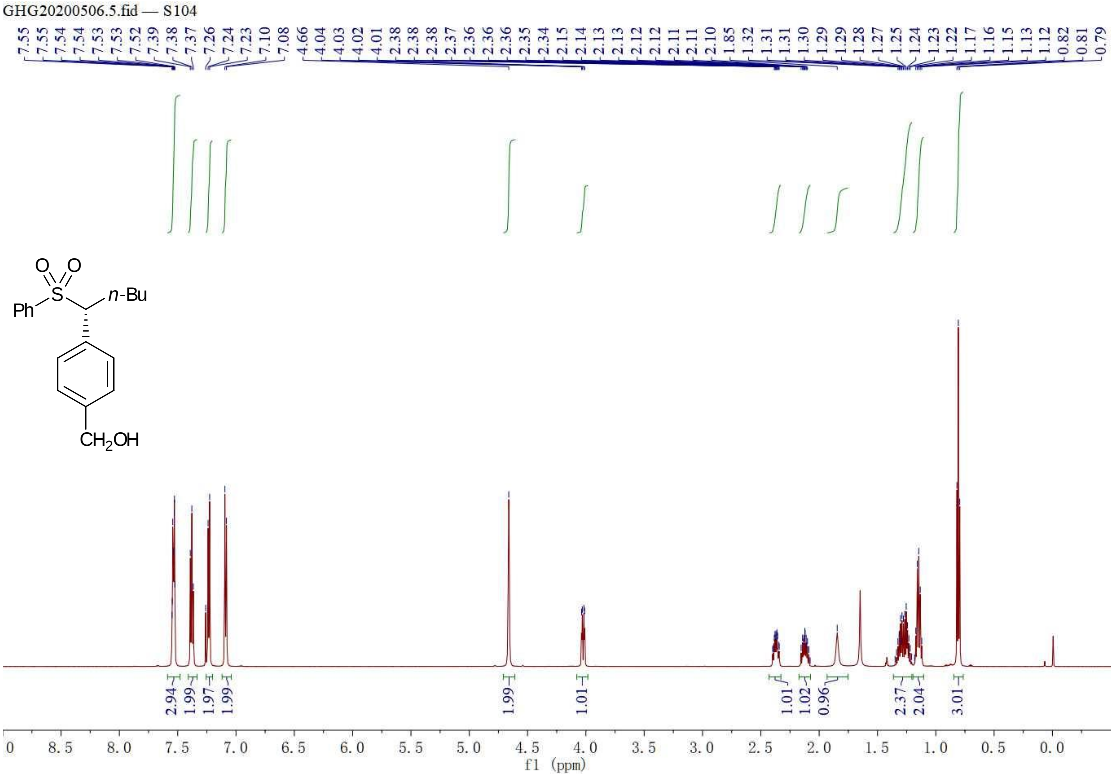

27, ^{13}C NMR (151 MHz, CDCl$_3$)

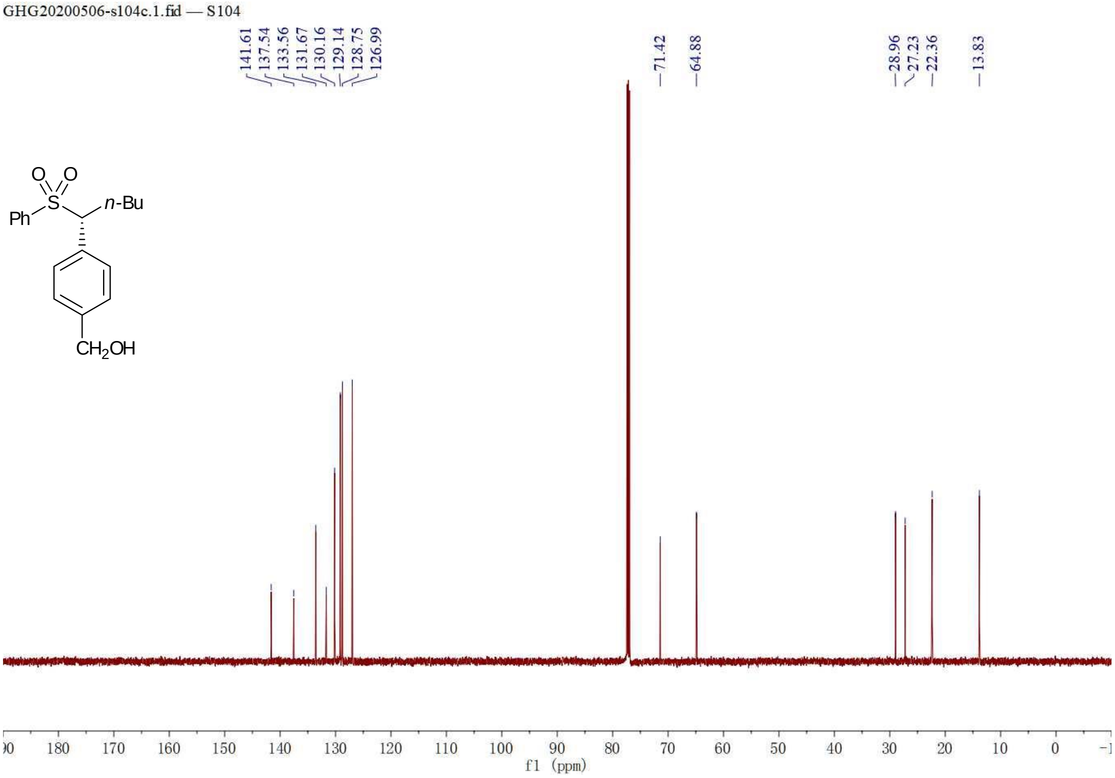

28, ^{1}H-NMR (600 MHZ, CDCl$_3$)

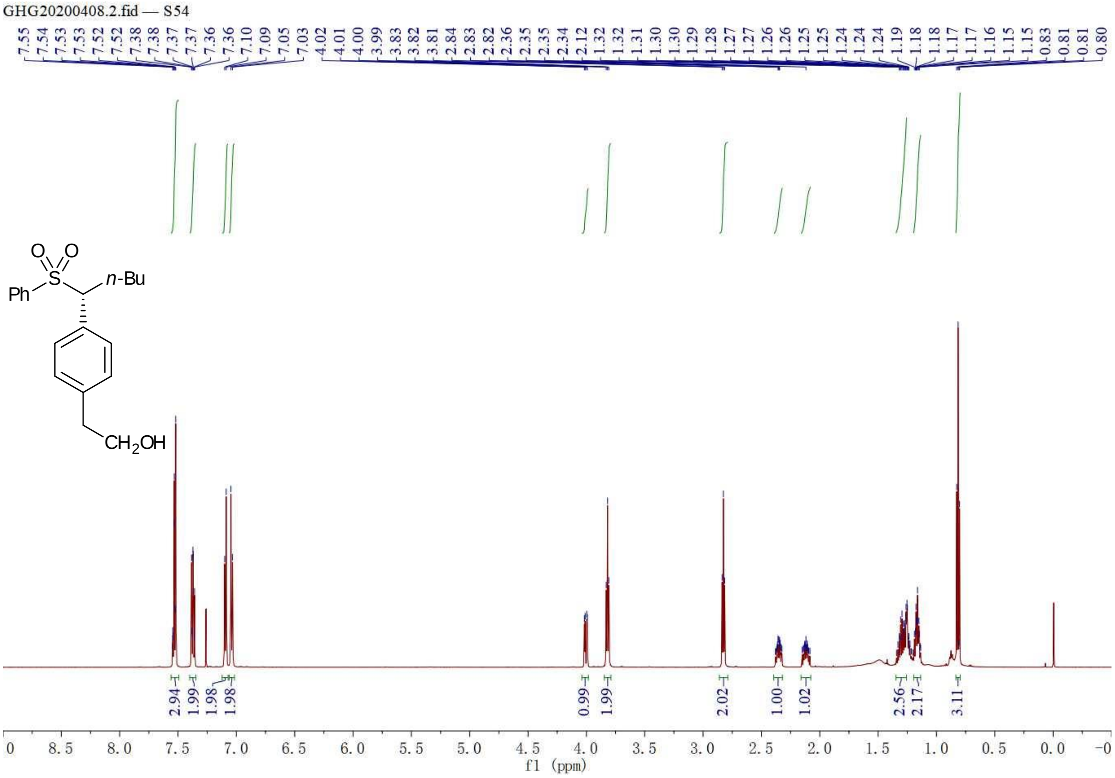

28, ^{13}C NMR (151 MHz, CDCl$_3$)

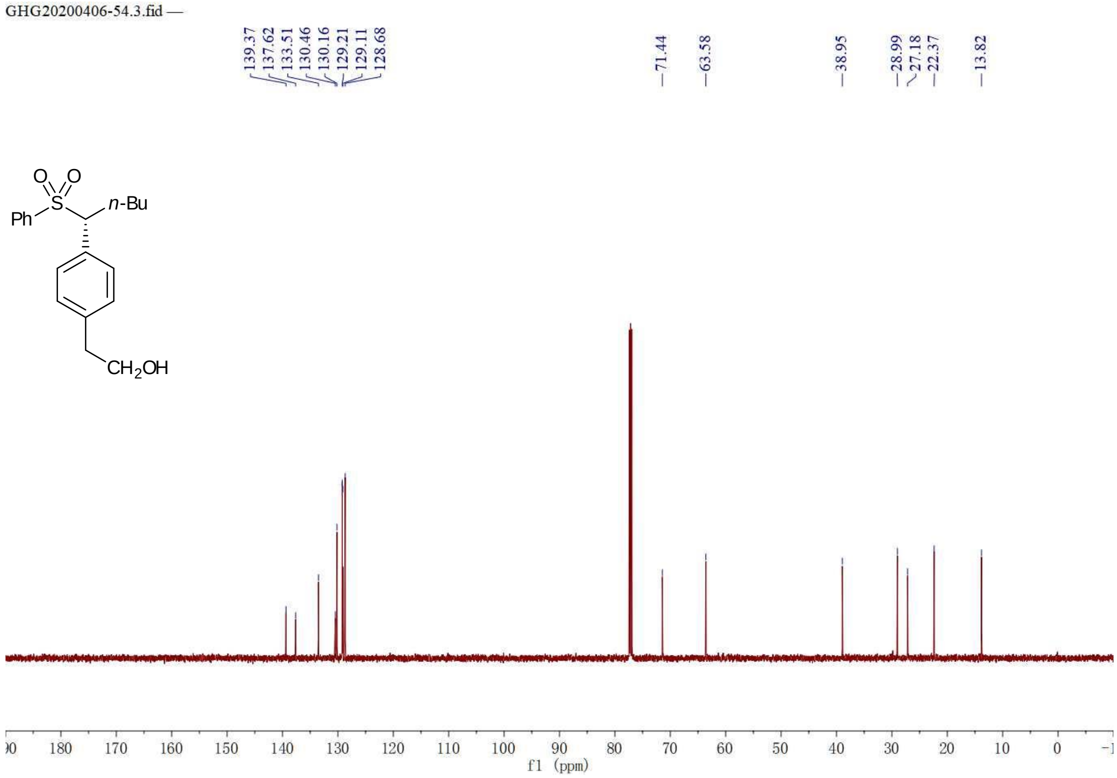

29, ^{1}H-NMR (600 MHZ, CDCl$_3$)

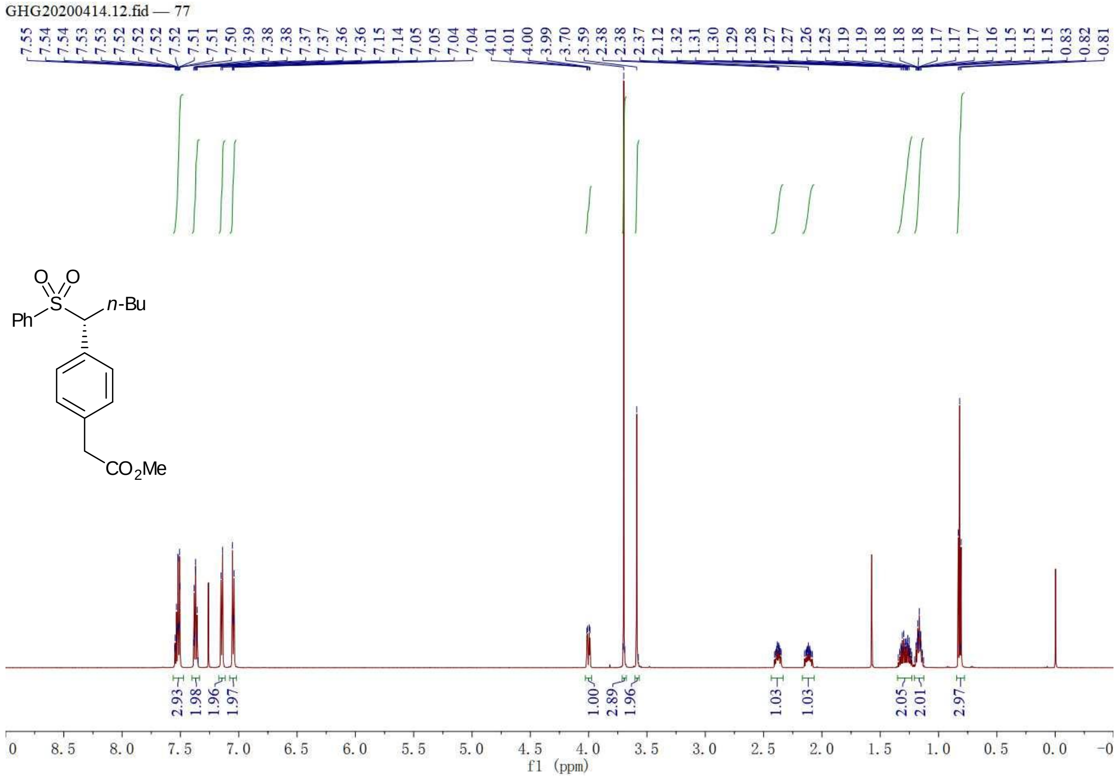

29, ^{13}C NMR (151 MHz, $CDCl_3$)

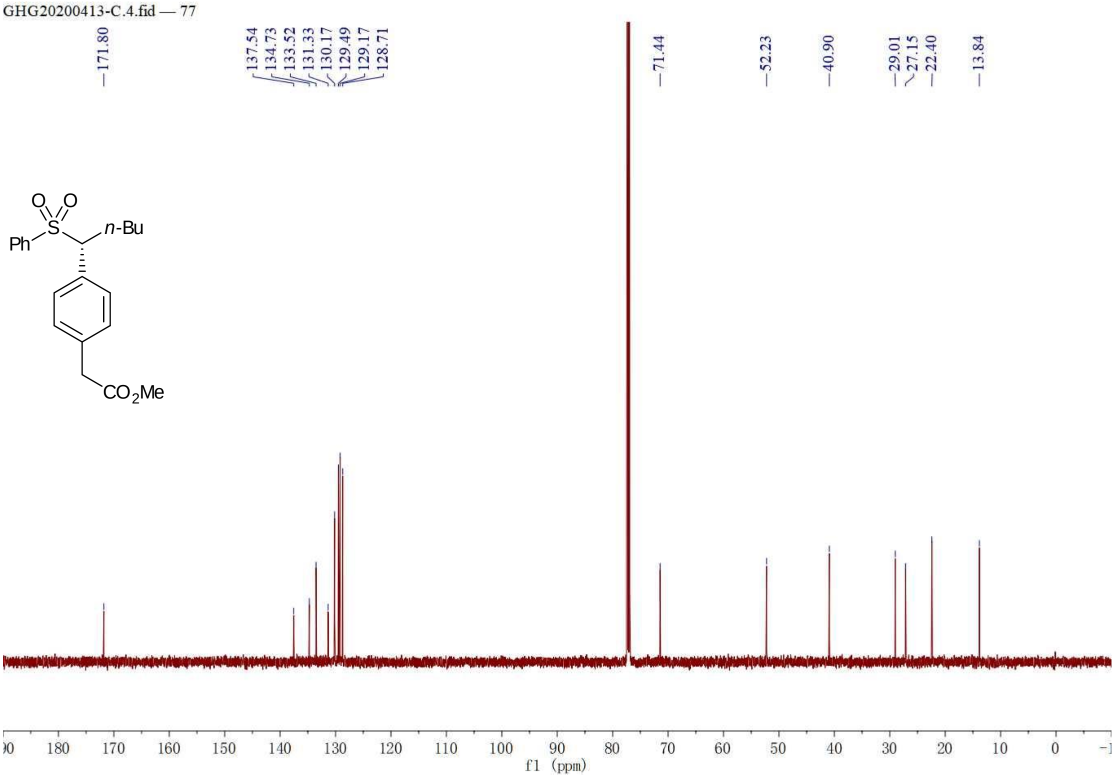

30, ^{1}H-NMR (600 MHZ, $CDCl_3$)

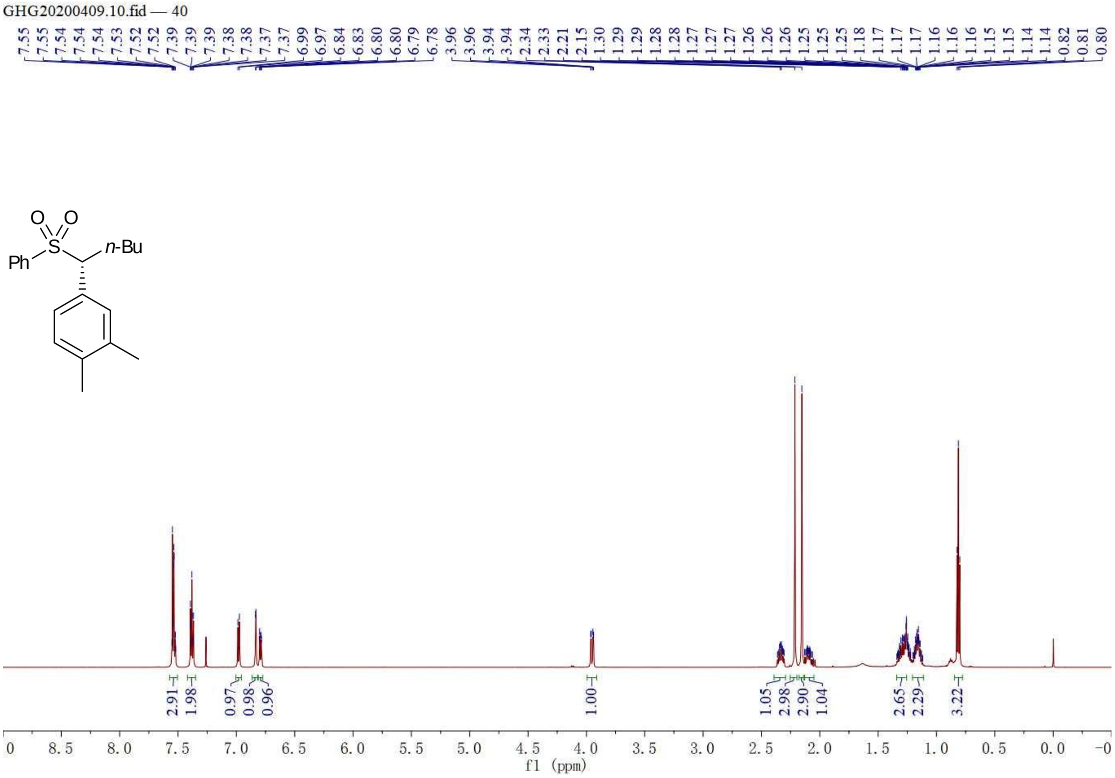

30, ^{13}C NMR (151 MHz, CDCl$_3$)

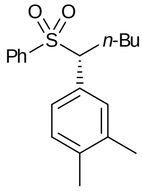

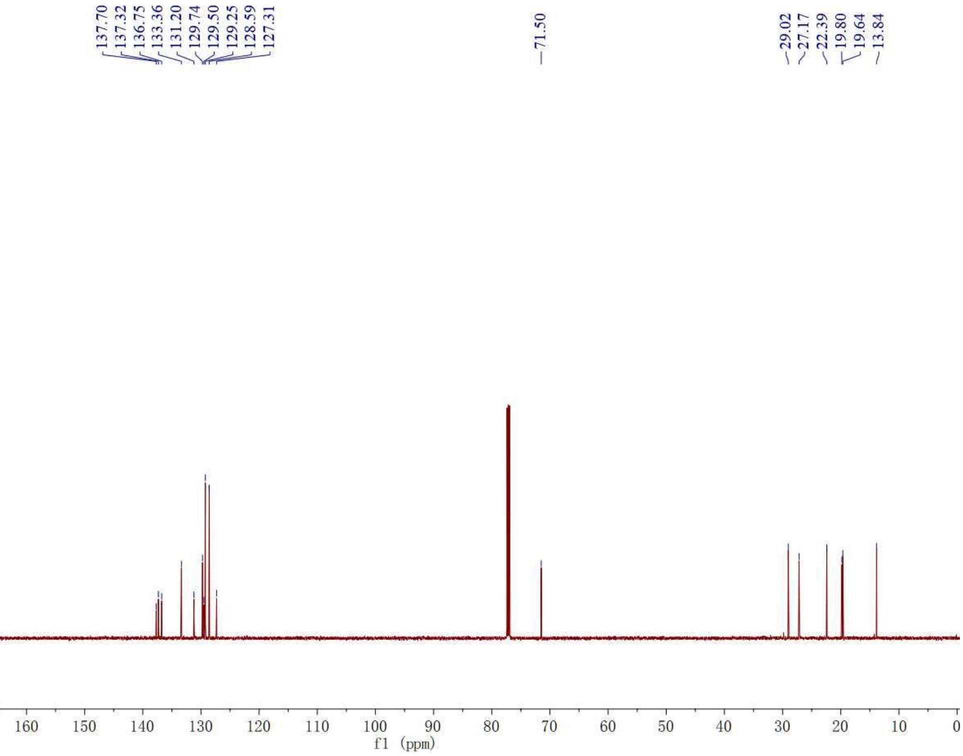

31, ^{1}H-NMR (600 MHZ, CDCl$_3$)

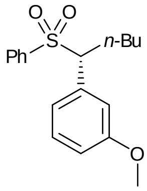

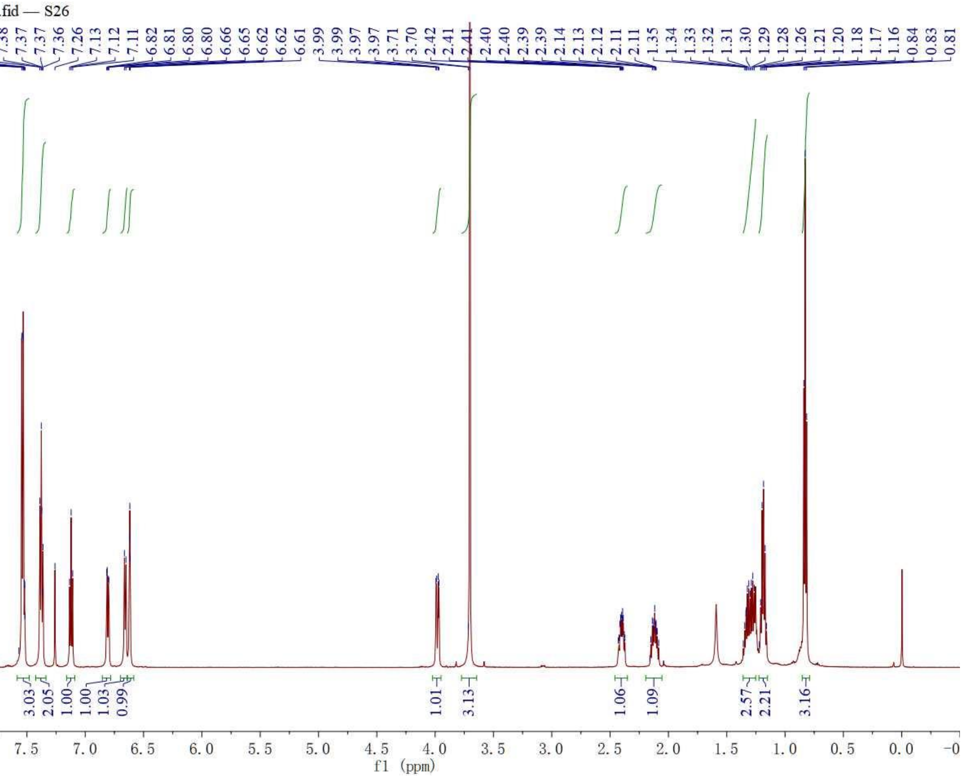

31, ^{13}C NMR (151 MHz, CDCl$_3$)

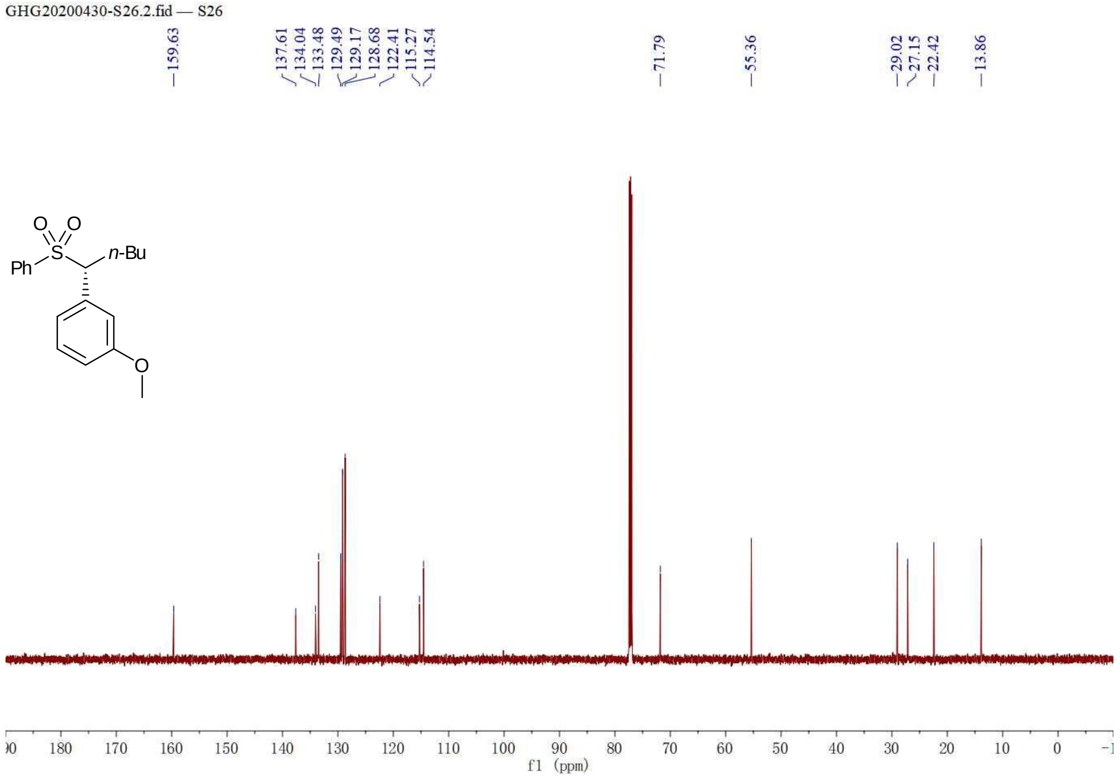

32, ^{1}H-NMR (600 MHZ, CDCl$_3$)

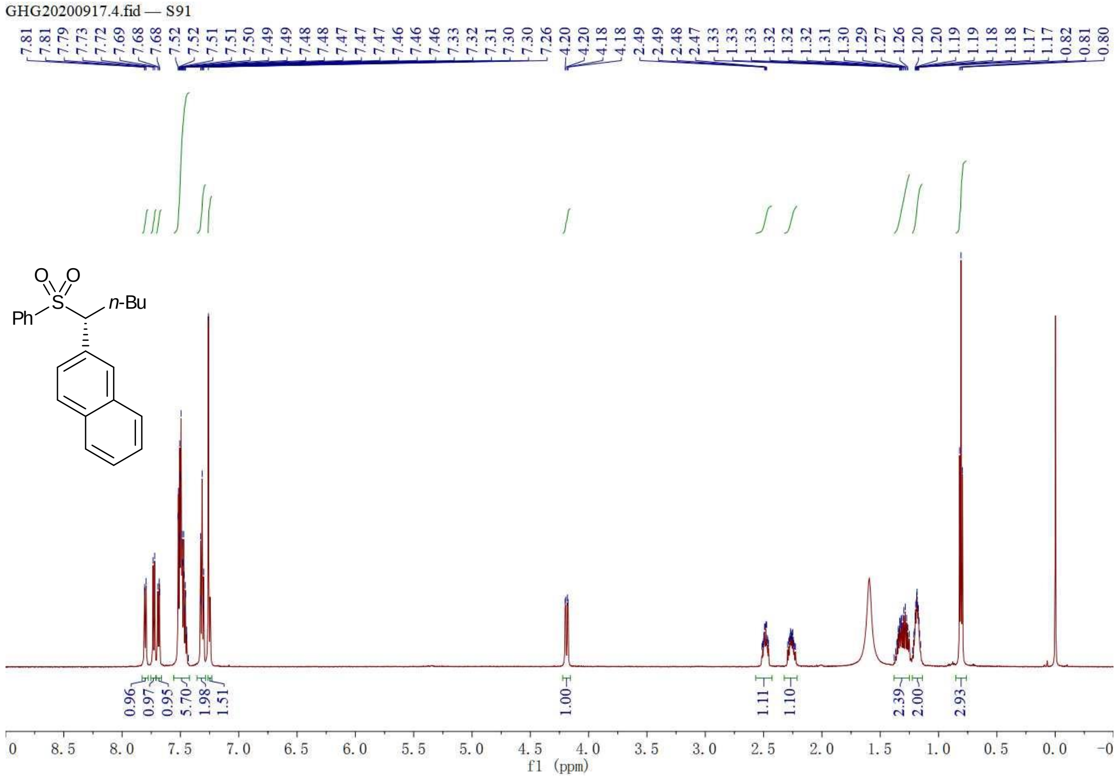

32, ^{13}C NMR (151 MHz, $CDCl_3$)

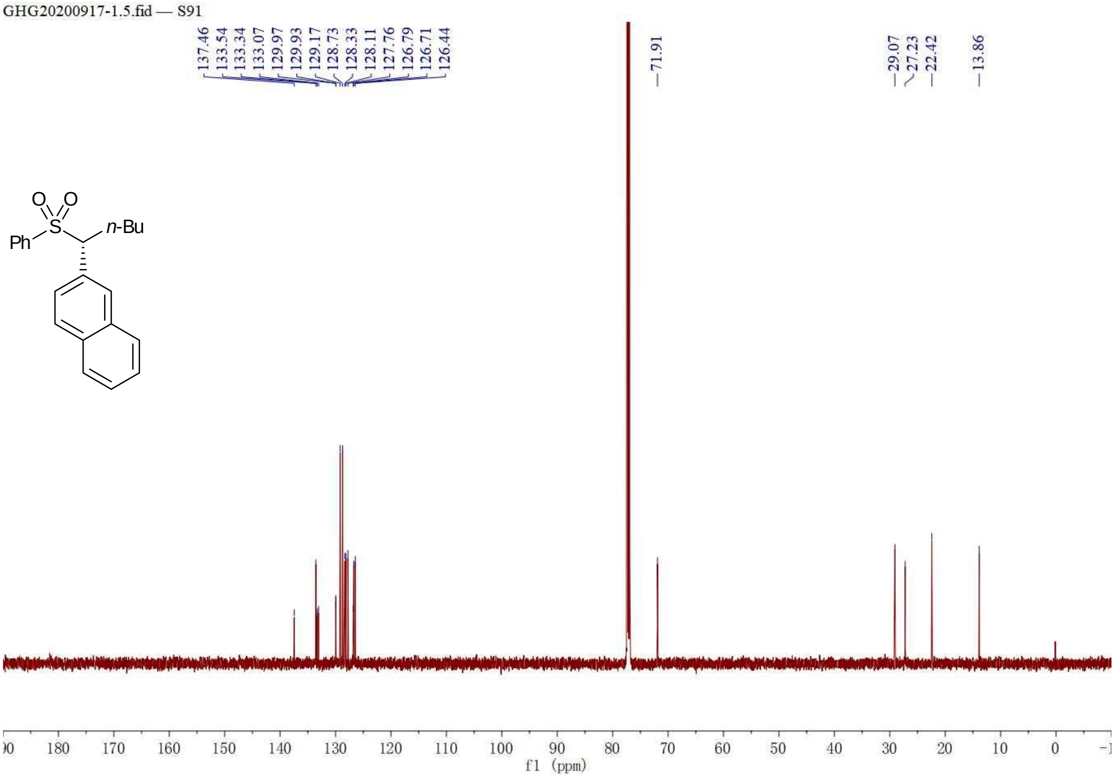

33, ^{1}H-NMR (600 MHZ, $CDCl_3$)

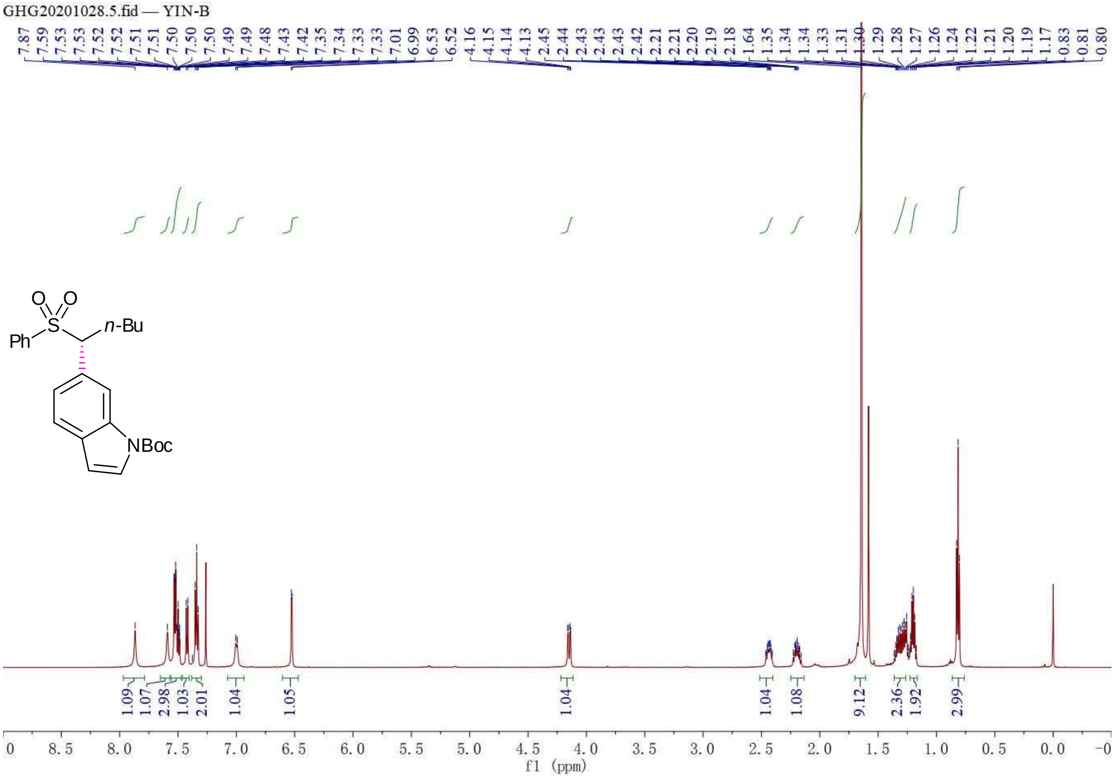

33, ^{13}C NMR (151 MHz, $CDCl_3$)

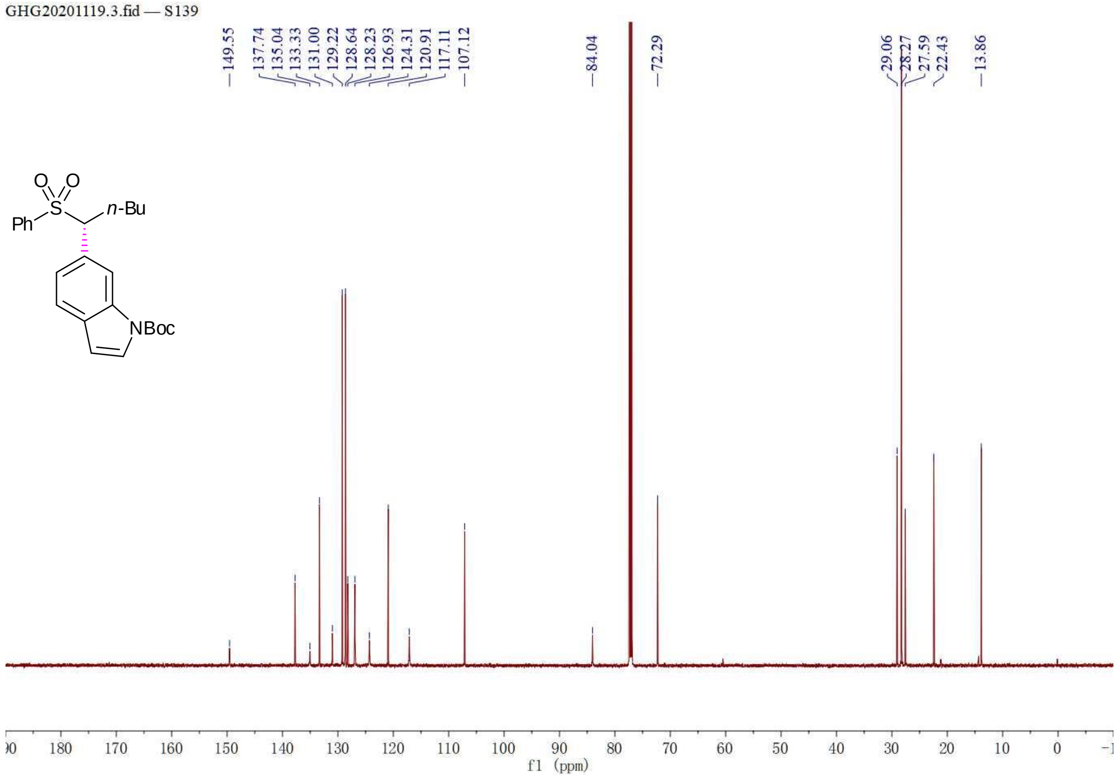

34, ^{1}H-NMR (600 MHZ, $CDCl_3$)

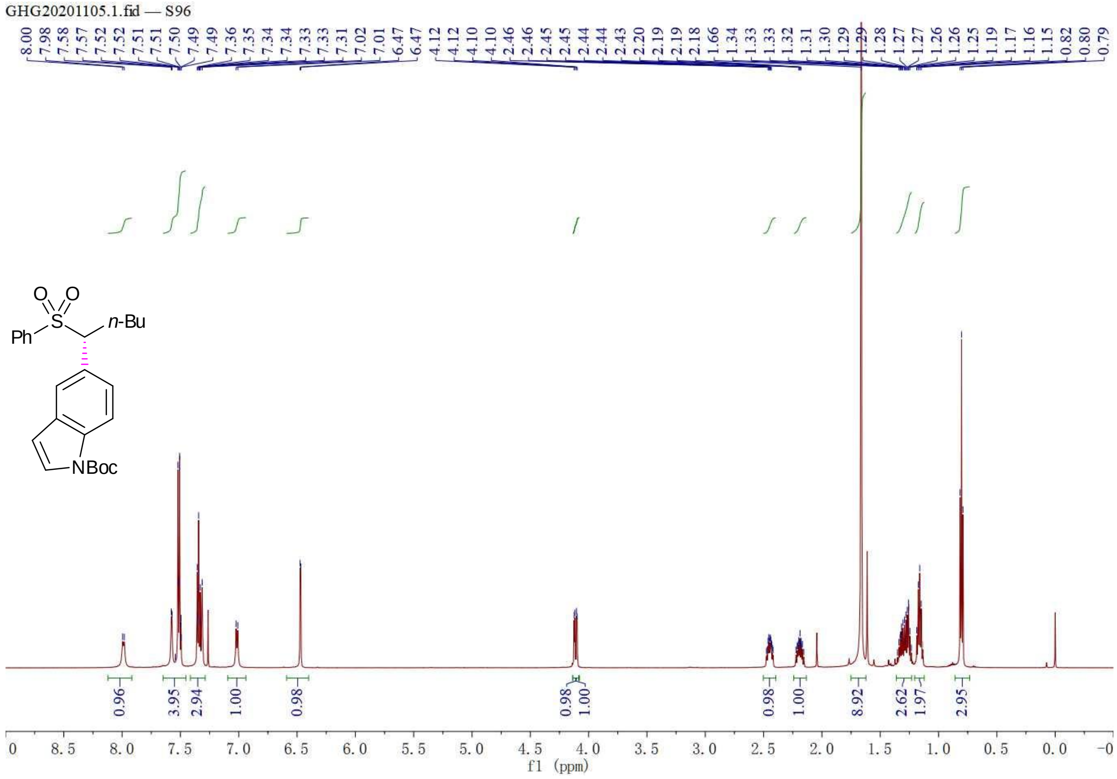

34, ^{13}C NMR (151 MHz, $CDCl_3$)

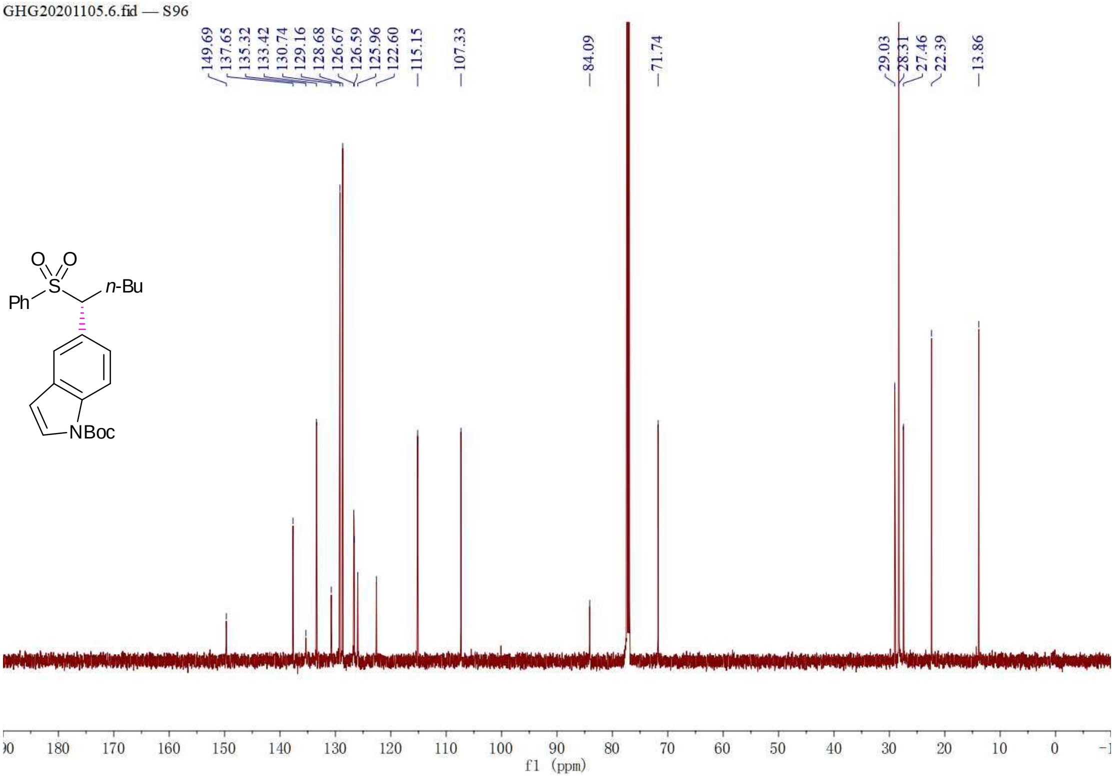

35, ^{1}H-NMR (600 MHZ, $CDCl_3$)

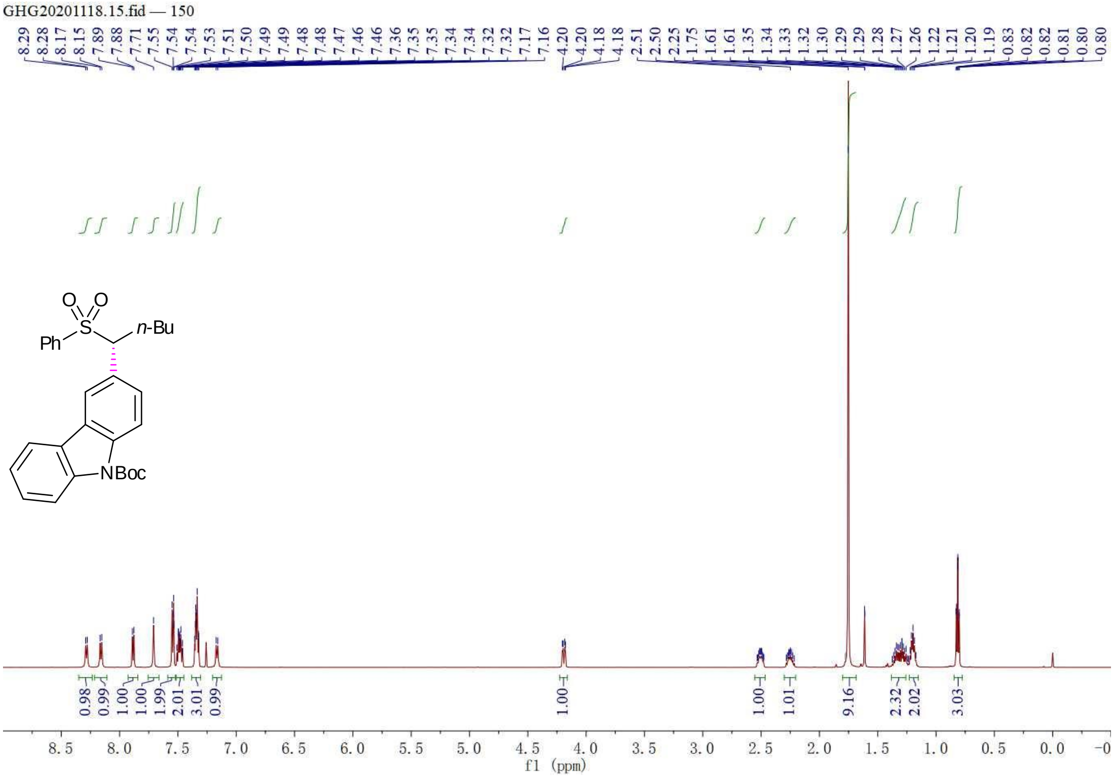

35, ^{13}C NMR (151 MHz, CDCl$_3$)

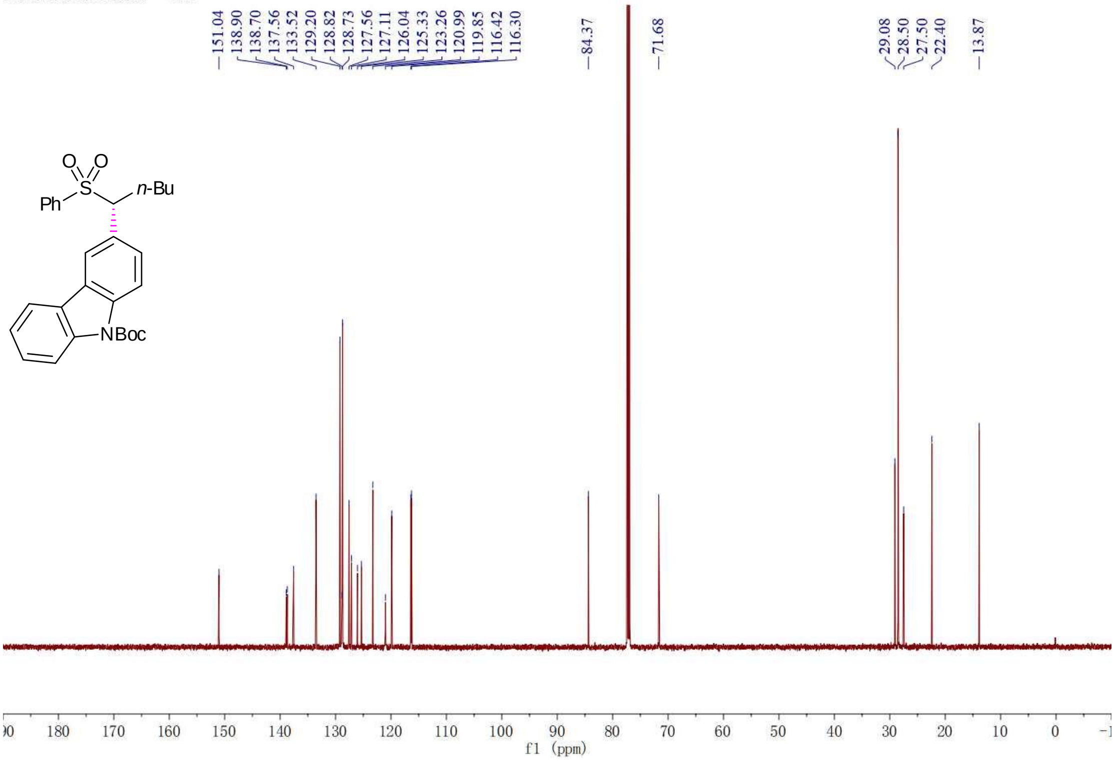

36, ^{1}H-NMR (600 MHZ, CDCl$_3$)

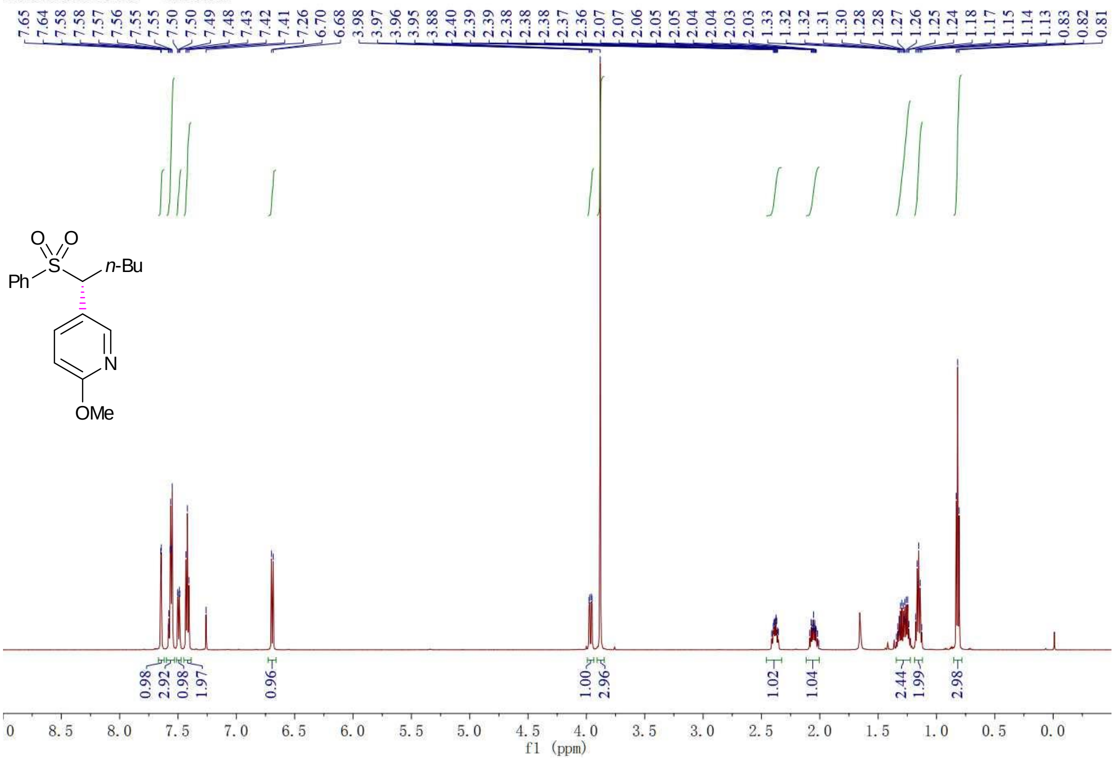

36, ^{13}C NMR (151 MHz, CDCl$_3$)

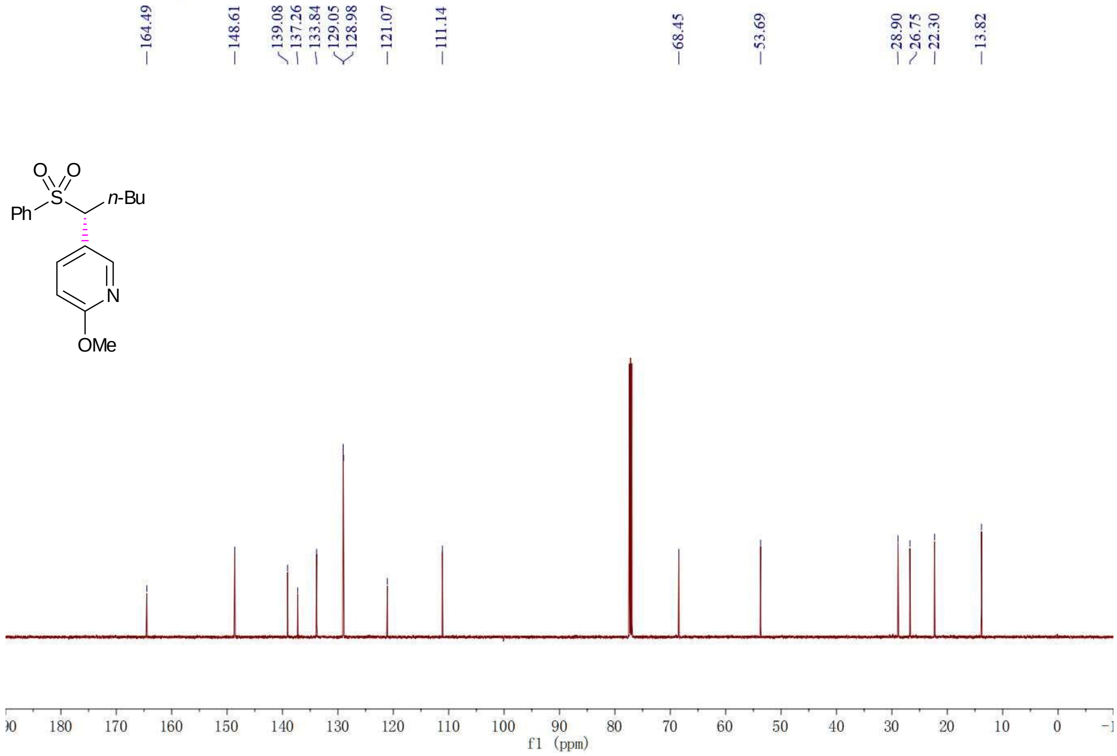

37, ^{1}H-NMR (600 MHZ, CDCl$_3$)

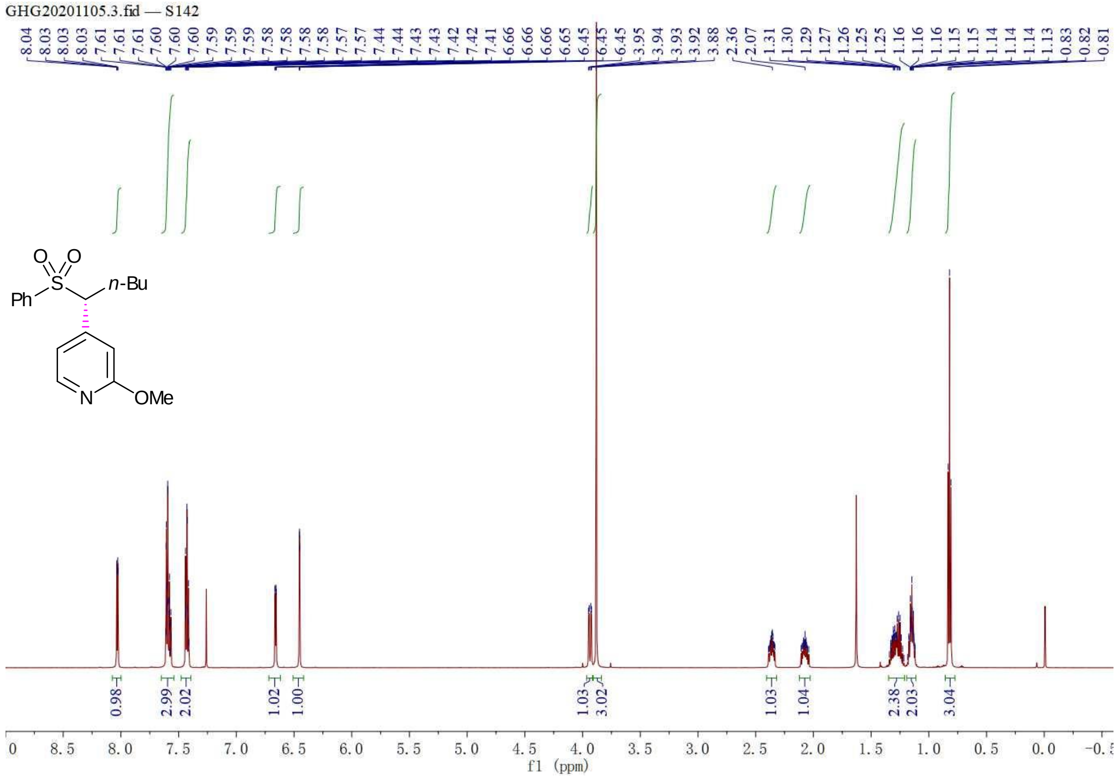

37, ^{13}C NMR (151 MHz, CDCl$_3$)

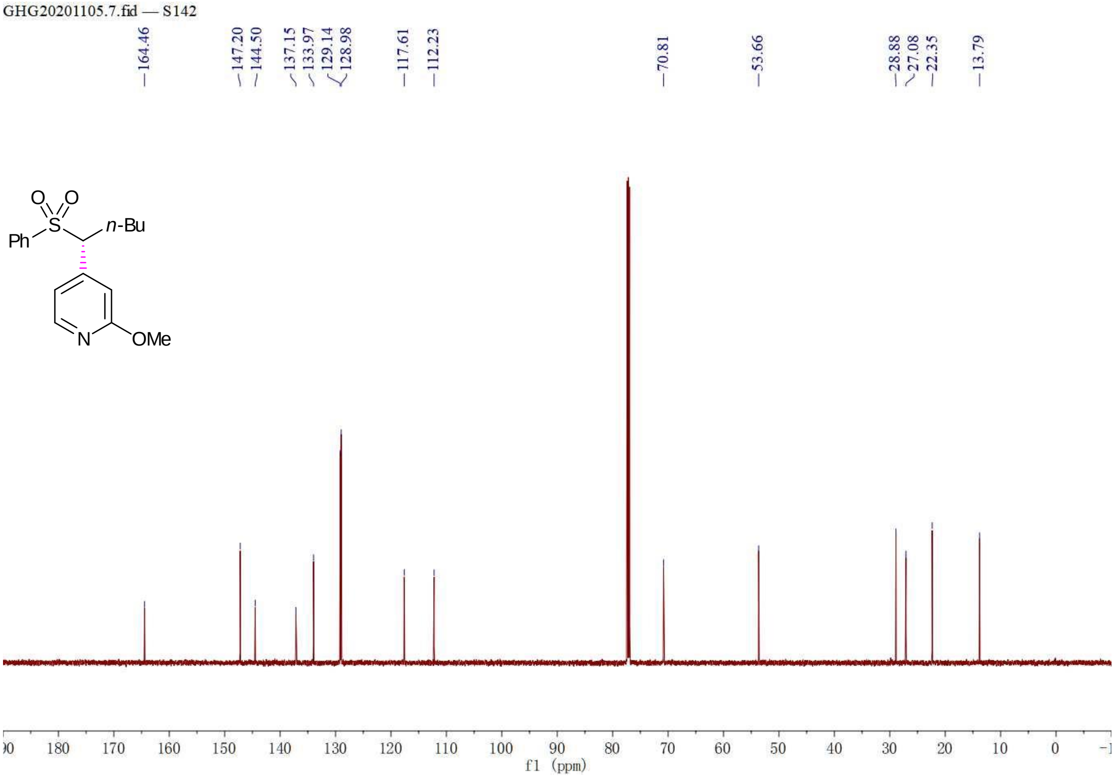

38, ^{1}H-NMR (600 MHZ, CDCl$_3$)

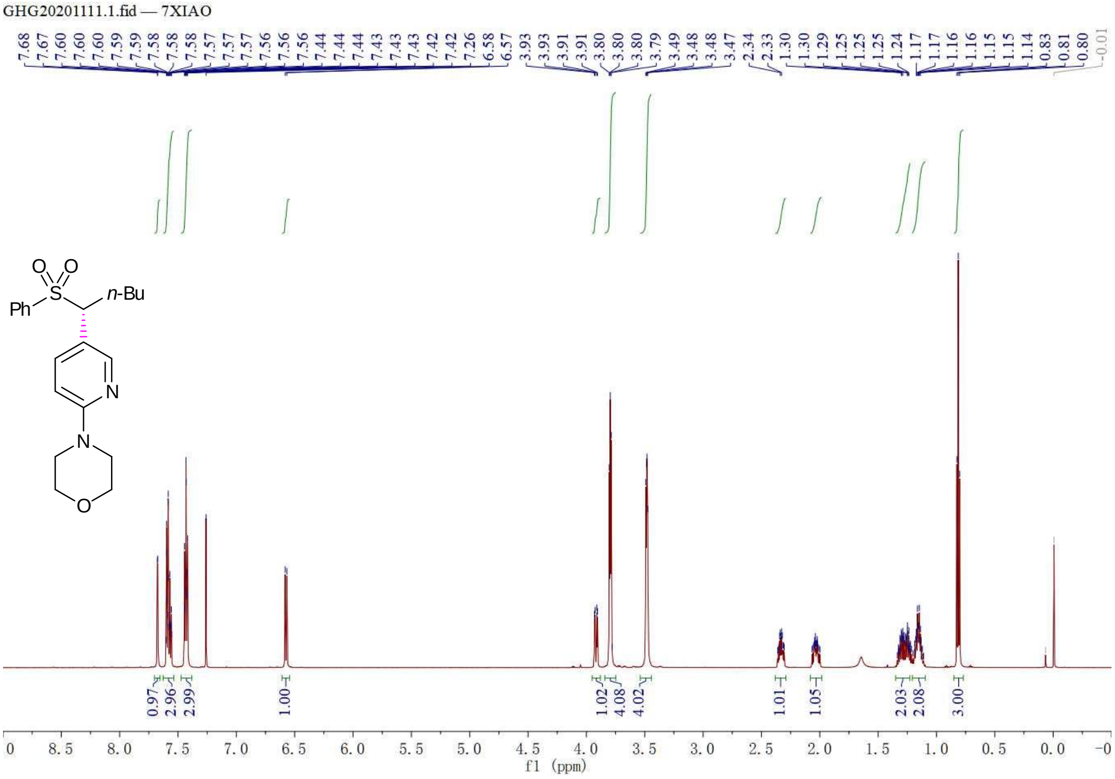

38, ^{13}C NMR (151 MHz, $CDCl_3$)

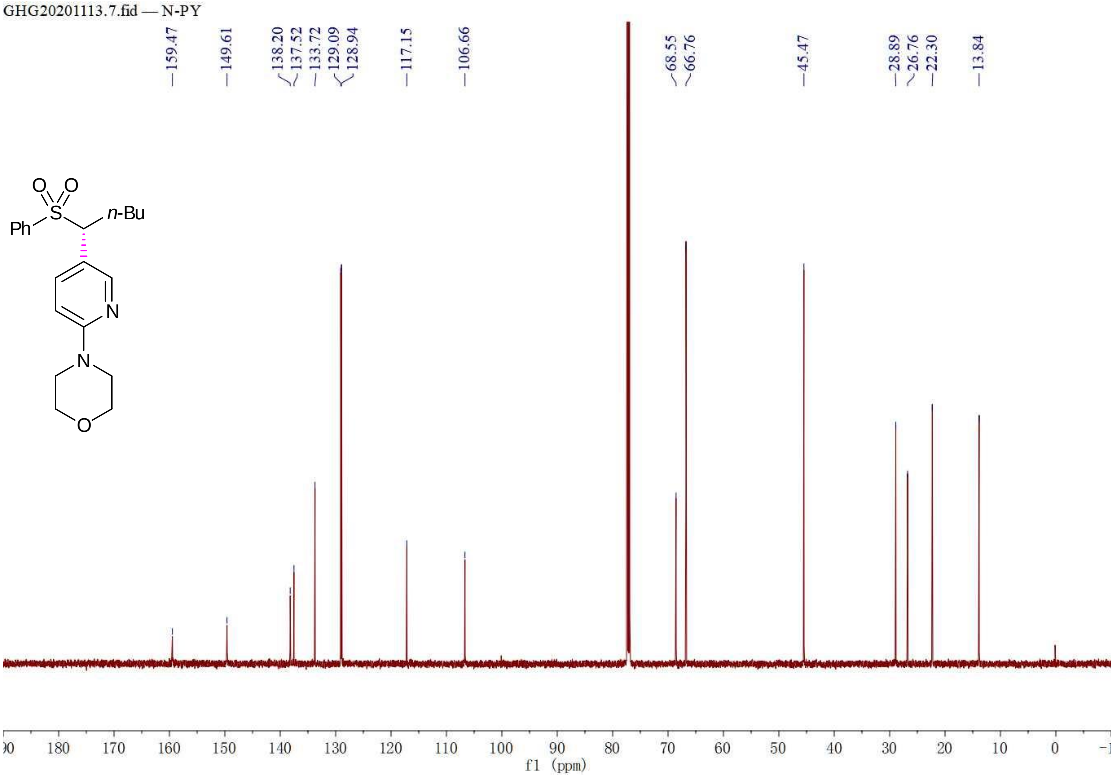

39, ^{1}H-NMR (600 MHZ, $CDCl_3$)

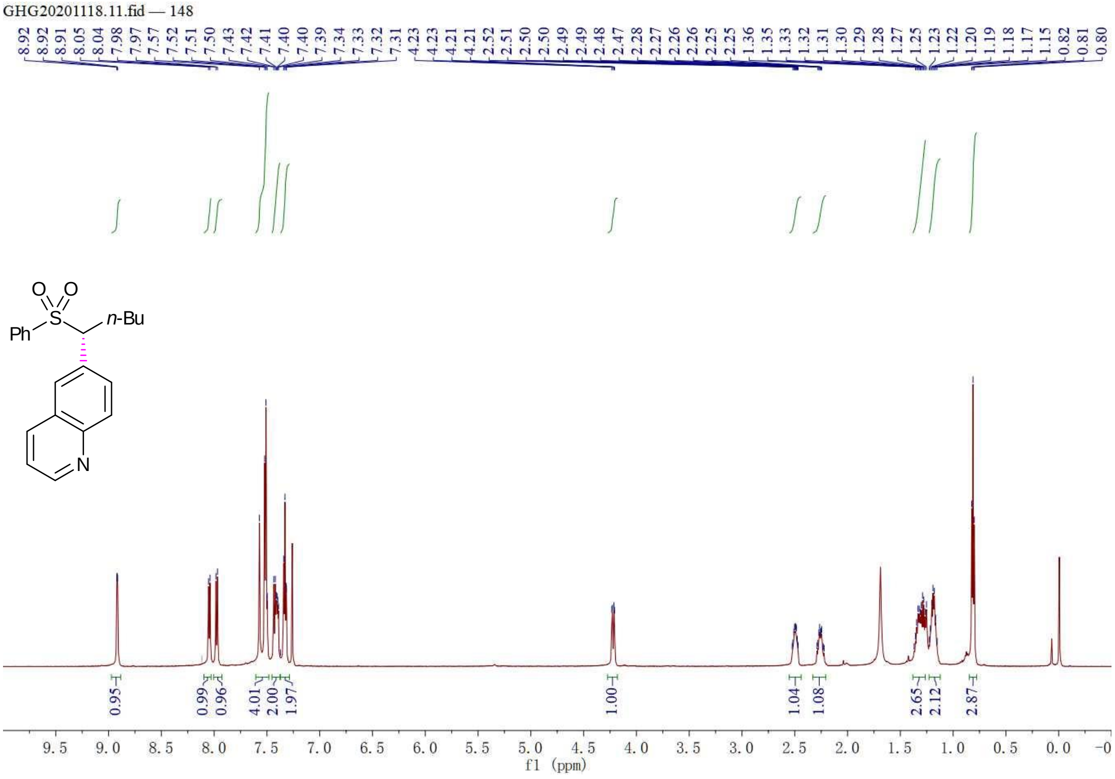

39, ^{13}C NMR (151 MHz, $CDCl_3$)

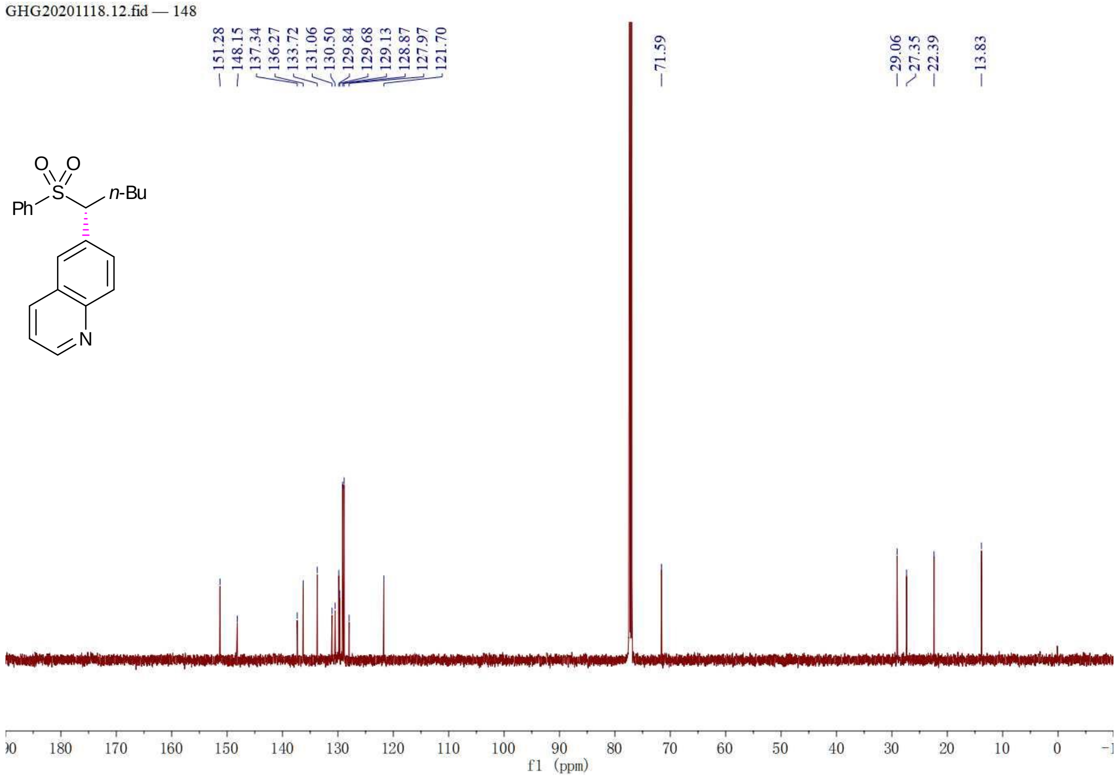

40, ^{1}H-NMR (600 MHZ, $CDCl_3$)

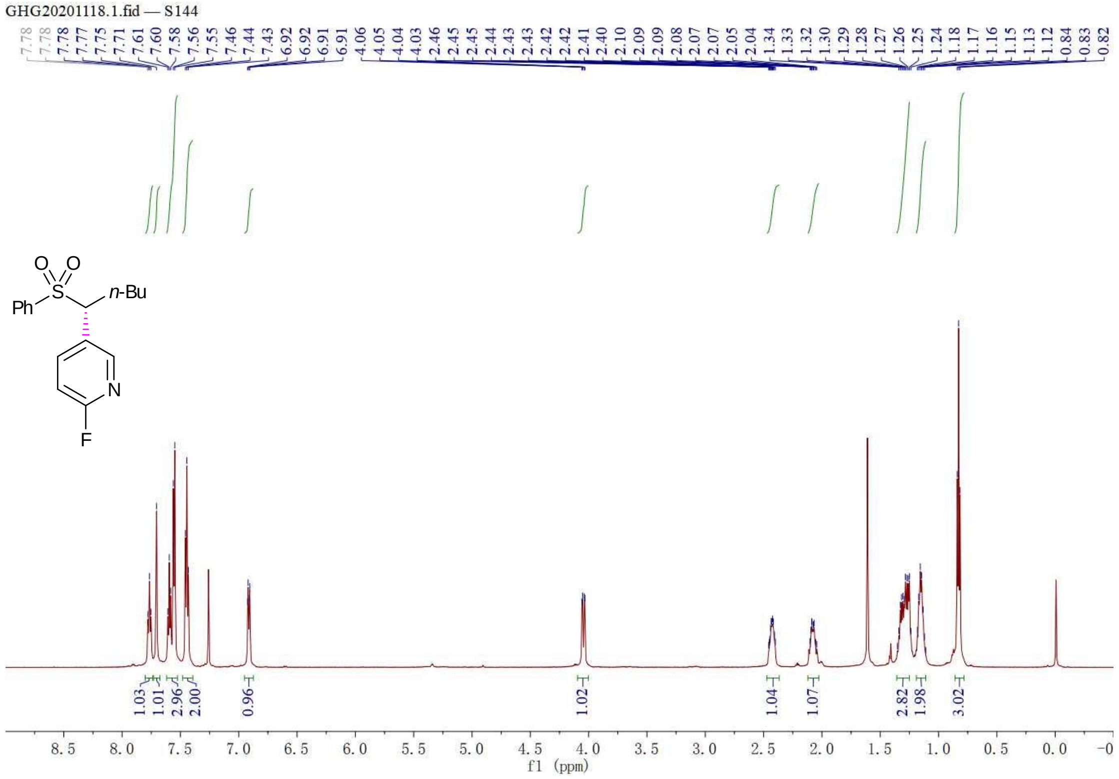

40, ^{13}C NMR (151 MHz, $CDCl_3$)

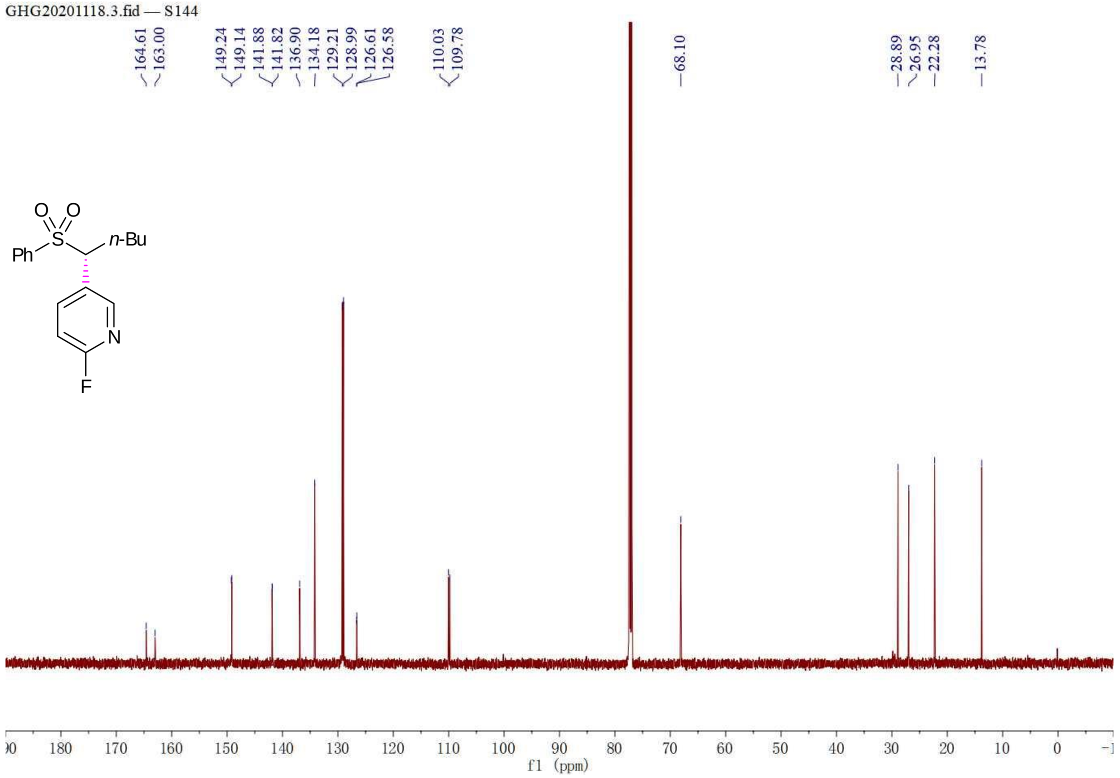

40, ^{19}F NMR (567 MHz, $CDCl_3$)

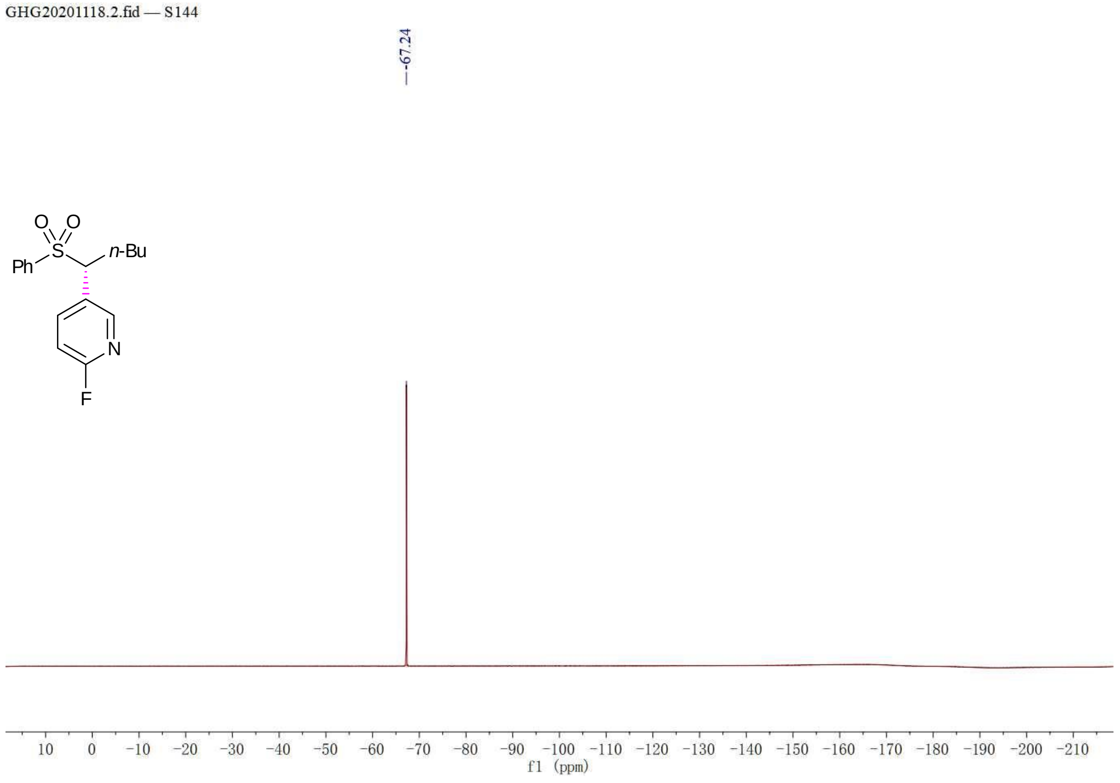

41, ^{1}H-NMR (600 MHZ, $CDCl_3$)

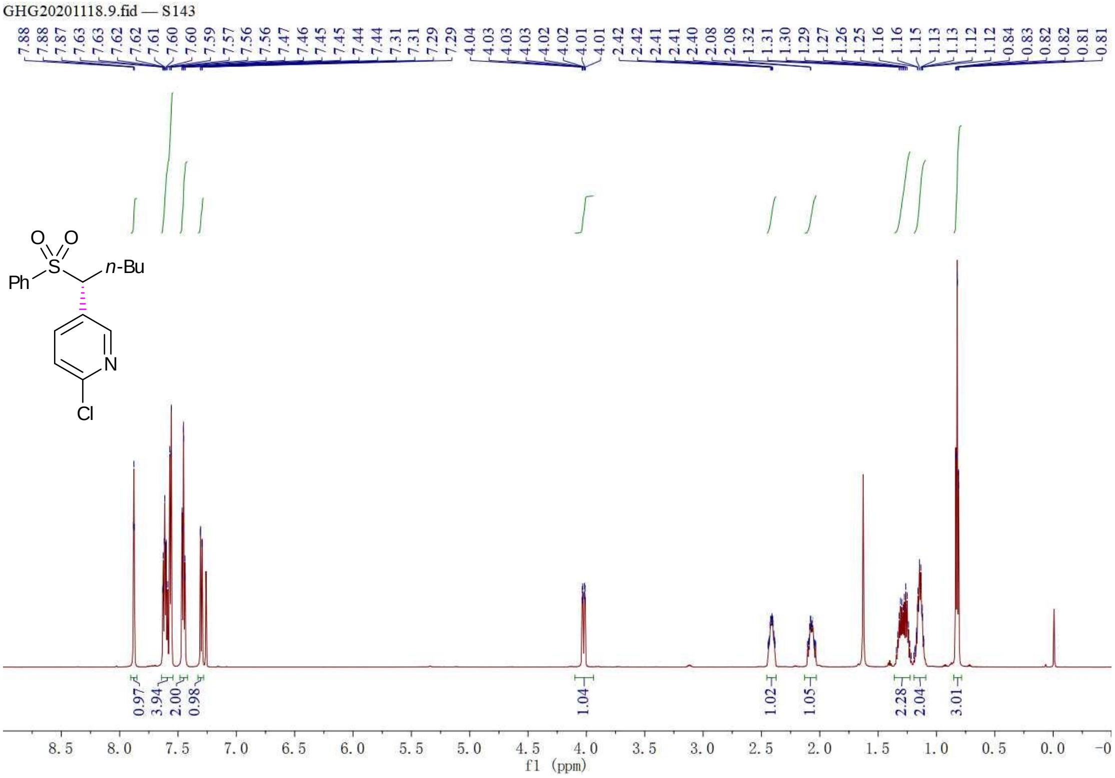

41, ^{13}C NMR (151 MHz, $CDCl_3$)

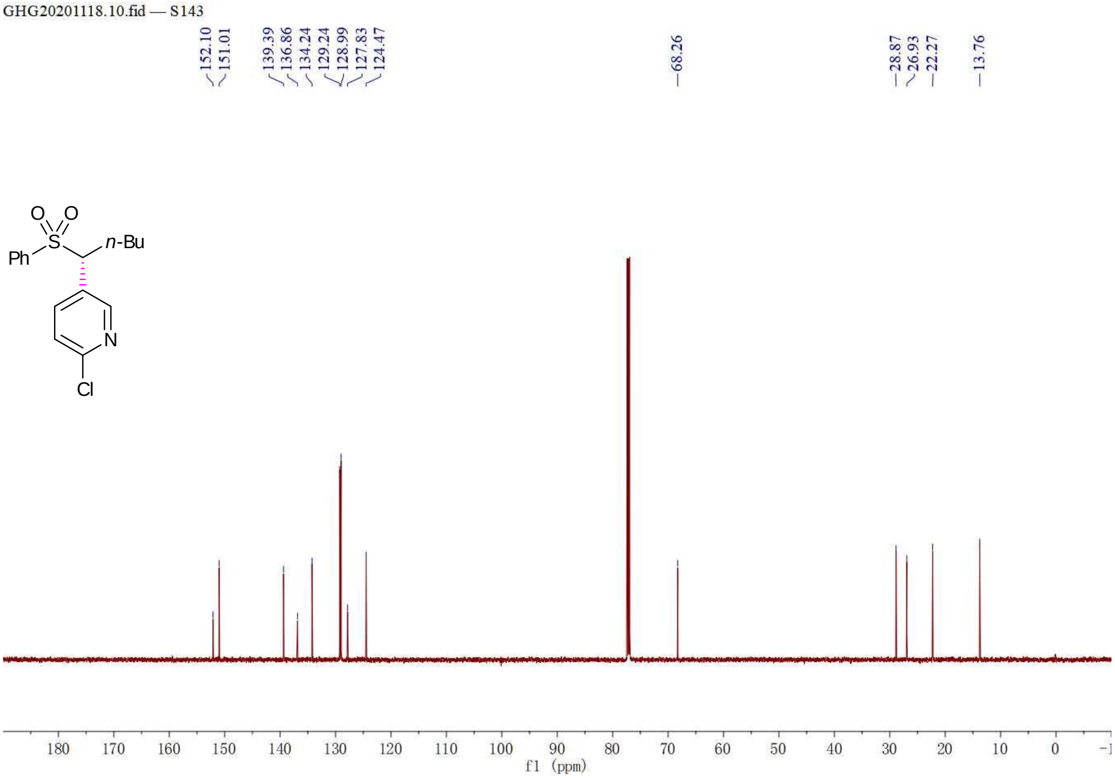

42, ¹H-NMR (600 MHZ, CDCl₃)

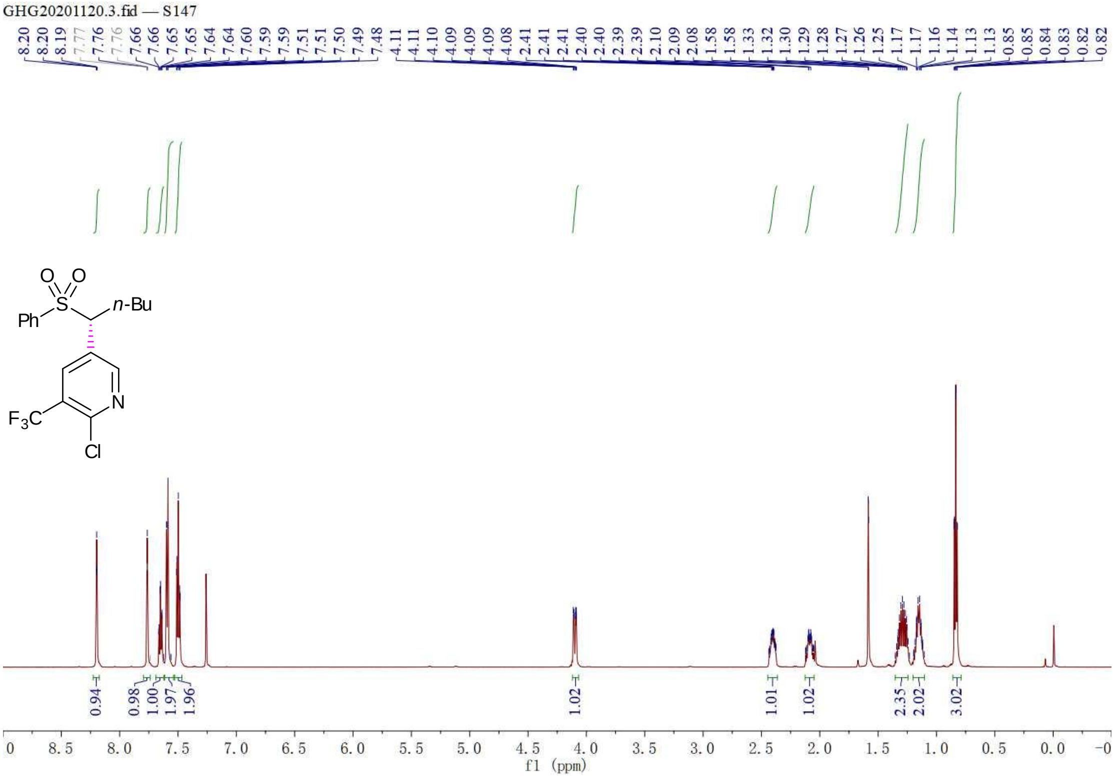

42, ¹³C NMR (151 MHz, CDCl₃)

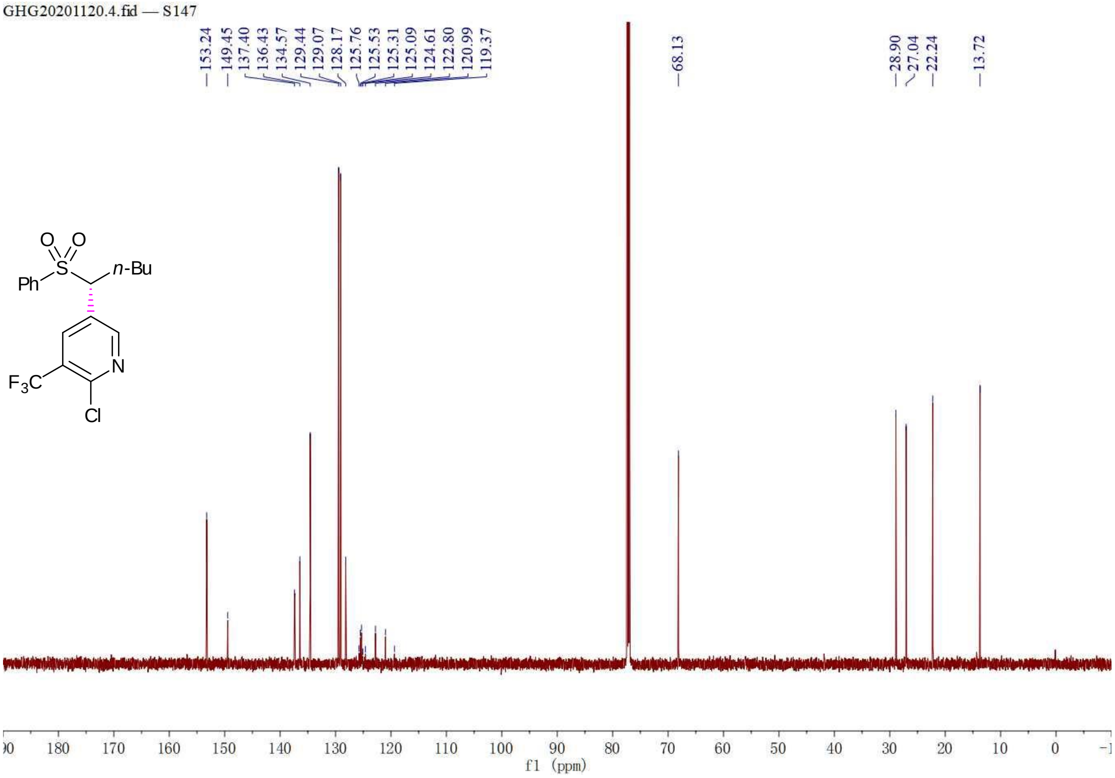

42, ^{19}F NMR (567 MHz, $CDCl_3$)

GHG20201220.2.fid — S147

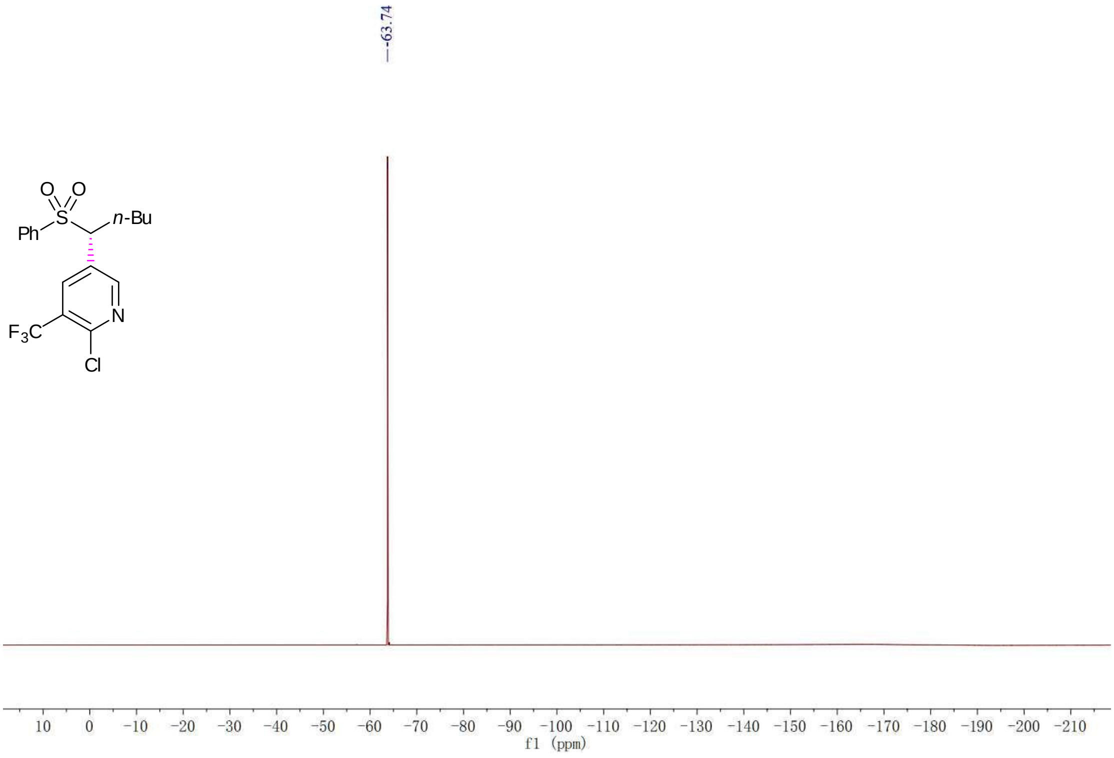

43, ^{1}H-NMR (600 MHZ, $CDCl_3$)

GHG20201020-1.2.fid — CF3

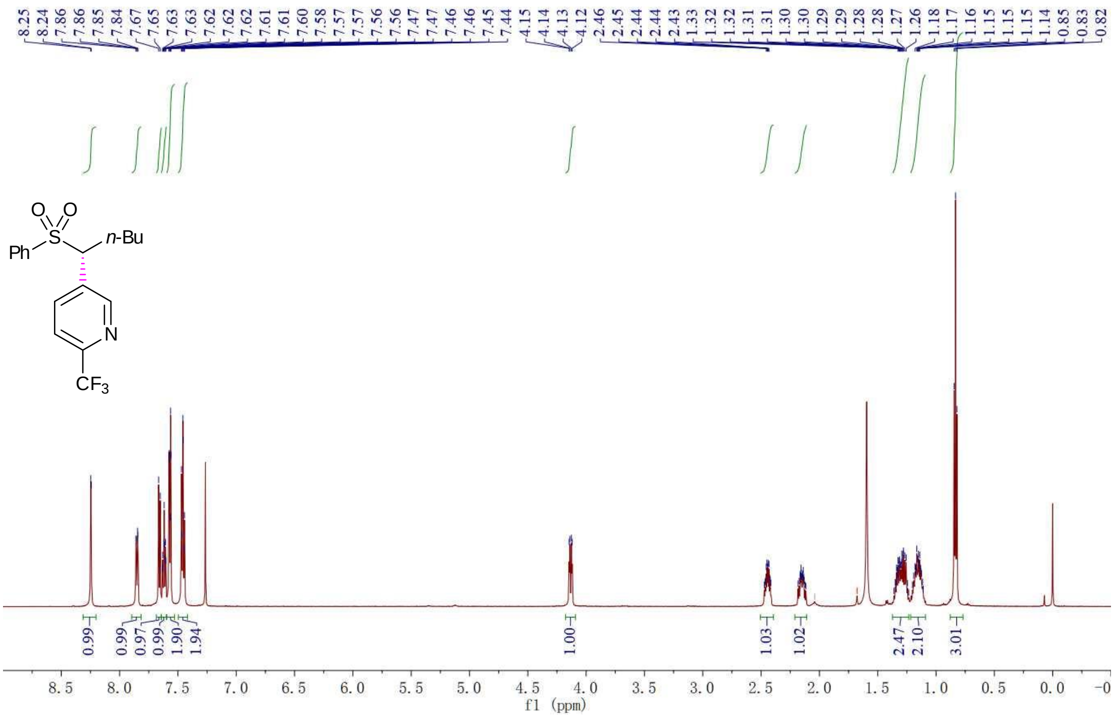

43, ^{13}C NMR (151 MHz, $CDCl_3$)

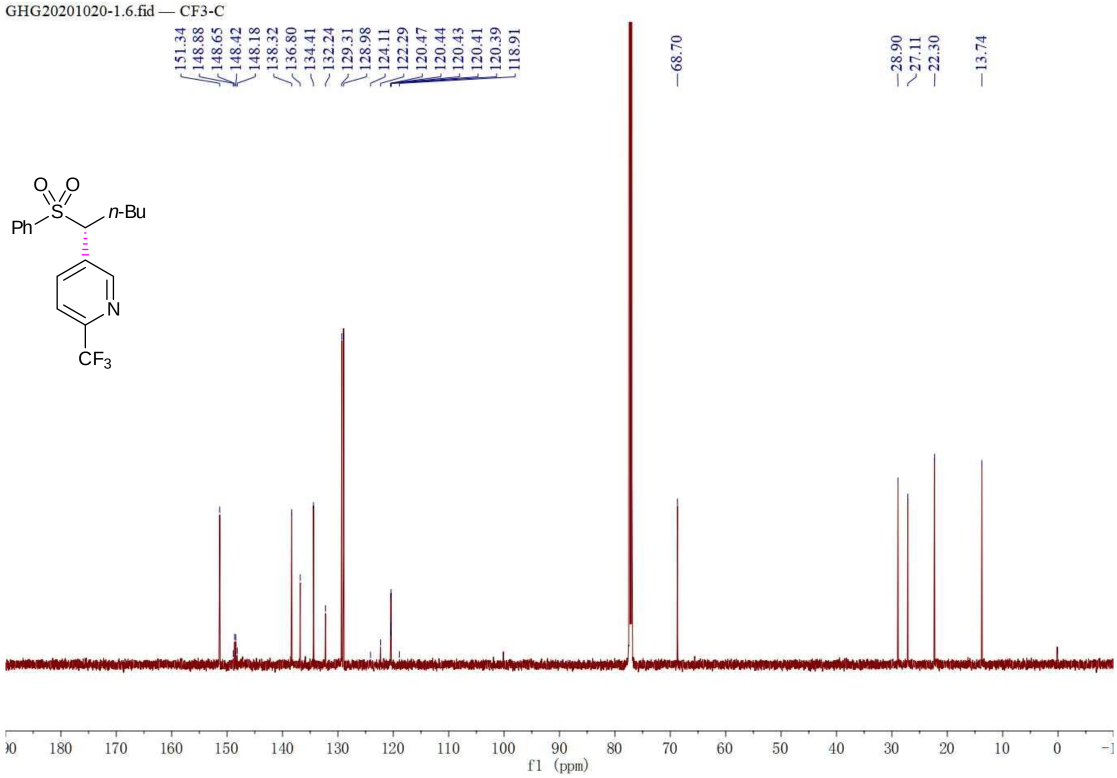

43, ^{19}F NMR (567 MHz, $CDCl_3$)

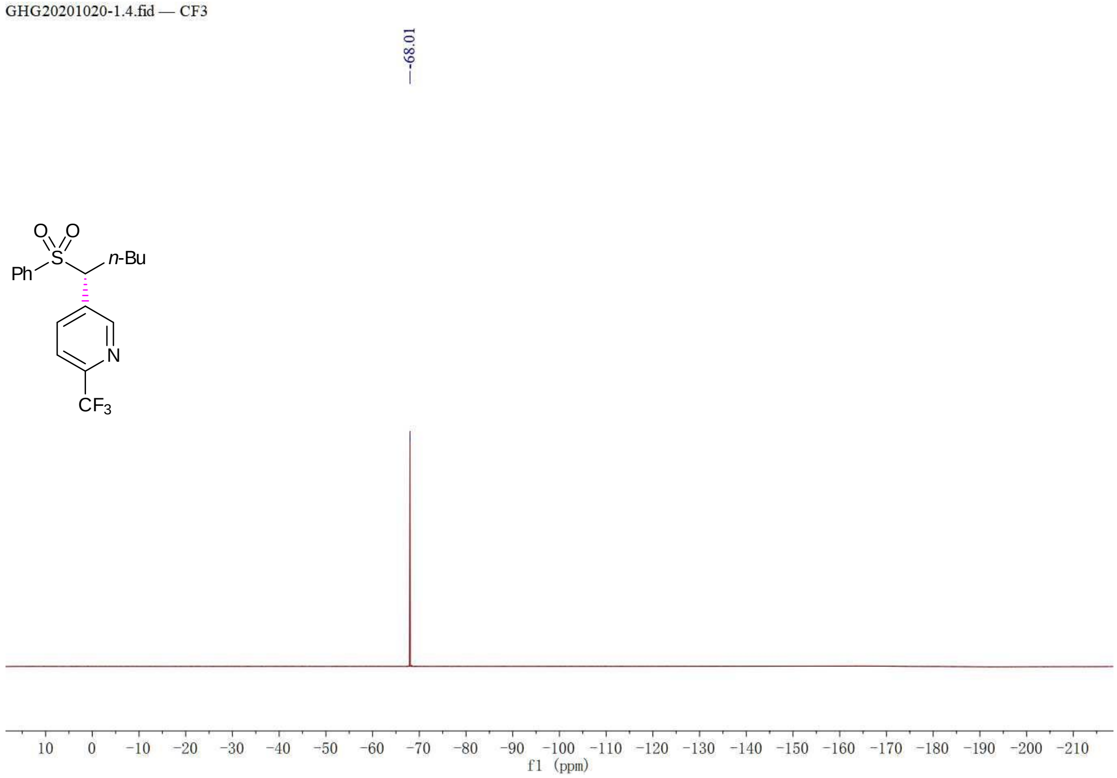

44, ¹H-NMR (600 MHZ, Acetone-d_6)

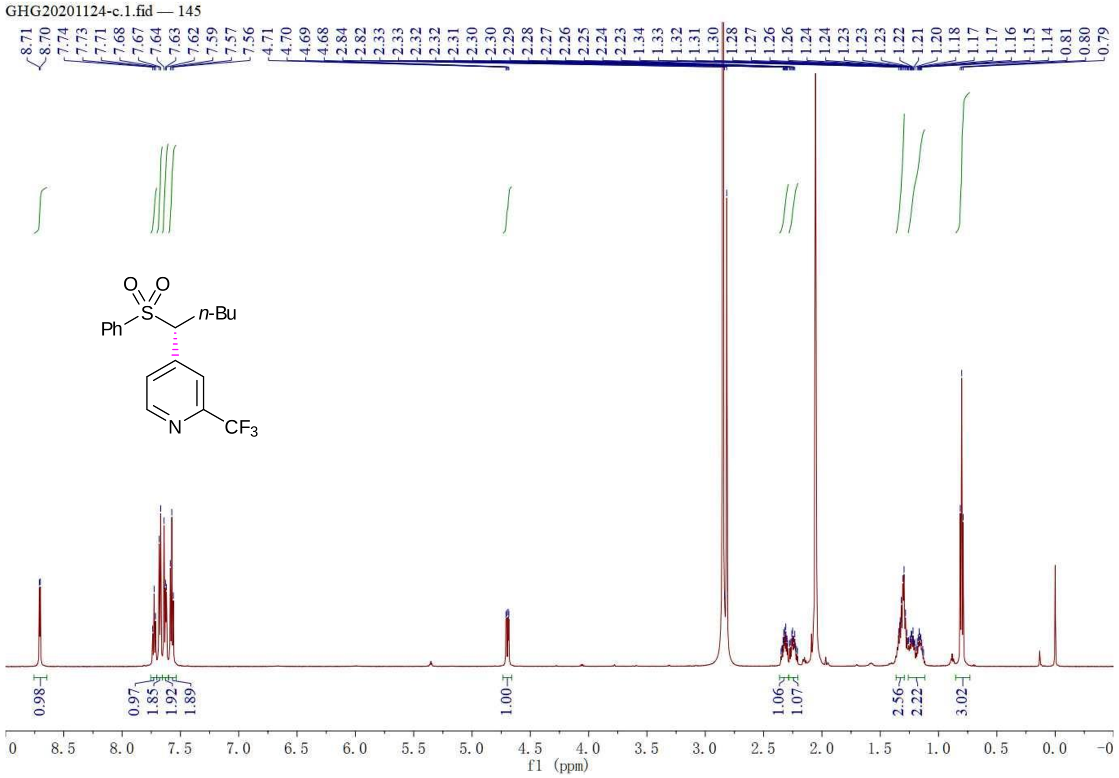

44, ¹³C NMR (151 MHz, Acetone-d_6)

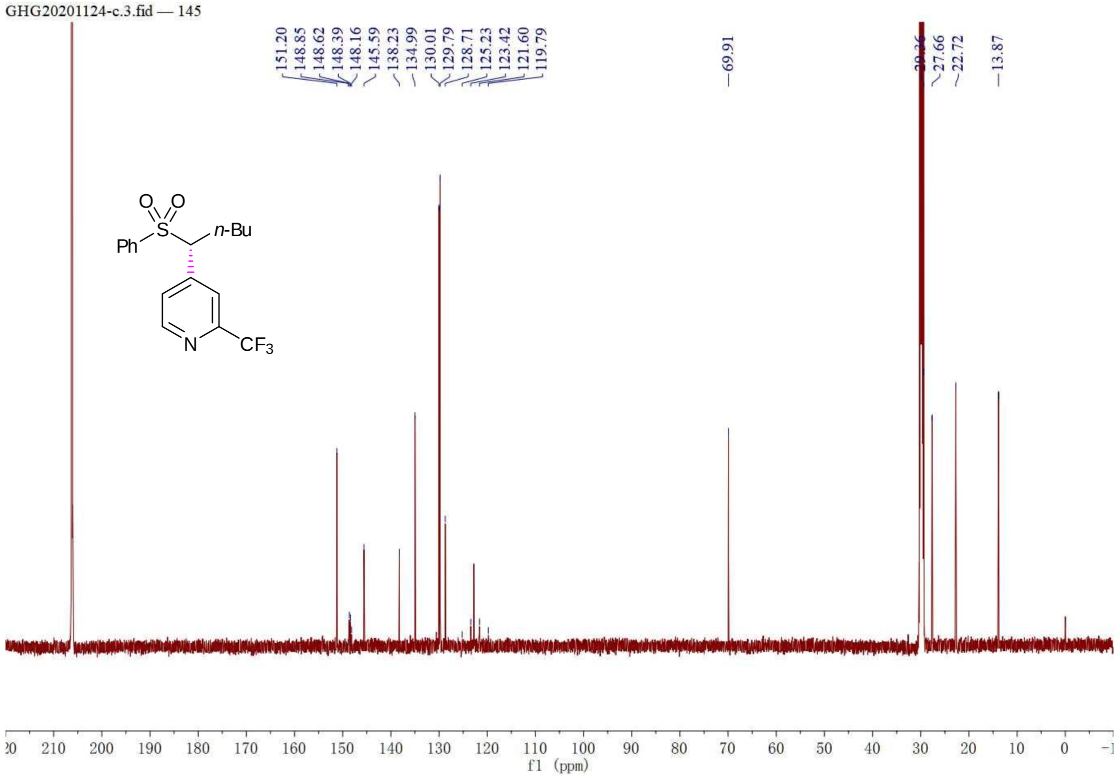

44, ^{19}F NMR (567 MHz, Acetone-d_6)

GHG20201124-c.2.fid — 145

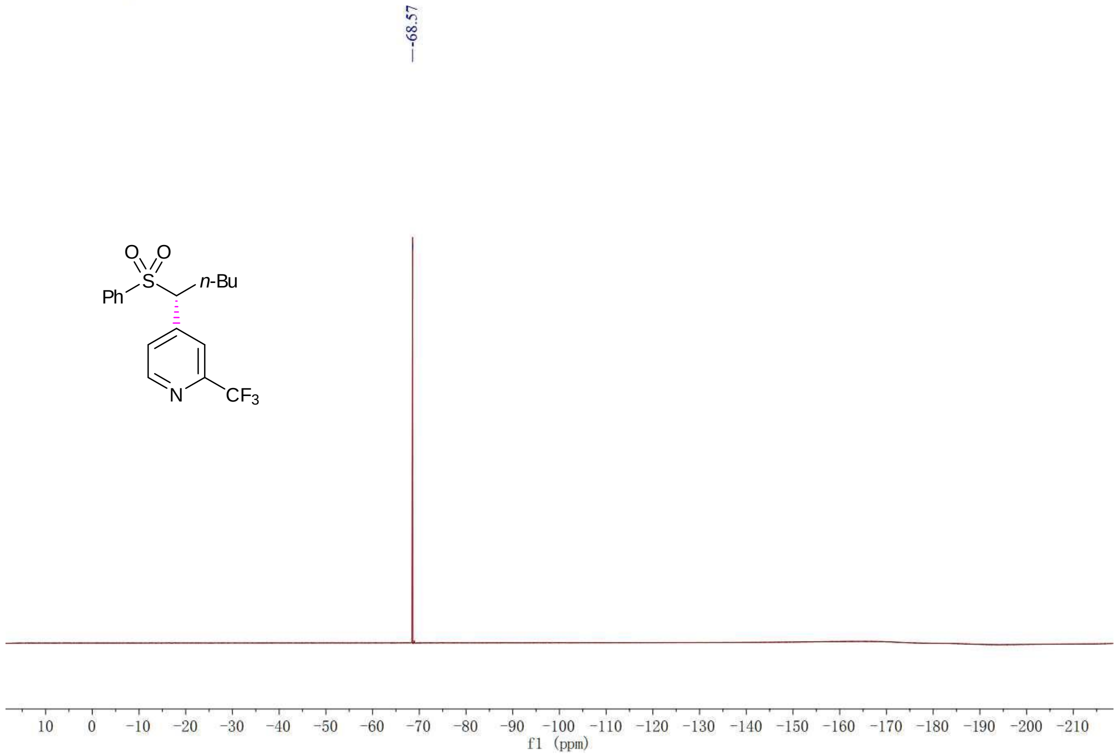

45, ^{1}H-NMR (600 MHZ, CDCl$_3$)

GHG20200409.15.fid — S68

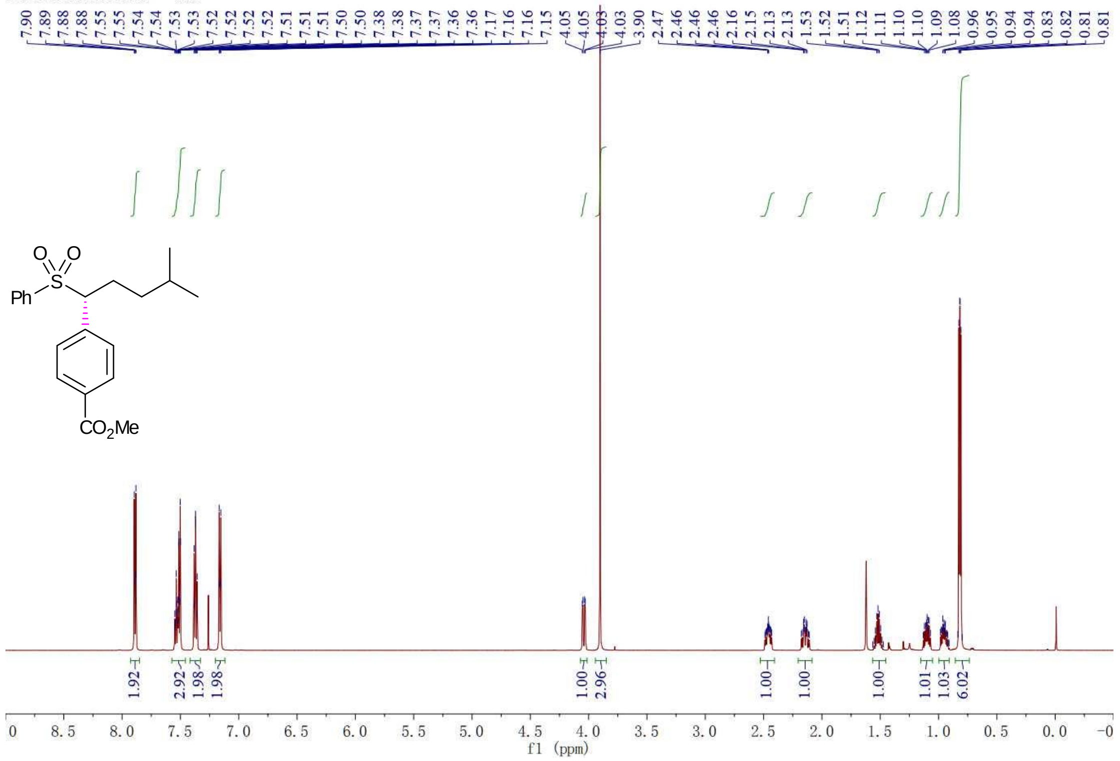

45, ^{13}C NMR (151 MHz, $CDCl_3$)

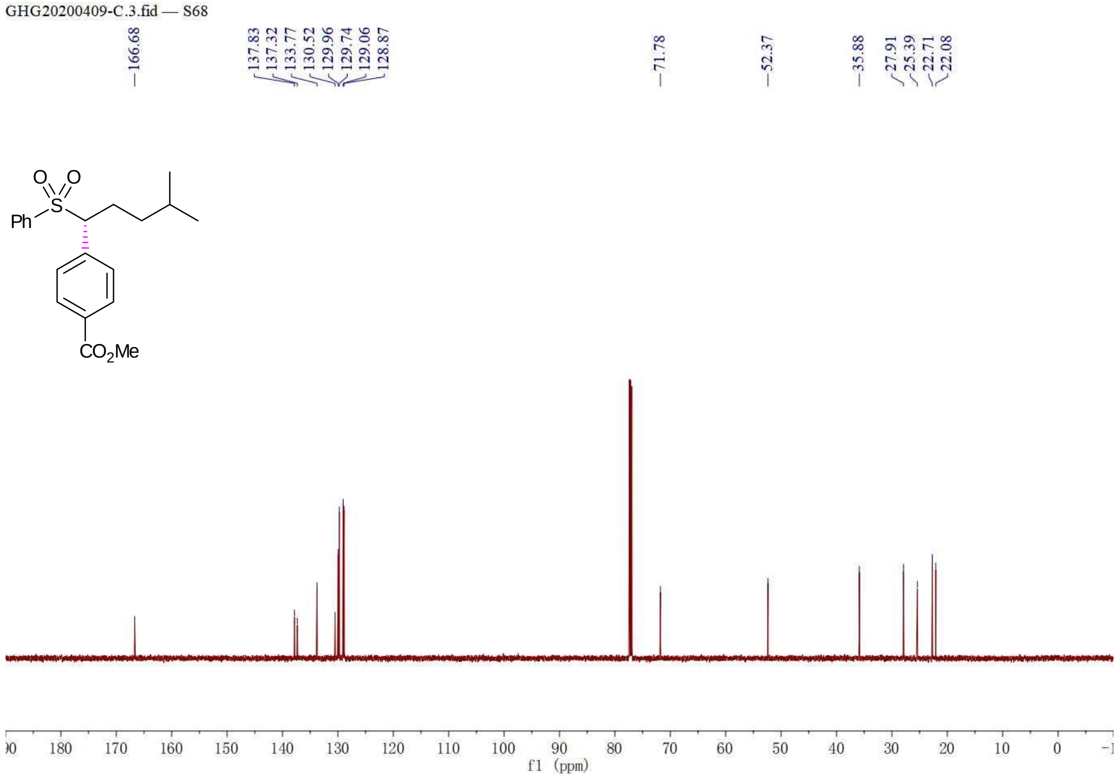

46, ^{1}H-NMR (600 MHZ, $CDCl_3$)

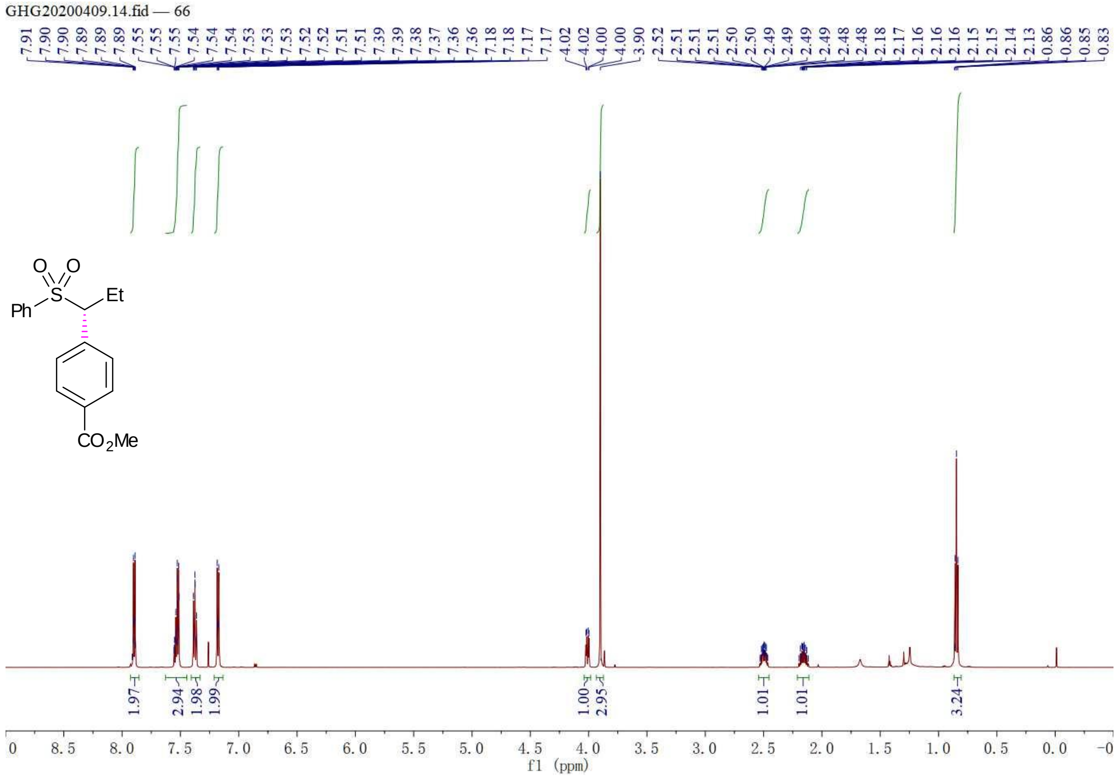

46, ^{13}C NMR (151 MHz, $CDCl_3$)

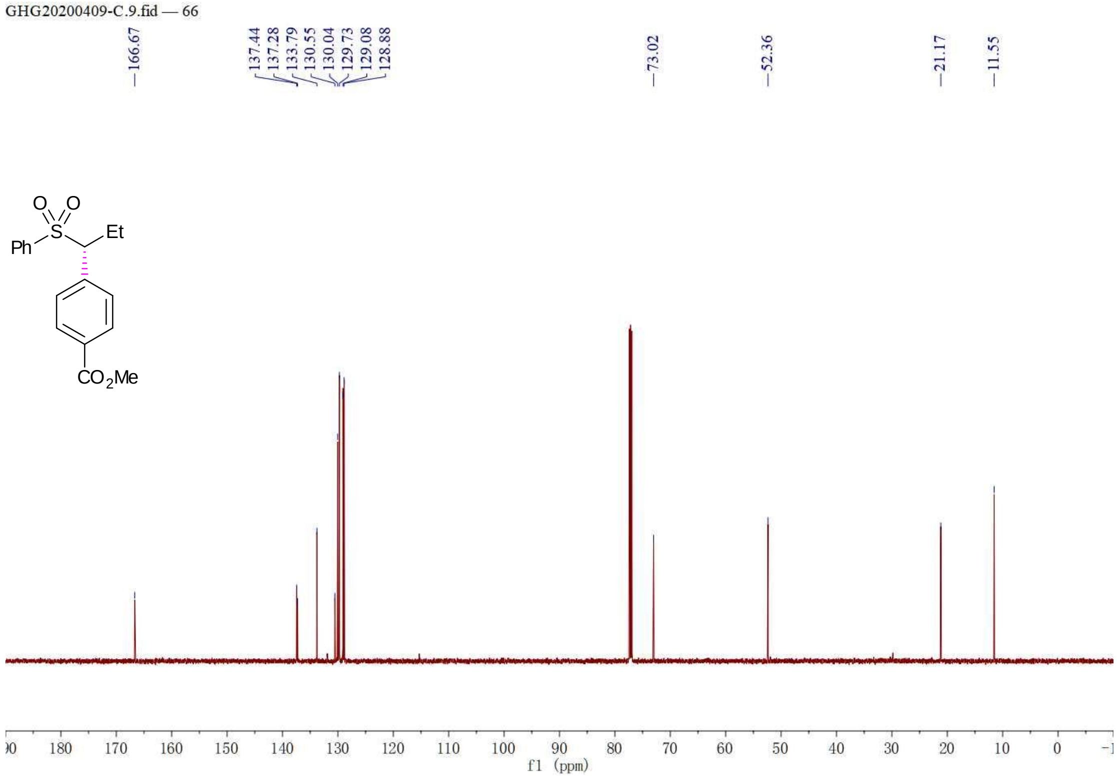

47, ^{1}H-NMR (600 MHZ, $CDCl_3$)

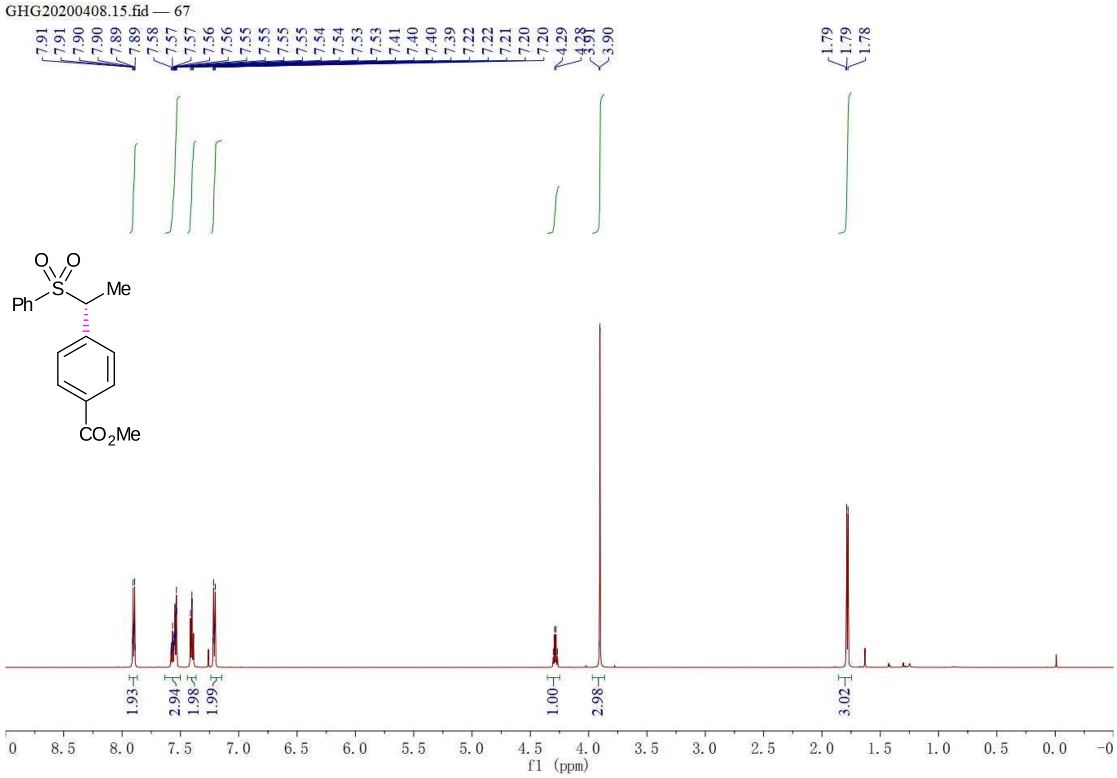

47, ^{13}C NMR (151 MHz, CDCl$_3$)

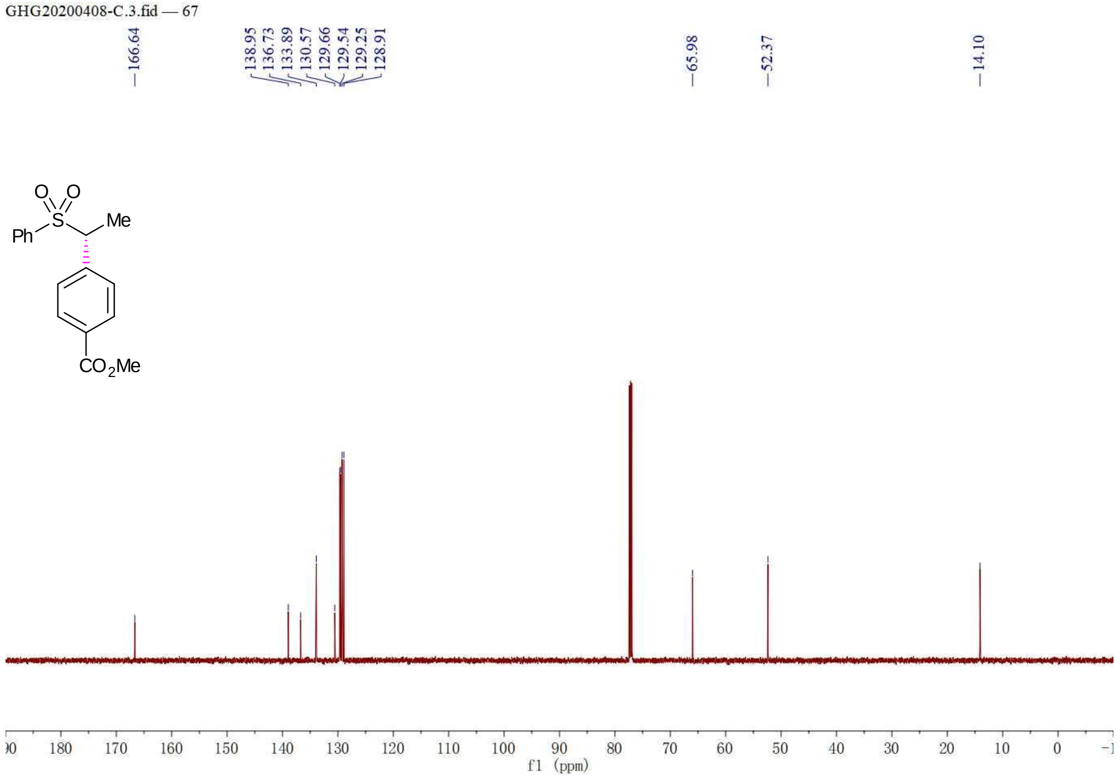

48, ^{1}H-NMR (600 MHZ, CDCl$_3$)

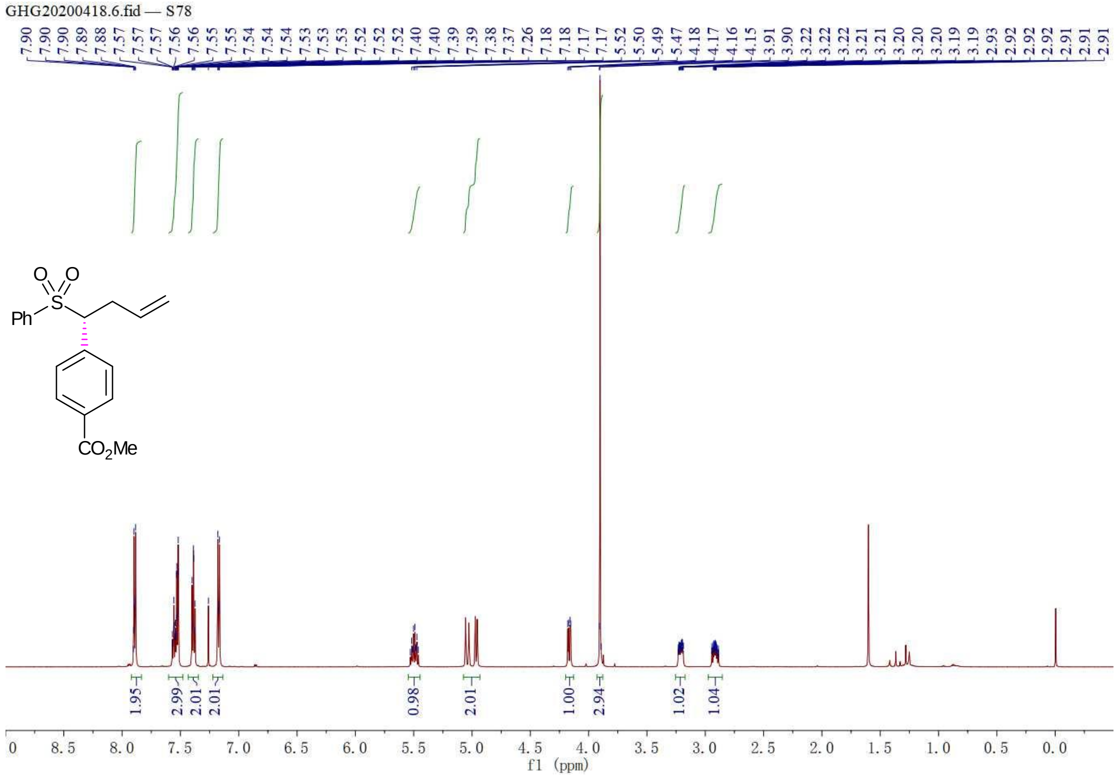

48, ^{13}C NMR (151 MHz, $CDCl_3$)

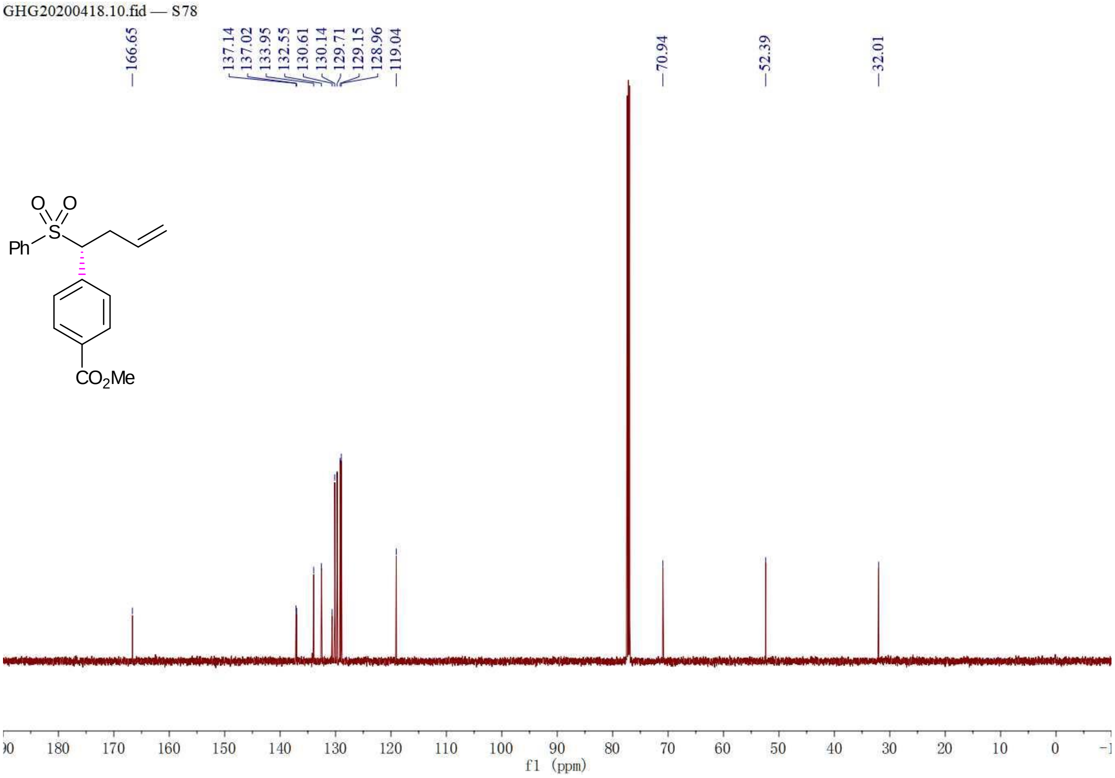

49, ^{1}H-NMR (600 MHZ, $CDCl_3$)

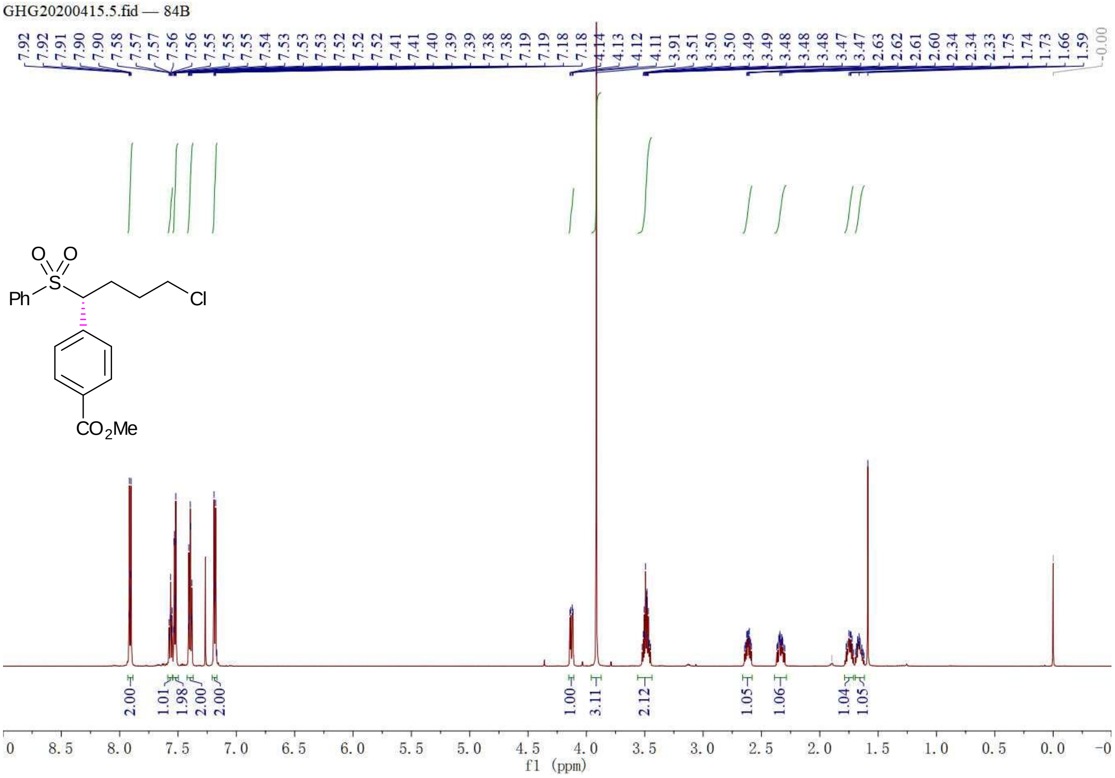

49, ^{13}C NMR (151 MHz, $CDCl_3$)

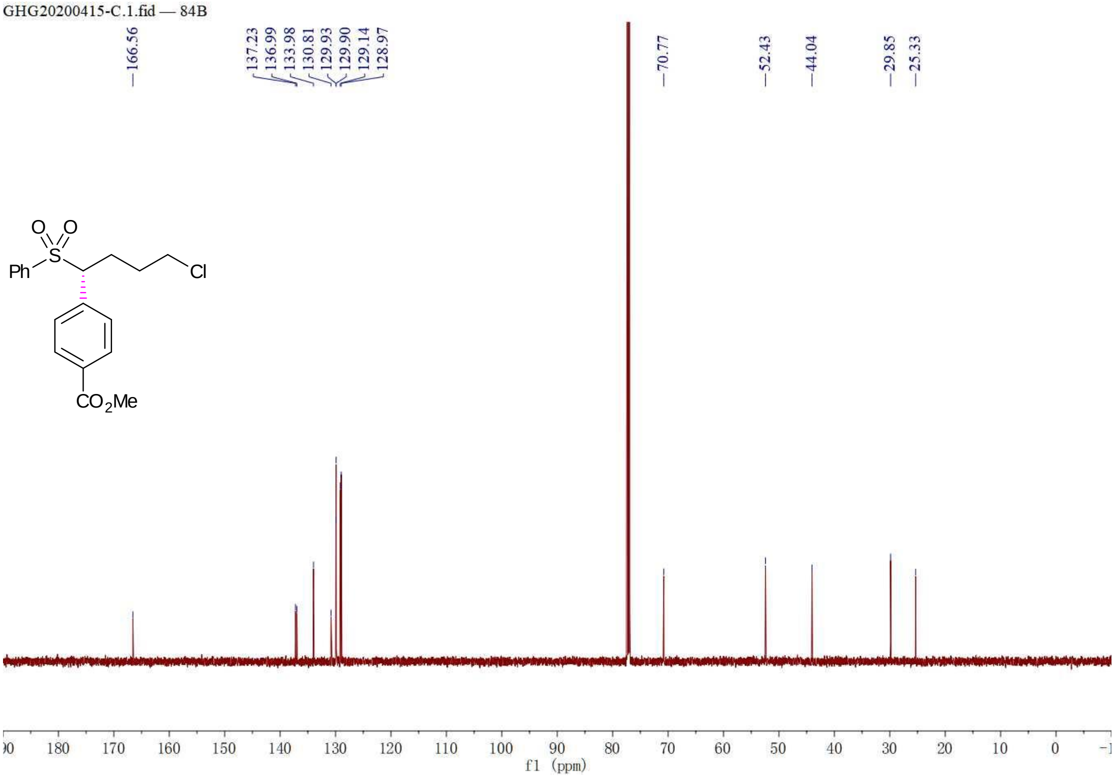

50, ^{1}H-NMR (600 MHZ, $CDCl_3$)

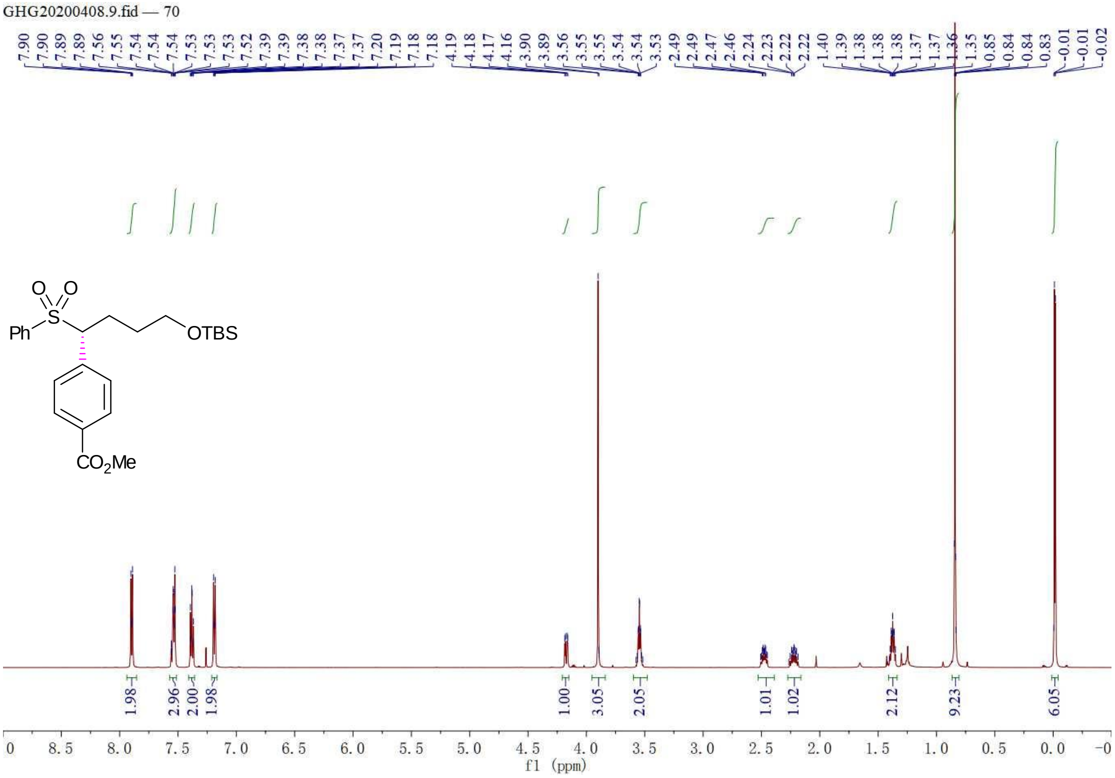

50, ^{13}C NMR (151 MHz, $CDCl_3$)

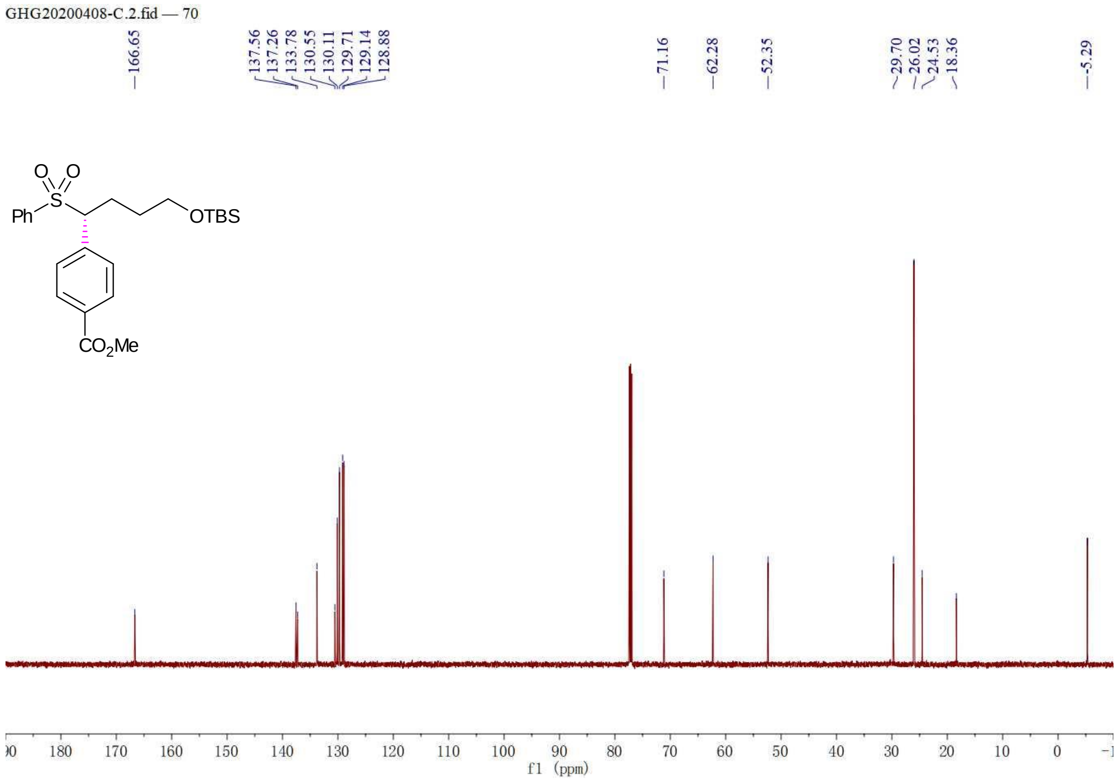

51, ^{1}H-NMR (600 MHZ, $CDCl_3$)

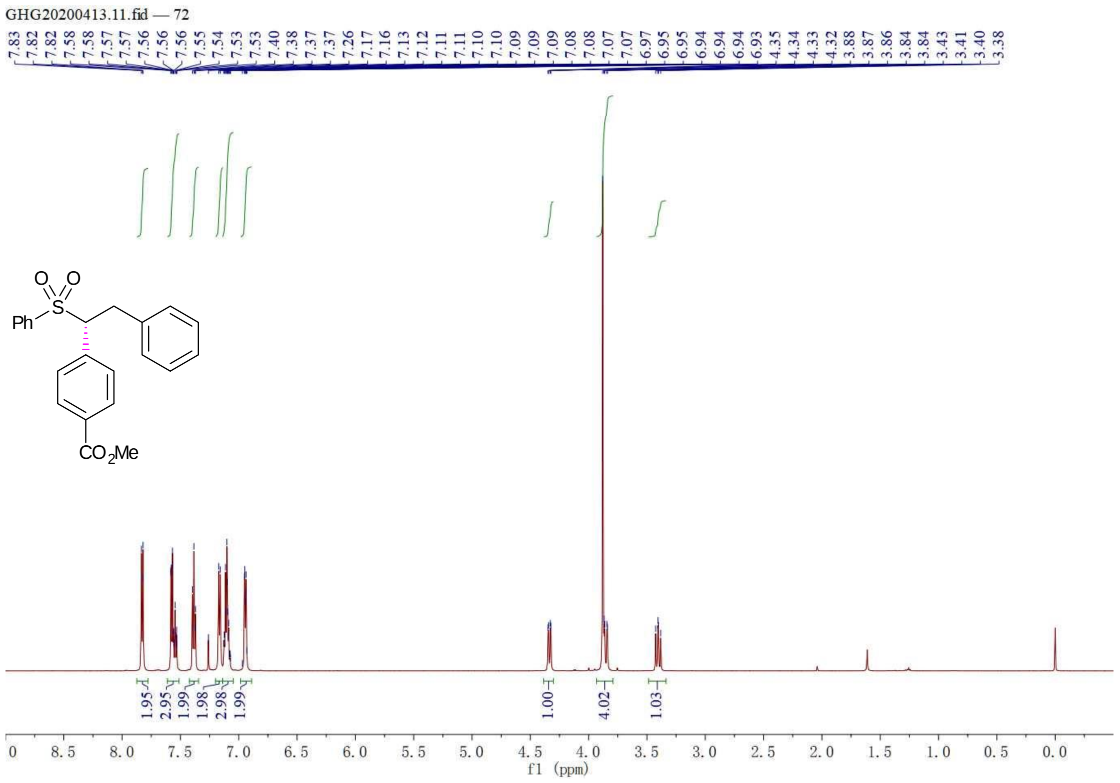

51, ^{13}C NMR (151 MHz, $CDCl_3$)

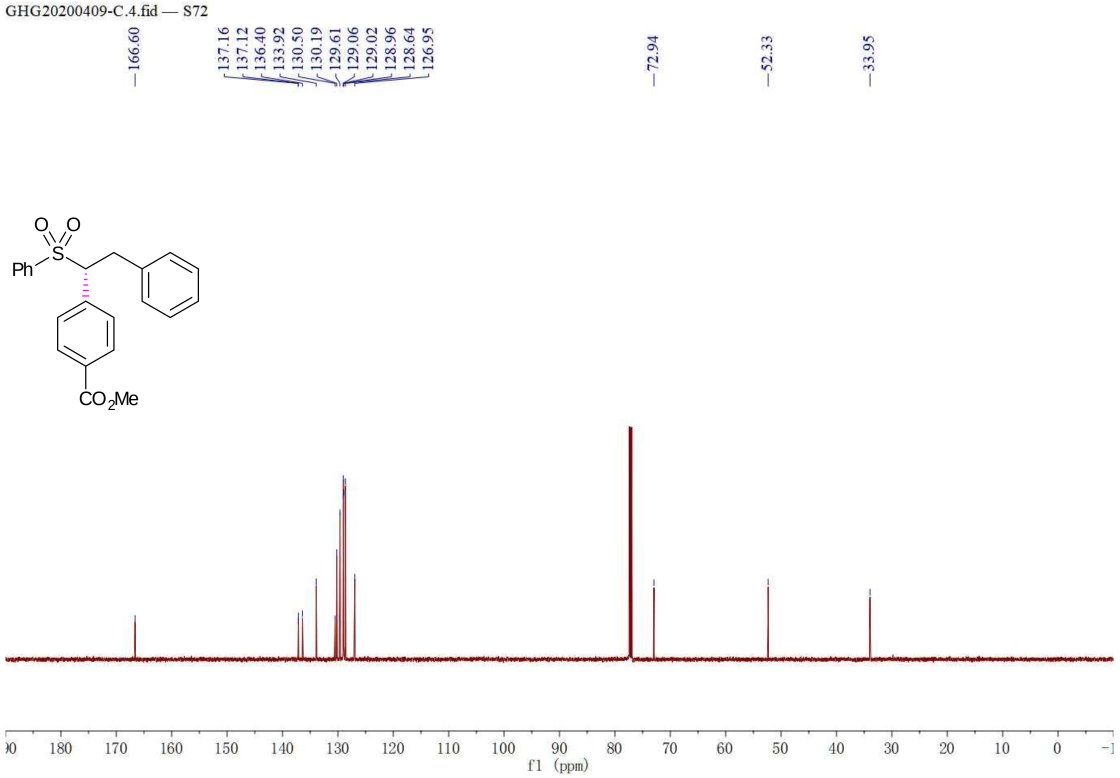

52, ^{1}H-NMR (600 MHZ, $CDCl_3$)

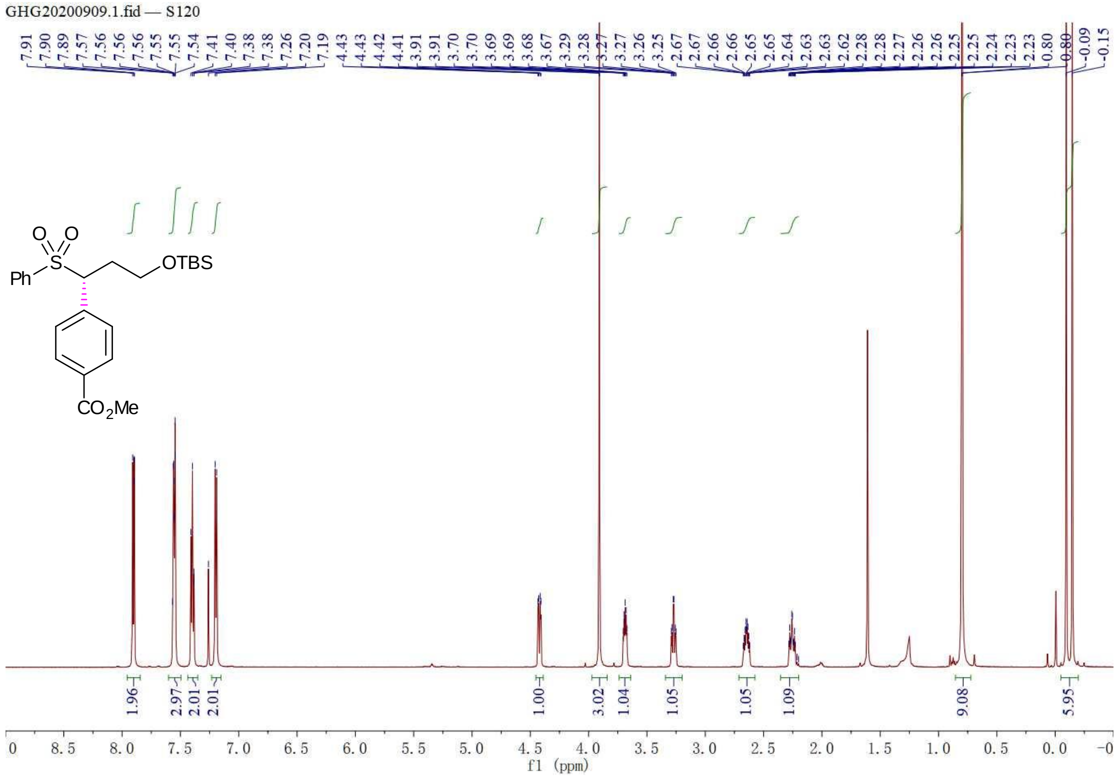

52, ^{13}C NMR (151 MHz, CDCl$_3$)

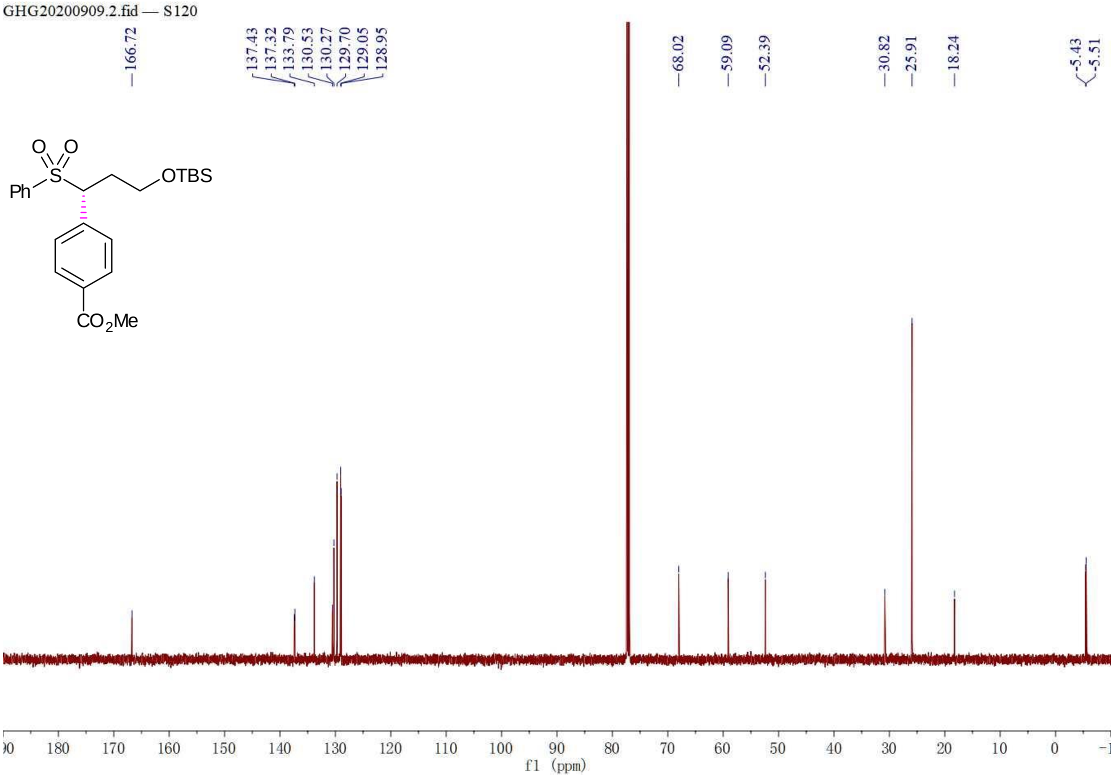

53, ^{1}H-NMR (600 MHZ, CDCl$_3$)

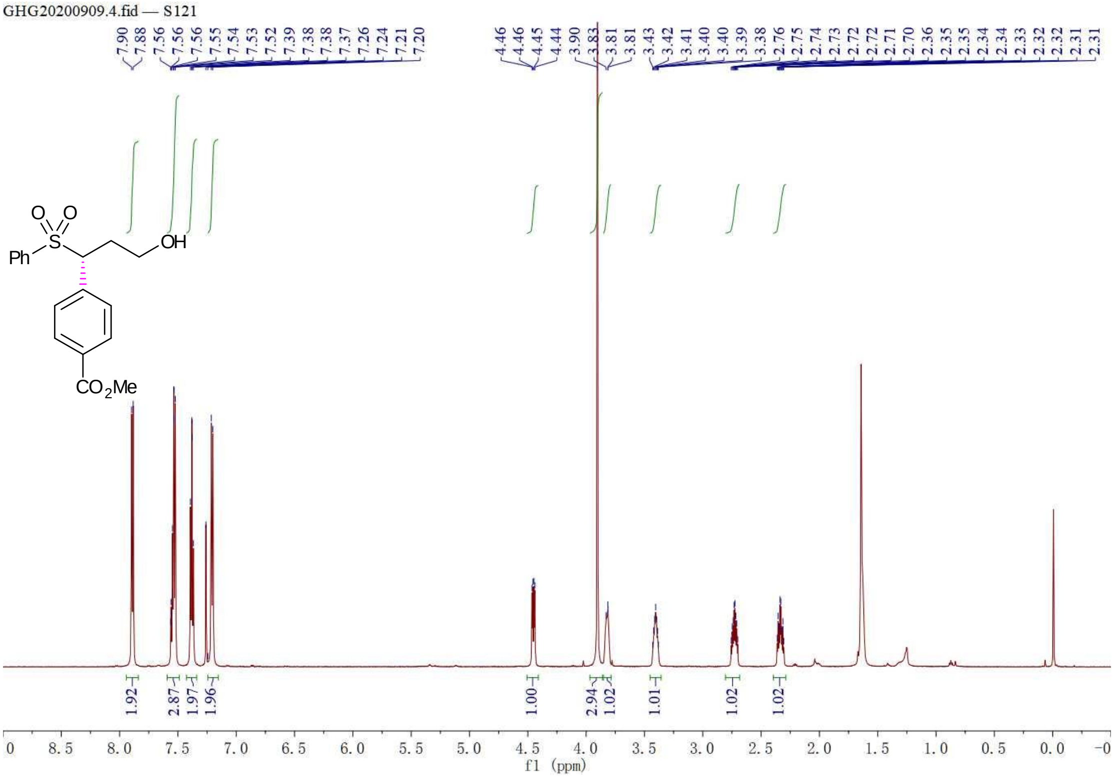

53, ^{13}C NMR (151 MHz, $CDCl_3$)

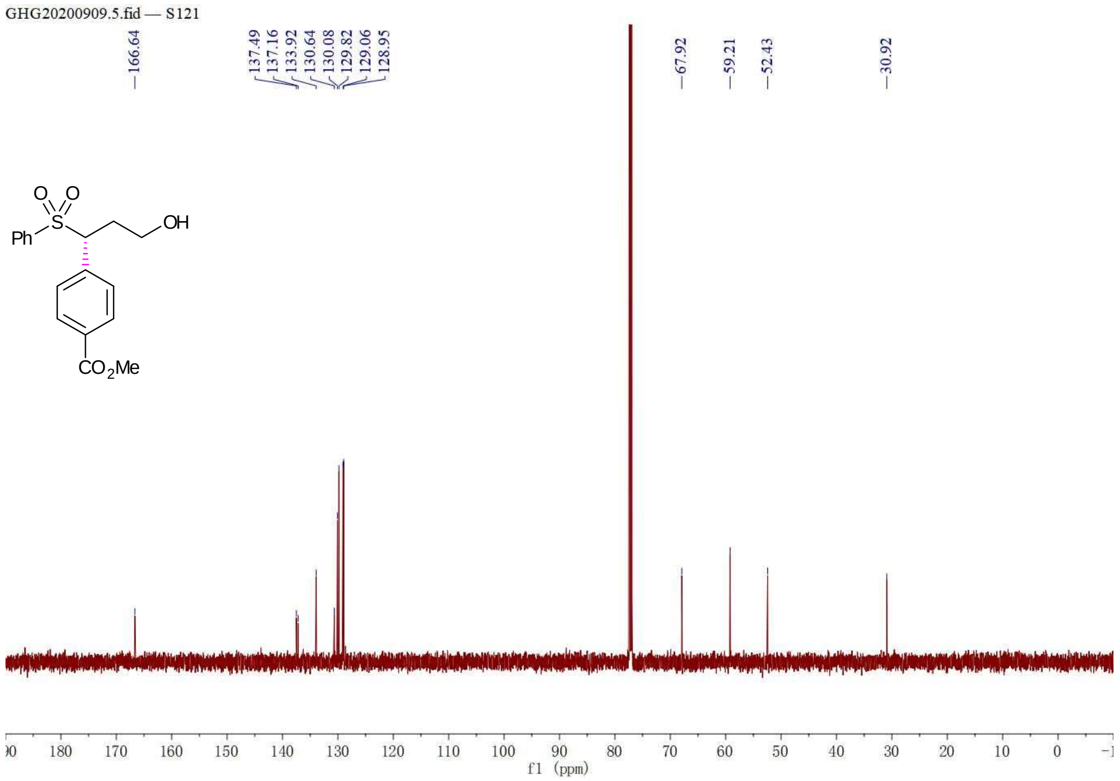

54, ^{1}H-NMR (600 MHZ, $CDCl_3$)

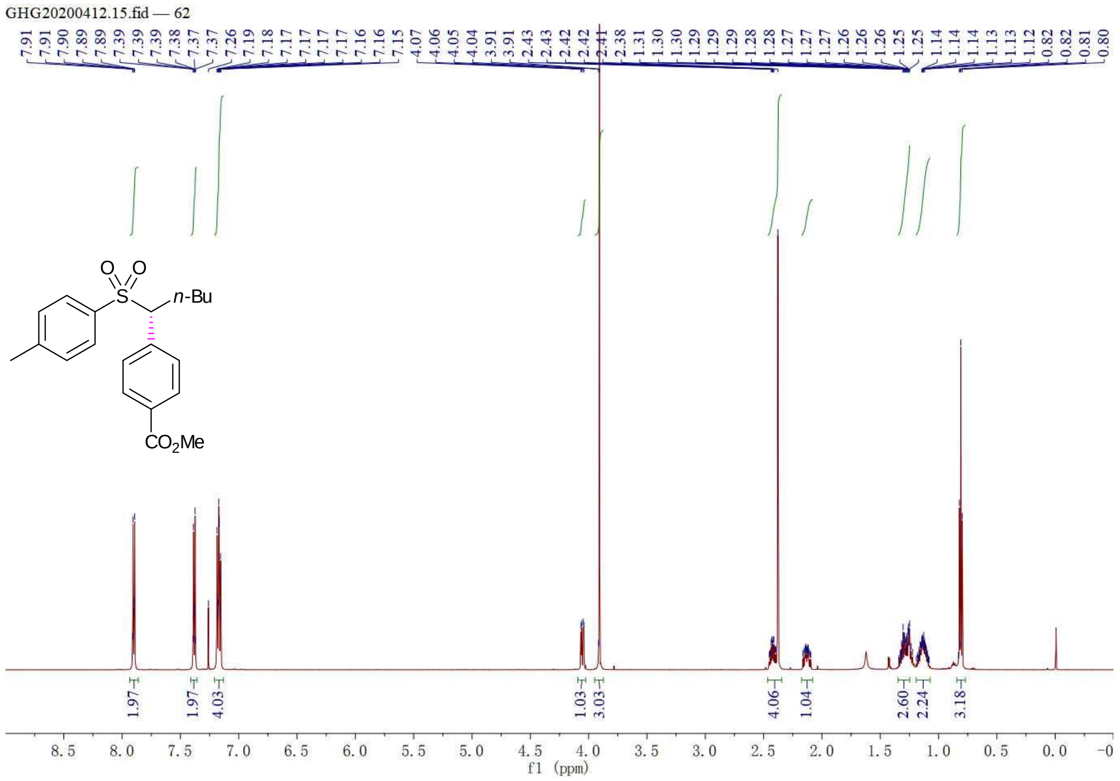

54, ^{13}C NMR (151 MHz, $CDCl_3$)

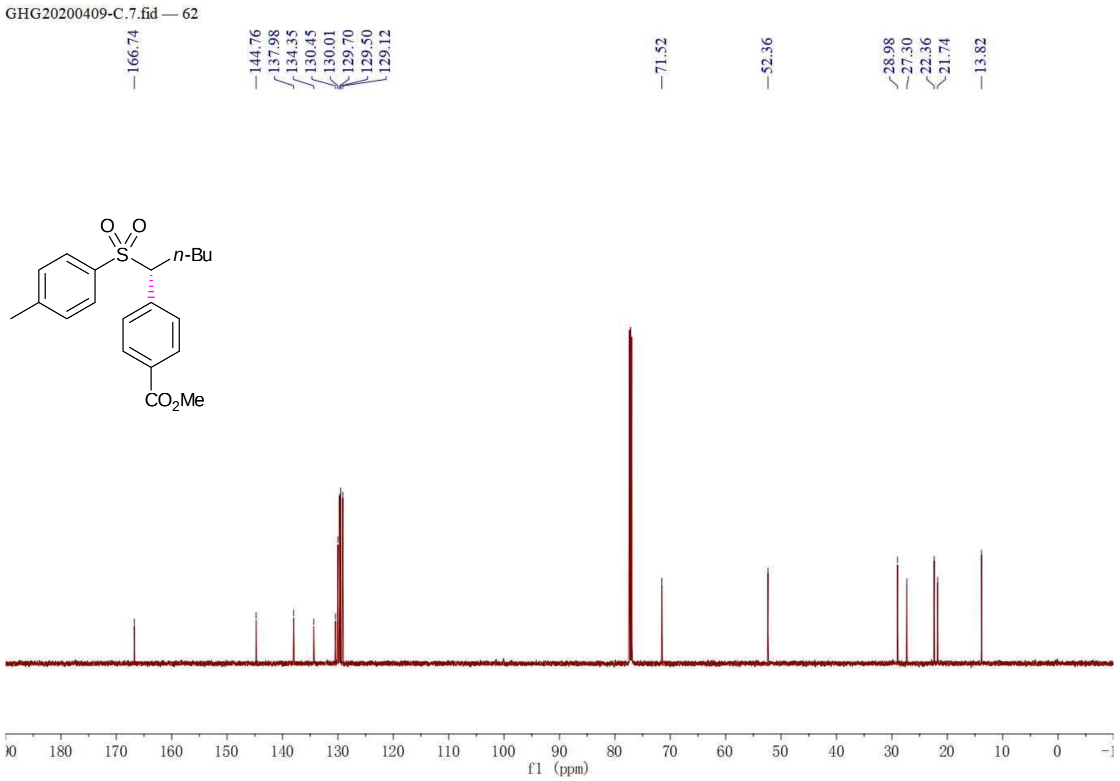

55, ^{1}H-NMR (600 MHZ, $CDCl_3$)

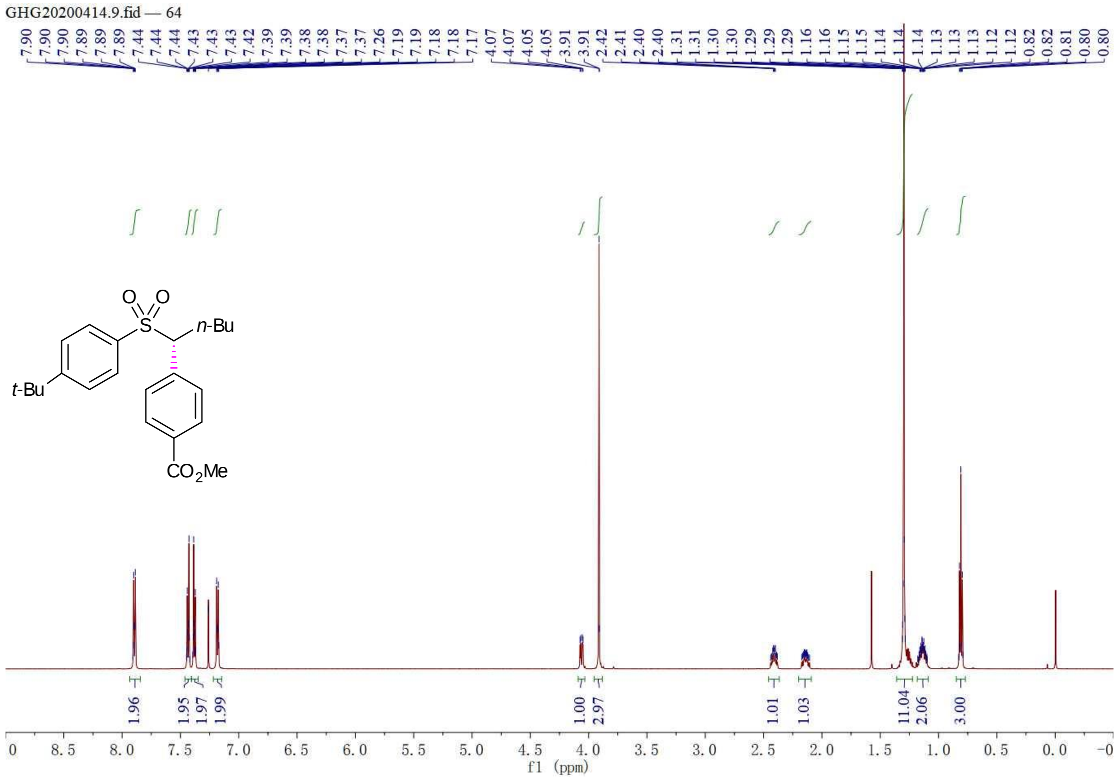

55, ^{13}C NMR (151 MHz, $CDCl_3$)

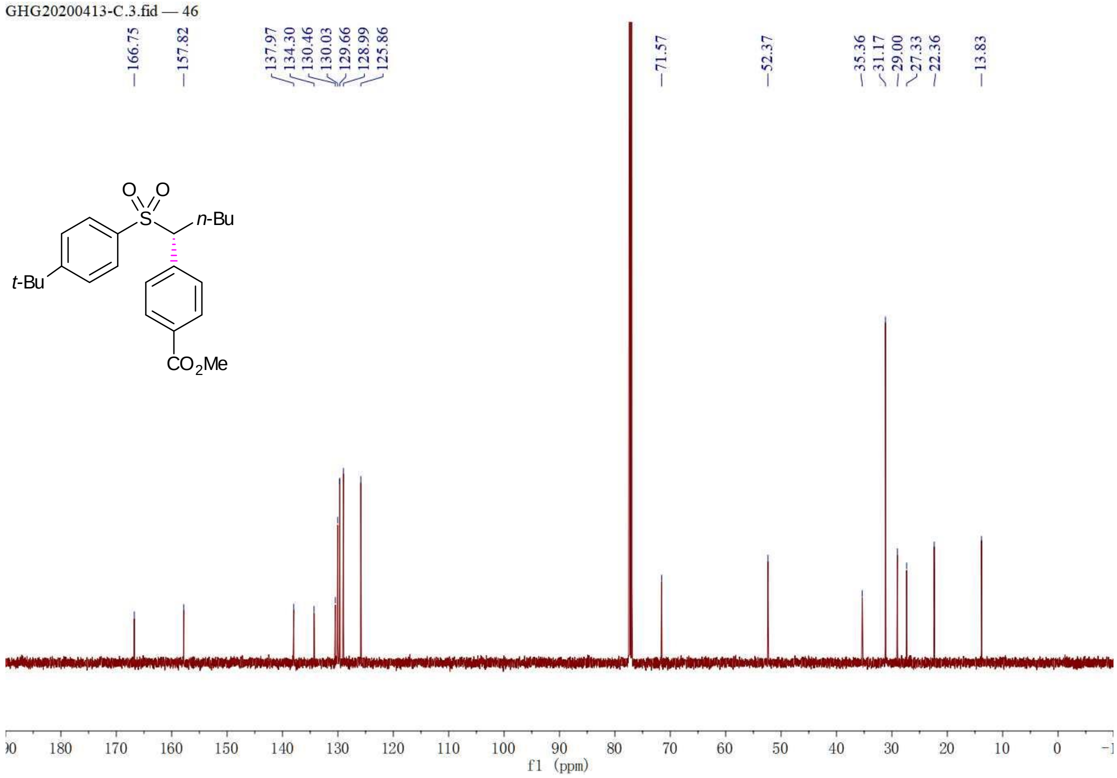

56, ^{1}H-NMR (600 MHZ, $CDCl_3$)

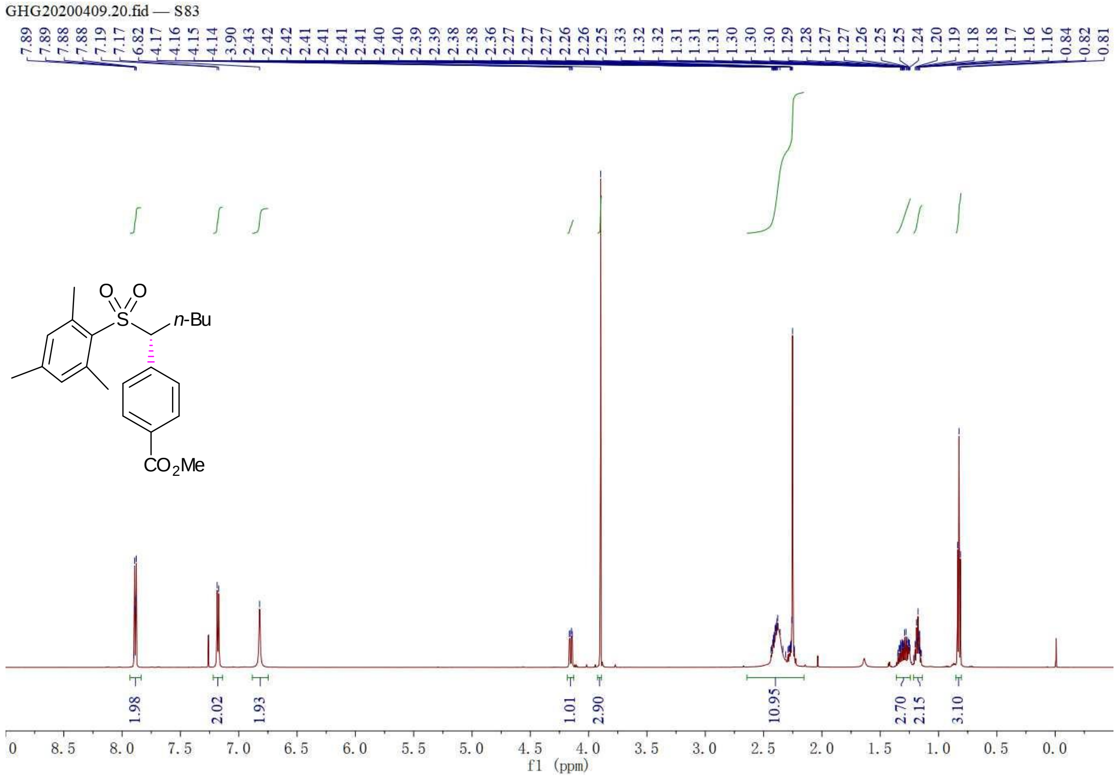

56, ^{13}C NMR (151 MHz, $CDCl_3$)

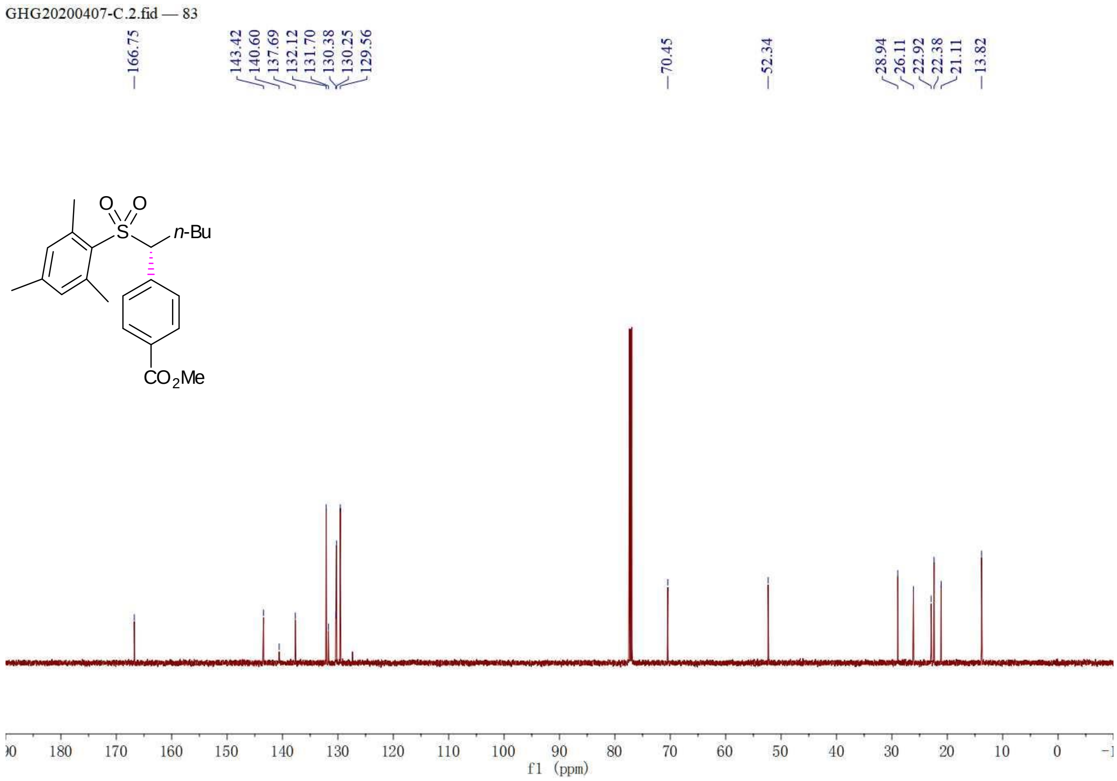

57, ^{1}H-NMR (600 MHZ, $CDCl_3$)

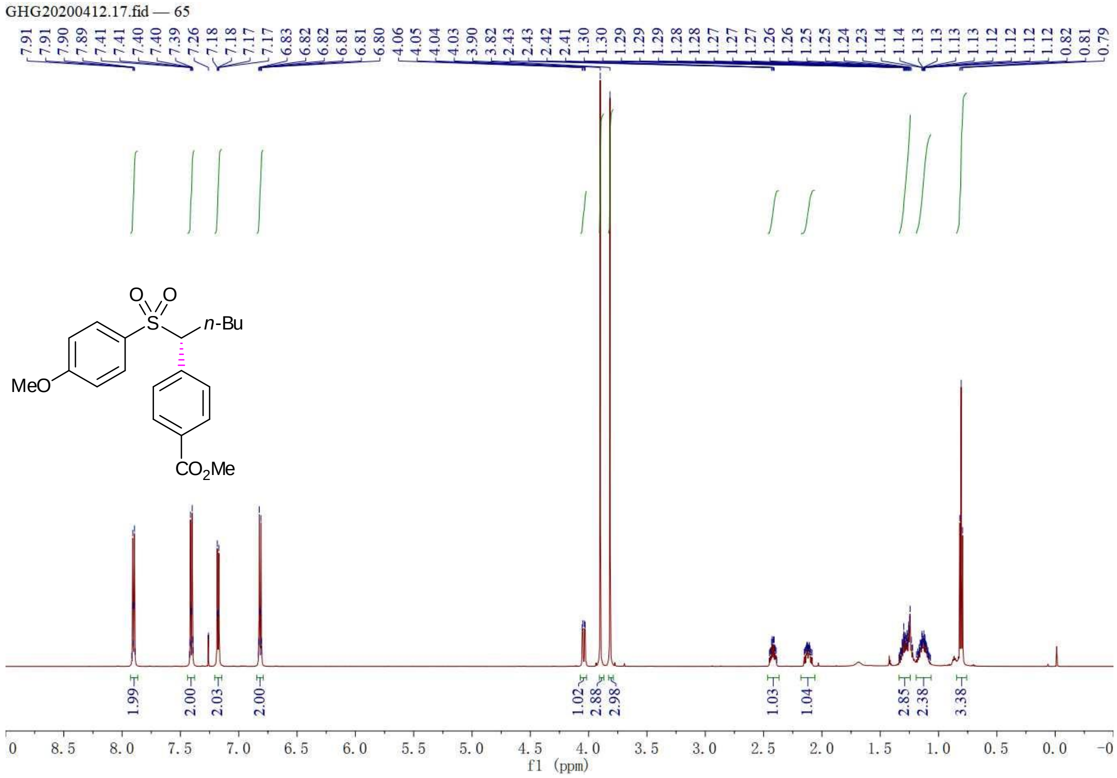

57, ^{13}C NMR (151 MHz, $CDCl_3$)

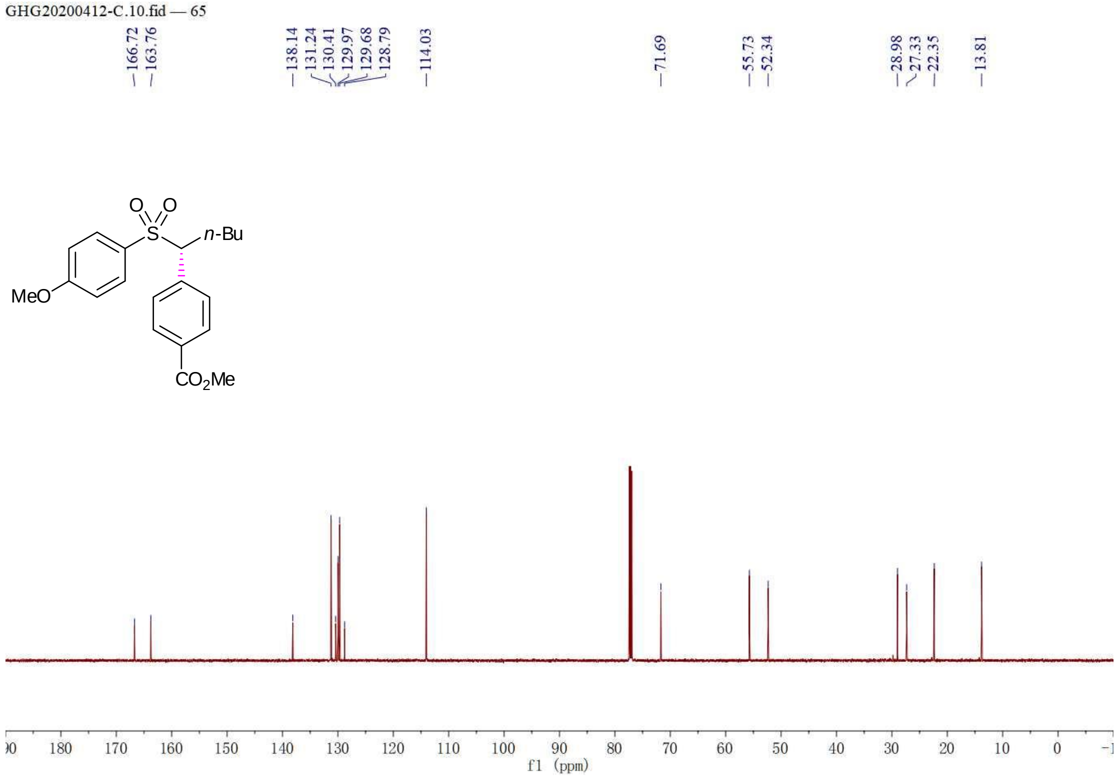

58, ^{1}H-NMR (600 MHZ, $CDCl_3$)

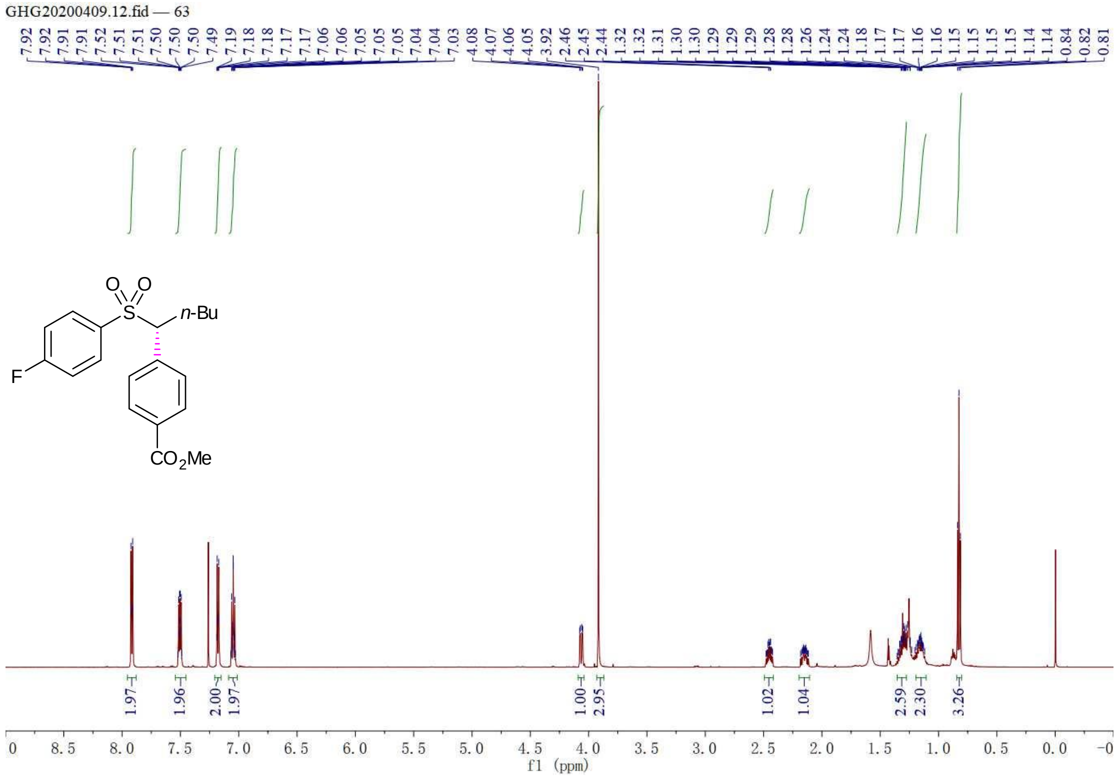

58, ^{13}C NMR (151 MHz, CDCl$_3$)

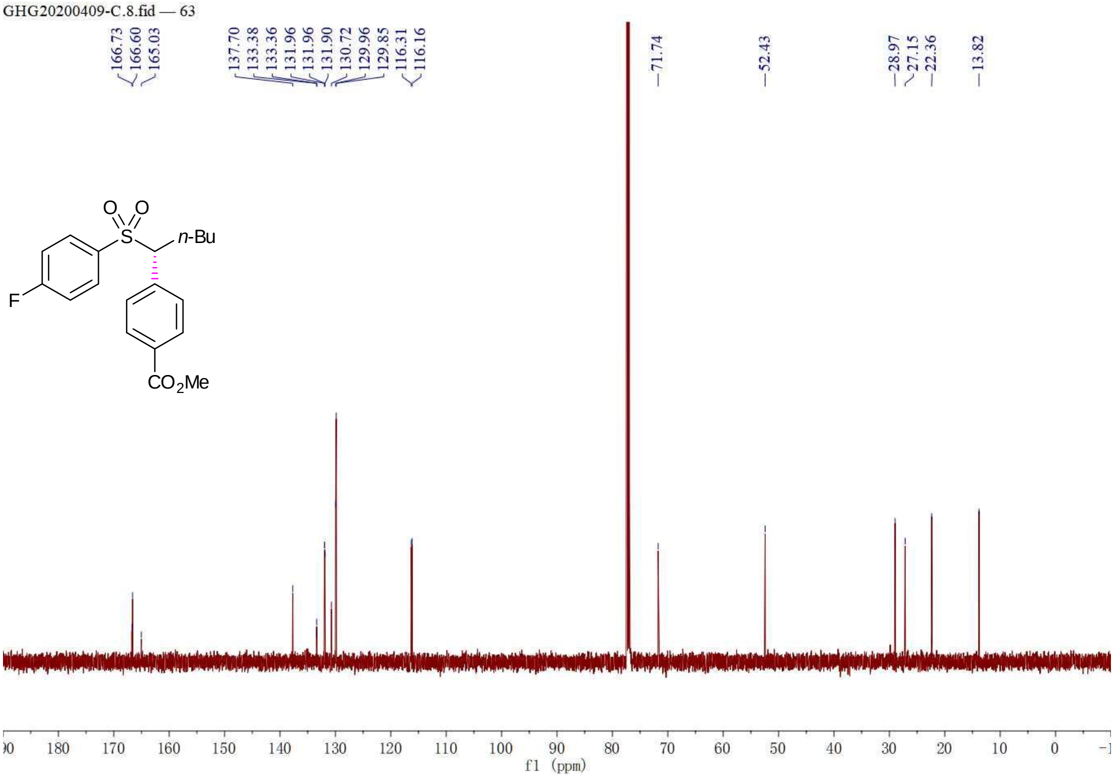

58, ^{19}F NMR (565 MHz, CDCl$_3$)

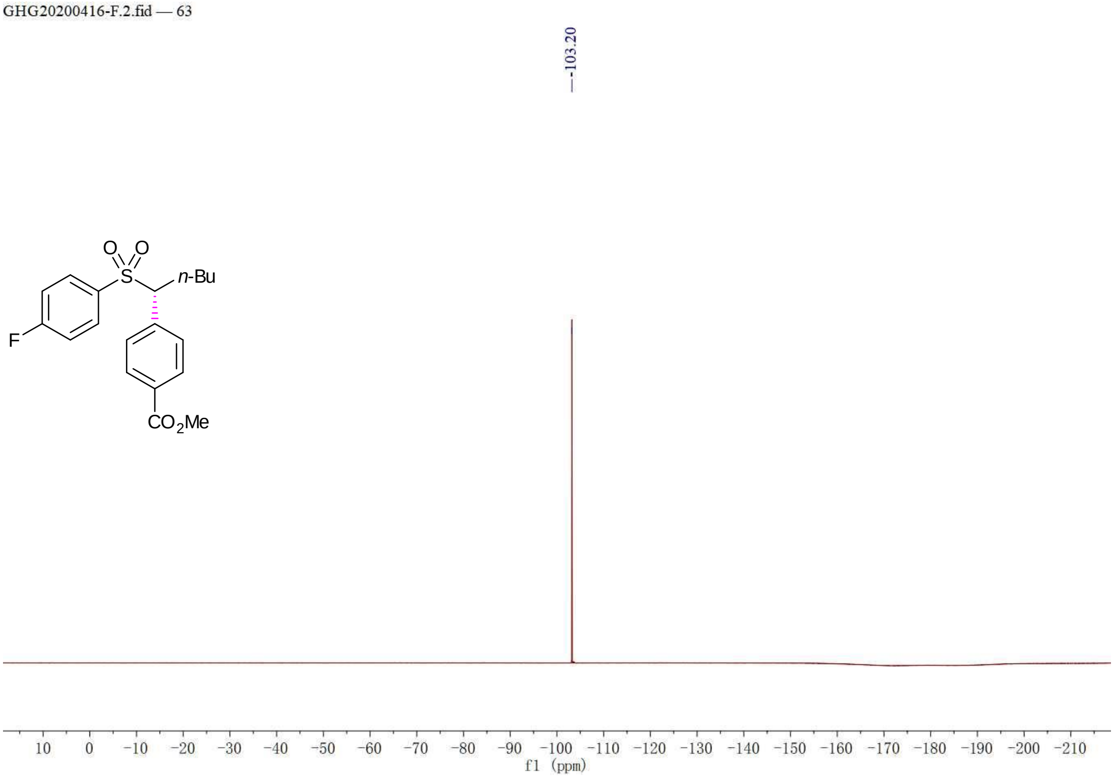

59, ^{1}H-NMR (600 MHZ, CDCl$_3$)

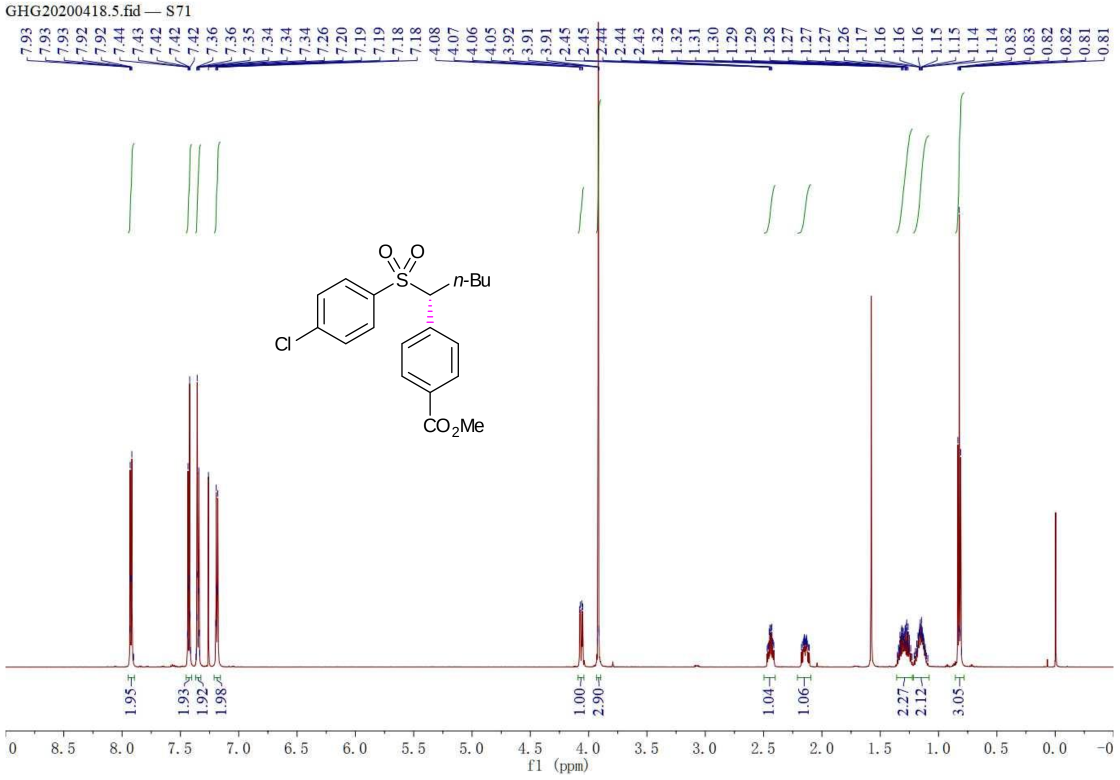

59, ^{13}C NMR (151 MHz, CDCl$_3$)

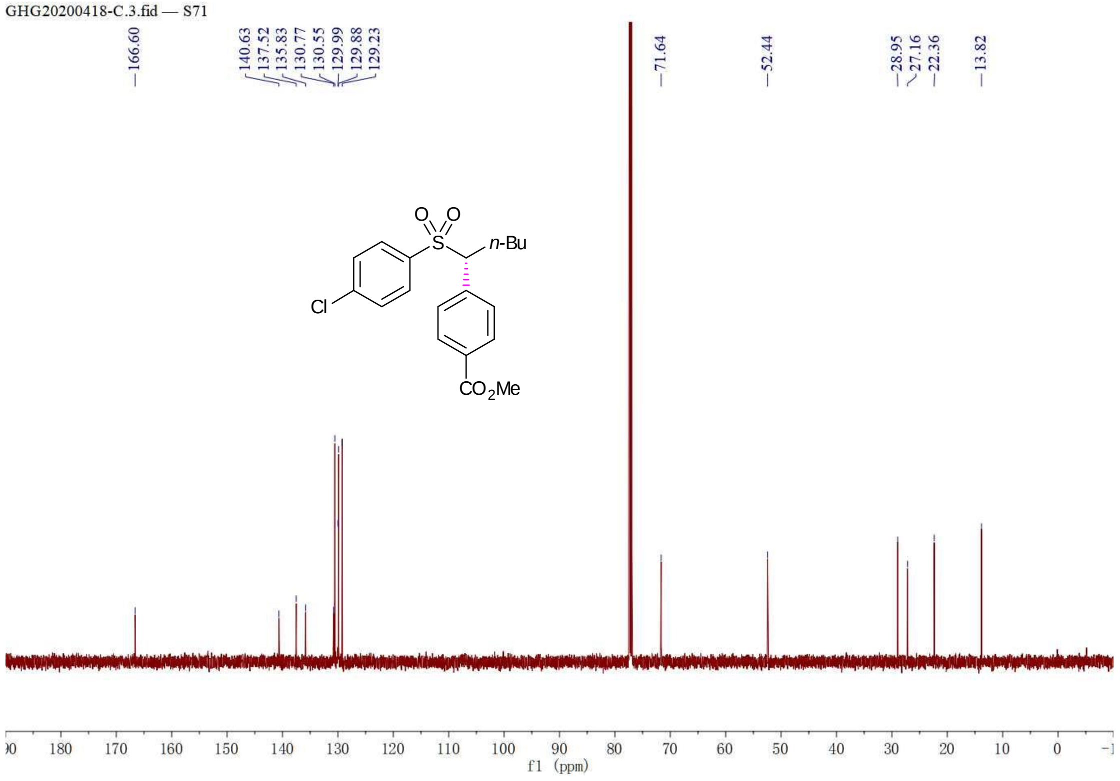

60, ^{1}H-NMR (600 MHZ, CDCl$_3$)

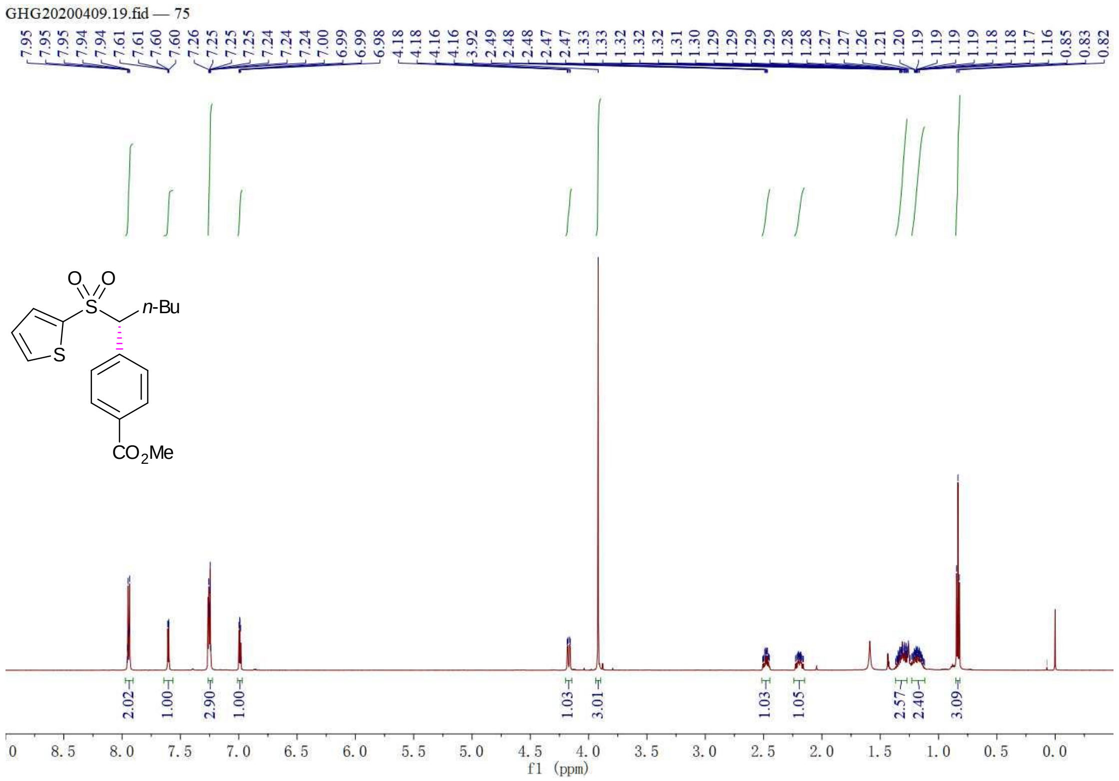

60, ^{13}C NMR (151 MHz, CDCl$_3$)

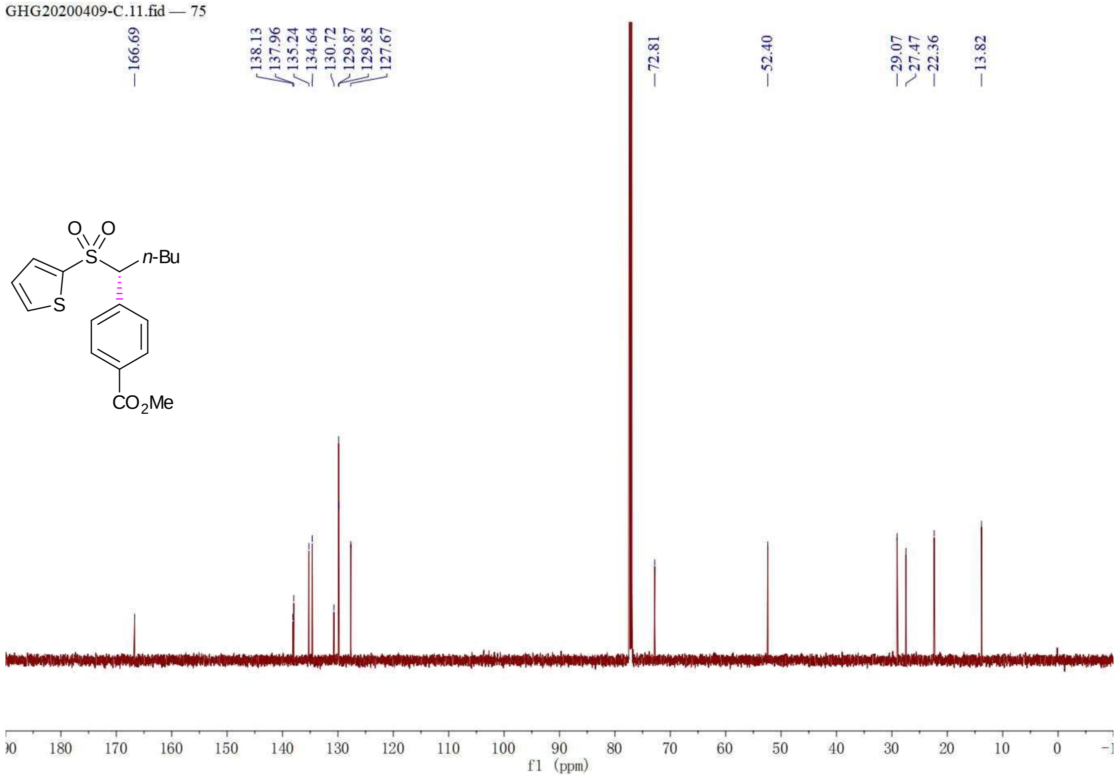

61, ^{1}H-NMR (600 MHZ, CDCl$_3$)

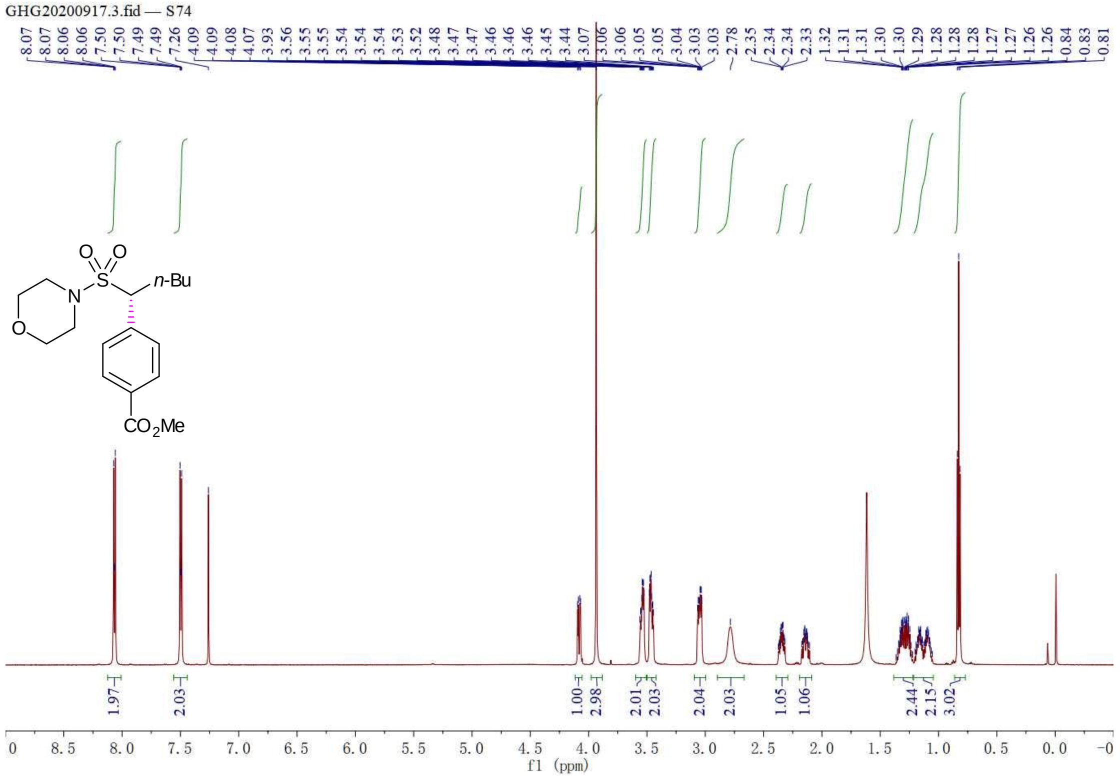

61, ^{13}C NMR (151 MHz, CDCl$_3$)

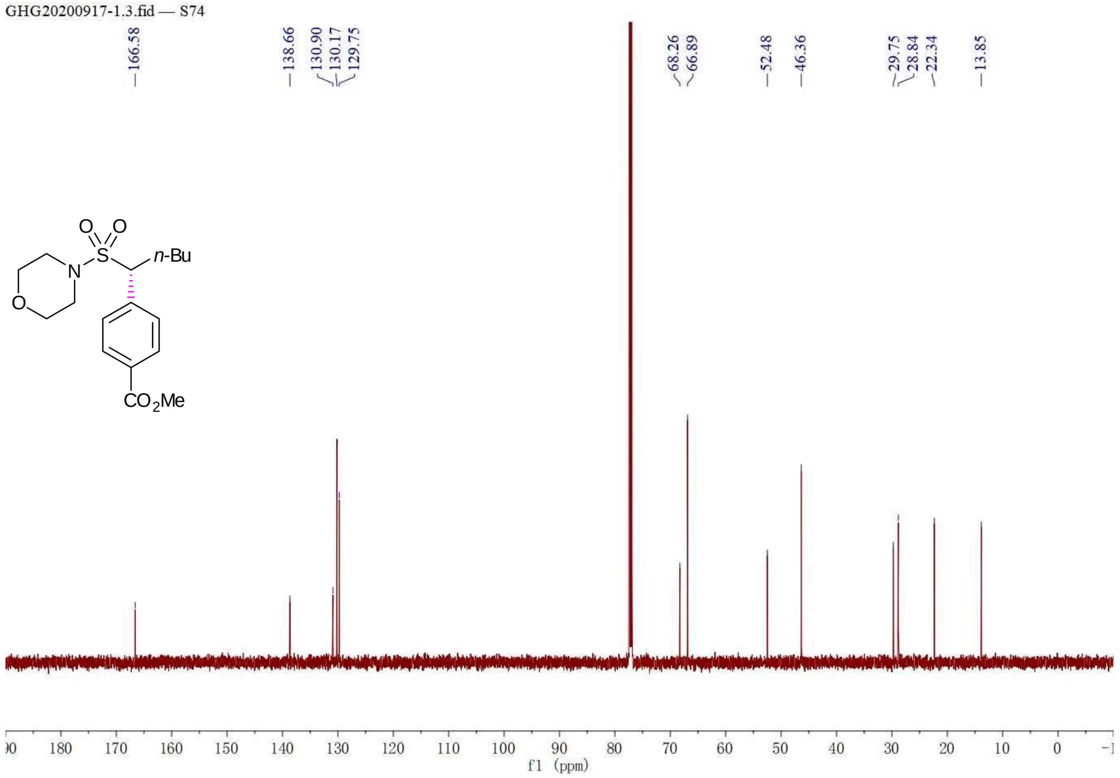

62, ^{1}H-NMR (600 MHZ, CDCl$_3$)

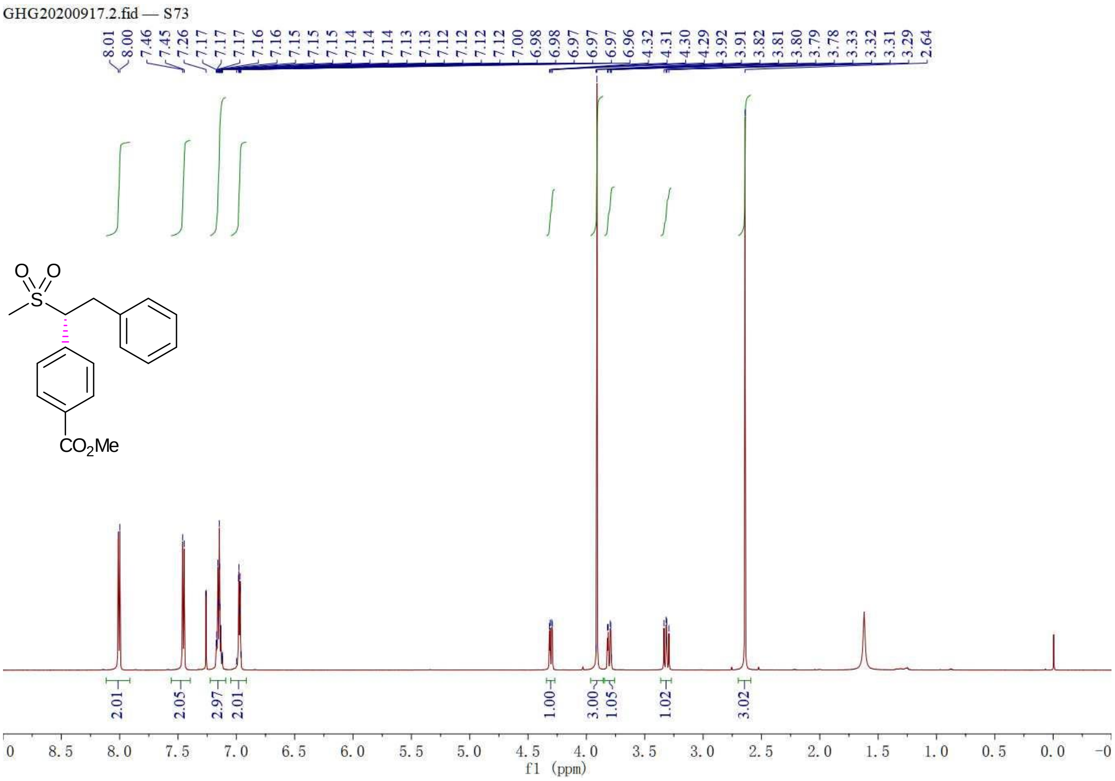

62, ^{13}C NMR (151 MHz, CDCl$_3$)

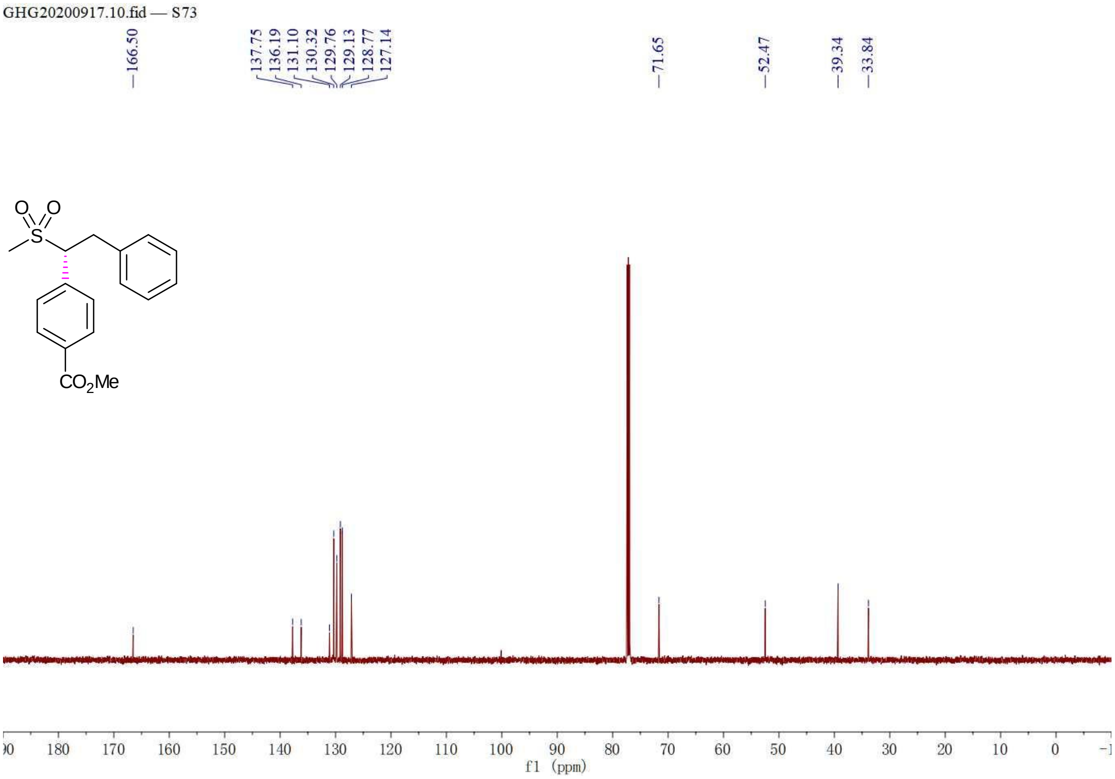

63, ^{1}H-NMR (600 MHZ, CDCl$_3$)

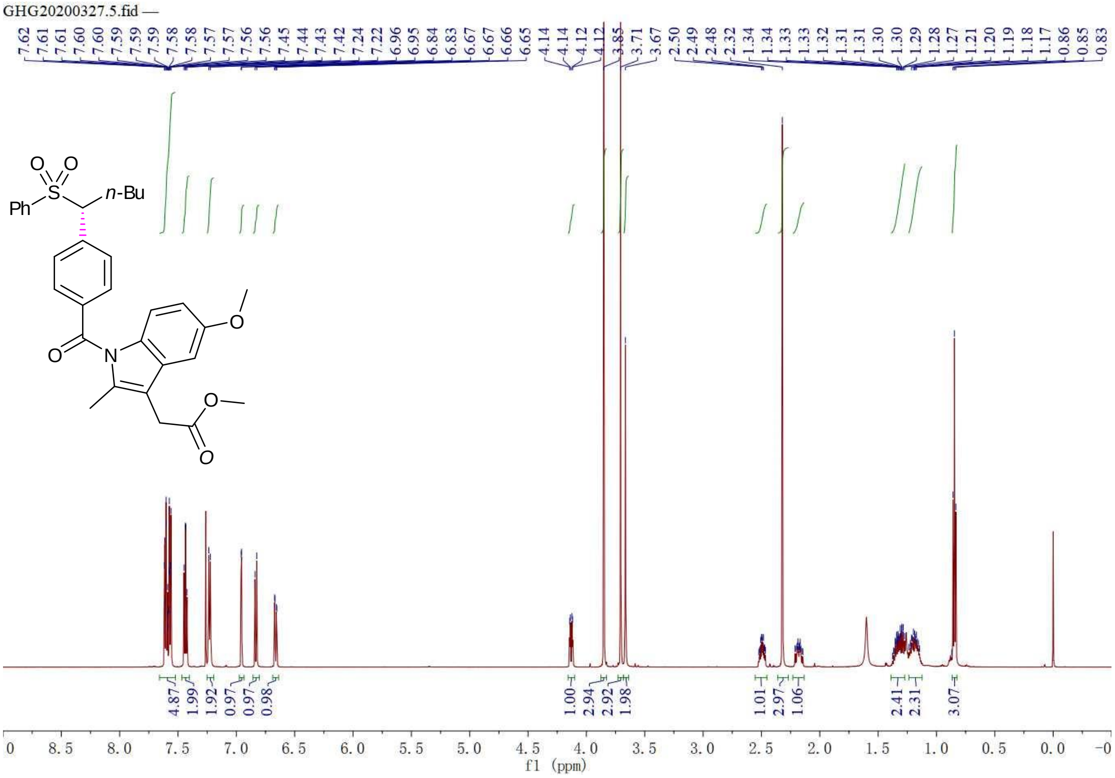

63, ^{13}C NMR (151 MHz, CDCl$_3$)

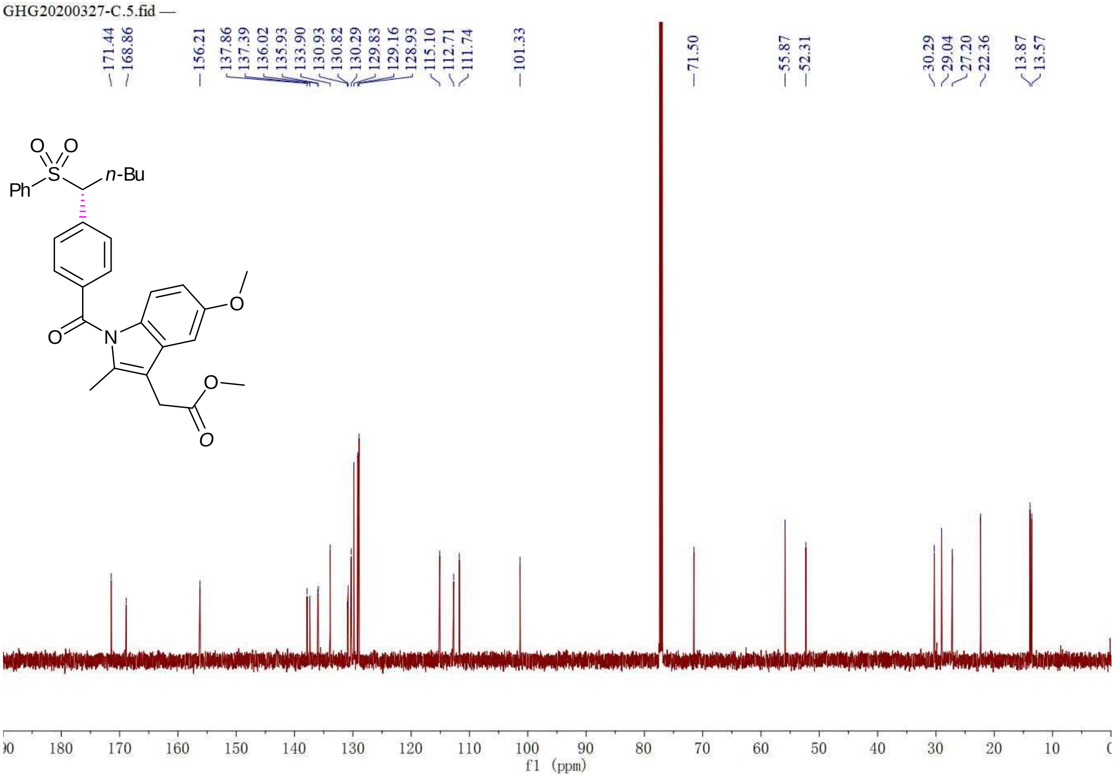

64, ^{1}H-NMR (600 MHZ, $CDCl_3$)

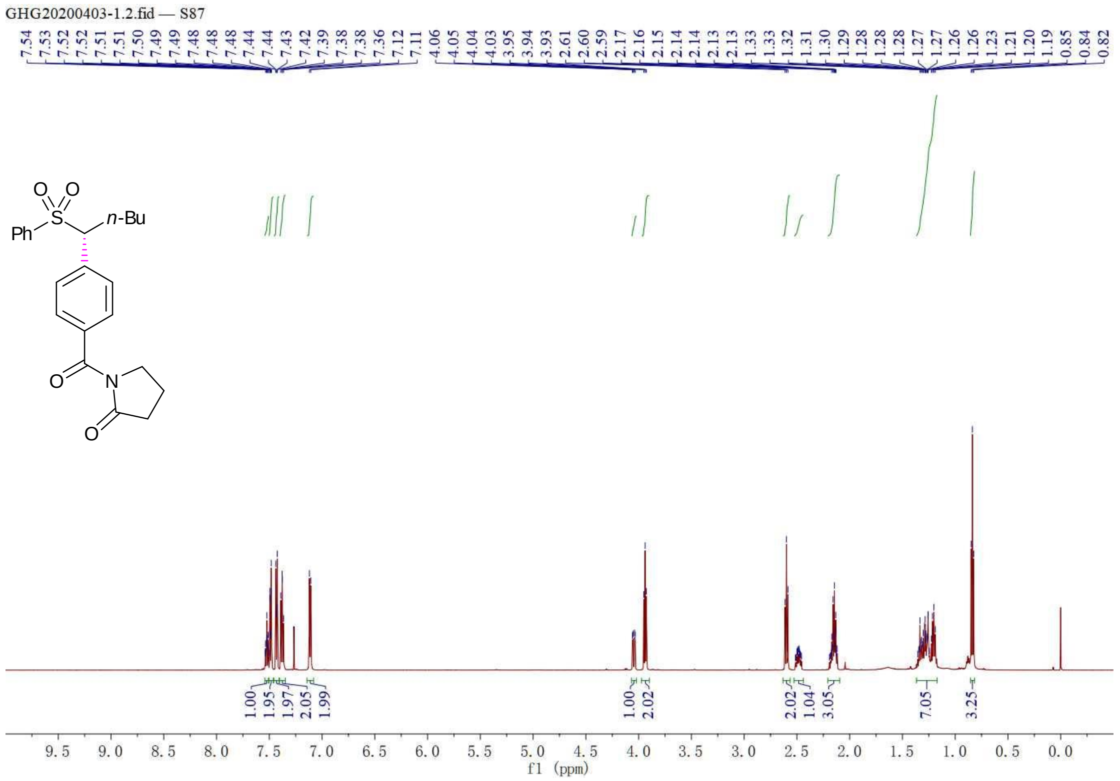

64, ^{13}C NMR (151 MHz, $CDCl_3$)

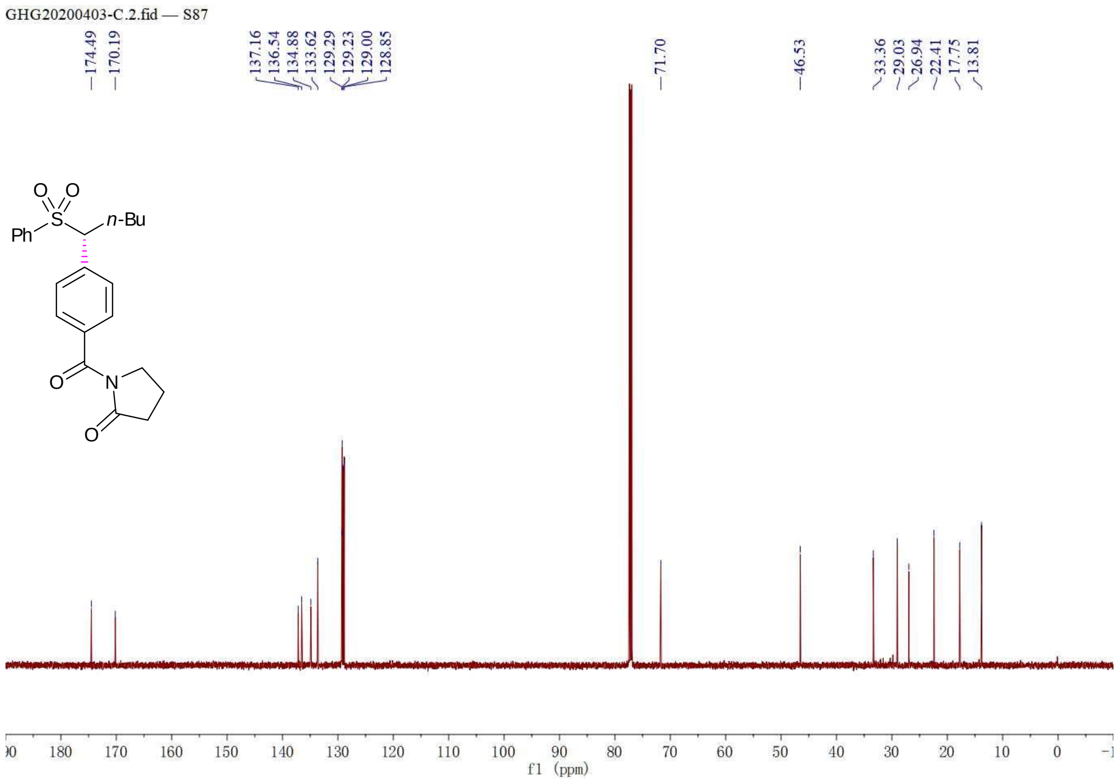

65, ^{1}H-NMR (600 MHZ, CDCl3)

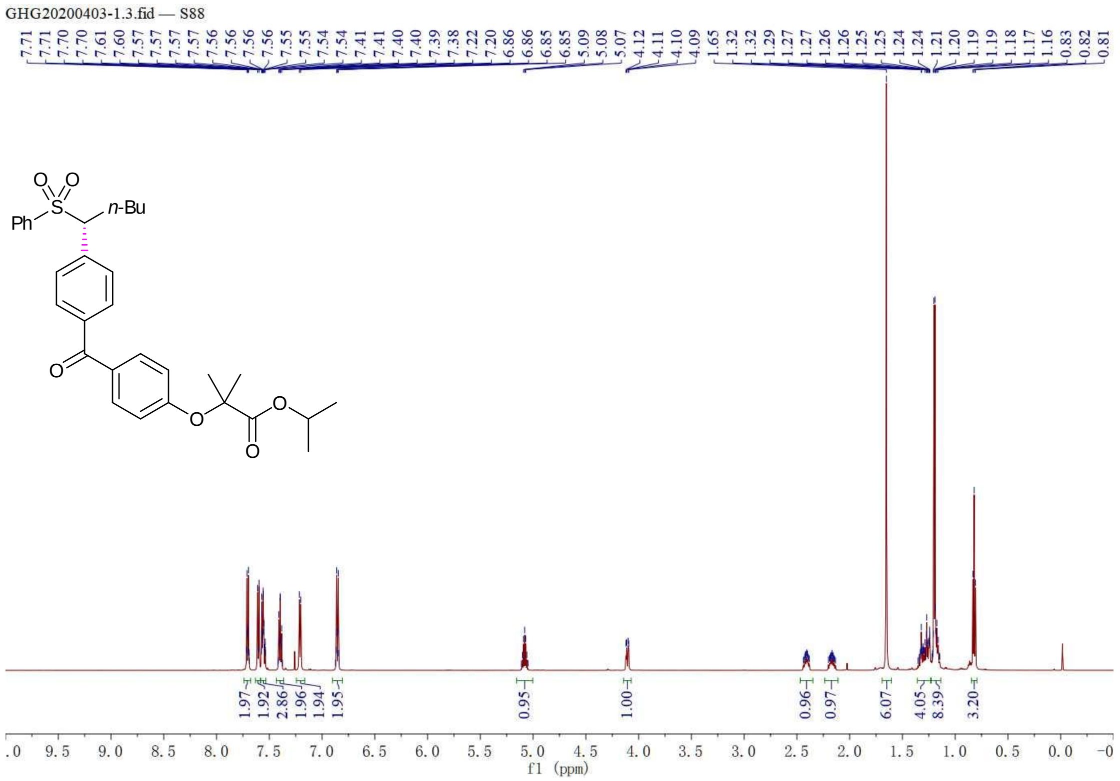

65, ^{13}C NMR (151 MHz, CDCl3)

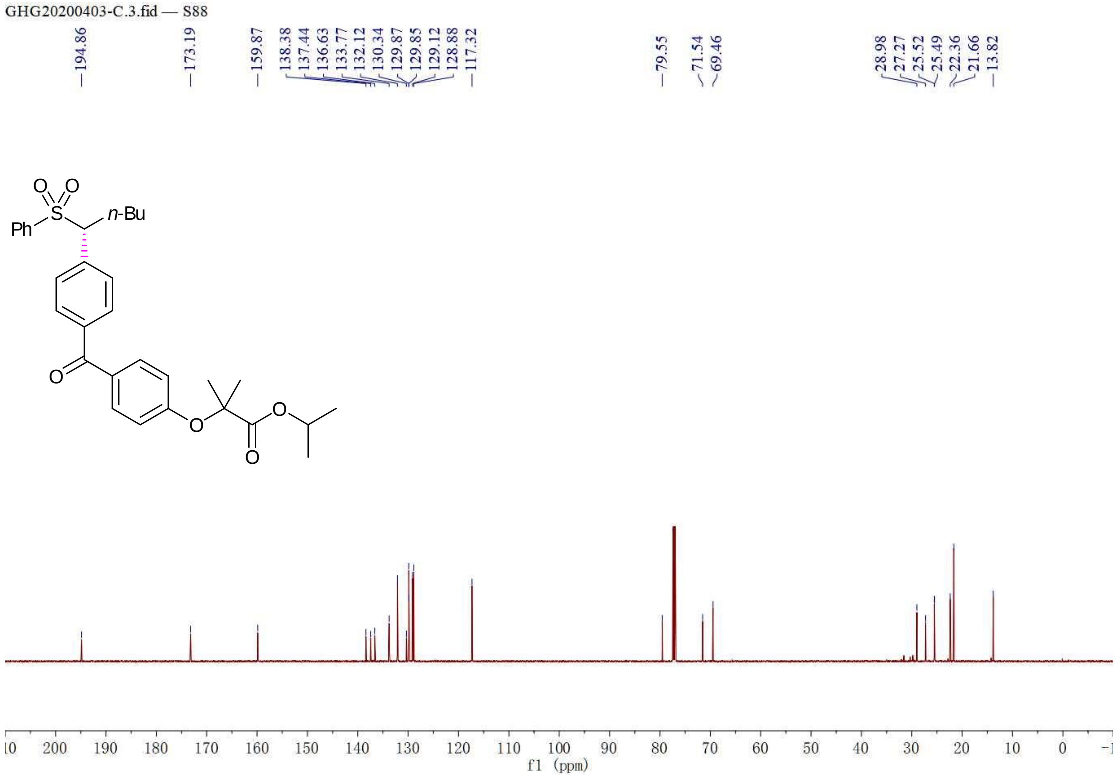

66, ^{1}H-NMR (600 MHZ, CDCl$_3$)

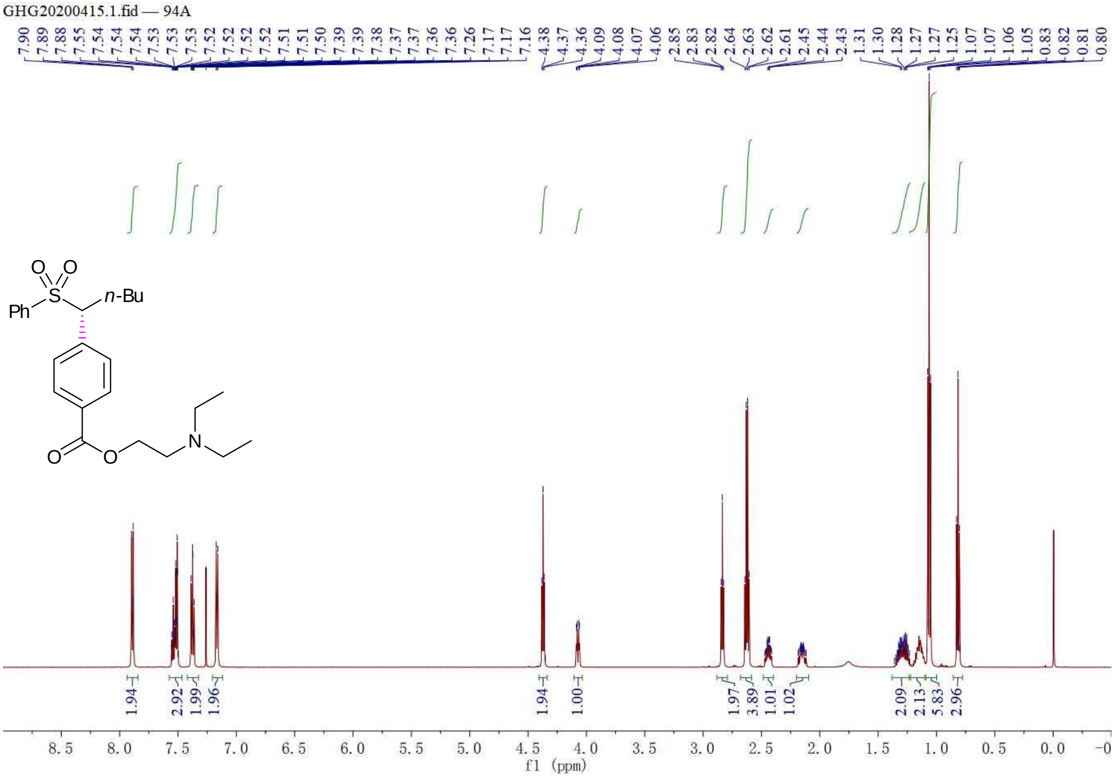

66, ^{13}C NMR (151 MHz, CDCl$_3$)

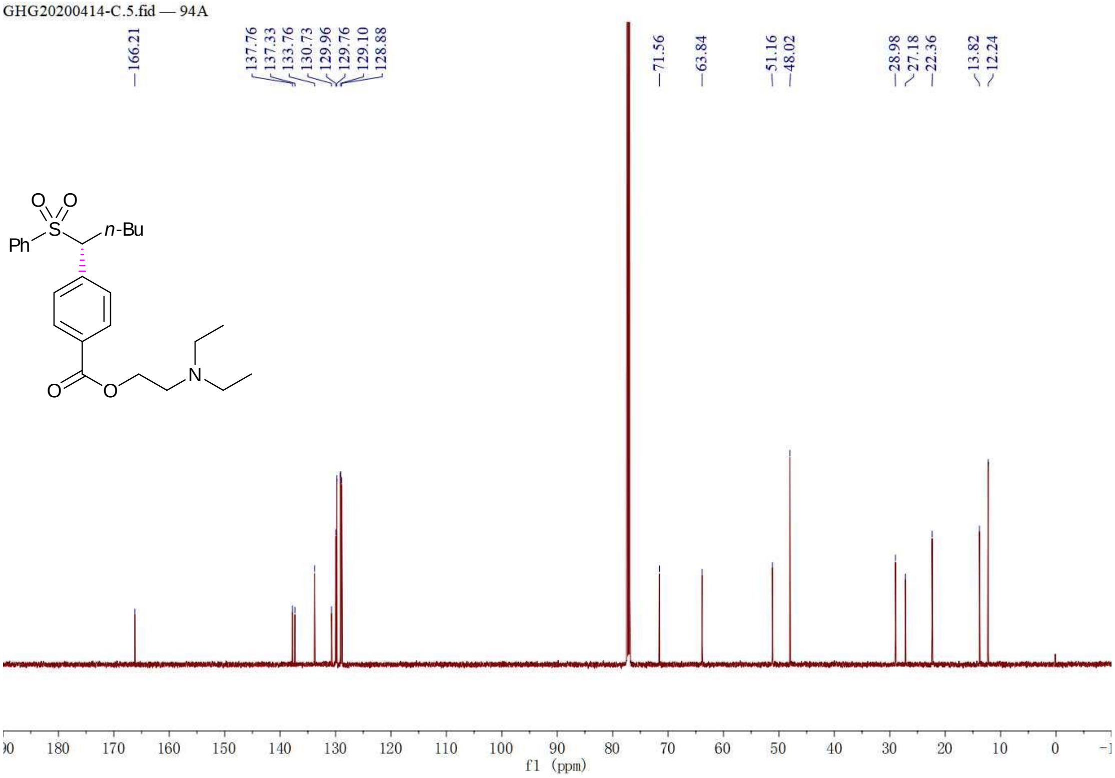

67, ^{1}H-NMR (600 MHZ, $CDCl_3$)

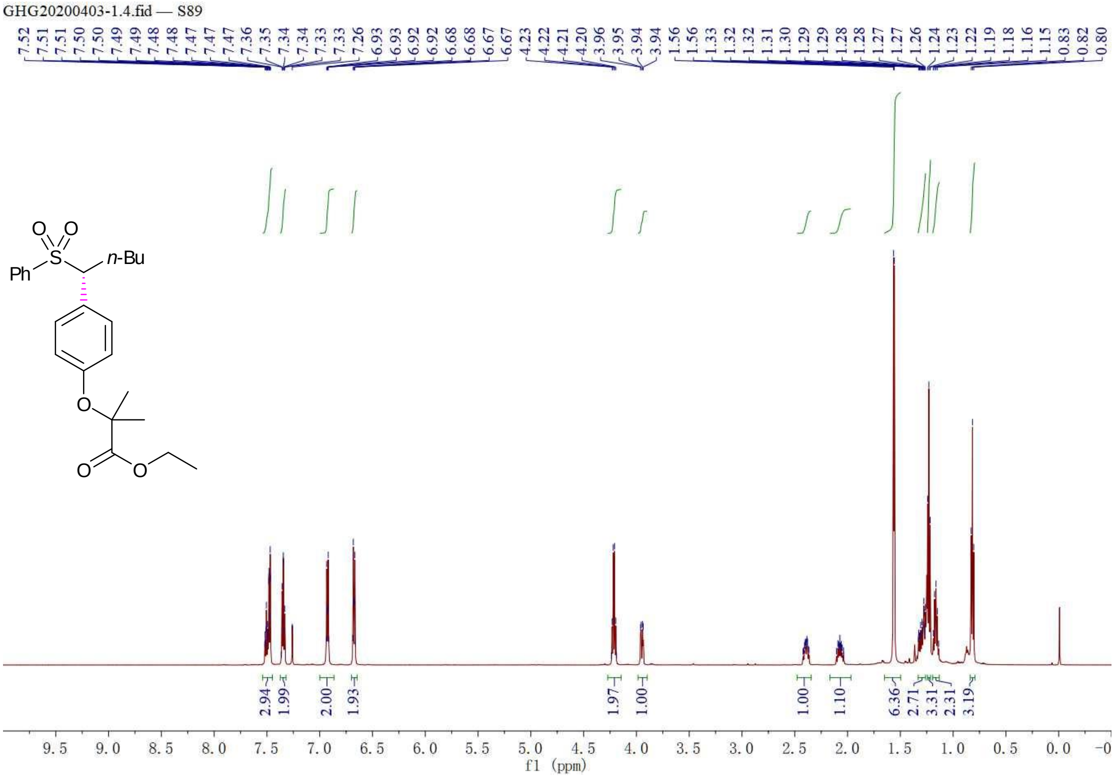

67, ^{13}C NMR (151 MHz, $CDCl_3$)

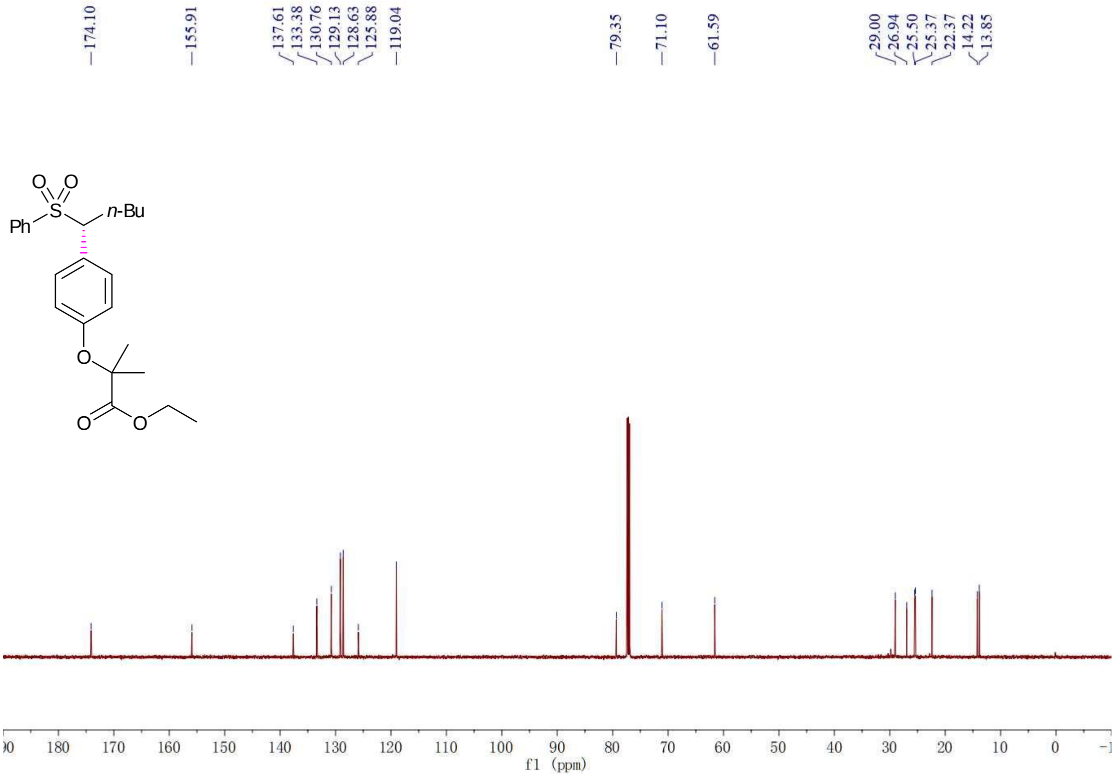

68, ^{1}H-NMR (600 MHZ, $CDCl_3$)

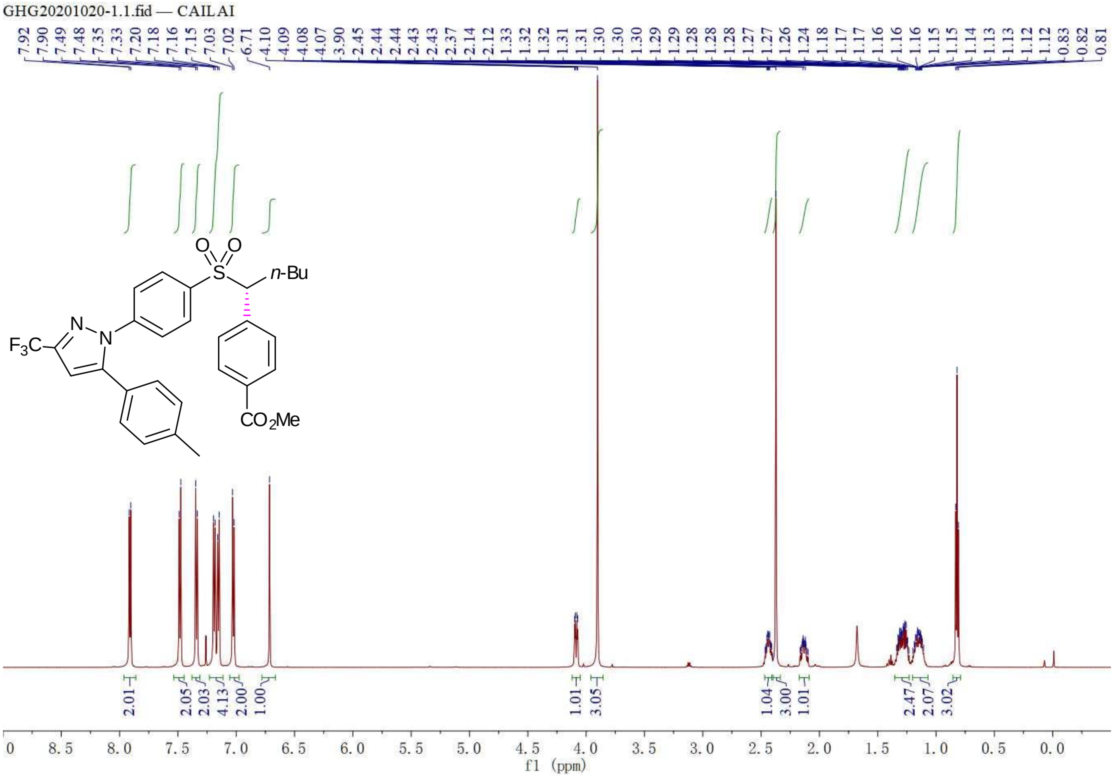

68, ^{13}C NMR (151 MHz, $CDCl_3$)

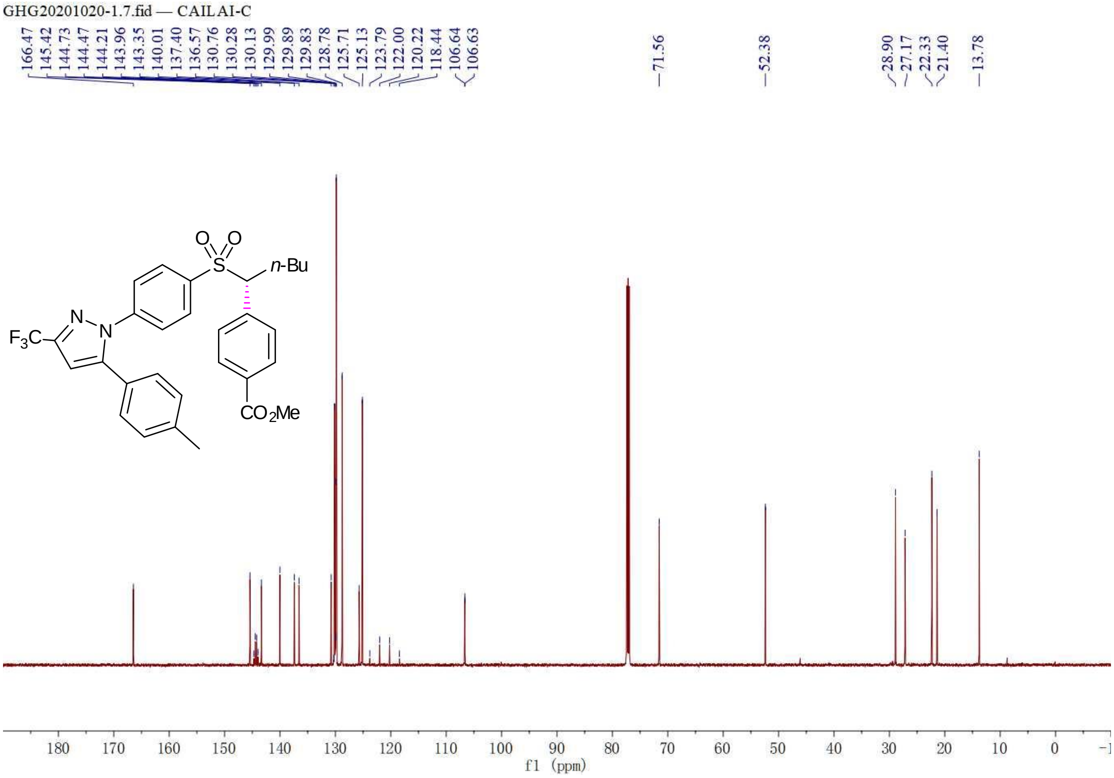

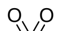

68, ^{19}F NMR (565 MHz, CDCl$_3$)

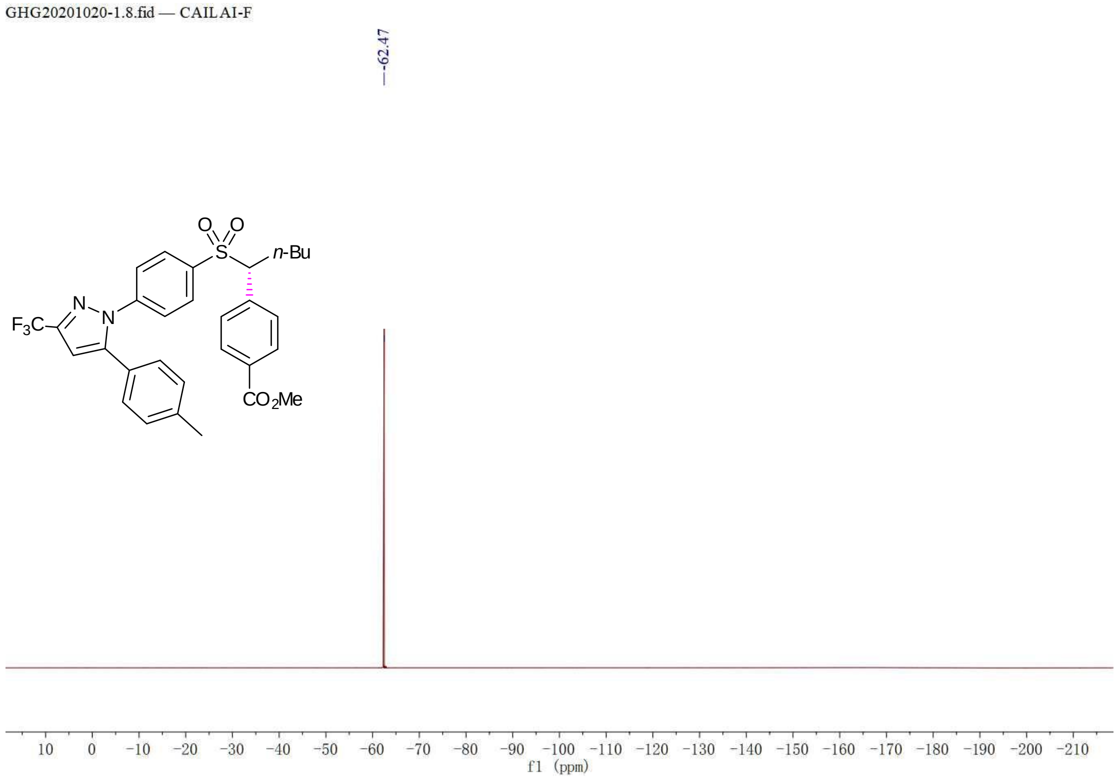

69, ^{1}H-NMR (600 MHZ, CDCl$_3$)

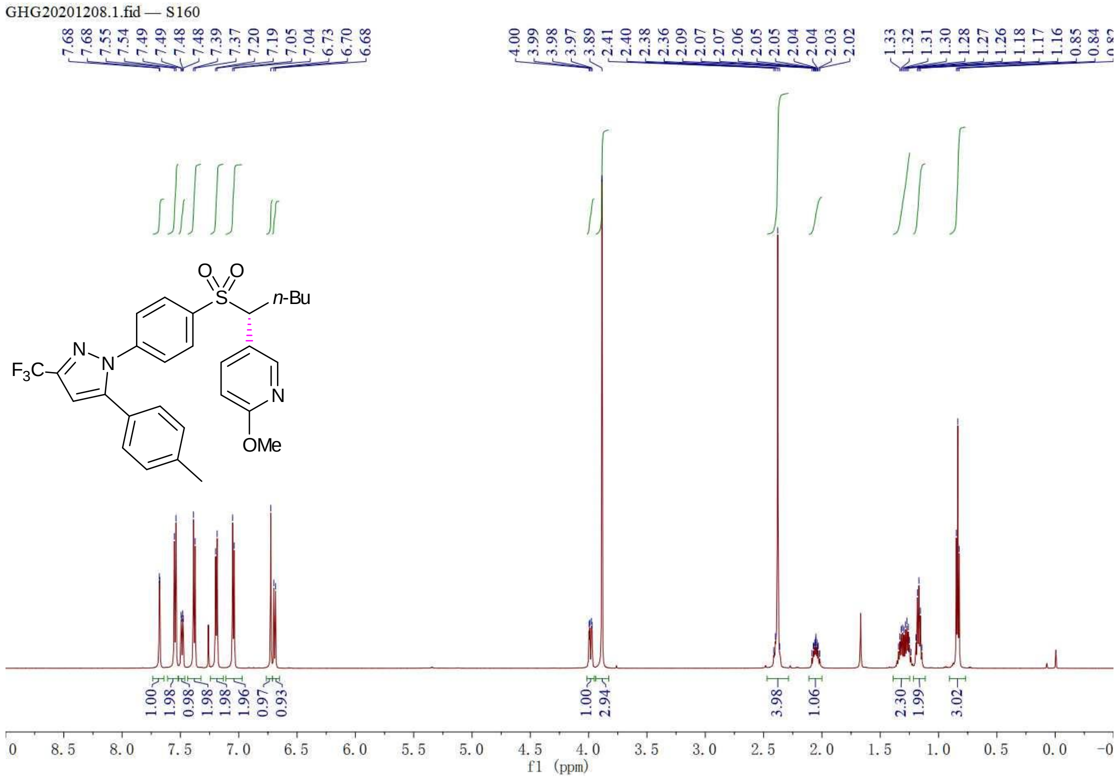

69, ^{13}C NMR (151 MHz, CDCl$_3$)

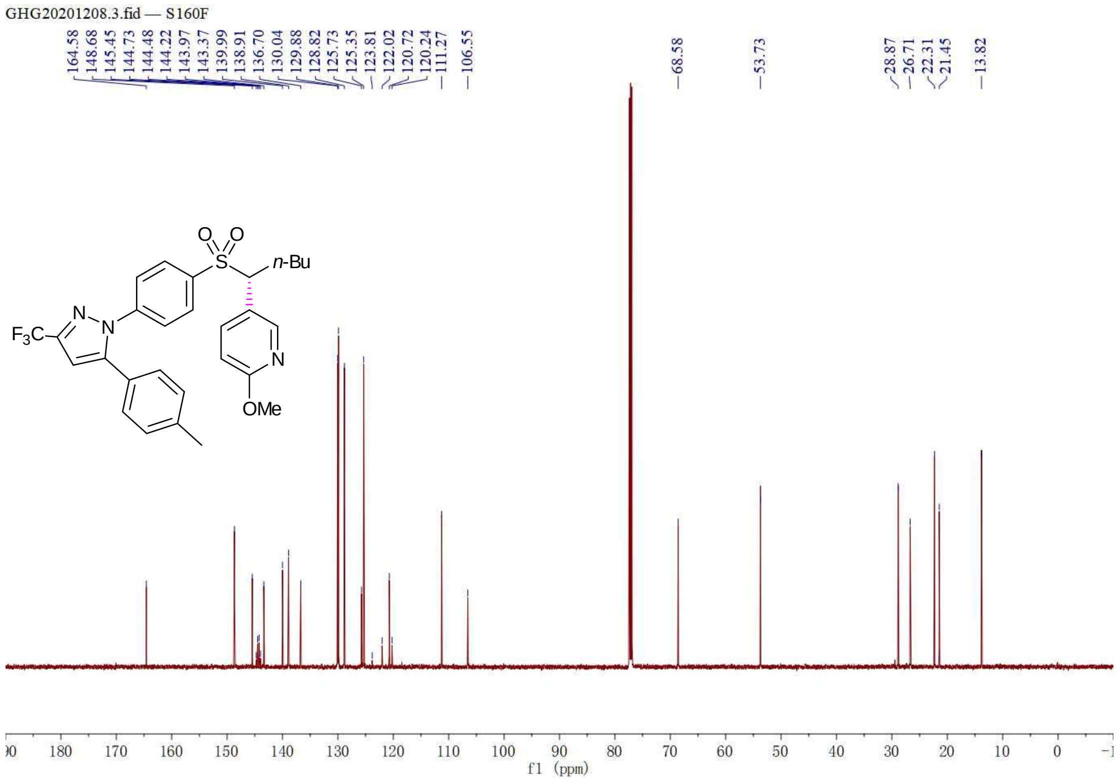

69, ^{19}F NMR (565 MHz, CDCl$_3$)

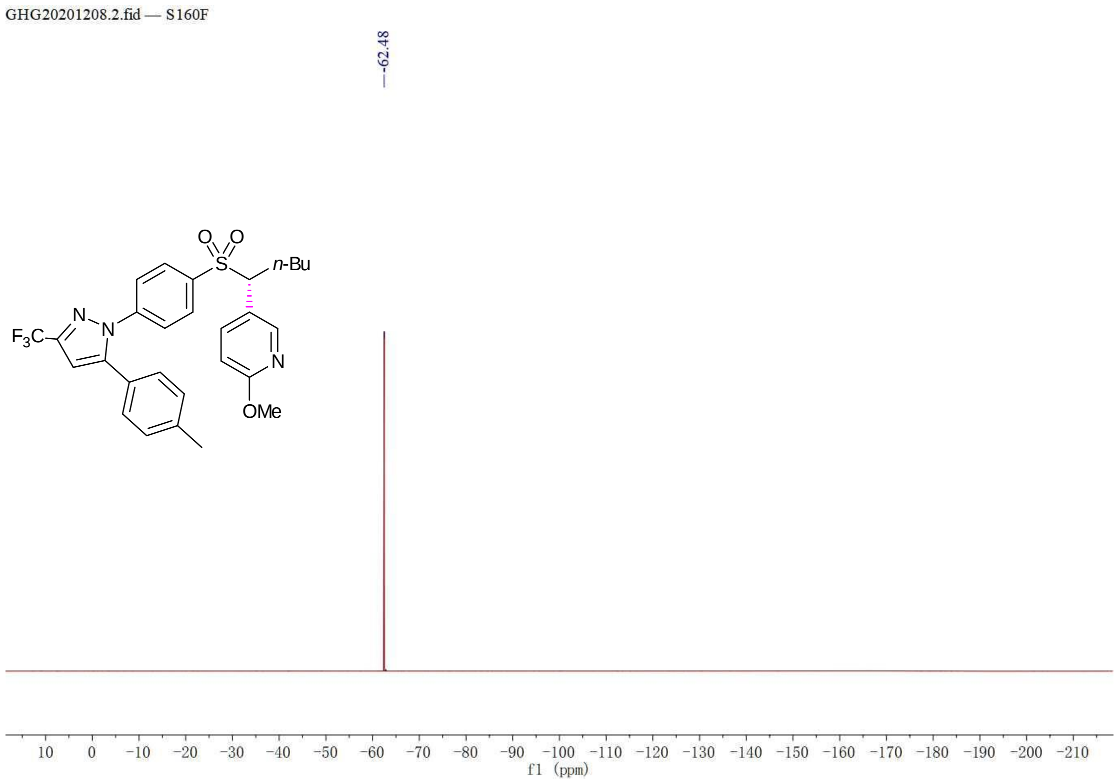

72, ^{1}H-NMR (600 MHZ, CDCl$_3$)

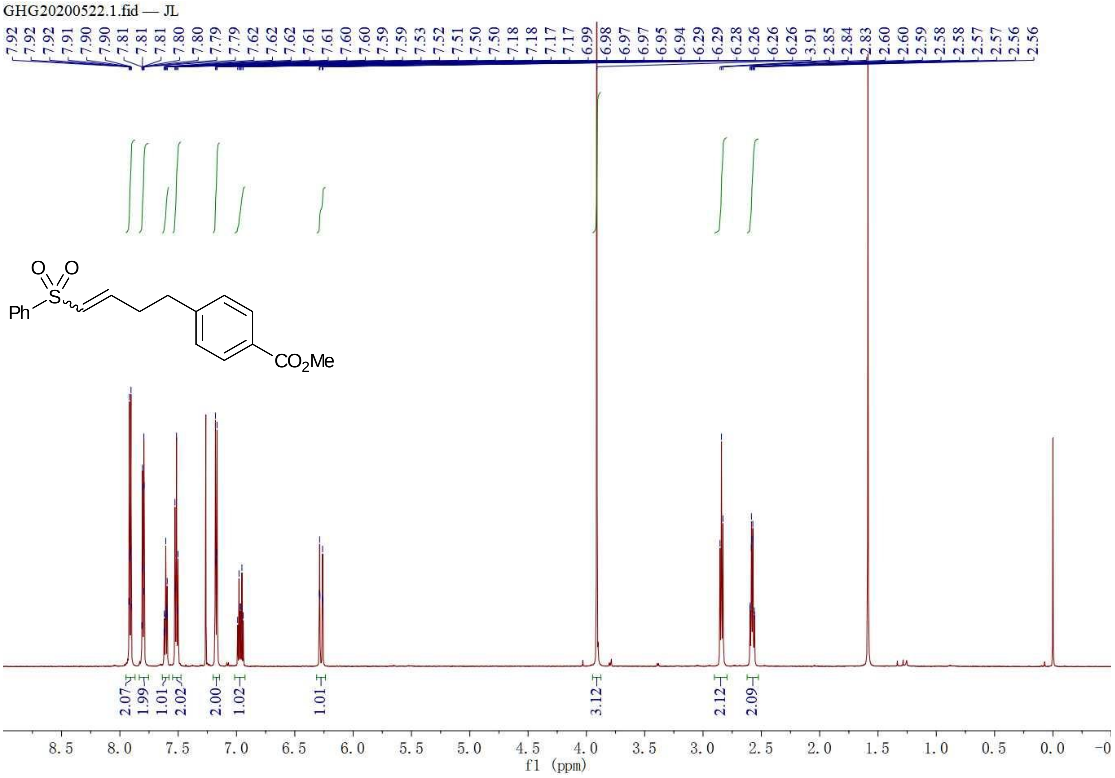

72, ^{13}C NMR (151 MHz, CDCl$_3$)

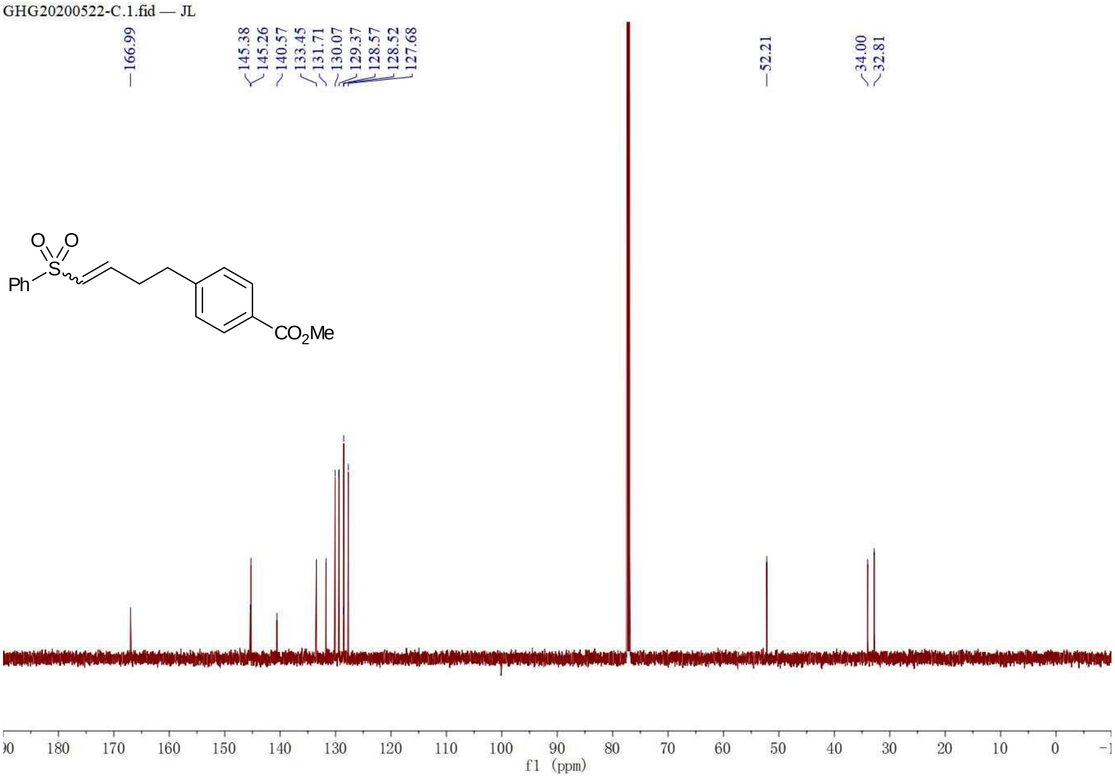

73, ^{1}H-NMR (600 MHZ, Acetone-d_6)

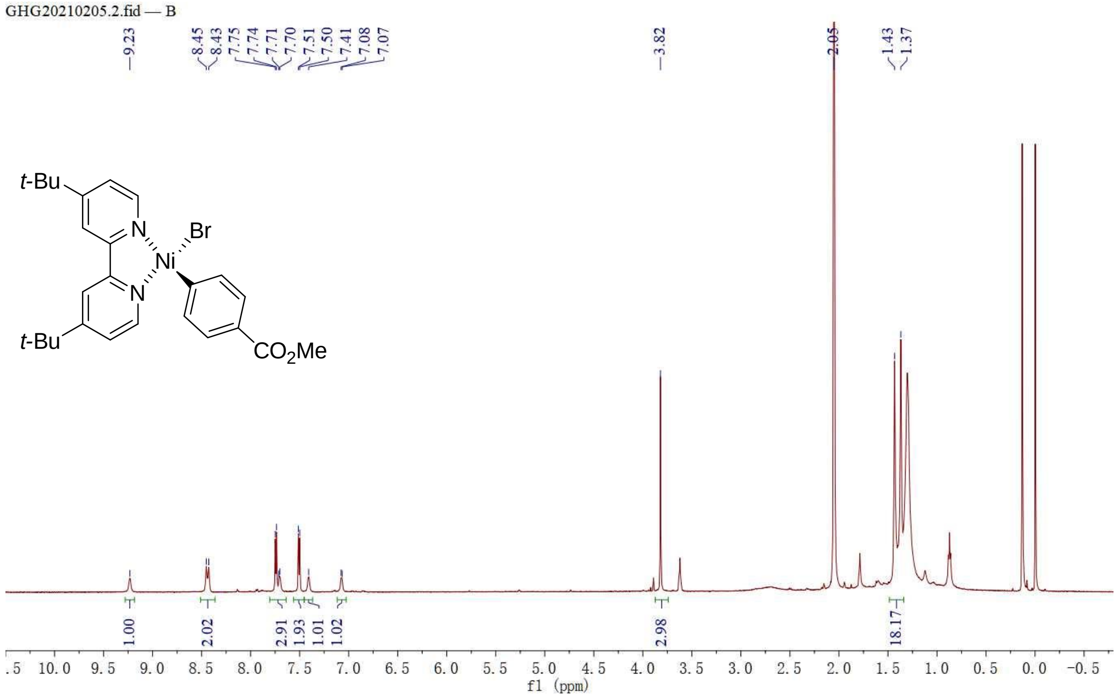

Supplement: SC-012-D1SC00283J-s001 [file SC-012-D1SC00283J-s001.pdf]
